# Identification of *NCAN* as a candidate gene for developmental dyslexia

**DOI:** 10.1038/s41598-017-10175-7

**Published:** 2017-08-24

**Authors:** Elisabet Einarsdottir, Myriam Peyrard-Janvid, Fahimeh Darki, Jetro J. Tuulari, Harri Merisaari, Linnea Karlsson, Noora M. Scheinin, Jani Saunavaara, Riitta Parkkola, Katri Kantojärvi, Antti-Jussi Ämmälä, Nancy Yiu-Lin Yu, Hans Matsson, Jaana Nopola-Hemmi, Hasse Karlsson, Tiina Paunio, Torkel Klingberg, Eira Leinonen, Juha Kere

**Affiliations:** 1 Folkhälsan Institute of Genetics, and Molecular Neurology Research Program, University of Helsinki, Helsinki, Finland; 2 Department of Biosciences and Nutrition, Karolinska Institutet, Huddinge, Sweden; 3 Department of Neuroscience, Karolinska Institutet, Solna, Sweden; 4 University of Turku, Institute of Clinical Medicine, Turku Brain and Mind Center, FinnBrain Birth Cohort Study, Turku, Finland; 5 Turku PET Centre, University of Turku, Turku, Finland; 6 University of Turku and Turku University Hospital, Department of Child Psychiatry, Turku, Finland; 7 University of Turku and Turku University Hospital, Department of Psychiatry, Turku, Finland; 8 Department of Medical Physics, Turku University Hospital, Turku, Finland; 9 Department of Radiology, Turku University Hospital and Turku University, 20520 Turku, Finland; 10 Genomics and Biomarkers Unit, National Institute for Health and Welfare, Helsinki, Finland; 11 Department of Psychiatry, University of Helsinki and Helsinki University Central Hospital, Helsinki, Finland; 12 Department of Paediatric Neurology, Helsinki University Central Hospital, Children’s Hospital, Helsinki, Finland; 13 Department of Medical & Molecular Genetics, King’s College London, Guy’s Hospital, London, UK

## Abstract

A whole-genome linkage analysis in a Finnish pedigree of eight cases with developmental dyslexia (DD) revealed several regions shared by the affected individuals. Analysis of coding variants from two affected individuals identified rs146011974G > A (Ala1039Thr), a rare variant within the *NCAN* gene co-segregating with DD in the pedigree. This variant prompted us to consider this gene as a putative candidate for DD. The RNA expression pattern of the *NCAN* gene in human tissues was highly correlated (R > 0.8) with that of the previously suggested DD susceptibility genes *KIAA0319*, *CTNND2*, *CNTNAP2* and *GRIN2B*. We investigated the association of common variation in *NCAN* to brain structures in two data sets: young adults (Brainchild study, Sweden) and infants (FinnBrain study, Finland). In young adults, we found associations between a common genetic variant in *NCAN*, rs1064395, and white matter volume in the left and right temporoparietal as well as the left inferior frontal brain regions. In infants, this same variant was found to be associated with cingulate and prefrontal grey matter volumes. Our results suggest *NCAN* as a new candidate gene for DD and indicate that *NCAN* variants affect brain structure.

## Introduction

Developmental dyslexia (DD) is characterized by literacy difficulties despite access to education, and normal vision, hearing and IQ^1^. Detection of chromosomal abnormalities such as balanced translocations in families has led to the identification of at least three DD susceptibility genes (*DYX1C1, ROBO1, CYP19A1*)^2–4^. This approach has also been used to identify genes implicated in learning difficulties similar to DD (e.g. *CTNND2*)^5^.

Linkage studies have led to identification of a number of other DD candidate genes, including *C2ORF3-GCFC2/MRPL19* ^6^, *DCDC2* ^7^, *KIAA0319* ^8^, *CNTNAP5* ^9^, *GRIN2B* ^10^, and *FOXP2* ^11^. Genome-wide association studies (GWAS), however, have been less fruitful. This may be due to low power, as DD is a heterogeneous phenotype. Despite large collaborations such as the NeuroDys study^12^, no such studies have been large enough to overcome this limitation. Family-based studies thus continue to be a powerful method of choice in DD. For example, exome-sequencing in such a family recently allowed us to identify the *CEP63* gene as a novel DD candidate gene^13^. A recent review by Mascheretti *et al*.^14^ gives a summary of the DD genes robustly supported by multiple studies as well as a number of highly interesting and plausible, but less studied putative DD candidates.

Previously, several studies have been performed to elucidate the connection between cognitive traits and variation in brain anatomy, e.g.^15–19^. As shown by Klingberg *et al*.^20^, white matter integrity in certain brain regions, such as the left temporoparietal cortex, plays a critical role in reading ability. This may be due to variation in the microstructure of white matter tracts, ultimately affecting communication between the cortical areas involved in reading and thus the processing of auditory and visual signals^20^.

Structural brain imaging has repeatedly highlighted certain regions in the left hemisphere correlated with DD, e.g.^5, 18, 21–23^, and white matter changes related to allelic variation in the DD susceptibility genes *DYX1C1*, *DCDC2* and *KIAA0319* are a recurring observation. Furthermore, multiple studies have shown correlation of variation in certain brain regions with genetic variants in specific genes, e.g. in *CEP63* ^13^, *SLC2A3* ^24^, and *NRSN1* ^25^. Dehaene *et al*., in their recent review, provide an overview of what is known regarding the mechanism of language acquisition and its impact on the brain^26^.

In the present study, we aimed to identify novel genes and variants co-segregating with DD in a large Finnish family. We combined initial prioritization of candidate genomic regions by genome-wide linkage analysis, with subsequent exome sequencing to identify putative risk variants. The results highlighted the *NCAN* gene as a plausible DD candidate. Subsequently, we explored the association of *NCAN* variants to brain volumes of infants and 6 to 25-year old typically developing participants.

## Material and Methods

All methods were carried out in accordance with the relevant guidelines and regulations.

### DD pedigree

The DD pedigree was originally recruited for the genetic study of dyslexia by Nopola-Hemmi *et al*.^27, 28^ from the Department of Paediatric Neurology at the Hospital for Children and Adolescents, University of Helsinki, Finland. The criteria for the probands (children under 16 years of age) included in the study were a severe reading impairment, an extended family history of dyslexia (at least four affected individuals) and a pedigree suggestive of autosomal dominant transmission. All individuals were Finnish, of North European/Caucasian origin and native Finnish speakers. Participants from the nuclear families and their first- and second degree relatives were interviewed by a pediatric neurologist or neuropsychologist and were sent a detailed questionnaire regarding their reading and spelling difficulties, school history, and remedial education.

All individuals included in the analyses (both affected and unaffected) were tested by a clinical neuropsychologist to verify the diagnosis of dyslexia. The diagnostic assessment included intelligence tests (WAIS-R, WISC-R), age- and grade-appropriate Finnish reading and spelling tests^29, 30^ and a neuropsychological test^31^. The criteria for dyslexia included a pronounced history of reading problems, remarkable deviation in age-related reading skills (depending on the age, at least two years) and a normal performance intelligence quotient (IQ > 85). The neurocognitive type of dyslexia segregating in the DD family consisted of deficits in phonological awareness, verbal short-term memory, and rapid naming. As the diagnoses in the family were performed more than 15 years ago, certain phenotypic information may have been lost or never collected. The protocol for diagnosing DD was, however, well according to the accepted approach at the time. Due to this, we here analyse only the binary DD affected/not affected phenotype in the current study.

**Figure 1:**
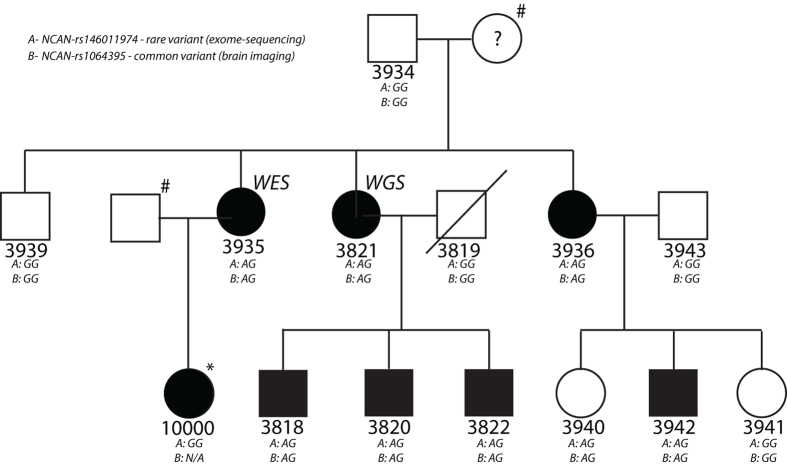
Pedigree of the presently studied family with developmental dyslexia (DD). Open boxes/circles indicate individuals without DD (confirmed no DD diagnosis), filled boxes/circles indicate individuals with confirmed DD. A question mark indicates an unknown phenotype. The exome-sequenced individual is marked with a “WES” and the whole-genome sequenced individual is marked with a “WGS”. The genotype of each individual for both *NCAN* markers is shown: (**A**) rs146011974 (a rare variant, identified through exome sequencing) and (**B**) rs1064395, (common variant, used in the brain imaging analysis). Individuals without DNA sample are indicated by the # symbol. Individual 10 000 (marked with a star) was not included in the linkage analysis. N/A = Genotype not available.

Genomic DNA from whole blood was available from 14 family members in the current study; 8 of them had confirmed DD (Fig. 1). Samples from the family were collected and analyzed according to ethical permissions Dnro 53/2006 and Dnro 4U/2016 by the ethics committee of the Central Finland Health Care District. Informed consent was obtained from all participating family members.

All available samples were genotyped on the Illumina HumanCoreExome12v1-0 genotyping chip (Illumina, San Diego, USA) and non-parametric multipoint linkage (NPL) analysis was run using Merlin version 1.12^32^ and the–exp option. The genotyping and linkage analysis are described in detail in Supplementary methods.

### Next generation sequencing

One hundred ng of genomic DNA from the affected individual 3935 was used for exome sequencing using an AmpliSeq library on the Ion Proton platform (Life Technologies, Carlsbad, CA) at the Uppsala Genome Center (Science for Life Laboratory, Uppsala University, Uppsala, Sweden). Variants identified using the Ion Torrent Suite software (Life Technologies) were filtered to retain only non-synonymous-, splice site- or stop codons variants. These were filtered further, to retain rare variants of < 1% frequency in the 1000 Genomes ALL dataset (all populations)^33^ and the ESP6500 dataset (all populations) within the regions of linkage (NPL > 1). The exome sequencing is described in more detail in Supplementary methods.

Two μg of genomic DNA from a second affected family member (3821) were used for whole-genome sequencing at Science for Life Laboratory, Stockholm, Sweden using a TruSeq library run on Illumina HiSeqX and the GATK best-practice pipeline. The sequencing is described in more detail in the Supplementary methods. As we hypothesized a highly penetrant autosomal dominant inheritance of DD in the family and a variant of strong risk-effect, we opted to extract only exonic variants and focused on variants shared by the two sequenced individuals, 3821 and 3935. The filtering steps were as above.

WES and WGS sequence data are available in the DDBJ/EMBL/GenBank databases under the accession number PRJEB12695. We used the human genome reference hg19 for all genomic positions of variants described here. Individuals 3935 and 3821 were selected for sequencing based on being affected carriers of the putative risk haplotype and due to the availability of high quality DNA.

### Gene expression correlation analysis

The Functional Annotation of the Mammalian Genome 5 (FANTOM5) human/mouse tissue and cell line promoter expression database^34^ was used to examine the expression of *NCAN* and compare it to the global expression levels of a representative set of DD candidate genes (*MRPL19, KIAA0319, ROBO1, CTNND2, DYX1C1, CEP63, KIAA0319L, PCNT, GCFC2, CYP19A1, DCDC2, FOXP2, CNTNAP2 and GRIN2B)* in 127 of the 152 tissue samples in the FANTOM5 dataset. We looked at the expression in all available human tissues (to get a general picture of the expression patterns), in brain tissue only (presumably yielding stronger correlation within the set of genes expressed primarily in neurological tissue), and in non-brain tissue (to further look into the expression patterns of the DD risk genes that are more ubiquitously expressed). Universal tissues, total RNA samples, and tissues with too shallow sequencing depths or low RNA quality were excluded from the analysis. The final dataset included 78 non-brain tissues and 49 brain tissues, listed in Supplementary Table 1. The ‘robust’ set of promoters was used for the gene-based analysis.

Spearman correlations (Rho) between the gene expressions were calculated for brain tissues, non-brain tissues, and all tissues (brain and non-brain tissues). As this non-parametric, ranking-based method gives R-values from −1 (complete negative correlation of ranks) to 1 (complete positive correlation of ranks), we here considered R-values of 0.8 or higher to indicate strong (positive) correlation. Hierarchical clustering was used to produce the correlation heatmaps.

### Brain imaging

As common variants in a number of previously identified DD susceptibility genes have shown significant associations with certain brain structures, we analyzed the potential association of a common variant in *NCAN* with structural variation in brain MRI data. We chose the polymorphism rs1064395 (NC_00019.10:g.19250926 G > A) in *NCAN*, a common variant previously associated with cognitive performance in healthy individuals^35^ and with grey matter volumes^36^. The minor alleles of rs1064395 and the rare variant rs146011974 (introduced further in Results) are carried on the same rare haplotype (data not shown) and therefore likely tag the same rare haplotype segregating with DD in the present family.

A longitudinal dataset (Brainchild) of brain imaging in 76 healthy Swedish children and young adults (age 6 to 25 years, 41 males and 35 females) was used to test for correlation of the *NCAN* genetic variant to white- and grey matter volumes. These same individuals have been studied previously^5, 13, 21, 22, 37^. Informed consent was obtained from all subjects (ethical approvals 2007/241–31/3 and 2012/116–32, Stockholm, Sweden); genomic DNA was analyzed using the Affymetrix Genome-wide SNP array 6.0 (Santa Clara, CA, USA). T1-weighted brain imaging was performed three times, each two years apart, by 3D magnetization prepared rapid gradient echo (MP-RAGE) sequence with TR = 2300 ms, TE = 2.92 ms, field of view of 256 × 256 mm^2^, 176 sagittal slices, and 1 mm^3^ voxel size. Diffeomorphic Anatomical Registration Through Exponentiated Lie Algebra (DARTEL)^38^ was performed to segment the brain into grey matter, white matter and CSF. An 8-mm Gaussian kernel was applied to the segmented white matter images. All modulated white and grey matter images were then analyzed using a flexible factorial design in Statistical Parametric Mapping (SPM) software (www.fil.ion.ucl.ac.uk/spm). The genetic variant rs1064395 was entered as a main factor, and the model was corrected for age, sex, handedness and total size of white matter volume. The gene interactions by age and sex were also added into the model. The main effect of the genetic variant on white matter structure was assessed by considering two thresholds (p = 0.01 and p = 0.001), and the clusters found significant at FDR corrected at the cluster level using nonstationary cluster extent correction^39^. The genotype counts for rs1064395 (*NCAN*) were 56 (CC) and 20 (CT). The frequency of the minor allele (T) of rs1064395 was 13% in the Brainchild dataset. To assess the overlap between the regions of white matter we overlaid the current *NCAN* findings at a threshold of p = 0.01 with the previous results from our imaging studies on DD/reading disability^5, 13, 21, 22, 37^ and susceptibility genes. Significant regions were overlaid on a human brain MRI template using MRIcro software (www.mccauslandcenter.sc.edu/mricr).

A second, independent dataset was used to explore the effect of rs1064395 on early structural characteristics of the brain. The FinnBrain Cohort is a Finnish general population-based pregnancy cohort where the main interest is to delineate the associations between maternal psychological well-being during pregnancy and the future development of the children (www.finnbrain.fi, Karlsson *et al*., submitted). The study was approved by the Ethics Committee of the Hospital District of Southwest Finland (ETMK: 31/180/2011 nr. 210) and informed consent was obtained from all subjects. The FinnBrain sub-sample available for the current study included 26 healthy, term-born infants (14 girls and 12 boys), imaged at mean age 22.5 days (SD 7.2) from birth. All MRI scans were performed at the Turku University Hospital using Siemens Magnetom Verio 3 T scanner (Siemens Medical Solutions, Erlangen, Germany). 12-element Head Matrix coil allowed the use of the Generalized Autocalibrating Partially Parallel Acquisition (GRAPPA) technique to accelerate acquisitions (PAT factor of 2 was used). 2D Dual Echo TSE (Turbo Spin Echo) sequence was used to acquire anatomical PD- and T2-weighted images. Parameters were optimized so the “whisper” gradient mode could be used in order to reduce acoustic noise during the scan. Slice thickness was 1 mm in order to acquire isotropic 1.0 × 1.0 × 1.0 mm voxels. TR time of 12070 ms and effective TE times of 13 ms and 102 ms were used to produce both PD- and T2-weighted images from the same acquisition. The total number of slices was 128. Only T2-weighted images were used in the subsequent analysis. Visual quality control (QC) was performed on the acquired volumes. Images were analyzed with iBEAT software^13^ that enables N3 bias correction, tissue segmentation and anatomical labeling (AAL atlas). iBEAT segmentation produces RAVENS maps that resemble modulated segmentation maps that are used in adult voxel-based morphometry (VBM) with more reliable metrics on highly abnormal brains (eg. infants). Subject to well- known uneven intensity distribution of the infant brain^14^, there were sub optimal results in segmenting the white matter, namely, portions of subcortical grey matter structures and myelinated central white matter appeared in grey matter segments. Correspondingly, white matter analysis in this data set was deemed unreliable and was not used in the analysis. Grey matter RAVENS maps were smoothed with 8 mm FWHM in SPM12 (http://www.fil.ion.ucl.ac.uk/spm/software/spm12/). Smoothed segmentations were entered into statistical modeling within the SPM. Similar to the Brainchild data set, we used a flexible factorial model, where the genetic variant rs1064395 was entered as a main factor and gestational age and sex as nuisance variables. The results were thresholded with two thresholds to provide an open view to the results: at p < 0.001 and with p < 0.01, FDR corrected (p < 0.05) for multiple comparison at the cluster level. The analysis was carried out in SPM and the MNI coordinates (in adult space) were used to identify the brain regions by overlaying the contrast on top of iBEAT AAL template with mricron.

In a complementary analysis, we used the individual tissue volumes that are produced in the iBEAT image processing pipelines. The volumes were calculated from the AAL template labeling and represent individual grey matter volumes (as contrasted to relative volumes/density of RAVENS maps).

The genotype counts for rs1064395 were 20 (CC) and 6 (CT). Genotyping was performed at the Estonian Genome Centre (Tartu, Estonia) on the Illumina Infinium PsychArray BeadChip. See Supplementary methods for further details on the analysis.

## Results

### Ten suggested regions of linkage to DD

We performed linkage analysis using single nucleotide variant genotype data. NPL plots and scores are shown in Supplementary Fig. S1 and Supplementary dataset S1, respectively. In the pedigree under study (Fig. 1), we identified ten genomic regions with NPL scores > 1 (Table 1) constituting the putative regions of interest in the subsequent exome data analysis. These regions did not overlap with the findings in Kaminen *et al*.^40^, where only a subset of the current pedigree was included (data not shown).

**Table 1:** Regions of NPL linkage > 1 in the DD pedigree. NPL = nonparametric lodscore.

Chromosome	max NPL	max NPL p-value	Locus	Size of locus (kb)
2	1.62	0.003	tel-rs6548285	963
	1.62	0.003	rs6706713-rs891881	5559
	1.62	0.003	rs1478644-rs12615297	855
3	1.62	0.003	rs11718068-rs6777084	3527
4	1.62	0.003	rs2353563-rs306364	16387
6	1.36	0.006	rs4896431-rs9376745	4246
8	2.63	0.0002	rs10088564-rs2527760	2879
18	1.36	0.006	rs695107-rs9950625	10608
19	1.36	0.006	rs11086080-rs1019937	12909
22	1.36	0.006	rs5750807-rs743377	2496

### Analysis of exome variants in the linkage regions

We extracted the coding variants in affected individuals 3935 and 3821 (Fig. 1). Table 2 shows the number of variants at each filtering step and in each region of linkage while Supplementary datasets S2 and S3 show the full list of these variants in 3935 and 3821, respectively. We identified only one rare non-synonymous variant within the regions of linkage that was shared by both individuals; it was located on chromosome 19. Sanger sequencing confirmed that all but one DD affected individual (10000) and one of the unaffected (3940), shared this variant (variant A in Fig. 1). This variant (rs146011974, NC_000019.10:g.19233884 G > A) is located within the *NCAN* gene (neurocan, also known as chondroitin sulfate proteoglycan 3, *CSPG3*), a gene expressed in multiple regions of the brain. It is predicted to result in an alanine to threonine change (NM_004386.2:c.3115 G > A (p.(Ala1039Thr)) and to be tolerated or probably benign by SIFT and Polyphen2, but possibly pathogenic by M-CAP^41^. Information on this variant has been submitted to the ClinVar database (www.ncbi.nlm.nih.gov/clinvar/), accession number SCV000330897. The alanine version of the protein is conserved in most mammals, including mouse, rat, chimp and cow. The minor allele (A) of rs146011974 has a frequency of 0.016% in the Genome Aggregation Database (gnomAD, gnomad.broadinstitute.org, accessed 7^th^ July 2017) all-populations dataset, 0.04% in the Finnish gnomAD population, 0.03% in the Sequencing Initiative Suomi (SISU database, www.sisuproject.fi, accessed 7^th^ July 2017), and it is not found at all in the 1000Genomes database. The rarity of this variant and the expression pattern of *NCAN* in human brain suggested that this variant might qualify as a candidate for DD susceptibility with high penetrance.

**Table 2:** Exome variants identified in the linkage regions at each stage of filtering. Ns = non-synonymous. WES = whole-exome sequencing. WGS = whole-genome sequencing.

		(WES)	(WGS)	
		**3935**	**3821**	
	**total variants**	48 833	4 058 706	
	PASS & depth > 9	48 015	3 578 878	
	exonic	20 484	24 646	
	exonic (ns/stop)	9 556	11 718	
	exonic (ns/stop) autosomal	9 389	11 501	
	<1% 1000Gall & ESP6500all autosomal	903	1304	
	**in linkage regions (b37)**	**3935**	**3821**	**in common**
2	tel-963334			
	50721511–56280428	1		
	70576997–71431885			
3	152631729–156158932			
4	115264835–131652041	1	1	
6	139411399–143657292			
8	2545224–5425042			
18	60597754–71205938	1	1	
19	17927041–30836248	14	2	**1** (*NCAN*)
22	22600912–25097248	1		

### Gene expression pattern correlations with other dyslexia candidate genes

**Figure 2:**
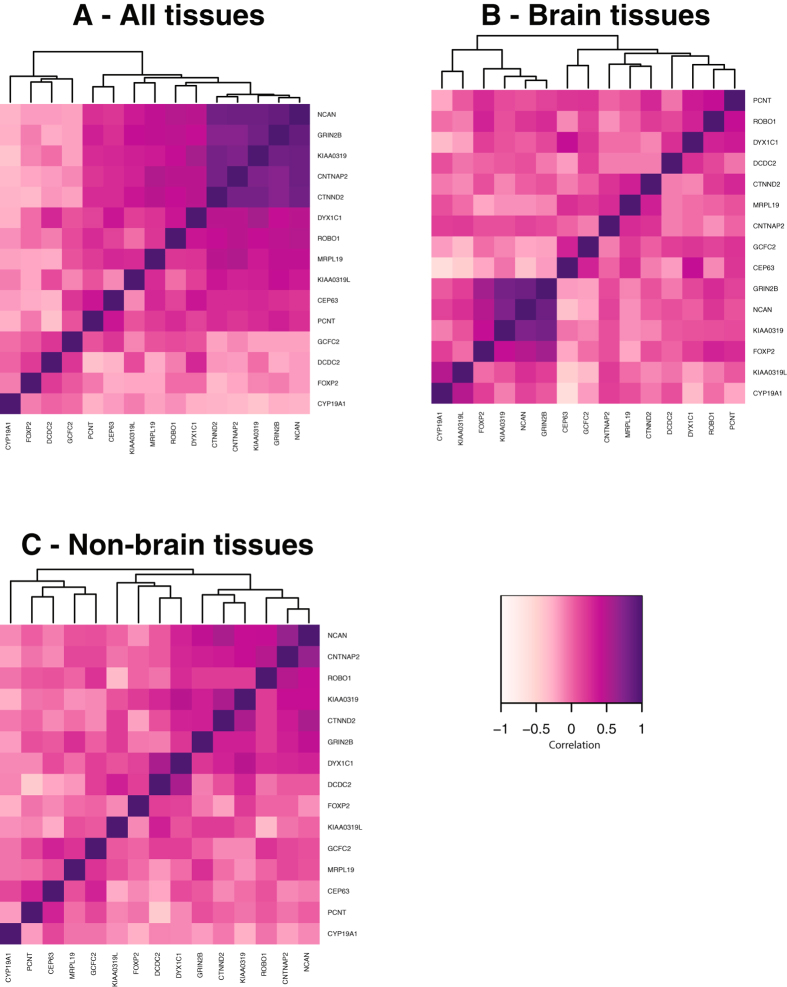
Correlation of mRNA expression of the dyslexia candidate genes from the FANTOM5 database. Spearman correlation of the global RNA expression of the previously described dyslexia candidate genes *KIAA0319*, *CTNND2*, *DYX1C1*, *ROBO1*, *MRPL19*, *KIAA0319L*, *CEP63*, *PCNT*, *GCFC2*, *DCDC2*, *CYP19A1*, *CNTNAP2*, *FOXP2* and *GRIN2B* as well as *NCAN*, the putative DD candidate gene identified in the present study. A dark purple color indicates strong positive correlation, while lighter color indicates negative correlation. (**A**) 127 tissue samples, (**B**) subset of 49 brain tissues and (**C**) subset of 78 non-brain tissues. Expression data was extracted from the FANTOM5 dataset, at http://fantom.gsc.riken.jp/5/data/.

In order to better understand the role *NCAN* may play in DD, we studied the correlation of *NCAN* expression with the expression profiles of a small subset of previously described known or candidate DD susceptibility genes across a wide selection of human tissues/organs. The aim was not to look systematically at all known or suggested DD genes, merely to compare *NCAN* to a subset of these and test if *NCAN* might make a plausible addition to this group. Figure 2 shows heat maps of these correlations. When all the tissue samples from the FANTOM5 dataset (Fig. 2A) were included in the analysis, we noted that *KIAA0319*, *CTNND2, CNTNAP2, GRIN2B* and *NCAN* showed strong correlation in global RNA expression levels (R > 0.80, Supplementary Table S2). A second cluster of putative DD genes comprised *DYX1C1*, *ROBO1*, *MRPL19*, *KIAA0319L*, *CEP63*, and *PCNT*. A further putative cluster of DD genes included *GCFC2*, *DCDC2*, *FOXP2* and *CYP19A1*.

For expression in brain only (Fig. 2B), we observed a strong correlation (R = 0.84) between *NCAN* and *GRIN2B*, and a somewhat weaker one between *NCAN* and *KIAA0319* (R = 0.75) (Supplementary Table S2). The correlations were weaker in non-brain tissues (Fig. 2C), the strongest being between *NCAN* and *CNTNAP2* (R = 0.65).

### Genetic variation and brain structure

We have previously presented genotyping and neuroimaging data indicating that common genetic variation in several DD candidate genes significantly associated with white or grey matter volumes^5, 13, 21, 22, 37^. In order to further investigate the role of *NCAN* in DD, we analyzed the association of a common genetic variant (rs1064395) in *NCAN* with structural white and grey matter variation in a longitudinal sample of 76 children and young adults (Brainchild dataset).

**Figure 3:**
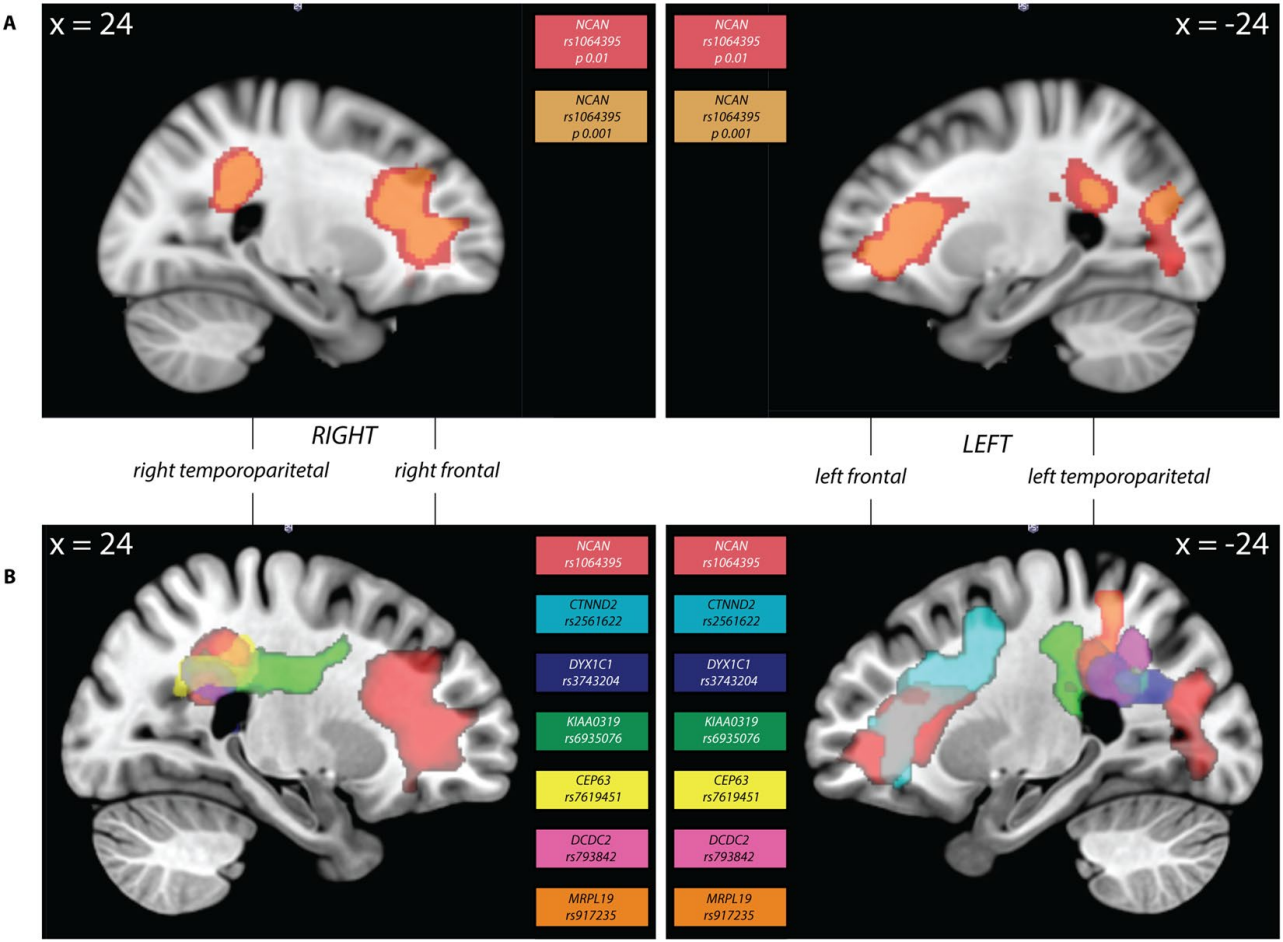
Common variation in DD susceptibility genes correlated with white matter volume. (**A**) Regions where white matter structure is significantly associated to common variation in *NCAN* (rs1064395; p < 0.01 shown in red and p < 0.001 shown in orange). (**B**) Summary of all the regions of white matter volume significantly associated to variation in *DYX1C1* (rs3743204, dark blue), *DCDC2* (rs793842, pink), *KIAA0319* (rs6935076, green), *MRPL19* (rs917235, purple), *CTNND2* (rs2561622, cyan), *CEP63* (rs7619451, yellow), and *NCAN* (rs1064395, red).

We found significant association of the minor allele of rs1064395 with increased white matter volume in the right temporoparietal and frontal regions (p = 0.01; p at cluster level: p _FWE corrected_ = 1.56 × 10^−6^) and the left temporoparietal, frontal and occipital regions (p = 0.01; p at cluster level p _FWE corrected_ = 4.48 × 10^−6^) (in red in Fig. 3A and Supplementary Table S3). We repeated the analysis with a lower p threshold to have fewer false positive voxels. The same clusters were significant at this threshold level (shown in orange in Fig. 3A and in Supplementary Table 4). The temporoparietal cluster partly overlapped with the superior longitudinal fasciculus, while the frontal and occipital clusters partly covered the inferior fronto-occipital fasciculus based on the John Hopkins University (JHU) white matter atlas^42^. Moreover, we found an association of the same allele with greater grey matter volume in the right superior temporal cortex (cluster level p _uncorrected_ = 0.009, peak coordinate: 68,−7,−5). However, this region did not survive FDR or FWE correction at the cluster level (cluster level p _FWE corrected_ = 0.22).

Previous studies have shown that the minor allele variants in several known DD candidate genes associate with larger white matter volume in certain brain regions^5, 13, 21, 22, 37^. We overlaid these brain regions with the *NCAN* regions from the present study. The right temporoparietal region associated with rs1064395 overlapped with a region previously associated with the DD susceptibility genes *KIAA0319*, *DYX1C1* and *MRPL19* ^21^ as well as *CEP63*, a more recently published DD susceptibility gene^13^ (Fig. 3B and Supplementary Table S3). In addition, the left frontal region associated with rs1064395 overlapped with a region associated with variation in *CTNND2*, another recent candidate DD susceptibility gene^5^. White matter volume in a third region, the left temporoparietal region, was associated with variation in the *DYX1C1*, *DCDC2* and *KIAA0319* genes^21^ as well as in the *MRPL19* gene^37^. In summary, these results showed that brain regions for which white matter volume associated with genetic variation in *NCAN* overlapped to a significant extent with those previously implicated for other DD susceptibility genes.

**Figure 4:**
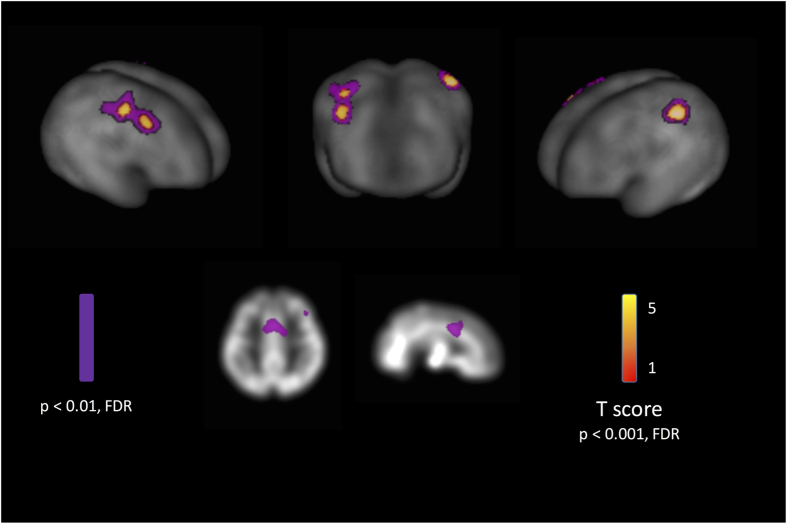
Association between the common *NCAN* genetic variant and infant grey matter structure (VBM). Upper panel - Cortical rendering of the results with thresholds p < 0.001 (red-to-yellow) and p < 0.01 (violet), FDR corrected for multiple comparisons. Lower panel - selected section from the same results, highlighting the cingulate associations.

To further explore the effect of the *NCAN* rs1064395 variant on brain structure, we looked at its associations with grey matter volumes in an independent set of infants (the FinnBrain study). We found that the minor allele (CT) of rs1064395 was associated with increased volume in infant grey matter in the left inferior parietal lobule, the right precentral gyrus and the right middle frontal gyrus. With a more lenient thresholding (p < 0.01) larger grey matter volumes associated to the minor allele in the bilateral middle cingulate cortices and superior frontal cortices ((Fig. 4, Supplementary Table S5). Regression analysis on volumes yielded results that are in line with the VBM analysis (Supplementary Table S6).

While we were unable to identify any putative DD variants within the linkage region on chromosome 8, it contains the 5’ and upstream regions of *CSMD1* (CUB and Sushi Multiple Domains 1) gene. Variants in *CSMD1* have been associated with disorders and traits affecting the central nervous system such as bipolar disorder disease^43^ and schizophrenia^44–46^; as well as general cognitive ability and executive function in healthy human subjects^47^. This gene may thus be of interest also in future studies of DD and related cognitive traits.

## Discussion

We used linkage analysis to prioritize potential candidate regions for autosomal dominant DD in a multiplex pedigree, followed up by evaluation of exome-sequencing data in those regions. This allowed us to highlight a new plausible candidate gene for DD, *NCAN*. Neither this gene nor genomic region have been previously suggested by linkage or GWA studies as a susceptibility locus or gene for dyslexia. While our average sequencing depth is satisfactory, we cannot exclude the existence of additional variants that would go unnoticed because of inexact base calling. Furthermore, even though the detected *NCAN* variant segregating with DD in the studied family leads to an amino-acid substitution, the true genetic risk variant(s) might conceivably be non-coding variation.

The rare *NCAN* variant identified in the current study is unlikely to explain a significant proportion of DD in the general population, and any studies of this specific variant are likely to be underpowered. This finding served, however, to highlight the *NCAN* gene as a potential novel DD susceptibility gene, and our subsequent work thus focused on understanding the general involvement of this gene in DD.

*NCAN* shares similar expression profiles with *CNTNAP2*, *CTNND2*, *KIAA0319* and *GRIN2B*, with higher expression in several brain regions and lower expressions in the rest of the body. This was reflected in the strong correlations seen in the expression correlation heatmaps. The specific functions of these genes and how their dysfunction might contribute to DD susceptibility are only partially understood. It is, however, tempting to speculate that these genes may represent a group of genes involved in shared mechanisms or pathways that are critical for the optimal development of the brain and its function in literacy skills. DD genes with different patterns of expression, e.g. the *CEP63* and *DCDC2* genes that are expressed in most of the tissues in the human body, are likely to contribute to DD risk through other, potentially less brain-specific, mechanisms.

NCAN is important for cell adhesion and neuronal cell migration and is a negative regulator of neurite outgrowth^48^. The rare variant rs146011974 lies within a highly conserved region in an EGF-like domain near the C -terminus of the protein. While it remains unclear how an amino acid change in this domain might affect DD risk, slight variation in signaling through this domain could plausibly influence communication of neurons with each other and their shared environment.

Carriers of the rare *NCAN* variant were unfortunately not available for imaging analysis. Instead, we looked further at how common variation in *NCAN* might affect white and grey matter volumes in two independent imaging datasets. The rationale behind this approach is that if a rare high-risk variant in *NCAN* drives DD susceptibility in the pedigree under study, common variants in the same gene might modulate brain structure in the general population. The common variant rs1064395 within the *NCAN* 3’ UTR here correlates with variation in white and grey matter structures and has previously been implicated in a number of psychiatric phenotypes such as bipolar disease^49^, schizophrenia^50^, cortical folding in schizophrenia^51^ and mania^52^. This is not the first time the same genetic variants have been connected to both dyslexia and other neurological disorders^53, 54^.

The white matter variability linked to *NCAN* in the Brainchild dataset was found in the left and right temporoparietal, occipital and frontal regions. Of these regions, the left inferior frontal area, with an extension to the region functionally defined as the Broca’s area, is known to be of importance for language processing, and overlaps with the region previously associated with *CTNND2* ^5^. The other cluster in the left temporoparietal region is also associated with several previously reported DD candidate brain regions^22, 37^. Disrupted brain activation patterns have also been reported in the left temporoparietal region when comparing poor readers to normal controls^55–57^. Moreover, both grey matter and white matter deviations in this region have been related to dyslexia and impaired reading^17, 20, 58^. A recent study^35^ showed association of rs1064395 with brain activation in the temporal lobe during a semantic verbal fluency task in healthy subjects. Previous studies have also reported associations of the same genetic variant with grey matter volumes of the hippocampus and amygdala^36^ as well as with volumes of the occipital region and prefrontal cortex^51^.

We went on to look further at this common *NCAN* variant in an independent dataset based on infant brain imaging. We were particularly interested in testing if the putative novel DD susceptibility gene might affect brain structure already at such a young age. We detected associations of grey matter volumes with *NCAN* variation in the right superior frontal cortex, inferior parietal and bilateral cingulate cortices in the infants. Interestingly, the direction of the effect was the same; the minor allele of rs1064395 was correlated with increased white matter volumes in the older imaging population and increased grey matter volumes in the infant dataset.

This suggests that *NCAN* may be important for efficient neuronal development, with the minor and putative DD risk allele of rs1064395 correlating with less efficient neuronal guidance. Such a defect has been suggested previously also with *DYX1C1*, *DCDC2*, *ROBO1* and *KIAA0319* (reviewed in ref. 8). Although the brain regions implicated are not language areas *per se*, the variation in them may modulate later cognitive performance in general^1^, and thus have intricate connections to reading, which requires a coordinated brain network function that directs attention^59^. It is possible that the white matter associations relate to the implicated initial grey matter characteristics, as the maturation of the white and grey matter in frontoparietal networks is strongly co-modulated^60^, but this remains to be studied with additional imaging techniques such as DTI. Of additional importance is the fact that the FinnBrain imaging study was performed soon after birth, minimizing the influence of postnatal environmental factors and thus yielding a possible insight into brain structural development as early as possible to *in utero*.

Although we were able to assess the genetic associations to brain morphology in a wide spectrum of ages (from infants to young adults), direct comparison of the findings from the two datasets is problematic and larger longitudinal datasets would be needed. Growing interest in low frequency variants also means that future studies need to be larger in order to attain an acceptable size for each genotype group.

The small size of both imaging datasets, the inherent limitations in studying only a common variant, and the questions arising recently about the validity of certain imaging studies^61^ and their power^62^, call for caution in drawing conclusions. However, with these datasets, we can lend robust support for the idea that *NCAN* is related to brain developmental processes and structural development at the more general level. How these findings directly relate to development of DD needs to be studied further.

In conclusion, we report here a family with multiple cases of DD individuals, and highlight *NCAN* as a putative novel DD susceptibility gene, which shares similar RNA expression profiles throughout multiple tissues within the human body with *KIAA0319*, *CTNND2*, *CNTNAP2* and *GRIN2B*. The brain imaging data reported in the present study support that *NCAN* variants have effects on brain structure from infancy to early adulthood, but the possible associations between genotype, neuroimaging and phenotype remain to be addressed in future studies.

## Electronic supplementary material

Supplementary information

Supplementary dataset 1

Supplementary dataset 2

Supplementary dataset 3
