# Supplementary material for: Identification of NCAN as a candidate gene for developmental dyslexia
Source: Sci Rep. 2017 Aug 24;7:9294. doi: 10.1038/s41598-017-10175-7 (PMC5570950; doi:10.1038/s41598-017-10175-7)
Supplement: Supplementary file 1 — Supplementary information [file 41598_2017_10175_MOESM1_ESM.pdf]

Supplementary Information for

Identification of *NCAN* as a candidate gene for developmental dyslexia

Authors
Elisabet Einarsdottir, Myriam Peyrard-Janvid, Fahimeh Darki, Jetro J. Tuulari, Harri Merisaari, Linnea Karlsson, Noora M. Scheinin, Jani Saunavaara, Riitta Parkkola, Katri Kantojärvi, Antti-Jussi Ämmälä, Nancy Yiu-Lin Yu, Hans Matsson, Jaana Nopola-Hemmi, Hasse Karlsson, Tiina Paunio, Torkel Klingberg, Eira Leinonen, Juha Kere

Supplementary methods

Supplementary table 1 – List of FANTOM5 human tissues used in correlation analysis. The brain-tissues and non-tissues together make up the "all tissues" set.

Supplementary table 2 - Spearman correlation (R) between the expression of known DD susceptibility genes and *NCAN*, as assessed from the FANTOM5 dataset. Values range from -1 (negative correlation) to 1 (positive correlation). The correlations in all tissues (A), brain tissues (B) and non-brain tissues (C) are shown. Correlations over 0.5 are highlighted in yellow, strong positive correlations (>0.8) are highlighted in orange.

Supplementary table 3 - Regions of white matter significantly associated with genetic variation in several known DD susceptibility genes and *NCAN* (Brainchild dataset).

Supplementary table 4 – Regions of white matter significantly associated with genetic variation in several known DD susceptibility genes and *NCAN* (Brainchild dataset) using a p 0.01 threshold.

Supplementary table 5 - Regions of grey matter and their association with genetic variation in *NCAN* (FinnBrain dataset).

Supplementary table 6 - MNI coordinates for the associations of *NCAN* variants to infant grey matter volumes. (FinnBrain dataset).

Supplementary figure 1 - All NPL graphs per chromosome
Non-parametric linkage (NPL) scores for each autosomal chromosome (1-22). The x-axis indicates the position along the chromosome in cM; the y-axis indicates NPL score (LOD). The K&C lin (linear) and exp (exponential) models of weighing are shown (black and blue lines, respectively).

Supplementary dataset 1 - All NPL values per chromosome
Non-parametric linkage scores (LOD) and the associated p-value for the linear and the exponential models of weighing, for each of the chromosomes 1-22. This data is presented graphically in Supplementary figure 1.

Supplementary dataset 2 - All stop/non-synonymous and rare genetic variants within the ten linkage regions of interest, as determined by exome sequencing of individual 3935.

Supplementary dataset 3- All stop/non-synonymous and rare genetic variants within the ten linkage regions of interest, as determined by exome sequence data obtained from individual 3821.

Supplementary Methods for

Identification of *NCAN* as a candidate gene for developmental dyslexia

Authors
Elisabet Einarsdottir, Myriam Peyrard-Janvid, Fahimeh Darki, Jetro J. Tuulari, Harri Merisaari, Linnea Karlsson, Noora M. Scheinin, Jani Saunavaara, Riitta Parkkola, Katri Kantojärvi, Antti-Jussi Ämmälä, Nancy Yiu-Lin Yu, Hans Matsson, Jaana Nopola-Hemmi, Hasse Karlsson, Tiina Paunio, Torkel Klingberg, Eira Leinonen, Juha Kere

Genotyping and linkage analysis:

Genotyping

Two µg of genomic DNA was used by the SNP&SEQ Technology Platform in Uppsala, Sweden for genotyping on Illumina HumanCoreExome12v1-0 genotyping arrays. This array contains assays for 243 345 variants. The genotyping was performed using the Illumina Infinium assay and the results were analyzed using the software GenomeStudio 2011.1 from Illumina (San Diego, US). Genotyping was performed based on cluster files generated from a set of 800 samples genotyped in other projects. Genome build 37 was used as reference. One CEPH control sample with known genotypes (from HapMap) was included in the genotyping. All data storage and the majority of subsequent analyses were performed through the BC|GENE system from BCPlatforms (www.bcplatforms.com, Espoo, Finland).

Quality control

Quality control of the data included removing all individuals with <99% call rate from the analysis, all markers with <95% call rate and any marker with any number of CEPH/HapMap inconsistencies. Subsequently, markers with a minor allele frequency of <5% in the dataset were removed from the analysis. Pedcheck [1] was run to identify inconsistencies in inheritance, identifying low quality genotypes as well as highlighting possible sample mix-ups or incorrectly defined family relationships. All markers showing any inheritance error in any sample were removed from the analysis. Unlikely genotypes were identified using the --

error option in Merlin v1.1.2 (www.csg.sph.umich.edu/abecasis/Merlin) [2]. Any such unlikely genotypes were also removed.

Linkage analysis

We pruned the genotype dataset to avoid inflation of linkage. PLINK v.1.07 [3], (www.pngu.mgh.harvard.edu/~purcell/plink) was used to allow no markers within a 50-marker sliding window to have an r^2 of more than 0.2. This reduced set of 28 085 markers was used for subsequent linkage analysis. The information content of the linkage analysis, using the pruned-in set of variants, was on average >95% (as assessed by the --information function of Merlin). The Rutgers genetic map v.3 (www.compgen.rutgers.edu/download_maps.shtml) [4] was used for the linkage analyses and any marker not found on this map was excluded from subsequent linkage analyses. The output of the analysis is presented as K&C LOD scores [5], hereafter called NPL, and accompanying p-values.

Library preparation, alignment and variant calling for next generation

Exome sequencing:

One hundred ng of genomic DNA from one affected individual was used for exome sequencing at the Uppsala Genome Center (Science for Life Laboratory, Uppsala University, Uppsala, Sweden). An AmpliSeq library was prepared and run on an Ion Proton (Life Technologies, Carlsbad, CA, US) instrument, according to standard protocols. The sequencing was performed on P1 chips, producing 200 bp reads.

Sequences were aligned to the hg19 genome assembly using the Ion Proton pipeline and single nucleotide variants (SNVs) were called using the Torrent Suite Software (Life Technologies). The total number of mapped reads was 52 784 338; 89.94% of these were on target. 91.94% of the bases were on target. The average coverage of the exons in 3935 was 135x. Target base coverage at 1x: 98.86%. Target base coverage at 20x: 93.45%. Exome variants were annotated using ANNOVAR 17, accessed through wANNOVAR at www.wannovar.usc.edu.

Whole-genome sequencing:

A TruSeq DNA library (350 bp insert PCR-free DNA) was prepared, followed by

sequencing on an Illumina HiSeqX machine in High Output mode, with PE 2x100bp fragments and average genomic sequencing depth of 30x. .

The WSG sample was analysed simultaneously with four other, idependent samples, run through the SciLife in-house Piper pipeline (BWA+GATK 3.5), available on Github (https://github.com/NationalGenomicsInfrastructure/piper). The total number of sequence reads was 1 187 654 947, of which 99.71% were successfully aligned to the human hg19 genome reference. The median insert size was 372bp, and the average autosomal coverage was 38x. 83.41% of the reference had 30x or higher coverage. Variant calling was performed using the current GATK best-practice guidelines [6] (with VQSR run on each single sample) and the list of variants was annotated using ANNOVAR.

Sanger sequencing

Sanger sequencing was used to validate the existence of the *NCAN* rs146011974 variant and assess its co-segregation with DD in the pedigree. Ten ng of DNA was amplified by PCR and sequenced by Eurofins Genomics (www.eurofinsgenomics.eu) according to standard protocols. Primers were designed using Primer3 (www.bioinfo.ut.ee/primer3), sequences are available upon request.

SUPPLEMENTARY REFERENCES

1 O'Connell, J. R. & Weeks, D. E. PedCheck: a program for identification of genotype incompatibilities in linkage analysis. *American journal of human genetics* **63**, 259-266, doi:10.1086/301904 (1998).

2 Abecasis, G. R., Cherny, S. S., Cookson, W. O. & Cardon, L. R. Merlin--rapid analysis of dense genetic maps using sparse gene flow trees. *Nature genetics* **30**, 97-101, doi:10.1038/ng786 (2002).

3 Purcell, S. *et al.* PLINK: a tool set for whole-genome association and population-based linkage analyses. *American journal of human genetics* **81**, 559-575, doi:10.1086/519795 (2007).

4 Matise, T. C. *et al.* A second-generation combined linkage physical map of the human genome. *Genome Res* **17**, 1783-1786, doi:10.1101/gr.7156307 (2007).
5 Kong, A. & Cox, N. J. Allele-sharing models: LOD scores and accurate linkage tests. *American journal of human genetics* **61**, 1179-1188, doi:10.1086/301592 (1997).
6 McKenna, A. *et al.* The Genome Analysis Toolkit: a MapReduce framework for analyzing next-generation DNA sequencing data. *Genome Res* **20**, 1297-1303, doi:10.1101/gr.107524.110 (2010).
7 Ashburner, J. A fast diffeomorphic image registration algorithm. *NeuroImage* **38**, 95-113, doi:10.1016/j.neuroimage.2007.07.007 (2007).
8 Hayasaka, S., Phan, K. L., Liberzon, I., Worsley, K. J. & Nichols, T. E. Nonstationary cluster-size inference with random field and permutation methods. *NeuroImage* **22**, 676-687, doi:10.1016/j.neuroimage.2004.01.041 (2004).
9 Darki, F., Peyrard-Janvid, M., Matsson, H., Kere, J. & Klingberg, T. Three dyslexia susceptibility genes, DYX1C1, DCDC2, and KIAA0319, affect temporo-parietal white matter structure. *Biological psychiatry* **72**, 671-676, doi:10.1016/j.biopsych.2012.05.008 (2012).
10 Darki, F., Peyrard-Janvid, M., Matsson, H., Kere, J. & Klingberg, T. DCDC2 polymorphism is associated with left temporoparietal gray and white matter structures during development. *The Journal of neuroscience : the official journal of the Society for Neuroscience* **34**, 14455-14462, doi:10.1523/JNEUROSCI.1216-14.2014 (2014).
11 Einarsdottir, E. *et al.* Mutation in CEP63 co-segregating with developmental dyslexia in a Swedish family. *Human genetics* **134**, 1239-1248, doi:10.1007/s00439-015-1602-1 (2015).
12 Hofmeister, W. *et al.* CTNND2-a candidate gene for reading problems and mild intellectual disability. *Journal of medical genetics* **52**, 111-122, doi:10.1136/jmedgenet-2014-102757 (2015).
13 Scerri, T. S. *et al.* The dyslexia candidate locus on 2p12 is associated with general cognitive ability and white matter structure. *PloS one* **7**, e50321, doi:10.1371/journal.pone.0050321 (2012).

Supplementary table 1 - FANTOM5 tissues

Brain-tissues	Non-brain tissues
amygdala_,_adult,_donor10196	adipose_tissue,_adult,_pool1
amygdala,_adult,_donor10252	aorta,_adult,_pool1
brain,_adult,_donor1	appendix,_adult
brain,_adult,_pool1	bladder,_adult,_pool1
caudate_nucleus_,_adult,_donor10196	bone_marrow,_adult
caudate_nucleus,_adult,_donor10252	breast,_adult,_donor1
cerebellum,_adult,_donor10252	cerebrospinal_fluid,_donor2
cerebellum,_adult,_pool1	cervix,_adult,_pool1
cerebral_meninges,_adult	colon,_adult,_donor1
corpus_callosum,_adult,_pool1	colon,_adult,_pool1
dura_mater,_adult,_donor1	colon,_fetal,_donor1
frontal_lobe,_adult,_pool1	cruciate_ligament,_donor2
globus_pallidus_,_adult,_donor10196	diaphragm,_fetal,_donor1
globus_pallidus,_adult,_donor10252	diencephalon,_adult
hippocampus_,_adult,_donor10196	ductus_deferens,_adult
hippocampus,_adult,_donor10252	duodenum,_fetal,_donor1_rep2
insula,_adult,_pool1	epididymis,_adult
locus_coeruleus_,_adult,_donor10196	esophagus,_adult,_pool1
locus_coeruleus,_adult,_donor10252	eye,_fetal,_donor1
medial_frontal_gyrus_,_adult,_donor10196	Fingernail_,including_nail_plate,_eponychium_and_hyponychium,_donor2
medial_temporal_gyrus_,_adult,_donor10196	gall_bladder,_adult
medial_temporal_gyrus,_adult,_donor10252	heart_,_mitral_valve,_adult
medulla_oblongata_,_adult,_donor10196	heart_,_pulmonic_valve,_adult
medulla_oblongata,_adult,_donor10252	heart_,_tricuspid_valve,_adult
medulla_oblongata,_adult,_pool1	heart,_adult,_diseased_post,infarction,_donor1
middle_temporal_gyrus,_donor10252	heart,_adult,_diseased,_donor1
nucleus_accumbens,_adult,_pool1	heart,_adult,_pool1
occipital_cortex,_adult,_donor10252	heart,_fetal,_pool1
occipital_lobe,_adult,_donor1	kidney,_adult,_pool1
occipital_lobe,_fetal,_donor1	kidney,_fetal,_pool1
occipital_pole,_adult,_pool1	left_atrium,_adult,_donor1
paracentral_gyrus,_adult,_pool1	left_ventricle,_adult,_donor1
parietal_lobe_,_adult,_donor10196	liver,_adult,_pool1
parietal_lobe,_adult,_donor10252	lung,_adult,_pool1
parietal_lobe,_adult,_pool1	lung,_fetal,_donor1
parietal_lobe,_fetal,_donor1	lung,_right_lower_lobe,_adult,_donor1
pineal_gland_,_adult,_donor10196	lymph_node,_adult,_donor1
pineal_gland,_adult,_donor10252	olfactory_region,_adult
pituitary_gland_,_adult,_donor10196	ovary,_adult,_pool1
pituitary_gland,_adult,_donor10252	pancreas,_adult,_donor1
pons,_adult,_pool1	parotid_gland,_adult
postcentral_gyrus,_adult,_pool1	penis,_adult
putamen,_adult,_donor10196	placenta,_adult,_pool1
substantia_nigra,_adult,_donor10252	prostate,_adult,_pool1
temporal_lobe,_adult,_pool1	rectum,_fetal,_donor1
temporal_lobe,_fetal,_donor1	retina,_adult,_pool1
temporal_lobe,_fetal,_donor1_rep2	salivary_gland,_adult,_pool1
thalamus_,_adult,_donor10196	seminal_vesicle,_adult
thalamus,_adult,_donor10252	skeletal_muscle_,_soleus_muscle,_donor1
	skeletal_muscle,_adult,_pool1
	skin,_fetal,_donor1
	small_intestine,_adult,_pool1
	small_intestine,_fetal,_donor1
	smooth_muscle,_adult,_pool1
	spinal_cord_,_adult,_donor10196
	spinal_cord,_adult,_donor10252
	spinal_cord,_fetal,_donor1
	spleen,_adult,_pool1
	spleen,_fetal,_pool1
	stomach,_fetal,_donor1
	submaxillary_gland,_adult
	testis,_adult,_pool1
	testis,_adult,_pool2
	throat,_adult
	throat,_fetal,_donor1
	thymus,_adult,_pool1
	thymus,_fetal,_pool1
	thyroid,_adult,_pool1
	thyroid,_fetal,_donor1
	tongue,_adult
	tongue,_fetal,_donor1
	tonsil,_adult,_pool1
	trachea,_adult,_pool1
	trachea,_fetal,_donor1
	uterus,_adult,_pool1
	uterus,_fetal,_donor1
	vagina,_adult
	vein,_adult

Supplementary table S2 - Spearman correlations of a set of representative DD genes

A - ALL TISSUES

	GCFC2	MRPL19	FOXP2	NCAN	KIAA0319	CYP19A1	KIAA0319L	DCDC2	PCNT	ROBO1	CTNND2	CNTNAP2	CEP63	DYX1C1	GRIN2B
**GCFC2**	*1*	0,070	0,041	-0,201	-0,217	-0,026	-0,110	0,218	0,075	0,118	-0,212	-0,134	0,207	0,075	-0,215
**MRPL19**	0,070	*1*	-0,221	0,430	0,354	-0,203	0,265	-0,247	0,187	0,203	0,471	0,503	0,294	0,237	0,435
**FOXP2**	0,041	-0,221	*1*	-0,183	-0,103	-0,099	-0,231	0,197	-0,072	-0,006	-0,320	-0,227	-0,247	-0,007	-0,088
**NCAN**	-0,201	0,430	-0,183	*1*	0,824	-0,282	0,343	-0,188	0,254	0,486	0,806	0,837	0,205	0,467	0,872
**KIAA0319**	-0,217	0,354	-0,103	0,824	*1*	-0,391	0,338	-0,030	0,264	0,411	0,793	0,754	0,280	0,577	0,813
**CYP19A1**	-0,026	-0,203	-0,099	-0,282	-0,391	*1*	-0,088	0,002	-0,272	-0,149	-0,290	-0,309	-0,188	-0,277	-0,263
**KIAA0319L**	-0,110	0,265	-0,231	0,343	0,338	-0,088	*1*	0,111	0,073	-0,031	0,361	0,311	-0,124	0,179	0,407
**DCDC2**	0,218	-0,247	0,197	-0,188	-0,030	0,002	0,111	*1*	-0,361	-0,093	-0,174	-0,186	-0,283	0,294	-0,247
**PCNT**	0,075	0,187	-0,072	0,254	0,264	-0,272	0,073	-0,361	*1*	0,264	0,272	0,219	0,366	0,159	0,321
**ROBO1**	0,118	0,203	-0,006	0,486	0,411	-0,149	-0,031	-0,093	0,264	*1*	0,417	0,467	0,153	0,362	0,449
**CTNND2**	-0,212	0,471	-0,320	0,806	0,793	-0,290	0,361	-0,174	0,272	0,417	*1*	0,784	0,271	0,473	0,716
**CNTNAP2**	-0,134	0,503	-0,227	0,837	0,754	-0,309	0,311	-0,186	0,219	0,467	0,784	*1*	0,272	0,476	0,717
**CEP63**	0,207	0,294	-0,247	0,205	0,280	-0,188	-0,124	-0,283	0,366	0,153	0,271	0,272	*1*	0,369	0,244
**DYX1C1**	0,075	0,237	-0,007	0,467	0,577	-0,277	0,179	0,294	0,159	0,362	0,473	0,476	0,369	*1*	0,406
**GRIN2B**	-0,215	0,435	-0,088	0,872	0,813	-0,263	0,407	-0,247	0,321	0,449	0,716	0,717	0,244	0,406	*1*

B -BRAIN TISSUES

	GCFC2	MRPL19	FOXP2	NCAN	KIAA0319	CYP19A1	KIAA0319L	DCDC2	PCNT	ROBO1	CTNND2	CNTNAP2	CEP63	DYX1C1	GRIN2B
**GCFC2**	*1*	0,194	-0,030	-0,233	-0,110	-0,192	-0,328	0,166	0,239	0,156	0,028	-0,093	0,318	0,203	-0,209
**MRPL19**	0,194	*1*	-0,234	-0,150	-0,156	0,086	-0,011	-0,081	0,073	0,013	0,326	0,279	0,246	-0,033	-0,102
**FOXP2**	-0,030	-0,234	*1*	0,489	0,403	0,162	-0,001	0,011	0,278	0,320	0,085	0,083	-0,229	0,171	0,599
**NCAN**	-0,233	-0,150	0,489	*1*	0,746	0,140	0,244	0,089	0,077	0,215	0,036	0,141	-0,369	0,023	0,836
**KIAA0319**	-0,110	-0,156	0,403	0,746	*1*	-0,089	0,077	0,054	0,119	0,094	-0,107	0,089	-0,055	0,078	0,784
**CYP19A1**	-0,192	0,086	0,162	0,140	-0,089	*1*	0,484	0,084	-0,227	-0,021	-0,067	0,162	-0,586	-0,324	0,045
**KIAA0319L**	-0,328	-0,011	-0,001	0,244	0,077	0,484	*1*	-0,055	0,056	-0,092	-0,124	0,183	-0,449	-0,219	0,162
**DCDC2**	0,166	-0,081	0,011	0,089	0,054	0,084	-0,055	*1*	-0,060	0,126	-0,097	-0,087	-0,187	0,241	-0,070
**PCNT**	0,239	0,073	0,278	0,077	0,119	-0,227	0,056	-0,060	*1*	0,417	0,285	0,008	0,213	0,356	0,147
**ROBO1**	0,156	0,013	0,320	0,215	0,094	-0,021	-0,092	0,126	0,417	*1*	0,196	-0,141	-0,128	0,314	0,275
**CTNND2**	0,028	0,326	0,085	0,036	-0,107	-0,067	-0,124	-0,097	0,285	0,196	*1*	0,231	-0,087	-0,106	0,077
**CNTNAP2**	-0,093	0,279	0,083	0,141	0,089	0,162	0,183	-0,087	0,008	-0,141	0,231	*1*	0,017	-0,026	0,105
**CEP63**	0,318	0,246	-0,229	-0,369	-0,055	-0,586	-0,449	-0,187	0,213	-0,128	-0,087	0,017	*1*	0,408	-0,193
**DYX1C1**	0,203	-0,033	0,171	0,023	0,078	-0,324	-0,219	0,241	0,356	0,314	-0,106	-0,026	0,408	*1*	0,127
**GRIN2B**	-0,209	-0,102	0,599	0,836	0,784	0,045	0,162	-0,070	0,147	0,275	0,077	0,105	-0,193	0,127	*1*

C -NON-BRAIN TISSUES

	GCFC2	MRPL19	FOXP2	NCAN	KIAA0319	CYP19A1	KIAA0319L	DCDC2	PCNT	ROBO1	CTNND2	CNTNAP2	CEP63	DYX1C1	GRIN2B
**GCFC2**	*1*	0,226	0,002	0,088	-0,121	-0,056	0,044	0,174	0,131	0,223	-0,123	0,135	0,311	0,170	0,028
**MRPL19**	0,226	*1*	-0,016	0,070	-0,148	-0,059	0,083	-0,176	-0,027	0,012	-0,022	0,121	0,082	-0,003	0,255
**FOXP2**	0,002	-0,016	*1*	-0,145	0,182	-0,280	-0,132	0,172	-0,053	0,004	-0,201	0,014	-0,128	0,141	-0,066
**NCAN**	0,088	0,070	-0,145	*1*	0,398	-0,122	0,016	0,041	0,020	0,417	0,544	0,651	-0,083	0,312	0,440
**KIAA0319**	-0,121	-0,148	0,182	0,398	*1*	-0,271	0,071	0,284	-0,047	0,181	0,535	0,394	-0,032	0,476	0,325
**CYP19A1**	-0,056	-0,059	-0,280	-0,122	-0,271	*1*	-0,148	-0,102	-0,198	-0,057	-0,080	-0,213	0,127	-0,128	-0,181
**KIAA0319L**	0,044	0,083	-0,132	0,016	0,071	-0,148	*1*	0,333	-0,113	-0,335	0,196	-0,045	-0,250	0,065	0,185
**DCDC2**	0,174	-0,176	0,172	0,041	0,284	-0,102	0,333	*1*	-0,428	-0,057	0,091	0,050	-0,232	0,541	-0,087
**PCNT**	0,131	-0,027	-0,053	0,020	-0,047	-0,198	-0,113	-0,428	*1*	0,059	0,008	-0,060	0,306	-0,129	0,088
**ROBO1**	0,223	0,012	0,004	0,417	0,181	-0,057	-0,335	-0,057	0,059	*1*	0,181	0,482	0,071	0,280	0,194
**CTNND2**	-0,123	-0,022	-0,201	0,544	0,535	-0,080	0,196	0,091	0,008	0,181	*1*	0,352	-0,131	0,303	0,321
**CNTNAP2**	0,135	0,121	0,014	0,651	0,394	-0,213	-0,045	0,050	-0,060	0,482	0,352	*1*	-0,138	0,295	0,322
**CEP63**	0,311	0,082	-0,128	-0,083	-0,032	0,127	-0,250	-0,232	0,306	0,071	-0,131	-0,138	*1*	0,103	0,056
**DYX1C1**	0,170	-0,003	0,141	0,312	0,476	-0,128	0,065	0,541	-0,129	0,280	0,303	0,295	0,103	*1*	0,210
**GRIN2B**	0,028	0,255	-0,066	0,440	0,325	-0,181	0,185	-0,087	0,088	0,194	0,321	0,322	0,056	0,210	*1*

Supplementary table S3. White matter density correlates with variation in dyslexia genes

Variant	Gene	Cluster p-value	Peak coordinates			Genotypes*	Brain region
rs3743204	*DYX1C1*	1.28×10^{-10}	−16	−54	18	GG>GT/TT	bilateral tempropariental
rs793842	*DCDC2*	8.19×10^{-5}	−28	−70	33	CC>CT>TT	left temporoparietal
rs6935076	*KIAA0319*	3.33×10^{-10}	−34	−58	31	CC>CT>TT	bilateral tempropariental
rs917235	MRPL19	$1.27{\times}10^{-3}$	-13	-8	8	AA>GG	bilateral tempropariental
rs2561622	*CTNND2*	1.28×10^{-5}	-47	25	22	GG>AG>AA	left frontal
rs7519451	*CEP63*	7.60×10-3	28	-55	29	AA/AC>CC	right temporoparietal
rs1064395	*NCAN*	$1.56{\times}10^{-6}$	44	-36	13	TC>CC	right temporoparietal and frontal
		$4.48{\times}10^{-10}$	-39	20	20	TC>CC	left temporoparietal, frontal and occipital

* Genotype groups ordered according to the highest white matter density in each specific cluster

Supplementary table S4

Associations to white mattter volume p < 0.01

Brain region	Coordinates in SPM MNI space			Cluster size	peak	cluster	
	x	y	z		T	p(FWE-corr)	p(FDR-corr)
right temporoparietal and frontal	44	-36	13	7878	6,51	1.56×10^{-6}	1.04×10^{-6}
left temporoparietal, frontal and occip	-39	20	20	14355	5,13	4.48×10^{-10}	5.96×10^{-10}

Associations to white mattter volume p < 0.001

Brain region	Coordinates in SPM MNI space			Cluster size	peak	cluster	
	x	y	z		T	p(FWE-corr)	p(FDR-corr)
right temporoparietal and frontal	44	-36	13	3549	6,51	8.13×10^{-8}	2.88×10^{-7}
left temporoparietal, frontal and occip	-39	20	20	3312	5,13	1.82×10^{-7}	3.23×10^{-7}

Associations to white mattter volume p < 0.05 (FDR)

Brain region	Coordinates in SPM MNI space			Cluster size	peak	cluster	
	x	y	z		T	p(FWE-corr)	p(FDR-corr)
right temporoparietal and frontal	44	-36	13	361	6,51	9.6×10−6	1.8×10^{-3}
left temporoparietal, frontal and occip	-39	20	20	75	5,13	2.5×10^{-3}	0,04

Supplementary table 5. Linear regression analysis of rs1064395 (*NCAN*) to grey matter volumes in newfant brain.

**A**	**Rs1064395***	
**Grey matter volume**	β	**P**
Total	.347	.175
Brain regions:		
Frontal	.424	.115
Cingulate	.516	**.049**
Limbic	.365	.183
Occipital	.226	.393
Parietal	.414	.077
Temporal	.196	.406

* MAF=.12; for genotypes Maj/Maj n=20, Maj/Min n=6

**B**	**Rs1064395**	
**Parietal lobe**	β	**P**
Postcentral gyrus	.515	**.045**
Superior parietal gyrus	-.126	.614
Inferior parietal lobule	.501	**.042**
Supramarginal gyrus	.657	**.005**
Angular gyrus	-.085	.718
Precuneus	.463	.066
Paracentral lobule	.148	.571

**C**	**Rs1064395**	
**Cingulate**	β	**P**
Anterior cingulate gyrus	.419	.121
Middle cingulate gyrus	.547	**.031**
Posterior cingulate gyrus	.180	.517

Supplementary table S6

A. Associations to infant grey matter volume $p < 0.001$, FDR corrected

**Brain region (iBEAT AAL label)**	**Coordinates in SPM MNI space**			**Cluster size**	**peak**	**cluster**
	x	y	z		T	p(FDR-corr)
Left inferior parietal lobule (61)	-30	-6	14	484	4.72	0.001
Right precentral gyrus (2)	28	10	12	411	4.40	0.001
Right middle frontal gyrus (8)	30	26	10	192	3.99	0.011

B. Associations to infant grey matter volume $p < 0.01$, FDR corrected

**Brain region (iBEAT AAL label)**	**Coordinates in SPM MNI space**			**Cluster size**	**peak**	**cluster**
	x	y	z		T	p(FDR-corr)
Left inferior parietal lobule (61)	-30	-6	14	1681	4.72	0.000
Right precentral gyrus (2)	28	10	12	3387	4.40	0.000
Right middle frontal gyrus (8)	30	26	10		3.99	
Right postcentral gyrus (58)	34	4	16		3.17	
Right middle cingulate gyrus (34)	4	14	6	1806	3.46	0.000
Left middle cingulate gyrus (33)	-4	20	8		2.97	

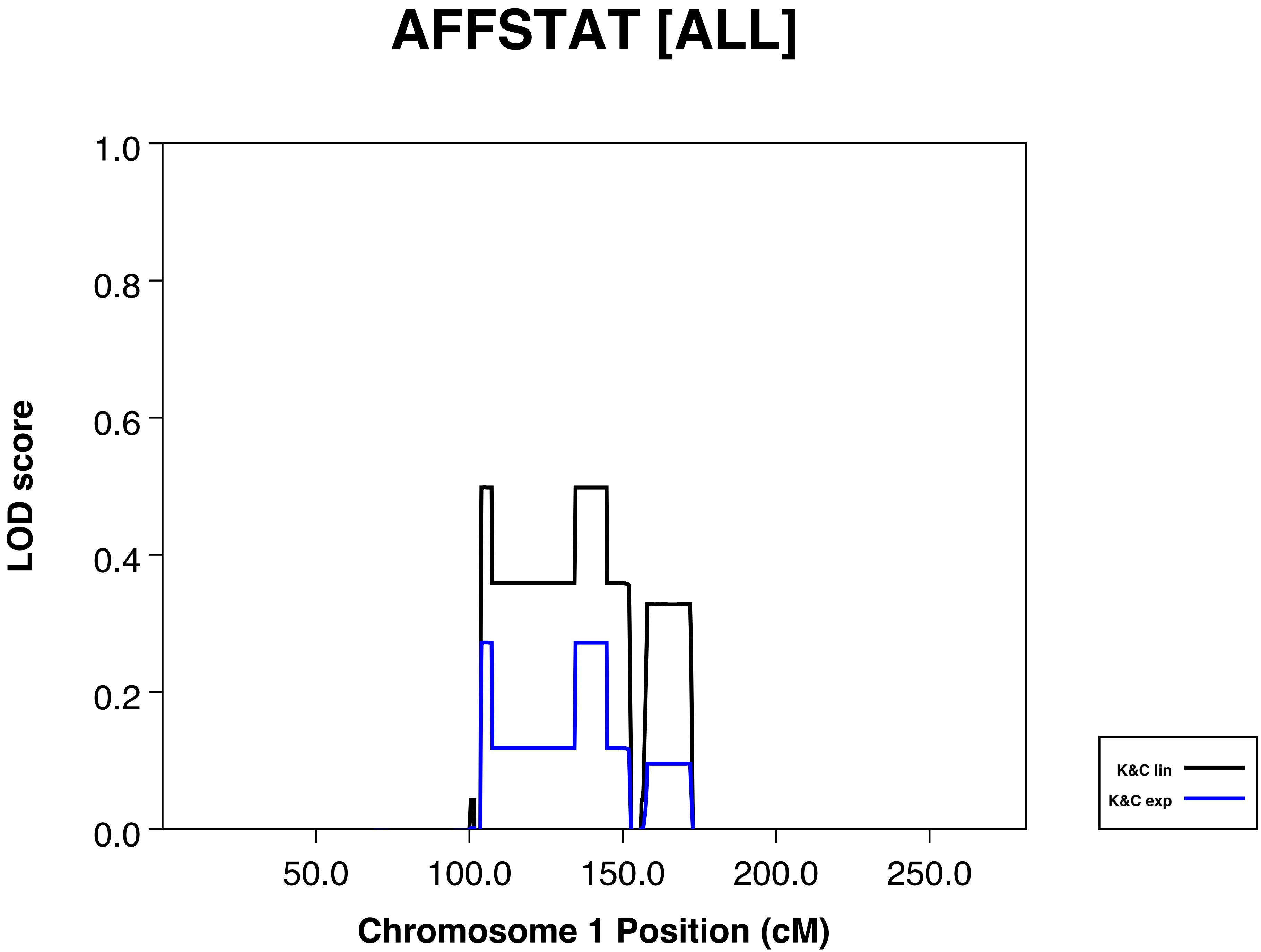
AFFSTAT [ALL]
1.0
0.8
0.6
0.4
0.2
0.0
LOD score
50.0
100.0
150.0
200.0
250.0
Chromosome 1 Position (cM)
K&C lin
K&C exp

AFFSTAT [ALL]

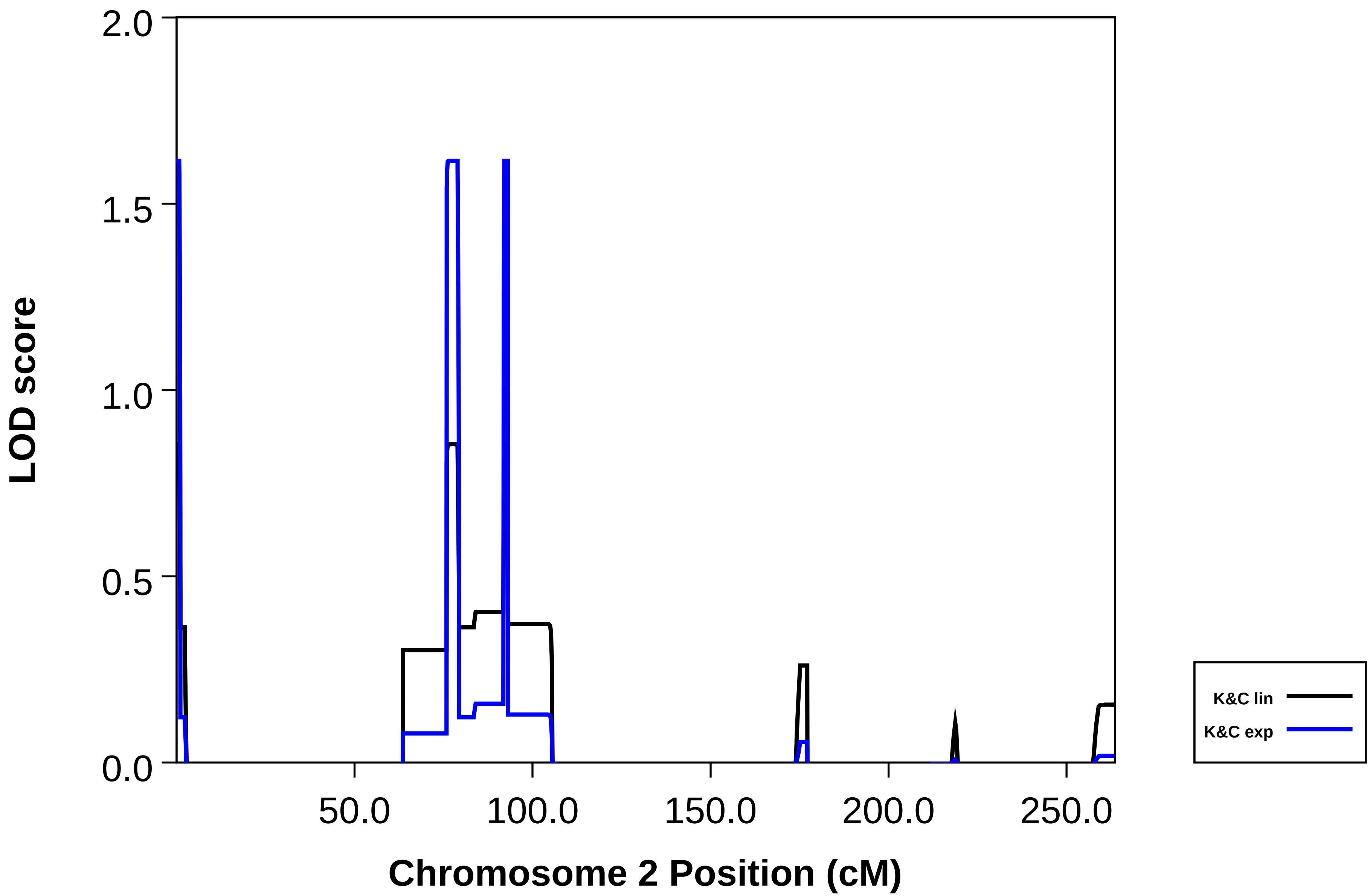

2.0
1.5
1.0
0.5
0.0
LOD score
50.0
100.0
150.0
200.0
250.0
Chromosome 2 Position (cM)
K&C lin
K&C exp

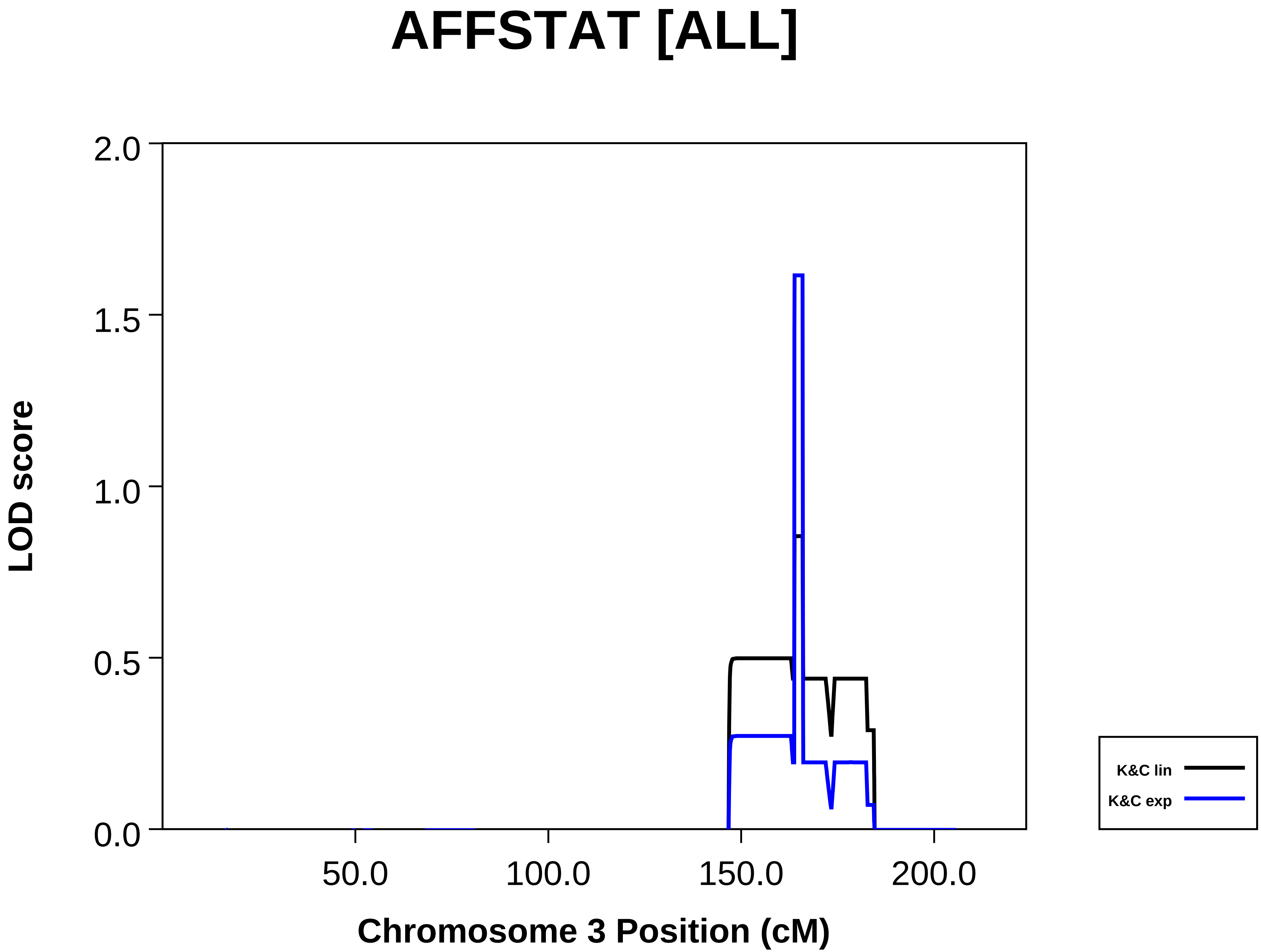
AFFSTAT [ALL]
2.0
1.5
1.0
0.5
0.0
LOD score
50.0
100.0
150.0
200.0
Chromosome 3 Position (cM)
K&C lin
K&C exp

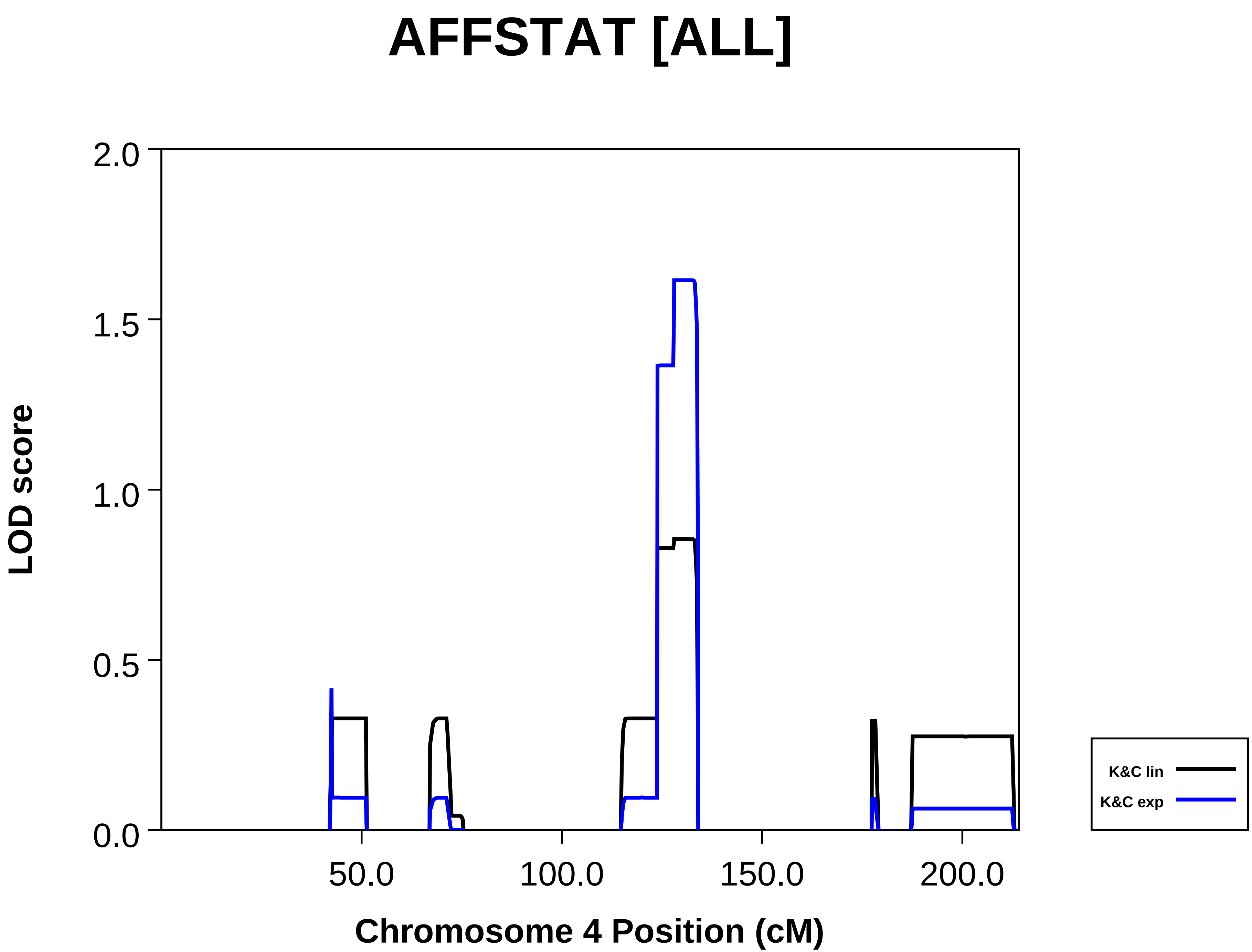
AFFSTAT [ALL]
LOD score
2.0
1.5
1.0
0.5
0.0
50.0
100.0
150.0
200.0
Chromosome 4 Position (cM)
K&C lin
K&C exp

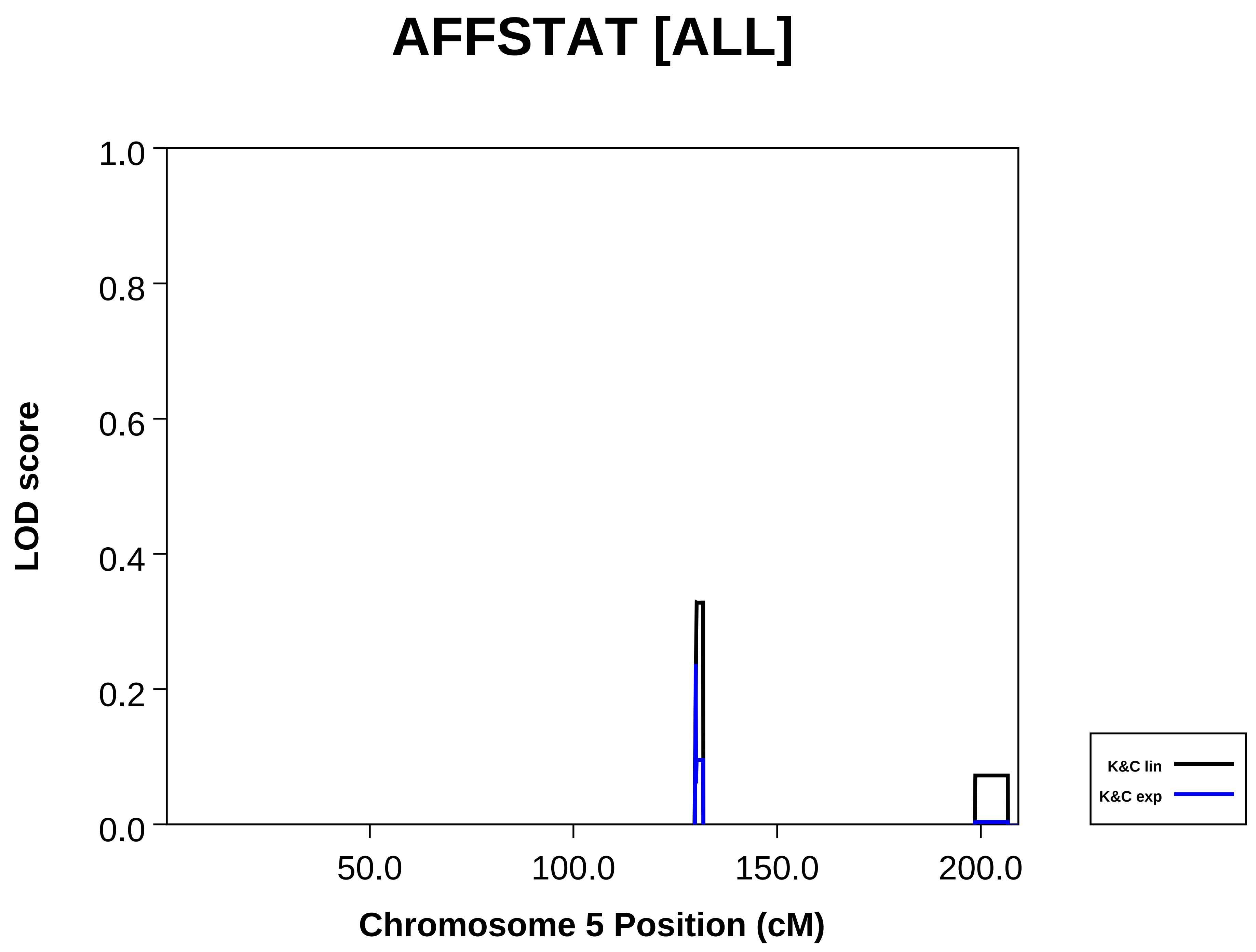

AFFSTAT [ALL]
LOD score
1.0
0.8
0.6
0.4
0.2
0.0
50.0
100.0
150.0
200.0
Chromosome 5 Position (cM)
K&C lin
K&C exp

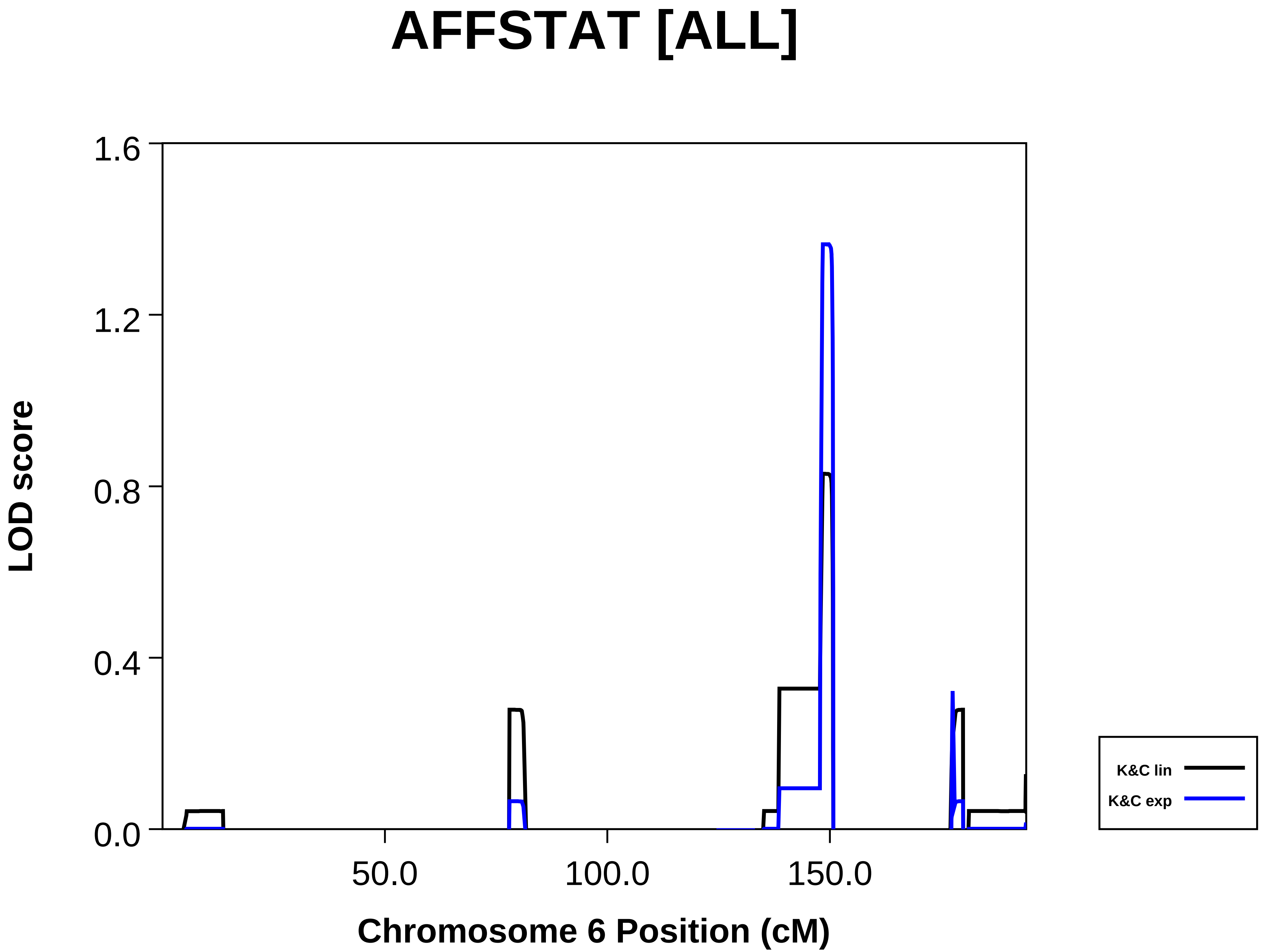
AFFSTAT [ALL]
1.6
1.2
0.8
0.4
0.0
LOD score
50.0
100.0
150.0
Chromosome 6 Position (cM)
K&C lin
K&C exp

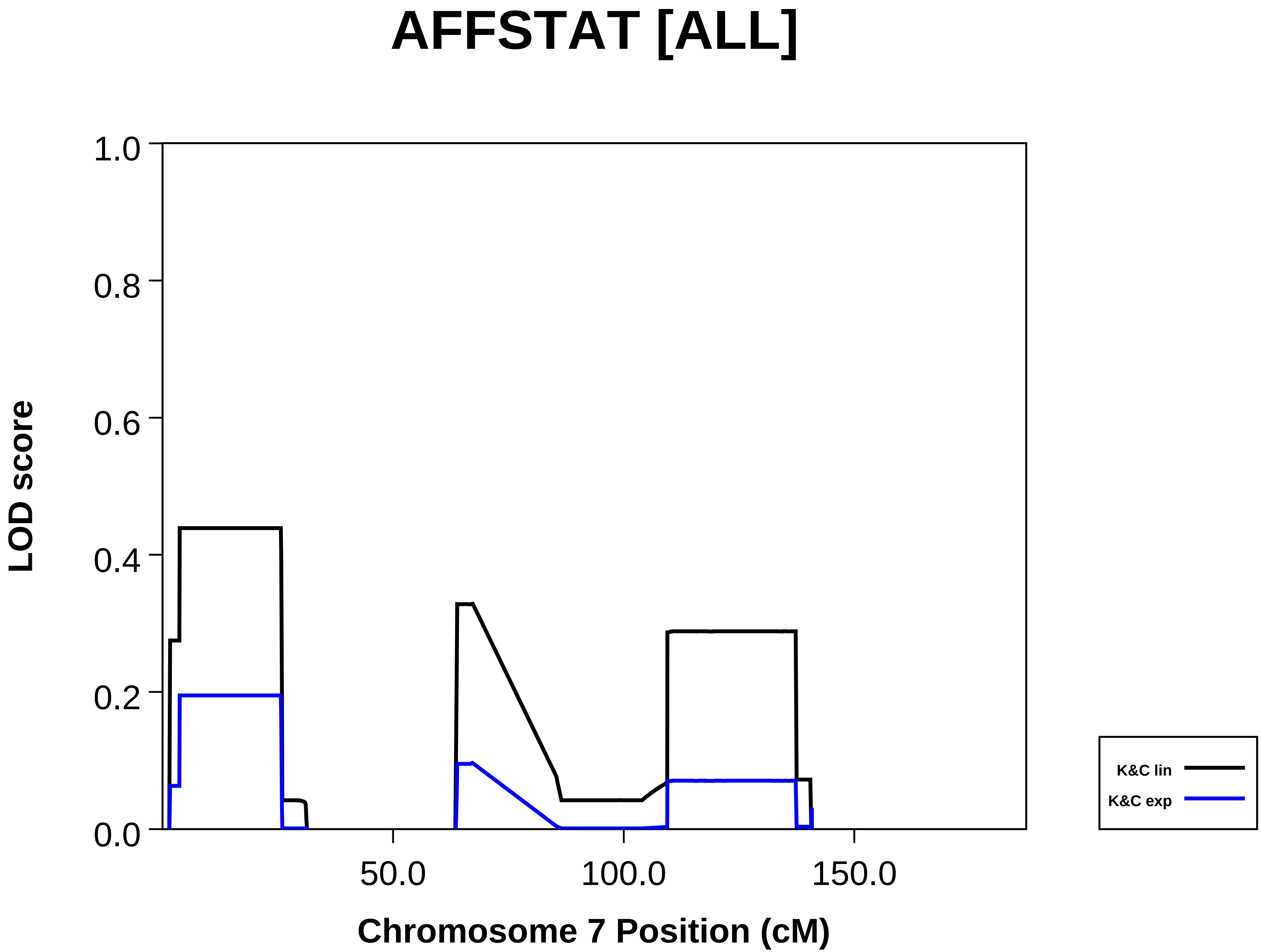

AFFSTAT [ALL]
LOD score
1.0
0.8
0.6
0.4
0.2
0.0
50.0
100.0
150.0
Chromosome 7 Position (cM)
K&C lin
K&C exp

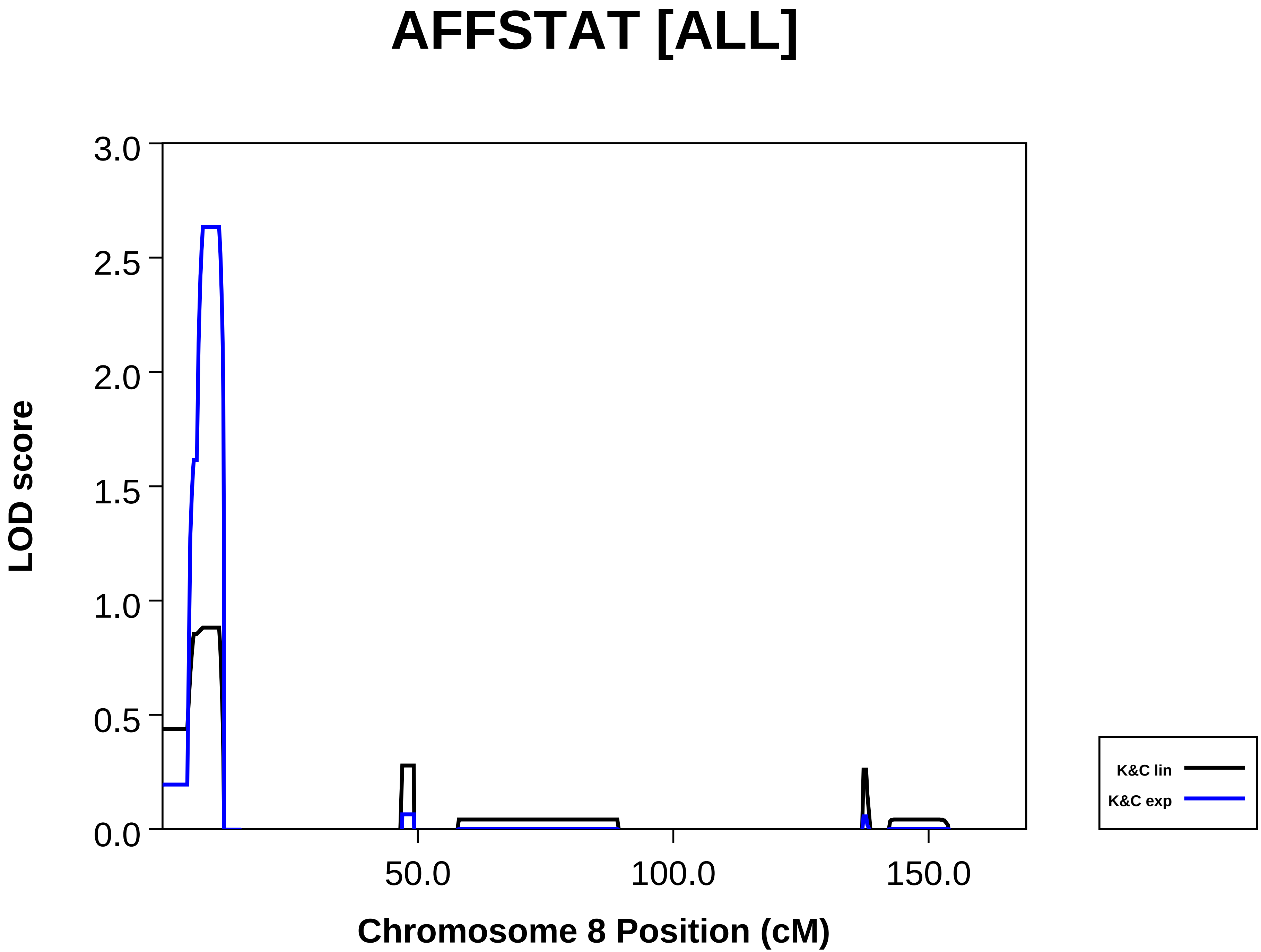
AFFSTAT [ALL]
LOD score
3.0
2.5
2.0
1.5
1.0
0.5
0.0
50.0
100.0
150.0
Chromosome 8 Position (cM)
K&C lin
K&C exp

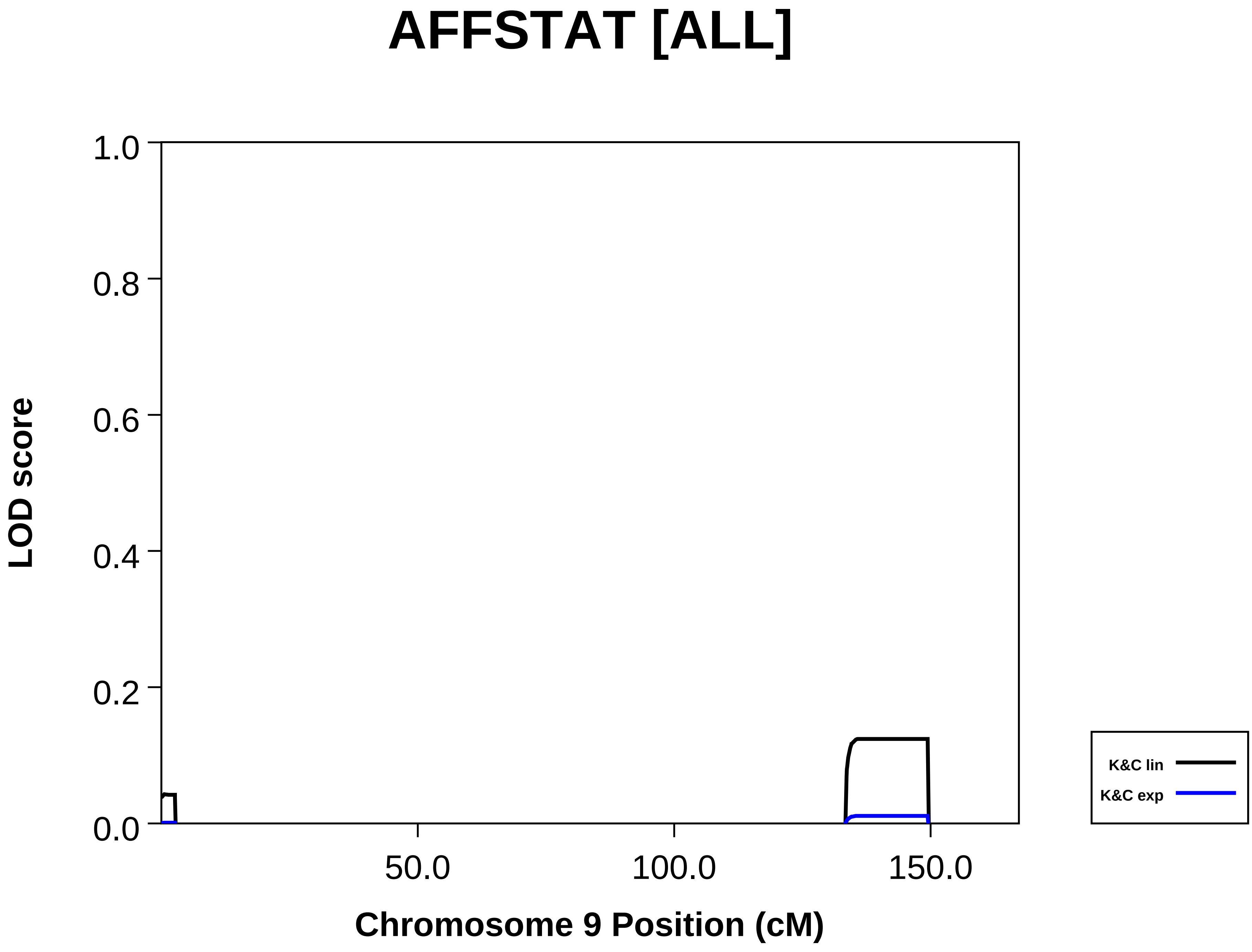
AFFSTAT [ALL]
1.0
0.8
0.6
0.4
0.2
0.0
LOD score
50.0
100.0
150.0
Chromosome 9 Position (cM)
K&C lin
K&C exp

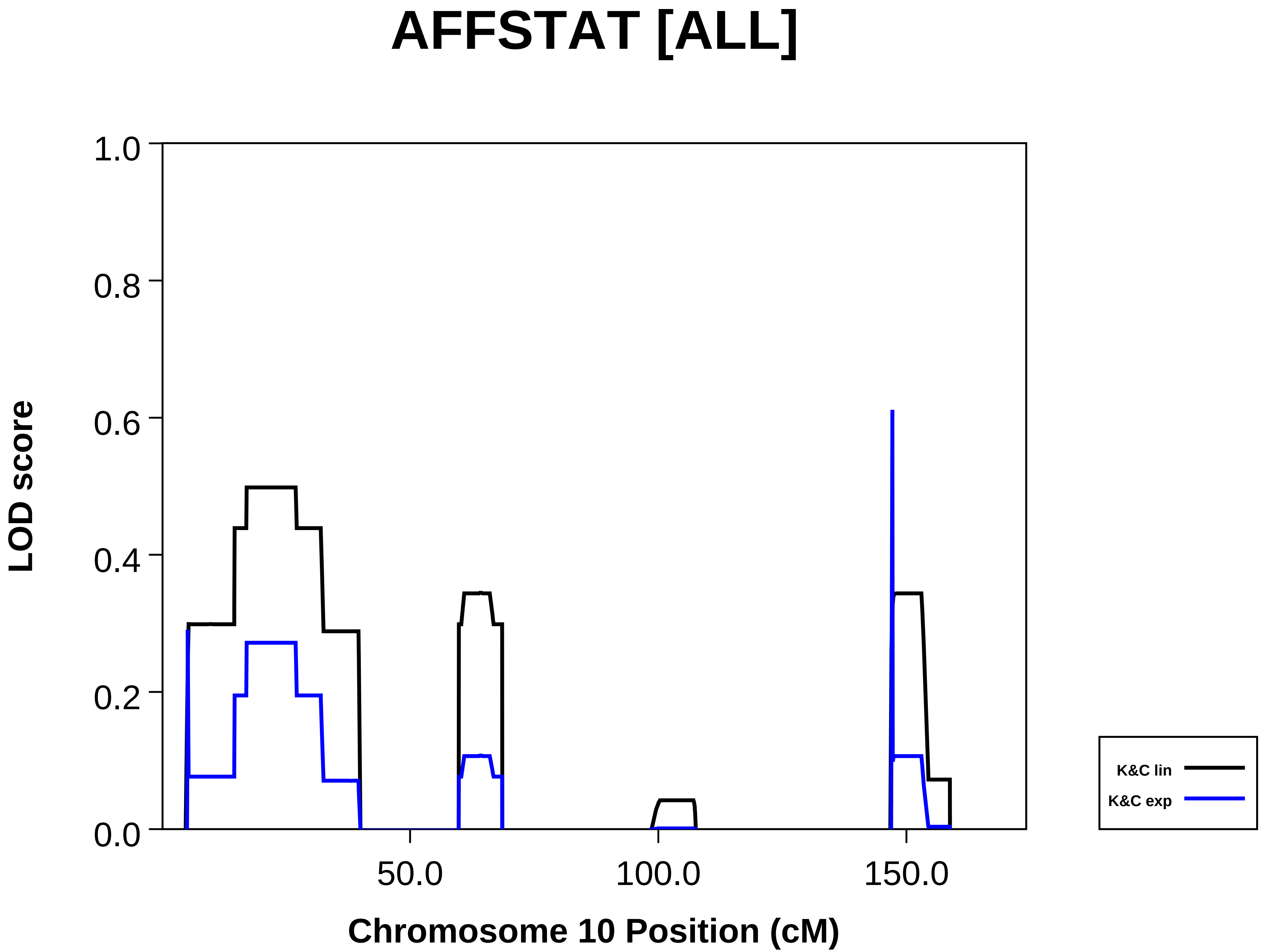
AFFSTAT [ALL]
LOD score
1.0
0.8
0.6
0.4
0.2
0.0
50.0
100.0
150.0
Chromosome 10 Position (cM)
K&C lin
K&C exp

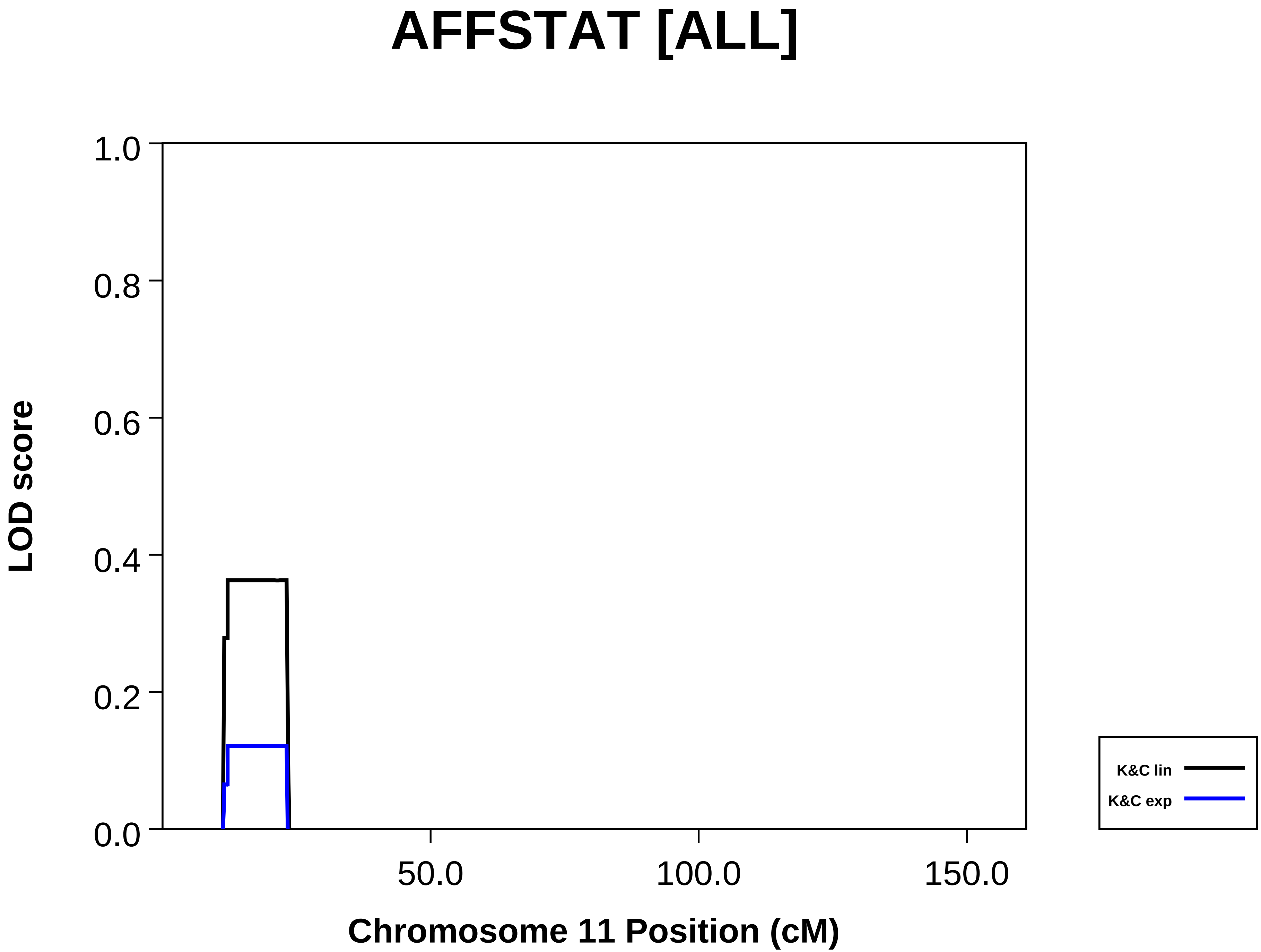
AFFSTAT [ALL]
1.0
0.8
0.6
0.4
0.2
0.0
LOD score
50.0
100.0
150.0
Chromosome 11 Position (cM)
K&C lin
K&C exp

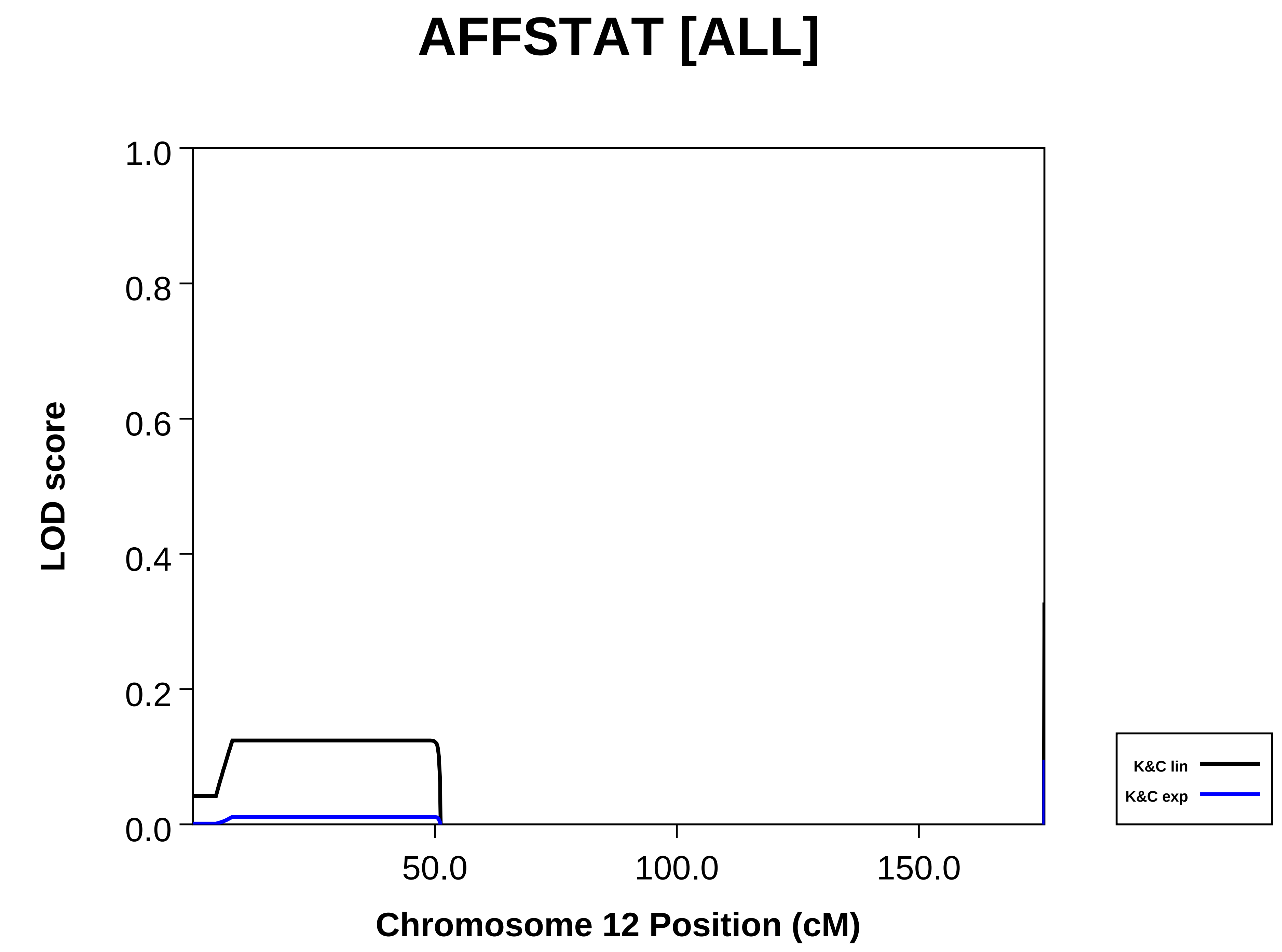

AFFSTAT [ALL]
LOD score
1.0
0.8
0.6
0.4
0.2
0.0
50.0
100.0
150.0
Chromosome 12 Position (cM)
K&C lin
K&C exp

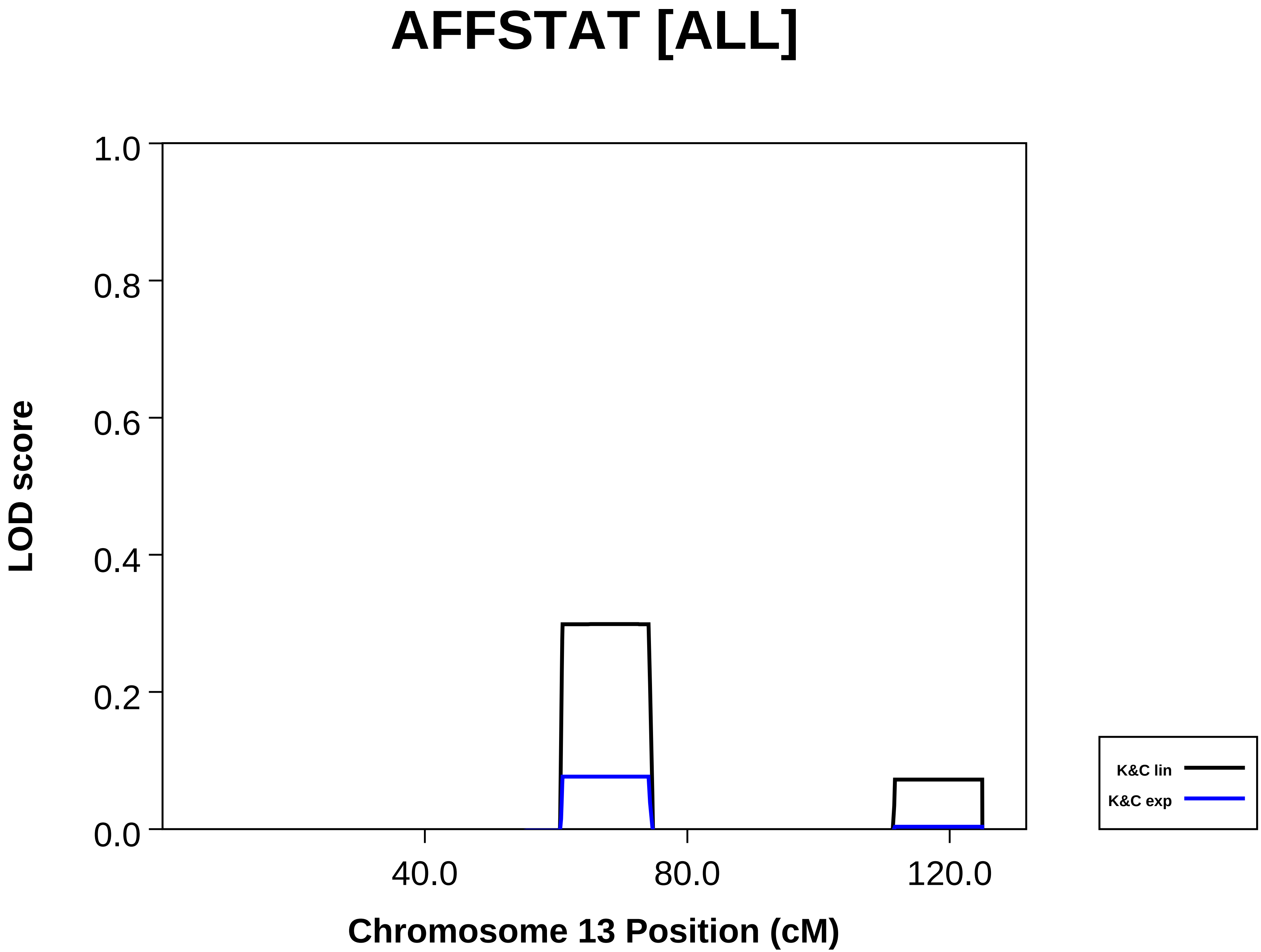
AFFSTAT [ALL]
1.0
0.8
0.6
0.4
0.2
0.0
LOD score
40.0
80.0
120.0
Chromosome 13 Position (cM)
K&C lin
K&C exp

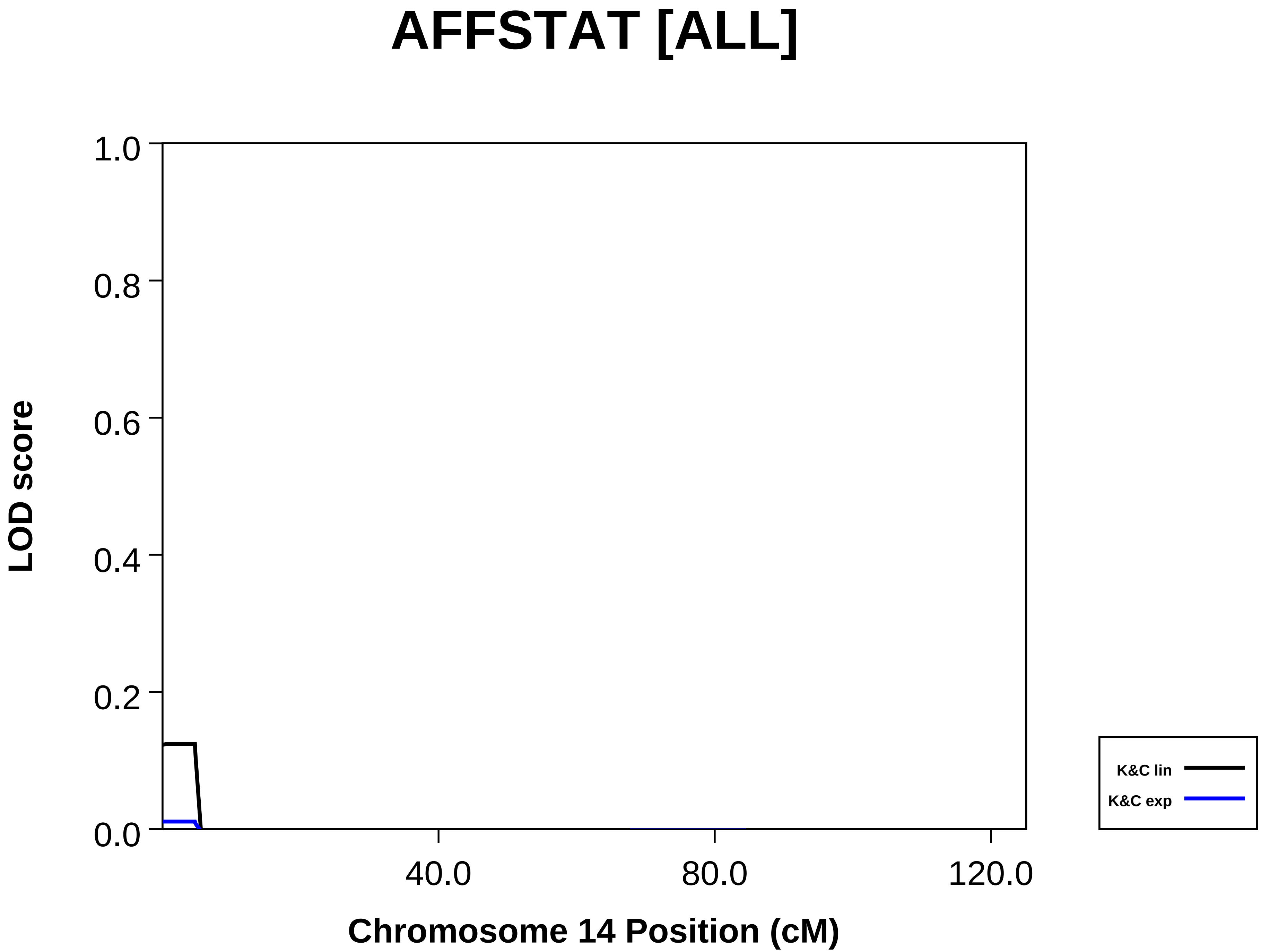

AFFSTAT [ALL]
LOD score
1.0
0.8
0.6
0.4
0.2
0.0
40.0
80.0
120.0
Chromosome 14 Position (cM)
K&C lin
K&C exp

AFFSTAT [ALL]

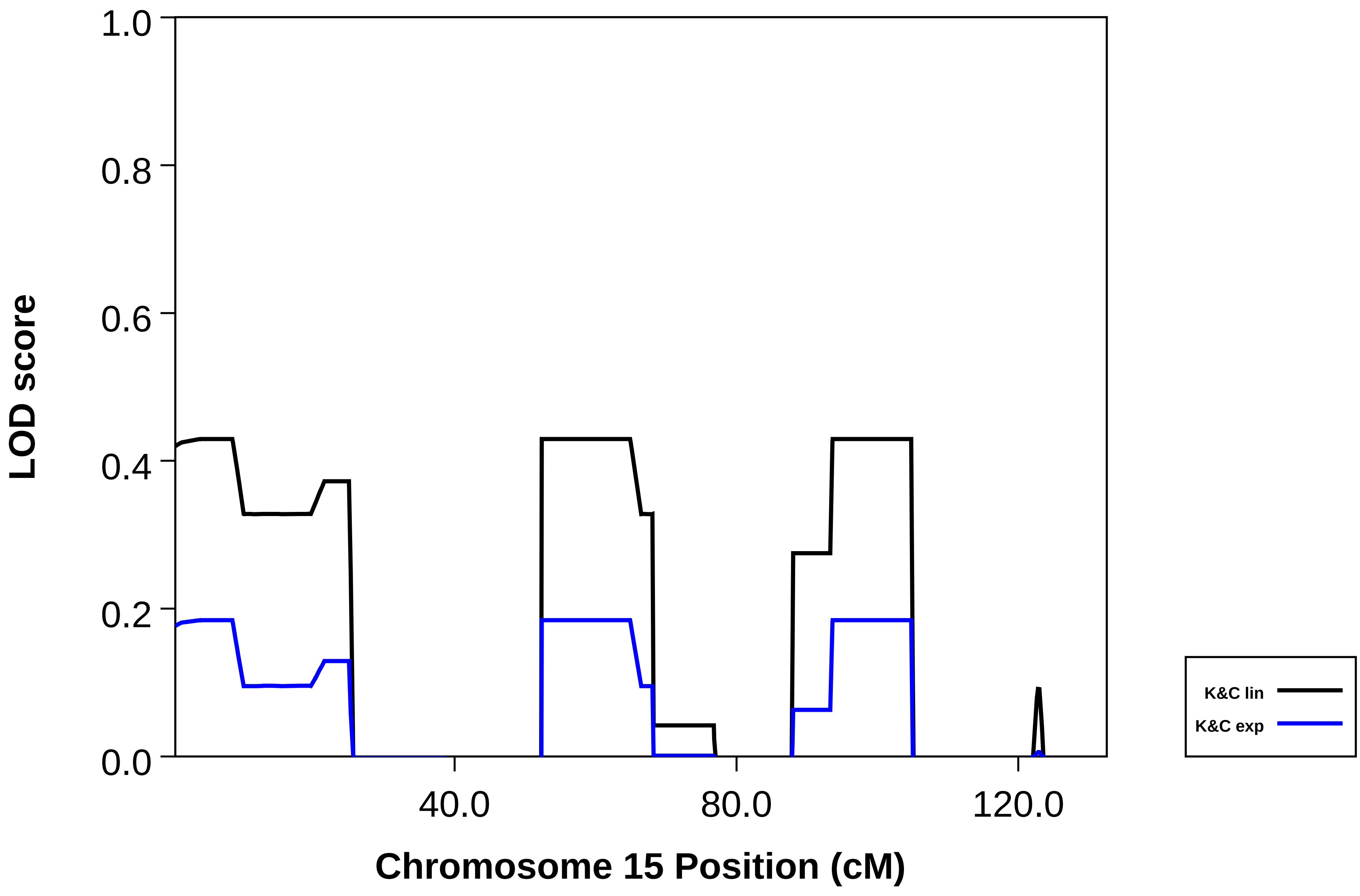

LOD score
1.0
0.8
0.6
0.4
0.2
0.0
40.0
80.0
120.0
Chromosome 15 Position (cM)
K&C lin
K&C exp

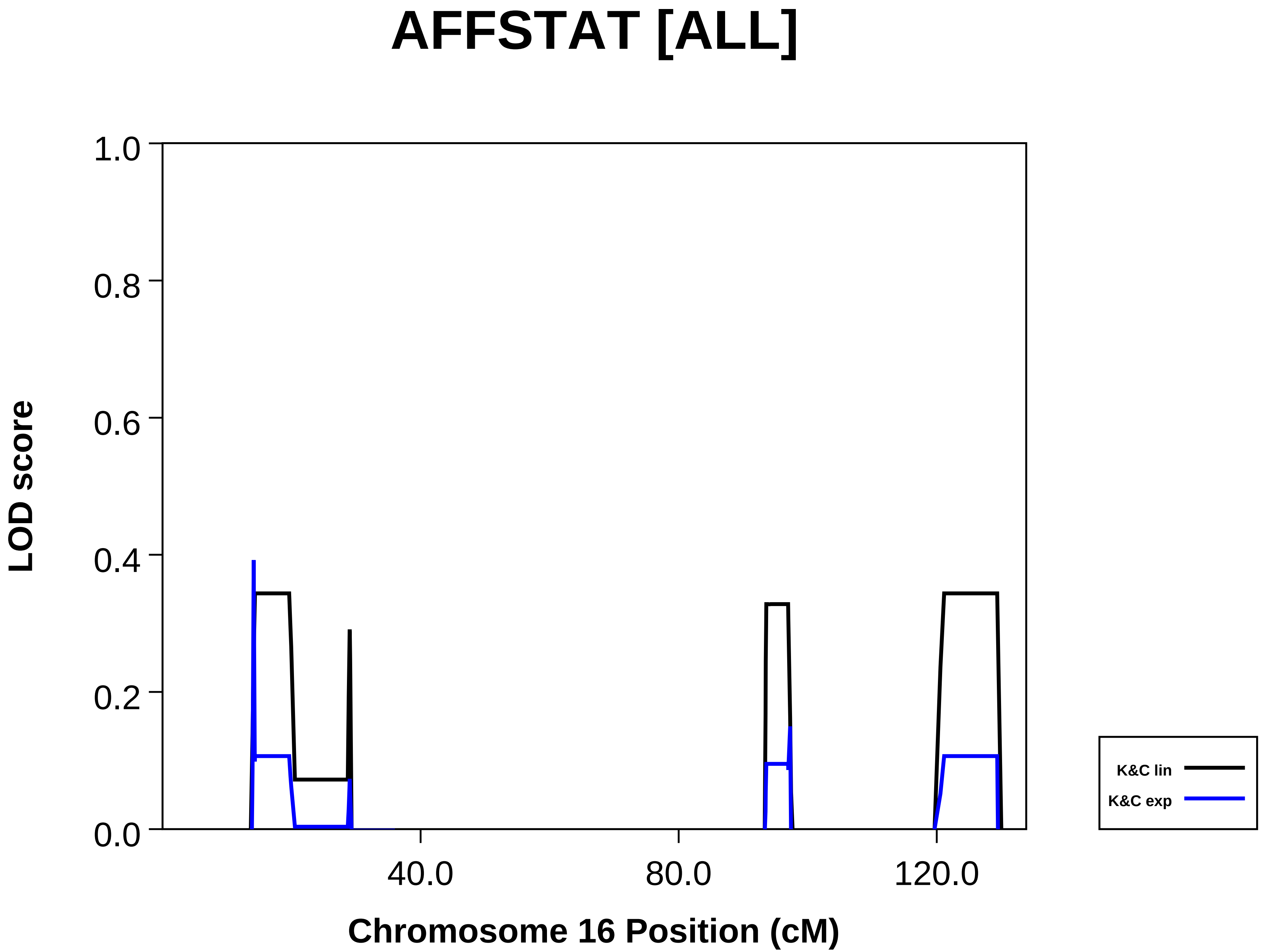

AFFSTAT [ALL]
LOD score
1.0
0.8
0.6
0.4
0.2
0.0
40.0
80.0
120.0
Chromosome 16 Position (cM)
K&C lin
K&C exp

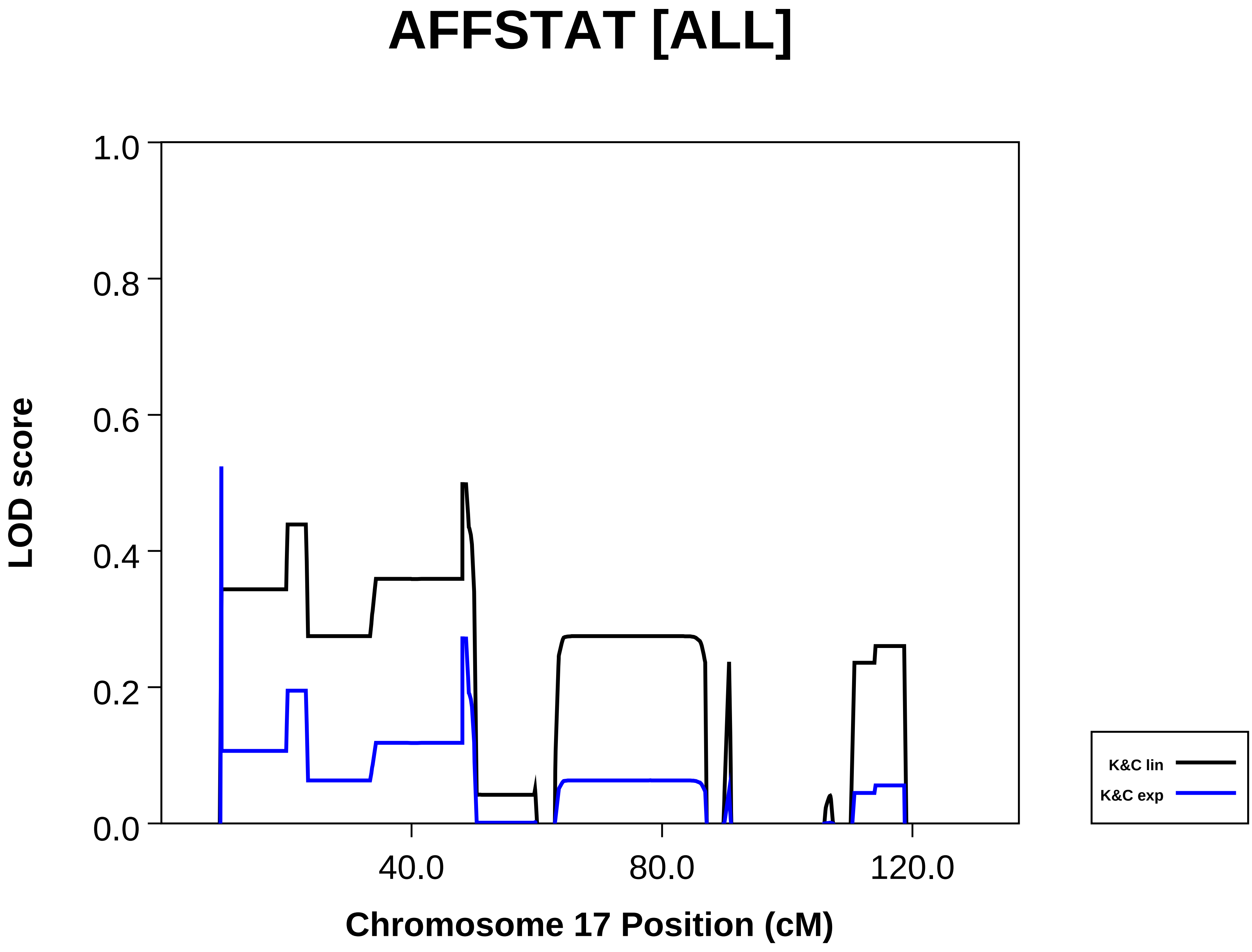
AFFSTAT [ALL]
1.0
0.8
0.6
0.4
0.2
0.0
LOD score
40.0
80.0
120.0
Chromosome 17 Position (cM)
K&C lin
K&C exp

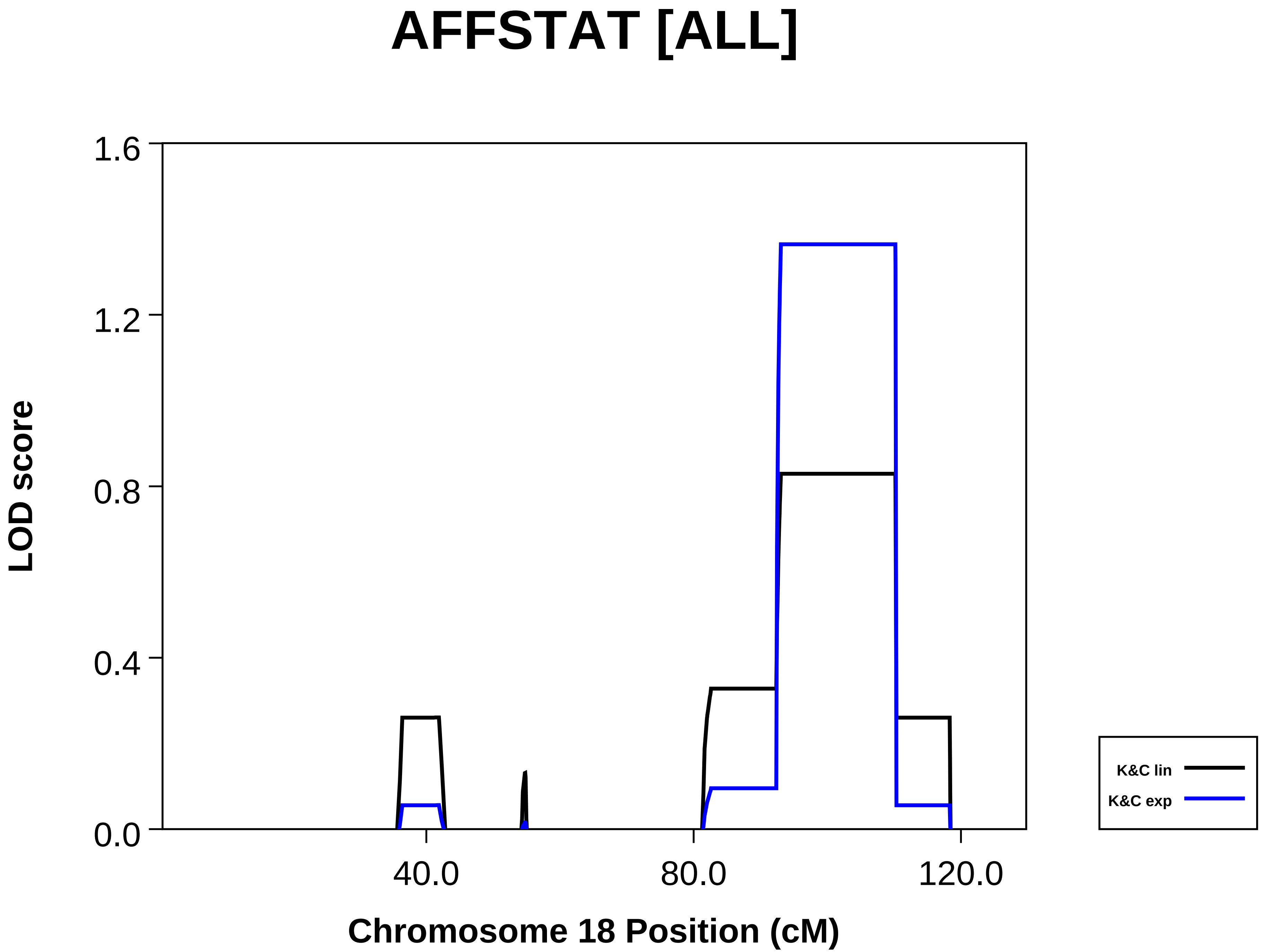

AFFSTAT [ALL]
LOD score
1.6
1.2
0.8
0.4
0.0
40.0
80.0
120.0
Chromosome 18 Position (cM)
K&C lin
K&C exp

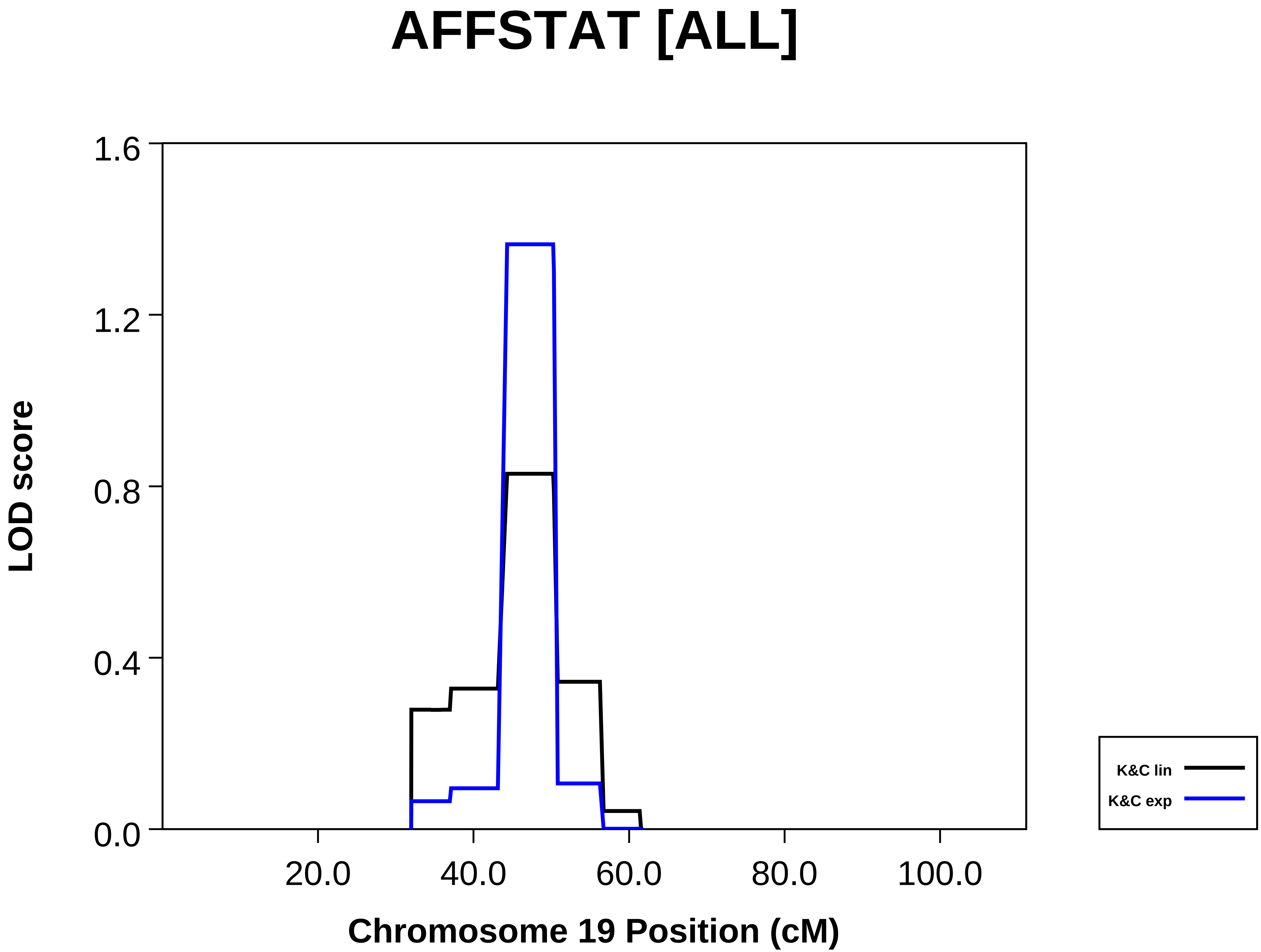
AFFSTAT [ALL]
1.6
1.2
0.8
0.4
0.0
LOD score
20.0
40.0
60.0
80.0
100.0
Chromosome 19 Position (cM)
K&C lin
K&C exp

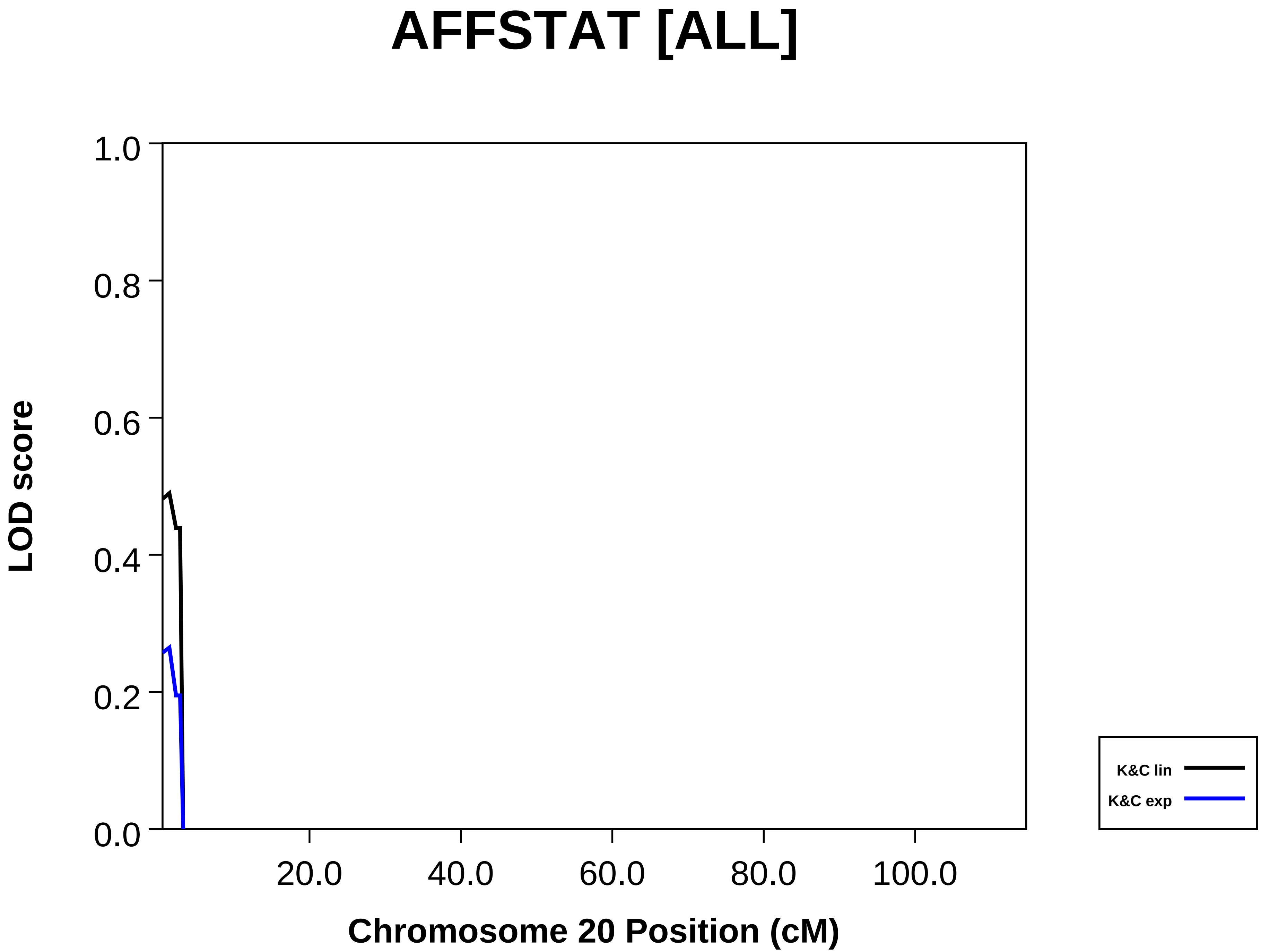
AFFSTAT [ALL]
1.0
0.8
0.6
0.4
0.2
0.0
LOD score
20.0
40.0
60.0
80.0
100.0
Chromosome 20 Position (cM)
K&C lin
K&C exp

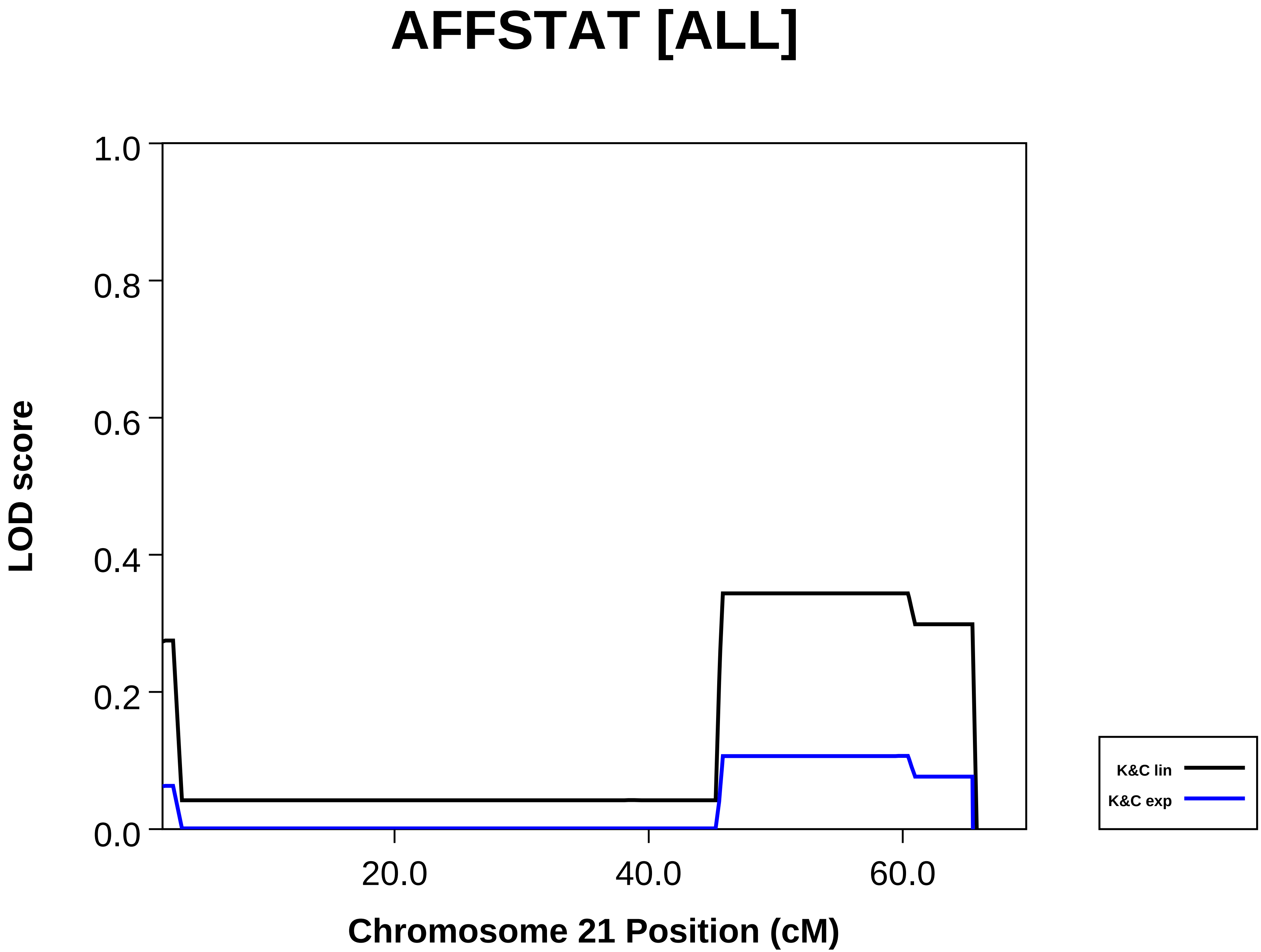

AFFSTAT [ALL]
LOD score
1.0
0.8
0.6
0.4
0.2
0.0
20.0
40.0
60.0
Chromosome 21 Position (cM)
K&C lin
K&C exp

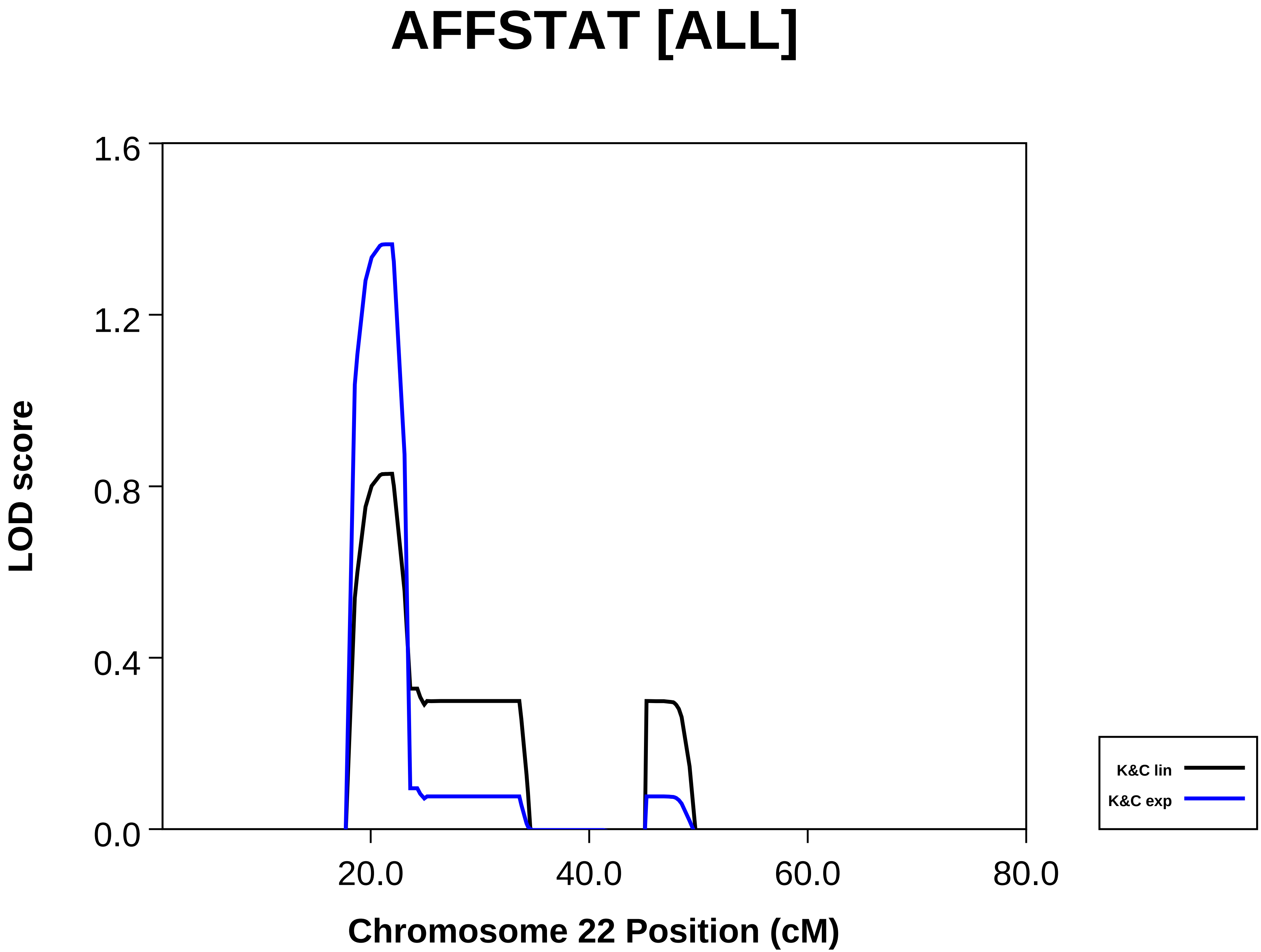
AFFSTAT [ALL]
LOD score
1.6
1.2
0.8
0.4
0.0
20.0
40.0
60.0
80.0
Chromosome 22 Position (cM)
K&C lin
K&C exp
