# Supplementary material for: Identification of NCAN as a candidate gene for developmental dyslexia
Source: Sci Rep. 2017 Aug 24;7:9294. doi: 10.1038/s41598-017-10175-7 (PMC5570950; doi:10.1038/s41598-017-10175-7)
Supplement: Supplementary file 2 — Supplementary dataset 1 [file 41598_2017_10175_MOESM2_ESM.doc]

**SUPPLEMENTARY DATASET 1 - NPL LINKAGE**

MERLIN 1.1.2 - (c) 2000-2007 Goncalo Abecasis

References for this version of Merlin:

Abecasis et al (2002) Nat Gen 30:97-101 [original citation]

Fingerlin et al (2004) AJHG 74:432-43 [case selection for association studies]

Abecasis and Wigginton (2005) AJHG 77:754-67 [ld modeling, parametric analyses]

Fingerlin et al (2006) Gen Epidemiol 30:384-96 [sex-specific maps]

Chen and Abecasis (2007) AJHG 81:913-26 [qtl association analysis, qtl simulation]

The following parameters are in effect:

Data File : merlin_dat.977280 (-dname)

Pedigree File : merlin_ped.977280 (-pname)

Missing Value Code : -99.999 (-xname)

Map File : merlin_map.977280 (-mname)

Allele Frequencies : merlin_frq.977280 (-f[a|e|f|m|file])

Random Seed : 977280 (-r9999)

Data Analysis Options

General : --error, --information, --likelihood, --model [param.tbl]

IBD States : --ibd, --kinship, --matrices, --extended, --select

NPL Linkage : --npl [ON], --pairs, --qtl, --deviates, --exp [ON]

VC Linkage : --vc, --useCovariates, --ascertainment, --unlinked [0.00]

Association : --infer, --assoc, --fastAssoc, --filter, --custom [cov.tbl]

Haplotyping : --best, --sample, --all, --founders, --horizontal

Recombination : --zero, --one, --two, --three, --singlepoint

Positions : --steps, --maxStep, --minStep, --grid, --start, --stop

LD Clusters : --clusters [], --distance, --rsq, --cfreq

Limits : --bits [24], --megabytes [4096], --minutes

Performance : --trim [ON], --noCoupleBits, --swap [ON], --smallSwap

Output : --quiet [ON], --markerNames [ON], --frequencies,

--perFamily, --pdf [ON], --tabulate [ON], --prefix [merlin]

Simulation : --simulate, --reruns, --save, --trait []

Estimating allele frequencies... [using all genotypes]

.

Done estimating frequencies for 2 markers

Analysing Chromosome 1

Phenotype: AFFSTAT [ALL] (1 family)

===============================================================================

Pos Zmean pvalue linDelta LOD pvalue expDelta LOD pvalue

min -0.76 0.8 -0.198 -0.06 0.7 -9.999 -0.55 0.9

max 5.05 0.00000 1.309 0.88 0.02 9.999 2.62 0.0003

RS11240777 -0.65 0.7 -0.198 -0.05 0.7 -7.521 -0.39 0.9

RS4970383 -0.65 0.7 -0.198 -0.05 0.7 -7.522 -0.39 0.9

RS2341354 -0.65 0.7 -0.198 -0.05 0.7 -7.522 -0.39 0.9

RS3128126 -0.65 0.7 -0.198 -0.05 0.7 -7.522 -0.39 0.9

RS9442372 -0.65 0.7 -0.198 -0.05 0.7 -7.522 -0.39 0.9

RS10907182 -0.65 0.7 -0.198 -0.05 0.7 -7.523 -0.39 0.9

RS28635343 -0.65 0.7 -0.198 -0.05 0.7 -7.522 -0.39 0.9

RS7407 -0.65 0.7 -0.198 -0.05 0.7 -7.523 -0.39 0.9

RS28508199 -0.65 0.7 -0.198 -0.05 0.7 -7.522 -0.39 0.9

RS3795283 -0.65 0.7 -0.198 -0.05 0.7 -7.522 -0.39 0.9

RS6605081 -0.65 0.7 -0.198 -0.05 0.7 -7.522 -0.39 0.9

RS7513222 -0.65 0.7 -0.198 -0.05 0.7 -7.522 -0.39 0.9

RS12084736 -0.65 0.7 -0.198 -0.05 0.7 -7.522 -0.39 0.9

RS903908 -0.65 0.7 -0.198 -0.05 0.7 -7.522 -0.39 0.9

RS10910047 -0.65 0.7 -0.198 -0.05 0.7 -7.522 -0.39 0.9

RS884940 -0.65 0.7 -0.198 -0.05 0.7 -7.522 -0.39 0.9

RS10910053 -0.65 0.7 -0.198 -0.05 0.7 -7.522 -0.39 0.9

RS3100865 -0.65 0.7 -0.198 -0.05 0.7 -7.522 -0.39 0.9

RS7340016 -0.65 0.7 -0.198 -0.05 0.7 -7.522 -0.39 0.9

RS10797368 -0.65 0.7 -0.198 -0.05 0.7 -7.522 -0.39 0.9

RS16823228 -0.65 0.7 -0.198 -0.05 0.7 -7.522 -0.39 0.9

RS12044030 -0.65 0.7 -0.198 -0.05 0.7 -7.522 -0.39 0.9

RS926244 -0.65 0.7 -0.198 -0.05 0.7 -7.522 -0.39 0.9

RS7543737 -0.65 0.7 -0.198 -0.05 0.7 -7.522 -0.39 0.9

RS1890336 -0.65 0.7 -0.198 -0.05 0.7 -7.522 -0.39 0.9

RS2817178 -0.65 0.7 -0.198 -0.05 0.7 -7.522 -0.39 0.9

RS2235176 -0.65 0.7 -0.198 -0.05 0.7 -7.522 -0.39 0.9

RS2742673 -0.65 0.7 -0.198 -0.05 0.7 -7.522 -0.39 0.9

RS2651935 -0.65 0.7 -0.198 -0.05 0.7 -7.522 -0.39 0.9

RS3002686 -0.65 0.7 -0.198 -0.05 0.7 -7.522 -0.39 0.9

RS2455144 -0.65 0.7 -0.198 -0.05 0.7 -7.522 -0.39 0.9

RS2483289 -0.65 0.7 -0.198 -0.05 0.7 -7.522 -0.39 0.9

RS7524171 -0.65 0.7 -0.198 -0.05 0.7 -7.522 -0.39 0.9

RS2483225 -0.65 0.7 -0.198 -0.05 0.7 -7.522 -0.39 0.9

RS867814 -0.65 0.7 -0.198 -0.05 0.7 -7.522 -0.39 0.9

RS12119711 -0.65 0.7 -0.198 -0.05 0.7 -7.522 -0.39 0.9

RS2821060 -0.65 0.7 -0.198 -0.05 0.7 -7.522 -0.39 0.9

RS4364818 -0.65 0.7 -0.198 -0.05 0.7 -7.522 -0.39 0.9

RS2224718 -0.65 0.7 -0.198 -0.05 0.7 -7.522 -0.39 0.9

RS6424089 -0.65 0.7 -0.198 -0.05 0.7 -7.522 -0.39 0.9

RS4648553 -0.65 0.7 -0.198 -0.05 0.7 -7.522 -0.39 0.9

RS6663840 -0.65 0.7 -0.198 -0.05 0.7 -7.522 -0.39 0.9

RS2275831 -0.65 0.7 -0.198 -0.05 0.7 -7.522 -0.39 0.9

RS6668103 -0.65 0.7 -0.198 -0.05 0.7 -7.522 -0.39 0.9

RS4446914 -0.65 0.7 -0.198 -0.05 0.7 -7.522 -0.39 0.9

RS12031275 -0.65 0.7 -0.198 -0.05 0.7 -7.522 -0.39 0.9

RS6675979 -0.65 0.7 -0.198 -0.05 0.7 -7.522 -0.39 0.9

RS10915659 -0.65 0.7 -0.198 -0.05 0.7 -7.522 -0.39 0.9

RS7548756 -0.65 0.7 -0.198 -0.05 0.7 -7.522 -0.39 0.9

RS6426389 -0.65 0.7 -0.198 -0.05 0.7 -7.522 -0.39 0.9

RS4614226 -0.65 0.7 -0.198 -0.05 0.7 -7.522 -0.39 0.9

RS16840873 -0.65 0.7 -0.198 -0.05 0.7 -7.522 -0.39 0.9

RS6687268 -0.65 0.7 -0.198 -0.05 0.7 -7.522 -0.39 0.9

RS2101576 -0.65 0.7 -0.198 -0.05 0.7 -7.522 -0.39 0.9

RS351619 -0.65 0.7 -0.198 -0.05 0.7 -7.522 -0.39 0.9

RS10799228 -0.65 0.7 -0.198 -0.05 0.7 -7.522 -0.39 0.9

RS349393 -0.65 0.7 -0.198 -0.05 0.7 -7.522 -0.39 0.9

RS6678792 -0.65 0.7 -0.198 -0.05 0.7 -7.522 -0.39 0.9

RS582598 -0.65 0.7 -0.198 -0.05 0.7 -7.522 -0.39 0.9

RS3896439 -0.65 0.7 -0.198 -0.05 0.7 -7.522 -0.39 0.9

RS875807 -0.65 0.7 -0.198 -0.05 0.7 -7.522 -0.39 0.9

RS7517857 -0.65 0.7 -0.198 -0.05 0.7 -7.522 -0.39 0.9

RS16839451 -0.65 0.7 -0.198 -0.05 0.7 -7.522 -0.39 0.9

RS2071995 -0.65 0.7 -0.198 -0.05 0.7 -7.522 -0.39 0.9

RS6688423 -0.65 0.7 -0.198 -0.05 0.7 -7.522 -0.39 0.9

RS242044 -0.65 0.7 -0.198 -0.05 0.7 -7.522 -0.39 0.9

RS10915626 -0.65 0.7 -0.198 -0.05 0.7 -7.522 -0.39 0.9

RS6683849 -0.65 0.7 -0.198 -0.05 0.7 -7.522 -0.39 0.9

RS10915668 -0.65 0.7 -0.198 -0.05 0.7 -7.522 -0.39 0.9

RS10915683 -0.65 0.7 -0.198 -0.05 0.7 -7.522 -0.39 0.9

RS9286971 -0.65 0.7 -0.198 -0.05 0.7 -7.522 -0.39 0.9

RS7535462 -0.65 0.7 -0.198 -0.05 0.7 -7.522 -0.39 0.9

RS7365286 -0.65 0.7 -0.198 -0.05 0.7 -7.522 -0.39 0.9

RS10158288 -0.65 0.7 -0.198 -0.05 0.7 -7.522 -0.39 0.9

RS6693990 -0.65 0.7 -0.198 -0.05 0.7 -7.522 -0.39 0.9

RS3128687 -0.65 0.7 -0.198 -0.05 0.7 -7.522 -0.39 0.9

RS10915341 -0.65 0.7 -0.198 -0.05 0.7 -7.522 -0.39 0.9

RS6671889 -0.65 0.7 -0.198 -0.05 0.7 -7.522 -0.39 0.9

RS7547229 -0.65 0.7 -0.198 -0.05 0.7 -7.522 -0.39 0.9

RS12135834 -0.65 0.7 -0.198 -0.05 0.7 -7.522 -0.39 0.9

RS6703948 -0.65 0.7 -0.198 -0.05 0.7 -7.522 -0.39 0.9

RS557477 -0.65 0.7 -0.198 -0.05 0.7 -7.522 -0.39 0.9

RS12040942 -0.65 0.7 -0.198 -0.05 0.7 -7.522 -0.39 0.9

RS12568815 -0.65 0.7 -0.198 -0.05 0.7 -7.522 -0.39 0.9

RS9659669 -0.65 0.7 -0.198 -0.05 0.7 -7.522 -0.39 0.9

RS1695645 -0.65 0.7 -0.198 -0.05 0.7 -7.522 -0.39 0.9

RS707582 -0.65 0.7 -0.198 -0.05 0.7 -7.522 -0.39 0.9

RS1414380 -0.65 0.7 -0.198 -0.05 0.7 -7.522 -0.39 0.9

RS2842258 -0.65 0.7 -0.198 -0.05 0.7 -7.522 -0.39 0.9

RS1287634 -0.65 0.7 -0.198 -0.05 0.7 -7.522 -0.39 0.9

RS546526 -0.65 0.7 -0.198 -0.05 0.7 -7.522 -0.39 0.9

RS2843495 -0.65 0.7 -0.198 -0.05 0.7 -7.522 -0.39 0.9

RS1883606 -0.65 0.7 -0.198 -0.05 0.7 -7.522 -0.39 0.9

RS4908863 -0.65 0.7 -0.198 -0.05 0.7 -7.522 -0.39 0.9

RS17029372 -0.65 0.7 -0.198 -0.05 0.7 -7.522 -0.39 0.9

RS4908554 -0.65 0.7 -0.198 -0.05 0.7 -7.522 -0.39 0.9

RS1043681 -0.65 0.7 -0.198 -0.05 0.7 -7.522 -0.39 0.9

RS201151 -0.65 0.7 -0.198 -0.05 0.7 -7.522 -0.39 0.9

RS447558 -0.65 0.7 -0.198 -0.05 0.7 -7.522 -0.39 0.9

RS11586867 -0.65 0.7 -0.198 -0.05 0.7 -7.522 -0.39 0.9

RS6577395 -0.65 0.7 -0.198 -0.05 0.7 -7.522 -0.39 0.9

RS6698901 -0.65 0.7 -0.198 -0.05 0.7 -7.522 -0.39 0.9

RS17030129 -0.65 0.7 -0.198 -0.05 0.7 -7.522 -0.39 0.9

RS11120840 -0.65 0.7 -0.198 -0.05 0.7 -7.522 -0.39 0.9

RS6694886 -0.65 0.7 -0.198 -0.05 0.7 -7.522 -0.39 0.9

RS970973 -0.64 0.7 -0.198 -0.05 0.7 -7.070 -0.37 0.9

RS12409413 -0.62 0.7 -0.198 -0.05 0.7 -5.437 -0.30 0.9

RS845270 -0.62 0.7 -0.198 -0.05 0.7 -5.437 -0.30 0.9

RS1193220 -0.62 0.7 -0.198 -0.05 0.7 -5.437 -0.30 0.9

RS1149331 -0.63 0.7 -0.198 -0.05 0.7 -6.073 -0.33 0.9

RS1193179 -0.64 0.7 -0.198 -0.05 0.7 -7.607 -0.37 0.9

RS3011937 -0.70 0.8 -0.198 -0.06 0.7 -9.999 -0.60 1.0

RS228666 -0.70 0.8 -0.198 -0.06 0.7 -9.999 -0.60 1.0

RS228648 -0.70 0.8 -0.198 -0.06 0.7 -9.999 -0.60 1.0

RS11582983 -0.70 0.8 -0.198 -0.06 0.7 -9.999 -0.60 1.0

RS225132 -0.70 0.8 -0.198 -0.06 0.7 -9.999 -0.60 1.0

RS299499 -0.70 0.8 -0.198 -0.06 0.7 -9.999 -0.60 1.0

RS7517675 -0.70 0.8 -0.198 -0.06 0.7 -9.999 -0.60 1.0

RS2274971 -0.70 0.8 -0.198 -0.06 0.7 -9.999 -0.60 1.0

RS10218541 -0.70 0.8 -0.198 -0.06 0.7 -9.999 -0.60 1.0

RS2274328 -0.70 0.8 -0.198 -0.06 0.7 -9.999 -0.60 1.0

RS875148 -0.70 0.8 -0.198 -0.06 0.7 -9.999 -0.60 1.0

RS12726360 -0.70 0.8 -0.198 -0.06 0.7 -9.999 -0.60 1.0

RS3753163 -0.70 0.8 -0.198 -0.06 0.7 -9.999 -0.60 1.0

RS916380 -0.70 0.8 -0.198 -0.06 0.7 -9.999 -0.60 1.0

RS621846 -0.70 0.8 -0.198 -0.06 0.7 -9.999 -0.60 1.0

RS9442562 -0.70 0.8 -0.198 -0.06 0.7 -9.999 -0.60 1.0

RS11121380 -0.70 0.8 -0.198 -0.06 0.7 -9.999 -0.60 1.0

RS2095904 -0.70 0.8 -0.198 -0.06 0.7 -9.999 -0.60 1.0

RS11121406 -0.70 0.8 -0.198 -0.06 0.7 -9.999 -0.60 1.0

RS10864418 -0.70 0.8 -0.198 -0.06 0.7 -9.999 -0.60 1.0

RS4926491 -0.70 0.8 -0.198 -0.06 0.7 -9.999 -0.60 1.0

RS7550613 -0.70 0.8 -0.198 -0.06 0.7 -9.999 -0.60 1.0

RS9430182 -0.70 0.8 -0.198 -0.06 0.7 -9.999 -0.60 1.0

RS9430506 -0.70 0.8 -0.198 -0.06 0.7 -9.999 -0.60 1.0

RS12028233 -0.70 0.8 -0.198 -0.06 0.7 -9.999 -0.60 1.0

RS4240895 -0.70 0.8 -0.198 -0.06 0.7 -9.999 -0.60 1.0

RS4333853 -0.70 0.8 -0.198 -0.06 0.7 -9.999 -0.60 1.0

RS12128766 -0.70 0.8 -0.198 -0.06 0.7 -9.999 -0.60 1.0

RS597441 -0.70 0.8 -0.198 -0.06 0.7 -9.999 -0.60 1.0

RS12565494 -0.66 0.7 -0.198 -0.05 0.7 -9.999 -0.48 0.9

RS3790630 -0.65 0.7 -0.198 -0.05 0.7 -9.999 -0.44 0.9

RS661352 -0.57 0.7 -0.198 -0.05 0.7 -3.689 -0.22 0.8

RS672270 -0.57 0.7 -0.198 -0.05 0.7 -3.440 -0.21 0.8

RS10157927 -0.57 0.7 -0.198 -0.05 0.7 -3.440 -0.21 0.8

RS6685896 -0.57 0.7 -0.198 -0.05 0.7 -3.440 -0.21 0.8

RS12565727 -0.57 0.7 -0.198 -0.05 0.7 -3.440 -0.21 0.8

RS2077480 -0.57 0.7 -0.198 -0.05 0.7 -3.440 -0.21 0.8

RS2922240 -0.57 0.7 -0.198 -0.05 0.7 -3.440 -0.21 0.8

RS12144924 -0.57 0.7 -0.198 -0.05 0.7 -3.440 -0.21 0.8

RS6540987 -0.57 0.7 -0.198 -0.05 0.7 -3.440 -0.21 0.8

RS9430624 -0.57 0.7 -0.198 -0.05 0.7 -3.440 -0.21 0.8

RS4846029 -0.57 0.7 -0.198 -0.05 0.7 -3.393 -0.21 0.8

RS17037520 -0.55 0.7 -0.198 -0.04 0.7 -2.955 -0.18 0.8

RS2273285 -0.55 0.7 -0.198 -0.04 0.7 -2.955 -0.18 0.8

RS2273291 -0.55 0.7 -0.198 -0.04 0.7 -2.955 -0.18 0.8

RS6665486 -0.55 0.7 -0.198 -0.04 0.7 -2.954 -0.18 0.8

RS12029016 -0.55 0.7 -0.198 -0.04 0.7 -2.954 -0.18 0.8

RS1201122 -0.55 0.7 -0.198 -0.04 0.7 -2.954 -0.18 0.8

RS630542 -0.55 0.7 -0.198 -0.04 0.7 -2.954 -0.18 0.8

RS235246 -0.55 0.7 -0.198 -0.04 0.7 -2.955 -0.18 0.8

RS4845904 -0.55 0.7 -0.198 -0.04 0.7 -2.954 -0.18 0.8

RS4997711 -0.55 0.7 -0.198 -0.04 0.7 -2.954 -0.18 0.8

RS12045736 -0.55 0.7 -0.198 -0.04 0.7 -2.954 -0.18 0.8

RS3000858 -0.55 0.7 -0.198 -0.04 0.7 -2.954 -0.18 0.8

RS4846143 -0.55 0.7 -0.198 -0.04 0.7 -2.954 -0.18 0.8

RS11122001 -0.55 0.7 -0.198 -0.04 0.7 -2.954 -0.18 0.8

RS3013083 -0.55 0.7 -0.198 -0.04 0.7 -2.954 -0.18 0.8

RS6684199 -0.55 0.7 -0.198 -0.04 0.7 -2.954 -0.18 0.8

RS3820304 -0.55 0.7 -0.198 -0.04 0.7 -2.954 -0.18 0.8

RS10927926 -0.55 0.7 -0.198 -0.04 0.7 -2.954 -0.18 0.8

RS4077841 -0.55 0.7 -0.198 -0.04 0.7 -2.954 -0.18 0.8

RS2244634 -0.55 0.7 -0.198 -0.04 0.7 -2.954 -0.18 0.8

RS982510 -0.55 0.7 -0.198 -0.04 0.7 -2.954 -0.18 0.8

RS1528786 -0.55 0.7 -0.198 -0.04 0.7 -2.954 -0.18 0.8

RS2744647 -0.55 0.7 -0.198 -0.04 0.7 -2.954 -0.18 0.8

RS12122426 -0.55 0.7 -0.198 -0.04 0.7 -2.954 -0.18 0.8

RS7542884 -0.55 0.7 -0.198 -0.04 0.7 -2.954 -0.18 0.8

RS12024045 -0.55 0.7 -0.198 -0.04 0.7 -2.954 -0.18 0.8

RS12129723 -0.55 0.7 -0.198 -0.04 0.7 -2.954 -0.18 0.8

RS2206586 -0.55 0.7 -0.198 -0.04 0.7 -2.954 -0.18 0.8

RS2359908 -0.55 0.7 -0.198 -0.04 0.7 -2.954 -0.18 0.8

RS10928004 -0.55 0.7 -0.198 -0.04 0.7 -2.954 -0.18 0.8

RS6671072 -0.55 0.7 -0.198 -0.04 0.7 -2.954 -0.18 0.8

RS1416628 -0.55 0.7 -0.198 -0.04 0.7 -2.954 -0.18 0.8

RS2789746 -0.55 0.7 -0.198 -0.04 0.7 -2.954 -0.18 0.8

RS4661496 -0.55 0.7 -0.198 -0.04 0.7 -2.954 -0.18 0.8

RS2486774 -0.55 0.7 -0.198 -0.04 0.7 -2.954 -0.18 0.8

RS12031064 -0.55 0.7 -0.198 -0.04 0.7 -2.954 -0.18 0.8

RS3892594 -0.55 0.7 -0.198 -0.04 0.7 -2.954 -0.18 0.8

RS2105239 -0.55 0.7 -0.198 -0.04 0.7 -2.954 -0.18 0.8

RS761191 -0.55 0.7 -0.198 -0.04 0.7 -2.954 -0.18 0.8

RS1997762 -0.55 0.7 -0.198 -0.04 0.7 -2.954 -0.18 0.8

RS4543785 -0.55 0.7 -0.198 -0.04 0.7 -2.954 -0.18 0.8

RS732469 -0.55 0.7 -0.198 -0.04 0.7 -2.954 -0.18 0.8

RS3845590 -0.55 0.7 -0.198 -0.04 0.7 -2.954 -0.18 0.8

RS11585327 -0.55 0.7 -0.198 -0.04 0.7 -2.954 -0.18 0.8

RS12564579 -0.55 0.7 -0.198 -0.04 0.7 -2.954 -0.18 0.8

RS6429696 -0.55 0.7 -0.198 -0.04 0.7 -2.955 -0.18 0.8

RS10927627 -0.55 0.7 -0.198 -0.04 0.7 -2.955 -0.18 0.8

RS16851241 -0.55 0.7 -0.198 -0.04 0.7 -2.954 -0.18 0.8

RS4661572 -0.55 0.7 -0.198 -0.04 0.7 -2.954 -0.18 0.8

RS761301 -0.55 0.7 -0.198 -0.04 0.7 -2.954 -0.18 0.8

RS12145963 -0.55 0.7 -0.198 -0.04 0.7 -2.954 -0.18 0.8

RS16851480 -0.55 0.7 -0.198 -0.04 0.7 -2.954 -0.18 0.8

RS6676098 -0.55 0.7 -0.198 -0.04 0.7 -2.954 -0.18 0.8

RS10927764 -0.55 0.7 -0.198 -0.04 0.7 -2.954 -0.18 0.8

RS733887 -0.55 0.7 -0.198 -0.04 0.7 -2.954 -0.18 0.8

RS6693417 -0.55 0.7 -0.198 -0.04 0.7 -2.954 -0.18 0.8

RS10927796 -0.55 0.7 -0.198 -0.04 0.7 -2.954 -0.18 0.8

RS1010069 -0.55 0.7 -0.198 -0.04 0.7 -2.954 -0.18 0.8

RS9442193 -0.55 0.7 -0.198 -0.04 0.7 -2.954 -0.18 0.8

RS4661351 -0.55 0.7 -0.198 -0.04 0.7 -2.954 -0.18 0.8

RS221031 -0.55 0.7 -0.198 -0.04 0.7 -2.954 -0.18 0.8

RS221035 -0.55 0.7 -0.198 -0.04 0.7 -2.954 -0.18 0.8

RS6698317 -0.55 0.7 -0.198 -0.04 0.7 -2.954 -0.18 0.8

RS6586513 -0.55 0.7 -0.198 -0.04 0.7 -2.954 -0.18 0.8

RS761422 -0.55 0.7 -0.198 -0.04 0.7 -2.954 -0.18 0.8

RS4920442 -0.55 0.7 -0.198 -0.04 0.7 -2.954 -0.18 0.8

RS2014725 -0.55 0.7 -0.198 -0.04 0.7 -2.954 -0.18 0.8

RS12408879 -0.55 0.7 -0.198 -0.04 0.7 -2.954 -0.18 0.8

RS3003406 -0.55 0.7 -0.198 -0.04 0.7 -2.954 -0.18 0.8

RS2977227 -0.55 0.7 -0.198 -0.04 0.7 -2.954 -0.18 0.8

RS874881 -0.55 0.7 -0.198 -0.04 0.7 -2.954 -0.18 0.8

RS6695214 -0.55 0.7 -0.198 -0.04 0.7 -2.954 -0.18 0.8

RS2883272 -0.55 0.7 -0.198 -0.04 0.7 -2.954 -0.18 0.8

RS6689677 -0.55 0.7 -0.198 -0.04 0.7 -2.954 -0.18 0.8

RS4920374 -0.55 0.7 -0.198 -0.04 0.7 -2.954 -0.18 0.8

RS4920379 -0.55 0.7 -0.198 -0.04 0.7 -2.954 -0.18 0.8

RS16830757 -0.55 0.7 -0.198 -0.04 0.7 -2.954 -0.18 0.8

RS561698 -0.55 0.7 -0.198 -0.04 0.7 -2.954 -0.18 0.8

RS620547 -0.55 0.7 -0.198 -0.04 0.7 -2.955 -0.18 0.8

RS11203232 -0.55 0.7 -0.198 -0.04 0.7 -2.955 -0.18 0.8

RS794230 -0.55 0.7 -0.198 -0.04 0.7 -2.955 -0.18 0.8

RS2095842 -0.55 0.7 -0.198 -0.04 0.7 -2.955 -0.18 0.8

RS12022529 -0.55 0.7 -0.198 -0.04 0.7 -2.955 -0.18 0.8

RS12740505 -0.55 0.7 -0.198 -0.04 0.7 -2.954 -0.18 0.8

RS4920435 -0.55 0.7 -0.198 -0.04 0.7 -2.954 -0.18 0.8

RS10907331 -0.55 0.7 -0.198 -0.04 0.7 -2.954 -0.18 0.8

RS749917 -0.55 0.7 -0.198 -0.04 0.7 -2.954 -0.18 0.8

RS4920459 -0.55 0.7 -0.198 -0.04 0.7 -2.954 -0.18 0.8

RS419971 -0.55 0.7 -0.198 -0.04 0.7 -2.954 -0.18 0.8

RS6663855 -0.55 0.7 -0.198 -0.04 0.7 -2.954 -0.18 0.8

RS552749 -0.55 0.7 -0.198 -0.04 0.7 -2.954 -0.18 0.8

RS542634 -0.55 0.7 -0.198 -0.04 0.7 -2.954 -0.18 0.8

RS12128025 -0.55 0.7 -0.198 -0.04 0.7 -2.954 -0.18 0.8

RS3007722 -0.55 0.7 -0.198 -0.04 0.7 -2.954 -0.18 0.8

RS10907314 -0.55 0.7 -0.198 -0.04 0.7 -2.954 -0.18 0.8

RS4920522 -0.55 0.7 -0.198 -0.04 0.7 -2.954 -0.18 0.8

RS2743201 -0.55 0.7 -0.198 -0.04 0.7 -2.954 -0.18 0.8

RS6670751 -0.55 0.7 -0.198 -0.04 0.7 -2.954 -0.18 0.8

RS669138 -0.55 0.7 -0.198 -0.04 0.7 -2.954 -0.18 0.8

RS12026106 -0.55 0.7 -0.198 -0.04 0.7 -2.954 -0.18 0.8

RS4912064 -0.55 0.7 -0.198 -0.04 0.7 -2.954 -0.18 0.8

RS214327 -0.55 0.7 -0.198 -0.04 0.7 -2.954 -0.18 0.8

RS2314146 -0.55 0.7 -0.198 -0.04 0.7 -2.954 -0.18 0.8

RS11807599 -0.55 0.7 -0.198 -0.04 0.7 -2.954 -0.18 0.8

RS1572823 -0.55 0.7 -0.198 -0.04 0.7 -2.954 -0.18 0.8

RS1115163 -0.55 0.7 -0.198 -0.04 0.7 -2.954 -0.18 0.8

RS4486471 -0.55 0.7 -0.198 -0.04 0.7 -2.954 -0.18 0.8

RS1266438 -0.55 0.7 -0.198 -0.04 0.7 -2.954 -0.18 0.8

RS3806310 -0.55 0.7 -0.198 -0.04 0.7 -2.954 -0.18 0.8

RS4654903 -0.55 0.7 -0.198 -0.04 0.7 -2.954 -0.18 0.8

RS1796915 -0.55 0.7 -0.198 -0.04 0.7 -2.954 -0.18 0.8

RS6672057 -0.55 0.7 -0.198 -0.04 0.7 -2.954 -0.18 0.8

RS876171 -0.55 0.7 -0.198 -0.04 0.7 -2.954 -0.18 0.8

RS2301478 -0.55 0.7 -0.198 -0.04 0.7 -2.954 -0.18 0.8

RS10916732 -0.55 0.7 -0.198 -0.04 0.7 -2.954 -0.18 0.8

RS10916749 -0.55 0.7 -0.198 -0.04 0.7 -2.954 -0.18 0.8

RS10753498 -0.55 0.7 -0.198 -0.04 0.7 -2.954 -0.18 0.8

RS7513147 -0.55 0.7 -0.198 -0.04 0.7 -2.954 -0.18 0.8

RS576436 -0.55 0.7 -0.198 -0.04 0.7 -2.954 -0.18 0.8

RS1152984 -0.55 0.7 -0.198 -0.04 0.7 -2.954 -0.18 0.8

RS472055 -0.55 0.7 -0.198 -0.04 0.7 -2.954 -0.18 0.8

RS1890005 -0.55 0.7 -0.198 -0.04 0.7 -2.954 -0.18 0.8

RS12036909 -0.55 0.7 -0.198 -0.04 0.7 -2.954 -0.18 0.8

RS10916848 -0.55 0.7 -0.198 -0.04 0.7 -2.954 -0.18 0.8

RS16824489 -0.55 0.7 -0.198 -0.04 0.7 -2.954 -0.18 0.8

RS7552264 -0.55 0.7 -0.198 -0.04 0.7 -2.954 -0.18 0.8

RS7520481 -0.55 0.7 -0.198 -0.04 0.7 -2.954 -0.18 0.8

RS2174689 -0.55 0.7 -0.198 -0.04 0.7 -2.955 -0.18 0.8

RS3026913 -0.55 0.7 -0.198 -0.04 0.7 -2.954 -0.18 0.8

RS213032 -0.55 0.7 -0.198 -0.04 0.7 -2.954 -0.18 0.8

RS785198 -0.55 0.7 -0.198 -0.04 0.7 -2.954 -0.18 0.8

RS1827293 -0.55 0.7 -0.198 -0.04 0.7 -2.954 -0.18 0.8

RS1472563 -0.55 0.7 -0.198 -0.04 0.7 -2.954 -0.18 0.8

RS3767155 -0.55 0.7 -0.198 -0.04 0.7 -2.954 -0.18 0.8

RS2275363 -0.55 0.7 -0.198 -0.04 0.7 -2.954 -0.18 0.8

RS829404 -0.55 0.7 -0.198 -0.04 0.7 -2.954 -0.18 0.8

RS7544500 -0.55 0.7 -0.198 -0.04 0.7 -2.955 -0.18 0.8

RS10489437 -0.55 0.7 -0.198 -0.04 0.7 -2.954 -0.18 0.8

RS10917165 -0.55 0.7 -0.198 -0.04 0.7 -2.954 -0.18 0.8

RS2505722 -0.55 0.7 -0.198 -0.04 0.7 -2.954 -0.18 0.8

RS2744750 -0.55 0.7 -0.198 -0.04 0.7 -2.954 -0.18 0.8

RS6704135 -0.55 0.7 -0.198 -0.04 0.7 -2.954 -0.18 0.8

RS4655059 -0.55 0.7 -0.198 -0.04 0.7 -2.954 -0.18 0.8

RS10493014 -0.55 0.7 -0.198 -0.04 0.7 -2.954 -0.18 0.8

RS766910 -0.55 0.7 -0.198 -0.04 0.7 -2.954 -0.18 0.8

RS158762 -0.55 0.7 -0.198 -0.04 0.7 -2.954 -0.18 0.8

RS17486657 -0.55 0.7 -0.198 -0.04 0.7 -2.954 -0.18 0.8

RS4655093 -0.55 0.7 -0.198 -0.04 0.7 -2.954 -0.18 0.8

RS2817909 -0.55 0.7 -0.198 -0.04 0.7 -2.954 -0.18 0.8

RS4390147 -0.55 0.7 -0.198 -0.04 0.7 -2.954 -0.18 0.8

RS309476 -0.55 0.7 -0.198 -0.04 0.7 -2.954 -0.18 0.8

RS648322 -0.55 0.7 -0.198 -0.04 0.7 -2.954 -0.18 0.8

RS7522616 -0.55 0.7 -0.198 -0.04 0.7 -2.954 -0.18 0.8

RS2282720 -0.55 0.7 -0.198 -0.04 0.7 -2.954 -0.18 0.8

RS1050096 -0.55 0.7 -0.198 -0.04 0.7 -2.954 -0.18 0.8

RS2811946 -0.55 0.7 -0.198 -0.04 0.7 -2.954 -0.18 0.8

RS2864884 -0.55 0.7 -0.198 -0.04 0.7 -2.954 -0.18 0.8

RS2280437 -0.55 0.7 -0.198 -0.04 0.7 -2.954 -0.18 0.8

RS4649175 -0.55 0.7 -0.198 -0.04 0.7 -2.954 -0.18 0.8

RS7524076 -0.55 0.7 -0.198 -0.04 0.7 -2.954 -0.18 0.8

RS11249045 -0.55 0.7 -0.198 -0.04 0.7 -2.954 -0.18 0.8

RS6670533 -0.55 0.7 -0.198 -0.04 0.7 -2.954 -0.18 0.8

RS7535789 -0.55 0.7 -0.198 -0.04 0.7 -2.954 -0.18 0.8

RS4648960 -0.55 0.7 -0.198 -0.04 0.7 -2.954 -0.18 0.8

RS2248219 -0.55 0.7 -0.198 -0.04 0.7 -2.954 -0.18 0.8

RS2272935 -0.55 0.7 -0.198 -0.04 0.7 -2.954 -0.18 0.8

RS195712 -0.55 0.7 -0.198 -0.04 0.7 -2.954 -0.18 0.8

RS4610948 -0.55 0.7 -0.198 -0.04 0.7 -2.954 -0.18 0.8

RS11249138 -0.55 0.7 -0.198 -0.04 0.7 -2.954 -0.18 0.8

RS4649002 -0.55 0.7 -0.198 -0.04 0.7 -2.954 -0.18 0.8

RS742230 -0.55 0.7 -0.198 -0.04 0.7 -2.954 -0.18 0.8

RS11249215 -0.55 0.7 -0.198 -0.04 0.7 -2.954 -0.18 0.8

RS9438880 -0.55 0.7 -0.198 -0.04 0.7 -2.954 -0.18 0.8

RS7520145 -0.55 0.7 -0.198 -0.04 0.7 -2.954 -0.18 0.8

RS4568778 -0.55 0.7 -0.198 -0.04 0.7 -2.954 -0.18 0.8

RS2660353 -0.55 0.7 -0.198 -0.04 0.7 -2.954 -0.18 0.8

RS742390 -0.55 0.7 -0.198 -0.04 0.7 -2.954 -0.18 0.8

RS12096438 -0.55 0.7 -0.198 -0.04 0.7 -2.954 -0.18 0.8

RS3820515 -0.55 0.7 -0.198 -0.04 0.7 -2.954 -0.18 0.8

RS7524154 -0.55 0.7 -0.198 -0.04 0.7 -2.954 -0.18 0.8

RS10794523 -0.55 0.7 -0.198 -0.04 0.7 -2.954 -0.18 0.8

RS4659411 -0.55 0.7 -0.198 -0.04 0.7 -2.954 -0.18 0.8

RS12096059 -0.55 0.7 -0.198 -0.04 0.7 -2.954 -0.18 0.8

RS4659431 -0.55 0.7 -0.198 -0.04 0.7 -2.954 -0.18 0.8

RS11247919 -0.55 0.7 -0.198 -0.04 0.7 -2.954 -0.18 0.8

RS7547496 -0.56 0.7 -0.198 -0.05 0.7 -3.157 -0.19 0.8

RS9438621 -0.56 0.7 -0.198 -0.05 0.7 -3.482 -0.21 0.8

RS12741800 -0.58 0.7 -0.198 -0.05 0.7 -4.588 -0.24 0.9

RS11247963 -0.58 0.7 -0.198 -0.05 0.7 -5.851 -0.26 0.9

RS17356923 -0.63 0.7 -0.198 -0.05 0.7 -9.999 -0.49 0.9

RS12565684 -0.67 0.7 -0.198 -0.05 0.7 -9.999 -0.61 1.0

RS6598891 -0.68 0.8 -0.198 -0.05 0.7 -9.999 -0.64 1.0

RS6676158 -0.70 0.8 -0.198 -0.06 0.7 -9.999 -0.68 1.0

RS4256835 -0.70 0.8 -0.198 -0.06 0.7 -9.999 -0.68 1.0

RS12565238 -0.70 0.8 -0.198 -0.06 0.7 -9.999 -0.68 1.0

RS6598919 -0.70 0.8 -0.198 -0.06 0.7 -9.999 -0.69 1.0

RS508772 -0.71 0.8 -0.198 -0.06 0.7 -9.999 -0.70 1.0

RS17163135 -0.71 0.8 -0.198 -0.06 0.7 -9.999 -0.71 1.0

RS12749204 -0.74 0.8 -0.198 -0.06 0.7 -9.999 -0.77 1.0

RS12032470 -0.74 0.8 -0.198 -0.06 0.7 -9.999 -0.77 1.0

RS2503011 -0.74 0.8 -0.198 -0.06 0.7 -9.999 -0.77 1.0

RS2007350 -0.74 0.8 -0.198 -0.06 0.7 -9.999 -0.77 1.0

RS212306 -0.74 0.8 -0.198 -0.06 0.7 -9.999 -0.77 1.0

RS1408857 -0.74 0.8 -0.198 -0.06 0.7 -9.999 -0.77 1.0

RS927727 -0.74 0.8 -0.198 -0.06 0.7 -9.999 -0.77 1.0

RS199613 -0.74 0.8 -0.198 -0.06 0.7 -9.999 -0.77 1.0

RS1441834 -0.74 0.8 -0.198 -0.06 0.7 -9.999 -0.77 1.0

RS894220 -0.74 0.8 -0.198 -0.06 0.7 -9.999 -0.77 1.0

RS6691847 -0.74 0.8 -0.198 -0.06 0.7 -9.999 -0.77 1.0

RS4949485 -0.74 0.8 -0.198 -0.06 0.7 -9.999 -0.77 1.0

RS12083826 -0.74 0.8 -0.198 -0.06 0.7 -9.999 -0.77 1.0

RS895796 -0.74 0.8 -0.198 -0.06 0.7 -9.999 -0.77 1.0

RS10914615 -0.74 0.8 -0.198 -0.06 0.7 -9.999 -0.77 1.0

RS267720 -0.74 0.8 -0.198 -0.06 0.7 -9.999 -0.77 1.0

RS1746272 -0.74 0.8 -0.198 -0.06 0.7 -9.999 -0.77 1.0

RS3934202 -0.74 0.8 -0.198 -0.06 0.7 -9.999 -0.77 1.0

RS609878 -0.74 0.8 -0.198 -0.06 0.7 -9.999 -0.77 1.0

RS6673363 -0.74 0.8 -0.198 -0.06 0.7 -9.999 -0.77 1.0

RS12406008 -0.74 0.8 -0.198 -0.06 0.7 -9.999 -0.77 1.0

RS415314 -0.74 0.8 -0.198 -0.06 0.7 -9.999 -0.77 1.0

RS396648 -0.74 0.8 -0.198 -0.06 0.7 -9.999 -0.77 1.0

RS525813 -0.74 0.8 -0.198 -0.06 0.7 -9.999 -0.77 1.0

RS11589302 -0.74 0.8 -0.198 -0.06 0.7 -9.999 -0.77 1.0

RS522294 -0.74 0.8 -0.198 -0.06 0.7 -9.999 -0.77 1.0

RS7555715 -0.74 0.8 -0.198 -0.06 0.7 -9.999 -0.77 1.0

RS1188357 -0.74 0.8 -0.198 -0.06 0.7 -9.999 -0.77 1.0

RS746700 -0.74 0.8 -0.198 -0.06 0.7 -9.999 -0.77 1.0

RS2488230 -0.74 0.8 -0.198 -0.06 0.7 -9.999 -0.77 1.0

RS2488233 -0.74 0.8 -0.198 -0.06 0.7 -9.999 -0.77 1.0

RS2488238 -0.74 0.8 -0.198 -0.06 0.7 -9.999 -0.77 1.0

RS2275741 -0.74 0.8 -0.198 -0.06 0.7 -9.999 -0.77 1.0

RS947643 -0.74 0.8 -0.198 -0.06 0.7 -9.999 -0.77 1.0

RS10914375 -0.74 0.8 -0.198 -0.06 0.7 -9.999 -0.77 1.0

RS4949416 -0.74 0.8 -0.198 -0.06 0.7 -9.999 -0.77 1.0

RS10914420 -0.74 0.8 -0.198 -0.06 0.7 -9.999 -0.77 1.0

RS10914453 -0.74 0.8 -0.198 -0.06 0.7 -9.999 -0.77 1.0

RS3818788 -0.74 0.8 -0.198 -0.06 0.7 -9.999 -0.77 1.0

RS6669563 -0.74 0.8 -0.198 -0.06 0.7 -9.999 -0.77 1.0

RS12565351 -0.74 0.8 -0.198 -0.06 0.7 -9.999 -0.77 1.0

RS687605 -0.74 0.8 -0.198 -0.06 0.7 -9.999 -0.77 1.0

RS669538 -0.74 0.8 -0.198 -0.06 0.7 -9.999 -0.77 1.0

RS644879 -0.74 0.8 -0.198 -0.06 0.7 -9.999 -0.77 1.0

RS4361958 -0.74 0.8 -0.198 -0.06 0.7 -9.999 -0.77 1.0

RS10798962 -0.74 0.8 -0.198 -0.06 0.7 -9.999 -0.77 1.0

RS10914746 -0.74 0.8 -0.198 -0.06 0.7 -9.999 -0.77 1.0

RS1874045 -0.74 0.8 -0.198 -0.06 0.7 -9.999 -0.77 1.0

RS649214 -0.74 0.8 -0.198 -0.06 0.7 -9.999 -0.77 1.0

RS565953 -0.74 0.8 -0.198 -0.06 0.7 -9.999 -0.77 1.0

RS544991 -0.74 0.8 -0.198 -0.06 0.7 -9.999 -0.77 1.0

RS12408037 -0.74 0.8 -0.198 -0.06 0.7 -9.999 -0.77 1.0

RS2180745 -0.74 0.8 -0.198 -0.06 0.7 -9.999 -0.77 1.0

RS911216 -0.74 0.8 -0.198 -0.06 0.7 -9.999 -0.77 1.0

RS749733 -0.74 0.8 -0.198 -0.06 0.7 -9.999 -0.77 1.0

RS487472 -0.74 0.8 -0.198 -0.06 0.7 -9.999 -0.77 1.0

RS1325258 -0.74 0.8 -0.198 -0.06 0.7 -9.999 -0.77 1.0

RS1465782 -0.74 0.8 -0.198 -0.06 0.7 -9.999 -0.77 1.0

RS7552457 -0.74 0.8 -0.198 -0.06 0.7 -9.999 -0.77 1.0

RS12043879 -0.74 0.8 -0.198 -0.06 0.7 -9.999 -0.77 1.0

RS771404 -0.74 0.8 -0.198 -0.06 0.7 -9.999 -0.77 1.0

RS7530700 -0.74 0.8 -0.198 -0.06 0.7 -9.999 -0.77 1.0

RS4320749 -0.74 0.8 -0.198 -0.06 0.7 -9.999 -0.77 1.0

RS10489658 -0.74 0.8 -0.198 -0.06 0.7 -9.999 -0.77 1.0

RS2093185 -0.74 0.8 -0.198 -0.06 0.7 -9.999 -0.77 1.0

RS4653091 -0.74 0.8 -0.198 -0.06 0.7 -9.999 -0.77 1.0

RS564226 -0.74 0.8 -0.198 -0.06 0.7 -9.999 -0.77 1.0

RS1407135 -0.74 0.8 -0.198 -0.06 0.7 -9.999 -0.77 1.0

RS4652869 -0.72 0.8 -0.198 -0.06 0.7 -9.999 -0.73 1.0

RS4411101 -0.68 0.8 -0.198 -0.05 0.7 -9.999 -0.64 1.0

RS4653160 -0.58 0.7 -0.198 -0.05 0.7 -9.999 -0.30 0.9

RS12116935 -0.56 0.7 -0.198 -0.05 0.7 -4.355 -0.22 0.8

RS4653185 -0.55 0.7 -0.198 -0.04 0.7 -3.418 -0.19 0.8

RS6667450 -0.53 0.7 -0.198 -0.04 0.7 -2.674 -0.17 0.8

RS17498436 -0.53 0.7 -0.198 -0.04 0.7 -2.437 -0.15 0.8

RS4653204 -0.53 0.7 -0.198 -0.04 0.7 -2.437 -0.15 0.8

RS218990 -0.53 0.7 -0.198 -0.04 0.7 -2.437 -0.15 0.8

RS11576073 -0.53 0.7 -0.198 -0.04 0.7 -2.437 -0.15 0.8

RS2859741 -0.53 0.7 -0.198 -0.04 0.7 -2.437 -0.15 0.8

RS6702349 -0.53 0.7 -0.198 -0.04 0.7 -2.437 -0.15 0.8

RS215791 -0.53 0.7 -0.198 -0.04 0.7 -2.437 -0.15 0.8

RS6691915 -0.53 0.7 -0.198 -0.04 0.7 -2.437 -0.15 0.8

RS11582151 -0.53 0.7 -0.198 -0.04 0.7 -2.437 -0.15 0.8

RS4428850 -0.53 0.7 -0.198 -0.04 0.7 -2.437 -0.15 0.8

RS10908350 -0.53 0.7 -0.198 -0.04 0.7 -2.437 -0.15 0.8

RS6700586 -0.53 0.7 -0.198 -0.04 0.7 -2.437 -0.15 0.8

RS7552071 -0.53 0.7 -0.198 -0.04 0.7 -2.437 -0.15 0.8

RS11264082 -0.53 0.7 -0.198 -0.04 0.7 -2.437 -0.15 0.8

RS663428 -0.53 0.7 -0.198 -0.04 0.7 -2.437 -0.15 0.8

RS489408 -0.53 0.7 -0.198 -0.04 0.7 -2.437 -0.15 0.8

RS12751325 -0.53 0.7 -0.198 -0.04 0.7 -2.437 -0.15 0.8

RS6600436 -0.53 0.7 -0.198 -0.04 0.7 -2.437 -0.15 0.8

RS569791 -0.53 0.7 -0.198 -0.04 0.7 -2.437 -0.15 0.8

RS2615811 -0.53 0.7 -0.198 -0.04 0.7 -2.437 -0.15 0.8

RS898982 -0.53 0.7 -0.198 -0.04 0.7 -2.437 -0.15 0.8

RS6675079 -0.53 0.7 -0.198 -0.04 0.7 -2.437 -0.15 0.8

RS10890173 -0.53 0.7 -0.198 -0.04 0.7 -2.437 -0.15 0.8

RS2040073 -0.53 0.7 -0.198 -0.04 0.7 -2.437 -0.15 0.8

RS12132779 -0.53 0.7 -0.198 -0.04 0.7 -2.437 -0.15 0.8

RS4970591 -0.53 0.7 -0.198 -0.04 0.7 -2.437 -0.15 0.8

RS882000 -0.50 0.7 -0.198 -0.04 0.7 -2.059 -0.13 0.8

RS2992434 -0.50 0.7 -0.198 -0.04 0.7 -2.059 -0.13 0.8

RS6687438 -0.50 0.7 -0.198 -0.04 0.7 -2.059 -0.13 0.8

RS10789490 -0.50 0.7 -0.198 -0.04 0.7 -2.059 -0.13 0.8

RS6675193 -0.48 0.7 -0.198 -0.04 0.7 -1.950 -0.12 0.8

RS4491033 -0.09 0.5 -0.198 -0.01 0.6 -0.110 -0.00 0.5

RS4660652 -0.09 0.5 -0.198 -0.01 0.6 -0.110 -0.00 0.5

RS11205665 -0.09 0.5 -0.198 -0.01 0.6 -0.110 -0.00 0.5

RS1537818 -0.09 0.5 -0.198 -0.01 0.6 -0.110 -0.00 0.5

RS687848 -0.09 0.5 -0.198 -0.01 0.6 -0.111 -0.00 0.5

RS4660892 -0.09 0.5 -0.198 -0.01 0.6 -0.111 -0.00 0.5

RS784634 -0.09 0.5 -0.198 -0.01 0.6 -0.111 -0.00 0.5

RS1046988 -0.09 0.5 -0.198 -0.01 0.6 -0.111 -0.00 0.5

RS2676697 -0.09 0.5 -0.198 -0.01 0.6 -0.111 -0.00 0.5

RS10489534 -0.09 0.5 -0.198 -0.01 0.6 -0.111 -0.00 0.5

RS3103778 -0.09 0.5 -0.198 -0.01 0.6 -0.112 -0.00 0.5

RS3737821 -0.10 0.5 -0.198 -0.01 0.6 -0.112 -0.00 0.5

RS209583 -0.10 0.5 -0.198 -0.01 0.6 -0.112 -0.00 0.5

RS2982500 -0.10 0.5 -0.198 -0.01 0.6 -0.112 -0.00 0.5

RS12568089 -0.10 0.5 -0.198 -0.01 0.6 -0.112 -0.00 0.5

RS4660438 -0.10 0.5 -0.198 -0.01 0.6 -0.112 -0.00 0.5

RS11208727 -0.10 0.5 -0.198 -0.01 0.6 -0.113 -0.00 0.5

RS4660464 -0.10 0.5 -0.198 -0.01 0.6 -0.113 -0.00 0.5

RS6697721 -0.10 0.5 -0.198 -0.01 0.6 -0.113 -0.00 0.5

RS3767943 -0.10 0.5 -0.198 -0.01 0.6 -0.113 -0.00 0.5

RS11209213 -0.10 0.5 -0.198 -0.01 0.6 -0.113 -0.00 0.5

RS10789262 -0.10 0.5 -0.198 -0.01 0.6 -0.113 -0.00 0.5

RS4660184 -0.10 0.5 -0.198 -0.01 0.6 -0.113 -0.00 0.5

RS3819842 -0.10 0.5 -0.198 -0.01 0.6 -0.113 -0.00 0.5

RS12030183 -0.10 0.5 -0.198 -0.01 0.6 -0.113 -0.00 0.5

RS7349069 -0.10 0.5 -0.198 -0.01 0.6 -0.114 -0.00 0.5

RS4660531 -0.10 0.5 -0.198 -0.01 0.6 -0.114 -0.00 0.5

RS2483692 -0.10 0.5 -0.198 -0.01 0.6 -0.114 -0.00 0.5

RS12718439 -0.10 0.5 -0.198 -0.01 0.6 -0.114 -0.00 0.5

RS12043801 -0.10 0.5 -0.198 -0.01 0.6 -0.114 -0.00 0.5

RS10890160 -0.10 0.5 -0.198 -0.01 0.6 -0.114 -0.00 0.5

RS2492604 -0.10 0.5 -0.198 -0.01 0.6 -0.116 -0.00 0.5

RS10493098 -0.10 0.5 -0.198 -0.01 0.6 -0.116 -0.00 0.5

RS168382 -0.10 0.5 -0.198 -0.01 0.6 -0.116 -0.00 0.5

RS2993120 -0.10 0.5 -0.198 -0.01 0.6 -0.118 -0.00 0.5

RS9943167 -0.10 0.5 -0.198 -0.01 0.6 -0.126 -0.00 0.5

RS12039696 -0.12 0.5 -0.198 -0.01 0.6 -0.158 -0.00 0.6

RS2762697 -0.13 0.6 -0.198 -0.01 0.6 -0.176 -0.00 0.6

RS1951918 -0.14 0.6 -0.198 -0.01 0.6 -0.198 -0.01 0.6

RS7548008 -0.16 0.6 -0.198 -0.01 0.6 -0.240 -0.01 0.6

RS626842 -0.27 0.6 -0.198 -0.02 0.6 -0.672 -0.03 0.6

RS10493115 -0.32 0.6 -0.198 -0.03 0.6 -9.999 -0.13 0.8

RS2991996 -0.33 0.6 -0.198 -0.03 0.6 -9.999 -0.14 0.8

RS7413861 -0.45 0.7 -0.198 -0.04 0.7 -9.999 -0.28 0.9

RS7528454 -0.61 0.7 -0.198 -0.05 0.7 -5.041 -0.29 0.9

RS12759480 -0.61 0.7 -0.198 -0.05 0.7 -5.041 -0.29 0.9

RS12022308 -0.61 0.7 -0.198 -0.05 0.7 -5.041 -0.29 0.9

RS7553056 -0.61 0.7 -0.198 -0.05 0.7 -5.041 -0.29 0.9

RS7354900 -0.61 0.7 -0.198 -0.05 0.7 -5.041 -0.29 0.9

RS782135 -0.61 0.7 -0.198 -0.05 0.7 -5.041 -0.29 0.9

RS270709 -0.61 0.7 -0.198 -0.05 0.7 -5.041 -0.29 0.9

RS264025 -0.61 0.7 -0.198 -0.05 0.7 -5.042 -0.29 0.9

RS11211262 -0.61 0.7 -0.198 -0.05 0.7 -5.041 -0.29 0.9

RS11211282 -0.61 0.7 -0.198 -0.05 0.7 -5.041 -0.29 0.9

RS4660957 -0.61 0.7 -0.198 -0.05 0.7 -5.041 -0.29 0.9

RS594387 -0.61 0.7 -0.198 -0.05 0.7 -5.041 -0.29 0.9

RS986034 -0.61 0.7 -0.198 -0.05 0.7 -5.041 -0.29 0.9

RS10749865 -0.61 0.7 -0.198 -0.05 0.7 -5.041 -0.29 0.9

RS9793989 -0.61 0.7 -0.198 -0.05 0.7 -5.041 -0.29 0.9

RS6690005 -0.61 0.7 -0.198 -0.05 0.7 -5.041 -0.29 0.9

RS2065945 -0.61 0.7 -0.198 -0.05 0.7 -5.041 -0.29 0.9

RS12028069 -0.61 0.7 -0.198 -0.05 0.7 -5.041 -0.29 0.9

RS10890492 -0.61 0.7 -0.198 -0.05 0.7 -5.041 -0.29 0.9

RS2282361 -0.61 0.7 -0.198 -0.05 0.7 -5.041 -0.29 0.9

RS2088361 -0.61 0.7 -0.198 -0.05 0.7 -5.041 -0.29 0.9

RS748142 -0.61 0.7 -0.198 -0.05 0.7 -5.041 -0.29 0.9

RS502819 -0.61 0.7 -0.198 -0.05 0.7 -5.041 -0.29 0.9

RS1722710 -0.61 0.7 -0.198 -0.05 0.7 -5.041 -0.29 0.9

RS7543402 -0.61 0.7 -0.198 -0.05 0.7 -5.041 -0.29 0.9

RS10888608 -0.61 0.7 -0.198 -0.05 0.7 -5.041 -0.29 0.9

RS320004 -0.61 0.7 -0.198 -0.05 0.7 -5.041 -0.29 0.9

RS1473455 -0.61 0.7 -0.198 -0.05 0.7 -5.041 -0.29 0.9

RS17105150 -0.61 0.7 -0.198 -0.05 0.7 -5.041 -0.29 0.9

RS1543562 -0.61 0.7 -0.198 -0.05 0.7 -5.041 -0.29 0.9

RS3902720 -0.61 0.7 -0.198 -0.05 0.7 -5.041 -0.29 0.9

RS4926844 -0.61 0.7 -0.198 -0.05 0.7 -5.041 -0.29 0.9

RS3001644 -0.61 0.7 -0.198 -0.05 0.7 -5.041 -0.29 0.9

RS17106219 -0.61 0.7 -0.198 -0.05 0.7 -5.041 -0.29 0.9

RS11205768 -0.61 0.7 -0.198 -0.05 0.7 -5.041 -0.29 0.9

RS12118519 -0.61 0.7 -0.198 -0.05 0.7 -5.041 -0.29 0.9

RS11205801 -0.61 0.7 -0.198 -0.05 0.7 -5.041 -0.29 0.9

RS7541084 -0.61 0.7 -0.198 -0.05 0.7 -5.042 -0.29 0.9

RS7534689 -0.61 0.7 -0.198 -0.05 0.7 -5.042 -0.29 0.9

RS9633423 -0.61 0.7 -0.198 -0.05 0.7 -5.041 -0.29 0.9

RS681865 -0.61 0.7 -0.198 -0.05 0.7 -5.041 -0.29 0.9

RS1672909 -0.61 0.7 -0.198 -0.05 0.7 -5.041 -0.29 0.9

RS3766775 -0.61 0.7 -0.198 -0.05 0.7 -5.041 -0.29 0.9

RS3766792 -0.61 0.7 -0.198 -0.05 0.7 -5.041 -0.29 0.9

RS6695567 -0.61 0.7 -0.198 -0.05 0.7 -5.041 -0.29 0.9

RS7535922 -0.61 0.7 -0.198 -0.05 0.7 -5.041 -0.29 0.9

RS6588474 -0.61 0.7 -0.198 -0.05 0.7 -5.041 -0.29 0.9

RS6686055 -0.61 0.7 -0.198 -0.05 0.7 -5.041 -0.29 0.9

RS12023145 -0.61 0.7 -0.198 -0.05 0.7 -5.041 -0.29 0.9

RS2280511 -0.61 0.7 -0.198 -0.05 0.7 -5.041 -0.29 0.9

RS11206169 -0.61 0.7 -0.198 -0.05 0.7 -5.041 -0.29 0.9

RS4575030 -0.61 0.7 -0.198 -0.05 0.7 -5.041 -0.29 0.9

RS2141079 -0.61 0.7 -0.198 -0.05 0.7 -5.041 -0.29 0.9

RS6588492 -0.61 0.7 -0.198 -0.05 0.7 -5.041 -0.29 0.9

RS12063422 -0.61 0.7 -0.198 -0.05 0.7 -5.041 -0.29 0.9

RS946448 -0.61 0.7 -0.198 -0.05 0.7 -5.041 -0.29 0.9

RS687050 -0.61 0.7 -0.198 -0.05 0.7 -5.041 -0.29 0.9

RS603901 -0.61 0.7 -0.198 -0.05 0.7 -5.041 -0.29 0.9

RS6694281 -0.61 0.7 -0.198 -0.05 0.7 -5.041 -0.29 0.9

RS12021758 -0.61 0.7 -0.198 -0.05 0.7 -5.041 -0.29 0.9

RS213496 -0.61 0.7 -0.198 -0.05 0.7 -5.041 -0.29 0.9

RS300269 -0.70 0.8 -0.198 -0.06 0.7 -9.999 -0.70 1.0

RS1172779 -0.71 0.8 -0.198 -0.06 0.7 -9.999 -0.72 1.0

RS10736390 -0.73 0.8 -0.198 -0.06 0.7 -9.999 -0.78 1.0

RS2078273 -0.75 0.8 -0.198 -0.06 0.7 -9.999 -0.81 1.0

RS744748 -0.76 0.8 -0.198 -0.06 0.7 -9.999 -0.84 1.0

RS6671173 -0.76 0.8 -0.198 -0.06 0.7 -9.999 -0.84 1.0

RS2764822 -0.74 0.8 -0.198 -0.06 0.7 -9.999 -0.83 1.0

RS628667 -0.74 0.8 -0.198 -0.06 0.7 -9.999 -0.83 1.0

RS638944 -0.73 0.8 -0.198 -0.06 0.7 -9.999 -0.83 1.0

RS7549203 -0.73 0.8 -0.198 -0.06 0.7 -9.999 -0.82 1.0

RS12016659 -0.73 0.8 -0.198 -0.06 0.7 -9.999 -0.82 1.0

RS4927186 -0.73 0.8 -0.198 -0.06 0.7 -9.999 -0.82 1.0

RS17111584 -0.73 0.8 -0.198 -0.06 0.7 -9.999 -0.82 1.0

RS6680729 -0.73 0.8 -0.198 -0.06 0.7 -9.999 -0.82 1.0

RS6680609 -0.73 0.8 -0.198 -0.06 0.7 -9.999 -0.82 1.0

RS2015252 -0.73 0.8 -0.198 -0.06 0.7 -9.999 -0.82 1.0

RS10888923 -0.73 0.8 -0.198 -0.06 0.7 -9.999 -0.82 1.0

RS6704127 -0.73 0.8 -0.198 -0.06 0.7 -9.999 -0.82 1.0

RS11577319 -0.73 0.8 -0.198 -0.06 0.7 -9.999 -0.83 1.0

RS4367814 -0.73 0.8 -0.198 -0.06 0.7 -9.999 -0.83 1.0

RS6681658 -0.74 0.8 -0.198 -0.06 0.7 -9.999 -0.83 1.0

RS11206718 -0.76 0.8 -0.198 -0.06 0.7 -9.999 -0.84 1.0

RS4912189 -0.76 0.8 -0.198 -0.06 0.7 -9.999 -0.84 1.0

RS2991265 -0.76 0.8 -0.198 -0.06 0.7 -9.999 -0.84 1.0

RS933001 -0.76 0.8 -0.198 -0.06 0.7 -9.999 -0.84 1.0

RS10749709 -0.76 0.8 -0.198 -0.06 0.7 -9.999 -0.84 1.0

RS857124 -0.76 0.8 -0.198 -0.06 0.7 -9.999 -0.84 1.0

RS1935561 -0.76 0.8 -0.198 -0.06 0.7 -9.999 -0.84 1.0

RS11206973 -0.76 0.8 -0.198 -0.06 0.7 -9.999 -0.84 1.0

RS534080 -0.76 0.8 -0.198 -0.06 0.7 -9.999 -0.84 1.0

RS563921 -0.76 0.8 -0.198 -0.06 0.7 -9.999 -0.84 1.0

RS502350 -0.76 0.8 -0.198 -0.06 0.7 -9.999 -0.84 1.0

RS746937 -0.76 0.8 -0.198 -0.06 0.7 -9.999 -0.84 1.0

RS11207036 -0.76 0.8 -0.198 -0.06 0.7 -9.999 -0.84 1.0

RS4279875 -0.76 0.8 -0.198 -0.06 0.7 -9.999 -0.84 1.0

RS11207039 -0.76 0.8 -0.198 -0.06 0.7 -9.999 -0.84 1.0

RS4568858 -0.76 0.8 -0.198 -0.06 0.7 -9.999 -0.84 1.0

RS1924267 -0.76 0.8 -0.198 -0.06 0.7 -9.999 -0.84 1.0

RS1581762 -0.76 0.8 -0.198 -0.06 0.7 -9.999 -0.84 1.0

RS852749 -0.76 0.8 -0.198 -0.06 0.7 -9.999 -0.84 1.0

RS1884487 -0.76 0.8 -0.198 -0.06 0.7 -9.999 -0.84 1.0

RS7532719 -0.76 0.8 -0.198 -0.06 0.7 -9.999 -0.84 1.0

RS4110981 -0.76 0.8 -0.198 -0.06 0.7 -9.999 -0.84 1.0

RS2764687 -0.76 0.8 -0.198 -0.06 0.7 -9.999 -0.84 1.0

RS515683 -0.76 0.8 -0.198 -0.06 0.7 -9.999 -0.84 1.0

RS639241 -0.76 0.8 -0.198 -0.06 0.7 -9.999 -0.84 1.0

RS4244014 -0.76 0.8 -0.198 -0.06 0.7 -9.999 -0.84 1.0

RS338934 -0.76 0.8 -0.198 -0.06 0.7 -9.999 -0.84 1.0

RS338911 -0.76 0.8 -0.198 -0.06 0.7 -9.999 -0.84 1.0

RS706439 -0.76 0.8 -0.198 -0.06 0.7 -9.999 -0.84 1.0

RS232851 -0.76 0.8 -0.198 -0.06 0.7 -9.999 -0.84 1.0

RS2716107 -0.76 0.8 -0.198 -0.06 0.7 -9.999 -0.84 1.0

RS3949912 -0.76 0.8 -0.198 -0.06 0.7 -9.999 -0.84 1.0

RS2764933 -0.76 0.8 -0.198 -0.06 0.7 -9.999 -0.84 1.0

RS6687397 -0.76 0.8 -0.198 -0.06 0.7 -9.999 -0.84 1.0

RS4912389 -0.76 0.8 -0.198 -0.06 0.7 -9.999 -0.84 1.0

RS331635 -0.76 0.8 -0.198 -0.06 0.7 -9.999 -0.84 1.0

RS6683082 -0.76 0.8 -0.198 -0.06 0.7 -9.999 -0.84 1.0

RS4912396 -0.76 0.8 -0.198 -0.06 0.7 -9.999 -0.84 1.0

RS1188236 -0.76 0.8 -0.198 -0.06 0.7 -9.999 -0.84 1.0

RS6690357 -0.76 0.8 -0.198 -0.06 0.7 -9.999 -0.84 1.0

RS9726956 -0.76 0.8 -0.198 -0.06 0.7 -9.999 -0.84 1.0

RS4915842 -0.76 0.8 -0.198 -0.06 0.7 -9.999 -0.84 1.0

RS640639 -0.76 0.8 -0.198 -0.06 0.7 -9.999 -0.84 1.0

RS6661520 -0.76 0.8 -0.198 -0.06 0.7 -9.999 -0.84 1.0

RS298147 -0.76 0.8 -0.198 -0.06 0.7 -9.999 -0.84 1.0

RS11806181 -0.76 0.8 -0.198 -0.06 0.7 -9.999 -0.84 1.0

RS10493291 -0.76 0.8 -0.198 -0.06 0.7 -9.999 -0.84 1.0

RS6657429 -0.76 0.8 -0.198 -0.06 0.7 -9.999 -0.84 1.0

RS1002005 -0.76 0.8 -0.198 -0.06 0.7 -9.999 -0.84 1.0

RS12062121 -0.76 0.8 -0.198 -0.06 0.7 -9.999 -0.84 1.0

RS12140465 -0.76 0.8 -0.198 -0.06 0.7 -9.999 -0.84 1.0

RS12028122 -0.76 0.8 -0.198 -0.06 0.7 -9.999 -0.84 1.0

RS437021 -0.76 0.8 -0.198 -0.06 0.7 -9.999 -0.84 1.0

RS1414265 -0.76 0.8 -0.198 -0.06 0.7 -9.999 -0.84 1.0

RS2806440 -0.76 0.8 -0.198 -0.06 0.7 -9.999 -0.84 1.0

RS1125777 -0.76 0.8 -0.198 -0.06 0.7 -9.999 -0.84 1.0

RS6587924 -0.76 0.8 -0.198 -0.06 0.7 -9.999 -0.84 1.0

RS6682077 -0.76 0.8 -0.198 -0.06 0.7 -9.999 -0.84 1.0

RS7519218 -0.76 0.8 -0.198 -0.06 0.7 -9.999 -0.84 1.0

RS7530003 -0.76 0.8 -0.198 -0.06 0.7 -9.999 -0.84 1.0

RS12139976 -0.76 0.8 -0.198 -0.06 0.7 -9.999 -0.84 1.0

RS6587943 -0.76 0.8 -0.198 -0.06 0.7 -9.999 -0.84 1.0

RS1332636 -0.76 0.8 -0.198 -0.06 0.7 -9.999 -0.84 1.0

RS4072372 -0.76 0.8 -0.198 -0.06 0.7 -9.999 -0.84 1.0

RS11207909 -0.76 0.8 -0.198 -0.06 0.7 -9.999 -0.84 1.0

RS2666504 -0.76 0.8 -0.198 -0.06 0.7 -9.999 -0.84 1.0

RS12116810 -0.76 0.8 -0.198 -0.06 0.7 -9.999 -0.84 1.0

RS11207944 -0.76 0.8 -0.198 -0.06 0.7 -9.999 -0.84 1.0

RS12062559 -0.51 0.7 -0.198 -0.04 0.7 -9.999 -0.64 1.0

RS12037499 -0.35 0.6 -0.198 -0.03 0.6 -9.999 -0.43 0.9

RS2366210 -0.32 0.6 -0.198 -0.03 0.6 -9.999 -0.37 0.9

RS4915853 -0.14 0.6 -0.198 -0.01 0.6 -0.185 -0.01 0.6

RS11208047 -0.11 0.5 -0.198 -0.01 0.6 -0.138 -0.00 0.5

RS12134779 -0.10 0.5 -0.198 -0.01 0.6 -0.119 -0.00 0.5

RS1333731 -0.10 0.5 -0.198 -0.01 0.6 -0.115 -0.00 0.5

RS6683386 -0.10 0.5 -0.198 -0.01 0.6 -0.113 -0.00 0.5

RS6696216 -0.10 0.5 -0.198 -0.01 0.6 -0.113 -0.00 0.5

RS4915629 -0.10 0.5 -0.198 -0.01 0.6 -0.113 -0.00 0.5

RS12021818 -0.10 0.5 -0.198 -0.01 0.6 -0.112 -0.00 0.5

RS1874930 -0.10 0.5 -0.198 -0.01 0.6 -0.112 -0.00 0.5

RS6588015 -0.09 0.5 -0.198 -0.01 0.6 -0.111 -0.00 0.5

RS4915891 -0.09 0.5 -0.198 -0.01 0.6 -0.111 -0.00 0.5

RS12119056 -0.09 0.5 -0.198 -0.01 0.6 -0.111 -0.00 0.5

RS2269266 -0.09 0.5 -0.198 -0.01 0.6 -0.110 -0.00 0.5

RS2269248 -0.09 0.5 -0.198 -0.01 0.6 -0.110 -0.00 0.5

RS2269236 -0.09 0.5 -0.198 -0.01 0.6 -0.110 -0.00 0.5

RS4915644 -0.09 0.5 -0.198 -0.01 0.6 -0.110 -0.00 0.5

RS640407 -0.09 0.5 -0.198 -0.01 0.6 -0.110 -0.00 0.5

RS6660751 -0.09 0.5 -0.198 -0.01 0.6 -0.110 -0.00 0.5

RS2806541 -0.09 0.5 -0.198 -0.01 0.6 -0.110 -0.00 0.5

RS7527017 -0.09 0.5 -0.198 -0.01 0.6 -0.110 -0.00 0.5

RS7545208 -0.09 0.5 -0.198 -0.01 0.6 -0.110 -0.00 0.5

RS11208420 -0.09 0.5 -0.198 -0.01 0.6 -0.110 -0.00 0.5

RS7523530 -0.09 0.5 -0.198 -0.01 0.6 -0.110 -0.00 0.5

RS11208446 -0.09 0.5 -0.198 -0.01 0.6 -0.110 -0.00 0.5

RS305535 -0.09 0.5 -0.198 -0.01 0.6 -0.110 -0.00 0.5

RS7517824 -0.09 0.5 -0.198 -0.01 0.6 -0.110 -0.00 0.5

RS12565594 -0.09 0.5 -0.198 -0.01 0.6 -0.110 -0.00 0.5

RS2375473 -0.09 0.5 -0.198 -0.01 0.6 -0.110 -0.00 0.5

RS10465850 -0.09 0.5 -0.198 -0.01 0.6 -0.110 -0.00 0.5

RS1413885 -0.09 0.5 -0.198 -0.01 0.6 -0.110 -0.00 0.5

RS10889548 -0.09 0.5 -0.198 -0.01 0.6 -0.110 -0.00 0.5

RS17127673 -0.09 0.5 -0.198 -0.01 0.6 -0.110 -0.00 0.5

RS4384209 -0.09 0.5 -0.198 -0.01 0.6 -0.110 -0.00 0.5

RS1316871 -0.09 0.5 -0.198 -0.01 0.6 -0.110 -0.00 0.5

RS650440 -0.09 0.5 -0.198 -0.01 0.6 -0.110 -0.00 0.5

RS2503174 -0.09 0.5 -0.198 -0.01 0.6 -0.110 -0.00 0.5

RS12043611 -0.09 0.5 -0.198 -0.01 0.6 -0.110 -0.00 0.5

RS910693 -0.09 0.5 -0.198 -0.01 0.6 -0.110 -0.00 0.5

RS17432003 -0.09 0.5 -0.198 -0.01 0.6 -0.110 -0.00 0.5

RS2121052 -0.09 0.5 -0.198 -0.01 0.6 -0.110 -0.00 0.5

RS6689755 -0.09 0.5 -0.198 -0.01 0.6 -0.110 -0.00 0.5

RS11208949 -0.08 0.5 -0.198 -0.01 0.6 -0.092 -0.00 0.5

RS549148 -0.05 0.5 -0.198 -0.00 0.6 -0.051 -0.00 0.5

RS2755259 0.02 0.5 1.309 0.01 0.4 0.023 0.00 0.5

RS7530511 0.08 0.5 1.309 0.04 0.3 0.070 0.00 0.5

RS12119179 0.08 0.5 1.309 0.04 0.3 0.070 0.00 0.5

RS10489626 0.08 0.5 1.309 0.04 0.3 0.070 0.00 0.5

RS11209063 0.08 0.5 1.309 0.04 0.3 0.070 0.00 0.5

RS12116575 0.08 0.5 1.309 0.04 0.3 0.070 0.00 0.5

RS9285766 0.08 0.5 1.309 0.04 0.3 0.070 0.00 0.5

RS11209101 0.08 0.5 1.309 0.04 0.3 0.070 0.00 0.5

RS12085093 0.08 0.5 1.309 0.04 0.3 0.070 0.00 0.5

RS787533 0.08 0.5 1.309 0.04 0.3 0.070 0.00 0.5

RS344934 0.08 0.5 1.309 0.04 0.3 0.070 0.00 0.5

RS1276303 0.08 0.5 1.309 0.04 0.3 0.070 0.00 0.5

RS7552577 0.08 0.5 1.309 0.04 0.3 0.070 0.00 0.5

RS6662448 0.08 0.5 1.309 0.04 0.3 0.070 0.00 0.5

RS7550608 -0.13 0.6 -0.198 -0.01 0.6 -0.171 -0.00 0.6

RS12134024 -0.37 0.6 -0.198 -0.03 0.6 -0.931 -0.05 0.7

RS12046920 -0.37 0.6 -0.198 -0.03 0.6 -0.931 -0.05 0.7

RS2820500 -0.37 0.6 -0.198 -0.03 0.6 -0.931 -0.05 0.7

RS3923451 -0.37 0.6 -0.198 -0.03 0.6 -0.931 -0.05 0.7

RS9436816 -0.37 0.6 -0.198 -0.03 0.6 -0.931 -0.05 0.7

RS3125929 -0.37 0.6 -0.198 -0.03 0.6 -0.931 -0.05 0.7

RS6588326 -0.37 0.6 -0.198 -0.03 0.6 -0.931 -0.05 0.7

RS12123555 -0.37 0.6 -0.198 -0.03 0.6 -0.931 -0.05 0.7

RS11209454 -0.37 0.6 -0.198 -0.03 0.6 -0.931 -0.05 0.7

RS1120087 -0.37 0.6 -0.198 -0.03 0.6 -0.931 -0.05 0.7

RS6424550 -0.37 0.6 -0.198 -0.03 0.6 -0.931 -0.05 0.7

RS6662257 -0.37 0.6 -0.198 -0.03 0.6 -0.931 -0.05 0.7

RS12030275 -0.37 0.6 -0.198 -0.03 0.6 -0.931 -0.05 0.7

RS12562375 -0.37 0.6 -0.198 -0.03 0.6 -0.931 -0.05 0.7

RS8179319 -0.37 0.6 -0.198 -0.03 0.6 -0.931 -0.05 0.7

RS527148 -0.37 0.6 -0.198 -0.03 0.6 -0.931 -0.05 0.7

RS682717 -0.27 0.6 -0.198 -0.02 0.6 -0.812 -0.03 0.7

RS1327460 1.19 0.12 1.309 0.41 0.09 0.573 0.20 0.2

RS875727 1.37 0.09 1.309 0.45 0.08 0.589 0.23 0.2

RS602383 1.44 0.07 1.309 0.46 0.07 0.594 0.24 0.15

RS726764 1.64 0.05 1.309 0.50 0.06 0.608 0.27 0.13

RS4272572 1.64 0.05 1.309 0.50 0.06 0.608 0.27 0.13

RS357237 1.64 0.05 1.309 0.50 0.06 0.608 0.27 0.13

RS10889924 1.64 0.05 1.309 0.50 0.06 0.608 0.27 0.13

RS12023418 1.64 0.05 1.309 0.50 0.06 0.608 0.27 0.13

RS3101340 1.64 0.05 1.309 0.50 0.06 0.608 0.27 0.13

RS3101338 1.64 0.05 1.309 0.50 0.06 0.608 0.27 0.13

RS1405062 1.64 0.05 1.309 0.50 0.06 0.608 0.27 0.13

RS6684341 1.64 0.05 1.309 0.50 0.06 0.609 0.27 0.13

RS4617355 1.64 0.05 1.309 0.50 0.06 0.609 0.27 0.13

RS1497840 1.64 0.05 1.309 0.50 0.06 0.609 0.27 0.13

RS6680989 1.64 0.05 1.309 0.50 0.06 0.609 0.27 0.13

RS2014651 1.64 0.05 1.309 0.50 0.06 0.609 0.27 0.13

RS17552114 1.64 0.05 1.309 0.50 0.06 0.609 0.27 0.13

RS11162738 1.64 0.05 1.309 0.50 0.06 0.608 0.27 0.13

RS1249794 1.64 0.05 1.309 0.50 0.06 0.608 0.27 0.13

RS11164042 1.64 0.05 1.309 0.50 0.06 0.608 0.27 0.13

RS1770878 1.64 0.05 1.309 0.50 0.06 0.608 0.27 0.13

RS1251467 1.64 0.05 1.309 0.50 0.06 0.608 0.27 0.13

RS915403 1.64 0.05 1.309 0.50 0.06 0.608 0.27 0.13

RS4245647 1.64 0.05 1.309 0.50 0.06 0.608 0.27 0.13

RS4949624 1.64 0.05 1.309 0.50 0.06 0.608 0.27 0.13

RS12131613 1.64 0.05 1.309 0.50 0.06 0.608 0.27 0.13

RS10493591 1.64 0.05 1.309 0.50 0.06 0.608 0.27 0.13

RS12239582 1.64 0.05 1.309 0.50 0.06 0.608 0.27 0.13

RS7544496 1.42 0.08 1.309 0.46 0.07 0.573 0.22 0.2

RS423480 1.28 0.10 1.309 0.43 0.08 0.542 0.19 0.2

RS1184626 0.98 0.2 1.309 0.36 0.10 0.456 0.12 0.2

RS507630 0.98 0.2 1.309 0.36 0.10 0.456 0.12 0.2

RS1566250 0.98 0.2 1.309 0.36 0.10 0.456 0.12 0.2

RS7534641 0.98 0.2 1.309 0.36 0.10 0.456 0.12 0.2

RS1766285 0.98 0.2 1.309 0.36 0.10 0.456 0.12 0.2

RS199710 0.98 0.2 1.309 0.36 0.10 0.456 0.12 0.2

RS11806344 0.98 0.2 1.309 0.36 0.10 0.456 0.12 0.2

RS10493606 0.98 0.2 1.309 0.36 0.10 0.456 0.12 0.2

RS6658302 0.98 0.2 1.309 0.36 0.10 0.456 0.12 0.2

RS2647503 0.98 0.2 1.309 0.36 0.10 0.456 0.12 0.2

RS2803136 0.98 0.2 1.309 0.36 0.10 0.456 0.12 0.2

RS1780050 0.98 0.2 1.309 0.36 0.10 0.456 0.12 0.2

RS7555504 0.98 0.2 1.309 0.36 0.10 0.456 0.12 0.2

RS10873998 0.98 0.2 1.309 0.36 0.10 0.456 0.12 0.2

RS1937019 0.98 0.2 1.309 0.36 0.10 0.456 0.12 0.2

RS7527803 0.98 0.2 1.309 0.36 0.10 0.456 0.12 0.2

RS6691975 0.98 0.2 1.309 0.36 0.10 0.456 0.12 0.2

RS10158555 0.98 0.2 1.309 0.36 0.10 0.456 0.12 0.2

RS6424661 0.98 0.2 1.309 0.36 0.10 0.456 0.12 0.2

RS11162772 0.98 0.2 1.309 0.36 0.10 0.456 0.12 0.2

RS6681417 0.98 0.2 1.309 0.36 0.10 0.456 0.12 0.2

RS989580 0.98 0.2 1.309 0.36 0.10 0.456 0.12 0.2

RS3927739 0.98 0.2 1.309 0.36 0.10 0.456 0.12 0.2

RS10874098 0.98 0.2 1.309 0.36 0.10 0.456 0.12 0.2

RS2184613 0.98 0.2 1.309 0.36 0.10 0.456 0.12 0.2

RS12407709 0.98 0.2 1.309 0.36 0.10 0.456 0.12 0.2

RS1687846 0.98 0.2 1.309 0.36 0.10 0.456 0.12 0.2

RS1937791 0.98 0.2 1.309 0.36 0.10 0.456 0.12 0.2

RS6680065 0.98 0.2 1.309 0.36 0.10 0.456 0.12 0.2

RS2152856 0.98 0.2 1.309 0.36 0.10 0.456 0.12 0.2

RS11163106 0.98 0.2 1.309 0.36 0.10 0.456 0.12 0.2

RS17105538 0.98 0.2 1.309 0.36 0.10 0.456 0.12 0.2

RS6659760 0.98 0.2 1.309 0.36 0.10 0.456 0.12 0.2

RS12041774 0.98 0.2 1.309 0.36 0.10 0.456 0.12 0.2

RS1768400 0.98 0.2 1.309 0.36 0.10 0.456 0.12 0.2

RS12565210 0.98 0.2 1.309 0.36 0.10 0.456 0.12 0.2

RS7534535 0.98 0.2 1.309 0.36 0.10 0.456 0.12 0.2

RS4526653 0.98 0.2 1.309 0.36 0.10 0.456 0.12 0.2

RS11163281 0.98 0.2 1.309 0.36 0.10 0.456 0.12 0.2

RS10874255 0.98 0.2 1.309 0.36 0.10 0.456 0.12 0.2

RS3748770 0.98 0.2 1.309 0.36 0.10 0.456 0.12 0.2

RS12145656 0.98 0.2 1.309 0.36 0.10 0.456 0.12 0.2

RS962249 0.98 0.2 1.309 0.36 0.10 0.456 0.12 0.2

RS12083586 0.98 0.2 1.309 0.36 0.10 0.456 0.12 0.2

RS10493712 0.98 0.2 1.309 0.36 0.10 0.456 0.12 0.2

RS1281603 0.98 0.2 1.309 0.36 0.10 0.456 0.12 0.2

RS1766629 0.98 0.2 1.309 0.36 0.10 0.456 0.12 0.2

RS1433761 0.98 0.2 1.309 0.36 0.10 0.456 0.12 0.2

RS2184006 0.98 0.2 1.309 0.36 0.10 0.456 0.12 0.2

RS6682943 0.98 0.2 1.309 0.36 0.10 0.456 0.12 0.2

RS12033542 0.98 0.2 1.309 0.36 0.10 0.456 0.12 0.2

RS12408136 0.98 0.2 1.309 0.36 0.10 0.456 0.12 0.2

RS12082801 0.98 0.2 1.309 0.36 0.10 0.456 0.12 0.2

RS17503362 0.98 0.2 1.309 0.36 0.10 0.456 0.12 0.2

RS977889 0.98 0.2 1.309 0.36 0.10 0.456 0.12 0.2

RS4539154 0.98 0.2 1.309 0.36 0.10 0.456 0.12 0.2

RS9787335 0.98 0.2 1.309 0.36 0.10 0.456 0.12 0.2

RS6691243 0.98 0.2 1.309 0.36 0.10 0.456 0.12 0.2

RS12083999 0.98 0.2 1.309 0.36 0.10 0.456 0.12 0.2

RS12057561 0.98 0.2 1.309 0.36 0.10 0.456 0.12 0.2

RS11163939 0.98 0.2 1.309 0.36 0.10 0.456 0.12 0.2

RS4907103 0.98 0.2 1.309 0.36 0.10 0.456 0.12 0.2

RS4907105 0.98 0.2 1.309 0.36 0.10 0.456 0.12 0.2

RS1536120 0.98 0.2 1.309 0.36 0.10 0.456 0.12 0.2

RS504504 0.98 0.2 1.309 0.36 0.10 0.456 0.12 0.2

RS1770691 0.98 0.2 1.309 0.36 0.10 0.456 0.12 0.2

RS709767 0.98 0.2 1.309 0.36 0.10 0.456 0.12 0.2

RS962409 0.98 0.2 1.309 0.36 0.10 0.456 0.12 0.2

RS233100 0.98 0.2 1.309 0.36 0.10 0.456 0.12 0.2

RS1403956 0.98 0.2 1.309 0.36 0.10 0.456 0.12 0.2

RS512298 0.98 0.2 1.309 0.36 0.10 0.456 0.12 0.2

RS11161637 0.98 0.2 1.309 0.36 0.10 0.456 0.12 0.2

RS11161686 0.98 0.2 1.309 0.36 0.10 0.456 0.12 0.2

RS1904947 0.98 0.2 1.309 0.36 0.10 0.456 0.12 0.2

RS585708 0.98 0.2 1.309 0.36 0.10 0.456 0.12 0.2

RS1108395 0.98 0.2 1.309 0.36 0.10 0.456 0.12 0.2

RS1321690 0.98 0.2 1.309 0.36 0.10 0.456 0.12 0.2

RS13376522 0.98 0.2 1.309 0.36 0.10 0.456 0.12 0.2

RS6679741 0.98 0.2 1.309 0.36 0.10 0.456 0.12 0.2

RS719015 0.98 0.2 1.309 0.36 0.10 0.456 0.12 0.2

RS4655941 0.98 0.2 1.309 0.36 0.10 0.456 0.12 0.2

RS4233323 0.98 0.2 1.309 0.36 0.10 0.456 0.12 0.2

RS3766022 0.98 0.2 1.309 0.36 0.10 0.456 0.12 0.2

RS6683030 0.98 0.2 1.309 0.36 0.10 0.456 0.12 0.2

RS923545 0.98 0.2 1.309 0.36 0.10 0.456 0.12 0.2

RS954653 0.98 0.2 1.309 0.36 0.10 0.456 0.12 0.2

RS2503264 0.98 0.2 1.309 0.36 0.10 0.456 0.12 0.2

RS10489944 0.98 0.2 1.309 0.36 0.10 0.456 0.12 0.2

RS1336578 0.98 0.2 1.309 0.36 0.10 0.456 0.12 0.2

RS4656031 0.98 0.2 1.309 0.36 0.10 0.456 0.12 0.2

RS10801678 0.98 0.2 1.309 0.36 0.10 0.456 0.12 0.2

RS7538497 0.98 0.2 1.309 0.36 0.10 0.456 0.12 0.2

RS7555039 0.98 0.2 1.309 0.36 0.10 0.456 0.12 0.2

RS6692501 0.98 0.2 1.309 0.36 0.10 0.456 0.12 0.2

RS9428043 0.98 0.2 1.309 0.36 0.10 0.456 0.12 0.2

RS6685584 0.98 0.2 1.309 0.36 0.10 0.456 0.12 0.2

RS10754283 0.98 0.2 1.309 0.36 0.10 0.456 0.12 0.2

RS7542239 0.98 0.2 1.309 0.36 0.10 0.456 0.12 0.2

RS7542860 0.98 0.2 1.309 0.36 0.10 0.456 0.12 0.2

RS2703999 0.98 0.2 1.309 0.36 0.10 0.456 0.12 0.2

RS1329644 0.98 0.2 1.309 0.36 0.10 0.456 0.12 0.2

RS12038587 0.98 0.2 1.309 0.36 0.10 0.456 0.12 0.2

RS4388690 0.98 0.2 1.309 0.36 0.10 0.456 0.12 0.2

RS12036175 0.98 0.2 1.309 0.36 0.10 0.456 0.12 0.2

RS4446947 0.98 0.2 1.309 0.36 0.10 0.456 0.12 0.2

RS12141568 0.98 0.2 1.309 0.36 0.10 0.456 0.12 0.2

RS7546709 0.98 0.2 1.309 0.36 0.10 0.456 0.12 0.2

RS527402 0.98 0.2 1.309 0.36 0.10 0.456 0.12 0.2

RS2797293 0.98 0.2 1.309 0.36 0.10 0.456 0.12 0.2

RS12354259 0.98 0.2 1.309 0.36 0.10 0.456 0.12 0.2

RS10493842 0.98 0.2 1.309 0.36 0.10 0.456 0.12 0.2

RS1997155 0.98 0.2 1.309 0.36 0.10 0.456 0.12 0.2

RS1358156 0.98 0.2 1.309 0.36 0.10 0.456 0.12 0.2

RS164989 0.98 0.2 1.309 0.36 0.10 0.456 0.12 0.2

RS240504 0.98 0.2 1.309 0.36 0.10 0.456 0.12 0.2

RS347028 0.98 0.2 1.309 0.36 0.10 0.456 0.12 0.2

RS969618 0.98 0.2 1.309 0.36 0.10 0.456 0.12 0.2

RS574364 0.98 0.2 1.309 0.36 0.10 0.456 0.12 0.2

RS12058802 0.98 0.2 1.309 0.36 0.10 0.456 0.12 0.2

RS4658244 0.98 0.2 1.309 0.36 0.10 0.456 0.12 0.2

RS4459159 0.98 0.2 1.309 0.36 0.10 0.456 0.12 0.2

RS6660484 0.98 0.2 1.309 0.36 0.10 0.456 0.12 0.2

RS12076549 0.98 0.2 1.309 0.36 0.10 0.456 0.12 0.2

RS17131552 0.98 0.2 1.309 0.36 0.10 0.456 0.12 0.2

RS6680614 0.98 0.2 1.309 0.36 0.10 0.456 0.12 0.2

RS1073001 0.98 0.2 1.309 0.36 0.10 0.456 0.12 0.2

RS10782946 0.98 0.2 1.309 0.36 0.10 0.456 0.12 0.2

RS1678778 0.98 0.2 1.309 0.36 0.10 0.456 0.12 0.2

RS236316 0.98 0.2 1.309 0.36 0.10 0.456 0.12 0.2

RS11164974 0.98 0.2 1.309 0.36 0.10 0.456 0.12 0.2

RS4526623 0.98 0.2 1.309 0.36 0.10 0.456 0.12 0.2

RS945067 0.98 0.2 1.309 0.36 0.10 0.456 0.12 0.2

RS4847196 0.98 0.2 1.309 0.36 0.10 0.456 0.12 0.2

RS3789439 0.98 0.2 1.309 0.36 0.10 0.456 0.12 0.2

RS3789688 0.98 0.2 1.309 0.36 0.10 0.456 0.12 0.2

RS10399785 0.98 0.2 1.309 0.36 0.10 0.456 0.12 0.2

RS1749541 0.98 0.2 1.309 0.36 0.10 0.456 0.12 0.2

RS1772895 0.98 0.2 1.309 0.36 0.10 0.456 0.12 0.2

RS11165191 0.98 0.2 1.309 0.36 0.10 0.456 0.12 0.2

RS17111996 0.98 0.2 1.309 0.36 0.10 0.456 0.12 0.2

RS841361 0.98 0.2 1.309 0.36 0.10 0.456 0.12 0.2

RS3933012 0.98 0.2 1.309 0.36 0.10 0.456 0.12 0.2

RS10493875 0.98 0.2 1.309 0.36 0.10 0.456 0.12 0.2

RS1146267 0.98 0.2 1.309 0.36 0.10 0.456 0.12 0.2

RS3849302 0.98 0.2 1.309 0.36 0.10 0.456 0.12 0.2

RS698963 0.98 0.2 1.309 0.36 0.10 0.456 0.12 0.2

RS9432593 0.98 0.2 1.309 0.36 0.10 0.456 0.12 0.2

RS6665763 0.98 0.2 1.309 0.36 0.10 0.456 0.12 0.2

RS1023330 0.98 0.2 1.309 0.36 0.10 0.456 0.12 0.2

RS2039614 0.98 0.2 1.309 0.36 0.10 0.456 0.12 0.2

RS1160318 0.98 0.2 1.309 0.36 0.10 0.456 0.12 0.2

RS4949960 0.98 0.2 1.309 0.36 0.10 0.456 0.12 0.2

RS7524217 0.98 0.2 1.309 0.36 0.10 0.456 0.12 0.2

RS10874924 0.98 0.2 1.309 0.36 0.10 0.456 0.12 0.2

RS10489562 0.98 0.2 1.309 0.36 0.10 0.456 0.12 0.2

RS3108719 0.98 0.2 1.309 0.36 0.10 0.456 0.12 0.2

RS7554994 0.98 0.2 1.309 0.36 0.10 0.456 0.12 0.2

RS1931262 0.98 0.2 1.309 0.36 0.10 0.456 0.12 0.2

RS6593693 0.98 0.2 1.309 0.36 0.10 0.456 0.12 0.2

RS321270 0.98 0.2 1.309 0.36 0.10 0.456 0.12 0.2

RS161120 0.98 0.2 1.309 0.36 0.10 0.456 0.12 0.2

RS1222069 0.98 0.2 1.309 0.36 0.10 0.456 0.12 0.2

RS12132961 0.98 0.2 1.309 0.36 0.10 0.456 0.12 0.2

RS2256459 0.98 0.2 1.309 0.36 0.10 0.456 0.12 0.2

RS1925833 0.98 0.2 1.309 0.36 0.10 0.456 0.12 0.2

RS6693873 0.98 0.2 1.309 0.36 0.10 0.456 0.12 0.2

RS17470762 0.98 0.2 1.309 0.36 0.10 0.456 0.12 0.2

RS291593 0.98 0.2 1.309 0.36 0.10 0.456 0.12 0.2

RS6593634 0.98 0.2 1.309 0.36 0.10 0.456 0.12 0.2

RS592609 0.98 0.2 1.309 0.36 0.10 0.456 0.12 0.2

RS10875062 0.98 0.2 1.309 0.36 0.10 0.456 0.12 0.2

RS2811183 0.98 0.2 1.309 0.36 0.10 0.456 0.12 0.2

RS2811174 0.98 0.2 1.309 0.36 0.10 0.456 0.12 0.2

RS7533902 0.98 0.2 1.309 0.36 0.10 0.456 0.12 0.2

RS9662365 0.98 0.2 1.309 0.36 0.10 0.456 0.12 0.2

RS4950107 0.98 0.2 1.309 0.36 0.10 0.456 0.12 0.2

RS11165996 0.98 0.2 1.309 0.36 0.10 0.456 0.12 0.2

RS728656 0.98 0.2 1.309 0.36 0.10 0.456 0.12 0.2

RS10157812 0.98 0.2 1.309 0.36 0.10 0.456 0.12 0.2

RS12123631 0.98 0.2 1.309 0.36 0.10 0.456 0.12 0.2

RS4908025 0.98 0.2 1.309 0.36 0.10 0.456 0.12 0.2

RS11166113 0.98 0.2 1.309 0.36 0.10 0.456 0.12 0.2

RS6577380 0.98 0.2 1.309 0.36 0.10 0.456 0.12 0.2

RS12060602 0.98 0.2 1.309 0.36 0.10 0.456 0.12 0.2

RS834963 0.98 0.2 1.309 0.36 0.10 0.456 0.12 0.2

RS17405754 0.98 0.2 1.309 0.36 0.10 0.456 0.12 0.2

RS7531793 0.98 0.2 1.309 0.36 0.10 0.456 0.12 0.2

RS17409324 0.98 0.2 1.309 0.36 0.10 0.456 0.12 0.2

RS3806239 0.98 0.2 1.309 0.36 0.10 0.456 0.12 0.2

RS599215 0.98 0.2 1.309 0.36 0.10 0.456 0.12 0.2

RS10875304 0.98 0.2 1.309 0.36 0.10 0.456 0.12 0.2

RS2809789 0.98 0.2 1.309 0.36 0.10 0.456 0.12 0.2

RS4908093 0.98 0.2 1.309 0.36 0.10 0.456 0.12 0.2

RS7543296 0.98 0.2 1.309 0.36 0.10 0.456 0.12 0.2

RS1983967 0.98 0.2 1.309 0.36 0.10 0.456 0.12 0.2

RS17123757 0.98 0.2 1.309 0.36 0.10 0.456 0.12 0.2

RS1571498 0.98 0.2 1.309 0.36 0.10 0.456 0.12 0.2

RS12408201 0.98 0.2 1.309 0.36 0.10 0.456 0.12 0.2

RS1033634 0.98 0.2 1.309 0.36 0.10 0.456 0.12 0.2

RS11164177 0.98 0.2 1.309 0.36 0.10 0.456 0.12 0.2

RS406138 0.98 0.2 1.309 0.36 0.10 0.456 0.12 0.2

RS4908170 0.98 0.2 1.309 0.36 0.10 0.456 0.12 0.2

RS11164278 0.98 0.2 1.309 0.36 0.10 0.456 0.12 0.2

RS10874506 0.98 0.2 1.309 0.36 0.10 0.456 0.12 0.2

RS4907955 0.98 0.2 1.309 0.36 0.10 0.456 0.12 0.2

RS6661054 0.98 0.2 1.309 0.36 0.10 0.456 0.12 0.2

RS9324336 0.98 0.2 1.309 0.36 0.10 0.456 0.12 0.2

RS11800637 0.98 0.2 1.309 0.36 0.10 0.456 0.12 0.2

RS17125667 0.98 0.2 1.309 0.36 0.10 0.456 0.12 0.2

RS2000150 0.98 0.2 1.309 0.36 0.10 0.456 0.12 0.2

RS6657751 0.98 0.2 1.309 0.36 0.10 0.456 0.12 0.2

RS6684718 0.98 0.2 1.309 0.36 0.10 0.456 0.12 0.2

RS2889234 0.98 0.2 1.309 0.36 0.10 0.456 0.12 0.2

RS10158366 0.98 0.2 1.309 0.36 0.10 0.456 0.12 0.2

RS12402460 0.98 0.2 1.309 0.36 0.10 0.456 0.12 0.2

RS11164585 0.98 0.2 1.309 0.36 0.10 0.456 0.12 0.2

RS10782904 0.98 0.2 1.309 0.36 0.10 0.456 0.12 0.2

RS6692914 0.98 0.2 1.309 0.36 0.10 0.456 0.12 0.2

RS4907988 0.98 0.2 1.309 0.36 0.10 0.456 0.12 0.2

RS11185384 0.98 0.2 1.309 0.36 0.10 0.456 0.12 0.2

RS17020664 0.98 0.2 1.309 0.36 0.10 0.456 0.12 0.2

RS6700638 0.98 0.2 1.309 0.36 0.10 0.456 0.12 0.2

RS2594664 0.98 0.2 1.309 0.36 0.10 0.456 0.12 0.2

RS12041707 0.98 0.2 1.309 0.36 0.10 0.456 0.12 0.2

RS373489 0.98 0.2 1.309 0.36 0.10 0.456 0.12 0.2

RS2034388 0.98 0.2 1.309 0.36 0.10 0.456 0.12 0.2

RS2167300 0.98 0.2 1.309 0.36 0.10 0.456 0.12 0.2

RS12030832 0.98 0.2 1.309 0.36 0.10 0.456 0.12 0.2

RS11184462 0.98 0.2 1.309 0.36 0.10 0.456 0.12 0.2

RS1524108 0.98 0.2 1.309 0.36 0.10 0.456 0.12 0.2

RS6421299 0.98 0.2 1.309 0.36 0.10 0.456 0.12 0.2

RS11184726 0.98 0.2 1.309 0.36 0.10 0.456 0.12 0.2

RS12404525 0.98 0.2 1.309 0.36 0.10 0.456 0.12 0.2

RS11184747 1.09 0.14 1.309 0.38 0.09 0.492 0.14 0.2

RS11184867 1.64 0.05 1.309 0.50 0.06 0.608 0.27 0.13

RS726669 1.64 0.05 1.309 0.50 0.06 0.608 0.27 0.13

RS9435334 1.64 0.05 1.309 0.50 0.06 0.608 0.27 0.13

RS7513206 1.64 0.05 1.309 0.50 0.06 0.608 0.27 0.13

RS6583016 1.64 0.05 1.309 0.50 0.06 0.608 0.27 0.13

RS7411507 1.64 0.05 1.309 0.50 0.06 0.608 0.27 0.13

RS12066815 1.64 0.05 1.309 0.50 0.06 0.608 0.27 0.13

RS6681988 1.64 0.05 1.309 0.50 0.06 0.608 0.27 0.13

RS597332 1.64 0.05 1.309 0.50 0.06 0.608 0.27 0.13

RS10785826 1.64 0.05 1.309 0.50 0.06 0.608 0.27 0.13

RS8676 1.64 0.05 1.309 0.50 0.06 0.608 0.27 0.13

RS10494083 1.64 0.05 1.309 0.50 0.06 0.608 0.27 0.13

RS7531042 1.64 0.05 1.309 0.50 0.06 0.608 0.27 0.13

RS11185274 1.64 0.05 1.309 0.50 0.06 0.608 0.27 0.13

RS829004 1.64 0.05 1.309 0.50 0.06 0.608 0.27 0.13

RS594397 1.64 0.05 1.309 0.50 0.06 0.608 0.27 0.13

RS6583070 1.64 0.05 1.309 0.50 0.06 0.608 0.27 0.13

RS2798184 1.64 0.05 1.309 0.50 0.06 0.608 0.27 0.13

RS10494096 1.64 0.05 1.309 0.50 0.06 0.608 0.27 0.13

RS839549 1.64 0.05 1.309 0.50 0.06 0.608 0.27 0.13

RS11102813 1.64 0.05 1.309 0.50 0.06 0.608 0.27 0.13

RS586254 1.64 0.05 1.309 0.50 0.06 0.608 0.27 0.13

RS653635 1.64 0.05 1.309 0.50 0.06 0.608 0.27 0.13

RS2281894 1.64 0.05 1.309 0.50 0.06 0.608 0.27 0.13

RS655246 1.64 0.05 1.309 0.50 0.06 0.608 0.27 0.13

RS3738772 1.64 0.05 1.309 0.50 0.06 0.608 0.27 0.13

RS12023963 1.64 0.05 1.309 0.50 0.06 0.608 0.27 0.13

RS4970768 1.64 0.05 1.309 0.50 0.06 0.608 0.27 0.13

RS12046107 1.64 0.05 1.309 0.50 0.06 0.608 0.27 0.13

RS681991 1.64 0.05 1.309 0.50 0.06 0.608 0.27 0.13

RS655315 1.64 0.05 1.309 0.50 0.06 0.608 0.27 0.13

RS1854962 1.64 0.05 1.309 0.50 0.06 0.608 0.27 0.13

RS4970777 1.64 0.05 1.309 0.50 0.06 0.608 0.27 0.13

RS525566 1.64 0.05 1.309 0.50 0.06 0.608 0.27 0.13

RS1021880 1.64 0.05 1.309 0.50 0.06 0.608 0.27 0.13

RS453577 1.64 0.05 1.309 0.50 0.06 0.608 0.27 0.13

RS333966 1.64 0.05 1.309 0.50 0.06 0.608 0.27 0.13

RS7365459 1.64 0.05 1.309 0.50 0.06 0.608 0.27 0.13

RS11102043 1.64 0.05 1.309 0.50 0.06 0.608 0.27 0.13

RS3754443 1.64 0.05 1.309 0.50 0.06 0.608 0.27 0.13

RS4839136 1.64 0.05 1.309 0.50 0.06 0.608 0.27 0.13

RS666493 1.64 0.05 1.309 0.50 0.06 0.608 0.27 0.13

RS3768460 1.64 0.05 1.309 0.50 0.06 0.608 0.27 0.13

RS7534042 1.64 0.05 1.309 0.50 0.06 0.608 0.27 0.13

RS12118420 1.64 0.05 1.309 0.50 0.06 0.608 0.27 0.13

RS4839417 1.64 0.05 1.309 0.50 0.06 0.608 0.27 0.13

RS17025859 1.64 0.05 1.309 0.50 0.06 0.608 0.27 0.13

RS815324 1.64 0.05 1.309 0.50 0.06 0.608 0.27 0.13

RS12748140 1.64 0.05 1.309 0.50 0.06 0.608 0.27 0.13

RS12062889 1.64 0.05 1.309 0.50 0.06 0.608 0.27 0.13

RS1058184 1.64 0.05 1.309 0.50 0.06 0.608 0.27 0.13

RS4839557 1.64 0.05 1.309 0.50 0.06 0.608 0.27 0.13

RS343769 1.64 0.05 1.309 0.50 0.06 0.608 0.27 0.13

RS1712674 1.64 0.05 1.309 0.50 0.06 0.608 0.27 0.13

RS1418426 1.64 0.05 1.309 0.50 0.06 0.608 0.27 0.13

RS694180 1.64 0.05 1.309 0.50 0.06 0.608 0.27 0.13

RS7514853 1.64 0.05 1.309 0.50 0.06 0.608 0.27 0.13

RS10776724 1.64 0.05 1.309 0.50 0.06 0.608 0.27 0.13

RS11102257 1.64 0.05 1.309 0.50 0.06 0.608 0.27 0.13

RS1544224 1.64 0.05 1.309 0.50 0.06 0.608 0.27 0.13

RS2789535 1.64 0.05 1.309 0.50 0.06 0.608 0.27 0.13

RS2364815 1.64 0.05 1.309 0.50 0.06 0.608 0.27 0.13

RS2786991 1.64 0.05 1.309 0.50 0.06 0.608 0.27 0.13

RS11102314 1.64 0.05 1.309 0.50 0.06 0.608 0.27 0.13

RS17028976 1.64 0.05 1.309 0.50 0.06 0.608 0.27 0.13

RS6682872 1.64 0.05 1.309 0.50 0.06 0.608 0.27 0.13

RS2000265 1.64 0.05 1.309 0.50 0.06 0.608 0.27 0.13

RS11578917 1.64 0.05 1.309 0.50 0.06 0.608 0.27 0.13

RS11102362 1.64 0.05 1.309 0.50 0.06 0.608 0.27 0.13

RS716954 1.64 0.05 1.309 0.50 0.06 0.608 0.27 0.13

RS10857922 1.64 0.05 1.309 0.50 0.06 0.608 0.27 0.13

RS385768 1.64 0.05 1.309 0.50 0.06 0.608 0.27 0.13

RS269101 1.64 0.05 1.309 0.50 0.06 0.608 0.27 0.13

RS10857934 1.64 0.05 1.309 0.50 0.06 0.608 0.27 0.13

RS2998359 1.64 0.05 1.309 0.50 0.06 0.608 0.27 0.13

RS11102441 1.64 0.05 1.309 0.50 0.06 0.608 0.27 0.13

RS1936050 1.64 0.05 1.309 0.50 0.06 0.608 0.27 0.13

RS7554916 1.64 0.05 1.309 0.50 0.06 0.608 0.27 0.13

RS11102524 1.64 0.05 1.309 0.50 0.06 0.608 0.27 0.13

RS6537756 1.64 0.05 1.309 0.50 0.06 0.608 0.27 0.13

RS6537765 1.64 0.05 1.309 0.50 0.06 0.608 0.27 0.13

RS773573 1.64 0.05 1.309 0.50 0.06 0.608 0.27 0.13

RS773564 1.64 0.05 1.309 0.50 0.06 0.608 0.27 0.13

RS4839306 1.64 0.05 1.309 0.50 0.06 0.608 0.27 0.13

RS1342650 1.64 0.05 1.309 0.50 0.06 0.608 0.27 0.13

RS1936930 1.64 0.05 1.309 0.50 0.06 0.608 0.27 0.13

RS7554019 1.64 0.05 1.309 0.50 0.06 0.608 0.27 0.13

RS6669008 1.64 0.05 1.309 0.50 0.06 0.608 0.27 0.13

RS3006988 1.64 0.05 1.309 0.50 0.06 0.608 0.27 0.13

RS12402202 1.64 0.05 1.309 0.50 0.06 0.608 0.27 0.13

RS11102724 1.64 0.05 1.309 0.50 0.06 0.608 0.27 0.13

RS12027243 1.64 0.05 1.309 0.50 0.06 0.608 0.27 0.13

RS901137 1.64 0.05 1.309 0.50 0.06 0.608 0.27 0.13

RS2629824 1.64 0.05 1.309 0.50 0.06 0.608 0.27 0.13

RS1385665 1.64 0.05 1.309 0.50 0.06 0.608 0.27 0.13

RS11589933 1.64 0.05 1.309 0.50 0.06 0.608 0.27 0.13

RS6664266 1.64 0.05 1.309 0.50 0.06 0.608 0.27 0.13

RS516610 1.64 0.05 1.309 0.50 0.06 0.608 0.27 0.13

RS11102807 1.64 0.05 1.309 0.50 0.06 0.608 0.27 0.13

RS10858046 1.64 0.05 1.309 0.50 0.06 0.608 0.27 0.13

RS6681671 1.64 0.05 1.309 0.50 0.06 0.608 0.27 0.13

RS7542599 1.64 0.05 1.309 0.50 0.06 0.608 0.27 0.13

RS11102901 1.64 0.05 1.309 0.50 0.06 0.608 0.27 0.13

RS6330 1.45 0.07 1.309 0.46 0.07 0.578 0.23 0.2

RS11102933 0.98 0.2 1.309 0.36 0.10 0.456 0.12 0.2

RS7552722 0.98 0.2 1.309 0.36 0.10 0.456 0.12 0.2

RS553146 0.98 0.2 1.309 0.36 0.10 0.456 0.12 0.2

RS4839038 0.98 0.2 1.309 0.36 0.10 0.456 0.12 0.2

RS3811003 0.98 0.2 1.309 0.36 0.10 0.456 0.12 0.2

RS9428087 0.98 0.2 1.309 0.36 0.10 0.456 0.12 0.2

RS4453038 0.98 0.2 1.309 0.36 0.10 0.456 0.12 0.2

RS4548442 0.98 0.2 1.309 0.36 0.10 0.456 0.12 0.2

RS2096210 0.98 0.2 1.309 0.36 0.10 0.456 0.12 0.2

RS10802159 0.98 0.2 1.309 0.36 0.10 0.456 0.12 0.2

RS2208365 0.98 0.2 1.309 0.36 0.10 0.456 0.12 0.2

RS2184483 0.98 0.2 1.309 0.36 0.10 0.456 0.12 0.2

RS6428666 0.98 0.2 1.309 0.36 0.10 0.456 0.12 0.2

RS2478766 0.98 0.2 1.309 0.36 0.10 0.456 0.12 0.2

RS3736907 0.98 0.2 1.309 0.36 0.10 0.456 0.12 0.2

RS2274253 0.98 0.2 1.309 0.36 0.10 0.456 0.12 0.2

RS1289663 0.98 0.2 1.309 0.36 0.10 0.456 0.12 0.2

RS6428686 0.98 0.2 1.309 0.36 0.10 0.456 0.12 0.2

RS1935780 0.98 0.2 1.309 0.36 0.10 0.456 0.12 0.2

RS12135026 0.98 0.2 1.309 0.36 0.10 0.456 0.12 0.2

RS1707113 0.98 0.2 1.309 0.36 0.10 0.456 0.12 0.2

RS10801963 0.98 0.2 1.309 0.36 0.10 0.456 0.12 0.2

RS826403 0.98 0.2 1.309 0.36 0.10 0.456 0.12 0.2

RS1891556 0.98 0.2 1.309 0.36 0.10 0.456 0.12 0.2

RS4659339 0.98 0.2 1.309 0.36 0.10 0.456 0.12 0.2

RS17257729 0.98 0.2 1.309 0.36 0.10 0.456 0.12 0.2

RS1146349 0.98 0.2 1.309 0.36 0.10 0.456 0.12 0.2

RS1146320 0.98 0.2 1.309 0.36 0.10 0.456 0.12 0.2

RS1228985 0.98 0.2 1.309 0.36 0.10 0.456 0.12 0.2

RS2755140 0.98 0.2 1.309 0.36 0.10 0.456 0.12 0.2

RS10923694 0.98 0.2 1.309 0.36 0.10 0.456 0.12 0.2

RS10923754 0.98 0.2 1.309 0.36 0.10 0.456 0.12 0.2

RS6678854 0.98 0.2 1.309 0.36 0.10 0.456 0.12 0.2

RS3820032 0.98 0.2 1.309 0.36 0.10 0.456 0.12 0.2

RS10802113 0.98 0.2 1.309 0.36 0.10 0.456 0.12 0.2

RS1923025 0.98 0.2 1.309 0.36 0.10 0.456 0.12 0.2

RS620741 0.98 0.2 1.309 0.36 0.10 0.456 0.12 0.2

RS12030925 0.98 0.2 1.309 0.36 0.10 0.456 0.12 0.2

RS543703 0.98 0.2 1.309 0.36 0.10 0.456 0.12 0.2

RS699778 0.98 0.2 1.309 0.36 0.10 0.456 0.12 0.2

RS10923916 0.98 0.2 1.309 0.36 0.10 0.456 0.12 0.2

RS12060624 0.98 0.2 1.309 0.36 0.10 0.456 0.12 0.2

RS10802138 0.98 0.2 1.309 0.36 0.10 0.456 0.12 0.2

RS10218795 0.98 0.2 1.309 0.36 0.10 0.456 0.12 0.2

RS11587821 0.98 0.2 1.309 0.36 0.10 0.455 0.12 0.2

RS1298954 0.98 0.2 1.309 0.36 0.10 0.455 0.12 0.2

RS647596 0.98 0.2 1.309 0.36 0.10 0.455 0.12 0.2

RS4950328 0.97 0.2 1.309 0.36 0.10 0.455 0.12 0.2

RS3766506 0.97 0.2 1.309 0.36 0.10 0.454 0.12 0.2

RS4950343 0.97 0.2 1.309 0.36 0.10 0.454 0.12 0.2

RS1342709 0.97 0.2 1.309 0.36 0.10 0.454 0.12 0.2

RS6664767 0.97 0.2 1.309 0.36 0.10 0.454 0.12 0.2

RS7541950 0.96 0.2 1.309 0.35 0.10 0.452 0.11 0.2

RS4950524 0.85 0.2 1.309 0.33 0.11 0.433 0.10 0.3

RS1763457 0.84 0.2 1.309 0.32 0.11 0.431 0.10 0.3

RS7545679 0.23 0.4 1.309 0.11 0.2 0.204 0.01 0.4

RS6685305 0.23 0.4 1.309 0.11 0.2 0.202 0.01 0.4

RS3738320 -0.12 0.5 -0.198 -0.01 0.6 -0.154 -0.00 0.6

RS2274127 -0.12 0.5 -0.198 -0.01 0.6 -0.154 -0.00 0.6

RS9659073 -0.12 0.5 -0.198 -0.01 0.6 -0.154 -0.00 0.6

RS11204675 -0.12 0.5 -0.198 -0.01 0.6 -0.154 -0.00 0.6

RS12045807 -0.12 0.5 -0.198 -0.01 0.6 -0.154 -0.00 0.6

RS11204754 -0.12 0.5 -0.198 -0.01 0.6 -0.154 -0.00 0.6

RS3738480 -0.12 0.5 -0.198 -0.01 0.6 -0.154 -0.00 0.6

RS12741361 -0.12 0.5 -0.198 -0.01 0.6 -0.154 -0.00 0.6

RS11204828 -0.12 0.5 -0.198 -0.01 0.6 -0.154 -0.00 0.6

RS6673116 -0.12 0.5 -0.198 -0.01 0.6 -0.154 -0.00 0.6

RS1891592 -0.12 0.5 -0.198 -0.01 0.6 -0.154 -0.00 0.6

RS1196350 -0.12 0.5 -0.198 -0.01 0.6 -0.154 -0.00 0.6

RS17646946 -0.12 0.5 -0.198 -0.01 0.6 -0.154 -0.00 0.6

RS1131471 -0.12 0.5 -0.198 -0.01 0.6 -0.154 -0.00 0.6

RS1552991 -0.12 0.5 -0.198 -0.01 0.6 -0.154 -0.00 0.6

RS4845741 -0.12 0.5 -0.198 -0.01 0.6 -0.154 -0.00 0.6

RS6587673 -0.12 0.5 -0.198 -0.01 0.6 -0.154 -0.00 0.6

RS6689049 -0.12 0.5 -0.198 -0.01 0.6 -0.154 -0.00 0.6

RS310103 -0.12 0.5 -0.198 -0.01 0.6 -0.154 -0.00 0.6

RS381115 -0.17 0.6 -0.198 -0.01 0.6 -0.245 -0.01 0.6

RS428913 -0.18 0.6 -0.198 -0.01 0.6 -0.249 -0.01 0.6

RS1360100 -0.24 0.6 -0.198 -0.02 0.6 -0.409 -0.02 0.6

RS873233 -0.27 0.6 -0.198 -0.02 0.6 -0.496 -0.02 0.6

RS2987760 -0.27 0.6 -0.198 -0.02 0.6 -0.496 -0.02 0.6

RS3006475 -0.27 0.6 -0.198 -0.02 0.6 -0.496 -0.02 0.6

RS3006430 -0.27 0.6 -0.198 -0.02 0.6 -0.496 -0.02 0.6

RS11488669 -0.27 0.6 -0.198 -0.02 0.6 -0.496 -0.02 0.6

RS3818632 -0.27 0.6 -0.198 -0.02 0.6 -0.497 -0.02 0.6

RS6427671 -0.27 0.6 -0.198 -0.02 0.6 -0.497 -0.02 0.6

RS3001365 -0.10 0.5 -0.198 -0.01 0.6 -0.125 -0.00 0.5

RS2988721 0.04 0.5 1.309 0.02 0.4 0.042 0.00 0.5

RS17654071 0.06 0.5 1.309 0.03 0.3 0.058 0.00 0.5

RS7553796 0.08 0.5 1.309 0.04 0.3 0.070 0.00 0.5

RS1876304 0.08 0.5 1.309 0.04 0.3 0.070 0.00 0.5

RS7534678 0.08 0.5 1.309 0.04 0.3 0.070 0.00 0.5

RS10494301 0.08 0.5 1.309 0.04 0.3 0.070 0.00 0.5

RS10908431 0.08 0.5 1.309 0.04 0.3 0.070 0.00 0.5

RS7513331 0.08 0.5 1.309 0.04 0.3 0.070 0.00 0.5

RS11264262 0.08 0.5 1.309 0.04 0.3 0.070 0.00 0.5

RS12029542 0.08 0.5 1.309 0.04 0.3 0.070 0.00 0.5

RS1218578 0.08 0.5 1.309 0.04 0.3 0.070 0.00 0.5

RS1218565 0.08 0.5 1.309 0.04 0.3 0.070 0.00 0.5

RS11264341 0.10 0.5 1.309 0.05 0.3 0.089 0.00 0.5

RS12034326 0.11 0.5 1.309 0.06 0.3 0.095 0.00 0.5

RS7555253 0.39 0.3 1.309 0.18 0.2 0.274 0.03 0.4

RS2248273 0.49 0.3 1.309 0.21 0.2 0.316 0.04 0.3

RS872120 0.57 0.3 1.309 0.24 0.15 0.345 0.05 0.3

RS2296373 0.60 0.3 1.309 0.25 0.14 0.355 0.06 0.3

RS6679425 0.73 0.2 1.309 0.29 0.12 0.392 0.07 0.3

RS3748570 0.86 0.2 1.309 0.33 0.11 0.423 0.10 0.3

RS12140437 0.86 0.2 1.309 0.33 0.11 0.423 0.10 0.3

RS6427327 0.86 0.2 1.309 0.33 0.11 0.423 0.10 0.3

RS967072 0.86 0.2 1.309 0.33 0.11 0.423 0.10 0.3

RS822572 0.86 0.2 1.309 0.33 0.11 0.423 0.10 0.3

RS946577 0.86 0.2 1.309 0.33 0.11 0.423 0.10 0.3

RS883328 0.86 0.2 1.309 0.33 0.11 0.423 0.10 0.3

RS1591785 0.86 0.2 1.309 0.33 0.11 0.423 0.10 0.3

RS12130232 0.86 0.2 1.309 0.33 0.11 0.423 0.10 0.3

RS7535100 0.86 0.2 1.309 0.33 0.11 0.423 0.10 0.3

RS4246535 0.86 0.2 1.309 0.33 0.11 0.423 0.10 0.3

RS16838537 0.86 0.2 1.309 0.33 0.11 0.423 0.10 0.3

RS4246538 0.86 0.2 1.309 0.33 0.11 0.423 0.10 0.3

RS11264737 0.86 0.2 1.309 0.33 0.11 0.423 0.10 0.3

RS6680515 0.86 0.2 1.309 0.33 0.11 0.423 0.10 0.3

RS7524550 0.86 0.2 1.309 0.33 0.11 0.423 0.10 0.3

RS7514610 0.86 0.2 1.309 0.33 0.11 0.423 0.10 0.3

RS1925036 0.86 0.2 1.309 0.33 0.11 0.423 0.10 0.3

RS6696582 0.86 0.2 1.309 0.33 0.11 0.423 0.10 0.3

RS7527735 0.86 0.2 1.309 0.33 0.11 0.423 0.10 0.3

RS1886389 0.86 0.2 1.309 0.33 0.11 0.423 0.10 0.3

RS12071018 0.86 0.2 1.309 0.33 0.11 0.423 0.10 0.3

RS10489828 0.86 0.2 1.309 0.33 0.11 0.423 0.10 0.3

RS4656284 0.86 0.2 1.309 0.33 0.11 0.423 0.10 0.3

RS2479861 0.86 0.2 1.309 0.33 0.11 0.423 0.10 0.3

RS1864348 0.86 0.2 1.309 0.33 0.11 0.423 0.10 0.3

RS857838 0.86 0.2 1.309 0.33 0.11 0.423 0.10 0.3

RS857788 0.86 0.2 1.309 0.33 0.11 0.423 0.10 0.3

RS1633292 0.86 0.2 1.309 0.33 0.11 0.423 0.10 0.3

RS1615480 0.86 0.2 1.309 0.33 0.11 0.423 0.10 0.3

RS1361567 0.86 0.2 1.309 0.33 0.11 0.423 0.10 0.3

RS2814779 0.86 0.2 1.309 0.33 0.11 0.423 0.10 0.3

RS2281300 0.86 0.2 1.309 0.33 0.11 0.423 0.10 0.3

RS2247584 0.86 0.2 1.309 0.33 0.11 0.423 0.10 0.3

RS7532282 0.86 0.2 1.309 0.33 0.11 0.423 0.10 0.3

RS6702567 0.86 0.2 1.309 0.33 0.11 0.423 0.10 0.3

RS1037143 0.86 0.2 1.309 0.33 0.11 0.423 0.10 0.3

RS1446975 0.86 0.2 1.309 0.33 0.11 0.423 0.10 0.3

RS2794520 0.86 0.2 1.309 0.33 0.11 0.423 0.10 0.3

RS3122012 0.86 0.2 1.309 0.33 0.11 0.423 0.10 0.3

RS7519478 0.86 0.2 1.309 0.33 0.11 0.423 0.10 0.3

RS4630124 0.86 0.2 1.309 0.33 0.11 0.423 0.10 0.3

RS2501346 0.86 0.2 1.309 0.33 0.11 0.423 0.10 0.3

RS11265289 0.86 0.2 1.309 0.33 0.11 0.423 0.10 0.3

RS12566514 0.86 0.2 1.309 0.33 0.11 0.423 0.10 0.3

RS2249707 0.86 0.2 1.309 0.33 0.11 0.423 0.10 0.3

RS2185214 0.86 0.2 1.309 0.33 0.11 0.423 0.10 0.3

RS2211107 0.86 0.2 1.309 0.33 0.11 0.423 0.10 0.3

RS1321650 0.86 0.2 1.309 0.33 0.11 0.423 0.10 0.3

RS12753283 0.86 0.2 1.309 0.33 0.11 0.423 0.10 0.3

RS6693877 0.86 0.2 1.309 0.33 0.11 0.423 0.10 0.3

RS7522166 0.86 0.2 1.309 0.33 0.11 0.423 0.10 0.3

RS10494343 0.86 0.2 1.309 0.33 0.11 0.423 0.10 0.3

RS2369611 0.86 0.2 1.309 0.33 0.11 0.423 0.10 0.3

RS1503859 0.86 0.2 1.309 0.33 0.11 0.423 0.10 0.3

RS10489639 0.86 0.2 1.309 0.33 0.11 0.423 0.10 0.3

RS571841 0.86 0.2 1.309 0.33 0.11 0.423 0.10 0.3

RS4656978 0.86 0.2 1.309 0.33 0.11 0.423 0.10 0.3

RS2502805 0.86 0.2 1.309 0.33 0.11 0.423 0.10 0.3

RS6698806 0.86 0.2 1.309 0.33 0.11 0.423 0.10 0.3

RS1801274 0.86 0.2 1.309 0.33 0.11 0.423 0.10 0.3

RS10918029 0.86 0.2 1.309 0.33 0.11 0.423 0.10 0.3

RS7539281 0.86 0.2 1.309 0.33 0.11 0.423 0.10 0.3

RS10157390 0.86 0.2 1.309 0.33 0.11 0.423 0.10 0.3

RS347300 0.86 0.2 1.309 0.33 0.11 0.423 0.10 0.3

RS10753789 0.86 0.2 1.309 0.33 0.11 0.423 0.10 0.3

RS449908 0.86 0.2 1.309 0.33 0.11 0.423 0.10 0.3

RS6681981 0.86 0.2 1.309 0.33 0.11 0.423 0.10 0.3

RS1047802 0.86 0.2 1.309 0.33 0.11 0.423 0.10 0.3

RS6673387 0.86 0.2 1.309 0.33 0.11 0.423 0.10 0.3

RS7544972 0.86 0.2 1.309 0.33 0.11 0.423 0.10 0.3

RS2805025 0.86 0.2 1.309 0.33 0.11 0.423 0.10 0.3

RS2684874 0.86 0.2 1.309 0.33 0.11 0.423 0.10 0.3

RS6679069 0.86 0.2 1.309 0.33 0.11 0.423 0.10 0.3

RS2032049 0.86 0.2 1.309 0.33 0.11 0.423 0.10 0.3

RS2343508 0.86 0.2 1.309 0.33 0.11 0.423 0.10 0.3

RS2661339 0.86 0.2 1.309 0.33 0.11 0.423 0.10 0.3

RS951437 0.86 0.2 1.309 0.33 0.11 0.423 0.10 0.3

RS2841959 0.86 0.2 1.309 0.33 0.11 0.423 0.10 0.3

RS1876451 0.86 0.2 1.309 0.33 0.11 0.423 0.10 0.3

RS1509019 0.86 0.2 1.309 0.33 0.11 0.423 0.10 0.3

RS11582919 0.86 0.2 1.309 0.33 0.11 0.423 0.10 0.3

RS1022058 0.86 0.2 1.309 0.33 0.11 0.423 0.10 0.3

RS12735706 0.86 0.2 1.309 0.33 0.11 0.423 0.10 0.3

RS4657284 0.86 0.2 1.309 0.33 0.11 0.423 0.10 0.3

RS2211183 0.86 0.2 1.309 0.33 0.11 0.423 0.10 0.3

RS10799925 0.86 0.2 1.309 0.33 0.11 0.423 0.10 0.3

RS10917787 0.86 0.2 1.309 0.33 0.11 0.423 0.10 0.3

RS2345965 0.86 0.2 1.309 0.33 0.11 0.423 0.10 0.3

RS884518 0.86 0.2 1.309 0.33 0.11 0.423 0.10 0.3

RS12129997 0.86 0.2 1.309 0.33 0.11 0.423 0.10 0.3

RS1122279 0.86 0.2 1.309 0.33 0.11 0.423 0.10 0.3

RS1289586 0.77 0.2 1.309 0.30 0.12 0.402 0.08 0.3

RS7533006 0.64 0.3 1.309 0.27 0.13 0.369 0.06 0.3

RS12043375 -0.08 0.5 -0.198 -0.01 0.6 -0.106 -0.00 0.5

RS10800024 -0.21 0.6 -0.198 -0.02 0.6 -0.341 -0.01 0.6

RS946253 -0.23 0.6 -0.198 -0.02 0.6 -0.401 -0.02 0.6

RS6426874 -0.32 0.6 -0.198 -0.03 0.6 -0.664 -0.04 0.7

RS10800043 -0.32 0.6 -0.198 -0.03 0.6 -0.664 -0.04 0.7

RS2171692 -0.32 0.6 -0.198 -0.03 0.6 -0.664 -0.04 0.7

RS2789444 -0.32 0.6 -0.198 -0.03 0.6 -0.664 -0.04 0.7

RS7521521 -0.32 0.6 -0.198 -0.03 0.6 -0.664 -0.04 0.7

RS3767371 -0.32 0.6 -0.198 -0.03 0.6 -0.664 -0.04 0.7

RS10800055 -0.32 0.6 -0.198 -0.03 0.6 -0.664 -0.04 0.7

RS1419067 -0.32 0.6 -0.198 -0.03 0.6 -0.664 -0.04 0.7

RS2348247 -0.32 0.6 -0.198 -0.03 0.6 -0.664 -0.04 0.7

RS7519089 -0.32 0.6 -0.198 -0.03 0.6 -0.664 -0.04 0.7

RS11809911 -0.32 0.6 -0.198 -0.03 0.6 -0.664 -0.04 0.7

RS1974080 -0.32 0.6 -0.198 -0.03 0.6 -0.664 -0.04 0.7

RS12057296 -0.32 0.6 -0.198 -0.03 0.6 -0.664 -0.04 0.7

RS3767333 -0.32 0.6 -0.198 -0.03 0.6 -0.664 -0.04 0.7

RS2134095 -0.32 0.6 -0.198 -0.03 0.6 -0.664 -0.04 0.7

RS10918196 -0.32 0.6 -0.198 -0.03 0.6 -0.664 -0.04 0.7

RS16847083 -0.32 0.6 -0.198 -0.03 0.6 -0.664 -0.04 0.7

RS1337444 -0.32 0.6 -0.198 -0.03 0.6 -0.664 -0.04 0.7

RS8133 -0.32 0.6 -0.198 -0.03 0.6 -0.664 -0.04 0.7

RS1143659 -0.32 0.6 -0.198 -0.03 0.6 -0.664 -0.04 0.7

RS10918276 -0.32 0.6 -0.198 -0.03 0.6 -0.664 -0.04 0.7

RS4535982 -0.32 0.6 -0.198 -0.03 0.6 -0.664 -0.04 0.7

RS6703280 -0.32 0.6 -0.198 -0.03 0.6 -0.664 -0.04 0.7

RS4657493 -0.32 0.6 -0.198 -0.03 0.6 -0.664 -0.04 0.7

RS10800173 -0.32 0.6 -0.198 -0.03 0.6 -0.664 -0.04 0.7

RS4074017 -0.32 0.6 -0.198 -0.03 0.6 -0.664 -0.04 0.7

RS3856211 -0.32 0.6 -0.198 -0.03 0.6 -0.664 -0.04 0.7

RS4074897 -0.32 0.6 -0.198 -0.03 0.6 -0.664 -0.04 0.7

RS4282794 -0.32 0.6 -0.198 -0.03 0.6 -0.664 -0.04 0.7

RS7544608 -0.32 0.6 -0.198 -0.03 0.6 -0.664 -0.04 0.7

RS530690 -0.32 0.6 -0.198 -0.03 0.6 -0.664 -0.04 0.7

RS3845541 -0.32 0.6 -0.198 -0.03 0.6 -0.664 -0.04 0.7

RS2064067 -0.32 0.6 -0.198 -0.03 0.6 -0.664 -0.04 0.7

RS11736 -0.32 0.6 -0.198 -0.03 0.6 -0.664 -0.04 0.7

RS2281962 -0.32 0.6 -0.198 -0.03 0.6 -0.664 -0.04 0.7

RS12122365 -0.32 0.6 -0.198 -0.03 0.6 -0.664 -0.04 0.7

RS7540969 -0.32 0.6 -0.198 -0.03 0.6 -0.664 -0.04 0.7

RS869714 -0.32 0.6 -0.198 -0.03 0.6 -0.664 -0.04 0.7

RS748800 -0.32 0.6 -0.198 -0.03 0.6 -0.664 -0.04 0.7

RS7529557 -0.32 0.6 -0.198 -0.03 0.6 -0.664 -0.04 0.7

RS12068411 -0.32 0.6 -0.198 -0.03 0.6 -0.664 -0.04 0.7

RS11584337 -0.32 0.6 -0.198 -0.03 0.6 -0.664 -0.04 0.7

RS2258497 -0.32 0.6 -0.198 -0.03 0.6 -0.664 -0.04 0.7

RS2995093 -0.32 0.6 -0.198 -0.03 0.6 -0.664 -0.04 0.7

RS858550 -0.32 0.6 -0.198 -0.03 0.6 -0.664 -0.04 0.7

RS7539688 -0.32 0.6 -0.198 -0.03 0.6 -0.664 -0.04 0.7

RS1737481 -0.32 0.6 -0.198 -0.03 0.6 -0.664 -0.04 0.7

RS926521 -0.32 0.6 -0.198 -0.03 0.6 -0.664 -0.04 0.7

RS1890130 -0.32 0.6 -0.198 -0.03 0.6 -0.664 -0.04 0.7

RS7605 -0.32 0.6 -0.198 -0.03 0.6 -0.664 -0.04 0.7

RS1476074 -0.32 0.6 -0.198 -0.03 0.6 -0.664 -0.04 0.7

RS204267 -0.32 0.6 -0.198 -0.03 0.6 -0.664 -0.04 0.7

RS3738235 -0.32 0.6 -0.198 -0.03 0.6 -0.664 -0.04 0.7

RS203838 -0.32 0.6 -0.198 -0.03 0.6 -0.664 -0.04 0.7

RS202248 -0.32 0.6 -0.198 -0.03 0.6 -0.664 -0.04 0.7

RS2235207 -0.32 0.6 -0.198 -0.03 0.6 -0.664 -0.04 0.7

RS2268558 -0.32 0.6 -0.198 -0.03 0.6 -0.664 -0.04 0.7

RS2300568 -0.32 0.6 -0.198 -0.03 0.6 -0.665 -0.04 0.7

RS10918901 -0.32 0.6 -0.198 -0.03 0.6 -0.664 -0.04 0.7

RS12094807 -0.32 0.6 -0.198 -0.03 0.6 -0.664 -0.04 0.7

RS1933107 -0.32 0.6 -0.198 -0.03 0.6 -0.664 -0.04 0.7

RS16860907 -0.32 0.6 -0.198 -0.03 0.6 -0.664 -0.04 0.7

RS10800363 -0.32 0.6 -0.198 -0.03 0.6 -0.664 -0.04 0.7

RS16861196 -0.32 0.6 -0.198 -0.03 0.6 -0.664 -0.04 0.7

RS6427139 -0.32 0.6 -0.198 -0.03 0.6 -0.664 -0.04 0.7

RS1933115 -0.32 0.6 -0.198 -0.03 0.6 -0.664 -0.04 0.7

RS2143091 -0.32 0.6 -0.198 -0.03 0.6 -0.664 -0.04 0.7

RS169266 -0.32 0.6 -0.198 -0.03 0.6 -0.664 -0.04 0.7

RS12048750 -0.32 0.6 -0.198 -0.03 0.6 -0.664 -0.04 0.7

RS7544086 -0.32 0.6 -0.198 -0.03 0.6 -0.664 -0.04 0.7

RS10919051 -0.32 0.6 -0.198 -0.03 0.6 -0.664 -0.04 0.7

RS926516 -0.32 0.6 -0.198 -0.03 0.6 -0.664 -0.04 0.7

RS10800431 -0.32 0.6 -0.198 -0.03 0.6 -0.664 -0.04 0.7

RS2040444 -0.32 0.6 -0.198 -0.03 0.6 -0.664 -0.04 0.7

RS1894702 -0.55 0.7 -0.198 -0.04 0.7 -2.954 -0.18 0.8

RS1018828 -0.55 0.7 -0.198 -0.04 0.7 -2.954 -0.18 0.8

RS3917775 -0.55 0.7 -0.198 -0.04 0.7 -2.954 -0.18 0.8

RS10800463 -0.64 0.7 -0.198 -0.05 0.7 -9.999 -0.50 0.9

RS1569475 -0.64 0.7 -0.198 -0.05 0.7 -9.999 -0.50 0.9

RS1051091 -0.74 0.8 -0.198 -0.06 0.7 -9.999 -0.77 1.0

RS3817859 -0.74 0.8 -0.198 -0.06 0.7 -9.999 -0.77 1.0

RS12042109 -0.74 0.8 -0.198 -0.06 0.7 -9.999 -0.77 1.0

RS7518785 -0.74 0.8 -0.198 -0.06 0.7 -9.999 -0.77 1.0

RS4437927 -0.74 0.8 -0.198 -0.06 0.7 -9.999 -0.77 1.0

RS1412788 -0.74 0.8 -0.198 -0.06 0.7 -9.999 -0.77 1.0

RS1333141 -0.74 0.8 -0.198 -0.06 0.7 -9.999 -0.77 1.0

RS520131 -0.74 0.8 -0.198 -0.06 0.7 -9.999 -0.77 1.0

RS6677593 -0.74 0.8 -0.198 -0.06 0.7 -9.999 -0.77 1.0

RS10800546 -0.74 0.8 -0.198 -0.06 0.7 -9.999 -0.77 1.0

RS10489239 -0.74 0.8 -0.198 -0.06 0.7 -9.999 -0.77 1.0

RS9427206 -0.74 0.8 -0.198 -0.06 0.7 -9.999 -0.77 1.0

RS16863898 -0.74 0.8 -0.198 -0.06 0.7 -9.999 -0.77 1.0

RS12076145 -0.74 0.8 -0.198 -0.06 0.7 -9.999 -0.77 1.0

RS2020870 -0.74 0.8 -0.198 -0.06 0.7 -9.999 -0.77 1.0

RS11806072 -0.74 0.8 -0.198 -0.06 0.7 -9.999 -0.77 1.0

RS6670432 -0.74 0.8 -0.198 -0.06 0.7 -9.999 -0.77 1.0

RS2207189 -0.74 0.8 -0.198 -0.06 0.7 -9.999 -0.77 1.0

RS235913 -0.74 0.8 -0.198 -0.06 0.7 -9.999 -0.77 1.0

RS2294719 -0.74 0.8 -0.198 -0.06 0.7 -9.999 -0.77 1.0

RS6425438 -0.74 0.8 -0.198 -0.06 0.7 -9.999 -0.77 1.0

RS2206544 -0.74 0.8 -0.198 -0.06 0.7 -9.999 -0.77 1.0

RS733190 -0.74 0.8 -0.198 -0.06 0.7 -9.999 -0.77 1.0

RS1475230 -0.74 0.8 -0.198 -0.06 0.7 -9.999 -0.77 1.0

RS1023479 -0.74 0.8 -0.198 -0.06 0.7 -9.999 -0.77 1.0

RS1063412 -0.74 0.8 -0.198 -0.06 0.7 -9.999 -0.77 1.0

RS4916263 -0.74 0.8 -0.198 -0.06 0.7 -9.999 -0.77 1.0

RS6424956 -0.74 0.8 -0.198 -0.06 0.7 -9.999 -0.77 1.0

RS4916288 -0.74 0.8 -0.198 -0.06 0.7 -9.999 -0.77 1.0

RS1883478 -0.74 0.8 -0.198 -0.06 0.7 -9.999 -0.77 1.0

RS16845308 -0.74 0.8 -0.198 -0.06 0.7 -9.999 -0.77 1.0

RS2422272 -0.74 0.8 -0.198 -0.06 0.7 -9.999 -0.77 1.0

RS3861948 -0.74 0.8 -0.198 -0.06 0.7 -9.999 -0.77 1.0

RS844644 -0.74 0.8 -0.198 -0.06 0.7 -9.999 -0.77 1.0

RS704840 -0.74 0.8 -0.198 -0.06 0.7 -9.999 -0.77 1.0

RS6685520 -0.74 0.8 -0.198 -0.06 0.7 -9.999 -0.77 1.0

RS10798269 -0.74 0.8 -0.198 -0.06 0.7 -9.999 -0.77 1.0

RS10798282 -0.74 0.8 -0.198 -0.06 0.7 -9.999 -0.77 1.0

RS6681627 -0.74 0.8 -0.198 -0.06 0.7 -9.999 -0.77 1.0

RS7547843 -0.74 0.8 -0.198 -0.06 0.7 -9.999 -0.77 1.0

RS7521981 -0.74 0.8 -0.198 -0.06 0.7 -9.999 -0.77 1.0

RS2012862 -0.74 0.8 -0.198 -0.06 0.7 -9.999 -0.77 1.0

RS2236883 -0.74 0.8 -0.198 -0.06 0.7 -9.999 -0.77 1.0

RS10912968 -0.74 0.8 -0.198 -0.06 0.7 -9.999 -0.77 1.0

RS4652090 -0.74 0.8 -0.198 -0.06 0.7 -9.999 -0.77 1.0

RS2861311 -0.74 0.8 -0.198 -0.06 0.7 -9.999 -0.77 1.0

RS6676780 -0.74 0.8 -0.198 -0.06 0.7 -9.999 -0.77 1.0

RS7515728 -0.74 0.8 -0.198 -0.06 0.7 -9.999 -0.77 1.0

RS4652099 -0.74 0.8 -0.198 -0.06 0.7 -9.999 -0.77 1.0

RS6675876 -0.74 0.8 -0.198 -0.06 0.7 -9.999 -0.77 1.0

RS671000 -0.74 0.8 -0.198 -0.06 0.7 -9.999 -0.77 1.0

RS11810084 -0.74 0.8 -0.198 -0.06 0.7 -9.999 -0.77 1.0

RS352314 -0.74 0.8 -0.198 -0.06 0.7 -9.999 -0.77 1.0

RS1015901 -0.74 0.8 -0.198 -0.06 0.7 -9.999 -0.77 1.0

RS10913267 -0.74 0.8 -0.198 -0.06 0.7 -9.999 -0.77 1.0

RS227992 -0.74 0.8 -0.198 -0.06 0.7 -9.999 -0.77 1.0

RS228008 -0.74 0.8 -0.198 -0.06 0.7 -9.999 -0.77 1.0

RS6678480 -0.74 0.8 -0.198 -0.06 0.7 -9.999 -0.77 1.0

RS12128612 -0.74 0.8 -0.198 -0.06 0.7 -9.999 -0.77 1.0

RS11587000 -0.74 0.8 -0.198 -0.06 0.7 -9.999 -0.77 1.0

RS9425805 -0.74 0.8 -0.198 -0.06 0.7 -9.999 -0.77 1.0

RS1923630 -0.74 0.8 -0.198 -0.06 0.7 -9.999 -0.77 1.0

RS3131323 -0.74 0.8 -0.198 -0.06 0.7 -9.999 -0.77 1.0

RS7411090 -0.74 0.8 -0.198 -0.06 0.7 -9.999 -0.77 1.0

RS3131310 -0.74 0.8 -0.198 -0.06 0.7 -9.999 -0.77 1.0

RS3131313 -0.74 0.8 -0.198 -0.06 0.7 -9.999 -0.77 1.0

RS10798578 -0.74 0.8 -0.198 -0.06 0.7 -9.999 -0.77 1.0

RS522367 -0.74 0.8 -0.198 -0.06 0.7 -9.999 -0.77 1.0

RS12410468 -0.74 0.8 -0.198 -0.06 0.7 -9.999 -0.77 1.0

RS178397 -0.74 0.8 -0.198 -0.06 0.7 -9.999 -0.77 1.0

RS2811301 -0.74 0.8 -0.198 -0.06 0.7 -9.999 -0.77 1.0

RS6425496 -0.74 0.8 -0.198 -0.06 0.7 -9.999 -0.77 1.0

RS16853350 -0.74 0.8 -0.198 -0.06 0.7 -9.999 -0.77 1.0

RS6425507 -0.74 0.8 -0.198 -0.06 0.7 -9.999 -0.77 1.0

RS997419 -0.74 0.8 -0.198 -0.06 0.7 -9.999 -0.77 1.0

RS12725164 -0.74 0.8 -0.198 -0.06 0.7 -9.999 -0.77 1.0

RS10913728 -0.74 0.8 -0.198 -0.06 0.7 -9.999 -0.77 1.0

RS3753527 -0.74 0.8 -0.198 -0.06 0.7 -9.999 -0.77 1.0

RS12725126 -0.74 0.8 -0.198 -0.06 0.7 -9.999 -0.77 1.0

RS1410592 -0.74 0.8 -0.198 -0.06 0.7 -9.999 -0.77 1.0

RS2245195 -0.74 0.8 -0.198 -0.06 0.7 -9.999 -0.77 1.0

RS12756748 -0.74 0.8 -0.198 -0.06 0.7 -9.999 -0.77 1.0

RS2647225 -0.74 0.8 -0.198 -0.06 0.7 -9.999 -0.77 1.0

RS12121845 -0.74 0.8 -0.198 -0.06 0.7 -9.999 -0.77 1.0

RS6704314 -0.74 0.8 -0.198 -0.06 0.7 -9.999 -0.77 1.0

RS7521602 -0.74 0.8 -0.198 -0.06 0.7 -9.999 -0.77 1.0

RS2331545 -0.74 0.8 -0.198 -0.06 0.7 -9.999 -0.77 1.0

RS2331901 -0.74 0.8 -0.198 -0.06 0.7 -9.999 -0.77 1.0

RS3795504 -0.74 0.8 -0.198 -0.06 0.7 -9.999 -0.77 1.0

RS10494536 -0.74 0.8 -0.198 -0.06 0.7 -9.999 -0.77 1.0

RS4651082 -0.74 0.8 -0.198 -0.06 0.7 -9.999 -0.77 1.0

RS10910888 -0.74 0.8 -0.198 -0.06 0.7 -9.999 -0.77 1.0

RS2804682 -0.74 0.8 -0.198 -0.06 0.7 -9.999 -0.77 1.0

RS12139587 -0.74 0.8 -0.198 -0.06 0.7 -9.999 -0.77 1.0

RS617322 -0.74 0.8 -0.198 -0.06 0.7 -9.999 -0.77 1.0

RS681271 -0.74 0.8 -0.198 -0.06 0.7 -9.999 -0.77 1.0

RS6424814 -0.74 0.8 -0.198 -0.06 0.7 -9.999 -0.77 1.0

RS12135959 -0.74 0.8 -0.198 -0.06 0.7 -9.999 -0.77 1.0

RS3767000 -0.74 0.8 -0.198 -0.06 0.7 -9.999 -0.77 1.0

RS695072 -0.74 0.8 -0.198 -0.06 0.7 -9.999 -0.77 1.0

RS1281322 -0.74 0.8 -0.198 -0.06 0.7 -9.999 -0.77 1.0

RS10494545 -0.74 0.8 -0.198 -0.06 0.7 -9.999 -0.77 1.0

RS7524031 -0.74 0.8 -0.198 -0.06 0.7 -9.999 -0.77 1.0

RS2246119 -0.74 0.8 -0.198 -0.06 0.7 -9.999 -0.77 1.0

RS2148026 -0.74 0.8 -0.198 -0.06 0.7 -9.999 -0.77 1.0

RS501085 -0.74 0.8 -0.198 -0.06 0.7 -9.999 -0.77 1.0

RS11072 -0.74 0.8 -0.198 -0.06 0.7 -9.999 -0.77 1.0

RS516567 -0.74 0.8 -0.198 -0.06 0.7 -9.999 -0.77 1.0

RS1287816 -0.74 0.8 -0.198 -0.06 0.7 -9.999 -0.77 1.0

RS587306 -0.74 0.8 -0.198 -0.06 0.7 -9.999 -0.77 1.0

RS508607 -0.74 0.8 -0.198 -0.06 0.7 -9.999 -0.77 1.0

RS6660584 -0.74 0.8 -0.198 -0.06 0.7 -9.999 -0.77 1.0

RS12118059 -0.74 0.8 -0.198 -0.06 0.7 -9.999 -0.77 1.0

RS10797795 -0.74 0.8 -0.198 -0.06 0.7 -9.999 -0.77 1.0

RS4652795 -0.74 0.8 -0.198 -0.06 0.7 -9.999 -0.77 1.0

RS4652802 -0.74 0.8 -0.198 -0.06 0.7 -9.999 -0.77 1.0

RS16861532 -0.74 0.8 -0.198 -0.06 0.7 -9.999 -0.77 1.0

RS2500103 -0.74 0.8 -0.198 -0.06 0.7 -9.999 -0.77 1.0

RS10797921 -0.74 0.8 -0.198 -0.06 0.7 -9.999 -0.77 1.0

RS2986550 -0.74 0.8 -0.198 -0.06 0.7 -9.999 -0.77 1.0

RS10494574 -0.74 0.8 -0.198 -0.06 0.7 -9.999 -0.77 1.0

RS16822379 -0.74 0.8 -0.198 -0.06 0.7 -9.999 -0.77 1.0

RS2840274 -0.74 0.8 -0.198 -0.06 0.7 -9.999 -0.77 1.0

RS4568789 -0.74 0.8 -0.198 -0.06 0.7 -9.999 -0.77 1.0

RS10797957 -0.74 0.8 -0.198 -0.06 0.7 -9.999 -0.77 1.0

RS10489726 -0.74 0.8 -0.198 -0.06 0.7 -9.999 -0.77 1.0

RS1416751 -0.74 0.8 -0.198 -0.06 0.7 -9.999 -0.77 1.0

RS1339563 -0.74 0.8 -0.198 -0.06 0.7 -9.999 -0.77 1.0

RS4651223 -0.74 0.8 -0.198 -0.06 0.7 -9.999 -0.77 1.0

RS861620 -0.74 0.8 -0.198 -0.06 0.7 -9.999 -0.77 1.0

RS170885 -0.74 0.8 -0.198 -0.06 0.7 -9.999 -0.77 1.0

RS1074229 -0.74 0.8 -0.198 -0.06 0.7 -9.999 -0.77 1.0

RS2144291 -0.74 0.8 -0.198 -0.06 0.7 -9.999 -0.77 1.0

RS10798014 -0.74 0.8 -0.198 -0.06 0.7 -9.999 -0.77 1.0

RS1407277 -0.74 0.8 -0.198 -0.06 0.7 -9.999 -0.77 1.0

RS971224 -0.74 0.8 -0.198 -0.06 0.7 -9.999 -0.77 1.0

RS4141459 -0.74 0.8 -0.198 -0.06 0.7 -9.999 -0.77 1.0

RS2208692 -0.74 0.8 -0.198 -0.06 0.7 -9.999 -0.77 1.0

RS857753 -0.74 0.8 -0.198 -0.06 0.7 -9.999 -0.77 1.0

RS2273779 -0.74 0.8 -0.198 -0.06 0.7 -9.999 -0.77 1.0

RS12407957 -0.74 0.8 -0.198 -0.06 0.7 -9.999 -0.77 1.0

RS3131557 -0.74 0.8 -0.198 -0.06 0.7 -9.999 -0.77 1.0

RS10911898 -0.74 0.8 -0.198 -0.06 0.7 -9.999 -0.77 1.0

RS1417585 -0.65 0.7 -0.198 -0.05 0.7 -7.530 -0.38 0.9

RS1538974 -0.65 0.7 -0.198 -0.05 0.7 -7.526 -0.39 0.9

RS4658939 -0.65 0.7 -0.198 -0.05 0.7 -7.519 -0.39 0.9

RS1538977 -0.65 0.7 -0.198 -0.05 0.7 -7.520 -0.39 0.9

RS12046794 -0.65 0.7 -0.198 -0.05 0.7 -7.520 -0.39 0.9

RS12130935 -0.65 0.7 -0.198 -0.05 0.7 -7.521 -0.39 0.9

RS1535529 -0.65 0.7 -0.198 -0.05 0.7 -7.521 -0.39 0.9

RS11122347 -0.65 0.7 -0.198 -0.05 0.7 -7.522 -0.39 0.9

RS1079344 -0.65 0.7 -0.198 -0.05 0.7 -7.522 -0.39 0.9

RS9432040 -0.65 0.7 -0.198 -0.05 0.7 -7.522 -0.39 0.9

RS872625 -0.65 0.7 -0.198 -0.05 0.7 -7.522 -0.39 0.9

RS1888601 -0.65 0.7 -0.198 -0.05 0.7 -7.522 -0.39 0.9

RS10864724 -0.65 0.7 -0.198 -0.05 0.7 -7.522 -0.39 0.9

RS6666516 -0.65 0.7 -0.198 -0.05 0.7 -7.522 -0.39 0.9

RS4595321 -0.65 0.7 -0.198 -0.05 0.7 -7.522 -0.39 0.9

RS4564083 -0.65 0.7 -0.198 -0.05 0.7 -7.522 -0.39 0.9

RS1338302 -0.65 0.7 -0.198 -0.05 0.7 -7.522 -0.39 0.9

RS4649326 -0.65 0.7 -0.198 -0.05 0.7 -7.522 -0.39 0.9

RS789360 -0.65 0.7 -0.198 -0.05 0.7 -7.522 -0.39 0.9

RS789651 -0.65 0.7 -0.198 -0.05 0.7 -7.522 -0.39 0.9

RS4545285 -0.65 0.7 -0.198 -0.05 0.7 -7.522 -0.39 0.9

RS17808157 -0.65 0.7 -0.198 -0.05 0.7 -7.522 -0.39 0.9

RS6689943 -0.65 0.7 -0.198 -0.05 0.7 -7.522 -0.39 0.9

RS9424612 -0.65 0.7 -0.198 -0.05 0.7 -7.522 -0.39 0.9

RS17453089 -0.65 0.7 -0.198 -0.05 0.7 -7.522 -0.39 0.9

RS3820119 -0.65 0.7 -0.198 -0.05 0.7 -7.522 -0.39 0.9

RS10752798 -0.65 0.7 -0.198 -0.05 0.7 -7.522 -0.39 0.9

RS1016405 -0.65 0.7 -0.198 -0.05 0.7 -7.522 -0.39 0.9

RS6685114 -0.65 0.7 -0.198 -0.05 0.7 -7.522 -0.39 0.9

RS16858853 -0.65 0.7 -0.198 -0.05 0.7 -7.522 -0.39 0.9

RS6697791 -0.65 0.7 -0.198 -0.05 0.7 -7.522 -0.39 0.9

RS10737197 -0.65 0.7 -0.198 -0.05 0.7 -7.522 -0.39 0.9

RS4233060 -0.65 0.7 -0.198 -0.05 0.7 -7.522 -0.39 0.9

RS3845310 -0.65 0.7 -0.198 -0.05 0.7 -7.522 -0.39 0.9

RS12740439 -0.65 0.7 -0.198 -0.05 0.7 -7.522 -0.39 0.9

RS4649335 -0.65 0.7 -0.198 -0.05 0.7 -7.522 -0.39 0.9

RS1693229 -0.65 0.7 -0.198 -0.05 0.7 -7.522 -0.39 0.9

RS7530423 -0.65 0.7 -0.198 -0.05 0.7 -7.522 -0.39 0.9

RS11802061 -0.65 0.7 -0.198 -0.05 0.7 -7.522 -0.39 0.9

RS4920167 -0.65 0.7 -0.198 -0.05 0.7 -7.522 -0.39 0.9

RS4027081 -0.65 0.7 -0.198 -0.05 0.7 -7.522 -0.39 0.9

RS6695462 -0.65 0.7 -0.198 -0.05 0.7 -7.522 -0.39 0.9

RS10910404 -0.65 0.7 -0.198 -0.05 0.7 -7.522 -0.39 0.9

RS10752781 -0.65 0.7 -0.198 -0.05 0.7 -7.522 -0.39 0.9

RS6669190 -0.65 0.7 -0.198 -0.05 0.7 -7.522 -0.39 0.9

RS915184 -0.65 0.7 -0.198 -0.05 0.7 -7.522 -0.39 0.9

RS1619856 -0.65 0.7 -0.198 -0.05 0.7 -7.522 -0.39 0.9

RS270505 -0.65 0.7 -0.198 -0.05 0.7 -7.522 -0.39 0.9

RS2793861 -0.65 0.7 -0.198 -0.05 0.7 -7.522 -0.39 0.9

RS6673545 -0.65 0.7 -0.198 -0.05 0.7 -7.522 -0.39 0.9

RS6663049 -0.65 0.7 -0.198 -0.05 0.7 -7.522 -0.39 0.9

RS564212 -0.65 0.7 -0.198 -0.05 0.7 -7.522 -0.39 0.9

RS822928 -0.65 0.7 -0.198 -0.05 0.7 -7.522 -0.39 0.9

RS2439500 -0.65 0.7 -0.198 -0.05 0.7 -7.522 -0.39 0.9

RS3914503 -0.65 0.7 -0.198 -0.05 0.7 -7.522 -0.39 0.9

RS1515656 -0.65 0.7 -0.198 -0.05 0.7 -7.522 -0.39 0.9

RS2674005 -0.65 0.7 -0.198 -0.05 0.7 -7.522 -0.39 0.9

RS908329 -0.65 0.7 -0.198 -0.05 0.7 -7.522 -0.39 0.9

RS2802963 -0.65 0.7 -0.198 -0.05 0.7 -7.522 -0.39 0.9

RS7531062 -0.65 0.7 -0.198 -0.05 0.7 -7.522 -0.39 0.9

RS9727449 -0.65 0.7 -0.198 -0.05 0.7 -7.522 -0.39 0.9

RS10925482 -0.65 0.7 -0.198 -0.05 0.7 -7.522 -0.39 0.9

RS2774335 -0.65 0.7 -0.198 -0.05 0.7 -7.522 -0.39 0.9

RS6429196 -0.65 0.7 -0.198 -0.05 0.7 -7.522 -0.39 0.9

RS10926264 -0.65 0.7 -0.198 -0.05 0.7 -7.522 -0.39 0.9

RS7554426 -0.65 0.7 -0.198 -0.05 0.7 -7.522 -0.39 0.9

RS7545788 -0.65 0.7 -0.198 -0.05 0.7 -7.522 -0.39 0.9

RS10803141 -0.65 0.7 -0.198 -0.05 0.7 -7.522 -0.39 0.9

RS12566222 -0.65 0.7 -0.198 -0.05 0.7 -7.522 -0.39 0.9

RS4659618 -0.65 0.7 -0.198 -0.05 0.7 -7.522 -0.39 0.9

RS927730 -0.65 0.7 -0.198 -0.05 0.7 -7.522 -0.39 0.9

RS4659633 -0.65 0.7 -0.198 -0.05 0.7 -7.522 -0.39 0.9

RS557205 -0.65 0.7 -0.198 -0.05 0.7 -7.522 -0.39 0.9

RS605998 -0.65 0.7 -0.198 -0.05 0.7 -7.522 -0.39 0.9

RS2120959 -0.65 0.7 -0.198 -0.05 0.7 -7.522 -0.39 0.9

RS635487 -0.65 0.7 -0.198 -0.05 0.7 -7.522 -0.39 0.9

RS17582358 -0.65 0.7 -0.198 -0.05 0.7 -7.522 -0.39 0.9

RS1253618 -0.65 0.7 -0.198 -0.05 0.7 -7.522 -0.39 0.9

RS7512191 -0.65 0.7 -0.198 -0.05 0.7 -7.522 -0.39 0.9

RS12753714 -0.65 0.7 -0.198 -0.05 0.7 -7.522 -0.39 0.9

RS10925137 -0.65 0.7 -0.198 -0.05 0.7 -7.522 -0.39 0.9

RS3905125 -0.65 0.7 -0.198 -0.05 0.7 -7.522 -0.39 0.9

RS2275568 -0.65 0.7 -0.198 -0.05 0.7 -7.522 -0.39 0.9

RS12403151 -0.65 0.7 -0.198 -0.05 0.7 -7.522 -0.39 0.9

RS12125175 -0.65 0.7 -0.198 -0.05 0.7 -7.522 -0.39 0.9

RS2485564 -0.65 0.7 -0.198 -0.05 0.7 -7.522 -0.39 0.9

RS6661229 -0.65 0.7 -0.198 -0.05 0.7 -7.522 -0.39 0.9

RS2275287 -0.65 0.7 -0.198 -0.05 0.7 -7.522 -0.39 0.9

RS10802607 -0.65 0.7 -0.198 -0.05 0.7 -7.522 -0.39 0.9

RS4593814 -0.65 0.7 -0.198 -0.05 0.7 -7.522 -0.39 0.9

RS2010045 -0.65 0.7 -0.198 -0.05 0.7 -7.522 -0.39 0.9

RS1031862 -0.65 0.7 -0.198 -0.05 0.7 -7.522 -0.39 0.9

RS1817410 -0.65 0.7 -0.198 -0.05 0.7 -7.522 -0.39 0.9

RS490057 -0.65 0.7 -0.198 -0.05 0.7 -7.522 -0.39 0.9

RS2250079 -0.65 0.7 -0.198 -0.05 0.7 -7.522 -0.39 0.9

RS2790347 -0.65 0.7 -0.198 -0.05 0.7 -7.522 -0.39 0.9

RS2819742 -0.65 0.7 -0.198 -0.05 0.7 -7.522 -0.39 0.9

RS6703045 -0.57 0.7 -0.198 -0.05 0.7 -7.146 -0.30 0.9

RS12085662 -0.55 0.7 -0.198 -0.05 0.7 -7.067 -0.29 0.9

RS12123002 -0.21 0.6 -0.198 -0.02 0.6 -0.316 -0.01 0.6

RS2499604 -0.21 0.6 -0.198 -0.02 0.6 -0.316 -0.01 0.6

RS2499566 -0.21 0.6 -0.198 -0.02 0.6 -0.316 -0.01 0.6

RS2487091 -0.21 0.6 -0.198 -0.02 0.6 -0.316 -0.01 0.6

RS10925623 -0.21 0.6 -0.198 -0.02 0.6 -0.316 -0.01 0.6

RS6678429 -0.21 0.6 -0.198 -0.02 0.6 -0.316 -0.01 0.6

RS923543 -0.21 0.6 -0.198 -0.02 0.6 -0.316 -0.01 0.6

RS10925644 -0.21 0.6 -0.198 -0.02 0.6 -0.316 -0.01 0.6

RS11810140 -0.21 0.6 -0.198 -0.02 0.6 -0.316 -0.01 0.6

RS535043 -0.21 0.6 -0.198 -0.02 0.6 -0.316 -0.01 0.6

RS10754644 -0.21 0.6 -0.198 -0.02 0.6 -0.316 -0.01 0.6

RS683070 -0.21 0.6 -0.198 -0.02 0.6 -0.316 -0.01 0.6

RS6678175 -0.21 0.6 -0.198 -0.02 0.6 -0.316 -0.01 0.6

RS12726612 -0.21 0.6 -0.198 -0.02 0.6 -0.316 -0.01 0.6

RS2202215 -0.21 0.6 -0.198 -0.02 0.6 -0.316 -0.01 0.6

RS10925740 -0.21 0.6 -0.198 -0.02 0.6 -0.316 -0.01 0.6

RS3011577 -0.21 0.6 -0.198 -0.02 0.6 -0.316 -0.01 0.6

RS10925806 -0.21 0.6 -0.198 -0.02 0.6 -0.316 -0.01 0.6

RS1509701 -0.21 0.6 -0.198 -0.02 0.6 -0.316 -0.01 0.6

RS1848751 -0.21 0.6 -0.198 -0.02 0.6 -0.316 -0.01 0.6

RS6657123 -0.21 0.6 -0.198 -0.02 0.6 -0.316 -0.01 0.6

RS9787223 -0.21 0.6 -0.198 -0.02 0.6 -0.316 -0.01 0.6

RS6703086 -0.21 0.6 -0.198 -0.02 0.6 -0.316 -0.01 0.6

RS2664057 -0.21 0.6 -0.198 -0.02 0.6 -0.316 -0.01 0.6

RS10802757 -0.21 0.6 -0.198 -0.02 0.6 -0.316 -0.01 0.6

RS10925867 -0.21 0.6 -0.198 -0.02 0.6 -0.316 -0.01 0.6

RS12041476 -0.21 0.6 -0.198 -0.02 0.6 -0.316 -0.01 0.6

RS6661899 -0.21 0.6 -0.198 -0.02 0.6 -0.316 -0.01 0.6

RS7551236 -0.21 0.6 -0.198 -0.02 0.6 -0.316 -0.01 0.6

RS9287229 -0.21 0.6 -0.198 -0.02 0.6 -0.316 -0.01 0.6

RS10925910 -0.21 0.6 -0.198 -0.02 0.6 -0.316 -0.01 0.6

RS12406493 -0.21 0.6 -0.198 -0.02 0.6 -0.316 -0.01 0.6

RS532718 -0.21 0.6 -0.198 -0.02 0.6 -0.316 -0.01 0.6

RS12072181 -0.21 0.6 -0.198 -0.02 0.6 -0.316 -0.01 0.6

RS12742834 -0.21 0.6 -0.198 -0.02 0.6 -0.316 -0.01 0.6

RS4659940 -0.21 0.6 -0.198 -0.02 0.6 -0.316 -0.01 0.6

RS10926089 -0.21 0.6 -0.198 -0.02 0.6 -0.316 -0.01 0.6

RS6693552 -0.21 0.6 -0.198 -0.02 0.6 -0.316 -0.01 0.6

RS6690640 -0.21 0.6 -0.198 -0.02 0.6 -0.316 -0.01 0.6

RS17669357 -0.21 0.6 -0.198 -0.02 0.6 -0.316 -0.01 0.6

RS1537850 -0.21 0.6 -0.198 -0.02 0.6 -0.316 -0.01 0.6

RS2152556 -0.21 0.6 -0.198 -0.02 0.6 -0.316 -0.01 0.6

RS12139890 -0.21 0.6 -0.198 -0.02 0.6 -0.316 -0.01 0.6

RS11590081 -0.21 0.6 -0.198 -0.02 0.6 -0.316 -0.01 0.6

RS10802871 -0.21 0.6 -0.198 -0.02 0.6 -0.316 -0.01 0.6

RS9442211 -0.21 0.6 -0.198 -0.02 0.6 -0.316 -0.01 0.6

RS12137158 -0.21 0.6 -0.198 -0.02 0.6 -0.316 -0.01 0.6

RS2185285 -0.21 0.6 -0.198 -0.02 0.6 -0.316 -0.01 0.6

RS6702434 -0.21 0.6 -0.198 -0.02 0.6 -0.316 -0.01 0.6

RS10495471 -0.21 0.6 -0.198 -0.02 0.6 -0.316 -0.01 0.6

RS12069813 -0.21 0.6 -0.198 -0.02 0.6 -0.316 -0.01 0.6

RS10802901 -0.21 0.6 -0.198 -0.02 0.6 -0.316 -0.01 0.6

RS1361748 -0.21 0.6 -0.198 -0.02 0.6 -0.316 -0.01 0.6

RS3911618 -0.21 0.6 -0.198 -0.02 0.6 -0.316 -0.01 0.6

RS6657011 -0.21 0.6 -0.198 -0.02 0.6 -0.316 -0.01 0.6

RS523361 -0.21 0.6 -0.198 -0.02 0.6 -0.316 -0.01 0.6

RS10926408 -0.21 0.6 -0.198 -0.02 0.6 -0.316 -0.01 0.6

RS2678780 -0.21 0.6 -0.198 -0.02 0.6 -0.316 -0.01 0.6

RS12126298 -0.21 0.6 -0.198 -0.02 0.6 -0.316 -0.01 0.6

RS6429246 -0.21 0.6 -0.198 -0.02 0.6 -0.316 -0.01 0.6

RS6667456 -0.21 0.6 -0.198 -0.02 0.6 -0.316 -0.01 0.6

RS1915877 -0.21 0.6 -0.198 -0.02 0.6 -0.316 -0.01 0.6

RS2686240 -0.21 0.6 -0.198 -0.02 0.6 -0.316 -0.01 0.6

RS9428501 -0.21 0.6 -0.198 -0.02 0.6 -0.316 -0.01 0.6

RS7512603 -0.21 0.6 -0.198 -0.02 0.6 -0.316 -0.01 0.6

RS10737861 -0.21 0.6 -0.198 -0.02 0.6 -0.316 -0.01 0.6

RS4660075 -0.21 0.6 -0.198 -0.02 0.6 -0.316 -0.01 0.6

RS2341938 -0.21 0.6 -0.198 -0.02 0.6 -0.316 -0.01 0.6

RS1544190 -0.21 0.6 -0.198 -0.02 0.6 -0.316 -0.01 0.6

RS10157091 -0.21 0.6 -0.198 -0.02 0.6 -0.316 -0.01 0.6

RS1053230 -0.21 0.6 -0.198 -0.02 0.6 -0.316 -0.01 0.6

RS1053183 -0.21 0.6 -0.198 -0.02 0.6 -0.316 -0.01 0.6

RS12133073 -0.21 0.6 -0.198 -0.02 0.6 -0.316 -0.01 0.6

RS7524442 -0.21 0.6 -0.198 -0.02 0.6 -0.316 -0.01 0.6

RS12565340 -0.21 0.6 -0.198 -0.02 0.6 -0.316 -0.01 0.6

RS2526699 -0.21 0.6 -0.198 -0.02 0.6 -0.316 -0.01 0.6

RS851785 -0.21 0.6 -0.198 -0.02 0.6 -0.316 -0.01 0.6

RS6664298 -0.21 0.6 -0.198 -0.02 0.6 -0.316 -0.01 0.6

RS3845563 -0.21 0.6 -0.198 -0.02 0.6 -0.316 -0.01 0.6

RS3851304 -0.21 0.6 -0.198 -0.02 0.6 -0.316 -0.01 0.6

RS28757855 -0.21 0.6 -0.198 -0.02 0.6 -0.316 -0.01 0.6

RS9428536 -0.21 0.6 -0.198 -0.02 0.6 -0.316 -0.01 0.6

RS892929 -0.21 0.6 -0.198 -0.02 0.6 -0.316 -0.01 0.6

RS2036408 -0.21 0.6 -0.198 -0.02 0.6 -0.316 -0.01 0.6

RS6690415 -0.21 0.6 -0.198 -0.02 0.6 -0.316 -0.01 0.6

RS1609951 -0.21 0.6 -0.198 -0.02 0.6 -0.316 -0.01 0.6

RS1837400 -0.21 0.6 -0.198 -0.02 0.6 -0.316 -0.01 0.6

RS3863747 -0.21 0.6 -0.198 -0.02 0.6 -0.316 -0.01 0.6

RS7532175 -0.21 0.6 -0.198 -0.02 0.6 -0.316 -0.01 0.6

RS2810017 -0.21 0.6 -0.198 -0.02 0.6 -0.316 -0.01 0.6

RS11589847 -0.21 0.6 -0.198 -0.02 0.6 -0.316 -0.01 0.6

RS4658484 -0.21 0.6 -0.198 -0.02 0.6 -0.316 -0.01 0.6

RS10926756 -0.21 0.6 -0.198 -0.02 0.6 -0.316 -0.01 0.6

RS6658662 -0.21 0.6 -0.198 -0.02 0.6 -0.316 -0.01 0.6

RS6686107 -0.21 0.6 -0.198 -0.02 0.6 -0.316 -0.01 0.6

RS2184185 -0.21 0.6 -0.198 -0.02 0.6 -0.316 -0.01 0.6

RS10159343 -0.21 0.6 -0.198 -0.02 0.6 -0.316 -0.01 0.6

RS10926825 -0.21 0.6 -0.198 -0.02 0.6 -0.316 -0.01 0.6

RS12561770 -0.21 0.6 -0.198 -0.02 0.6 -0.316 -0.01 0.6

RS2780784 -0.21 0.6 -0.198 -0.02 0.6 -0.316 -0.01 0.6

RS12407970 -0.21 0.6 -0.198 -0.02 0.6 -0.316 -0.01 0.6

RS12032023 -0.21 0.6 -0.198 -0.02 0.6 -0.316 -0.01 0.6

RS4658507 -0.21 0.6 -0.198 -0.02 0.6 -0.316 -0.01 0.6

RS3003542 -0.21 0.6 -0.198 -0.02 0.6 -0.316 -0.01 0.6

RS6429411 -0.21 0.6 -0.198 -0.02 0.6 -0.316 -0.01 0.6

RS2802723 -0.21 0.6 -0.198 -0.02 0.6 -0.316 -0.01 0.6

RS4658405 -0.21 0.6 -0.198 -0.02 0.6 -0.316 -0.01 0.6

RS543626 -0.21 0.6 -0.198 -0.02 0.6 -0.316 -0.01 0.6

RS692981 -0.21 0.6 -0.198 -0.02 0.6 -0.316 -0.01 0.6

RS2486538 -0.21 0.6 -0.198 -0.02 0.6 -0.316 -0.01 0.6

RS2454238 -0.21 0.6 -0.198 -0.02 0.6 -0.316 -0.01 0.6

RS10927144 -0.21 0.6 -0.198 -0.02 0.6 -0.316 -0.01 0.6

RS4658413 -0.21 0.6 -0.198 -0.02 0.6 -0.316 -0.01 0.6

RS7555626 -0.21 0.6 -0.198 -0.02 0.6 -0.316 -0.01 0.6

RS6429457 -0.21 0.6 -0.198 -0.02 0.6 -0.316 -0.01 0.6

RS10803196 -0.21 0.6 -0.198 -0.02 0.6 -0.316 -0.01 0.6

RS10927202 -0.21 0.6 -0.198 -0.02 0.6 -0.316 -0.01 0.6

RS7547047 -0.21 0.6 -0.198 -0.02 0.6 -0.316 -0.01 0.6

RS3101457 -0.21 0.6 -0.198 -0.02 0.6 -0.316 -0.01 0.6

RS12068598 -0.21 0.6 -0.198 -0.02 0.6 -0.316 -0.01 0.6

RS10803228 -0.21 0.6 -0.198 -0.02 0.6 -0.316 -0.01 0.6

RS6695943 -0.21 0.6 -0.198 -0.02 0.6 -0.316 -0.01 0.6

RS4658662 -0.21 0.6 -0.198 -0.02 0.6 -0.316 -0.01 0.6

RS9662967 -0.21 0.6 -0.198 -0.02 0.6 -0.316 -0.01 0.6

RS2172955 -0.21 0.6 -0.198 -0.02 0.6 -0.316 -0.01 0.6

RS6692143 -0.21 0.6 -0.198 -0.02 0.6 -0.316 -0.01 0.6

RS12039117 -0.29 0.6 -0.198 -0.02 0.6 -0.554 -0.03 0.6

RS6671796 -0.31 0.6 -0.198 -0.03 0.6 -0.638 -0.03 0.7

RS1173842 -0.37 0.6 -0.198 -0.03 0.6 -0.906 -0.05 0.7

RS819876 -0.37 0.6 -0.198 -0.03 0.6 -0.935 -0.05 0.7

RS1111813 -0.37 0.6 -0.198 -0.03 0.6 -0.935 -0.05 0.7

RS6687098 -0.37 0.6 -0.198 -0.03 0.6 -0.935 -0.05 0.7

RS1538471 -0.37 0.6 -0.198 -0.03 0.6 -0.935 -0.05 0.7

RS12133360 -0.38 0.6 -0.198 -0.03 0.6 -0.935 -0.05 0.7

RS10924243 -0.38 0.6 -0.198 -0.03 0.6 -0.935 -0.05 0.7

RS1620343 -0.37 0.6 -0.198 -0.03 0.6 -0.935 -0.05 0.7

RS947103 -0.37 0.6 -0.198 -0.03 0.6 -0.935 -0.05 0.7

RS1771523 -0.37 0.6 -0.198 -0.03 0.6 -0.935 -0.05 0.7

RS12045474 -0.38 0.6 -0.198 -0.03 0.6 -0.935 -0.05 0.7

RS2262933 -0.38 0.6 -0.198 -0.03 0.6 -0.936 -0.05 0.7

RS2791399 -0.38 0.6 -0.198 -0.03 0.6 -0.936 -0.05 0.7

RS1316491 -0.38 0.6 -0.198 -0.03 0.6 -0.936 -0.05 0.7

RS10924351 -0.38 0.6 -0.198 -0.03 0.6 -0.936 -0.05 0.7

RS12137129 -0.38 0.6 -0.198 -0.03 0.6 -0.936 -0.05 0.7

RS12137320 -0.38 0.6 -0.198 -0.03 0.6 -0.936 -0.05 0.7

RS1538294 -0.38 0.6 -0.198 -0.03 0.6 -0.936 -0.05 0.7

RS11586168 -0.38 0.6 -0.198 -0.03 0.6 -0.936 -0.05 0.7

RS10924537 -0.38 0.6 -0.198 -0.03 0.6 -0.936 -0.05 0.7

RS7550853 -0.38 0.6 -0.198 -0.03 0.6 -0.936 -0.05 0.7

RS10924739 -0.38 0.6 -0.198 -0.03 0.6 -0.936 -0.05 0.7

RS4654291 -0.38 0.6 -0.198 -0.03 0.6 -0.935 -0.05 0.7

RS3124075 -0.38 0.6 -0.198 -0.03 0.6 -0.936 -0.05 0.7

RS10802432 -0.38 0.6 -0.198 -0.03 0.6 -0.936 -0.05 0.7

RS6694274 -0.38 0.6 -0.198 -0.03 0.6 -0.936 -0.05 0.7

RS10924852 -0.38 0.6 -0.198 -0.03 0.6 -0.936 -0.05 0.7

RS1779985 -0.38 0.6 -0.198 -0.03 0.6 -0.936 -0.05 0.7

RS6426218 -0.38 0.6 -0.198 -0.03 0.6 -0.936 -0.05 0.7

RS9793102 -0.38 0.6 -0.198 -0.03 0.6 -0.936 -0.05 0.7

RS1609840 -0.38 0.6 -0.198 -0.03 0.6 -0.936 -0.05 0.7

RS12131404 -0.38 0.6 -0.198 -0.03 0.6 -0.936 -0.05 0.7

RS11581163 -0.38 0.6 -0.198 -0.03 0.6 -0.936 -0.05 0.7

RS6702432 -0.38 0.6 -0.198 -0.03 0.6 -0.936 -0.05 0.7

RS12137901 -0.38 0.6 -0.198 -0.03 0.6 -0.936 -0.05 0.7

RS7525979 -0.38 0.6 -0.198 -0.03 0.6 -0.936 -0.05 0.7

RS10925027 -0.38 0.6 -0.198 -0.03 0.6 -0.935 -0.05 0.7

RS4472787 -0.37 0.6 -0.198 -0.03 0.6 -0.935 -0.05 0.7

RS1881797 -0.37 0.6 -0.198 -0.03 0.6 -0.934 -0.05 0.7

RS1105488 -0.37 0.6 -0.198 -0.03 0.6 -0.934 -0.05 0.7

RS3862182 -0.37 0.6 -0.198 -0.03 0.6 -0.934 -0.05 0.7

RS12402077 -0.37 0.6 -0.198 -0.03 0.6 -0.934 -0.05 0.7

RS1176018 -0.37 0.6 -0.198 -0.03 0.6 -0.934 -0.05 0.7

RS1391483 -0.37 0.6 -0.198 -0.03 0.6 -0.934 -0.05 0.7

RS10888252 -0.37 0.6 -0.198 -0.03 0.6 -0.934 -0.05 0.7

RS6587386 -0.37 0.6 -0.198 -0.03 0.6 -0.933 -0.05 0.7

RS10888268 -0.37 0.6 -0.198 -0.03 0.6 -0.933 -0.05 0.7

Analysing Chromosome 2

Phenotype: AFFSTAT [ALL] (1 family)

===============================================================================

Pos Zmean pvalue linDelta LOD pvalue expDelta LOD pvalue

min -0.76 0.8 -0.198 -0.06 0.7 -9.999 -0.55 0.9

max 5.05 0.00000 1.309 0.88 0.02 9.999 2.62 0.0003

RS436446 4.67 0.00000 1.309 0.85 0.02 3.130 1.61 0.003

RS12714396 4.67 0.00000 1.309 0.85 0.02 3.125 1.61 0.003

RS12472764 4.70 0.00000 1.309 0.85 0.02 3.125 1.62 0.003

RS2685230 4.70 0.00000 1.309 0.85 0.02 3.125 1.62 0.003

RS2724913 4.70 0.00000 1.309 0.85 0.02 3.125 1.62 0.003

RS6707477 4.70 0.00000 1.309 0.85 0.02 3.125 1.62 0.003

RS4549126 4.40 0.00001 1.309 0.83 0.03 3.125 1.58 0.004

RS11127524 2.65 0.004 1.309 0.65 0.04 3.125 1.27 0.008

RS4263142 2.19 0.014 1.309 0.59 0.05 3.124 1.12 0.011

RS6419706 1.83 0.03 1.309 0.53 0.06 3.123 0.97 0.02

RS4971358 1.39 0.08 1.309 0.45 0.08 3.120 0.64 0.04

RS6548285 1.00 0.2 1.309 0.36 0.10 0.460 0.12 0.2

RS4586665 1.00 0.2 1.309 0.36 0.10 0.460 0.12 0.2

RS6754505 1.00 0.2 1.309 0.36 0.10 0.460 0.12 0.2

RS4927600 1.00 0.2 1.309 0.36 0.10 0.460 0.12 0.2

RS11675434 1.00 0.2 1.309 0.36 0.10 0.460 0.12 0.2

RS1567919 1.00 0.2 1.309 0.36 0.10 0.460 0.12 0.2

RS4927608 1.00 0.2 1.309 0.36 0.10 0.460 0.12 0.2

RS9752413 1.00 0.2 1.309 0.36 0.10 0.460 0.12 0.2

RS9752743 1.00 0.2 1.309 0.36 0.10 0.460 0.12 0.2

RS1544846 1.00 0.2 1.309 0.36 0.10 0.460 0.12 0.2

RS6706107 1.00 0.2 1.309 0.36 0.10 0.460 0.12 0.2

RS6759577 1.00 0.2 1.309 0.36 0.10 0.460 0.12 0.2

RS891878 0.24 0.4 1.309 0.12 0.2 -4.749 0.05 0.5

RS6709211 -0.47 0.7 -0.198 -0.04 0.7 -4.785 -0.24 0.9

RS10865533 -0.61 0.7 -0.198 -0.05 0.7 -4.789 -0.28 0.9

RS1862112 -0.61 0.7 -0.198 -0.05 0.7 -4.789 -0.28 0.9

RS12470297 -0.61 0.7 -0.198 -0.05 0.7 -4.789 -0.28 0.9

RS1024143 -0.61 0.7 -0.198 -0.05 0.7 -4.789 -0.28 0.9

RS9636493 -0.61 0.7 -0.198 -0.05 0.7 -4.789 -0.28 0.9

RS2668843 -0.61 0.7 -0.198 -0.05 0.7 -4.789 -0.28 0.9

RS7568276 -0.61 0.7 -0.198 -0.05 0.7 -4.789 -0.28 0.9

RS908355 -0.61 0.7 -0.198 -0.05 0.7 -4.789 -0.28 0.9

RS7576292 -0.61 0.7 -0.198 -0.05 0.7 -4.789 -0.28 0.9

RS1551128 -0.61 0.7 -0.198 -0.05 0.7 -4.789 -0.28 0.9

RS10184017 -0.61 0.7 -0.198 -0.05 0.7 -4.789 -0.28 0.9

RS17495097 -0.61 0.7 -0.198 -0.05 0.7 -4.789 -0.28 0.9

RS11127338 -0.61 0.7 -0.198 -0.05 0.7 -4.789 -0.28 0.9

RS2122728 -0.61 0.7 -0.198 -0.05 0.7 -4.789 -0.28 0.9

RS10203375 -0.61 0.7 -0.198 -0.05 0.7 -4.789 -0.28 0.9

RS2385327 -0.61 0.7 -0.198 -0.05 0.7 -4.789 -0.28 0.9

RS12988342 -0.61 0.7 -0.198 -0.05 0.7 -4.789 -0.28 0.9

RS7592135 -0.61 0.7 -0.198 -0.05 0.7 -4.788 -0.28 0.9

RS2031028 -0.61 0.7 -0.198 -0.05 0.7 -4.788 -0.28 0.9

RS1869416 -0.61 0.7 -0.198 -0.05 0.7 -4.789 -0.28 0.9

RS10165836 -0.61 0.7 -0.198 -0.05 0.7 -4.789 -0.28 0.9

RS10188054 -0.61 0.7 -0.198 -0.05 0.7 -4.788 -0.28 0.9

RS7592700 -0.61 0.7 -0.198 -0.05 0.7 -4.788 -0.28 0.9

RS7586092 -0.61 0.7 -0.198 -0.05 0.7 -4.789 -0.28 0.9

RS4583476 -0.61 0.7 -0.198 -0.05 0.7 -4.789 -0.28 0.9

RS3820897 -0.61 0.7 -0.198 -0.05 0.7 -4.789 -0.28 0.9

RS13000184 -0.61 0.7 -0.198 -0.05 0.7 -4.789 -0.28 0.9

RS357967 -0.61 0.7 -0.198 -0.05 0.7 -4.789 -0.28 0.9

RS12466058 -0.61 0.7 -0.198 -0.05 0.7 -4.789 -0.28 0.9

RS1454236 -0.61 0.7 -0.198 -0.05 0.7 -4.789 -0.28 0.9

RS10184902 -0.61 0.7 -0.198 -0.05 0.7 -4.789 -0.28 0.9

RS6542670 -0.61 0.7 -0.198 -0.05 0.7 -4.789 -0.28 0.9

RS1437700 -0.61 0.7 -0.198 -0.05 0.7 -4.789 -0.28 0.9

RS7589994 -0.61 0.7 -0.198 -0.05 0.7 -4.789 -0.28 0.9

RS938767 -0.61 0.7 -0.198 -0.05 0.7 -4.789 -0.28 0.9

RS11123646 -0.61 0.7 -0.198 -0.05 0.7 -4.789 -0.28 0.9

RS10165034 -0.61 0.7 -0.198 -0.05 0.7 -4.789 -0.28 0.9

RS2602629 -0.61 0.7 -0.198 -0.05 0.7 -4.789 -0.28 0.9

RS589804 -0.61 0.7 -0.198 -0.05 0.7 -4.789 -0.28 0.9

RS591265 -0.61 0.7 -0.198 -0.05 0.7 -4.789 -0.28 0.9

RS12711951 -0.61 0.7 -0.198 -0.05 0.7 -4.789 -0.28 0.9

RS826021 -0.61 0.7 -0.198 -0.05 0.7 -4.789 -0.28 0.9

RS1446137 -0.61 0.7 -0.198 -0.05 0.7 -4.790 -0.28 0.9

RS1439829 -0.61 0.7 -0.198 -0.05 0.7 -4.790 -0.28 0.9

RS11123605 -0.61 0.7 -0.198 -0.05 0.7 -4.790 -0.28 0.9

RS1154756 -0.61 0.7 -0.198 -0.05 0.7 -4.789 -0.28 0.9

RS1869408 -0.61 0.7 -0.198 -0.05 0.7 -4.789 -0.28 0.9

RS17330726 -0.61 0.7 -0.198 -0.05 0.7 -4.789 -0.28 0.9

RS10181393 -0.61 0.7 -0.198 -0.05 0.7 -4.789 -0.28 0.9

RS1404253 -0.61 0.7 -0.198 -0.05 0.7 -4.789 -0.28 0.9

RS10432402 -0.61 0.7 -0.198 -0.05 0.7 -4.789 -0.28 0.9

RS6734151 -0.61 0.7 -0.198 -0.05 0.7 -4.789 -0.28 0.9

RS6720039 -0.61 0.7 -0.198 -0.05 0.7 -4.789 -0.28 0.9

RS6431957 -0.61 0.7 -0.198 -0.05 0.7 -4.789 -0.28 0.9

RS969768 -0.61 0.7 -0.198 -0.05 0.7 -4.789 -0.28 0.9

RS2882274 -0.61 0.7 -0.198 -0.05 0.7 -4.789 -0.28 0.9

RS10929661 -0.61 0.7 -0.198 -0.05 0.7 -4.789 -0.28 0.9

RS17363466 -0.61 0.7 -0.198 -0.05 0.7 -4.789 -0.28 0.9

RS2722602 -0.61 0.7 -0.198 -0.05 0.7 -4.789 -0.28 0.9

RS2564075 -0.61 0.7 -0.198 -0.05 0.7 -4.789 -0.28 0.9

RS2722591 -0.61 0.7 -0.198 -0.05 0.7 -4.789 -0.28 0.9

RS6732445 -0.61 0.7 -0.198 -0.05 0.7 -4.789 -0.28 0.9

RS965021 -0.61 0.7 -0.198 -0.05 0.7 -4.789 -0.28 0.9

RS2609187 -0.61 0.7 -0.198 -0.05 0.7 -4.789 -0.28 0.9

RS2693864 -0.61 0.7 -0.198 -0.05 0.7 -4.789 -0.28 0.9

RS9798084 -0.61 0.7 -0.198 -0.05 0.7 -4.789 -0.28 0.9

RS9287668 -0.61 0.7 -0.198 -0.05 0.7 -4.789 -0.28 0.9

RS921229 -0.61 0.7 -0.198 -0.05 0.7 -4.789 -0.28 0.9

RS17369213 -0.61 0.7 -0.198 -0.05 0.7 -4.789 -0.28 0.9

RS12623864 -0.61 0.7 -0.198 -0.05 0.7 -4.789 -0.28 0.9

RS12995389 -0.61 0.7 -0.198 -0.05 0.7 -4.789 -0.28 0.9

RS4594412 -0.61 0.7 -0.198 -0.05 0.7 -4.789 -0.28 0.9

RS16865170 -0.61 0.7 -0.198 -0.05 0.7 -4.789 -0.28 0.9

RS3919811 -0.61 0.7 -0.198 -0.05 0.7 -4.789 -0.28 0.9

RS12619231 -0.61 0.7 -0.198 -0.05 0.7 -4.789 -0.28 0.9

RS11675022 -0.61 0.7 -0.198 -0.05 0.7 -4.789 -0.28 0.9

RS930978 -0.61 0.7 -0.198 -0.05 0.7 -4.789 -0.28 0.9

RS930976 -0.61 0.7 -0.198 -0.05 0.7 -4.789 -0.28 0.9

RS11694230 -0.61 0.7 -0.198 -0.05 0.7 -4.789 -0.28 0.9

RS954845 -0.61 0.7 -0.198 -0.05 0.7 -4.789 -0.28 0.9

RS6760979 -0.61 0.7 -0.198 -0.05 0.7 -4.789 -0.28 0.9

RS1566982 -0.61 0.7 -0.198 -0.05 0.7 -4.789 -0.28 0.9

RS2174519 -0.61 0.7 -0.198 -0.05 0.7 -4.789 -0.28 0.9

RS1876105 -0.61 0.7 -0.198 -0.05 0.7 -4.789 -0.28 0.9

RS771283 -0.67 0.7 -0.198 -0.05 0.7 -9.999 -0.49 0.9

RS771300 -0.68 0.8 -0.198 -0.05 0.7 -9.999 -0.52 0.9

RS6720046 -0.68 0.8 -0.198 -0.05 0.7 -9.999 -0.52 0.9

RS309292 -0.68 0.8 -0.198 -0.05 0.7 -9.999 -0.52 0.9

RS1901765 -0.68 0.8 -0.198 -0.05 0.7 -9.999 -0.52 0.9

RS4669139 -0.68 0.8 -0.198 -0.05 0.7 -9.999 -0.52 0.9

RS10495552 -0.68 0.8 -0.198 -0.05 0.7 -9.999 -0.52 0.9

RS870863 -0.68 0.8 -0.198 -0.05 0.7 -9.999 -0.52 0.9

RS10191135 -0.68 0.8 -0.198 -0.05 0.7 -9.999 -0.52 0.9

RS6431885 -0.68 0.8 -0.198 -0.05 0.7 -9.999 -0.52 0.9

RS6708659 -0.68 0.8 -0.198 -0.05 0.7 -9.999 -0.52 0.9

RS1861500 -0.68 0.8 -0.198 -0.05 0.7 -9.999 -0.52 0.9

RS4669228 -0.68 0.8 -0.198 -0.05 0.7 -9.999 -0.52 0.9

RS6718111 -0.68 0.8 -0.198 -0.05 0.7 -9.999 -0.52 0.9

RS4668561 -0.68 0.8 -0.198 -0.05 0.7 -9.999 -0.52 0.9

RS3102947 -0.68 0.8 -0.198 -0.05 0.7 -9.999 -0.52 0.9

RS7587352 -0.68 0.8 -0.198 -0.05 0.7 -9.999 -0.52 0.9

RS11693395 -0.68 0.8 -0.198 -0.05 0.7 -9.999 -0.52 0.9

RS6431949 -0.68 0.8 -0.198 -0.05 0.7 -9.999 -0.52 0.9

RS345893 -0.68 0.8 -0.198 -0.05 0.7 -9.999 -0.52 0.9

RS4669322 -0.68 0.8 -0.198 -0.05 0.7 -9.999 -0.52 0.9

RS4669330 -0.68 0.8 -0.198 -0.05 0.7 -9.999 -0.52 0.9

RS8179714 -0.68 0.8 -0.198 -0.05 0.7 -9.999 -0.52 0.9

RS9636252 -0.68 0.8 -0.198 -0.05 0.7 -9.999 -0.52 0.9

RS1543042 -0.68 0.8 -0.198 -0.05 0.7 -9.999 -0.52 0.9

RS10495563 -0.68 0.8 -0.198 -0.05 0.7 -9.999 -0.52 0.9

RS6432018 -0.68 0.8 -0.198 -0.05 0.7 -9.999 -0.52 0.9

RS4669413 -0.68 0.8 -0.198 -0.05 0.7 -9.999 -0.52 0.9

RS13422487 -0.68 0.8 -0.198 -0.05 0.7 -9.999 -0.52 0.9

RS2357266 -0.68 0.8 -0.198 -0.05 0.7 -9.999 -0.52 0.9

RS7592934 -0.68 0.8 -0.198 -0.05 0.7 -9.999 -0.52 0.9

RS7559852 -0.68 0.8 -0.198 -0.05 0.7 -9.999 -0.52 0.9

RS2303914 -0.68 0.8 -0.198 -0.05 0.7 -9.999 -0.52 0.9

RS16867256 -0.68 0.8 -0.198 -0.05 0.7 -9.999 -0.52 0.9

RS2011516 -0.68 0.8 -0.198 -0.05 0.7 -9.999 -0.52 0.9

RS6432057 -0.68 0.8 -0.198 -0.05 0.7 -9.999 -0.52 0.9

RS12622986 -0.68 0.8 -0.198 -0.05 0.7 -9.999 -0.52 0.9

RS4668664 -0.68 0.8 -0.198 -0.05 0.7 -9.999 -0.52 0.9

RS2357468 -0.68 0.8 -0.198 -0.05 0.7 -9.999 -0.52 0.9

RS741260 -0.68 0.8 -0.198 -0.05 0.7 -9.999 -0.52 0.9

RS887958 -0.68 0.8 -0.198 -0.05 0.7 -9.999 -0.52 0.9

RS2110638 -0.68 0.8 -0.198 -0.05 0.7 -9.999 -0.52 0.9

RS12052519 -0.68 0.8 -0.198 -0.05 0.7 -9.999 -0.52 0.9

RS1476985 -0.68 0.8 -0.198 -0.05 0.7 -9.999 -0.52 0.9

RS7560149 -0.68 0.8 -0.198 -0.05 0.7 -9.999 -0.52 0.9

RS1420107 -0.68 0.8 -0.198 -0.05 0.7 -9.999 -0.52 0.9

RS4668685 -0.68 0.8 -0.198 -0.05 0.7 -9.999 -0.52 0.9

RS1405948 -0.68 0.8 -0.198 -0.05 0.7 -9.999 -0.52 0.9

RS818148 -0.68 0.8 -0.198 -0.05 0.7 -9.999 -0.52 0.9

RS6710665 -0.68 0.8 -0.198 -0.05 0.7 -9.999 -0.52 0.9

RS12475194 -0.68 0.8 -0.198 -0.05 0.7 -9.999 -0.52 0.9

RS12613865 -0.68 0.8 -0.198 -0.05 0.7 -9.999 -0.52 0.9

RS3732114 -0.68 0.8 -0.198 -0.05 0.7 -9.999 -0.52 0.9

RS6736664 -0.68 0.8 -0.198 -0.05 0.7 -9.999 -0.52 0.9

RS12468286 -0.68 0.8 -0.198 -0.05 0.7 -9.999 -0.52 0.9

RS1734343 -0.68 0.8 -0.198 -0.05 0.7 -9.999 -0.52 0.9

RS1734383 -0.68 0.8 -0.198 -0.05 0.7 -9.999 -0.52 0.9

RS2178740 -0.68 0.8 -0.198 -0.05 0.7 -9.999 -0.52 0.9

RS12623956 -0.68 0.8 -0.198 -0.05 0.7 -9.999 -0.52 0.9

RS921605 -0.68 0.8 -0.198 -0.05 0.7 -9.999 -0.52 0.9

RS7564899 -0.68 0.8 -0.198 -0.05 0.7 -9.999 -0.52 0.9

RS7608117 -0.68 0.8 -0.198 -0.05 0.7 -9.999 -0.52 0.9

RS6716817 -0.68 0.8 -0.198 -0.05 0.7 -9.999 -0.52 0.9

RS16857592 -0.68 0.8 -0.198 -0.05 0.7 -9.999 -0.52 0.9

RS12476319 -0.68 0.8 -0.198 -0.05 0.7 -9.999 -0.52 0.9

RS2304400 -0.68 0.8 -0.198 -0.05 0.7 -9.999 -0.52 0.9

RS33969665 -0.68 0.8 -0.198 -0.05 0.7 -9.999 -0.52 0.9

RS2304402 -0.68 0.8 -0.198 -0.05 0.7 -9.999 -0.52 0.9

RS7567183 -0.68 0.8 -0.198 -0.05 0.7 -9.999 -0.52 0.9

RS4669772 -0.67 0.7 -0.198 -0.05 0.7 -9.999 -0.48 0.9

RS10197022 -0.52 0.7 -0.198 -0.04 0.7 -2.334 -0.15 0.8

RS10495589 -0.52 0.7 -0.198 -0.04 0.7 -2.334 -0.15 0.8

RS11901591 -0.52 0.7 -0.198 -0.04 0.7 -2.334 -0.15 0.8

RS12477218 -0.52 0.7 -0.198 -0.04 0.7 -2.334 -0.15 0.8

RS2380426 -0.52 0.7 -0.198 -0.04 0.7 -2.334 -0.15 0.8

RS6432285 -0.52 0.7 -0.198 -0.04 0.7 -2.334 -0.15 0.8

RS10211429 -0.52 0.7 -0.198 -0.04 0.7 -2.334 -0.15 0.8

RS7594033 -0.52 0.7 -0.198 -0.04 0.7 -2.334 -0.15 0.8

RS4669855 -0.52 0.7 -0.198 -0.04 0.7 -2.334 -0.15 0.8

RS779919 -0.53 0.7 -0.198 -0.04 0.7 -2.639 -0.17 0.8

RS6432315 -0.55 0.7 -0.198 -0.04 0.7 -3.079 -0.19 0.8

RS890073 -0.60 0.7 -0.198 -0.05 0.7 -4.396 -0.26 0.9

RS6739633 -0.60 0.7 -0.198 -0.05 0.7 -4.396 -0.26 0.9

RS12692488 -0.60 0.7 -0.198 -0.05 0.7 -4.396 -0.26 0.9

RS2138500 -0.60 0.7 -0.198 -0.05 0.7 -4.396 -0.26 0.9

RS9808277 -0.60 0.7 -0.198 -0.05 0.7 -4.396 -0.26 0.9

RS2046266 -0.60 0.7 -0.198 -0.05 0.7 -4.396 -0.26 0.9

RS2380487 -0.60 0.7 -0.198 -0.05 0.7 -4.396 -0.26 0.9

RS10495614 -0.60 0.7 -0.198 -0.05 0.7 -4.396 -0.26 0.9

RS12618935 -0.60 0.7 -0.198 -0.05 0.7 -4.396 -0.26 0.9

RS12623139 -0.60 0.7 -0.198 -0.05 0.7 -4.396 -0.26 0.9

RS1947429 -0.60 0.7 -0.198 -0.05 0.7 -4.396 -0.26 0.9

RS2890456 -0.60 0.7 -0.198 -0.05 0.7 -4.396 -0.26 0.9

RS10206108 -0.60 0.7 -0.198 -0.05 0.7 -4.396 -0.26 0.9

RS360485 -0.60 0.7 -0.198 -0.05 0.7 -4.396 -0.26 0.9

RS12986520 -0.60 0.7 -0.198 -0.05 0.7 -4.396 -0.26 0.9

RS9287779 -0.60 0.7 -0.198 -0.05 0.7 -4.396 -0.26 0.9

RS875199 -0.60 0.7 -0.198 -0.05 0.7 -4.388 -0.26 0.9

RS12692539 -0.60 0.7 -0.198 -0.05 0.7 -4.388 -0.26 0.9

RS6720338 -0.60 0.7 -0.198 -0.05 0.7 -4.387 -0.26 0.9

RS2216536 -0.60 0.7 -0.198 -0.05 0.7 -4.386 -0.26 0.9

RS4668873 -0.60 0.7 -0.198 -0.05 0.7 -4.386 -0.26 0.9

RS6714977 -0.60 0.7 -0.198 -0.05 0.7 -4.386 -0.26 0.9

RS6431679 -0.60 0.7 -0.198 -0.05 0.7 -4.386 -0.26 0.9

RS4668936 -0.60 0.7 -0.198 -0.05 0.7 -4.396 -0.26 0.9

RS807597 -0.60 0.7 -0.198 -0.05 0.7 -4.396 -0.26 0.9

RS4668960 -0.60 0.7 -0.198 -0.05 0.7 -4.396 -0.26 0.9

RS6746441 -0.60 0.7 -0.198 -0.05 0.7 -4.396 -0.26 0.9

RS1981806 -0.60 0.7 -0.198 -0.05 0.7 -4.396 -0.26 0.9

RS2544527 -0.60 0.7 -0.198 -0.05 0.7 -4.396 -0.26 0.9

RS7583876 -0.60 0.7 -0.198 -0.05 0.7 -4.397 -0.26 0.9

RS12613665 -0.60 0.7 -0.198 -0.05 0.7 -4.397 -0.26 0.9

RS2380684 -0.60 0.7 -0.198 -0.05 0.7 -4.397 -0.26 0.9

RS13431879 -0.60 0.7 -0.198 -0.05 0.7 -4.396 -0.26 0.9

RS4233848 -0.60 0.7 -0.198 -0.05 0.7 -4.396 -0.26 0.9

RS11685620 -0.60 0.7 -0.198 -0.05 0.7 -4.396 -0.26 0.9

RS6735335 -0.60 0.7 -0.198 -0.05 0.7 -4.396 -0.26 0.9

RS1427682 -0.60 0.7 -0.198 -0.05 0.7 -4.396 -0.26 0.9

RS4669037 -0.60 0.7 -0.198 -0.05 0.7 -4.396 -0.26 0.9

RS16982164 -0.60 0.7 -0.198 -0.05 0.7 -4.397 -0.26 0.9

RS11894986 -0.60 0.7 -0.198 -0.05 0.7 -4.397 -0.26 0.9

RS12465117 -0.60 0.7 -0.198 -0.05 0.7 -4.397 -0.26 0.9

RS1430043 -0.60 0.7 -0.198 -0.05 0.7 -4.396 -0.26 0.9

RS1430058 -0.60 0.7 -0.198 -0.05 0.7 -4.396 -0.26 0.9

RS7586823 -0.60 0.7 -0.198 -0.05 0.7 -4.396 -0.26 0.9

RS1024925 -0.60 0.7 -0.198 -0.05 0.7 -4.396 -0.26 0.9

RS1722418 -0.60 0.7 -0.198 -0.05 0.7 -4.396 -0.26 0.9

RS1990834 -0.60 0.7 -0.198 -0.05 0.7 -4.396 -0.26 0.9

RS1643189 -0.60 0.7 -0.198 -0.05 0.7 -4.397 -0.26 0.9

RS10177601 -0.60 0.7 -0.198 -0.05 0.7 -4.396 -0.26 0.9

RS17386061 -0.60 0.7 -0.198 -0.05 0.7 -4.396 -0.26 0.9

RS17477667 -0.60 0.7 -0.198 -0.05 0.7 -4.396 -0.26 0.9

RS1996610 -0.60 0.7 -0.198 -0.05 0.7 -4.396 -0.26 0.9

RS1991315 -0.53 0.7 -0.198 -0.04 0.7 -3.143 -0.18 0.8

RS13393469 -0.45 0.7 -0.198 -0.04 0.7 -1.613 -0.10 0.7

RS12470034 -0.43 0.7 -0.198 -0.04 0.7 -1.392 -0.08 0.7

RS1564004 -0.40 0.7 -0.198 -0.03 0.7 -1.162 -0.07 0.7

RS6711333 -0.40 0.7 -0.198 -0.03 0.7 -1.118 -0.07 0.7

RS4073911 -0.39 0.7 -0.198 -0.03 0.7 -1.067 -0.06 0.7

RS10205974 -0.38 0.6 -0.198 -0.03 0.6 -0.972 -0.06 0.7

RS1516903 -0.38 0.6 -0.198 -0.03 0.6 -0.970 -0.06 0.7

RS16984751 -0.38 0.6 -0.198 -0.03 0.6 -0.961 -0.06 0.7

RS7562205 -0.38 0.6 -0.198 -0.03 0.6 -0.958 -0.06 0.7

RS4832589 -0.38 0.6 -0.198 -0.03 0.6 -0.951 -0.06 0.7

RS10190589 -0.38 0.6 -0.198 -0.03 0.6 -0.946 -0.05 0.7

RS11096540 -0.38 0.6 -0.198 -0.03 0.6 -0.946 -0.05 0.7

RS16985306 -0.38 0.6 -0.198 -0.03 0.6 -0.945 -0.05 0.7

RS17701746 -0.38 0.6 -0.198 -0.03 0.6 -0.944 -0.05 0.7

RS6531119 -0.38 0.6 -0.198 -0.03 0.6 -0.940 -0.05 0.7

RS12464512 -0.38 0.6 -0.198 -0.03 0.6 -0.937 -0.05 0.7

RS10169702 -0.37 0.6 -0.198 -0.03 0.6 -0.932 -0.05 0.7

RS1965786 -0.37 0.6 -0.198 -0.03 0.6 -0.932 -0.05 0.7

RS6707044 -0.37 0.6 -0.198 -0.03 0.6 -0.932 -0.05 0.7

RS12710706 -0.37 0.6 -0.198 -0.03 0.6 -0.932 -0.05 0.7

RS2060790 -0.37 0.6 -0.198 -0.03 0.6 -0.931 -0.05 0.7

RS11692444 -0.37 0.6 -0.198 -0.03 0.6 -0.931 -0.05 0.7

RS6705213 -0.37 0.6 -0.198 -0.03 0.6 -0.931 -0.05 0.7

RS17760223 -0.37 0.6 -0.198 -0.03 0.6 -0.931 -0.05 0.7

RS6733236 -0.37 0.6 -0.198 -0.03 0.6 -0.931 -0.05 0.7

RS17564315 -0.37 0.6 -0.198 -0.03 0.6 -0.931 -0.05 0.7

RS12710729 -0.37 0.6 -0.198 -0.03 0.6 -0.931 -0.05 0.7

RS988318 -0.37 0.6 -0.198 -0.03 0.6 -0.931 -0.05 0.7

RS1513833 -0.37 0.6 -0.198 -0.03 0.6 -0.931 -0.05 0.7

RS4666360 -0.37 0.6 -0.198 -0.03 0.6 -0.931 -0.05 0.7

RS3924843 -0.37 0.6 -0.198 -0.03 0.6 -0.931 -0.05 0.7

RS11898154 -0.37 0.6 -0.198 -0.03 0.6 -0.931 -0.05 0.7

RS2084920 -0.37 0.6 -0.198 -0.03 0.6 -0.931 -0.05 0.7

RS7572838 -0.37 0.6 -0.198 -0.03 0.6 -0.931 -0.05 0.7

RS2289083 -0.37 0.6 -0.198 -0.03 0.6 -0.931 -0.05 0.7

RS6728591 -0.37 0.6 -0.198 -0.03 0.6 -0.931 -0.05 0.7

RS4666420 -0.37 0.6 -0.198 -0.03 0.6 -0.931 -0.05 0.7

RS4666431 -0.37 0.6 -0.198 -0.03 0.6 -0.931 -0.05 0.7

RS934601 -0.37 0.6 -0.198 -0.03 0.6 -0.931 -0.05 0.7

RS722705 -0.37 0.6 -0.198 -0.03 0.6 -0.931 -0.05 0.7

RS6531255 -0.37 0.6 -0.198 -0.03 0.6 -0.931 -0.05 0.7

RS12994068 -0.37 0.6 -0.198 -0.03 0.6 -0.931 -0.05 0.7

RS4426495 -0.37 0.6 -0.198 -0.03 0.6 -0.931 -0.05 0.7

RS1713222 -0.37 0.6 -0.198 -0.03 0.6 -0.931 -0.05 0.7

RS571468 -0.37 0.6 -0.198 -0.03 0.6 -0.931 -0.05 0.7

RS7605304 -0.37 0.6 -0.198 -0.03 0.6 -0.931 -0.05 0.7

RS11689154 -0.37 0.6 -0.198 -0.03 0.6 -0.931 -0.05 0.7

RS219553 -0.37 0.6 -0.198 -0.03 0.6 -0.931 -0.05 0.7

RS7601920 -0.37 0.6 -0.198 -0.03 0.6 -0.931 -0.05 0.7

RS2338557 -0.37 0.6 -0.198 -0.03 0.6 -0.931 -0.05 0.7

RS992996 -0.37 0.6 -0.198 -0.03 0.6 -0.931 -0.05 0.7

RS1022377 -0.37 0.6 -0.198 -0.03 0.6 -0.931 -0.05 0.7

RS1876766 -0.37 0.6 -0.198 -0.03 0.6 -0.931 -0.05 0.7

RS1471767 -0.37 0.6 -0.198 -0.03 0.6 -0.931 -0.05 0.7

RS12469094 -0.37 0.6 -0.198 -0.03 0.6 -0.931 -0.05 0.7

RS2879555 -0.37 0.6 -0.198 -0.03 0.6 -0.931 -0.05 0.7

RS10206536 -0.37 0.6 -0.198 -0.03 0.6 -0.931 -0.05 0.7

RS6726919 -0.37 0.6 -0.198 -0.03 0.6 -0.931 -0.05 0.7

RS17321693 -0.37 0.6 -0.198 -0.03 0.6 -0.931 -0.05 0.7

RS2592781 -0.37 0.6 -0.198 -0.03 0.6 -0.931 -0.05 0.7

RS11676189 -0.37 0.6 -0.198 -0.03 0.6 -0.931 -0.05 0.7

RS1446875 -0.37 0.6 -0.198 -0.03 0.6 -0.931 -0.05 0.7

RS12478810 -0.37 0.6 -0.198 -0.03 0.6 -0.931 -0.05 0.7

RS1025702 -0.37 0.6 -0.198 -0.03 0.6 -0.931 -0.05 0.7

RS1561322 -0.37 0.6 -0.198 -0.03 0.6 -0.931 -0.05 0.7

RS2164866 -0.37 0.6 -0.198 -0.03 0.6 -0.931 -0.05 0.7

RS1709330 -0.37 0.6 -0.198 -0.03 0.6 -0.931 -0.05 0.7

RS1709318 -0.37 0.6 -0.198 -0.03 0.6 -0.931 -0.05 0.7

RS1709295 -0.37 0.6 -0.198 -0.03 0.6 -0.931 -0.05 0.7

RS7581165 -0.37 0.6 -0.198 -0.03 0.6 -0.931 -0.05 0.7

RS2712098 -0.37 0.6 -0.198 -0.03 0.6 -0.931 -0.05 0.7

RS2551325 -0.37 0.6 -0.198 -0.03 0.6 -0.931 -0.05 0.7

RS13035774 -0.37 0.6 -0.198 -0.03 0.6 -0.931 -0.05 0.7

RS2584937 -0.37 0.6 -0.198 -0.03 0.6 -0.931 -0.05 0.7

RS2044149 -0.37 0.6 -0.198 -0.03 0.6 -0.931 -0.05 0.7

RS6545729 -0.37 0.6 -0.198 -0.03 0.6 -0.931 -0.05 0.7

RS478222 -0.37 0.6 -0.198 -0.03 0.6 -0.931 -0.05 0.7

RS13395518 -0.37 0.6 -0.198 -0.03 0.6 -0.931 -0.05 0.7

RS2304429 -0.37 0.6 -0.198 -0.03 0.6 -0.931 -0.05 0.7

RS17745923 -0.37 0.6 -0.198 -0.03 0.6 -0.931 -0.05 0.7

RS10199771 -0.37 0.6 -0.198 -0.03 0.6 -0.931 -0.05 0.7

RS13004845 -0.37 0.6 -0.198 -0.03 0.6 -0.931 -0.05 0.7

RS2384319 -0.47 0.7 -0.198 -0.04 0.7 -2.145 -0.12 0.8

RS3792006 -0.58 0.7 -0.198 -0.05 0.7 -5.967 -0.27 0.9

RS7601607 -0.61 0.7 -0.198 -0.05 0.7 -6.656 -0.32 0.9

RS3739075 -0.65 0.7 -0.198 -0.05 0.7 -7.257 -0.38 0.9

RS2272464 -0.65 0.7 -0.198 -0.05 0.7 -7.257 -0.38 0.9

RS752472 -0.65 0.7 -0.198 -0.05 0.7 -7.257 -0.38 0.9

RS1731260 -0.65 0.7 -0.198 -0.05 0.7 -7.257 -0.38 0.9

RS1919126 -0.65 0.7 -0.198 -0.05 0.7 -7.257 -0.38 0.9

RS11901133 -0.65 0.7 -0.198 -0.05 0.7 -7.256 -0.38 0.9

RS10187665 -0.65 0.7 -0.198 -0.05 0.7 -7.257 -0.38 0.9

RS7576048 -0.65 0.7 -0.198 -0.05 0.7 -7.257 -0.38 0.9

RS11127145 -0.65 0.7 -0.198 -0.05 0.7 -7.257 -0.38 0.9

RS13411730 -0.65 0.7 -0.198 -0.05 0.7 -7.257 -0.38 0.9

RS10190267 -0.65 0.7 -0.198 -0.05 0.7 -7.257 -0.38 0.9

RS1581035 -0.65 0.7 -0.198 -0.05 0.7 -7.257 -0.38 0.9

RS7579759 -0.65 0.7 -0.198 -0.05 0.7 -7.257 -0.38 0.9

RS11885873 -0.65 0.7 -0.198 -0.05 0.7 -7.257 -0.38 0.9

RS1881254 -0.65 0.7 -0.198 -0.05 0.7 -7.257 -0.38 0.9

RS7595633 -0.65 0.7 -0.198 -0.05 0.7 -7.257 -0.38 0.9

RS11682523 -0.65 0.7 -0.198 -0.05 0.7 -7.257 -0.38 0.9

RS9653591 -0.65 0.7 -0.198 -0.05 0.7 -7.257 -0.38 0.9

RS6727948 -0.65 0.7 -0.198 -0.05 0.7 -7.257 -0.38 0.9

RS1867945 -0.65 0.7 -0.198 -0.05 0.7 -7.257 -0.38 0.9

RS3100241 -0.65 0.7 -0.198 -0.05 0.7 -7.257 -0.38 0.9

RS12997875 -0.65 0.7 -0.198 -0.05 0.7 -7.257 -0.38 0.9

RS12465220 -0.65 0.7 -0.198 -0.05 0.7 -7.257 -0.38 0.9

RS6547930 -0.65 0.7 -0.198 -0.05 0.7 -7.257 -0.38 0.9

RS4665463 -0.65 0.7 -0.198 -0.05 0.7 -7.257 -0.38 0.9

RS6547948 -0.65 0.7 -0.198 -0.05 0.7 -7.257 -0.38 0.9

RS4233746 -0.65 0.7 -0.198 -0.05 0.7 -7.257 -0.38 0.9

RS12466048 -0.65 0.7 -0.198 -0.05 0.7 -7.257 -0.38 0.9

RS11690312 -0.65 0.7 -0.198 -0.05 0.7 -7.257 -0.38 0.9

RS13026132 -0.65 0.7 -0.198 -0.05 0.7 -7.257 -0.38 0.9

RS4545964 -0.65 0.7 -0.198 -0.05 0.7 -7.257 -0.38 0.9

RS5018732 -0.65 0.7 -0.198 -0.05 0.7 -7.257 -0.38 0.9

RS6718327 -0.65 0.7 -0.198 -0.05 0.7 -7.257 -0.38 0.9

RS4952125 -0.65 0.7 -0.198 -0.05 0.7 -7.257 -0.38 0.9

RS1562450 -0.65 0.7 -0.198 -0.05 0.7 -7.257 -0.38 0.9

RS2028983 -0.65 0.7 -0.198 -0.05 0.7 -7.257 -0.38 0.9

RS7567202 -0.65 0.7 -0.198 -0.05 0.7 -7.257 -0.38 0.9

RS6718106 -0.65 0.7 -0.198 -0.05 0.7 -7.257 -0.38 0.9

RS7579944 -0.65 0.7 -0.198 -0.05 0.7 -7.257 -0.38 0.9

RS6718743 -0.65 0.7 -0.198 -0.05 0.7 -7.257 -0.38 0.9

RS4563275 -0.65 0.7 -0.198 -0.05 0.7 -7.257 -0.38 0.9

RS711237 -0.65 0.7 -0.198 -0.05 0.7 -7.257 -0.38 0.9

RS1662955 -0.65 0.7 -0.198 -0.05 0.7 -7.257 -0.38 0.9

RS13033111 -0.65 0.7 -0.198 -0.05 0.7 -7.256 -0.38 0.9

RS1880540 -0.65 0.7 -0.198 -0.05 0.7 -7.256 -0.38 0.9

RS2253464 -0.65 0.7 -0.198 -0.05 0.7 -7.257 -0.38 0.9

RS4597574 -0.65 0.7 -0.198 -0.05 0.7 -7.257 -0.38 0.9

RS2364430 -0.65 0.7 -0.198 -0.05 0.7 -7.257 -0.38 0.9

RS10179187 -0.65 0.7 -0.198 -0.05 0.7 -7.257 -0.38 0.9

RS4952017 -0.65 0.7 -0.198 -0.05 0.7 -7.257 -0.38 0.9

RS12465448 -0.65 0.7 -0.198 -0.05 0.7 -7.257 -0.38 0.9

RS1862978 -0.65 0.7 -0.198 -0.05 0.7 -7.256 -0.38 0.9

RS4952051 -0.65 0.7 -0.198 -0.05 0.7 -7.257 -0.38 0.9

RS4952064 -0.65 0.7 -0.198 -0.05 0.7 -7.257 -0.38 0.9

RS2344 -0.65 0.7 -0.198 -0.05 0.7 -7.257 -0.38 0.9

RS207417 -0.65 0.7 -0.198 -0.05 0.7 -7.257 -0.38 0.9

RS1054889 -0.65 0.7 -0.198 -0.05 0.7 -7.257 -0.38 0.9

RS743163 -0.65 0.7 -0.198 -0.05 0.7 -7.257 -0.38 0.9

RS6718214 -0.65 0.7 -0.198 -0.05 0.7 -7.257 -0.38 0.9

RS1031261 -0.65 0.7 -0.198 -0.05 0.7 -7.257 -0.38 0.9

RS979050 -0.65 0.7 -0.198 -0.05 0.7 -7.257 -0.38 0.9

RS6745339 -0.65 0.7 -0.198 -0.05 0.7 -7.257 -0.38 0.9

RS107195 -0.65 0.7 -0.198 -0.05 0.7 -7.257 -0.38 0.9

RS4952336 -0.65 0.7 -0.198 -0.05 0.7 -7.257 -0.38 0.9

RS218221 -0.65 0.7 -0.198 -0.05 0.7 -7.257 -0.38 0.9

RS1065324 -0.65 0.7 -0.198 -0.05 0.7 -7.257 -0.38 0.9

RS2305502 -0.65 0.7 -0.198 -0.05 0.7 -7.257 -0.38 0.9

RS642472 -0.65 0.7 -0.198 -0.05 0.7 -7.257 -0.38 0.9

RS2887087 -0.65 0.7 -0.198 -0.05 0.7 -7.256 -0.38 0.9

RS10188211 -0.65 0.7 -0.198 -0.05 0.7 -7.260 -0.38 0.9

RS6711516 -0.65 0.7 -0.198 -0.05 0.7 -7.261 -0.38 0.9

RS7576239 -0.65 0.7 -0.198 -0.05 0.7 -7.263 -0.38 0.9

RS17014142 -0.65 0.7 -0.198 -0.05 0.7 -7.263 -0.38 0.9

RS2140136 -0.65 0.7 -0.198 -0.05 0.7 -7.263 -0.38 0.9

RS1718712 -0.65 0.7 -0.198 -0.05 0.7 -7.264 -0.38 0.9

RS10495805 -0.65 0.7 -0.198 -0.05 0.7 -7.264 -0.38 0.9

RS12712378 -0.65 0.7 -0.198 -0.05 0.7 -7.263 -0.38 0.9

RS1368934 -0.65 0.7 -0.198 -0.05 0.7 -7.263 -0.38 0.9

RS892458 -0.65 0.7 -0.198 -0.05 0.7 -7.262 -0.38 0.9

RS6543865 -0.65 0.7 -0.198 -0.05 0.7 -7.258 -0.38 0.9

RS280734 -0.65 0.7 -0.198 -0.05 0.7 -7.257 -0.38 0.9

RS13391731 -0.65 0.7 -0.198 -0.05 0.7 -7.257 -0.38 0.9

RS10495831 -0.65 0.7 -0.198 -0.05 0.7 -7.257 -0.38 0.9

RS1922366 -0.65 0.7 -0.198 -0.05 0.7 -7.257 -0.38 0.9

RS13403909 -0.65 0.7 -0.198 -0.05 0.7 -7.257 -0.38 0.9

RS13384335 -0.65 0.7 -0.198 -0.05 0.7 -7.257 -0.38 0.9

RS11694690 -0.65 0.7 -0.198 -0.05 0.7 -7.257 -0.38 0.9

RS4560089 -0.65 0.7 -0.198 -0.05 0.7 -7.257 -0.38 0.9

RS13011599 -0.65 0.7 -0.198 -0.05 0.7 -7.257 -0.38 0.9

RS2968636 -0.65 0.7 -0.198 -0.05 0.7 -7.256 -0.38 0.9

RS1721781 -0.65 0.7 -0.198 -0.05 0.7 -7.256 -0.38 0.9

RS1397443 -0.65 0.7 -0.198 -0.05 0.7 -7.256 -0.38 0.9

RS10199383 -0.65 0.7 -0.198 -0.05 0.7 -7.256 -0.38 0.9

RS1167450 -0.65 0.7 -0.198 -0.05 0.7 -7.256 -0.38 0.9

RS2699150 -0.65 0.7 -0.198 -0.05 0.7 -7.255 -0.38 0.9

RS2052626 -0.65 0.7 -0.198 -0.05 0.7 -7.255 -0.38 0.9

RS7560345 -0.65 0.7 -0.198 -0.05 0.7 -7.255 -0.38 0.9

RS4670546 -0.65 0.7 -0.198 -0.05 0.7 -7.255 -0.38 0.9

RS4670145 -0.65 0.7 -0.198 -0.05 0.7 -7.256 -0.38 0.9

RS848503 -0.65 0.7 -0.198 -0.05 0.7 -7.256 -0.38 0.9

RS848642 -0.65 0.7 -0.198 -0.05 0.7 -7.256 -0.38 0.9

RS2372692 -0.65 0.7 -0.198 -0.05 0.7 -7.255 -0.38 0.9

RS10167726 -0.65 0.7 -0.198 -0.05 0.7 -7.255 -0.38 0.9

RS721348 -0.65 0.7 -0.198 -0.05 0.7 -7.255 -0.38 0.9

RS11124542 -0.65 0.7 -0.198 -0.05 0.7 -7.255 -0.38 0.9

RS3821147 -0.65 0.7 -0.198 -0.05 0.7 -7.255 -0.38 0.9

RS888083 -0.65 0.7 -0.198 -0.05 0.7 -7.256 -0.38 0.9

RS12712526 -0.58 0.7 -0.198 -0.05 0.7 -5.203 -0.26 0.9

RS10460527 -0.56 0.7 -0.198 -0.05 0.7 -4.140 -0.22 0.8

RS4670686 -0.55 0.7 -0.198 -0.04 0.7 -3.895 -0.21 0.8

RS10192261 -0.52 0.7 -0.198 -0.04 0.7 -2.869 -0.16 0.8

RS17021120 -0.49 0.7 -0.198 -0.04 0.7 -2.152 -0.13 0.8

RS10165323 -0.46 0.7 -0.198 -0.04 0.7 -1.610 -0.10 0.8

RS10865127 -0.46 0.7 -0.198 -0.04 0.7 -1.535 -0.10 0.7

RS11687301 -0.46 0.7 -0.198 -0.04 0.7 -1.536 -0.10 0.7

RS11124631 -0.46 0.7 -0.198 -0.04 0.7 -1.535 -0.10 0.7

RS163084 -0.46 0.7 -0.198 -0.04 0.7 -1.535 -0.10 0.7

RS232572 -0.46 0.7 -0.198 -0.04 0.7 -1.535 -0.10 0.7

RS232588 -0.46 0.7 -0.198 -0.04 0.7 -1.535 -0.10 0.7

RS11894455 -0.46 0.7 -0.198 -0.04 0.7 -1.536 -0.10 0.7

RS6720959 -0.46 0.7 -0.198 -0.04 0.7 -1.535 -0.10 0.7

RS4490160 -0.46 0.7 -0.198 -0.04 0.7 -1.535 -0.10 0.7

RS7601994 -0.46 0.7 -0.198 -0.04 0.7 -1.535 -0.10 0.7

RS4670264 -0.46 0.7 -0.198 -0.04 0.7 -1.535 -0.10 0.7

RS13014787 -0.46 0.7 -0.198 -0.04 0.7 -1.535 -0.10 0.7

RS9677244 -0.46 0.7 -0.198 -0.04 0.7 -1.536 -0.10 0.7

RS2110952 -0.46 0.7 -0.198 -0.04 0.7 -1.536 -0.10 0.7

RS6749057 -0.46 0.7 -0.198 -0.04 0.7 -1.536 -0.10 0.7

RS4670978 -0.46 0.7 -0.198 -0.04 0.7 -1.536 -0.10 0.7

RS2192703 -0.46 0.7 -0.198 -0.04 0.7 -1.536 -0.10 0.7

RS7566309 -0.46 0.7 -0.198 -0.04 0.7 -1.536 -0.10 0.7

RS7599493 -0.46 0.7 -0.198 -0.04 0.7 -1.535 -0.10 0.7

RS6716299 -0.46 0.7 -0.198 -0.04 0.7 -1.535 -0.10 0.7

RS12615150 -0.46 0.7 -0.198 -0.04 0.7 -1.536 -0.10 0.7

RS17025712 -0.46 0.7 -0.198 -0.04 0.7 -1.536 -0.10 0.7

RS717827 -0.46 0.7 -0.198 -0.04 0.7 -1.536 -0.10 0.7

RS10210665 -0.46 0.7 -0.198 -0.04 0.7 -1.536 -0.10 0.7

RS4952410 0.77 0.2 1.309 0.30 0.12 0.394 0.08 0.3

RS13017237 0.77 0.2 1.309 0.30 0.12 0.394 0.08 0.3

RS6737745 0.77 0.2 1.309 0.30 0.12 0.394 0.08 0.3

RS2373864 0.77 0.2 1.309 0.30 0.12 0.394 0.08 0.3

RS17026290 0.77 0.2 1.309 0.30 0.12 0.394 0.08 0.3

RS17026346 0.77 0.2 1.309 0.30 0.12 0.394 0.08 0.3

RS2045342 0.77 0.2 1.309 0.30 0.12 0.394 0.08 0.3

RS1013187 0.77 0.2 1.309 0.30 0.12 0.394 0.08 0.3

RS11898602 0.77 0.2 1.309 0.30 0.12 0.394 0.08 0.3

RS12989241 0.77 0.2 1.309 0.30 0.12 0.394 0.08 0.3

RS10199504 0.77 0.2 1.309 0.30 0.12 0.394 0.08 0.3

RS11687208 0.77 0.2 1.309 0.30 0.12 0.394 0.08 0.3

RS11886398 0.77 0.2 1.309 0.30 0.12 0.394 0.08 0.3

RS1441231 0.77 0.2 1.309 0.30 0.12 0.394 0.08 0.3

RS6544449 0.77 0.2 1.309 0.30 0.12 0.394 0.08 0.3

RS1978635 0.77 0.2 1.309 0.30 0.12 0.394 0.08 0.3

RS2374292 0.77 0.2 1.309 0.30 0.12 0.394 0.08 0.3

RS17028402 0.77 0.2 1.309 0.30 0.12 0.394 0.08 0.3

RS10167265 0.77 0.2 1.309 0.30 0.12 0.394 0.08 0.3

RS6731940 0.77 0.2 1.309 0.30 0.12 0.394 0.08 0.3

RS4500987 0.77 0.2 1.309 0.30 0.12 0.394 0.08 0.3

RS7570390 0.77 0.2 1.309 0.30 0.12 0.394 0.08 0.3

RS6737623 0.77 0.2 1.309 0.30 0.12 0.394 0.08 0.3

RS7570219 0.77 0.2 1.309 0.30 0.12 0.394 0.08 0.3

RS6733455 0.77 0.2 1.309 0.30 0.12 0.394 0.08 0.3

RS17029761 0.77 0.2 1.309 0.30 0.12 0.394 0.08 0.3

RS3821349 0.77 0.2 1.309 0.30 0.12 0.394 0.08 0.3

RS12613026 0.77 0.2 1.309 0.30 0.12 0.394 0.08 0.3

RS11899294 0.77 0.2 1.309 0.30 0.12 0.394 0.08 0.3

RS13396426 0.77 0.2 1.309 0.30 0.12 0.394 0.08 0.3

RS7593685 0.77 0.2 1.309 0.30 0.12 0.394 0.08 0.3

RS4485603 0.77 0.2 1.309 0.30 0.12 0.394 0.08 0.3

RS7578467 0.77 0.2 1.309 0.30 0.12 0.394 0.08 0.3

RS6718520 0.77 0.2 1.309 0.30 0.12 0.394 0.08 0.3

RS13026309 0.77 0.2 1.309 0.30 0.12 0.394 0.08 0.3

RS6544647 0.77 0.2 1.309 0.30 0.12 0.394 0.08 0.3

RS4952676 0.77 0.2 1.309 0.30 0.12 0.394 0.08 0.3

RS930194 0.77 0.2 1.309 0.30 0.12 0.394 0.08 0.3

RS3795860 0.77 0.2 1.309 0.30 0.12 0.394 0.08 0.3

RS4953045 0.77 0.2 1.309 0.30 0.12 0.394 0.08 0.3

RS17425341 0.77 0.2 1.309 0.30 0.12 0.394 0.08 0.3

RS4146528 0.77 0.2 1.309 0.30 0.12 0.394 0.08 0.3

RS698819 0.77 0.2 1.309 0.30 0.12 0.395 0.08 0.3

RS10201401 0.77 0.2 1.309 0.30 0.12 0.395 0.08 0.3

RS12620511 0.77 0.2 1.309 0.30 0.12 0.395 0.08 0.3

RS7565620 0.77 0.2 1.309 0.30 0.12 0.395 0.08 0.3

RS11674185 0.77 0.2 1.309 0.30 0.12 0.395 0.08 0.3

RS737447 0.77 0.2 1.309 0.30 0.12 0.395 0.08 0.3

RS6707760 0.77 0.2 1.309 0.30 0.12 0.395 0.08 0.3

RS1561227 0.77 0.2 1.309 0.30 0.12 0.395 0.08 0.3

RS1447107 0.77 0.2 1.309 0.30 0.12 0.394 0.08 0.3

RS908570 0.77 0.2 1.309 0.30 0.12 0.394 0.08 0.3

RS1517024 0.77 0.2 1.309 0.30 0.12 0.394 0.08 0.3

RS10209754 0.77 0.2 1.309 0.30 0.12 0.394 0.08 0.3

RS4952752 0.77 0.2 1.309 0.30 0.12 0.394 0.08 0.3

RS883413 0.77 0.2 1.309 0.30 0.12 0.394 0.08 0.3

RS17322370 0.77 0.2 1.309 0.30 0.12 0.395 0.08 0.3

RS2344662 0.77 0.2 1.309 0.30 0.12 0.395 0.08 0.3

RS6720975 0.77 0.2 1.309 0.30 0.12 0.394 0.08 0.3

RS4245804 0.77 0.2 1.309 0.30 0.12 0.394 0.08 0.3

RS6544868 0.77 0.2 1.309 0.30 0.12 0.394 0.08 0.3

RS12467673 0.77 0.2 1.309 0.30 0.12 0.394 0.08 0.3

RS1446317 0.77 0.2 1.309 0.30 0.12 0.394 0.08 0.3

RS4337520 0.77 0.2 1.309 0.30 0.12 0.394 0.08 0.3

RS7581090 0.77 0.2 1.309 0.30 0.12 0.394 0.08 0.3

RS6758592 0.77 0.2 1.309 0.30 0.12 0.394 0.08 0.3

RS2346177 0.77 0.2 1.309 0.30 0.12 0.394 0.08 0.3

RS2305350 0.77 0.2 1.309 0.30 0.12 0.394 0.08 0.3

RS10167561 0.77 0.2 1.309 0.30 0.12 0.394 0.08 0.3

RS9309137 0.77 0.2 1.309 0.30 0.12 0.394 0.08 0.3

RS17774013 0.77 0.2 1.309 0.30 0.12 0.394 0.08 0.3

RS7576915 0.77 0.2 1.309 0.30 0.12 0.394 0.08 0.3

RS7596354 0.77 0.2 1.309 0.30 0.12 0.394 0.08 0.3

RS4953485 0.77 0.2 1.309 0.30 0.12 0.394 0.08 0.3

RS11894871 0.77 0.2 1.309 0.30 0.12 0.394 0.08 0.3

RS7604511 0.77 0.2 1.309 0.30 0.12 0.394 0.08 0.3

RS4422207 0.77 0.2 1.309 0.30 0.12 0.394 0.08 0.3

RS17036537 0.77 0.2 1.309 0.30 0.12 0.394 0.08 0.3

RS13000537 0.77 0.2 1.309 0.30 0.12 0.394 0.08 0.3

RS4953503 0.77 0.2 1.309 0.30 0.12 0.394 0.08 0.3

RS4953509 0.77 0.2 1.309 0.30 0.12 0.394 0.08 0.3

RS7603494 0.77 0.2 1.309 0.30 0.12 0.394 0.08 0.3

RS3136228 0.77 0.2 1.309 0.30 0.12 0.394 0.08 0.3

RS900511 0.77 0.2 1.309 0.30 0.12 0.394 0.08 0.3

RS6545018 0.77 0.2 1.309 0.30 0.12 0.394 0.08 0.3

RS10191938 0.77 0.2 1.309 0.30 0.12 0.394 0.08 0.3

RS4430989 0.77 0.2 1.309 0.30 0.12 0.394 0.08 0.3

RS1464727 0.77 0.2 1.309 0.30 0.12 0.394 0.08 0.3

RS4352270 0.77 0.2 1.309 0.30 0.12 0.394 0.08 0.3

RS11897846 0.77 0.2 1.309 0.30 0.12 0.394 0.08 0.3

RS2301267 0.77 0.2 1.309 0.30 0.12 0.394 0.08 0.3

RS4538253 0.77 0.2 1.309 0.30 0.12 0.394 0.08 0.3

RS6545073 0.77 0.2 1.309 0.30 0.12 0.394 0.08 0.3

RS10202351 0.77 0.2 1.309 0.30 0.12 0.394 0.08 0.3

RS4953638 0.77 0.2 1.309 0.30 0.12 0.394 0.08 0.3

RS6165 0.77 0.2 1.309 0.30 0.12 0.394 0.08 0.3

RS1922466 0.77 0.2 1.309 0.30 0.12 0.394 0.08 0.3

RS12052611 0.77 0.2 1.309 0.30 0.12 0.394 0.08 0.3

RS13016730 0.77 0.2 1.309 0.30 0.12 0.394 0.08 0.3

RS9636436 0.77 0.2 1.309 0.30 0.12 0.394 0.08 0.3

RS7557485 0.77 0.2 1.309 0.30 0.12 0.394 0.08 0.3

RS17038751 0.77 0.2 1.309 0.30 0.12 0.394 0.08 0.3

RS1405959 0.77 0.2 1.309 0.30 0.12 0.394 0.08 0.3

RS4971860 0.77 0.2 1.309 0.30 0.12 0.394 0.08 0.3

RS13405761 0.77 0.2 1.309 0.30 0.12 0.394 0.08 0.3

RS17039201 0.77 0.2 1.309 0.30 0.12 0.394 0.08 0.3

RS11903512 0.77 0.2 1.309 0.30 0.12 0.394 0.08 0.3

RS17039577 0.77 0.2 1.309 0.30 0.12 0.394 0.08 0.3

RS6753833 0.77 0.2 1.309 0.30 0.12 0.394 0.08 0.3

RS4971552 0.77 0.2 1.309 0.30 0.12 0.394 0.08 0.3

RS6545153 0.77 0.2 1.309 0.30 0.12 0.394 0.08 0.3

RS1452786 0.77 0.2 1.309 0.30 0.12 0.394 0.08 0.3

RS12619144 0.77 0.2 1.309 0.30 0.12 0.394 0.08 0.3

RS1915234 0.77 0.2 1.309 0.30 0.12 0.394 0.08 0.3

RS9678669 0.77 0.2 1.309 0.30 0.12 0.394 0.08 0.3

RS7595040 0.77 0.2 1.309 0.30 0.12 0.394 0.08 0.3

RS6706713 0.77 0.2 1.309 0.30 0.12 0.394 0.08 0.3

RS2351767 3.17 0.0008 1.309 0.71 0.04 3.125 1.40 0.006

RS2216784 3.36 0.0004 1.309 0.73 0.03 3.125 1.43 0.005

RS10182729 3.68 0.00012 1.309 0.76 0.03 3.125 1.48 0.004

RS858951 4.10 0.00002 1.309 0.80 0.03 3.125 1.54 0.004

RS3850332 4.20 0.00001 1.309 0.81 0.03 3.125 1.56 0.004

RS17569028 4.23 0.00001 1.309 0.82 0.03 3.125 1.56 0.004

RS7559936 4.44 0.00000 1.309 0.83 0.03 3.125 1.59 0.003

RS11889192 4.46 0.00000 1.309 0.83 0.02 3.125 1.59 0.003

RS36016587 4.62 0.00000 1.309 0.85 0.02 3.125 1.61 0.003

RS984795 4.68 0.00000 1.309 0.85 0.02 3.125 1.61 0.003

RS2715053 4.68 0.00000 1.309 0.85 0.02 3.125 1.61 0.003

RS1528820 4.69 0.00000 1.309 0.85 0.02 3.125 1.61 0.003

RS12478948 4.69 0.00000 1.309 0.85 0.02 3.125 1.61 0.003

RS13405097 4.70 0.00000 1.309 0.85 0.02 3.125 1.61 0.003

RS7564650 4.70 0.00000 1.309 0.85 0.02 3.125 1.61 0.003

RS11125398 4.70 0.00000 1.309 0.85 0.02 3.125 1.61 0.003

RS2355803 4.70 0.00000 1.309 0.85 0.02 3.125 1.61 0.003

RS6723027 4.70 0.00000 1.309 0.85 0.02 3.125 1.61 0.003

RS11891667 4.70 0.00000 1.309 0.85 0.02 3.125 1.62 0.003

RS11895971 4.70 0.00000 1.309 0.85 0.02 3.125 1.62 0.003

RS4672216 4.70 0.00000 1.309 0.85 0.02 3.125 1.62 0.003

RS350779 4.70 0.00000 1.309 0.85 0.02 3.125 1.62 0.003

RS11897448 4.70 0.00000 1.309 0.85 0.02 3.125 1.62 0.003

RS2626385 4.70 0.00000 1.309 0.85 0.02 3.125 1.62 0.003

RS7577197 4.70 0.00000 1.309 0.85 0.02 3.125 1.62 0.003

RS9653536 4.70 0.00000 1.309 0.85 0.02 3.125 1.62 0.003

RS195592 4.70 0.00000 1.309 0.85 0.02 3.125 1.62 0.003

RS11125480 4.70 0.00000 1.309 0.85 0.02 3.125 1.62 0.003

RS11125482 4.70 0.00000 1.309 0.85 0.02 3.125 1.62 0.003

RS2194403 4.70 0.00000 1.309 0.85 0.02 3.125 1.62 0.003

RS10189098 4.70 0.00000 1.309 0.85 0.02 3.125 1.62 0.003

RS6705735 4.70 0.00000 1.309 0.85 0.02 3.126 1.62 0.003

RS10177230 4.70 0.00000 1.309 0.85 0.02 3.126 1.62 0.003

RS11681872 4.70 0.00000 1.309 0.85 0.02 3.126 1.62 0.003

RS7571459 4.70 0.00000 1.309 0.85 0.02 3.126 1.62 0.003

RS7601692 4.70 0.00000 1.309 0.85 0.02 3.126 1.62 0.003

RS10176342 4.70 0.00000 1.309 0.85 0.02 3.126 1.62 0.003

RS13417493 4.70 0.00000 1.309 0.85 0.02 3.126 1.62 0.003

RS843718 4.70 0.00000 1.309 0.85 0.02 3.126 1.62 0.003

RS7565034 4.70 0.00000 1.309 0.85 0.02 3.126 1.62 0.003

RS4306748 4.70 0.00000 1.309 0.85 0.02 3.126 1.62 0.003

RS6545432 4.70 0.00000 1.309 0.85 0.02 3.125 1.62 0.003

RS1510613 4.70 0.00000 1.309 0.85 0.02 3.125 1.62 0.003

RS6705079 4.70 0.00000 1.309 0.85 0.02 3.125 1.62 0.003

RS13422312 4.70 0.00000 1.309 0.85 0.02 3.125 1.62 0.003

RS6725139 4.70 0.00000 1.309 0.85 0.02 3.125 1.62 0.003

RS14026 4.54 0.00000 1.309 0.84 0.02 3.125 1.60 0.003

RS6545506 3.06 0.0011 1.309 0.70 0.04 3.125 1.36 0.006

RS6718634 1.51 0.07 1.309 0.47 0.07 3.121 0.75 0.03

RS891881 1.00 0.2 1.309 0.36 0.10 0.460 0.12 0.2

RS2023514 1.00 0.2 1.309 0.36 0.10 0.460 0.12 0.2

RS2111469 1.00 0.2 1.309 0.36 0.10 0.460 0.12 0.2

RS17047515 1.00 0.2 1.309 0.36 0.10 0.460 0.12 0.2

RS2869528 1.00 0.2 1.309 0.36 0.10 0.460 0.12 0.2

RS2869529 1.00 0.2 1.309 0.36 0.10 0.460 0.12 0.2

RS2193485 1.00 0.2 1.309 0.36 0.10 0.460 0.12 0.2

RS7576924 1.00 0.2 1.309 0.36 0.10 0.460 0.12 0.2

RS12469239 1.00 0.2 1.309 0.36 0.10 0.460 0.12 0.2

RS9808416 1.00 0.2 1.309 0.36 0.10 0.460 0.12 0.2

RS13423780 1.00 0.2 1.309 0.36 0.10 0.460 0.12 0.2

RS6545600 1.00 0.2 1.309 0.36 0.10 0.460 0.12 0.2

RS1582644 1.00 0.2 1.309 0.36 0.10 0.460 0.12 0.2

RS4290697 1.00 0.2 1.309 0.36 0.10 0.460 0.12 0.2

RS7603447 1.00 0.2 1.309 0.36 0.10 0.460 0.12 0.2

RS10210959 1.00 0.2 1.309 0.36 0.10 0.460 0.12 0.2

RS2030945 1.00 0.2 1.309 0.36 0.10 0.460 0.12 0.2

RS1568452 1.00 0.2 1.309 0.36 0.10 0.460 0.12 0.2

RS986512 1.00 0.2 1.309 0.36 0.10 0.460 0.12 0.2

RS11889211 1.00 0.2 1.309 0.36 0.10 0.460 0.12 0.2

RS6739403 1.00 0.2 1.309 0.36 0.10 0.460 0.12 0.2

RS6761469 1.00 0.2 1.309 0.36 0.10 0.460 0.12 0.2

RS11896571 1.00 0.2 1.309 0.36 0.10 0.460 0.12 0.2

RS2862733 1.00 0.2 1.309 0.36 0.10 0.460 0.12 0.2

RS1073975 1.00 0.2 1.309 0.36 0.10 0.460 0.12 0.2

RS7586333 1.00 0.2 1.309 0.36 0.10 0.460 0.12 0.2

RS7423968 1.00 0.2 1.309 0.36 0.10 0.460 0.12 0.2

RS12994597 1.00 0.2 1.309 0.36 0.10 0.460 0.12 0.2

RS6750614 1.00 0.2 1.309 0.36 0.10 0.460 0.12 0.2

RS13018470 1.00 0.2 1.309 0.36 0.10 0.460 0.12 0.2

RS10207525 1.00 0.2 1.309 0.36 0.10 0.460 0.12 0.2

RS17050204 1.00 0.2 1.309 0.36 0.10 0.460 0.12 0.2

RS6545742 1.00 0.2 1.309 0.36 0.10 0.460 0.12 0.2

RS1002343 1.00 0.2 1.309 0.36 0.10 0.460 0.12 0.2

RS11691352 1.00 0.2 1.309 0.36 0.10 0.460 0.12 0.2

RS4672315 1.00 0.2 1.309 0.36 0.10 0.460 0.12 0.2

RS929616 1.00 0.2 1.309 0.36 0.10 0.460 0.12 0.2

RS890067 1.00 0.2 1.309 0.36 0.10 0.460 0.12 0.2

RS994972 1.00 0.2 1.309 0.36 0.10 0.460 0.12 0.2

RS4671386 1.00 0.2 1.309 0.36 0.10 0.460 0.12 0.2

RS13408761 1.00 0.2 1.309 0.36 0.10 0.460 0.12 0.2

RS243035 1.00 0.2 1.309 0.36 0.10 0.460 0.12 0.2

RS243081 1.00 0.2 1.309 0.36 0.10 0.460 0.12 0.2

RS356991 1.00 0.2 1.309 0.36 0.10 0.460 0.12 0.2

RS971563 1.00 0.2 1.309 0.36 0.10 0.460 0.12 0.2

RS356999 1.00 0.2 1.309 0.36 0.10 0.460 0.12 0.2

RS13019590 1.00 0.2 1.309 0.36 0.10 0.460 0.12 0.2

RS1177283 1.05 0.15 1.309 0.38 0.09 0.475 0.13 0.2

RS13020758 1.17 0.12 1.309 0.40 0.09 0.504 0.16 0.2

RS10865326 1.17 0.12 1.309 0.40 0.09 0.504 0.16 0.2

RS2901428 1.17 0.12 1.309 0.40 0.09 0.504 0.16 0.2

RS7574498 1.17 0.12 1.309 0.40 0.09 0.504 0.16 0.2

RS10200191 1.17 0.12 1.309 0.40 0.09 0.504 0.16 0.2

RS3924400 1.17 0.12 1.309 0.40 0.09 0.504 0.16 0.2

RS7424968 1.17 0.12 1.309 0.40 0.09 0.504 0.16 0.2

RS6726083 1.17 0.12 1.309 0.40 0.09 0.504 0.16 0.2

RS2710647 1.17 0.12 1.309 0.40 0.09 0.504 0.16 0.2

RS12329383 1.17 0.12 1.309 0.40 0.09 0.504 0.16 0.2

RS6732746 1.17 0.12 1.309 0.40 0.09 0.504 0.16 0.2

RS2917793 1.17 0.12 1.309 0.40 0.09 0.504 0.16 0.2

RS11125991 1.17 0.12 1.309 0.40 0.09 0.504 0.16 0.2

RS6734073 1.17 0.12 1.309 0.40 0.09 0.504 0.16 0.2

RS2114430 1.17 0.12 1.309 0.40 0.09 0.504 0.16 0.2

RS4300812 1.17 0.12 1.309 0.40 0.09 0.504 0.16 0.2

RS13391261 1.17 0.12 1.309 0.40 0.09 0.504 0.16 0.2

RS888527 1.17 0.12 1.309 0.40 0.09 0.504 0.16 0.2

RS4671609 1.17 0.12 1.309 0.40 0.09 0.504 0.16 0.2

RS6546102 1.17 0.12 1.309 0.40 0.09 0.504 0.16 0.2

RS4296412 1.17 0.12 1.309 0.40 0.09 0.504 0.16 0.2

RS6546108 1.17 0.12 1.309 0.40 0.09 0.504 0.16 0.2

RS1035165 1.17 0.12 1.309 0.40 0.09 0.504 0.16 0.2

RS9677813 1.17 0.12 1.309 0.40 0.09 0.504 0.16 0.2

RS7556894 1.17 0.12 1.309 0.40 0.09 0.504 0.16 0.2

RS754110 1.17 0.12 1.309 0.40 0.09 0.504 0.16 0.2

RS840947 1.17 0.12 1.309 0.40 0.09 0.504 0.16 0.2

RS3845817 1.17 0.12 1.309 0.40 0.09 0.504 0.16 0.2

RS1673459 1.17 0.12 1.309 0.40 0.09 0.504 0.16 0.2

RS2860850 1.17 0.12 1.309 0.40 0.09 0.504 0.16 0.2

RS9678488 1.17 0.12 1.309 0.40 0.09 0.504 0.16 0.2

RS2111464 1.17 0.12 1.309 0.40 0.09 0.504 0.16 0.2

RS2631669 1.17 0.12 1.309 0.40 0.09 0.504 0.16 0.2

RS6546197 1.17 0.12 1.309 0.40 0.09 0.504 0.16 0.2

RS2699780 1.17 0.12 1.309 0.40 0.09 0.504 0.16 0.2

RS1000087 1.17 0.12 1.309 0.40 0.09 0.504 0.16 0.2

RS2951282 1.17 0.12 1.309 0.40 0.09 0.504 0.16 0.2

RS6716792 1.17 0.12 1.309 0.40 0.09 0.504 0.16 0.2

RS6730722 1.17 0.12 1.309 0.40 0.09 0.504 0.16 0.2

RS4671730 1.17 0.12 1.309 0.40 0.09 0.504 0.16 0.2

RS12987658 1.17 0.12 1.309 0.40 0.09 0.504 0.16 0.2

RS6705424 1.17 0.12 1.309 0.40 0.09 0.504 0.16 0.2

RS6546274 1.17 0.12 1.309 0.40 0.09 0.504 0.16 0.2

RS10197180 1.17 0.12 1.309 0.40 0.09 0.504 0.16 0.2

RS1548900 1.17 0.12 1.309 0.40 0.09 0.504 0.16 0.2

RS17032968 1.17 0.12 1.309 0.40 0.09 0.504 0.16 0.2

RS10180549 1.17 0.12 1.309 0.40 0.09 0.504 0.16 0.2

RS6754571 1.17 0.12 1.309 0.40 0.09 0.504 0.16 0.2

RS623457 1.17 0.12 1.309 0.40 0.09 0.504 0.16 0.2

RS6419640 1.17 0.12 1.309 0.40 0.09 0.504 0.16 0.2

RS1521043 1.17 0.12 1.309 0.40 0.09 0.504 0.16 0.2

RS17033681 1.17 0.12 1.309 0.40 0.09 0.504 0.16 0.2

RS1568471 1.17 0.12 1.309 0.40 0.09 0.504 0.16 0.2

RS13413974 1.17 0.12 1.309 0.40 0.09 0.504 0.16 0.2

RS10205913 1.17 0.12 1.309 0.40 0.09 0.504 0.16 0.2

RS10519124 1.17 0.12 1.309 0.40 0.09 0.504 0.16 0.2

RS12713602 1.17 0.12 1.309 0.40 0.09 0.504 0.16 0.2

RS12713604 1.17 0.12 1.309 0.40 0.09 0.504 0.16 0.2

RS4308115 1.17 0.12 1.309 0.40 0.09 0.504 0.16 0.2

RS13030887 1.17 0.12 1.309 0.40 0.09 0.504 0.16 0.2

RS6741698 1.17 0.12 1.309 0.40 0.09 0.504 0.16 0.2

RS1503241 1.17 0.12 1.309 0.40 0.09 0.504 0.16 0.2

RS1503237 1.17 0.12 1.309 0.40 0.09 0.504 0.16 0.2

RS4671202 1.17 0.12 1.309 0.40 0.09 0.504 0.16 0.2

RS13391405 1.17 0.12 1.309 0.40 0.09 0.504 0.16 0.2

RS6759864 1.17 0.12 1.309 0.40 0.09 0.504 0.16 0.2

RS10168414 1.17 0.12 1.309 0.40 0.09 0.504 0.16 0.2

RS6546420 1.17 0.12 1.309 0.40 0.09 0.504 0.16 0.2

RS6736893 1.17 0.12 1.309 0.40 0.09 0.504 0.16 0.2

RS4854436 1.17 0.12 1.309 0.40 0.09 0.504 0.16 0.2

RS4854515 1.17 0.12 1.309 0.40 0.09 0.504 0.16 0.2

RS2280310 1.17 0.12 1.309 0.40 0.09 0.504 0.16 0.2

RS1316845 1.17 0.12 1.309 0.40 0.09 0.504 0.16 0.2

RS10169464 1.17 0.12 1.309 0.40 0.09 0.504 0.16 0.2

RS4417751 1.17 0.12 1.309 0.40 0.09 0.504 0.16 0.2

RS4315565 1.17 0.12 1.309 0.40 0.09 0.504 0.16 0.2

RS4542888 1.17 0.12 1.309 0.40 0.09 0.504 0.16 0.2

RS10176087 1.17 0.12 1.309 0.40 0.09 0.504 0.16 0.2

RS6722492 1.17 0.12 1.309 0.40 0.09 0.504 0.16 0.2

RS10865380 1.17 0.12 1.309 0.40 0.09 0.504 0.16 0.2

RS6743318 1.17 0.12 1.309 0.40 0.09 0.504 0.16 0.2

RS2272532 1.17 0.12 1.309 0.40 0.09 0.504 0.16 0.2

RS1478644 1.17 0.12 1.309 0.40 0.09 0.504 0.16 0.2

RS10865381 2.73 0.003 1.309 0.66 0.04 3.124 1.26 0.008

RS418647 4.33 0.00001 1.309 0.82 0.03 3.125 1.57 0.004

RS2215021 4.70 0.00000 1.309 0.85 0.02 3.125 1.62 0.003

RS11126290 4.70 0.00000 1.309 0.85 0.02 3.125 1.62 0.003

RS17006285 4.70 0.00000 1.309 0.85 0.02 3.125 1.62 0.003

RS2287101 4.70 0.00000 1.309 0.85 0.02 3.125 1.62 0.003

RS1030046 4.70 0.00000 1.309 0.85 0.02 3.125 1.62 0.003

RS7584322 4.70 0.00000 1.309 0.85 0.02 3.125 1.62 0.003

RS10165187 4.70 0.00000 1.309 0.85 0.02 3.125 1.62 0.003

RS2239484 4.70 0.00000 1.309 0.85 0.02 3.125 1.62 0.003

RS2418891 4.70 0.00000 1.309 0.85 0.02 3.125 1.62 0.003

RS1458868 4.70 0.00000 1.309 0.85 0.02 3.125 1.62 0.003

RS12615297 1.04 0.15 1.309 0.37 0.10 0.470 0.13 0.2

RS12476972 1.04 0.15 1.309 0.37 0.10 0.470 0.13 0.2

RS10187354 1.04 0.15 1.309 0.37 0.10 0.470 0.13 0.2

RS4852792 1.04 0.15 1.309 0.37 0.10 0.470 0.13 0.2

RS11680124 1.04 0.15 1.309 0.37 0.10 0.470 0.13 0.2

RS1473228 1.04 0.15 1.309 0.37 0.10 0.470 0.13 0.2

RS227772 1.04 0.15 1.309 0.37 0.10 0.470 0.13 0.2

RS6749644 1.04 0.15 1.309 0.37 0.10 0.470 0.13 0.2

RS2419777 1.04 0.15 1.309 0.37 0.10 0.470 0.13 0.2

RS1471219 1.04 0.15 1.309 0.37 0.10 0.470 0.13 0.2

RS13425927 1.04 0.15 1.309 0.37 0.10 0.470 0.13 0.2

RS4852279 1.04 0.15 1.309 0.37 0.10 0.470 0.13 0.2

RS6750558 1.04 0.15 1.309 0.37 0.10 0.470 0.13 0.2

RS194242 1.04 0.15 1.309 0.37 0.10 0.470 0.13 0.2

RS17008015 1.04 0.15 1.309 0.37 0.10 0.470 0.13 0.2

RS11903912 1.04 0.15 1.309 0.37 0.10 0.470 0.13 0.2

RS1508060 1.04 0.15 1.309 0.37 0.10 0.470 0.13 0.2

RS1561245 1.04 0.15 1.309 0.37 0.10 0.470 0.13 0.2

RS10196456 1.04 0.15 1.309 0.37 0.10 0.470 0.13 0.2

RS9989755 1.04 0.15 1.309 0.37 0.10 0.470 0.13 0.2

RS12464589 1.04 0.15 1.309 0.37 0.10 0.470 0.13 0.2

RS1018342 1.04 0.15 1.309 0.37 0.10 0.470 0.13 0.2

RS1721244 1.04 0.15 1.309 0.37 0.10 0.470 0.13 0.2

RS1623605 1.04 0.15 1.309 0.37 0.10 0.470 0.13 0.2

RS7593050 1.04 0.15 1.309 0.37 0.10 0.470 0.13 0.2

RS1026150 1.04 0.15 1.309 0.37 0.10 0.470 0.13 0.2

RS828842 1.04 0.15 1.309 0.37 0.10 0.470 0.13 0.2

RS6723819 1.04 0.15 1.309 0.37 0.10 0.470 0.13 0.2

RS11678377 1.04 0.15 1.309 0.37 0.10 0.470 0.13 0.2

RS7574100 1.04 0.15 1.309 0.37 0.10 0.470 0.13 0.2

RS627152 1.04 0.15 1.309 0.37 0.10 0.470 0.13 0.2

RS3771766 1.04 0.15 1.309 0.37 0.10 0.470 0.13 0.2

RS10496197 1.04 0.15 1.309 0.37 0.10 0.470 0.13 0.2

RS7596427 1.04 0.15 1.309 0.37 0.10 0.470 0.13 0.2

RS12989728 1.04 0.15 1.309 0.37 0.10 0.470 0.13 0.2

RS8179569 1.04 0.15 1.309 0.37 0.10 0.470 0.13 0.2

RS2287102 1.04 0.15 1.309 0.37 0.10 0.470 0.13 0.2

RS3771823 1.04 0.15 1.309 0.37 0.10 0.470 0.13 0.2

RS4852364 1.04 0.15 1.309 0.37 0.10 0.470 0.13 0.2

RS12612891 1.04 0.15 1.309 0.37 0.10 0.470 0.13 0.2

RS10490307 1.04 0.15 1.309 0.37 0.10 0.470 0.13 0.2

RS10205827 1.04 0.15 1.309 0.37 0.10 0.470 0.13 0.2

RS1861429 1.04 0.15 1.309 0.37 0.10 0.470 0.13 0.2

RS13429274 1.04 0.15 1.309 0.37 0.10 0.470 0.13 0.2

RS7564504 1.04 0.15 1.309 0.37 0.10 0.470 0.13 0.2

RS12612199 1.04 0.15 1.309 0.37 0.10 0.470 0.13 0.2

RS2539992 1.04 0.15 1.309 0.37 0.10 0.470 0.13 0.2

RS7574273 1.04 0.15 1.309 0.37 0.10 0.470 0.13 0.2

RS917235 1.04 0.15 1.309 0.37 0.10 0.470 0.13 0.2

RS11675652 1.04 0.15 1.309 0.37 0.10 0.470 0.13 0.2

RS10179871 1.04 0.15 1.309 0.37 0.10 0.470 0.13 0.2

RS10194197 1.04 0.15 1.309 0.37 0.10 0.470 0.13 0.2

RS2583526 1.04 0.15 1.309 0.37 0.10 0.470 0.13 0.2

RS3843860 1.04 0.15 1.309 0.37 0.10 0.470 0.13 0.2

RS2860732 1.04 0.15 1.309 0.37 0.10 0.470 0.13 0.2

RS11126537 1.04 0.15 1.309 0.37 0.10 0.470 0.13 0.2

RS2141733 1.04 0.15 1.309 0.37 0.10 0.470 0.13 0.2

RS955949 1.04 0.15 1.309 0.37 0.10 0.470 0.13 0.2

RS10172954 1.04 0.15 1.309 0.37 0.10 0.470 0.13 0.2

RS971121 1.04 0.15 1.309 0.37 0.10 0.470 0.13 0.2

RS1470504 1.04 0.15 1.309 0.37 0.10 0.470 0.13 0.2

RS4853301 1.04 0.15 1.309 0.37 0.10 0.470 0.13 0.2

RS6705164 1.04 0.15 1.309 0.37 0.10 0.470 0.13 0.2

RS1354592 1.04 0.15 1.309 0.37 0.10 0.470 0.13 0.2

RS13403846 1.04 0.15 1.309 0.37 0.10 0.470 0.13 0.2

RS10206763 1.04 0.15 1.309 0.37 0.10 0.470 0.13 0.2

RS10865430 1.04 0.15 1.309 0.37 0.10 0.470 0.13 0.2

RS4485592 1.04 0.15 1.309 0.37 0.10 0.470 0.13 0.2

RS6707409 1.04 0.15 1.309 0.37 0.10 0.470 0.13 0.2

RS4513323 1.04 0.15 1.309 0.37 0.10 0.470 0.13 0.2

RS1487033 1.04 0.15 1.309 0.37 0.10 0.470 0.13 0.2

RS11676317 1.04 0.15 1.309 0.37 0.10 0.470 0.13 0.2

RS2129495 1.04 0.15 1.309 0.37 0.10 0.470 0.13 0.2

RS1586422 1.04 0.15 1.309 0.37 0.10 0.470 0.13 0.2

RS992870 1.04 0.15 1.309 0.37 0.10 0.470 0.13 0.2

RS6547205 1.04 0.15 1.309 0.37 0.10 0.470 0.13 0.2

RS1519624 1.04 0.15 1.309 0.37 0.10 0.470 0.13 0.2

RS1239084 1.04 0.15 1.309 0.37 0.10 0.470 0.13 0.2

RS1521973 1.04 0.15 1.309 0.37 0.10 0.470 0.13 0.2

RS10208925 1.04 0.15 1.309 0.37 0.10 0.470 0.13 0.2

RS10179963 1.04 0.15 1.309 0.37 0.10 0.470 0.13 0.2

RS364446 1.04 0.15 1.309 0.37 0.10 0.470 0.13 0.2

RS6710164 1.04 0.15 1.309 0.37 0.10 0.470 0.13 0.2

RS7588571 1.04 0.15 1.309 0.37 0.10 0.470 0.13 0.2

RS10779960 1.04 0.15 1.309 0.37 0.10 0.470 0.13 0.2

RS1434207 1.04 0.15 1.309 0.37 0.10 0.470 0.13 0.2

RS17705745 1.04 0.15 1.309 0.37 0.10 0.470 0.13 0.2

RS1584666 1.04 0.2 1.309 0.37 0.10 0.470 0.13 0.2

RS2100290 1.04 0.2 1.309 0.37 0.10 0.470 0.13 0.2

RS6716273 1.04 0.2 1.309 0.37 0.10 0.470 0.13 0.2

RS2861854 1.03 0.2 1.309 0.37 0.10 0.470 0.13 0.2

RS13028762 1.03 0.2 1.309 0.37 0.10 0.470 0.13 0.2

RS2974157 1.02 0.2 1.309 0.37 0.10 0.468 0.13 0.2

RS12472674 1.01 0.2 1.309 0.37 0.10 0.466 0.13 0.2

RS10170918 0.98 0.2 1.309 0.36 0.10 0.462 0.12 0.2

RS1484465 0.96 0.2 1.309 0.35 0.10 0.458 0.12 0.2

RS1867805 0.90 0.2 1.309 0.34 0.11 0.447 0.11 0.2

RS7604121 0.81 0.2 1.309 0.31 0.11 0.429 0.09 0.3

RS465119 0.70 0.2 1.309 0.28 0.13 0.405 0.08 0.3

RS6712681 0.57 0.3 1.309 0.24 0.15 0.366 0.06 0.3

RS11896302 0.10 0.5 1.309 0.05 0.3 0.104 0.00 0.5

RS1444543 -0.11 0.5 -0.198 -0.01 0.6 -0.136 -0.00 0.5

RS216640 -0.11 0.5 -0.198 -0.01 0.6 -0.136 -0.00 0.5

RS2131255 -0.11 0.5 -0.198 -0.01 0.6 -0.136 -0.00 0.5

RS6710603 -0.11 0.5 -0.198 -0.01 0.6 -0.136 -0.00 0.5

RS1982543 -0.11 0.5 -0.198 -0.01 0.6 -0.136 -0.00 0.5

RS1595071 -0.11 0.5 -0.198 -0.01 0.6 -0.136 -0.00 0.5

RS1347504 -0.11 0.5 -0.198 -0.01 0.6 -0.136 -0.00 0.5

RS11890114 -0.11 0.5 -0.198 -0.01 0.6 -0.136 -0.00 0.5

RS10188944 -0.11 0.5 -0.198 -0.01 0.6 -0.136 -0.00 0.5

RS7355329 -0.11 0.5 -0.198 -0.01 0.6 -0.136 -0.00 0.5

RS10167584 -0.12 0.5 -0.198 -0.01 0.6 -0.154 -0.00 0.6

RS1430726 -0.14 0.6 -0.198 -0.01 0.6 -0.183 -0.01 0.6

RS13011567 -0.16 0.6 -0.198 -0.01 0.6 -0.223 -0.01 0.6

RS17022447 -0.24 0.6 -0.198 -0.02 0.6 -0.400 -0.02 0.6

RS6547446 -0.25 0.6 -0.198 -0.02 0.6 -0.414 -0.02 0.6

RS12469609 -0.25 0.6 -0.198 -0.02 0.6 -0.414 -0.02 0.6

RS11126908 -0.25 0.6 -0.198 -0.02 0.6 -0.414 -0.02 0.6

RS2115619 -0.25 0.6 -0.198 -0.02 0.6 -0.414 -0.02 0.6

RS7590960 -0.25 0.6 -0.198 -0.02 0.6 -0.414 -0.02 0.6

RS7603217 -0.25 0.6 -0.198 -0.02 0.6 -0.414 -0.02 0.6

RS11126949 -0.25 0.6 -0.198 -0.02 0.6 -0.414 -0.02 0.6

RS13002679 -0.25 0.6 -0.198 -0.02 0.6 -0.414 -0.02 0.6

RS1365899 -0.25 0.6 -0.198 -0.02 0.6 -0.414 -0.02 0.6

RS7591327 -0.25 0.6 -0.198 -0.02 0.6 -0.414 -0.02 0.6

RS4832135 -0.25 0.6 -0.198 -0.02 0.6 -0.414 -0.02 0.6

RS11904105 -0.25 0.6 -0.198 -0.02 0.6 -0.414 -0.02 0.6

RS12714138 -0.25 0.6 -0.198 -0.02 0.6 -0.414 -0.02 0.6

RS4832176 -0.25 0.6 -0.198 -0.02 0.6 -0.414 -0.02 0.6

RS6714421 -0.25 0.6 -0.198 -0.02 0.6 -0.414 -0.02 0.6

RS7591175 -0.25 0.6 -0.198 -0.02 0.6 -0.414 -0.02 0.6

RS7316 -0.25 0.6 -0.198 -0.02 0.6 -0.414 -0.02 0.6

RS7355681 -0.25 0.6 -0.198 -0.02 0.6 -0.414 -0.02 0.6

RS4240202 -0.25 0.6 -0.198 -0.02 0.6 -0.414 -0.02 0.6

RS13430978 -0.25 0.6 -0.198 -0.02 0.6 -0.414 -0.02 0.6

RS13388951 -0.25 0.6 -0.198 -0.02 0.6 -0.414 -0.02 0.6

RS11690161 -0.25 0.6 -0.198 -0.02 0.6 -0.414 -0.02 0.6

RS11691934 -0.25 0.6 -0.198 -0.02 0.6 -0.414 -0.02 0.6

RS1863059 -0.25 0.6 -0.198 -0.02 0.6 -0.414 -0.02 0.6

RS308901 -0.25 0.6 -0.198 -0.02 0.6 -0.414 -0.02 0.6

RS2138397 -0.25 0.6 -0.198 -0.02 0.6 -0.414 -0.02 0.6

RS10191614 -0.25 0.6 -0.198 -0.02 0.6 -0.414 -0.02 0.6

RS28587600 -0.25 0.6 -0.198 -0.02 0.6 -0.414 -0.02 0.6

RS11684204 -0.25 0.6 -0.198 -0.02 0.6 -0.414 -0.02 0.6

RS6547750 -0.25 0.6 -0.198 -0.02 0.6 -0.414 -0.02 0.6

RS1978939 -0.25 0.6 -0.198 -0.02 0.6 -0.414 -0.02 0.6

RS1545224 -0.25 0.6 -0.198 -0.02 0.6 -0.414 -0.02 0.6

RS10865489 -0.25 0.6 -0.198 -0.02 0.6 -0.414 -0.02 0.6

RS11127090 -0.25 0.6 -0.198 -0.02 0.6 -0.414 -0.02 0.6

RS10211505 -0.25 0.6 -0.198 -0.02 0.6 -0.414 -0.02 0.6

RS12623469 -0.25 0.6 -0.198 -0.02 0.6 -0.414 -0.02 0.6

RS1016839 -0.25 0.6 -0.198 -0.02 0.6 -0.414 -0.02 0.6

RS10167879 -0.25 0.6 -0.198 -0.02 0.6 -0.414 -0.02 0.6

RS867529 -0.25 0.6 -0.198 -0.02 0.6 -0.414 -0.02 0.6

RS2195078 -0.25 0.6 -0.198 -0.02 0.6 -0.414 -0.02 0.6

RS7587312 -0.25 0.6 -0.198 -0.02 0.6 -0.414 -0.02 0.6

RS6745441 -0.25 0.6 -0.198 -0.02 0.6 -0.414 -0.02 0.6

RS2579505 -0.25 0.6 -0.198 -0.02 0.6 -0.414 -0.02 0.6

RS10874468 -0.25 0.6 -0.198 -0.02 0.6 -0.414 -0.02 0.6

RS298904 -0.25 0.6 -0.198 -0.02 0.6 -0.414 -0.02 0.6

RS17426403 -0.25 0.6 -0.198 -0.02 0.6 -0.414 -0.02 0.6

RS4851839 -0.25 0.6 -0.198 -0.02 0.6 -0.414 -0.02 0.6

RS7578035 -0.25 0.6 -0.198 -0.02 0.6 -0.414 -0.02 0.6

RS11695379 -0.25 0.6 -0.198 -0.02 0.6 -0.414 -0.02 0.6

RS717454 -0.25 0.6 -0.198 -0.02 0.6 -0.414 -0.02 0.6

RS10183501 -0.25 0.6 -0.198 -0.02 0.6 -0.414 -0.02 0.6

RS11675333 -0.25 0.6 -0.198 -0.02 0.6 -0.414 -0.02 0.6

RS12712066 -0.25 0.6 -0.198 -0.02 0.6 -0.414 -0.02 0.6

RS13394110 -0.25 0.6 -0.198 -0.02 0.6 -0.414 -0.02 0.6

RS4438499 -0.25 0.6 -0.198 -0.02 0.6 -0.414 -0.02 0.6

RS6542950 -0.25 0.6 -0.198 -0.02 0.6 -0.414 -0.02 0.6

RS2176733 -0.25 0.6 -0.198 -0.02 0.6 -0.414 -0.02 0.6

RS7594990 -0.25 0.6 -0.198 -0.02 0.6 -0.414 -0.02 0.6

RS7570854 -0.25 0.6 -0.198 -0.02 0.6 -0.414 -0.02 0.6

RS1028045 -0.25 0.6 -0.198 -0.02 0.6 -0.414 -0.02 0.6

RS356643 -0.25 0.6 -0.198 -0.02 0.6 -0.414 -0.02 0.6

RS895522 -0.25 0.6 -0.198 -0.02 0.6 -0.414 -0.02 0.6

RS11123859 -0.25 0.6 -0.198 -0.02 0.6 -0.414 -0.02 0.6

RS4851398 -0.25 0.6 -0.198 -0.02 0.6 -0.414 -0.02 0.6

RS4850962 -0.25 0.6 -0.198 -0.02 0.6 -0.414 -0.02 0.6

RS1192827 -0.25 0.6 -0.198 -0.02 0.6 -0.414 -0.02 0.6

RS4851416 -0.25 0.6 -0.198 -0.02 0.6 -0.414 -0.02 0.6

RS6732292 -0.25 0.6 -0.198 -0.02 0.6 -0.414 -0.02 0.6

RS11123891 -0.25 0.6 -0.198 -0.02 0.6 -0.414 -0.02 0.6

RS3900546 -0.25 0.6 -0.198 -0.02 0.6 -0.414 -0.02 0.6

RS7603851 -0.25 0.6 -0.198 -0.02 0.6 -0.414 -0.02 0.6

RS4851475 -0.25 0.6 -0.198 -0.02 0.6 -0.414 -0.02 0.6

RS11123901 -0.25 0.6 -0.198 -0.02 0.6 -0.414 -0.02 0.6

RS4851522 -0.25 0.6 -0.198 -0.02 0.6 -0.414 -0.02 0.6

RS2072474 -0.25 0.6 -0.198 -0.02 0.6 -0.414 -0.02 0.6

RS868251 -0.25 0.6 -0.198 -0.02 0.6 -0.414 -0.02 0.6

RS3771188 -0.25 0.6 -0.198 -0.02 0.6 -0.414 -0.02 0.6

RS1035131 -0.25 0.6 -0.198 -0.02 0.6 -0.414 -0.02 0.6

RS6761291 -0.25 0.6 -0.198 -0.02 0.6 -0.414 -0.02 0.6

RS11681159 -0.25 0.6 -0.198 -0.02 0.6 -0.414 -0.02 0.6

RS7608617 -0.25 0.6 -0.198 -0.02 0.6 -0.414 -0.02 0.6

RS1052431 -0.25 0.6 -0.198 -0.02 0.6 -0.414 -0.02 0.6

RS2732822 -0.25 0.6 -0.198 -0.02 0.6 -0.414 -0.02 0.6

RS7602289 -0.25 0.6 -0.198 -0.02 0.6 -0.414 -0.02 0.6

RS6543194 -0.25 0.6 -0.198 -0.02 0.6 -0.414 -0.02 0.6

RS17028232 -0.25 0.6 -0.198 -0.02 0.6 -0.414 -0.02 0.6

RS6709515 -0.25 0.6 -0.198 -0.02 0.6 -0.414 -0.02 0.6

RS1728260 -0.25 0.6 -0.198 -0.02 0.6 -0.414 -0.02 0.6

RS10496364 -0.25 0.6 -0.198 -0.02 0.6 -0.414 -0.02 0.6

RS1550533 -0.25 0.6 -0.198 -0.02 0.6 -0.414 -0.02 0.6

RS2610681 -0.25 0.6 -0.198 -0.02 0.6 -0.414 -0.02 0.6

RS2570481 -0.25 0.6 -0.198 -0.02 0.6 -0.414 -0.02 0.6

RS7581688 -0.25 0.6 -0.198 -0.02 0.6 -0.414 -0.02 0.6

RS4552208 -0.25 0.6 -0.198 -0.02 0.6 -0.414 -0.02 0.6

RS6743263 -0.25 0.6 -0.198 -0.02 0.6 -0.414 -0.02 0.6

RS11123995 -0.25 0.6 -0.198 -0.02 0.6 -0.414 -0.02 0.6

RS1866558 -0.25 0.6 -0.198 -0.02 0.6 -0.414 -0.02 0.6

RS6543262 -0.25 0.6 -0.198 -0.02 0.6 -0.414 -0.02 0.6

RS444636 -0.25 0.6 -0.198 -0.02 0.6 -0.414 -0.02 0.6

RS3739154 -0.25 0.6 -0.198 -0.02 0.6 -0.414 -0.02 0.6

RS4605389 -0.25 0.6 -0.198 -0.02 0.6 -0.414 -0.02 0.6

RS2679851 -0.25 0.6 -0.198 -0.02 0.6 -0.414 -0.02 0.6

RS7584136 -0.25 0.6 -0.198 -0.02 0.6 -0.414 -0.02 0.6

RS2241797 -0.25 0.6 -0.198 -0.02 0.6 -0.414 -0.02 0.6

RS2164718 -0.25 0.6 -0.198 -0.02 0.6 -0.414 -0.02 0.6

RS12712206 -0.25 0.6 -0.198 -0.02 0.6 -0.414 -0.02 0.6

RS7608329 -0.25 0.6 -0.198 -0.02 0.6 -0.414 -0.02 0.6

RS17031046 -0.25 0.6 -0.198 -0.02 0.6 -0.414 -0.02 0.6

RS7563743 -0.25 0.6 -0.198 -0.02 0.6 -0.414 -0.02 0.6

RS4851089 -0.25 0.6 -0.198 -0.02 0.6 -0.414 -0.02 0.6

RS7602442 -0.25 0.6 -0.198 -0.02 0.6 -0.414 -0.02 0.6

RS1111513 -0.25 0.6 -0.198 -0.02 0.6 -0.414 -0.02 0.6

RS2377442 -0.25 0.6 -0.198 -0.02 0.6 -0.414 -0.02 0.6

RS12465304 -0.25 0.6 -0.198 -0.02 0.6 -0.414 -0.02 0.6

RS1901460 -0.25 0.6 -0.198 -0.02 0.6 -0.414 -0.02 0.6

RS12712247 -0.25 0.6 -0.198 -0.02 0.6 -0.414 -0.02 0.6

RS11673736 -0.25 0.6 -0.198 -0.02 0.6 -0.414 -0.02 0.6

RS6543408 -0.25 0.6 -0.198 -0.02 0.6 -0.414 -0.02 0.6

RS7575343 -0.25 0.6 -0.198 -0.02 0.6 -0.414 -0.02 0.6

RS6704791 -0.25 0.6 -0.198 -0.02 0.6 -0.414 -0.02 0.6

RS1375000 -0.25 0.6 -0.198 -0.02 0.6 -0.414 -0.02 0.6

RS11679344 -0.25 0.6 -0.198 -0.02 0.6 -0.414 -0.02 0.6

RS11681787 -0.25 0.6 -0.198 -0.02 0.6 -0.414 -0.02 0.6

RS6729112 -0.25 0.6 -0.198 -0.02 0.6 -0.414 -0.02 0.6

RS2906607 -0.25 0.6 -0.198 -0.02 0.6 -0.414 -0.02 0.6

RS1990409 -0.25 0.6 -0.198 -0.02 0.6 -0.414 -0.02 0.6

RS6744712 -0.25 0.6 -0.198 -0.02 0.6 -0.414 -0.02 0.6

RS758822 -0.25 0.6 -0.198 -0.02 0.6 -0.414 -0.02 0.6

RS6746088 -0.25 0.6 -0.198 -0.02 0.6 -0.414 -0.02 0.6

RS10184114 -0.25 0.6 -0.198 -0.02 0.6 -0.414 -0.02 0.6

RS6753886 -0.25 0.6 -0.198 -0.02 0.6 -0.414 -0.02 0.6

RS10865017 -0.25 0.6 -0.198 -0.02 0.6 -0.414 -0.02 0.6

RS17036061 -0.25 0.6 -0.198 -0.02 0.6 -0.414 -0.02 0.6

RS17036146 -0.25 0.6 -0.198 -0.02 0.6 -0.414 -0.02 0.6

RS1478516 -0.25 0.6 -0.198 -0.02 0.6 -0.414 -0.02 0.6

RS260651 -0.25 0.6 -0.198 -0.02 0.6 -0.414 -0.02 0.6

RS11676676 -0.25 0.6 -0.198 -0.02 0.6 -0.414 -0.02 0.6

RS10190545 -0.25 0.6 -0.198 -0.02 0.6 -0.414 -0.02 0.6

RS7605559 -0.25 0.6 -0.198 -0.02 0.6 -0.414 -0.02 0.6

RS11240790 -0.25 0.6 -0.198 -0.02 0.6 -0.414 -0.02 0.6

RS13417566 -0.25 0.6 -0.198 -0.02 0.6 -0.414 -0.02 0.6

RS3789090 -0.25 0.6 -0.198 -0.02 0.6 -0.414 -0.02 0.6

RS13012948 -0.25 0.6 -0.198 -0.02 0.6 -0.414 -0.02 0.6

RS9308731 -0.25 0.6 -0.198 -0.02 0.6 -0.414 -0.02 0.6

RS13411196 -0.25 0.6 -0.198 -0.02 0.6 -0.414 -0.02 0.6

RS1463118 -0.25 0.6 -0.198 -0.02 0.6 -0.414 -0.02 0.6

RS11123551 -0.25 0.6 -0.198 -0.02 0.6 -0.414 -0.02 0.6

RS12328538 -0.25 0.6 -0.198 -0.02 0.6 -0.414 -0.02 0.6

RS12612210 -0.25 0.6 -0.198 -0.02 0.6 -0.414 -0.02 0.6

RS10174353 -0.25 0.6 -0.198 -0.02 0.6 -0.414 -0.02 0.6

RS1400323 -0.25 0.6 -0.198 -0.02 0.6 -0.414 -0.02 0.6

RS10200201 -0.25 0.6 -0.198 -0.02 0.6 -0.414 -0.02 0.6

RS6542045 -0.25 0.6 -0.198 -0.02 0.6 -0.414 -0.02 0.6

RS4849091 -0.25 0.6 -0.198 -0.02 0.6 -0.414 -0.02 0.6

RS17042407 -0.25 0.6 -0.198 -0.02 0.6 -0.414 -0.02 0.6

RS1143627 -0.25 0.6 -0.198 -0.02 0.6 -0.414 -0.02 0.6

RS11692384 -0.25 0.6 -0.198 -0.02 0.6 -0.414 -0.02 0.6

RS28928309 -0.25 0.6 -0.198 -0.02 0.6 -0.414 -0.02 0.6

RS3791336 -0.25 0.6 -0.198 -0.02 0.6 -0.414 -0.02 0.6

RS6755077 -0.25 0.6 -0.198 -0.02 0.6 -0.414 -0.02 0.6

RS4848323 -0.25 0.6 -0.198 -0.02 0.6 -0.414 -0.02 0.6

RS4849195 -0.25 0.6 -0.198 -0.02 0.6 -0.414 -0.02 0.6

RS2121243 -0.25 0.6 -0.198 -0.02 0.6 -0.414 -0.02 0.6

RS2418915 -0.25 0.6 -0.198 -0.02 0.6 -0.414 -0.02 0.6

RS6708711 -0.25 0.6 -0.198 -0.02 0.6 -0.414 -0.02 0.6

RS10208204 -0.25 0.6 -0.198 -0.02 0.6 -0.414 -0.02 0.6

RS7577695 -0.25 0.6 -0.198 -0.02 0.6 -0.414 -0.02 0.6

RS6733541 -0.25 0.6 -0.198 -0.02 0.6 -0.414 -0.02 0.6

RS3132058 -0.25 0.6 -0.198 -0.02 0.6 -0.414 -0.02 0.6

RS1430094 -0.25 0.6 -0.198 -0.02 0.6 -0.414 -0.02 0.6

RS1430090 -0.25 0.6 -0.198 -0.02 0.6 -0.414 -0.02 0.6

RS6542214 -0.25 0.6 -0.198 -0.02 0.6 -0.414 -0.02 0.6

RS7606549 -0.25 0.6 -0.198 -0.02 0.6 -0.414 -0.02 0.6

RS7597658 -0.25 0.6 -0.198 -0.02 0.6 -0.414 -0.02 0.6

RS4353658 -0.25 0.6 -0.198 -0.02 0.6 -0.414 -0.02 0.6

RS1396933 -0.25 0.6 -0.198 -0.02 0.6 -0.414 -0.02 0.6

RS12991665 -0.25 0.6 -0.198 -0.02 0.6 -0.414 -0.02 0.6

RS7570085 -0.25 0.6 -0.198 -0.02 0.6 -0.414 -0.02 0.6

RS7569451 -0.25 0.6 -0.198 -0.02 0.6 -0.414 -0.02 0.6

RS13387046 -0.25 0.6 -0.198 -0.02 0.6 -0.414 -0.02 0.6

RS6542299 -0.25 0.6 -0.198 -0.02 0.6 -0.414 -0.02 0.6

RS7604612 -0.25 0.6 -0.198 -0.02 0.6 -0.414 -0.02 0.6

RS7594984 -0.25 0.6 -0.198 -0.02 0.6 -0.414 -0.02 0.6

RS1515126 -0.25 0.6 -0.198 -0.02 0.6 -0.414 -0.02 0.6

RS4848430 -0.25 0.6 -0.198 -0.02 0.6 -0.414 -0.02 0.6

RS2615828 -0.25 0.6 -0.198 -0.02 0.6 -0.414 -0.02 0.6

RS17046973 -0.25 0.6 -0.198 -0.02 0.6 -0.414 -0.02 0.6

RS17047157 -0.25 0.6 -0.198 -0.02 0.6 -0.414 -0.02 0.6

RS17498503 -0.25 0.6 -0.198 -0.02 0.6 -0.414 -0.02 0.6

RS6728975 -0.25 0.6 -0.198 -0.02 0.6 -0.414 -0.02 0.6

RS17512698 -0.25 0.6 -0.198 -0.02 0.6 -0.414 -0.02 0.6

RS12616545 -0.25 0.6 -0.198 -0.02 0.6 -0.414 -0.02 0.6

RS4276037 -0.25 0.6 -0.198 -0.02 0.6 -0.414 -0.02 0.6

RS10192071 -0.25 0.6 -0.198 -0.02 0.6 -0.414 -0.02 0.6

RS6704674 -0.25 0.6 -0.198 -0.02 0.6 -0.414 -0.02 0.6

RS1437683 -0.25 0.6 -0.198 -0.02 0.6 -0.414 -0.02 0.6

RS6736836 -0.25 0.6 -0.198 -0.02 0.6 -0.414 -0.02 0.6

RS332675 -0.25 0.6 -0.198 -0.02 0.6 -0.414 -0.02 0.6

RS4849721 -0.25 0.6 -0.198 -0.02 0.6 -0.414 -0.02 0.6

RS11694004 -0.25 0.6 -0.198 -0.02 0.6 -0.414 -0.02 0.6

RS3754855 -0.25 0.6 -0.198 -0.02 0.6 -0.414 -0.02 0.6

RS4848530 -0.25 0.6 -0.198 -0.02 0.6 -0.414 -0.02 0.6

RS2422460 -0.25 0.6 -0.198 -0.02 0.6 -0.414 -0.02 0.6

RS893769 -0.25 0.6 -0.198 -0.02 0.6 -0.414 -0.02 0.6

RS12711924 -0.25 0.6 -0.198 -0.02 0.6 -0.414 -0.02 0.6

RS6711445 -0.25 0.6 -0.198 -0.02 0.6 -0.414 -0.02 0.6

RS11693629 -0.25 0.6 -0.198 -0.02 0.6 -0.414 -0.02 0.6

RS11123523 -0.25 0.6 -0.198 -0.02 0.6 -0.414 -0.02 0.6

RS2579612 -0.25 0.6 -0.198 -0.02 0.6 -0.414 -0.02 0.6

RS4383344 -0.25 0.6 -0.198 -0.02 0.6 -0.414 -0.02 0.6

RS17049444 -0.25 0.6 -0.198 -0.02 0.6 -0.414 -0.02 0.6

RS6542545 -0.25 0.6 -0.198 -0.02 0.6 -0.414 -0.02 0.6

RS11673874 -0.25 0.6 -0.198 -0.02 0.6 -0.414 -0.02 0.6

RS13018516 -0.25 0.6 -0.198 -0.02 0.6 -0.414 -0.02 0.6

RS7589138 -0.25 0.6 -0.198 -0.02 0.6 -0.414 -0.02 0.6

RS1548039 -0.25 0.6 -0.198 -0.02 0.6 -0.414 -0.02 0.6

RS12711941 -0.25 0.6 -0.198 -0.02 0.6 -0.414 -0.02 0.6

RS1869026 -0.25 0.6 -0.198 -0.02 0.6 -0.414 -0.02 0.6

RS4848094 -0.25 0.6 -0.198 -0.02 0.6 -0.414 -0.02 0.6

RS707482 -0.25 0.6 -0.198 -0.02 0.6 -0.414 -0.02 0.6

RS2053989 -0.25 0.6 -0.198 -0.02 0.6 -0.414 -0.02 0.6

RS895485 -0.25 0.6 -0.198 -0.02 0.6 -0.414 -0.02 0.6

RS4848641 -0.25 0.6 -0.198 -0.02 0.6 -0.414 -0.02 0.6

RS4848126 -0.25 0.6 -0.198 -0.02 0.6 -0.414 -0.02 0.6

RS4271771 -0.38 0.6 -0.198 -0.03 0.6 -1.005 -0.06 0.7

RS17006117 -0.40 0.7 -0.198 -0.03 0.7 -1.099 -0.07 0.7

RS2002067 -0.40 0.7 -0.198 -0.03 0.7 -1.099 -0.07 0.7

RS2304667 -0.40 0.7 -0.198 -0.03 0.7 -1.099 -0.07 0.7

RS2250092 -0.40 0.7 -0.198 -0.03 0.7 -1.099 -0.07 0.7

RS2580352 -0.40 0.7 -0.198 -0.03 0.7 -1.099 -0.07 0.7

RS12479320 -0.40 0.7 -0.198 -0.03 0.7 -1.099 -0.07 0.7

RS4522608 -0.40 0.7 -0.198 -0.03 0.7 -1.099 -0.07 0.7

RS6541804 -0.40 0.7 -0.198 -0.03 0.7 -1.099 -0.07 0.7

RS4316942 -0.40 0.7 -0.198 -0.03 0.7 -1.099 -0.07 0.7

RS11889169 -0.40 0.7 -0.198 -0.03 0.7 -1.099 -0.07 0.7

RS11690406 -0.40 0.7 -0.198 -0.03 0.7 -1.099 -0.07 0.7

RS6735231 -0.40 0.7 -0.198 -0.03 0.7 -1.099 -0.07 0.7

RS1881881 -0.40 0.7 -0.198 -0.03 0.7 -1.099 -0.07 0.7

RS11122903 -0.40 0.7 -0.198 -0.03 0.7 -1.099 -0.07 0.7

RS6541829 -0.40 0.7 -0.198 -0.03 0.7 -1.099 -0.07 0.7

RS896902 -0.40 0.7 -0.198 -0.03 0.7 -1.099 -0.07 0.7

RS17008320 -0.40 0.7 -0.198 -0.03 0.7 -1.099 -0.07 0.7

RS17008648 -0.40 0.7 -0.198 -0.03 0.7 -1.099 -0.07 0.7

RS297470 -0.40 0.7 -0.198 -0.03 0.7 -1.099 -0.07 0.7

RS17009204 -0.40 0.7 -0.198 -0.03 0.7 -1.099 -0.07 0.7

RS7571273 -0.40 0.7 -0.198 -0.03 0.7 -1.099 -0.07 0.7

RS12477571 -0.40 0.7 -0.198 -0.03 0.7 -1.099 -0.07 0.7

RS993934 -0.40 0.7 -0.198 -0.03 0.7 -1.099 -0.07 0.7

RS11904509 -0.40 0.7 -0.198 -0.03 0.7 -1.099 -0.07 0.7

RS2670616 -0.40 0.7 -0.198 -0.03 0.7 -1.099 -0.07 0.7

RS7565459 -0.40 0.7 -0.198 -0.03 0.7 -1.099 -0.07 0.7

RS10496618 -0.40 0.7 -0.198 -0.03 0.7 -1.099 -0.07 0.7

RS925796 -0.40 0.7 -0.198 -0.03 0.7 -1.099 -0.07 0.7

RS6760710 -0.40 0.7 -0.198 -0.03 0.7 -1.099 -0.07 0.7

RS17010809 -0.40 0.7 -0.198 -0.03 0.7 -1.099 -0.07 0.7

RS1919839 -0.40 0.7 -0.198 -0.03 0.7 -1.099 -0.07 0.7

RS779985 -0.40 0.7 -0.198 -0.03 0.7 -1.099 -0.07 0.7

RS884376 -0.40 0.7 -0.198 -0.03 0.7 -1.099 -0.07 0.7

RS6541961 -0.40 0.7 -0.198 -0.03 0.7 -1.099 -0.07 0.7

RS10445861 -0.40 0.7 -0.198 -0.03 0.7 -1.099 -0.07 0.7

RS13021629 -0.40 0.7 -0.198 -0.03 0.7 -1.099 -0.07 0.7

RS2168024 -0.40 0.7 -0.198 -0.03 0.7 -1.099 -0.07 0.7

RS922862 -0.40 0.7 -0.198 -0.03 0.7 -1.099 -0.07 0.7

RS314721 -0.40 0.7 -0.198 -0.03 0.7 -1.099 -0.07 0.7

RS6733261 -0.40 0.7 -0.198 -0.03 0.7 -1.099 -0.07 0.7

RS314946 -0.40 0.7 -0.198 -0.03 0.7 -1.099 -0.07 0.7

RS313295 -0.40 0.7 -0.198 -0.03 0.7 -1.099 -0.07 0.7

RS1377122 -0.52 0.7 -0.198 -0.04 0.7 -2.334 -0.15 0.8

RS11675893 -0.52 0.7 -0.198 -0.04 0.7 -2.334 -0.15 0.8

RS1452515 -0.52 0.7 -0.198 -0.04 0.7 -2.334 -0.15 0.8

RS1452512 -0.52 0.7 -0.198 -0.04 0.7 -2.334 -0.15 0.8

RS2052815 -0.52 0.7 -0.198 -0.04 0.7 -2.334 -0.15 0.8

RS3962035 -0.52 0.7 -0.198 -0.04 0.7 -2.334 -0.15 0.8

RS7589808 -0.52 0.7 -0.198 -0.04 0.7 -2.334 -0.15 0.8

RS17013262 -0.52 0.7 -0.198 -0.04 0.7 -2.334 -0.15 0.8

RS11687421 -0.52 0.7 -0.198 -0.04 0.7 -2.334 -0.15 0.8

RS17013442 -0.52 0.7 -0.198 -0.04 0.7 -2.334 -0.15 0.8

RS7565286 -0.42 0.7 -0.198 -0.03 0.7 -1.220 -0.07 0.7

RS6714079 -0.40 0.7 -0.198 -0.03 0.7 -1.079 -0.06 0.7

RS1899038 -0.40 0.7 -0.198 -0.03 0.7 -1.078 -0.06 0.7

RS6431169 -0.39 0.7 -0.198 -0.03 0.7 -1.045 -0.06 0.7

RS4663038 -0.39 0.7 -0.198 -0.03 0.7 -1.045 -0.06 0.7

RS6732528 -0.39 0.7 -0.198 -0.03 0.7 -1.045 -0.06 0.7

RS2404962 -0.39 0.7 -0.198 -0.03 0.7 -1.045 -0.06 0.7

RS1914458 -0.39 0.7 -0.198 -0.03 0.7 -1.045 -0.06 0.7

RS10178199 -0.39 0.7 -0.198 -0.03 0.7 -1.045 -0.06 0.7

RS754737 -0.39 0.7 -0.198 -0.03 0.7 -1.045 -0.06 0.7

RS7573824 -0.39 0.7 -0.198 -0.03 0.7 -1.045 -0.06 0.7

RS6713701 -0.39 0.7 -0.198 -0.03 0.7 -1.045 -0.06 0.7

RS10194375 -0.39 0.7 -0.198 -0.03 0.7 -1.045 -0.06 0.7

RS749008 -0.39 0.7 -0.198 -0.03 0.7 -1.045 -0.06 0.7

RS6751498 -0.39 0.7 -0.198 -0.03 0.7 -1.045 -0.06 0.7

RS4663101 -0.39 0.7 -0.198 -0.03 0.7 -1.045 -0.06 0.7

RS2896885 -0.39 0.7 -0.198 -0.03 0.7 -1.045 -0.06 0.7

RS11682330 -0.39 0.7 -0.198 -0.03 0.7 -1.045 -0.06 0.7

RS10928772 -0.39 0.7 -0.198 -0.03 0.7 -1.045 -0.06 0.7

RS17600636 -0.39 0.7 -0.198 -0.03 0.7 -1.045 -0.06 0.7

RS17015719 -0.39 0.7 -0.198 -0.03 0.7 -1.045 -0.06 0.7

RS6430975 -0.39 0.7 -0.198 -0.03 0.7 -1.045 -0.06 0.7

RS1699 -0.39 0.7 -0.198 -0.03 0.7 -1.045 -0.06 0.7

RS13384737 -0.39 0.7 -0.198 -0.03 0.7 -1.045 -0.06 0.7

RS6756336 -0.39 0.7 -0.198 -0.03 0.7 -1.045 -0.06 0.7

RS4662791 -0.39 0.7 -0.198 -0.03 0.7 -1.045 -0.06 0.7

RS10048789 -0.39 0.7 -0.198 -0.03 0.7 -1.045 -0.06 0.7

RS714129 -0.39 0.7 -0.198 -0.03 0.7 -1.045 -0.06 0.7

RS4662800 -0.39 0.7 -0.198 -0.03 0.7 -1.045 -0.06 0.7

RS7564226 -0.39 0.7 -0.198 -0.03 0.7 -1.045 -0.06 0.7

RS841470 -0.39 0.7 -0.198 -0.03 0.7 -1.045 -0.06 0.7

RS10928827 -0.39 0.7 -0.198 -0.03 0.7 -1.045 -0.06 0.7

RS11684311 -0.39 0.7 -0.198 -0.03 0.7 -1.045 -0.06 0.7

RS17048766 -0.39 0.7 -0.198 -0.03 0.7 -1.045 -0.06 0.7

RS1344242 -0.39 0.7 -0.198 -0.03 0.7 -1.045 -0.06 0.7

RS12478682 -0.39 0.7 -0.198 -0.03 0.7 -1.045 -0.06 0.7

RS908848 -0.39 0.7 -0.198 -0.03 0.7 -1.045 -0.06 0.7

RS4662910 -0.39 0.7 -0.198 -0.03 0.7 -1.045 -0.06 0.7

RS13387772 -0.39 0.7 -0.198 -0.03 0.7 -1.045 -0.06 0.7

RS1453147 -0.39 0.7 -0.198 -0.03 0.7 -1.045 -0.06 0.7

RS2521933 -0.39 0.7 -0.198 -0.03 0.7 -1.045 -0.06 0.7

RS10928926 -0.39 0.7 -0.198 -0.03 0.7 -1.045 -0.06 0.7

RS4662986 -0.39 0.7 -0.198 -0.03 0.7 -1.045 -0.06 0.7

RS2464786 -0.39 0.7 -0.198 -0.03 0.7 -1.045 -0.06 0.7

RS4662667 -0.39 0.7 -0.198 -0.03 0.7 -1.045 -0.06 0.7

RS2407073 -0.39 0.7 -0.198 -0.03 0.7 -1.045 -0.06 0.7

RS4600594 -0.39 0.7 -0.198 -0.03 0.7 -1.045 -0.06 0.7

RS949771 -0.39 0.7 -0.198 -0.03 0.7 -1.045 -0.06 0.7

RS1430707 -0.39 0.7 -0.198 -0.03 0.7 -1.045 -0.06 0.7

RS12470817 -0.39 0.7 -0.198 -0.03 0.7 -1.045 -0.06 0.7

RS34052165 -0.39 0.7 -0.198 -0.03 0.7 -1.045 -0.06 0.7

RS7573518 -0.39 0.7 -0.198 -0.03 0.7 -1.045 -0.06 0.7

RS6731616 -0.39 0.7 -0.198 -0.03 0.7 -1.045 -0.06 0.7

RS4850246 -0.39 0.7 -0.198 -0.03 0.7 -1.045 -0.06 0.7

RS4353696 -0.39 0.7 -0.198 -0.03 0.7 -1.045 -0.06 0.7

RS4577331 -0.39 0.7 -0.198 -0.03 0.7 -1.045 -0.06 0.7

RS12691770 -0.39 0.7 -0.198 -0.03 0.7 -1.045 -0.06 0.7

RS3762466 -0.39 0.7 -0.198 -0.03 0.7 -1.045 -0.06 0.7

RS3109140 -0.39 0.7 -0.198 -0.03 0.7 -1.045 -0.06 0.7

RS1367393 -0.39 0.7 -0.198 -0.03 0.7 -1.045 -0.06 0.7

RS6430373 -0.39 0.7 -0.198 -0.03 0.7 -1.045 -0.06 0.7

RS10186393 -0.39 0.7 -0.198 -0.03 0.7 -1.045 -0.06 0.7

RS1370602 -0.36 0.6 -0.198 -0.03 0.6 -0.898 -0.05 0.7

RS6708646 -0.33 0.6 -0.198 -0.03 0.6 -0.735 -0.04 0.7

RS6430397 -0.23 0.6 -0.198 -0.02 0.6 -0.362 -0.02 0.6

RS10176380 -0.21 0.6 -0.198 -0.02 0.6 -0.316 -0.01 0.6

RS7577925 -0.21 0.6 -0.198 -0.02 0.6 -0.316 -0.01 0.6

RS7604549 -0.21 0.6 -0.198 -0.02 0.6 -0.316 -0.01 0.6

RS16826052 -0.21 0.6 -0.198 -0.02 0.6 -0.316 -0.01 0.6

RS1368092 -0.21 0.6 -0.198 -0.02 0.6 -0.316 -0.01 0.6

RS16826387 -0.21 0.6 -0.198 -0.02 0.6 -0.316 -0.01 0.6

RS891821 -0.21 0.6 -0.198 -0.02 0.6 -0.316 -0.01 0.6

RS7588567 -0.21 0.6 -0.198 -0.02 0.6 -0.316 -0.01 0.6

RS2167529 -0.21 0.6 -0.198 -0.02 0.6 -0.316 -0.01 0.6

RS17279673 -0.21 0.6 -0.198 -0.02 0.6 -0.316 -0.01 0.6

RS7569670 -0.21 0.6 -0.198 -0.02 0.6 -0.316 -0.01 0.6

RS7579501 -0.21 0.6 -0.198 -0.02 0.6 -0.316 -0.01 0.6

RS2321299 -0.21 0.6 -0.198 -0.02 0.6 -0.316 -0.01 0.6

RS13382243 -0.21 0.6 -0.198 -0.02 0.6 -0.316 -0.01 0.6

RS6736758 -0.21 0.6 -0.198 -0.02 0.6 -0.316 -0.01 0.6

RS12477481 -0.21 0.6 -0.198 -0.02 0.6 -0.316 -0.01 0.6

RS764686 -0.21 0.6 -0.198 -0.02 0.6 -0.316 -0.01 0.6

RS1257174 -0.21 0.6 -0.198 -0.02 0.6 -0.316 -0.01 0.6

RS1401568 -0.21 0.6 -0.198 -0.02 0.6 -0.316 -0.01 0.6

RS636975 -0.21 0.6 -0.198 -0.02 0.6 -0.316 -0.01 0.6

RS624817 -0.21 0.6 -0.198 -0.02 0.6 -0.316 -0.01 0.6

RS859767 -0.21 0.6 -0.198 -0.02 0.6 -0.316 -0.01 0.6

RS10469555 -0.21 0.6 -0.198 -0.02 0.6 -0.316 -0.01 0.6

RS10496732 -0.21 0.6 -0.198 -0.02 0.6 -0.316 -0.01 0.6

RS749873 -0.21 0.6 -0.198 -0.02 0.6 -0.316 -0.01 0.6

RS1519523 -0.21 0.6 -0.198 -0.02 0.6 -0.316 -0.01 0.6

RS4954579 -0.21 0.6 -0.198 -0.02 0.6 -0.316 -0.01 0.6

RS12465599 -0.21 0.6 -0.198 -0.02 0.6 -0.316 -0.01 0.6

RS2558095 -0.21 0.6 -0.198 -0.02 0.6 -0.316 -0.01 0.6

RS13007264 -0.21 0.6 -0.198 -0.02 0.6 -0.316 -0.01 0.6

RS10194354 -0.21 0.6 -0.198 -0.02 0.6 -0.316 -0.01 0.6

RS550612 -0.21 0.6 -0.198 -0.02 0.6 -0.316 -0.01 0.6

RS6735885 -0.21 0.6 -0.198 -0.02 0.6 -0.316 -0.01 0.6

RS6728076 -0.21 0.6 -0.198 -0.02 0.6 -0.316 -0.01 0.6

RS352188 -0.21 0.6 -0.198 -0.02 0.6 -0.316 -0.01 0.6

RS16839281 -0.21 0.6 -0.198 -0.02 0.6 -0.316 -0.01 0.6

RS11691636 -0.21 0.6 -0.198 -0.02 0.6 -0.316 -0.01 0.6

RS4273272 -0.21 0.6 -0.198 -0.02 0.6 -0.316 -0.01 0.6

RS4476402 -0.21 0.6 -0.198 -0.02 0.6 -0.316 -0.01 0.6

RS4311107 -0.21 0.6 -0.198 -0.02 0.6 -0.316 -0.01 0.6

RS13024237 -0.21 0.6 -0.198 -0.02 0.6 -0.316 -0.01 0.6

RS13405108 -0.21 0.6 -0.198 -0.02 0.6 -0.316 -0.01 0.6

RS13004492 -0.21 0.6 -0.198 -0.02 0.6 -0.316 -0.01 0.6

RS6742749 -0.21 0.6 -0.198 -0.02 0.6 -0.316 -0.01 0.6

RS12470031 -0.21 0.6 -0.198 -0.02 0.6 -0.316 -0.01 0.6

RS963629 -0.21 0.6 -0.198 -0.02 0.6 -0.316 -0.01 0.6

RS1437330 -0.21 0.6 -0.198 -0.02 0.6 -0.316 -0.01 0.6

RS1437305 -0.21 0.6 -0.198 -0.02 0.6 -0.316 -0.01 0.6

RS4538141 -0.21 0.6 -0.198 -0.02 0.6 -0.316 -0.01 0.6

RS7564459 -0.21 0.6 -0.198 -0.02 0.6 -0.316 -0.01 0.6

RS1401935 -0.21 0.6 -0.198 -0.02 0.6 -0.316 -0.01 0.6

RS2141652 -0.21 0.6 -0.198 -0.02 0.6 -0.316 -0.01 0.6

RS825714 -0.21 0.6 -0.198 -0.02 0.6 -0.316 -0.01 0.6

RS10174088 -0.21 0.6 -0.198 -0.02 0.6 -0.316 -0.01 0.6

RS11894060 -0.21 0.6 -0.198 -0.02 0.6 -0.316 -0.01 0.6

RS17387292 -0.21 0.6 -0.198 -0.02 0.6 -0.316 -0.01 0.6

RS11683334 -0.21 0.6 -0.198 -0.02 0.6 -0.316 -0.01 0.6

RS511625 -0.21 0.6 -0.198 -0.02 0.6 -0.316 -0.01 0.6

RS544510 -0.21 0.6 -0.198 -0.02 0.6 -0.316 -0.01 0.6

RS1564934 -0.21 0.6 -0.198 -0.02 0.6 -0.316 -0.01 0.6

RS16844617 -0.21 0.6 -0.198 -0.02 0.6 -0.316 -0.01 0.6

RS387927 -0.21 0.6 -0.198 -0.02 0.6 -0.316 -0.01 0.6

RS9287300 -0.21 0.6 -0.198 -0.02 0.6 -0.316 -0.01 0.6

RS13416050 -0.21 0.6 -0.198 -0.02 0.6 -0.316 -0.01 0.6

RS2091302 -0.21 0.6 -0.198 -0.02 0.6 -0.316 -0.01 0.6

RS2222233 -0.21 0.6 -0.198 -0.02 0.6 -0.316 -0.01 0.6

RS6726240 -0.21 0.6 -0.198 -0.02 0.6 -0.316 -0.01 0.6

RS10496882 -0.21 0.6 -0.198 -0.02 0.6 -0.316 -0.01 0.6

RS12477186 -0.21 0.6 -0.198 -0.02 0.6 -0.316 -0.01 0.6

RS10928120 -0.21 0.6 -0.198 -0.02 0.6 -0.316 -0.01 0.6

RS7598314 -0.21 0.6 -0.198 -0.02 0.6 -0.316 -0.01 0.6

RS352980 -0.21 0.6 -0.198 -0.02 0.6 -0.316 -0.01 0.6

RS352985 -0.21 0.6 -0.198 -0.02 0.6 -0.316 -0.01 0.6

RS2053175 -0.21 0.6 -0.198 -0.02 0.6 -0.316 -0.01 0.6

RS1584201 -0.21 0.6 -0.198 -0.02 0.6 -0.316 -0.01 0.6

RS966624 -0.21 0.6 -0.198 -0.02 0.6 -0.316 -0.01 0.6

RS3768844 -0.21 0.6 -0.198 -0.02 0.6 -0.316 -0.01 0.6

RS354690 -0.21 0.6 -0.198 -0.02 0.6 -0.316 -0.01 0.6

RS16858984 -0.21 0.6 -0.198 -0.02 0.6 -0.316 -0.01 0.6

RS12612084 -0.21 0.6 -0.198 -0.02 0.6 -0.316 -0.01 0.6

RS12691682 -0.21 0.6 -0.198 -0.02 0.6 -0.316 -0.01 0.6

RS12614491 -0.21 0.6 -0.198 -0.02 0.6 -0.316 -0.01 0.6

RS13409480 -0.21 0.6 -0.198 -0.02 0.6 -0.316 -0.01 0.6

RS1257346 -0.21 0.6 -0.198 -0.02 0.6 -0.316 -0.01 0.6

RS7589628 -0.21 0.6 -0.198 -0.02 0.6 -0.316 -0.01 0.6

RS7597006 -0.21 0.6 -0.198 -0.02 0.6 -0.316 -0.01 0.6

RS7600781 -0.21 0.6 -0.198 -0.02 0.6 -0.316 -0.01 0.6

RS4563167 -0.21 0.6 -0.198 -0.02 0.6 -0.316 -0.01 0.6

RS13026163 -0.21 0.6 -0.198 -0.02 0.6 -0.316 -0.01 0.6

RS274846 -0.21 0.6 -0.198 -0.02 0.6 -0.316 -0.01 0.6

RS10445672 -0.21 0.6 -0.198 -0.02 0.6 -0.316 -0.01 0.6

RS10496979 -0.21 0.6 -0.198 -0.02 0.6 -0.316 -0.01 0.6

RS4662248 -0.21 0.6 -0.198 -0.02 0.6 -0.316 -0.01 0.6

RS10496984 -0.21 0.6 -0.198 -0.02 0.6 -0.316 -0.01 0.6

RS16825349 -0.21 0.6 -0.198 -0.02 0.6 -0.316 -0.01 0.6

RS7592820 -0.21 0.6 -0.198 -0.02 0.6 -0.316 -0.01 0.6

RS2246147 -0.21 0.6 -0.198 -0.02 0.6 -0.316 -0.01 0.6

RS1437711 -0.21 0.6 -0.198 -0.02 0.6 -0.316 -0.01 0.6

RS13417105 -0.21 0.6 -0.198 -0.02 0.6 -0.316 -0.01 0.6

RS10496992 -0.21 0.6 -0.198 -0.02 0.6 -0.316 -0.01 0.6

RS2381864 -0.21 0.6 -0.198 -0.02 0.6 -0.316 -0.01 0.6

RS7594453 -0.21 0.6 -0.198 -0.02 0.6 -0.316 -0.01 0.6

RS6745791 -0.21 0.6 -0.198 -0.02 0.6 -0.316 -0.01 0.6

RS7559270 -0.21 0.6 -0.198 -0.02 0.6 -0.316 -0.01 0.6

RS9653189 -0.21 0.6 -0.198 -0.02 0.6 -0.316 -0.01 0.6

RS4275971 -0.21 0.6 -0.198 -0.02 0.6 -0.316 -0.01 0.6

RS6430137 -0.21 0.6 -0.198 -0.02 0.6 -0.316 -0.01 0.6

RS373970 -0.21 0.6 -0.198 -0.02 0.6 -0.316 -0.01 0.6

RS16826971 -0.21 0.6 -0.198 -0.02 0.6 -0.316 -0.01 0.6

RS16827454 -0.21 0.6 -0.198 -0.02 0.6 -0.316 -0.01 0.6

RS6430189 -0.21 0.6 -0.198 -0.02 0.6 -0.316 -0.01 0.6

RS1438873 -0.21 0.6 -0.198 -0.02 0.6 -0.316 -0.01 0.6

RS2118769 -0.21 0.6 -0.198 -0.02 0.6 -0.316 -0.01 0.6

RS4591297 -0.21 0.6 -0.198 -0.02 0.6 -0.316 -0.01 0.6

RS6733995 -0.21 0.6 -0.198 -0.02 0.6 -0.316 -0.01 0.6

RS10928364 -0.21 0.6 -0.198 -0.02 0.6 -0.316 -0.01 0.6

RS10199719 -0.21 0.6 -0.198 -0.02 0.6 -0.316 -0.01 0.6

RS769079 -0.21 0.6 -0.198 -0.02 0.6 -0.316 -0.01 0.6

RS2121433 -0.21 0.6 -0.198 -0.02 0.6 -0.316 -0.01 0.6

RS2377510 -0.21 0.6 -0.198 -0.02 0.6 -0.316 -0.01 0.6

RS10930135 -0.21 0.6 -0.198 -0.02 0.6 -0.316 -0.01 0.6

RS7570723 -0.21 0.6 -0.198 -0.02 0.6 -0.316 -0.01 0.6

RS1196696 -0.21 0.6 -0.198 -0.02 0.6 -0.316 -0.01 0.6

RS1196642 -0.21 0.6 -0.198 -0.02 0.6 -0.316 -0.01 0.6

RS10211034 -0.21 0.6 -0.198 -0.02 0.6 -0.316 -0.01 0.6

RS4130046 -0.21 0.6 -0.198 -0.02 0.6 -0.316 -0.01 0.6

RS12616417 -0.21 0.6 -0.198 -0.02 0.6 -0.316 -0.01 0.6

RS4667393 -0.21 0.6 -0.198 -0.02 0.6 -0.316 -0.01 0.6

RS6710538 -0.21 0.6 -0.198 -0.02 0.6 -0.316 -0.01 0.6

RS12479035 -0.21 0.6 -0.198 -0.02 0.6 -0.316 -0.01 0.6

RS330613 -0.21 0.6 -0.198 -0.02 0.6 -0.316 -0.01 0.6

RS330609 -0.21 0.6 -0.198 -0.02 0.6 -0.316 -0.01 0.6

RS4667379 -0.21 0.6 -0.198 -0.02 0.6 -0.316 -0.01 0.6

RS7563730 -0.21 0.6 -0.198 -0.02 0.6 -0.316 -0.01 0.6

RS11684751 -0.21 0.6 -0.198 -0.02 0.6 -0.316 -0.01 0.6

RS11679729 -0.21 0.6 -0.198 -0.02 0.6 -0.316 -0.01 0.6

RS816895 -0.21 0.6 -0.198 -0.02 0.6 -0.316 -0.01 0.6

RS2341068 -0.21 0.6 -0.198 -0.02 0.6 -0.316 -0.01 0.6

RS4271766 -0.21 0.6 -0.198 -0.02 0.6 -0.316 -0.01 0.6

RS1519768 -0.21 0.6 -0.198 -0.02 0.6 -0.316 -0.01 0.6

RS1859724 -0.21 0.6 -0.198 -0.02 0.6 -0.316 -0.01 0.6

RS12692945 -0.21 0.6 -0.198 -0.02 0.6 -0.316 -0.01 0.6

RS11675356 -0.21 0.6 -0.198 -0.02 0.6 -0.316 -0.01 0.6

RS816709 -0.21 0.6 -0.198 -0.02 0.6 -0.316 -0.01 0.6

RS289932 -0.21 0.6 -0.198 -0.02 0.6 -0.316 -0.01 0.6

RS289904 -0.21 0.6 -0.198 -0.02 0.6 -0.316 -0.01 0.6

RS9287983 -0.21 0.6 -0.198 -0.02 0.6 -0.316 -0.01 0.6

RS16830435 -0.21 0.6 -0.198 -0.02 0.6 -0.316 -0.01 0.6

RS12471646 -0.21 0.6 -0.198 -0.02 0.6 -0.316 -0.01 0.6

RS6740224 -0.21 0.6 -0.198 -0.02 0.6 -0.316 -0.01 0.6

RS4664583 -0.21 0.6 -0.198 -0.02 0.6 -0.316 -0.01 0.6

RS4664593 -0.21 0.6 -0.198 -0.02 0.6 -0.316 -0.01 0.6

RS12474927 -0.21 0.6 -0.198 -0.02 0.6 -0.316 -0.01 0.6

RS9288121 -0.21 0.6 -0.198 -0.02 0.6 -0.316 -0.01 0.6

RS6731128 -0.21 0.6 -0.198 -0.02 0.6 -0.316 -0.01 0.6

RS12476976 -0.21 0.6 -0.198 -0.02 0.6 -0.316 -0.01 0.6

RS1435022 -0.21 0.6 -0.198 -0.02 0.6 -0.316 -0.01 0.6

RS10931477 -0.21 0.6 -0.198 -0.02 0.6 -0.316 -0.01 0.6

RS13405095 -0.21 0.6 -0.198 -0.02 0.6 -0.316 -0.01 0.6

RS6745205 -0.21 0.6 -0.198 -0.02 0.6 -0.316 -0.01 0.6

RS11686661 -0.21 0.6 -0.198 -0.02 0.6 -0.316 -0.01 0.6

RS1221839 -0.21 0.6 -0.198 -0.02 0.6 -0.316 -0.01 0.6

RS16833593 -0.21 0.6 -0.198 -0.02 0.6 -0.316 -0.01 0.6

RS1401741 -0.21 0.6 -0.198 -0.02 0.6 -0.316 -0.01 0.6

RS6723606 -0.21 0.6 -0.198 -0.02 0.6 -0.316 -0.01 0.6

RS1519640 -0.21 0.6 -0.198 -0.02 0.6 -0.316 -0.01 0.6

RS11695673 -0.21 0.6 -0.198 -0.02 0.6 -0.316 -0.01 0.6

RS7592121 -0.21 0.6 -0.198 -0.02 0.6 -0.316 -0.01 0.6

RS7592907 -0.21 0.6 -0.198 -0.02 0.6 -0.316 -0.01 0.6

RS707081 -0.21 0.6 -0.198 -0.02 0.6 -0.316 -0.01 0.6

RS12617045 -0.21 0.6 -0.198 -0.02 0.6 -0.316 -0.01 0.6

RS1896831 -0.21 0.6 -0.198 -0.02 0.6 -0.316 -0.01 0.6

RS16837822 -0.21 0.6 -0.198 -0.02 0.6 -0.316 -0.01 0.6

RS1835154 -0.21 0.6 -0.198 -0.02 0.6 -0.316 -0.01 0.6

RS10210023 -0.21 0.6 -0.198 -0.02 0.6 -0.316 -0.01 0.6

RS3111006 -0.21 0.6 -0.198 -0.02 0.6 -0.316 -0.01 0.6

RS985092 -0.21 0.6 -0.198 -0.02 0.6 -0.316 -0.01 0.6

RS7425016 -0.21 0.6 -0.198 -0.02 0.6 -0.316 -0.01 0.6

RS16838536 -0.21 0.6 -0.198 -0.02 0.6 -0.316 -0.01 0.6

RS10195519 -0.21 0.6 -0.198 -0.02 0.6 -0.316 -0.01 0.6

RS11691512 -0.21 0.6 -0.198 -0.02 0.6 -0.316 -0.01 0.6

RS707120 -0.21 0.6 -0.198 -0.02 0.6 -0.316 -0.01 0.6

RS2882513 -0.21 0.6 -0.198 -0.02 0.6 -0.316 -0.01 0.6

RS1918173 -0.21 0.6 -0.198 -0.02 0.6 -0.316 -0.01 0.6

RS1113060 -0.21 0.6 -0.198 -0.02 0.6 -0.316 -0.01 0.6

RS10497173 -0.21 0.6 -0.198 -0.02 0.6 -0.316 -0.01 0.6

RS2695445 -0.21 0.6 -0.198 -0.02 0.6 -0.316 -0.01 0.6

RS6436373 -0.21 0.6 -0.198 -0.02 0.6 -0.316 -0.01 0.6

RS11689150 -0.21 0.6 -0.198 -0.02 0.6 -0.316 -0.01 0.6

RS8179646 -0.21 0.6 -0.198 -0.02 0.6 -0.316 -0.01 0.6

RS4664215 -0.21 0.6 -0.198 -0.02 0.6 -0.316 -0.01 0.6

RS295830 -0.21 0.6 -0.198 -0.02 0.6 -0.316 -0.01 0.6

RS6732936 -0.21 0.6 -0.198 -0.02 0.6 -0.316 -0.01 0.6

RS263317 -0.21 0.6 -0.198 -0.02 0.6 -0.316 -0.01 0.6

RS3214040 -0.21 0.6 -0.198 -0.02 0.6 -0.316 -0.01 0.6

RS3934681 -0.21 0.6 -0.198 -0.02 0.6 -0.316 -0.01 0.6

RS11899550 -0.21 0.6 -0.198 -0.02 0.6 -0.316 -0.01 0.6

RS7560416 -0.21 0.6 -0.198 -0.02 0.6 -0.316 -0.01 0.6

RS720159 -0.21 0.6 -0.198 -0.02 0.6 -0.316 -0.01 0.6

RS13023991 -0.21 0.6 -0.198 -0.02 0.6 -0.316 -0.01 0.6

RS6707705 -0.21 0.6 -0.198 -0.02 0.6 -0.316 -0.01 0.6

RS16842635 -0.21 0.6 -0.198 -0.02 0.6 -0.316 -0.01 0.6

RS7592105 -0.21 0.6 -0.198 -0.02 0.6 -0.316 -0.01 0.6

RS7567129 -0.21 0.6 -0.198 -0.02 0.6 -0.316 -0.01 0.6

RS13399899 -0.21 0.6 -0.198 -0.02 0.6 -0.316 -0.01 0.6

RS10190089 -0.21 0.6 -0.198 -0.02 0.6 -0.316 -0.01 0.6

RS2193707 -0.21 0.6 -0.198 -0.02 0.6 -0.316 -0.01 0.6

RS6437198 -0.21 0.6 -0.198 -0.02 0.6 -0.316 -0.01 0.6

RS11687201 -0.21 0.6 -0.198 -0.02 0.6 -0.316 -0.01 0.6

RS11686680 -0.21 0.6 -0.198 -0.02 0.6 -0.316 -0.01 0.6

RS264581 -0.21 0.6 -0.198 -0.02 0.6 -0.316 -0.01 0.6

RS6754934 -0.21 0.6 -0.198 -0.02 0.6 -0.316 -0.01 0.6

RS6743012 -0.21 0.6 -0.198 -0.02 0.6 -0.316 -0.01 0.6

RS52848 -0.21 0.6 -0.198 -0.02 0.6 -0.316 -0.01 0.6

RS174253 -0.21 0.6 -0.198 -0.02 0.6 -0.316 -0.01 0.6

RS2193923 -0.21 0.6 -0.198 -0.02 0.6 -0.316 -0.01 0.6

RS6736104 -0.21 0.6 -0.198 -0.02 0.6 -0.316 -0.01 0.6

RS6432565 -0.21 0.6 -0.198 -0.02 0.6 -0.316 -0.01 0.6

RS2556094 -0.21 0.6 -0.198 -0.02 0.6 -0.316 -0.01 0.6

RS2162499 -0.21 0.6 -0.198 -0.02 0.6 -0.316 -0.01 0.6

RS10497205 -0.21 0.6 -0.198 -0.02 0.6 -0.316 -0.01 0.6

RS17341301 -0.21 0.6 -0.198 -0.02 0.6 -0.316 -0.01 0.6

RS6730023 -0.21 0.6 -0.198 -0.02 0.6 -0.316 -0.01 0.6

RS16844863 -0.21 0.6 -0.198 -0.02 0.6 -0.316 -0.01 0.6

RS3772069 -0.21 0.6 -0.198 -0.02 0.6 -0.316 -0.01 0.6

RS6728797 -0.21 0.6 -0.198 -0.02 0.6 -0.316 -0.01 0.6

RS10192369 -0.21 0.6 -0.198 -0.02 0.6 -0.316 -0.01 0.6

RS11888274 -0.21 0.6 -0.198 -0.02 0.6 -0.316 -0.01 0.6

RS6752228 -0.21 0.6 -0.198 -0.02 0.6 -0.316 -0.01 0.6

RS6729946 -0.21 0.6 -0.198 -0.02 0.6 -0.316 -0.01 0.6

RS16845368 -0.21 0.6 -0.198 -0.02 0.6 -0.316 -0.01 0.6

RS6750514 -0.21 0.6 -0.198 -0.02 0.6 -0.316 -0.01 0.6

RS7309 -0.21 0.6 -0.198 -0.02 0.6 -0.316 -0.01 0.6

RS7588198 -0.21 0.6 -0.198 -0.02 0.6 -0.316 -0.01 0.6

RS7597134 -0.21 0.6 -0.198 -0.02 0.6 -0.316 -0.01 0.6

RS10497223 -0.21 0.6 -0.198 -0.02 0.6 -0.316 -0.01 0.6

RS3788976 -0.21 0.6 -0.198 -0.02 0.6 -0.316 -0.01 0.6

RS1861975 -0.21 0.6 -0.198 -0.02 0.6 -0.316 -0.01 0.6

RS10930042 -0.21 0.6 -0.198 -0.02 0.6 -0.316 -0.01 0.6

RS984971 -0.21 0.6 -0.198 -0.02 0.6 -0.316 -0.01 0.6

RS6734436 -0.21 0.6 -0.198 -0.02 0.6 -0.316 -0.01 0.6

RS985309 -0.21 0.6 -0.198 -0.02 0.6 -0.316 -0.01 0.6

RS10194641 -0.21 0.6 -0.198 -0.02 0.6 -0.316 -0.01 0.6

RS16848037 -0.21 0.6 -0.198 -0.02 0.6 -0.316 -0.01 0.6

RS1489636 -0.21 0.6 -0.198 -0.02 0.6 -0.316 -0.01 0.6

RS6752189 -0.21 0.6 -0.198 -0.02 0.6 -0.316 -0.01 0.6

RS1221518 -0.21 0.6 -0.198 -0.02 0.6 -0.316 -0.01 0.6

RS2615330 -0.21 0.6 -0.198 -0.02 0.6 -0.316 -0.01 0.6

RS7424428 -0.21 0.6 -0.198 -0.02 0.6 -0.316 -0.01 0.6

RS12692701 -0.21 0.6 -0.198 -0.02 0.6 -0.316 -0.01 0.6

RS1031736 -0.21 0.6 -0.198 -0.02 0.6 -0.316 -0.01 0.6

RS4667702 -0.21 0.6 -0.198 -0.02 0.6 -0.316 -0.01 0.6

RS6432773 -0.21 0.6 -0.198 -0.02 0.6 -0.316 -0.01 0.6

RS9287843 -0.21 0.6 -0.198 -0.02 0.6 -0.316 -0.01 0.6

RS16849404 -0.21 0.6 -0.198 -0.02 0.6 -0.316 -0.01 0.6

RS1530897 -0.21 0.6 -0.198 -0.02 0.6 -0.316 -0.01 0.6

RS12466514 -0.21 0.6 -0.198 -0.02 0.6 -0.316 -0.01 0.6

RS6737277 -0.21 0.6 -0.198 -0.02 0.6 -0.316 -0.01 0.6

RS17244444 -0.21 0.6 -0.198 -0.02 0.6 -0.316 -0.01 0.6

RS4667772 -0.21 0.6 -0.198 -0.02 0.6 -0.316 -0.01 0.6

RS355810 -0.21 0.6 -0.198 -0.02 0.6 -0.316 -0.01 0.6

RS7596422 -0.21 0.6 -0.198 -0.02 0.6 -0.316 -0.01 0.6

RS4667786 -0.21 0.6 -0.198 -0.02 0.6 -0.316 -0.01 0.6

RS13389345 -0.21 0.6 -0.198 -0.02 0.6 -0.316 -0.01 0.6

RS2288704 -0.21 0.6 -0.198 -0.02 0.6 -0.316 -0.01 0.6

RS6432851 -0.21 0.6 -0.198 -0.02 0.6 -0.316 -0.01 0.6

RS10210369 -0.21 0.6 -0.198 -0.02 0.6 -0.316 -0.01 0.6

RS3924001 -0.21 0.6 -0.198 -0.02 0.6 -0.316 -0.01 0.6

RS7596100 -0.21 0.6 -0.198 -0.02 0.6 -0.316 -0.01 0.6

RS952462 -0.21 0.6 -0.198 -0.02 0.6 -0.316 -0.01 0.6

RS9967700 -0.21 0.6 -0.198 -0.02 0.6 -0.316 -0.01 0.6

RS2218603 -0.21 0.6 -0.198 -0.02 0.6 -0.316 -0.01 0.6

RS2664016 -0.21 0.6 -0.198 -0.02 0.6 -0.316 -0.01 0.6

RS7599152 -0.21 0.6 -0.198 -0.02 0.6 -0.316 -0.01 0.6

RS1431900 -0.21 0.6 -0.198 -0.02 0.6 -0.316 -0.01 0.6

RS10208250 -0.21 0.6 -0.198 -0.02 0.6 -0.316 -0.01 0.6

RS2044682 -0.21 0.6 -0.198 -0.02 0.6 -0.316 -0.01 0.6

RS10930282 -0.21 0.6 -0.198 -0.02 0.6 -0.316 -0.01 0.6

RS1446502 -0.21 0.6 -0.198 -0.02 0.6 -0.316 -0.01 0.6

RS836696 -0.21 0.6 -0.198 -0.02 0.6 -0.316 -0.01 0.6

RS6734516 -0.21 0.6 -0.198 -0.02 0.6 -0.316 -0.01 0.6

RS7574351 -0.21 0.6 -0.198 -0.02 0.6 -0.316 -0.01 0.6

RS17176633 -0.21 0.6 -0.198 -0.02 0.6 -0.316 -0.01 0.6

RS7562413 -0.21 0.6 -0.198 -0.02 0.6 -0.316 -0.01 0.6

RS932167 -0.21 0.6 -0.198 -0.02 0.6 -0.316 -0.01 0.6

RS12616285 -0.21 0.6 -0.198 -0.02 0.6 -0.316 -0.01 0.6

RS12618858 -0.21 0.6 -0.198 -0.02 0.6 -0.316 -0.01 0.6

RS7597374 -0.21 0.6 -0.198 -0.02 0.6 -0.316 -0.01 0.6

RS2044958 -0.21 0.6 -0.198 -0.02 0.6 -0.316 -0.01 0.6

RS6716086 -0.21 0.6 -0.198 -0.02 0.6 -0.316 -0.01 0.6

RS1020603 -0.21 0.6 -0.198 -0.02 0.6 -0.316 -0.01 0.6

RS4668089 -0.21 0.6 -0.198 -0.02 0.6 -0.316 -0.01 0.6

RS10514622 -0.21 0.6 -0.198 -0.02 0.6 -0.316 -0.01 0.6

RS1424937 -0.21 0.6 -0.198 -0.02 0.6 -0.316 -0.01 0.6

RS4668102 -0.21 0.6 -0.198 -0.02 0.6 -0.316 -0.01 0.6

RS1063729 -0.17 0.6 -0.198 -0.01 0.6 -0.241 -0.01 0.6

RS16855886 -0.14 0.6 -0.198 -0.01 0.6 -0.204 -0.01 0.6

RS17200300 -0.11 0.5 -0.198 -0.01 0.6 -0.155 -0.00 0.6

RS3821116 -0.03 0.5 -0.198 -0.00 0.5 -0.039 -0.00 0.5

RS497692 0.04 0.5 1.309 0.02 0.4 0.046 0.00 0.5

RS2241340 0.10 0.5 1.309 0.05 0.3 0.098 0.00 0.5

RS16856332 0.12 0.5 1.309 0.06 0.3 0.112 0.00 0.5

RS6759013 0.34 0.4 1.309 0.16 0.2 0.252 0.02 0.4

RS831043 0.47 0.3 1.309 0.21 0.2 0.303 0.04 0.3

RS830968 0.57 0.3 1.309 0.24 0.15 0.334 0.05 0.3

RS12614394 0.63 0.3 1.309 0.26 0.14 0.349 0.06 0.3

RS830955 0.63 0.3 1.309 0.26 0.14 0.349 0.06 0.3

RS13387104 0.63 0.3 1.309 0.26 0.14 0.349 0.06 0.3

RS13396747 0.63 0.3 1.309 0.26 0.14 0.349 0.06 0.3

RS3829806 0.63 0.3 1.309 0.26 0.14 0.349 0.06 0.3

RS4668163 0.63 0.3 1.309 0.26 0.14 0.349 0.06 0.3

RS1017771 0.63 0.3 1.309 0.26 0.14 0.349 0.06 0.3

RS10930391 0.63 0.3 1.309 0.26 0.14 0.349 0.06 0.3

RS6738152 0.63 0.3 1.309 0.26 0.14 0.349 0.06 0.3

RS4668205 0.63 0.3 1.309 0.26 0.14 0.349 0.06 0.3

RS10930407 0.63 0.3 1.309 0.26 0.14 0.349 0.06 0.3

RS4668224 0.63 0.3 1.309 0.26 0.14 0.349 0.06 0.3

RS10930416 0.63 0.3 1.309 0.26 0.14 0.349 0.06 0.3

RS12611789 0.63 0.3 1.309 0.26 0.14 0.349 0.06 0.3

RS6761096 0.63 0.3 1.309 0.26 0.14 0.349 0.06 0.3

RS10497368 0.63 0.3 1.309 0.26 0.14 0.349 0.06 0.3

RS12616150 0.63 0.3 1.309 0.26 0.14 0.349 0.06 0.3

RS724806 0.63 0.3 1.309 0.26 0.14 0.349 0.06 0.3

RS1012078 0.63 0.3 1.309 0.26 0.14 0.349 0.06 0.3

RS2883782 0.63 0.3 1.309 0.26 0.14 0.349 0.06 0.3

RS724529 -0.21 0.6 -0.198 -0.02 0.6 -0.423 -0.02 0.6

RS1861290 -0.35 0.6 -0.198 -0.03 0.6 -0.788 -0.04 0.7

RS12619910 -0.35 0.6 -0.198 -0.03 0.6 -0.788 -0.04 0.7

RS10192049 -0.35 0.6 -0.198 -0.03 0.6 -0.788 -0.04 0.7

RS4233833 -0.35 0.6 -0.198 -0.03 0.6 -0.788 -0.04 0.7

RS7566096 -0.35 0.6 -0.198 -0.03 0.6 -0.788 -0.04 0.7

RS4667682 -0.35 0.6 -0.198 -0.03 0.6 -0.788 -0.04 0.7

RS6433294 -0.35 0.6 -0.198 -0.03 0.6 -0.788 -0.04 0.7

RS960748 -0.35 0.6 -0.198 -0.03 0.6 -0.788 -0.04 0.7

RS2356791 -0.35 0.6 -0.198 -0.03 0.6 -0.788 -0.04 0.7

RS12476299 -0.35 0.6 -0.198 -0.03 0.6 -0.788 -0.04 0.7

RS6433321 -0.35 0.6 -0.198 -0.03 0.6 -0.788 -0.04 0.7

RS7584187 -0.35 0.6 -0.198 -0.03 0.6 -0.788 -0.04 0.7

RS2016394 -0.35 0.6 -0.198 -0.03 0.6 -0.788 -0.04 0.7

RS10195843 -0.35 0.6 -0.198 -0.03 0.6 -0.788 -0.04 0.7

RS4438453 -0.35 0.6 -0.198 -0.03 0.6 -0.788 -0.04 0.7

RS17286255 -0.35 0.6 -0.198 -0.03 0.6 -0.788 -0.04 0.7

RS836610 -0.35 0.6 -0.198 -0.03 0.6 -0.788 -0.04 0.7

RS10203199 -0.35 0.6 -0.198 -0.03 0.6 -0.788 -0.04 0.7

RS11695769 -0.35 0.6 -0.198 -0.03 0.6 -0.788 -0.04 0.7

RS17761169 -0.35 0.6 -0.198 -0.03 0.6 -0.788 -0.04 0.7

RS935007 -0.35 0.6 -0.198 -0.03 0.6 -0.788 -0.04 0.7

RS3769148 -0.35 0.6 -0.198 -0.03 0.6 -0.788 -0.04 0.7

RS1838023 -0.35 0.6 -0.198 -0.03 0.6 -0.788 -0.04 0.7

RS7562518 -0.35 0.6 -0.198 -0.03 0.6 -0.788 -0.04 0.7

RS11691325 -0.35 0.6 -0.198 -0.03 0.6 -0.788 -0.04 0.7

RS6717268 -0.35 0.6 -0.198 -0.03 0.6 -0.788 -0.04 0.7

RS7567442 -0.35 0.6 -0.198 -0.03 0.6 -0.788 -0.04 0.7

RS13417028 -0.35 0.6 -0.198 -0.03 0.6 -0.788 -0.04 0.7

RS16861690 -0.35 0.6 -0.198 -0.03 0.6 -0.788 -0.04 0.7

RS12473093 -0.35 0.6 -0.198 -0.03 0.6 -0.788 -0.04 0.7

RS10168874 -0.35 0.6 -0.198 -0.03 0.6 -0.788 -0.04 0.7

RS2358235 -0.35 0.6 -0.198 -0.03 0.6 -0.788 -0.04 0.7

RS821147 -0.35 0.6 -0.198 -0.03 0.6 -0.788 -0.04 0.7

RS9287968 -0.35 0.6 -0.198 -0.03 0.6 -0.788 -0.04 0.7

RS2121362 -0.35 0.6 -0.198 -0.03 0.6 -0.788 -0.04 0.7

RS6433461 -0.35 0.6 -0.198 -0.03 0.6 -0.788 -0.04 0.7

RS1595796 -0.35 0.6 -0.198 -0.03 0.6 -0.788 -0.04 0.7

RS7563578 -0.35 0.6 -0.198 -0.03 0.6 -0.788 -0.04 0.7

RS6732100 -0.35 0.6 -0.198 -0.03 0.6 -0.788 -0.04 0.7

RS4972452 -0.35 0.6 -0.198 -0.03 0.6 -0.788 -0.04 0.7

RS4378760 -0.35 0.6 -0.198 -0.03 0.6 -0.788 -0.04 0.7

RS2646179 -0.35 0.6 -0.198 -0.03 0.6 -0.788 -0.04 0.7

RS9646737 -0.35 0.6 -0.198 -0.03 0.6 -0.788 -0.04 0.7

RS12474376 -0.35 0.6 -0.198 -0.03 0.6 -0.788 -0.04 0.7

RS11896469 -0.35 0.6 -0.198 -0.03 0.6 -0.788 -0.04 0.7

RS2121391 -0.35 0.6 -0.198 -0.03 0.6 -0.788 -0.04 0.7

RS16863530 -0.35 0.6 -0.198 -0.03 0.6 -0.788 -0.04 0.7

RS711815 -0.35 0.6 -0.198 -0.03 0.6 -0.788 -0.04 0.7

RS7593931 -0.35 0.6 -0.198 -0.03 0.6 -0.788 -0.04 0.7

RS970797 -0.35 0.6 -0.198 -0.03 0.6 -0.788 -0.04 0.7

RS818361 -0.35 0.6 -0.198 -0.03 0.6 -0.788 -0.04 0.7

RS13024543 -0.35 0.6 -0.198 -0.03 0.6 -0.788 -0.04 0.7

RS16863814 -0.35 0.6 -0.198 -0.03 0.6 -0.788 -0.04 0.7

RS6722762 -0.35 0.6 -0.198 -0.03 0.6 -0.788 -0.04 0.7

RS2969348 -0.35 0.6 -0.198 -0.03 0.6 -0.788 -0.04 0.7

RS6433596 -0.35 0.6 -0.198 -0.03 0.6 -0.788 -0.04 0.7

RS1355849 -0.35 0.6 -0.198 -0.03 0.6 -0.788 -0.04 0.7

RS9287993 -0.35 0.6 -0.198 -0.03 0.6 -0.788 -0.04 0.7

RS11688232 -0.35 0.6 -0.198 -0.03 0.6 -0.788 -0.04 0.7

RS1901826 -0.35 0.6 -0.198 -0.03 0.6 -0.788 -0.04 0.7

RS1370661 -0.35 0.6 -0.198 -0.03 0.6 -0.788 -0.04 0.7

RS16866032 -0.35 0.6 -0.198 -0.03 0.6 -0.788 -0.04 0.7

RS930191 -0.35 0.6 -0.198 -0.03 0.6 -0.788 -0.04 0.7

RS333993 -0.35 0.6 -0.198 -0.03 0.6 -0.788 -0.04 0.7

RS6706330 -0.35 0.6 -0.198 -0.03 0.6 -0.788 -0.04 0.7

RS890579 -0.35 0.6 -0.198 -0.03 0.6 -0.788 -0.04 0.7

RS12469367 -0.35 0.6 -0.198 -0.03 0.6 -0.788 -0.04 0.7

RS726215 -0.35 0.6 -0.198 -0.03 0.6 -0.788 -0.04 0.7

RS7565966 -0.35 0.6 -0.198 -0.03 0.6 -0.788 -0.04 0.7

RS6750760 -0.35 0.6 -0.198 -0.03 0.6 -0.788 -0.04 0.7

RS12992925 -0.35 0.6 -0.198 -0.03 0.6 -0.788 -0.04 0.7

RS7591863 -0.35 0.6 -0.198 -0.03 0.6 -0.788 -0.04 0.7

RS12998067 -0.48 0.7 -0.198 -0.04 0.7 -2.810 -0.14 0.8

RS6757845 -0.49 0.7 -0.198 -0.04 0.7 -3.212 -0.15 0.8

RS13024531 -0.51 0.7 -0.198 -0.04 0.7 -3.954 -0.17 0.8

RS3112931 -0.62 0.7 -0.198 -0.05 0.7 -7.165 -0.34 0.9

RS13012946 -0.65 0.7 -0.198 -0.05 0.7 -7.522 -0.39 0.9

RS4894115 -0.65 0.7 -0.198 -0.05 0.7 -7.522 -0.39 0.9

RS3112943 -0.65 0.7 -0.198 -0.05 0.7 -7.522 -0.39 0.9

RS2276573 -0.65 0.7 -0.198 -0.05 0.7 -7.522 -0.39 0.9

RS723357 -0.65 0.7 -0.198 -0.05 0.7 -7.522 -0.39 0.9

RS13418648 -0.65 0.7 -0.198 -0.05 0.7 -7.522 -0.39 0.9

RS259822 -0.65 0.7 -0.198 -0.05 0.7 -7.522 -0.39 0.9

RS262254 -0.65 0.7 -0.198 -0.05 0.7 -7.522 -0.39 0.9

RS262283 -0.65 0.7 -0.198 -0.05 0.7 -7.522 -0.39 0.9

RS2887173 -0.65 0.7 -0.198 -0.05 0.7 -7.522 -0.39 0.9

RS1529121 -0.65 0.7 -0.198 -0.05 0.7 -7.522 -0.39 0.9

RS1401262 -0.65 0.7 -0.198 -0.05 0.7 -7.522 -0.39 0.9

RS1840111 -0.65 0.7 -0.198 -0.05 0.7 -7.522 -0.39 0.9

RS766598 -0.65 0.7 -0.198 -0.05 0.7 -7.522 -0.39 0.9

RS6727573 -0.65 0.7 -0.198 -0.05 0.7 -7.522 -0.39 0.9

RS6712389 -0.65 0.7 -0.198 -0.05 0.7 -7.522 -0.39 0.9

RS4550629 -0.65 0.7 -0.198 -0.05 0.7 -7.522 -0.39 0.9

RS10048799 -0.65 0.7 -0.198 -0.05 0.7 -7.522 -0.39 0.9

RS10173769 -0.65 0.7 -0.198 -0.05 0.7 -7.522 -0.39 0.9

RS13020845 -0.65 0.7 -0.198 -0.05 0.7 -7.522 -0.39 0.9

RS13393709 -0.65 0.7 -0.198 -0.05 0.7 -7.522 -0.39 0.9

RS6740847 -0.65 0.7 -0.198 -0.05 0.7 -7.522 -0.39 0.9

RS155106 -0.65 0.7 -0.198 -0.05 0.7 -7.522 -0.39 0.9

RS6707704 -0.65 0.7 -0.198 -0.05 0.7 -7.522 -0.39 0.9

RS993648 -0.65 0.7 -0.198 -0.05 0.7 -7.522 -0.39 0.9

RS1473295 -0.65 0.7 -0.198 -0.05 0.7 -7.522 -0.39 0.9

RS6705911 -0.65 0.7 -0.198 -0.05 0.7 -7.522 -0.39 0.9

RS12470644 -0.65 0.7 -0.198 -0.05 0.7 -7.522 -0.39 0.9

RS4643498 -0.65 0.7 -0.198 -0.05 0.7 -7.522 -0.39 0.9

RS10931012 -0.65 0.7 -0.198 -0.05 0.7 -7.522 -0.39 0.9

RS1978650 -0.65 0.7 -0.198 -0.05 0.7 -7.522 -0.39 0.9

RS10931040 -0.65 0.7 -0.198 -0.05 0.7 -7.522 -0.39 0.9

RS6718462 -0.62 0.7 -0.198 -0.05 0.7 -5.437 -0.30 0.9

RS2675093 -0.62 0.7 -0.198 -0.05 0.7 -5.437 -0.30 0.9

RS17758247 -0.62 0.7 -0.198 -0.05 0.7 -5.437 -0.30 0.9

RS11684526 -0.62 0.7 -0.198 -0.05 0.7 -5.437 -0.30 0.9

RS555543 -0.62 0.7 -0.198 -0.05 0.7 -5.437 -0.30 0.9

RS4233772 -0.62 0.7 -0.198 -0.05 0.7 -5.437 -0.30 0.9

RS9288091 -0.62 0.7 -0.198 -0.05 0.7 -5.437 -0.30 0.9

RS1463328 -0.62 0.7 -0.198 -0.05 0.7 -5.437 -0.30 0.9

RS6732860 -0.62 0.7 -0.198 -0.05 0.7 -5.437 -0.30 0.9

RS10803967 -0.62 0.7 -0.198 -0.05 0.7 -5.437 -0.30 0.9

RS6721530 -0.62 0.7 -0.198 -0.05 0.7 -5.437 -0.30 0.9

RS12477832 -0.62 0.7 -0.198 -0.05 0.7 -5.437 -0.30 0.9

RS1682430 -0.62 0.7 -0.198 -0.05 0.7 -5.437 -0.30 0.9

RS11883645 -0.62 0.7 -0.198 -0.05 0.7 -5.437 -0.30 0.9

RS4638756 -0.62 0.7 -0.198 -0.05 0.7 -5.437 -0.30 0.9

RS1443029 -0.62 0.7 -0.198 -0.05 0.7 -5.435 -0.30 0.9

RS899847 -0.62 0.7 -0.198 -0.05 0.7 -5.435 -0.30 0.9

RS7588907 -0.62 0.7 -0.198 -0.05 0.7 -5.435 -0.30 0.9

RS11901504 -0.62 0.7 -0.198 -0.05 0.7 -5.435 -0.30 0.9

RS1483242 -0.62 0.7 -0.198 -0.05 0.7 -5.437 -0.30 0.9

RS13392489 -0.62 0.7 -0.198 -0.05 0.7 -5.437 -0.30 0.9

RS1159985 -0.62 0.7 -0.198 -0.05 0.7 -5.437 -0.30 0.9

RS6711108 -0.62 0.7 -0.198 -0.05 0.7 -5.437 -0.30 0.9

RS13030170 -0.62 0.7 -0.198 -0.05 0.7 -5.437 -0.30 0.9

RS6434158 -0.62 0.7 -0.198 -0.05 0.7 -5.437 -0.30 0.9

RS13030356 -0.62 0.7 -0.198 -0.05 0.7 -5.437 -0.30 0.9

RS7572652 -0.62 0.7 -0.198 -0.05 0.7 -5.437 -0.30 0.9

RS17754256 -0.62 0.7 -0.198 -0.05 0.7 -5.437 -0.30 0.9

RS840616 -0.62 0.7 -0.198 -0.05 0.7 -5.437 -0.30 0.9

RS10187989 -0.62 0.7 -0.198 -0.05 0.7 -5.437 -0.30 0.9

RS2139631 -0.62 0.7 -0.198 -0.05 0.7 -5.437 -0.30 0.9

RS10181512 -0.62 0.7 -0.198 -0.05 0.7 -5.437 -0.30 0.9

RS10193506 -0.62 0.7 -0.198 -0.05 0.7 -5.437 -0.30 0.9

RS781288 -0.62 0.7 -0.198 -0.05 0.7 -5.437 -0.30 0.9

RS1878204 -0.62 0.7 -0.198 -0.05 0.7 -5.437 -0.30 0.9

RS17357652 -0.62 0.7 -0.198 -0.05 0.7 -5.437 -0.30 0.9

RS3106796 -0.62 0.7 -0.198 -0.05 0.7 -5.437 -0.30 0.9

RS6717231 -0.62 0.7 -0.198 -0.05 0.7 -5.437 -0.30 0.9

RS10194682 -0.62 0.7 -0.198 -0.05 0.7 -5.437 -0.30 0.9

RS6718798 -0.62 0.7 -0.198 -0.05 0.7 -5.437 -0.30 0.9

RS11901582 -0.62 0.7 -0.198 -0.05 0.7 -5.437 -0.30 0.9

RS1055091 -0.62 0.7 -0.198 -0.05 0.7 -5.437 -0.30 0.9

RS13034723 -0.62 0.7 -0.198 -0.05 0.7 -5.437 -0.30 0.9

RS10804032 -0.62 0.7 -0.198 -0.05 0.7 -5.437 -0.30 0.9

RS785248 -0.62 0.7 -0.198 -0.05 0.7 -5.437 -0.30 0.9

RS2664253 -0.62 0.7 -0.198 -0.05 0.7 -5.437 -0.30 0.9

RS9646748 -0.62 0.7 -0.198 -0.05 0.7 -5.437 -0.30 0.9

RS10931458 -0.62 0.7 -0.198 -0.05 0.7 -5.437 -0.30 0.9

RS2293765 -0.62 0.7 -0.198 -0.05 0.7 -5.437 -0.30 0.9

RS1263126 -0.62 0.7 -0.198 -0.05 0.7 -5.437 -0.30 0.9

RS3771316 -0.62 0.7 -0.198 -0.05 0.7 -5.437 -0.30 0.9

RS3024908 -0.62 0.7 -0.198 -0.05 0.7 -5.437 -0.30 0.9

RS1517352 -0.62 0.7 -0.198 -0.05 0.7 -5.437 -0.30 0.9

RS7601754 -0.62 0.7 -0.198 -0.05 0.7 -5.437 -0.30 0.9

RS6738544 -0.62 0.7 -0.198 -0.05 0.7 -5.437 -0.30 0.9

RS6704784 -0.62 0.7 -0.198 -0.05 0.7 -5.437 -0.30 0.9

RS4853614 -0.62 0.7 -0.198 -0.05 0.7 -5.437 -0.30 0.9

RS6737786 -0.62 0.7 -0.198 -0.05 0.7 -5.437 -0.30 0.9

RS12472343 -0.62 0.7 -0.198 -0.05 0.7 -5.437 -0.30 0.9

RS4527158 -0.58 0.7 -0.198 -0.05 0.7 -4.513 -0.25 0.9

RS7569546 -0.57 0.7 -0.198 -0.05 0.7 -4.193 -0.23 0.8

RS6434514 -0.57 0.7 -0.198 -0.05 0.7 -4.113 -0.23 0.8

RS7575939 -0.56 0.7 -0.198 -0.05 0.7 -4.003 -0.22 0.8

RS7583281 -0.56 0.7 -0.198 -0.05 0.7 -3.962 -0.22 0.8

RS10931523 -0.56 0.7 -0.198 -0.05 0.7 -3.847 -0.21 0.8

RS6434535 -0.56 0.7 -0.198 -0.05 0.7 -3.839 -0.21 0.8

RS1484887 -0.56 0.7 -0.198 -0.05 0.7 -3.775 -0.21 0.8

RS4850494 -0.56 0.7 -0.198 -0.05 0.7 -3.772 -0.21 0.8

RS9288211 -0.56 0.7 -0.198 -0.05 0.7 -3.804 -0.21 0.8

RS6753418 -0.56 0.7 -0.198 -0.05 0.7 -3.819 -0.21 0.8

RS2719164 -0.56 0.7 -0.198 -0.05 0.7 -3.837 -0.21 0.8

RS6750903 -0.56 0.7 -0.198 -0.05 0.7 -3.844 -0.21 0.8

RS4558599 -0.56 0.7 -0.198 -0.05 0.7 -3.855 -0.21 0.8

RS896441 -0.56 0.7 -0.198 -0.05 0.7 -3.898 -0.22 0.8

RS7421656 -0.56 0.7 -0.198 -0.05 0.7 -3.908 -0.22 0.8

RS16830373 -0.57 0.7 -0.198 -0.05 0.7 -4.145 -0.23 0.8

RS1835438 -0.57 0.7 -0.198 -0.05 0.7 -4.257 -0.23 0.9

RS10200877 -0.57 0.7 -0.198 -0.05 0.7 -4.279 -0.23 0.9

RS12478243 -0.58 0.7 -0.198 -0.05 0.7 -4.376 -0.24 0.9

RS1392658 -0.62 0.7 -0.198 -0.05 0.7 -5.437 -0.30 0.9

RS6434788 -0.62 0.7 -0.198 -0.05 0.7 -5.437 -0.30 0.9

RS12471439 -0.62 0.7 -0.198 -0.05 0.7 -5.437 -0.30 0.9

RS12616415 -0.62 0.7 -0.198 -0.05 0.7 -5.439 -0.30 0.9

RS11893319 -0.62 0.7 -0.198 -0.05 0.7 -5.439 -0.30 0.9

RS16849064 -0.62 0.7 -0.198 -0.05 0.7 -5.439 -0.30 0.9

RS6735887 -0.62 0.7 -0.198 -0.05 0.7 -5.440 -0.30 0.9

RS2376211 -0.62 0.7 -0.198 -0.05 0.7 -5.440 -0.30 0.9

RS4395261 -0.62 0.7 -0.198 -0.05 0.7 -5.441 -0.30 0.9

RS2697298 -0.62 0.7 -0.198 -0.05 0.7 -5.440 -0.30 0.9

RS10931779 -0.62 0.7 -0.198 -0.05 0.7 -5.440 -0.30 0.9

RS7578368 -0.62 0.7 -0.198 -0.05 0.7 -5.440 -0.30 0.9

RS1595823 -0.62 0.7 -0.198 -0.05 0.7 -5.438 -0.30 0.9

RS11682314 -0.62 0.7 -0.198 -0.05 0.7 -5.437 -0.30 0.9

RS1456562 -0.62 0.7 -0.198 -0.05 0.7 -5.437 -0.30 0.9

RS758063 -0.62 0.7 -0.198 -0.05 0.7 -5.437 -0.30 0.9

RS2727783 -0.62 0.7 -0.198 -0.05 0.7 -5.437 -0.30 0.9

RS6434983 -0.62 0.7 -0.198 -0.05 0.7 -5.437 -0.30 0.9

RS13035389 -0.62 0.7 -0.198 -0.05 0.7 -5.437 -0.30 0.9

RS1456520 -0.62 0.7 -0.198 -0.05 0.7 -5.437 -0.30 0.9

RS720527 -0.62 0.7 -0.198 -0.05 0.7 -5.437 -0.30 0.9

RS10931878 -0.62 0.7 -0.198 -0.05 0.7 -5.437 -0.30 0.9

RS3769439 -0.62 0.7 -0.198 -0.05 0.7 -5.437 -0.30 0.9

RS2465665 -0.62 0.7 -0.198 -0.05 0.7 -5.437 -0.30 0.9

RS11684227 -0.62 0.7 -0.198 -0.05 0.7 -5.437 -0.30 0.9

RS10185328 -0.62 0.7 -0.198 -0.05 0.7 -5.437 -0.30 0.9

RS11685853 -0.62 0.7 -0.198 -0.05 0.7 -5.437 -0.30 0.9

RS2518142 -0.62 0.7 -0.198 -0.05 0.7 -5.437 -0.30 0.9

RS3817579 -0.62 0.7 -0.198 -0.05 0.7 -5.437 -0.30 0.9

RS12693942 -0.62 0.7 -0.198 -0.05 0.7 -5.437 -0.30 0.9

RS3754932 -0.62 0.7 -0.198 -0.05 0.7 -5.437 -0.30 0.9

RS9646756 -0.62 0.7 -0.198 -0.05 0.7 -5.437 -0.30 0.9

RS4675217 -0.62 0.7 -0.198 -0.05 0.7 -5.437 -0.30 0.9

RS7561592 -0.62 0.7 -0.198 -0.05 0.7 -5.437 -0.30 0.9

RS4675229 -0.62 0.7 -0.198 -0.05 0.7 -5.437 -0.30 0.9

RS967693 -0.62 0.7 -0.198 -0.05 0.7 -5.437 -0.30 0.9

RS1477032 -0.62 0.7 -0.198 -0.05 0.7 -5.437 -0.30 0.9

RS1521879 -0.62 0.7 -0.198 -0.05 0.7 -5.437 -0.30 0.9

RS2350722 -0.62 0.7 -0.198 -0.05 0.7 -5.437 -0.30 0.9

RS4675261 -0.62 0.7 -0.198 -0.05 0.7 -5.437 -0.30 0.9

RS34829484 -0.62 0.7 -0.198 -0.05 0.7 -5.437 -0.30 0.9

RS7583801 -0.62 0.7 -0.198 -0.05 0.7 -5.437 -0.30 0.9

RS7602496 -0.62 0.7 -0.198 -0.05 0.7 -5.436 -0.30 0.9

RS4675351 -0.62 0.7 -0.198 -0.05 0.7 -5.436 -0.30 0.9

RS7370984 -0.62 0.7 -0.198 -0.05 0.7 -5.436 -0.30 0.9

RS231779 -0.62 0.7 -0.198 -0.05 0.7 -5.437 -0.30 0.9

RS1896286 -0.62 0.7 -0.198 -0.05 0.7 -5.437 -0.30 0.9

RS3096735 -0.62 0.7 -0.198 -0.05 0.7 -5.437 -0.30 0.9

RS6717395 -0.62 0.7 -0.198 -0.05 0.7 -5.437 -0.30 0.9

RS6747093 -0.62 0.7 -0.198 -0.05 0.7 -5.437 -0.30 0.9

RS11892294 -0.62 0.7 -0.198 -0.05 0.7 -5.437 -0.30 0.9

RS2288218 -0.39 0.7 -0.198 -0.03 0.7 -1.289 -0.07 0.7

RS723021 -0.31 0.6 -0.198 -0.03 0.6 -0.649 -0.03 0.7

RS10205524 -0.29 0.6 -0.198 -0.02 0.6 -0.579 -0.03 0.6

RS6760812 -0.28 0.6 -0.198 -0.02 0.6 -0.527 -0.03 0.6

RS7599719 -0.28 0.6 -0.198 -0.02 0.6 -0.525 -0.03 0.6

RS7608404 -0.28 0.6 -0.198 -0.02 0.6 -0.513 -0.02 0.6

RS12373835 -0.27 0.6 -0.198 -0.02 0.6 -0.497 -0.02 0.6

RS10932071 -0.27 0.6 -0.198 -0.02 0.6 -0.495 -0.02 0.6

RS11695066 -0.26 0.6 -0.198 -0.02 0.6 -0.454 -0.02 0.6

RS6741267 -0.26 0.6 -0.198 -0.02 0.6 -0.453 -0.02 0.6

RS1510765 -0.26 0.6 -0.198 -0.02 0.6 -0.452 -0.02 0.6

RS7558785 -0.26 0.6 -0.198 -0.02 0.6 -0.451 -0.02 0.6

RS11681930 -0.26 0.6 -0.198 -0.02 0.6 -0.450 -0.02 0.6

RS1921797 -0.26 0.6 -0.198 -0.02 0.6 -0.450 -0.02 0.6

RS6435279 -0.26 0.6 -0.198 -0.02 0.6 -0.450 -0.02 0.6

RS10932118 -0.26 0.6 -0.198 -0.02 0.6 -0.449 -0.02 0.6

RS6435293 -0.26 0.6 -0.198 -0.02 0.6 -0.449 -0.02 0.6

RS918058 -0.26 0.6 -0.198 -0.02 0.6 -0.449 -0.02 0.6

RS6435300 -0.26 0.6 -0.198 -0.02 0.6 -0.449 -0.02 0.6

RS3770996 -0.26 0.6 -0.198 -0.02 0.6 -0.449 -0.02 0.6

RS10932131 -0.26 0.6 -0.198 -0.02 0.6 -0.449 -0.02 0.6

RS4675571 -0.26 0.6 -0.198 -0.02 0.6 -0.449 -0.02 0.6

RS6735654 -0.26 0.6 -0.198 -0.02 0.6 -0.449 -0.02 0.6

RS4675587 -0.26 0.6 -0.198 -0.02 0.6 -0.449 -0.02 0.6

RS12694045 -0.26 0.6 -0.198 -0.02 0.6 -0.449 -0.02 0.6

RS6435342 -0.26 0.6 -0.198 -0.02 0.6 -0.449 -0.02 0.6

RS10490749 -0.26 0.6 -0.198 -0.02 0.6 -0.449 -0.02 0.6

RS13004504 -0.26 0.6 -0.198 -0.02 0.6 -0.449 -0.02 0.6

RS2287630 -0.26 0.6 -0.198 -0.02 0.6 -0.449 -0.02 0.6

RS13003233 -0.26 0.6 -0.198 -0.02 0.6 -0.449 -0.02 0.6

RS6728532 -0.26 0.6 -0.198 -0.02 0.6 -0.449 -0.02 0.6

RS4675644 -0.26 0.6 -0.198 -0.02 0.6 -0.449 -0.02 0.6

RS1263632 -0.26 0.6 -0.198 -0.02 0.6 -0.449 -0.02 0.6

RS16839275 -0.26 0.6 -0.198 -0.02 0.6 -0.449 -0.02 0.6

RS3791997 -0.26 0.6 -0.198 -0.02 0.6 -0.449 -0.02 0.6

RS6732962 -0.26 0.6 -0.198 -0.02 0.6 -0.449 -0.02 0.6

RS16839586 -0.26 0.6 -0.198 -0.02 0.6 -0.449 -0.02 0.6

RS2161767 -0.26 0.6 -0.198 -0.02 0.6 -0.449 -0.02 0.6

RS2551968 -0.26 0.6 -0.198 -0.02 0.6 -0.449 -0.02 0.6

RS2663867 -0.26 0.6 -0.198 -0.02 0.6 -0.449 -0.02 0.6

RS9636269 -0.26 0.6 -0.198 -0.02 0.6 -0.449 -0.02 0.6

RS720631 -0.26 0.6 -0.198 -0.02 0.6 -0.449 -0.02 0.6

RS10200650 -0.26 0.6 -0.198 -0.02 0.6 -0.449 -0.02 0.6

RS2305430 -0.26 0.6 -0.198 -0.02 0.6 -0.449 -0.02 0.6

RS1437410 -0.26 0.6 -0.198 -0.02 0.6 -0.449 -0.02 0.6

RS1980188 -0.26 0.6 -0.198 -0.02 0.6 -0.449 -0.02 0.6

RS7587994 -0.26 0.6 -0.198 -0.02 0.6 -0.449 -0.02 0.6

RS6728378 -0.26 0.6 -0.198 -0.02 0.6 -0.449 -0.02 0.6

RS11692345 -0.26 0.6 -0.198 -0.02 0.6 -0.449 -0.02 0.6

RS2191915 -0.26 0.6 -0.198 -0.02 0.6 -0.449 -0.02 0.6

RS10490026 -0.26 0.6 -0.198 -0.02 0.6 -0.449 -0.02 0.6

RS6736257 -0.26 0.6 -0.198 -0.02 0.6 -0.449 -0.02 0.6

RS2302539 -0.26 0.6 -0.198 -0.02 0.6 -0.449 -0.02 0.6

RS1509569 -0.26 0.6 -0.198 -0.02 0.6 -0.449 -0.02 0.6

RS1458281 -0.26 0.6 -0.198 -0.02 0.6 -0.449 -0.02 0.6

RS2270476 -0.26 0.6 -0.198 -0.02 0.6 -0.449 -0.02 0.6

RS4672587 -0.26 0.6 -0.198 -0.02 0.6 -0.449 -0.02 0.6

RS13386028 -0.26 0.6 -0.198 -0.02 0.6 -0.449 -0.02 0.6

RS13021675 -0.26 0.6 -0.198 -0.02 0.6 -0.449 -0.02 0.6

RS13019409 -0.26 0.6 -0.198 -0.02 0.6 -0.449 -0.02 0.6

RS6759308 -0.26 0.6 -0.198 -0.02 0.6 -0.449 -0.02 0.6

RS10166020 -0.26 0.6 -0.198 -0.02 0.6 -0.449 -0.02 0.6

RS11901164 -0.26 0.6 -0.198 -0.02 0.6 -0.449 -0.02 0.6

RS1836733 -0.26 0.6 -0.198 -0.02 0.6 -0.449 -0.02 0.6

RS6725395 -0.26 0.6 -0.198 -0.02 0.6 -0.449 -0.02 0.6

RS12329252 -0.26 0.6 -0.198 -0.02 0.6 -0.449 -0.02 0.6

RS10207288 -0.26 0.6 -0.198 -0.02 0.6 -0.449 -0.02 0.6

RS13032114 -0.26 0.6 -0.198 -0.02 0.6 -0.449 -0.02 0.6

RS7423708 -0.26 0.6 -0.198 -0.02 0.6 -0.449 -0.02 0.6

RS7588431 -0.26 0.6 -0.198 -0.02 0.6 -0.449 -0.02 0.6

RS12694259 -0.26 0.6 -0.198 -0.02 0.6 -0.449 -0.02 0.6

RS10189975 -0.26 0.6 -0.198 -0.02 0.6 -0.449 -0.02 0.6

RS1521636 -0.26 0.6 -0.198 -0.02 0.6 -0.449 -0.02 0.6

RS12473610 -0.26 0.6 -0.198 -0.02 0.6 -0.449 -0.02 0.6

RS1394782 -0.26 0.6 -0.198 -0.02 0.6 -0.449 -0.02 0.6

RS1394780 -0.26 0.6 -0.198 -0.02 0.6 -0.449 -0.02 0.6

RS1351593 -0.26 0.6 -0.198 -0.02 0.6 -0.449 -0.02 0.6

RS7355673 -0.26 0.6 -0.198 -0.02 0.6 -0.449 -0.02 0.6

RS6704686 -0.26 0.6 -0.198 -0.02 0.6 -0.449 -0.02 0.6

RS7587255 -0.26 0.6 -0.198 -0.02 0.6 -0.449 -0.02 0.6

RS10469770 -0.26 0.6 -0.198 -0.02 0.6 -0.449 -0.02 0.6

RS1482581 -0.26 0.6 -0.198 -0.02 0.6 -0.449 -0.02 0.6

RS12473314 -0.26 0.6 -0.198 -0.02 0.6 -0.449 -0.02 0.6

RS4673711 -0.26 0.6 -0.198 -0.02 0.6 -0.449 -0.02 0.6

RS10497991 -0.12 0.5 -0.198 -0.01 0.6 -0.154 -0.00 0.6

RS6435760 -0.12 0.5 -0.198 -0.01 0.6 -0.154 -0.00 0.6

RS13001761 -0.12 0.5 -0.198 -0.01 0.6 -0.154 -0.00 0.6

RS10804214 -0.12 0.5 -0.198 -0.01 0.6 -0.154 -0.00 0.6

RS724954 -0.12 0.5 -0.198 -0.01 0.6 -0.154 -0.00 0.6

RS953133 -0.12 0.5 -0.198 -0.01 0.6 -0.154 -0.00 0.6

RS1033070 -0.12 0.5 -0.198 -0.01 0.6 -0.154 -0.00 0.6

RS6715997 -0.12 0.5 -0.198 -0.01 0.6 -0.154 -0.00 0.6

RS10190431 -0.12 0.5 -0.198 -0.01 0.6 -0.154 -0.00 0.6

RS1912196 -0.12 0.5 -0.198 -0.01 0.6 -0.154 -0.00 0.6

RS1912209 -0.12 0.5 -0.198 -0.01 0.6 -0.154 -0.00 0.6

RS4673851 -0.12 0.5 -0.198 -0.01 0.6 -0.154 -0.00 0.6

RS7425093 -0.12 0.5 -0.198 -0.01 0.6 -0.154 -0.00 0.6

RS1838802 -0.12 0.5 -0.198 -0.01 0.6 -0.154 -0.00 0.6

RS13024862 -0.12 0.5 -0.198 -0.01 0.6 -0.154 -0.00 0.6

RS17493319 -0.12 0.5 -0.198 -0.01 0.6 -0.154 -0.00 0.6

RS2030628 -0.12 0.5 -0.198 -0.01 0.6 -0.154 -0.00 0.6

RS6731654 -0.12 0.5 -0.198 -0.01 0.6 -0.154 -0.00 0.6

RS4476347 -0.12 0.5 -0.198 -0.01 0.6 -0.154 -0.00 0.6

RS11687225 -0.12 0.5 -0.198 -0.01 0.6 -0.154 -0.00 0.6

RS16853680 -0.12 0.5 -0.198 -0.01 0.6 -0.154 -0.00 0.6

RS9789612 -0.12 0.5 -0.198 -0.01 0.6 -0.154 -0.00 0.6

RS12995526 -0.12 0.5 -0.198 -0.01 0.6 -0.154 -0.00 0.6

RS1250252 -0.12 0.5 -0.198 -0.01 0.6 -0.154 -0.00 0.6

RS1437793 -0.12 0.5 -0.198 -0.01 0.6 -0.154 -0.00 0.6

RS10181605 -0.12 0.5 -0.198 -0.01 0.6 -0.154 -0.00 0.6

RS1898716 -0.12 0.5 -0.198 -0.01 0.6 -0.154 -0.00 0.6

RS2680185 -0.12 0.5 -0.198 -0.01 0.6 -0.154 -0.00 0.6

RS4672779 -0.12 0.5 -0.198 -0.01 0.6 -0.154 -0.00 0.6

RS940020 -0.12 0.5 -0.198 -0.01 0.6 -0.154 -0.00 0.6

RS2204167 -0.12 0.5 -0.198 -0.01 0.6 -0.154 -0.00 0.6

RS6435922 -0.12 0.5 -0.198 -0.01 0.6 -0.154 -0.00 0.6

RS10432532 -0.12 0.5 -0.198 -0.01 0.6 -0.154 -0.00 0.6

RS7562243 -0.12 0.5 -0.198 -0.01 0.6 -0.154 -0.00 0.6

RS4365463 -0.12 0.5 -0.198 -0.01 0.6 -0.154 -0.00 0.6

RS6706891 -0.12 0.5 -0.198 -0.01 0.6 -0.154 -0.00 0.6

RS934153 -0.12 0.5 -0.198 -0.01 0.6 -0.154 -0.00 0.6

RS6721207 -0.12 0.5 -0.198 -0.01 0.6 -0.154 -0.00 0.6

RS1510839 -0.12 0.5 -0.198 -0.01 0.6 -0.154 -0.00 0.6

RS284574 -0.12 0.5 -0.198 -0.01 0.6 -0.154 -0.00 0.6

RS2012243 -0.12 0.5 -0.198 -0.01 0.6 -0.154 -0.00 0.6

RS17561224 -0.12 0.5 -0.198 -0.01 0.6 -0.154 -0.00 0.6

RS1525608 -0.12 0.5 -0.198 -0.01 0.6 -0.154 -0.00 0.6

RS9341105 -0.12 0.5 -0.198 -0.01 0.6 -0.154 -0.00 0.6

RS2712163 -0.03 0.5 -0.198 -0.00 0.5 -0.031 -0.00 0.5

RS1922001 -0.00 0.5 -0.198 -0.00 0.5 -0.004 -0.00 0.5

RS2241191 0.01 0.5 1.309 0.01 0.4 0.013 0.00 0.5

RS4674132 0.13 0.4 1.309 0.07 0.3 0.114 0.00 0.4

RS13015994 0.15 0.4 1.309 0.08 0.3 0.125 0.00 0.4

RS6723845 0.15 0.4 1.309 0.08 0.3 0.126 0.00 0.4

RS11684828 0.21 0.4 1.309 0.11 0.2 0.165 0.01 0.4

RS7561131 0.17 0.4 1.309 0.09 0.3 0.140 0.01 0.4

RS12620884 0.16 0.4 1.309 0.08 0.3 0.136 0.01 0.4

RS13008340 0.01 0.5 1.309 0.00 0.4 0.009 0.00 0.5

RS2373078 0.00 0.5 1.309 0.00 0.5 0.004 0.00 0.5

RS12694420 -0.20 0.6 -0.198 -0.02 0.6 -0.447 -0.01 0.6

RS6729308 -0.52 0.7 -0.198 -0.04 0.7 -2.335 -0.15 0.8

RS3791950 -0.52 0.7 -0.198 -0.04 0.7 -2.334 -0.15 0.8

RS4672859 -0.52 0.7 -0.198 -0.04 0.7 -2.334 -0.15 0.8

RS6753621 -0.52 0.7 -0.198 -0.04 0.7 -2.334 -0.15 0.8

RS16858496 -0.52 0.7 -0.198 -0.04 0.7 -2.334 -0.15 0.8

RS6712327 -0.52 0.7 -0.198 -0.04 0.7 -2.334 -0.15 0.8

RS876961 -0.52 0.7 -0.198 -0.04 0.7 -2.334 -0.15 0.8

RS3181211 -0.52 0.7 -0.198 -0.04 0.7 -2.334 -0.15 0.8

RS1008562 -0.52 0.7 -0.198 -0.04 0.7 -2.334 -0.15 0.8

RS2556388 -0.52 0.7 -0.198 -0.04 0.7 -2.334 -0.15 0.8

RS359980 -0.52 0.7 -0.198 -0.04 0.7 -2.334 -0.15 0.8

RS883045 -0.52 0.7 -0.198 -0.04 0.7 -2.335 -0.15 0.8

RS7017 -0.52 0.7 -0.198 -0.04 0.7 -2.334 -0.15 0.8

RS10177476 -0.52 0.7 -0.198 -0.04 0.7 -2.334 -0.15 0.8

RS3755064 -0.52 0.7 -0.198 -0.04 0.7 -2.334 -0.15 0.8

RS1043833 -0.52 0.7 -0.198 -0.04 0.7 -2.334 -0.15 0.8

RS6729914 -0.52 0.7 -0.198 -0.04 0.7 -2.334 -0.15 0.8

RS678134 -0.52 0.7 -0.198 -0.04 0.7 -2.334 -0.15 0.8

RS4674423 -0.52 0.7 -0.198 -0.04 0.7 -2.334 -0.15 0.8

RS1346012 -0.52 0.7 -0.198 -0.04 0.7 -2.334 -0.15 0.8

RS6720347 -0.52 0.7 -0.198 -0.04 0.7 -2.334 -0.15 0.8

RS12694490 -0.52 0.7 -0.198 -0.04 0.7 -2.334 -0.15 0.8

RS750764 -0.52 0.7 -0.198 -0.04 0.7 -2.334 -0.15 0.8

RS6756097 -0.52 0.7 -0.198 -0.04 0.7 -2.334 -0.15 0.8

RS13001386 -0.52 0.7 -0.198 -0.04 0.7 -2.334 -0.15 0.8

RS4459724 -0.52 0.7 -0.198 -0.04 0.7 -2.334 -0.15 0.8

RS11676335 -0.52 0.7 -0.198 -0.04 0.7 -2.334 -0.15 0.8

RS724158 -0.52 0.7 -0.198 -0.04 0.7 -2.334 -0.15 0.8

RS4674529 -0.52 0.7 -0.198 -0.04 0.7 -2.334 -0.15 0.8

RS2708138 -0.45 0.7 -0.198 -0.04 0.7 -1.703 -0.10 0.8

RS2708123 -0.39 0.7 -0.198 -0.03 0.7 -1.152 -0.06 0.7

RS7606753 -0.32 0.6 -0.198 -0.03 0.6 -0.708 -0.04 0.7

RS1517459 -0.31 0.6 -0.198 -0.03 0.6 -0.668 -0.03 0.7

RS7562441 -0.26 0.6 -0.198 -0.02 0.6 -0.449 -0.02 0.6

RS2893611 -0.26 0.6 -0.198 -0.02 0.6 -0.449 -0.02 0.6

RS830747 -0.26 0.6 -0.198 -0.02 0.6 -0.449 -0.02 0.6

RS597256 -0.26 0.6 -0.198 -0.02 0.6 -0.449 -0.02 0.6

RS6755709 -0.26 0.6 -0.198 -0.02 0.6 -0.449 -0.02 0.6

RS12476074 -0.26 0.6 -0.198 -0.02 0.6 -0.449 -0.02 0.6

RS1369327 -0.26 0.6 -0.198 -0.02 0.6 -0.449 -0.02 0.6

RS10084181 -0.26 0.6 -0.198 -0.02 0.6 -0.449 -0.02 0.6

RS1991736 -0.26 0.6 -0.198 -0.02 0.6 -0.449 -0.02 0.6

RS6737417 -0.26 0.6 -0.198 -0.02 0.6 -0.449 -0.02 0.6

RS3770205 -0.26 0.6 -0.198 -0.02 0.6 -0.449 -0.02 0.6

RS2710521 -0.26 0.6 -0.198 -0.02 0.6 -0.449 -0.02 0.6

RS13405578 -0.26 0.6 -0.198 -0.02 0.6 -0.449 -0.02 0.6

RS13022647 -0.26 0.6 -0.198 -0.02 0.6 -0.449 -0.02 0.6

RS1025082 -0.26 0.6 -0.198 -0.02 0.6 -0.449 -0.02 0.6

RS348990 -0.26 0.6 -0.198 -0.02 0.6 -0.449 -0.02 0.6

RS6710838 -0.26 0.6 -0.198 -0.02 0.6 -0.449 -0.02 0.6

RS7589708 -0.26 0.6 -0.198 -0.02 0.6 -0.449 -0.02 0.6

RS6757951 -0.26 0.6 -0.198 -0.02 0.6 -0.449 -0.02 0.6

RS6723091 -0.26 0.6 -0.198 -0.02 0.6 -0.449 -0.02 0.6

RS6749903 -0.26 0.6 -0.198 -0.02 0.6 -0.449 -0.02 0.6

RS4674700 -0.26 0.6 -0.198 -0.02 0.6 -0.449 -0.02 0.6

RS1046108 -0.26 0.6 -0.198 -0.02 0.6 -0.449 -0.02 0.6

RS1899039 -0.26 0.6 -0.198 -0.02 0.6 -0.449 -0.02 0.6

RS2166877 -0.26 0.6 -0.198 -0.02 0.6 -0.449 -0.02 0.6

RS2203733 -0.26 0.6 -0.198 -0.02 0.6 -0.449 -0.02 0.6

RS2395982 -0.26 0.6 -0.198 -0.02 0.6 -0.449 -0.02 0.6

RS721357 -0.26 0.6 -0.198 -0.02 0.6 -0.449 -0.02 0.6

RS10176854 -0.26 0.6 -0.198 -0.02 0.6 -0.449 -0.02 0.6

RS4673073 -0.26 0.6 -0.198 -0.02 0.6 -0.449 -0.02 0.6

RS4674815 -0.26 0.6 -0.198 -0.02 0.6 -0.449 -0.02 0.6

RS581815 -0.26 0.6 -0.198 -0.02 0.6 -0.449 -0.02 0.6

RS7604002 -0.26 0.6 -0.198 -0.02 0.6 -0.449 -0.02 0.6

RS6721089 -0.26 0.6 -0.198 -0.02 0.6 -0.448 -0.02 0.6

RS6754561 -0.26 0.6 -0.198 -0.02 0.6 -0.448 -0.02 0.6

RS2894520 -0.26 0.6 -0.198 -0.02 0.6 -0.448 -0.02 0.6

RS6740660 -0.26 0.6 -0.198 -0.02 0.6 -0.448 -0.02 0.6

RS6756793 -0.26 0.6 -0.198 -0.02 0.6 -0.448 -0.02 0.6

RS6436470 -0.26 0.6 -0.198 -0.02 0.6 -0.448 -0.02 0.6

RS16865890 -0.26 0.6 -0.198 -0.02 0.6 -0.448 -0.02 0.6

RS1568751 -0.26 0.6 -0.198 -0.02 0.6 -0.448 -0.02 0.6

RS6717108 -0.26 0.6 -0.198 -0.02 0.6 -0.448 -0.02 0.6

RS7559750 -0.26 0.6 -0.198 -0.02 0.6 -0.448 -0.02 0.6

RS388208 -0.26 0.6 -0.198 -0.02 0.6 -0.449 -0.02 0.6

RS12104926 -0.26 0.6 -0.198 -0.02 0.6 -0.449 -0.02 0.6

RS1443663 -0.26 0.6 -0.198 -0.02 0.6 -0.449 -0.02 0.6

RS4674980 -0.26 0.6 -0.198 -0.02 0.6 -0.449 -0.02 0.6

RS12467977 -0.26 0.6 -0.198 -0.02 0.6 -0.449 -0.02 0.6

RS7571208 -0.26 0.6 -0.198 -0.02 0.6 -0.449 -0.02 0.6

RS1897229 -0.26 0.6 -0.198 -0.02 0.6 -0.449 -0.02 0.6

RS11687544 -0.26 0.6 -0.198 -0.02 0.6 -0.449 -0.02 0.6

RS873024 -0.26 0.6 -0.198 -0.02 0.6 -0.449 -0.02 0.6

RS2943633 -0.26 0.6 -0.198 -0.02 0.6 -0.449 -0.02 0.6

RS1849878 -0.26 0.6 -0.198 -0.02 0.6 -0.449 -0.02 0.6

RS2396306 -0.26 0.6 -0.198 -0.02 0.6 -0.449 -0.02 0.6

RS4395242 -0.26 0.6 -0.198 -0.02 0.6 -0.449 -0.02 0.6

RS11686139 -0.26 0.6 -0.198 -0.02 0.6 -0.449 -0.02 0.6

RS1875336 -0.26 0.6 -0.198 -0.02 0.6 -0.449 -0.02 0.6

RS1316328 -0.26 0.6 -0.198 -0.02 0.6 -0.449 -0.02 0.6

RS1560251 -0.26 0.6 -0.198 -0.02 0.6 -0.449 -0.02 0.6

RS4675140 -0.26 0.6 -0.198 -0.02 0.6 -0.449 -0.02 0.6

RS10174459 -0.26 0.6 -0.198 -0.02 0.6 -0.449 -0.02 0.6

RS11686140 -0.26 0.6 -0.198 -0.02 0.6 -0.449 -0.02 0.6

RS7558432 -0.26 0.6 -0.198 -0.02 0.6 -0.449 -0.02 0.6

RS2396464 -0.26 0.6 -0.198 -0.02 0.6 -0.449 -0.02 0.6

RS6737679 -0.26 0.6 -0.198 -0.02 0.6 -0.449 -0.02 0.6

RS13028299 -0.26 0.6 -0.198 -0.02 0.6 -0.449 -0.02 0.6

RS7577732 -0.26 0.6 -0.198 -0.02 0.6 -0.449 -0.02 0.6

RS9646881 -0.26 0.6 -0.198 -0.02 0.6 -0.449 -0.02 0.6

RS4271760 -0.26 0.6 -0.198 -0.02 0.6 -0.449 -0.02 0.6

RS4496327 -0.26 0.6 -0.198 -0.02 0.6 -0.449 -0.02 0.6

RS7585777 -0.26 0.6 -0.198 -0.02 0.6 -0.449 -0.02 0.6

RS2396492 -0.26 0.6 -0.198 -0.02 0.6 -0.449 -0.02 0.6

RS4973257 -0.26 0.6 -0.198 -0.02 0.6 -0.449 -0.02 0.6

RS10184090 -0.26 0.6 -0.198 -0.02 0.6 -0.449 -0.02 0.6

RS13034664 -0.26 0.6 -0.198 -0.02 0.6 -0.449 -0.02 0.6

RS1403970 -0.26 0.6 -0.198 -0.02 0.6 -0.449 -0.02 0.6

RS6436743 -0.26 0.6 -0.198 -0.02 0.6 -0.449 -0.02 0.6

RS4973044 -0.26 0.6 -0.198 -0.02 0.6 -0.449 -0.02 0.6

RS6436750 -0.26 0.6 -0.198 -0.02 0.6 -0.449 -0.02 0.6

RS4438470 -0.26 0.6 -0.198 -0.02 0.6 -0.449 -0.02 0.6

RS4419223 -0.26 0.6 -0.198 -0.02 0.6 -0.449 -0.02 0.6

RS1528223 -0.26 0.6 -0.198 -0.02 0.6 -0.449 -0.02 0.6

RS17235156 -0.26 0.6 -0.198 -0.02 0.6 -0.449 -0.02 0.6

RS1469181 -0.26 0.6 -0.198 -0.02 0.6 -0.449 -0.02 0.6

RS1435859 -0.26 0.6 -0.198 -0.02 0.6 -0.449 -0.02 0.6

RS800726 -0.26 0.6 -0.198 -0.02 0.6 -0.449 -0.02 0.6

RS6705266 -0.26 0.6 -0.198 -0.02 0.6 -0.449 -0.02 0.6

RS2191835 -0.26 0.6 -0.198 -0.02 0.6 -0.449 -0.02 0.6

RS990672 -0.26 0.6 -0.198 -0.02 0.6 -0.449 -0.02 0.6

RS9808529 -0.26 0.6 -0.198 -0.02 0.6 -0.449 -0.02 0.6

RS6745202 -0.26 0.6 -0.198 -0.02 0.6 -0.449 -0.02 0.6

RS10184838 -0.26 0.6 -0.198 -0.02 0.6 -0.449 -0.02 0.6

RS10933281 -0.26 0.6 -0.198 -0.02 0.6 -0.449 -0.02 0.6

RS1226988 -0.26 0.6 -0.198 -0.02 0.6 -0.449 -0.02 0.6

RS6724020 -0.26 0.6 -0.198 -0.02 0.6 -0.449 -0.02 0.6

RS9288645 -0.26 0.6 -0.198 -0.02 0.6 -0.449 -0.02 0.6

RS6739440 -0.26 0.6 -0.198 -0.02 0.6 -0.449 -0.02 0.6

RS11675728 -0.26 0.6 -0.198 -0.02 0.6 -0.449 -0.02 0.6

RS2193906 -0.26 0.6 -0.198 -0.02 0.6 -0.449 -0.02 0.6

RS10193281 -0.26 0.6 -0.198 -0.02 0.6 -0.449 -0.02 0.6

RS208789 -0.26 0.6 -0.198 -0.02 0.6 -0.449 -0.02 0.6

RS533241 -0.26 0.6 -0.198 -0.02 0.6 -0.449 -0.02 0.6

RS4973246 -0.26 0.6 -0.198 -0.02 0.6 -0.449 -0.02 0.6

RS6754280 -0.26 0.6 -0.198 -0.02 0.6 -0.449 -0.02 0.6

RS6436908 -0.26 0.6 -0.198 -0.02 0.6 -0.449 -0.02 0.6

RS6727261 -0.26 0.6 -0.198 -0.02 0.6 -0.449 -0.02 0.6

RS10498244 -0.26 0.6 -0.198 -0.02 0.6 -0.449 -0.02 0.6

RS2162480 -0.26 0.6 -0.198 -0.02 0.6 -0.449 -0.02 0.6

RS3769845 -0.26 0.6 -0.198 -0.02 0.6 -0.449 -0.02 0.6

RS17330963 -0.26 0.6 -0.198 -0.02 0.6 -0.449 -0.02 0.6

RS16827148 -0.26 0.6 -0.198 -0.02 0.6 -0.449 -0.02 0.6

RS12105723 -0.26 0.6 -0.198 -0.02 0.6 -0.449 -0.02 0.6

RS6728027 -0.26 0.6 -0.198 -0.02 0.6 -0.449 -0.02 0.6

RS13398034 -0.26 0.6 -0.198 -0.02 0.6 -0.449 -0.02 0.6

RS10202501 -0.26 0.6 -0.198 -0.02 0.6 -0.449 -0.02 0.6

RS9288668 -0.26 0.6 -0.198 -0.02 0.6 -0.449 -0.02 0.6

RS11695625 -0.26 0.6 -0.198 -0.02 0.6 -0.449 -0.02 0.6

RS1649917 -0.26 0.6 -0.198 -0.02 0.6 -0.449 -0.02 0.6

RS7562389 -0.26 0.6 -0.198 -0.02 0.6 -0.449 -0.02 0.6

RS6735336 -0.26 0.6 -0.198 -0.02 0.6 -0.449 -0.02 0.6

RS4972964 -0.26 0.6 -0.198 -0.02 0.6 -0.449 -0.02 0.6

RS4972965 -0.26 0.6 -0.198 -0.02 0.6 -0.449 -0.02 0.6

RS2720162 -0.26 0.6 -0.198 -0.02 0.6 -0.449 -0.02 0.6

RS4355094 -0.26 0.6 -0.198 -0.02 0.6 -0.449 -0.02 0.6

RS9973676 -0.26 0.6 -0.198 -0.02 0.6 -0.449 -0.02 0.6

RS3106075 -0.26 0.6 -0.198 -0.02 0.6 -0.449 -0.02 0.6

RS6436988 -0.26 0.6 -0.198 -0.02 0.6 -0.449 -0.02 0.6

RS753855 -0.26 0.6 -0.198 -0.02 0.6 -0.449 -0.02 0.6

RS6750502 -0.26 0.6 -0.198 -0.02 0.6 -0.449 -0.02 0.6

RS1797397 -0.26 0.6 -0.198 -0.02 0.6 -0.449 -0.02 0.6

RS10204481 -0.26 0.6 -0.198 -0.02 0.6 -0.449 -0.02 0.6

RS13424754 -0.26 0.6 -0.198 -0.02 0.6 -0.449 -0.02 0.6

RS16828513 -0.26 0.6 -0.198 -0.02 0.6 -0.449 -0.02 0.6

RS12988522 -0.26 0.6 -0.198 -0.02 0.6 -0.449 -0.02 0.6

RS12996863 -0.26 0.6 -0.198 -0.02 0.6 -0.449 -0.02 0.6

RS4521030 -0.32 0.6 -0.198 -0.03 0.6 -0.721 -0.04 0.7

RS909431 -0.37 0.6 -0.198 -0.03 0.6 -1.033 -0.06 0.7

RS2767 -0.52 0.7 -0.198 -0.04 0.7 -2.385 -0.15 0.8

RS1190452 -0.52 0.7 -0.198 -0.04 0.7 -2.385 -0.15 0.8

RS10933405 -0.52 0.7 -0.198 -0.04 0.7 -2.385 -0.15 0.8

RS1973675 -0.52 0.7 -0.198 -0.04 0.7 -2.385 -0.15 0.8

RS6437079 -0.52 0.7 -0.198 -0.04 0.7 -2.385 -0.15 0.8

RS4439944 -0.52 0.7 -0.198 -0.04 0.7 -2.385 -0.15 0.8

RS10933428 -0.52 0.7 -0.198 -0.04 0.7 -2.385 -0.15 0.8

RS7570061 -0.52 0.7 -0.198 -0.04 0.7 -2.385 -0.15 0.8

RS9247 -0.52 0.7 -0.198 -0.04 0.7 -2.385 -0.15 0.8

RS7563345 -0.52 0.7 -0.198 -0.04 0.7 -2.385 -0.15 0.8

RS1046974 -0.52 0.7 -0.198 -0.04 0.7 -2.385 -0.15 0.8

RS838709 -0.52 0.7 -0.198 -0.04 0.7 -2.385 -0.15 0.8

RS6726046 -0.52 0.7 -0.198 -0.04 0.7 -2.385 -0.15 0.8

RS2603547 -0.52 0.7 -0.198 -0.04 0.7 -2.385 -0.15 0.8

RS1817154 -0.52 0.7 -0.198 -0.04 0.7 -2.385 -0.15 0.8

RS11680450 -0.52 0.7 -0.198 -0.04 0.7 -2.385 -0.15 0.8

RS17863783 -0.52 0.7 -0.198 -0.04 0.7 -2.385 -0.15 0.8

RS4663327 -0.52 0.7 -0.198 -0.04 0.7 -2.385 -0.15 0.8

RS1500481 -0.52 0.7 -0.198 -0.04 0.7 -2.385 -0.15 0.8

RS7606893 -0.52 0.7 -0.198 -0.04 0.7 -2.385 -0.15 0.8

RS13401339 -0.52 0.7 -0.198 -0.04 0.7 -2.385 -0.15 0.8

RS28902187 -0.52 0.7 -0.198 -0.04 0.7 -2.385 -0.15 0.8

RS6708995 -0.52 0.7 -0.198 -0.04 0.7 -2.385 -0.15 0.8

RS599294 -0.52 0.7 -0.198 -0.04 0.7 -2.385 -0.15 0.8

RS10171587 -0.52 0.7 -0.198 -0.04 0.7 -2.385 -0.15 0.8

RS6716659 -0.52 0.7 -0.198 -0.04 0.7 -2.385 -0.15 0.8

RS7584695 -0.52 0.7 -0.198 -0.04 0.7 -2.385 -0.15 0.8

RS2049026 -0.52 0.7 -0.198 -0.04 0.7 -2.385 -0.15 0.8

RS6717293 -0.52 0.7 -0.198 -0.04 0.7 -2.385 -0.15 0.8

RS2176652 -0.52 0.7 -0.198 -0.04 0.7 -2.385 -0.15 0.8

RS11679226 -0.52 0.7 -0.198 -0.04 0.7 -2.385 -0.15 0.8

RS12469734 -0.52 0.7 -0.198 -0.04 0.7 -2.385 -0.15 0.8

RS10190832 -0.52 0.7 -0.198 -0.04 0.7 -2.385 -0.15 0.8

RS884062 -0.52 0.7 -0.198 -0.04 0.7 -2.385 -0.15 0.8

RS7602550 -0.52 0.7 -0.198 -0.04 0.7 -2.385 -0.15 0.8

RS1946949 -0.52 0.7 -0.198 -0.04 0.7 -2.385 -0.15 0.8

RS6431311 -0.52 0.7 -0.198 -0.04 0.7 -2.385 -0.15 0.8

RS2379187 -0.52 0.7 -0.198 -0.04 0.7 -2.385 -0.15 0.8

RS10929076 -0.52 0.7 -0.198 -0.04 0.7 -2.385 -0.15 0.8

RS10174126 -0.52 0.7 -0.198 -0.04 0.7 -2.385 -0.15 0.8

RS10205183 -0.52 0.7 -0.198 -0.04 0.7 -2.385 -0.15 0.8

RS6738858 -0.52 0.7 -0.198 -0.04 0.7 -2.385 -0.15 0.8

RS6741246 -0.52 0.7 -0.198 -0.04 0.7 -2.385 -0.15 0.8

RS6746703 -0.52 0.7 -0.198 -0.04 0.7 -2.385 -0.15 0.8

RS2060126 -0.52 0.7 -0.198 -0.04 0.7 -2.385 -0.15 0.8

RS1431794 -0.52 0.7 -0.198 -0.04 0.7 -2.385 -0.15 0.8

RS1367882 -0.52 0.7 -0.198 -0.04 0.7 -2.385 -0.15 0.8

RS4561612 -0.52 0.7 -0.198 -0.04 0.7 -2.385 -0.15 0.8

RS2116399 -0.52 0.7 -0.198 -0.04 0.7 -2.385 -0.15 0.8

RS1878393 -0.52 0.7 -0.198 -0.04 0.7 -2.385 -0.15 0.8

RS13428454 -0.52 0.7 -0.198 -0.04 0.7 -2.385 -0.15 0.8

RS12474898 -0.52 0.7 -0.198 -0.04 0.7 -2.385 -0.15 0.8

RS6731720 -0.52 0.7 -0.198 -0.04 0.7 -2.385 -0.15 0.8

RS13431390 -0.52 0.7 -0.198 -0.04 0.7 -2.385 -0.15 0.8

RS2103279 -0.52 0.7 -0.198 -0.04 0.7 -2.384 -0.15 0.8

RS12472001 -0.52 0.7 -0.198 -0.04 0.7 -2.384 -0.15 0.8

RS3754670 -0.52 0.7 -0.198 -0.04 0.7 -2.384 -0.15 0.8

RS4663619 -0.52 0.7 -0.198 -0.04 0.7 -2.385 -0.15 0.8

RS7420415 -0.52 0.7 -0.198 -0.04 0.7 -2.385 -0.15 0.8

RS7581447 -0.52 0.7 -0.198 -0.04 0.7 -2.385 -0.15 0.8

RS3768939 -0.52 0.7 -0.198 -0.04 0.7 -2.385 -0.15 0.8

RS1377445 -0.52 0.7 -0.198 -0.04 0.7 -2.385 -0.15 0.8

RS10204742 -0.52 0.7 -0.198 -0.04 0.7 -2.385 -0.15 0.8

RS6431461 -0.52 0.7 -0.198 -0.04 0.7 -2.385 -0.15 0.8

RS7594454 -0.52 0.7 -0.198 -0.04 0.7 -2.385 -0.15 0.8

RS2720100 -0.52 0.7 -0.198 -0.04 0.7 -2.385 -0.15 0.8

RS7556982 -0.52 0.7 -0.198 -0.04 0.7 -2.385 -0.15 0.8

RS13396122 -0.52 0.7 -0.198 -0.04 0.7 -2.385 -0.15 0.8

RS4503948 -0.52 0.7 -0.198 -0.04 0.7 -2.385 -0.15 0.8

RS2701336 -0.52 0.7 -0.198 -0.04 0.7 -2.385 -0.15 0.8

RS7577630 -0.52 0.7 -0.198 -0.04 0.7 -2.385 -0.15 0.8

RS10929217 -0.52 0.7 -0.198 -0.04 0.7 -2.385 -0.15 0.8

RS4663708 -0.52 0.7 -0.198 -0.04 0.7 -2.385 -0.15 0.8

RS6720773 -0.52 0.7 -0.198 -0.04 0.7 -2.385 -0.15 0.8

RS13418586 -0.52 0.7 -0.198 -0.04 0.7 -2.385 -0.15 0.8

RS6742960 -0.52 0.7 -0.198 -0.04 0.7 -2.385 -0.15 0.8

RS1316476 -0.52 0.7 -0.198 -0.04 0.7 -2.385 -0.15 0.8

RS13411547 -0.52 0.7 -0.198 -0.04 0.7 -2.385 -0.15 0.8

RS4278886 -0.52 0.7 -0.198 -0.04 0.7 -2.385 -0.15 0.8

RS7597504 -0.52 0.7 -0.198 -0.04 0.7 -2.385 -0.15 0.8

RS10187629 -0.52 0.7 -0.198 -0.04 0.7 -2.385 -0.15 0.8

RS733579 -0.52 0.7 -0.198 -0.04 0.7 -2.385 -0.15 0.8

RS1198819 -0.52 0.7 -0.198 -0.04 0.7 -2.385 -0.15 0.8

RS12479385 -0.52 0.7 -0.198 -0.04 0.7 -2.385 -0.15 0.8

RS11694250 -0.52 0.7 -0.198 -0.04 0.7 -2.385 -0.15 0.8

RS12692218 -0.52 0.7 -0.198 -0.04 0.7 -2.385 -0.15 0.8

RS4663305 -0.52 0.7 -0.198 -0.04 0.7 -2.385 -0.15 0.8

RS2136772 -0.52 0.7 -0.198 -0.04 0.7 -2.385 -0.15 0.8

RS6731687 -0.52 0.7 -0.198 -0.04 0.7 -2.385 -0.15 0.8

RS1039888 -0.52 0.7 -0.198 -0.04 0.7 -2.385 -0.15 0.8

RS12692242 -0.52 0.7 -0.198 -0.04 0.7 -2.385 -0.15 0.8

RS11124234 -0.52 0.7 -0.198 -0.04 0.7 -2.385 -0.15 0.8

RS4851976 -0.52 0.7 -0.198 -0.04 0.7 -2.385 -0.15 0.8

RS11124207 -0.52 0.7 -0.198 -0.04 0.7 -2.385 -0.15 0.8

RS13405988 -0.52 0.7 -0.198 -0.04 0.7 -2.385 -0.15 0.8

RS3791370 -0.23 0.6 -0.198 -0.02 0.6 -0.708 -0.02 0.6

RS3791406 -0.14 0.6 -0.198 -0.01 0.6 -0.255 -0.01 0.6

RS10182074 0.19 0.4 1.309 0.10 0.3 0.159 0.01 0.4

RS843462 0.25 0.4 1.309 0.12 0.2 0.191 0.01 0.4

RS13430446 0.31 0.4 1.309 0.15 0.2 0.218 0.02 0.4

RS1796449 0.32 0.4 1.309 0.15 0.2 0.222 0.02 0.4

RS1564974 0.32 0.4 1.309 0.15 0.2 0.223 0.02 0.4

RS2008714 0.32 0.4 1.309 0.15 0.2 0.225 0.02 0.4

RS12612406 0.33 0.4 1.309 0.15 0.2 0.226 0.02 0.4

RS12470565 0.33 0.4 1.309 0.15 0.2 0.226 0.02 0.4

RS2412033 0.33 0.4 1.309 0.16 0.2 0.226 0.02 0.4

RS2412042 0.33 0.4 1.309 0.16 0.2 0.226 0.02 0.4

RS4852140 0.33 0.4 1.309 0.16 0.2 0.226 0.02 0.4

RS4853981 0.33 0.4 1.309 0.16 0.2 0.226 0.02 0.4

RS6731916 0.33 0.4 1.309 0.16 0.2 0.226 0.02 0.4

RS2165216 0.33 0.4 1.309 0.16 0.2 0.226 0.02 0.4

RS9784121 0.33 0.4 1.309 0.16 0.2 0.226 0.02 0.4

RS12695007 0.33 0.4 1.309 0.16 0.2 0.226 0.02 0.4

RS903756 0.33 0.4 1.309 0.16 0.2 0.226 0.02 0.4

RS7592376 0.33 0.4 1.309 0.16 0.2 0.226 0.02 0.4

RS6437301 0.33 0.4 1.309 0.16 0.2 0.226 0.02 0.4

RS12695010 0.33 0.4 1.309 0.16 0.2 0.226 0.02 0.4

RS10933587 0.33 0.4 1.309 0.16 0.2 0.226 0.02 0.4

RS11679243 0.33 0.4 1.309 0.16 0.2 0.226 0.02 0.4

RS12614493 0.33 0.4 1.309 0.16 0.2 0.226 0.02 0.4

RS13005508 0.33 0.4 1.309 0.16 0.2 0.226 0.02 0.4

RS6730110 0.33 0.4 1.309 0.16 0.2 0.226 0.02 0.4

RS6728549 0.33 0.4 1.309 0.16 0.2 0.226 0.02 0.4

RS4676380 0.33 0.4 1.309 0.16 0.2 0.226 0.02 0.4

RS4676447 0.33 0.4 1.309 0.16 0.2 0.226 0.02 0.4

RS11886288 0.33 0.4 1.309 0.16 0.2 0.226 0.02 0.4

RS9288742 0.33 0.4 1.309 0.16 0.2 0.226 0.02 0.4

RS4234090 0.33 0.4 1.309 0.16 0.2 0.226 0.02 0.4

RS13030338 0.33 0.4 1.309 0.16 0.2 0.226 0.02 0.4

RS10933532 0.33 0.4 1.309 0.16 0.2 0.226 0.02 0.4

RS16843440 0.33 0.4 1.309 0.16 0.2 0.226 0.02 0.4

RS3771343 0.33 0.4 1.309 0.16 0.2 0.226 0.02 0.4

RS10185319 0.33 0.4 1.309 0.16 0.2 0.226 0.02 0.4

RS12619647 0.33 0.4 1.309 0.16 0.2 0.226 0.02 0.4

RS12469165 0.33 0.4 1.309 0.15 0.2 0.226 0.02 0.4

RS7588212 0.33 0.4 1.309 0.16 0.2 0.227 0.02 0.4

RS760133 0.33 0.4 1.309 0.16 0.2 0.228 0.02 0.4

RS6737791 0.33 0.4 1.309 0.16 0.2 0.228 0.02 0.4

Analysing Chromosome 3

Phenotype: AFFSTAT [ALL] (1 family)

===============================================================================

Pos Zmean pvalue linDelta LOD pvalue expDelta LOD pvalue

min -0.76 0.8 -0.198 -0.06 0.7 -9.999 -0.55 0.9

max 5.05 0.00000 1.309 0.88 0.02 9.999 2.62 0.0003

RS1516337 -0.40 0.7 -0.198 -0.03 0.7 -1.073 -0.06 0.7

RS17329247 -0.40 0.7 -0.198 -0.03 0.7 -1.074 -0.06 0.7

RS3864046 -0.40 0.7 -0.198 -0.03 0.7 -1.074 -0.06 0.7

RS9637430 -0.40 0.7 -0.198 -0.03 0.7 -1.074 -0.06 0.7

RS11130113 -0.40 0.7 -0.198 -0.03 0.7 -1.074 -0.06 0.7

RS9631481 -0.40 0.7 -0.198 -0.03 0.7 -1.074 -0.06 0.7

RS13090889 -0.40 0.7 -0.198 -0.03 0.7 -1.074 -0.06 0.7

RS1357616 -0.40 0.7 -0.198 -0.03 0.7 -1.074 -0.06 0.7

RS7631088 -0.40 0.7 -0.198 -0.03 0.7 -1.074 -0.06 0.7

RS2727996 -0.40 0.7 -0.198 -0.03 0.7 -1.074 -0.06 0.7

RS2655198 -0.40 0.7 -0.198 -0.03 0.7 -1.074 -0.06 0.7

RS7646994 -0.40 0.7 -0.198 -0.03 0.7 -1.074 -0.06 0.7

RS9823491 -0.40 0.7 -0.198 -0.03 0.7 -1.074 -0.06 0.7

RS2013828 -0.40 0.7 -0.198 -0.03 0.7 -1.074 -0.06 0.7

RS17036322 -0.40 0.7 -0.198 -0.03 0.7 -1.074 -0.06 0.7

RS3915436 -0.40 0.7 -0.198 -0.03 0.7 -1.074 -0.06 0.7

RS6777408 -0.40 0.7 -0.198 -0.03 0.7 -1.074 -0.06 0.7

RS7626838 -0.40 0.7 -0.198 -0.03 0.7 -1.074 -0.06 0.7

RS9813890 -0.40 0.7 -0.198 -0.03 0.7 -1.074 -0.06 0.7

RS428179 -0.40 0.7 -0.198 -0.03 0.7 -1.074 -0.06 0.7

RS2128252 -0.40 0.7 -0.198 -0.03 0.7 -1.074 -0.06 0.7

RS9835271 -0.40 0.7 -0.198 -0.03 0.7 -1.074 -0.06 0.7

RS9857772 -0.40 0.7 -0.198 -0.03 0.7 -1.074 -0.06 0.7

RS7636546 -0.40 0.7 -0.198 -0.03 0.7 -1.074 -0.06 0.7

RS9861760 -0.40 0.7 -0.198 -0.03 0.7 -1.074 -0.06 0.7

RS6807710 -0.40 0.7 -0.198 -0.03 0.7 -1.074 -0.06 0.7

RS4317081 -0.40 0.7 -0.198 -0.03 0.7 -1.074 -0.06 0.7

RS6777569 -0.40 0.7 -0.198 -0.03 0.7 -1.074 -0.06 0.7

RS2729005 -0.40 0.7 -0.198 -0.03 0.7 -1.074 -0.06 0.7

RS17007773 -0.40 0.7 -0.198 -0.03 0.7 -1.074 -0.06 0.7

RS4267633 -0.40 0.7 -0.198 -0.03 0.7 -1.074 -0.06 0.7

RS2727927 -0.40 0.7 -0.198 -0.03 0.7 -1.074 -0.06 0.7

RS11720157 -0.40 0.7 -0.198 -0.03 0.7 -1.074 -0.06 0.7

RS11915941 -0.40 0.7 -0.198 -0.03 0.7 -1.074 -0.06 0.7

RS1685496 -0.40 0.7 -0.198 -0.03 0.7 -1.074 -0.06 0.7

RS9841813 -0.40 0.7 -0.198 -0.03 0.7 -1.074 -0.06 0.7

RS11718965 -0.40 0.7 -0.198 -0.03 0.7 -1.074 -0.06 0.7

RS13068877 -0.40 0.7 -0.198 -0.03 0.7 -1.074 -0.06 0.7

RS6442742 -0.40 0.7 -0.198 -0.03 0.7 -1.074 -0.06 0.7

RS6442743 -0.40 0.7 -0.198 -0.03 0.7 -1.074 -0.06 0.7

RS7628663 -0.40 0.7 -0.198 -0.03 0.7 -1.074 -0.06 0.7

RS3935671 -0.40 0.7 -0.198 -0.03 0.7 -1.074 -0.06 0.7

RS2728090 -0.40 0.7 -0.198 -0.03 0.7 -1.074 -0.06 0.7

RS975334 -0.40 0.7 -0.198 -0.03 0.7 -1.074 -0.06 0.7

RS6781149 -0.40 0.7 -0.198 -0.03 0.7 -1.074 -0.06 0.7

RS17584516 -0.40 0.7 -0.198 -0.03 0.7 -1.074 -0.06 0.7

RS11713158 -0.40 0.7 -0.198 -0.03 0.7 -1.074 -0.06 0.7

RS17586083 -0.40 0.7 -0.198 -0.03 0.7 -1.074 -0.06 0.7

RS6781913 -0.40 0.7 -0.198 -0.03 0.7 -1.074 -0.06 0.7

RS751056 -0.40 0.7 -0.198 -0.03 0.7 -1.074 -0.06 0.7

RS7633715 -0.40 0.7 -0.198 -0.03 0.7 -1.074 -0.06 0.7

RS9853379 -0.40 0.7 -0.198 -0.03 0.7 -1.074 -0.06 0.7

RS1039255 -0.40 0.7 -0.198 -0.03 0.7 -1.074 -0.06 0.7

RS339287 -0.40 0.7 -0.198 -0.03 0.7 -1.074 -0.06 0.7

RS149150 -0.40 0.7 -0.198 -0.03 0.7 -1.074 -0.06 0.7

RS3913308 -0.40 0.7 -0.198 -0.03 0.7 -1.074 -0.06 0.7

RS340828 -0.40 0.7 -0.198 -0.03 0.7 -1.074 -0.06 0.7

RS2290610 -0.40 0.7 -0.198 -0.03 0.7 -1.074 -0.06 0.7

RS11713419 -0.40 0.7 -0.198 -0.03 0.7 -1.074 -0.06 0.7

RS967963 -0.40 0.7 -0.198 -0.03 0.7 -1.074 -0.06 0.7

RS3856858 -0.40 0.7 -0.198 -0.03 0.7 -1.074 -0.06 0.7

RS6762199 -0.45 0.7 -0.198 -0.04 0.7 -1.709 -0.10 0.8

RS9817602 -0.59 0.7 -0.198 -0.05 0.7 -9.546 -0.35 0.9

RS17030006 -0.68 0.8 -0.198 -0.05 0.7 -9.999 -0.49 0.9

RS9838824 -0.68 0.8 -0.198 -0.05 0.7 -9.999 -0.49 0.9

RS2060642 -0.68 0.8 -0.198 -0.05 0.7 -9.999 -0.49 0.9

RS9879339 -0.68 0.8 -0.198 -0.05 0.7 -9.999 -0.49 0.9

RS6794507 -0.68 0.8 -0.198 -0.05 0.7 -9.999 -0.49 0.9

RS11713638 -0.68 0.8 -0.198 -0.05 0.7 -9.999 -0.49 0.9

RS901859 -0.68 0.8 -0.198 -0.05 0.7 -9.999 -0.49 0.9

RS13075893 -0.68 0.8 -0.198 -0.05 0.7 -9.999 -0.49 0.9

RS6442820 -0.68 0.8 -0.198 -0.05 0.7 -9.999 -0.49 0.9

RS769799 -0.68 0.8 -0.198 -0.05 0.7 -9.999 -0.49 0.9

RS1385464 -0.68 0.8 -0.198 -0.05 0.7 -9.999 -0.49 0.9

RS10865884 -0.68 0.8 -0.198 -0.05 0.7 -9.999 -0.49 0.9

RS317588 -0.68 0.8 -0.198 -0.05 0.7 -9.999 -0.49 0.9

RS10865894 -0.68 0.8 -0.198 -0.05 0.7 -9.999 -0.49 0.9

RS1987888 -0.68 0.8 -0.198 -0.05 0.7 -9.999 -0.49 0.9

RS795727 -0.68 0.8 -0.198 -0.05 0.7 -9.999 -0.49 0.9

RS711676 -0.68 0.8 -0.198 -0.05 0.7 -9.999 -0.49 0.9

RS7630606 -0.68 0.8 -0.198 -0.05 0.7 -9.999 -0.49 0.9

RS2322679 -0.68 0.8 -0.198 -0.05 0.7 -9.999 -0.49 0.9

RS809437 -0.68 0.8 -0.198 -0.05 0.7 -9.999 -0.49 0.9

RS4685744 -0.68 0.8 -0.198 -0.05 0.7 -9.999 -0.49 0.9

RS1687894 -0.68 0.8 -0.198 -0.05 0.7 -9.999 -0.49 0.9

RS11919486 -0.68 0.8 -0.198 -0.05 0.7 -9.999 -0.49 0.9

RS964403 -0.68 0.8 -0.198 -0.05 0.7 -9.999 -0.49 0.9

RS2322683 -0.68 0.8 -0.198 -0.05 0.7 -9.999 -0.49 0.9

RS6782233 -0.68 0.8 -0.198 -0.05 0.7 -9.999 -0.49 0.9

RS9818111 -0.68 0.8 -0.198 -0.05 0.7 -9.999 -0.49 0.9

RS4685785 -0.68 0.8 -0.198 -0.05 0.7 -9.999 -0.49 0.9

RS6773688 -0.68 0.8 -0.198 -0.05 0.7 -9.999 -0.49 0.9

RS17041262 -0.68 0.8 -0.198 -0.05 0.7 -9.999 -0.49 0.9

RS1866999 -0.68 0.8 -0.198 -0.05 0.7 -9.999 -0.49 0.9

RS9831960 -0.68 0.8 -0.198 -0.05 0.7 -9.999 -0.49 0.9

RS1018109 -0.68 0.8 -0.198 -0.05 0.7 -9.999 -0.49 0.9

RS1873020 -0.68 0.8 -0.198 -0.05 0.7 -9.999 -0.49 0.9

RS1514453 -0.68 0.8 -0.198 -0.05 0.7 -9.999 -0.49 0.9

RS728389 -0.68 0.8 -0.198 -0.05 0.7 -9.999 -0.49 0.9

RS908072 -0.67 0.8 -0.198 -0.05 0.7 -9.999 -0.49 0.9

RS17791096 -0.67 0.8 -0.198 -0.05 0.7 -9.999 -0.49 0.9

RS3864053 -0.61 0.7 -0.198 -0.05 0.7 -9.999 -0.50 0.9

RS463156 -0.17 0.6 -0.198 -0.01 0.6 -0.224 -0.01 0.6

RS1874972 -0.15 0.6 -0.198 -0.01 0.6 -0.200 -0.01 0.6

RS4685929 -0.12 0.5 -0.198 -0.01 0.6 -0.146 -0.00 0.6

RS9874195 -0.22 0.6 -0.198 -0.02 0.6 -0.439 -0.02 0.6

RS6787951 -0.32 0.6 -0.198 -0.03 0.6 -9.999 -0.31 0.9

RS7643083 -0.42 0.7 -0.198 -0.03 0.7 -9.999 -0.48 0.9

RS4685976 -0.50 0.7 -0.198 -0.04 0.7 -9.999 -0.57 0.9

RS7641425 -0.64 0.7 -0.198 -0.05 0.7 -9.999 -0.70 1.0

RS7624621 -0.70 0.8 -0.198 -0.06 0.7 -9.999 -0.75 1.0

RS1469805 -0.74 0.8 -0.198 -0.06 0.7 -9.999 -0.77 1.0

RS9844813 -0.74 0.8 -0.198 -0.06 0.7 -9.999 -0.77 1.0

RS6792001 -0.74 0.8 -0.198 -0.06 0.7 -9.999 -0.77 1.0

RS13088182 -0.74 0.8 -0.198 -0.06 0.7 -9.999 -0.77 1.0

RS9811754 -0.74 0.8 -0.198 -0.06 0.7 -9.999 -0.77 1.0

RS6800214 -0.74 0.8 -0.198 -0.06 0.7 -9.999 -0.77 1.0

RS9879475 -0.74 0.8 -0.198 -0.06 0.7 -9.999 -0.77 1.0

RS1352400 -0.74 0.8 -0.198 -0.06 0.7 -9.999 -0.77 1.0

RS156488 -0.74 0.8 -0.198 -0.06 0.7 -9.999 -0.77 1.0

RS267485 -0.74 0.8 -0.198 -0.06 0.7 -9.999 -0.77 1.0

RS6802713 -0.74 0.8 -0.198 -0.06 0.7 -9.999 -0.77 1.0

RS6787150 -0.74 0.8 -0.198 -0.06 0.7 -9.999 -0.77 1.0

RS11929315 -0.74 0.8 -0.198 -0.06 0.7 -9.999 -0.77 1.0

RS345206 -0.74 0.8 -0.198 -0.06 0.7 -9.999 -0.77 1.0

RS12635360 -0.74 0.8 -0.198 -0.06 0.7 -9.999 -0.77 1.0

RS437555 -0.74 0.8 -0.198 -0.06 0.7 -9.999 -0.77 1.0

RS342026 -0.74 0.8 -0.198 -0.06 0.7 -9.999 -0.77 1.0

RS9311966 -0.74 0.8 -0.198 -0.06 0.7 -9.999 -0.77 1.0

RS2116711 -0.74 0.8 -0.198 -0.06 0.7 -9.999 -0.77 1.0

RS2163735 -0.74 0.8 -0.198 -0.06 0.7 -9.999 -0.77 1.0

RS163326 -0.74 0.8 -0.198 -0.06 0.7 -9.999 -0.77 1.0

RS9848973 -0.74 0.8 -0.198 -0.06 0.7 -9.999 -0.77 1.0

RS1543146 -0.74 0.8 -0.198 -0.06 0.7 -9.999 -0.77 1.0

RS7647001 -0.74 0.8 -0.198 -0.06 0.7 -9.999 -0.77 1.0

RS13066750 -0.74 0.8 -0.198 -0.06 0.7 -9.999 -0.77 1.0

RS1083800 -0.74 0.8 -0.198 -0.06 0.7 -9.999 -0.77 1.0

RS10222352 -0.74 0.8 -0.198 -0.06 0.7 -9.999 -0.77 1.0

RS1569285 -0.74 0.8 -0.198 -0.06 0.7 -9.999 -0.77 1.0

RS1485172 -0.74 0.8 -0.198 -0.06 0.7 -9.999 -0.77 1.0

RS9826424 -0.74 0.8 -0.198 -0.06 0.7 -9.999 -0.77 1.0

RS3864074 -0.74 0.8 -0.198 -0.06 0.7 -9.999 -0.77 1.0

RS1504047 -0.74 0.8 -0.198 -0.06 0.7 -9.999 -0.77 1.0

RS9311998 -0.74 0.8 -0.198 -0.06 0.7 -9.999 -0.77 1.0

RS4686197 -0.74 0.8 -0.198 -0.06 0.7 -9.999 -0.77 1.0

RS7643459 -0.74 0.8 -0.198 -0.06 0.7 -9.999 -0.77 1.0

RS1488342 -0.74 0.8 -0.198 -0.06 0.7 -9.999 -0.77 1.0

RS908481 -0.74 0.8 -0.198 -0.06 0.7 -9.999 -0.77 1.0

RS394174 -0.74 0.8 -0.198 -0.06 0.7 -9.999 -0.77 1.0

RS6789772 -0.74 0.8 -0.198 -0.06 0.7 -9.999 -0.77 1.0

RS1488793 -0.74 0.8 -0.198 -0.06 0.7 -9.999 -0.77 1.0

RS6775971 -0.74 0.8 -0.198 -0.06 0.7 -9.999 -0.77 1.0

RS984475 -0.74 0.8 -0.198 -0.06 0.7 -9.999 -0.77 1.0

RS6799128 -0.74 0.8 -0.198 -0.06 0.7 -9.999 -0.77 1.0

RS1979582 -0.74 0.8 -0.198 -0.06 0.7 -9.999 -0.77 1.0

RS1485222 -0.74 0.8 -0.198 -0.06 0.7 -9.999 -0.77 1.0

RS359006 -0.74 0.8 -0.198 -0.06 0.7 -9.999 -0.77 1.0

RS6777857 -0.74 0.8 -0.198 -0.06 0.7 -9.999 -0.77 1.0

RS903116 -0.74 0.8 -0.198 -0.06 0.7 -9.999 -0.77 1.0

RS12490191 -0.74 0.8 -0.198 -0.06 0.7 -9.999 -0.77 1.0

RS237860 -0.74 0.8 -0.198 -0.06 0.7 -9.999 -0.77 1.0

RS2268491 -0.74 0.8 -0.198 -0.06 0.7 -9.999 -0.77 1.0

RS9846430 -0.74 0.8 -0.198 -0.06 0.7 -9.999 -0.77 1.0

RS11711315 -0.74 0.8 -0.198 -0.06 0.7 -9.999 -0.77 1.0

RS486012 -0.74 0.8 -0.198 -0.06 0.7 -9.999 -0.77 1.0

RS6777129 -0.74 0.8 -0.198 -0.06 0.7 -9.999 -0.77 1.0

RS2662090 -0.74 0.8 -0.198 -0.06 0.7 -9.999 -0.77 1.0

RS4686320 -0.74 0.8 -0.198 -0.06 0.7 -9.999 -0.77 1.0

RS2675186 -0.74 0.8 -0.198 -0.06 0.7 -9.999 -0.77 1.0

RS2700331 -0.74 0.8 -0.198 -0.06 0.7 -9.999 -0.77 1.0

RS2600192 -0.74 0.8 -0.198 -0.06 0.7 -9.999 -0.77 1.0

RS2648568 -0.74 0.8 -0.198 -0.06 0.7 -9.999 -0.77 1.0

RS17050225 -0.74 0.8 -0.198 -0.06 0.7 -9.999 -0.77 1.0

RS2648549 -0.74 0.8 -0.198 -0.06 0.7 -9.999 -0.77 1.0

RS2648573 -0.74 0.8 -0.198 -0.06 0.7 -9.999 -0.77 1.0

RS6443251 -0.74 0.8 -0.198 -0.06 0.7 -9.999 -0.77 1.0

RS4686371 -0.74 0.8 -0.198 -0.06 0.7 -9.999 -0.77 1.0

RS1695432 -0.74 0.8 -0.198 -0.06 0.7 -9.999 -0.77 1.0

RS11465897 -0.74 0.8 -0.198 -0.06 0.7 -9.999 -0.77 1.0

RS2278556 -0.74 0.8 -0.198 -0.06 0.7 -9.999 -0.77 1.0

RS40509 -0.71 0.8 -0.198 -0.06 0.7 -9.999 -0.69 1.0

RS808933 -0.70 0.8 -0.198 -0.06 0.7 -9.999 -0.65 1.0

RS6806666 -0.67 0.7 -0.198 -0.05 0.7 -9.999 -0.53 0.9

RS6799104 -0.66 0.7 -0.198 -0.05 0.7 -9.999 -0.46 0.9

RS1865395 -0.65 0.7 -0.198 -0.05 0.7 -8.400 -0.40 0.9

RS13065552 -0.65 0.7 -0.198 -0.05 0.7 -7.257 -0.38 0.9

RS1601365 -0.65 0.7 -0.198 -0.05 0.7 -7.257 -0.38 0.9

RS2581206 -0.65 0.7 -0.198 -0.05 0.7 -7.257 -0.38 0.9

RS2697153 -0.65 0.7 -0.198 -0.05 0.7 -7.257 -0.38 0.9

RS2675163 -0.65 0.7 -0.198 -0.05 0.7 -7.257 -0.38 0.9

RS2930142 -0.65 0.7 -0.198 -0.05 0.7 -7.257 -0.38 0.9

RS3915855 -0.65 0.7 -0.198 -0.05 0.7 -7.257 -0.38 0.9

RS11929549 -0.65 0.7 -0.198 -0.05 0.7 -7.256 -0.38 0.9

RS7639145 -0.65 0.7 -0.198 -0.05 0.7 -7.256 -0.38 0.9

RS2594972 -0.65 0.7 -0.198 -0.05 0.7 -7.257 -0.38 0.9

RS6766610 -0.65 0.7 -0.198 -0.05 0.7 -7.257 -0.38 0.9

RS1039201 -0.65 0.7 -0.198 -0.05 0.7 -7.257 -0.38 0.9

RS7647310 -0.65 0.7 -0.198 -0.05 0.7 -7.257 -0.38 0.9

RS420537 -0.65 0.7 -0.198 -0.05 0.7 -7.257 -0.38 0.9

RS7626703 -0.65 0.7 -0.198 -0.05 0.7 -7.257 -0.38 0.9

RS378603 -0.65 0.7 -0.198 -0.05 0.7 -7.257 -0.38 0.9

RS305516 -0.65 0.7 -0.198 -0.05 0.7 -7.257 -0.38 0.9

RS7610451 -0.65 0.7 -0.198 -0.05 0.7 -7.257 -0.38 0.9

RS1151999 -0.65 0.7 -0.198 -0.05 0.7 -7.257 -0.38 0.9

RS9872031 -0.65 0.7 -0.198 -0.05 0.7 -7.257 -0.38 0.9

RS6442325 -0.65 0.7 -0.198 -0.05 0.7 -7.257 -0.38 0.9

RS906673 -0.65 0.7 -0.198 -0.05 0.7 -7.257 -0.38 0.9

RS2569997 -0.65 0.7 -0.198 -0.05 0.7 -7.257 -0.38 0.9

RS1010412 -0.65 0.7 -0.198 -0.05 0.7 -7.257 -0.38 0.9

RS6810137 -0.65 0.7 -0.198 -0.05 0.7 -7.257 -0.38 0.9

RS360757 -0.65 0.7 -0.198 -0.05 0.7 -7.257 -0.38 0.9

RS6442359 -0.65 0.7 -0.198 -0.05 0.7 -7.257 -0.38 0.9

RS895752 -0.65 0.7 -0.198 -0.05 0.7 -7.257 -0.38 0.9

RS6785220 -0.65 0.7 -0.198 -0.05 0.7 -7.257 -0.38 0.9

RS2675239 -0.65 0.7 -0.198 -0.05 0.7 -7.257 -0.38 0.9

RS2655218 -0.65 0.7 -0.198 -0.05 0.7 -7.257 -0.38 0.9

RS2630441 -0.65 0.7 -0.198 -0.05 0.7 -7.257 -0.38 0.9

RS2630453 -0.65 0.7 -0.198 -0.05 0.7 -7.257 -0.38 0.9

RS1878173 -0.65 0.7 -0.198 -0.05 0.7 -7.257 -0.38 0.9

RS6442403 -0.65 0.7 -0.198 -0.05 0.7 -7.257 -0.38 0.9

RS9310429 -0.65 0.7 -0.198 -0.05 0.7 -7.258 -0.38 0.9

RS4450798 -0.65 0.7 -0.198 -0.05 0.7 -7.258 -0.38 0.9

RS6442409 -0.65 0.7 -0.198 -0.05 0.7 -7.258 -0.38 0.9

RS873853 -0.65 0.7 -0.198 -0.05 0.7 -7.258 -0.38 0.9

RS2733533 -0.65 0.7 -0.198 -0.05 0.7 -7.257 -0.38 0.9

RS3731149 -0.65 0.7 -0.198 -0.05 0.7 -7.257 -0.38 0.9

RS4428160 -0.65 0.7 -0.198 -0.05 0.7 -7.257 -0.38 0.9

RS1871856 -0.65 0.7 -0.198 -0.05 0.7 -7.257 -0.38 0.9

RS1482038 -0.65 0.7 -0.198 -0.05 0.7 -7.257 -0.38 0.9

RS13091718 -0.65 0.7 -0.198 -0.05 0.7 -7.257 -0.38 0.9

RS13081924 -0.65 0.7 -0.198 -0.05 0.7 -7.257 -0.38 0.9

RS2164576 -0.65 0.7 -0.198 -0.05 0.7 -7.257 -0.38 0.9

RS9683047 -0.65 0.7 -0.198 -0.05 0.7 -7.257 -0.38 0.9

RS7610741 -0.65 0.7 -0.198 -0.05 0.7 -7.257 -0.38 0.9

RS3773174 -0.65 0.7 -0.198 -0.05 0.7 -7.257 -0.38 0.9

RS7647806 -0.65 0.7 -0.198 -0.05 0.7 -7.257 -0.38 0.9

RS4685201 -0.65 0.7 -0.198 -0.05 0.7 -7.257 -0.38 0.9

RS7636830 -0.65 0.7 -0.198 -0.05 0.7 -7.257 -0.38 0.9

RS1687282 -0.65 0.7 -0.198 -0.05 0.7 -7.257 -0.38 0.9

RS752078 -0.65 0.7 -0.198 -0.05 0.7 -7.257 -0.38 0.9

RS293909 -0.65 0.7 -0.198 -0.05 0.7 -7.257 -0.38 0.9

RS2307088 -0.65 0.7 -0.198 -0.05 0.7 -7.257 -0.38 0.9

RS4685234 -0.65 0.7 -0.198 -0.05 0.7 -7.257 -0.38 0.9

RS6808450 -0.65 0.7 -0.198 -0.05 0.7 -7.257 -0.38 0.9

RS7639103 -0.65 0.7 -0.198 -0.05 0.7 -7.257 -0.38 0.9

RS7651842 -0.65 0.7 -0.198 -0.05 0.7 -7.257 -0.38 0.9

RS3773464 -0.65 0.7 -0.198 -0.05 0.7 -7.257 -0.38 0.9

RS7647032 -0.65 0.7 -0.198 -0.05 0.7 -7.257 -0.38 0.9

RS17041167 -0.65 0.7 -0.198 -0.05 0.7 -7.257 -0.38 0.9

RS7640807 -0.65 0.7 -0.198 -0.05 0.7 -7.256 -0.38 0.9

RS1454776 -0.65 0.7 -0.198 -0.05 0.7 -7.257 -0.38 0.9

RS7628888 -0.65 0.7 -0.198 -0.05 0.7 -7.257 -0.38 0.9

RS13060631 -0.65 0.7 -0.198 -0.05 0.7 -7.257 -0.38 0.9

RS924935 -0.65 0.7 -0.198 -0.05 0.7 -7.257 -0.38 0.9

RS6771632 -0.65 0.7 -0.198 -0.05 0.7 -7.257 -0.38 0.9

RS11711765 -0.65 0.7 -0.198 -0.05 0.7 -7.257 -0.38 0.9

RS6442592 -0.65 0.7 -0.198 -0.05 0.7 -7.257 -0.38 0.9

RS842252 -0.65 0.7 -0.198 -0.05 0.7 -7.257 -0.38 0.9

RS7622858 -0.65 0.7 -0.198 -0.05 0.7 -7.257 -0.38 0.9

RS553681 -0.65 0.7 -0.198 -0.05 0.7 -7.257 -0.38 0.9

RS1546650 -0.65 0.7 -0.198 -0.05 0.7 -7.257 -0.38 0.9

RS3773832 -0.65 0.7 -0.198 -0.05 0.7 -7.257 -0.38 0.9

RS6802075 -0.65 0.7 -0.198 -0.05 0.7 -7.257 -0.38 0.9

RS767375 -0.65 0.7 -0.198 -0.05 0.7 -7.257 -0.38 0.9

RS1806555 -0.65 0.7 -0.198 -0.05 0.7 -7.257 -0.38 0.9

RS6442662 -0.65 0.7 -0.198 -0.05 0.7 -7.257 -0.38 0.9

RS11710739 -0.65 0.7 -0.198 -0.05 0.7 -7.257 -0.38 0.9

RS6778609 -0.65 0.7 -0.198 -0.05 0.7 -7.257 -0.38 0.9

RS17043152 -0.65 0.7 -0.198 -0.05 0.7 -7.257 -0.38 0.9

RS1375817 -0.65 0.7 -0.198 -0.05 0.7 -7.257 -0.38 0.9

RS11128872 -0.65 0.7 -0.198 -0.05 0.7 -7.257 -0.38 0.9

RS9854107 -0.65 0.7 -0.198 -0.05 0.7 -7.256 -0.38 0.9

RS6762753 -0.65 0.7 -0.198 -0.05 0.7 -7.257 -0.38 0.9

RS7642423 -0.65 0.7 -0.198 -0.05 0.7 -7.257 -0.38 0.9

RS714141 -0.65 0.7 -0.198 -0.05 0.7 -7.257 -0.38 0.9

RS336630 -0.65 0.7 -0.198 -0.05 0.7 -7.257 -0.38 0.9

RS6802224 -0.65 0.7 -0.198 -0.05 0.7 -7.257 -0.38 0.9

RS11926588 -0.65 0.7 -0.198 -0.05 0.7 -7.257 -0.38 0.9

RS9881034 -0.65 0.7 -0.198 -0.05 0.7 -7.257 -0.38 0.9

RS11717739 -0.65 0.7 -0.198 -0.05 0.7 -7.257 -0.38 0.9

RS17005867 -0.65 0.7 -0.198 -0.05 0.7 -7.257 -0.38 0.9

RS7372627 -0.65 0.7 -0.198 -0.05 0.7 -7.256 -0.38 0.9

RS6804525 -0.65 0.7 -0.198 -0.05 0.7 -7.257 -0.38 0.9

RS4858660 -0.65 0.7 -0.198 -0.05 0.7 -7.257 -0.38 0.9

RS4103004 -0.65 0.7 -0.198 -0.05 0.7 -7.257 -0.38 0.9

RS1915919 -0.65 0.7 -0.198 -0.05 0.7 -7.257 -0.38 0.9

RS6771737 -0.65 0.7 -0.198 -0.05 0.7 -7.257 -0.38 0.9

RS17006760 -0.65 0.7 -0.198 -0.05 0.7 -7.257 -0.38 0.9

RS6801666 -0.65 0.7 -0.198 -0.05 0.7 -7.257 -0.38 0.9

RS4857943 -0.65 0.7 -0.198 -0.05 0.7 -7.257 -0.38 0.9

RS4858248 -0.65 0.7 -0.198 -0.05 0.7 -7.257 -0.38 0.9

RS9841080 -0.65 0.7 -0.198 -0.05 0.7 -7.257 -0.38 0.9

RS11716554 -0.65 0.7 -0.198 -0.05 0.7 -7.257 -0.38 0.9

RS1498994 -0.65 0.7 -0.198 -0.05 0.7 -7.257 -0.38 0.9

RS9864991 -0.65 0.7 -0.198 -0.05 0.7 -7.257 -0.38 0.9

RS11128984 -0.65 0.7 -0.198 -0.05 0.7 -7.257 -0.38 0.9

RS1846072 -0.65 0.7 -0.198 -0.05 0.7 -7.257 -0.38 0.9

RS4858297 -0.65 0.7 -0.198 -0.05 0.7 -7.257 -0.38 0.9

RS6763061 -0.65 0.7 -0.198 -0.05 0.7 -7.257 -0.38 0.9

RS2661406 -0.65 0.7 -0.198 -0.05 0.7 -7.257 -0.38 0.9

RS17008470 -0.65 0.7 -0.198 -0.05 0.7 -7.257 -0.38 0.9

RS1026690 -0.65 0.7 -0.198 -0.05 0.7 -7.257 -0.38 0.9

RS1457587 -0.65 0.7 -0.198 -0.05 0.7 -7.257 -0.38 0.9

RS17009067 -0.65 0.7 -0.198 -0.05 0.7 -7.257 -0.38 0.9

RS721623 -0.65 0.7 -0.198 -0.05 0.7 -7.257 -0.38 0.9

RS957589 -0.65 0.7 -0.198 -0.05 0.7 -7.257 -0.38 0.9

RS1320288 -0.65 0.7 -0.198 -0.05 0.7 -7.257 -0.38 0.9

RS9842530 -0.65 0.7 -0.198 -0.05 0.7 -7.257 -0.38 0.9

RS2630807 -0.65 0.7 -0.198 -0.05 0.7 -7.257 -0.38 0.9

RS2670253 -0.65 0.7 -0.198 -0.05 0.7 -7.257 -0.38 0.9

RS677508 -0.65 0.7 -0.198 -0.05 0.7 -7.257 -0.38 0.9

RS779162 -0.65 0.7 -0.198 -0.05 0.7 -7.257 -0.38 0.9

RS941349 -0.65 0.7 -0.198 -0.05 0.7 -7.257 -0.38 0.9

RS2928351 -0.65 0.7 -0.198 -0.05 0.7 -7.257 -0.38 0.9

RS779183 -0.65 0.7 -0.198 -0.05 0.7 -7.257 -0.38 0.9

RS2728981 -0.65 0.7 -0.198 -0.05 0.7 -7.257 -0.38 0.9

RS937105 -0.65 0.7 -0.198 -0.05 0.7 -7.257 -0.38 0.9

RS1389663 -0.65 0.7 -0.198 -0.05 0.7 -7.257 -0.38 0.9

RS1121249 -0.65 0.7 -0.198 -0.05 0.7 -7.257 -0.38 0.9

RS1901104 -0.65 0.7 -0.198 -0.05 0.7 -7.257 -0.38 0.9

RS11719717 -0.65 0.7 -0.198 -0.05 0.7 -7.257 -0.38 0.9

RS13071252 -0.65 0.7 -0.198 -0.05 0.7 -7.257 -0.38 0.9

RS12633771 -0.65 0.7 -0.198 -0.05 0.7 -7.257 -0.38 0.9

RS4858453 -0.65 0.7 -0.198 -0.05 0.7 -7.257 -0.38 0.9

RS296168 -0.65 0.7 -0.198 -0.05 0.7 -7.257 -0.38 0.9

RS978239 -0.65 0.7 -0.198 -0.05 0.7 -7.257 -0.38 0.9

RS13058941 -0.65 0.7 -0.198 -0.05 0.7 -7.257 -0.38 0.9

RS1485384 -0.65 0.7 -0.198 -0.05 0.7 -7.257 -0.38 0.9

RS4390954 -0.65 0.7 -0.198 -0.05 0.7 -7.257 -0.38 0.9

RS7630344 -0.65 0.7 -0.198 -0.05 0.7 -7.257 -0.38 0.9

RS1496653 -0.65 0.7 -0.198 -0.05 0.7 -7.257 -0.38 0.9

RS6787720 -0.65 0.7 -0.198 -0.05 0.7 -7.257 -0.38 0.9

RS9867427 -0.65 0.7 -0.198 -0.05 0.7 -7.257 -0.38 0.9

RS4396825 -0.65 0.7 -0.198 -0.05 0.7 -7.257 -0.38 0.9

RS17013665 -0.65 0.7 -0.198 -0.05 0.7 -7.257 -0.38 0.9

RS951015 -0.65 0.7 -0.198 -0.05 0.7 -7.257 -0.38 0.9

RS4858100 -0.65 0.7 -0.198 -0.05 0.7 -7.257 -0.38 0.9

RS1705734 -0.65 0.7 -0.198 -0.05 0.7 -7.257 -0.38 0.9

RS13097208 -0.65 0.7 -0.198 -0.05 0.7 -7.257 -0.38 0.9

RS7619754 -0.65 0.7 -0.198 -0.05 0.7 -7.257 -0.38 0.9

RS2360957 -0.65 0.7 -0.198 -0.05 0.7 -7.257 -0.38 0.9

RS7610039 -0.65 0.7 -0.198 -0.05 0.7 -7.257 -0.38 0.9

RS1857793 -0.65 0.7 -0.198 -0.05 0.7 -7.257 -0.38 0.9

RS6550858 -0.65 0.7 -0.198 -0.05 0.7 -7.257 -0.38 0.9

RS9852824 -0.65 0.7 -0.198 -0.05 0.7 -7.257 -0.38 0.9

RS9862855 -0.65 0.7 -0.198 -0.05 0.7 -7.257 -0.38 0.9

RS4077423 -0.65 0.7 -0.198 -0.05 0.7 -7.257 -0.38 0.9

RS9860870 -0.65 0.7 -0.198 -0.05 0.7 -7.257 -0.38 0.9

RS7429669 -0.65 0.7 -0.198 -0.05 0.7 -7.257 -0.38 0.9

RS6780762 -0.65 0.7 -0.198 -0.05 0.7 -7.257 -0.38 0.9

RS7640521 -0.65 0.7 -0.198 -0.05 0.7 -7.257 -0.38 0.9

RS11129180 -0.65 0.7 -0.198 -0.05 0.7 -7.257 -0.38 0.9

RS322707 -0.65 0.7 -0.198 -0.05 0.7 -7.256 -0.38 0.9

RS13069258 -0.65 0.7 -0.198 -0.05 0.7 -7.256 -0.38 0.9

RS322683 -0.65 0.7 -0.198 -0.05 0.7 -7.256 -0.38 0.9

RS12497123 -0.65 0.7 -0.198 -0.05 0.7 -7.257 -0.38 0.9

RS7628541 -0.65 0.7 -0.198 -0.05 0.7 -7.257 -0.38 0.9

RS6800566 -0.65 0.7 -0.198 -0.05 0.7 -7.257 -0.38 0.9

RS13314219 -0.65 0.7 -0.198 -0.05 0.7 -7.257 -0.38 0.9

RS1153603 -0.65 0.7 -0.198 -0.05 0.7 -7.257 -0.38 0.9

RS3773439 -0.65 0.7 -0.198 -0.05 0.7 -7.257 -0.38 0.9

RS1881708 -0.65 0.7 -0.198 -0.05 0.7 -7.257 -0.38 0.9

RS6779291 -0.48 0.7 -0.198 -0.04 0.7 -6.777 -0.22 0.8

RS9856369 -0.39 0.7 -0.198 -0.03 0.7 -5.879 -0.11 0.8

RS10470654 -0.34 0.6 -0.198 -0.03 0.6 -1.375 -0.06 0.7

RS10510573 -0.17 0.6 -0.198 -0.01 0.6 -0.255 -0.01 0.6

RS959685 -0.15 0.6 -0.198 -0.01 0.6 -0.208 -0.01 0.6

RS2036430 -0.13 0.6 -0.198 -0.01 0.6 -0.168 -0.00 0.6

RS7638812 -0.12 0.5 -0.198 -0.01 0.6 -0.157 -0.00 0.6

RS7643655 -0.12 0.5 -0.198 -0.01 0.6 -0.150 -0.00 0.6

RS6790894 -0.11 0.5 -0.198 -0.01 0.6 -0.134 -0.00 0.5

RS6788033 -0.11 0.5 -0.198 -0.01 0.6 -0.133 -0.00 0.5

RS9858519 -0.11 0.5 -0.198 -0.01 0.6 -0.134 -0.00 0.5

RS6792668 -0.11 0.5 -0.198 -0.01 0.6 -0.136 -0.00 0.5

RS1155107 -0.11 0.5 -0.198 -0.01 0.6 -0.138 -0.00 0.5

RS12635000 -0.12 0.5 -0.198 -0.01 0.6 -0.144 -0.00 0.5

RS1466957 -0.14 0.6 -0.198 -0.01 0.6 -0.185 -0.01 0.6

RS1374798 -0.18 0.6 -0.198 -0.01 0.6 -0.258 -0.01 0.6

RS9871261 -0.45 0.7 -0.198 -0.04 0.7 -1.838 -0.10 0.8

RS2643818 -0.52 0.7 -0.198 -0.04 0.7 -2.385 -0.15 0.8

RS6762388 -0.52 0.7 -0.198 -0.04 0.7 -2.385 -0.15 0.8

RS3806624 -0.52 0.7 -0.198 -0.04 0.7 -2.385 -0.15 0.8

RS7621069 -0.52 0.7 -0.198 -0.04 0.7 -2.385 -0.15 0.8

RS2618108 -0.52 0.7 -0.198 -0.04 0.7 -2.385 -0.15 0.8

RS2063034 -0.52 0.7 -0.198 -0.04 0.7 -2.385 -0.15 0.8

RS669607 -0.52 0.7 -0.198 -0.04 0.7 -2.385 -0.15 0.8

RS9823676 -0.52 0.7 -0.198 -0.04 0.7 -2.385 -0.15 0.8

RS11706637 -0.52 0.7 -0.198 -0.04 0.7 -2.385 -0.15 0.8

RS11712251 -0.52 0.7 -0.198 -0.04 0.7 -2.385 -0.15 0.8

RS6800659 -0.52 0.7 -0.198 -0.04 0.7 -2.385 -0.15 0.8

RS4306817 -0.52 0.7 -0.198 -0.04 0.7 -2.385 -0.15 0.8

RS1461803 -0.52 0.7 -0.198 -0.04 0.7 -2.385 -0.15 0.8

RS4378932 -0.52 0.7 -0.198 -0.04 0.7 -2.385 -0.15 0.8

RS2199411 -0.52 0.7 -0.198 -0.04 0.7 -2.385 -0.15 0.8

RS9873347 -0.52 0.7 -0.198 -0.04 0.7 -2.385 -0.15 0.8

RS6793728 -0.52 0.7 -0.198 -0.04 0.7 -2.385 -0.15 0.8

RS17022316 -0.52 0.7 -0.198 -0.04 0.7 -2.385 -0.15 0.8

RS4680800 -0.52 0.7 -0.198 -0.04 0.7 -2.385 -0.15 0.8

RS634868 -0.52 0.7 -0.198 -0.04 0.7 -2.385 -0.15 0.8

RS641725 -0.52 0.7 -0.198 -0.04 0.7 -2.385 -0.15 0.8

RS4680807 -0.52 0.7 -0.198 -0.04 0.7 -2.385 -0.15 0.8

RS11129353 -0.39 0.7 -0.198 -0.03 0.7 -1.361 -0.07 0.7

RS1025644 -0.24 0.6 -0.198 -0.02 0.6 -0.442 -0.02 0.6

RS7624339 -0.20 0.6 -0.198 -0.02 0.6 -0.310 -0.01 0.6

RS6773182 -0.17 0.6 -0.198 -0.01 0.6 -0.233 -0.01 0.6

RS9849677 -0.11 0.5 -0.198 -0.01 0.6 -0.136 -0.00 0.5

RS11713209 -0.11 0.5 -0.198 -0.01 0.6 -0.136 -0.00 0.5

RS4680833 -0.11 0.5 -0.198 -0.01 0.6 -0.136 -0.00 0.5

RS1561374 -0.11 0.5 -0.198 -0.01 0.6 -0.136 -0.00 0.5

RS12489361 -0.11 0.5 -0.198 -0.01 0.6 -0.136 -0.00 0.5

RS9867172 -0.11 0.5 -0.198 -0.01 0.6 -0.136 -0.00 0.5

RS35883 -0.11 0.5 -0.198 -0.01 0.6 -0.136 -0.00 0.5

RS7626062 -0.11 0.5 -0.198 -0.01 0.6 -0.136 -0.00 0.5

RS1371844 -0.11 0.5 -0.198 -0.01 0.6 -0.136 -0.00 0.5

RS10510627 -0.11 0.5 -0.198 -0.01 0.6 -0.136 -0.00 0.5

RS7633961 -0.11 0.5 -0.198 -0.01 0.6 -0.136 -0.00 0.5

RS1449286 -0.11 0.5 -0.198 -0.01 0.6 -0.136 -0.00 0.5

RS7649490 -0.11 0.5 -0.198 -0.01 0.6 -0.136 -0.00 0.5

RS9863443 -0.11 0.5 -0.198 -0.01 0.6 -0.136 -0.00 0.5

RS1036308 -0.11 0.5 -0.198 -0.01 0.6 -0.136 -0.00 0.5

RS6797604 -0.11 0.5 -0.198 -0.01 0.6 -0.136 -0.00 0.5

RS2132298 -0.11 0.5 -0.198 -0.01 0.6 -0.136 -0.00 0.5

RS1495591 -0.11 0.5 -0.198 -0.01 0.6 -0.136 -0.00 0.5

RS7653262 -0.11 0.5 -0.198 -0.01 0.6 -0.136 -0.00 0.5

RS7642647 -0.11 0.5 -0.198 -0.01 0.6 -0.136 -0.00 0.5

RS1078985 -0.19 0.6 -0.198 -0.02 0.6 -0.279 -0.01 0.6

RS1346907 -0.20 0.6 -0.198 -0.02 0.6 -0.314 -0.01 0.6

RS3773658 -0.20 0.6 -0.198 -0.02 0.6 -0.315 -0.01 0.6

RS6767233 -0.21 0.6 -0.198 -0.02 0.6 -0.341 -0.01 0.6

RS2062775 -0.24 0.6 -0.198 -0.02 0.6 -0.433 -0.02 0.6

RS1393752 -0.25 0.6 -0.198 -0.02 0.6 -0.472 -0.02 0.6

RS11129429 -0.30 0.6 -0.198 -0.03 0.6 -0.675 -0.03 0.7

RS1494742 -0.33 0.6 -0.198 -0.03 0.6 -0.822 -0.04 0.7

RS294305 -0.37 0.6 -0.198 -0.03 0.6 -1.029 -0.06 0.7

RS4955185 -0.46 0.7 -0.198 -0.04 0.7 -1.536 -0.10 0.7

RS7646751 -0.46 0.7 -0.198 -0.04 0.7 -1.536 -0.10 0.7

RS13320098 -0.46 0.7 -0.198 -0.04 0.7 -1.536 -0.10 0.7

RS7638704 -0.46 0.7 -0.198 -0.04 0.7 -1.536 -0.10 0.7

RS7641067 -0.46 0.7 -0.198 -0.04 0.7 -1.536 -0.10 0.7

RS7627284 -0.46 0.7 -0.198 -0.04 0.7 -1.536 -0.10 0.7

RS7613225 -0.46 0.7 -0.198 -0.04 0.7 -1.535 -0.10 0.7

RS2168422 -0.46 0.7 -0.198 -0.04 0.7 -1.535 -0.10 0.7

RS4955203 -0.46 0.7 -0.198 -0.04 0.7 -1.535 -0.10 0.7

RS4476542 -0.46 0.7 -0.198 -0.04 0.7 -1.535 -0.10 0.7

RS6807471 -0.46 0.7 -0.198 -0.04 0.7 -1.535 -0.10 0.7

RS6550080 -0.46 0.7 -0.198 -0.04 0.7 -1.535 -0.10 0.7

RS11924760 -0.46 0.7 -0.198 -0.04 0.7 -1.535 -0.10 0.7

RS4444747 -0.46 0.7 -0.198 -0.04 0.7 -1.535 -0.10 0.7

RS12488844 -0.46 0.7 -0.198 -0.04 0.7 -1.536 -0.10 0.7

RS4955239 -0.46 0.7 -0.198 -0.04 0.7 -1.536 -0.10 0.7

RS9839669 -0.46 0.7 -0.198 -0.04 0.7 -1.535 -0.10 0.7

RS4955252 -0.46 0.7 -0.198 -0.04 0.7 -1.535 -0.10 0.7

RS4245886 -0.46 0.7 -0.198 -0.04 0.7 -1.536 -0.10 0.7

RS11129511 -0.46 0.7 -0.198 -0.04 0.7 -1.536 -0.10 0.7

RS9875310 -0.46 0.7 -0.198 -0.04 0.7 -1.536 -0.10 0.7

RS4364205 -0.46 0.7 -0.198 -0.04 0.7 -1.536 -0.10 0.7

RS3853720 -0.46 0.7 -0.198 -0.04 0.7 -1.536 -0.10 0.7

RS6550146 -0.46 0.7 -0.198 -0.04 0.7 -1.536 -0.10 0.7

RS6795737 -0.46 0.7 -0.198 -0.04 0.7 -1.535 -0.10 0.7

RS11129541 -0.46 0.7 -0.198 -0.04 0.7 -1.535 -0.10 0.7

RS6781225 -0.46 0.7 -0.198 -0.04 0.7 -1.535 -0.10 0.7

RS7623768 -0.46 0.7 -0.198 -0.04 0.7 -1.535 -0.10 0.7

RS11709715 -0.46 0.7 -0.198 -0.04 0.7 -1.535 -0.10 0.7

RS4678497 -0.46 0.7 -0.198 -0.04 0.7 -1.535 -0.10 0.7

RS4621286 -0.46 0.7 -0.198 -0.04 0.7 -1.535 -0.10 0.7

RS12493761 -0.46 0.7 -0.198 -0.04 0.7 -1.536 -0.10 0.7

RS13065450 -0.46 0.7 -0.198 -0.04 0.7 -1.535 -0.10 0.7

RS13080359 -0.46 0.7 -0.198 -0.04 0.7 -1.535 -0.10 0.7

RS9848859 -0.46 0.7 -0.198 -0.04 0.7 -1.535 -0.10 0.7

RS11916425 -0.46 0.7 -0.198 -0.04 0.7 -1.535 -0.10 0.7

RS11710273 -0.46 0.7 -0.198 -0.04 0.7 -1.535 -0.10 0.7

RS4072315 -0.46 0.7 -0.198 -0.04 0.7 -1.535 -0.10 0.7

RS4678662 -0.46 0.7 -0.198 -0.04 0.7 -1.535 -0.10 0.7

RS4678665 -0.46 0.7 -0.198 -0.04 0.7 -1.535 -0.10 0.7

RS9838476 -0.46 0.7 -0.198 -0.04 0.7 -1.535 -0.10 0.7

RS7635856 -0.37 0.6 -0.198 -0.03 0.6 -1.434 -0.07 0.7

RS11709953 -0.36 0.6 -0.198 -0.03 0.6 -1.426 -0.07 0.7

RS6774497 -0.36 0.6 -0.198 -0.03 0.6 -1.420 -0.07 0.7

RS11922997 -0.36 0.6 -0.198 -0.03 0.6 -1.417 -0.07 0.7

RS2884658 -0.35 0.6 -0.198 -0.03 0.6 -1.415 -0.07 0.7

RS6779123 -0.35 0.6 -0.198 -0.03 0.6 -1.415 -0.07 0.7

RS6788583 -0.35 0.6 -0.198 -0.03 0.6 -1.409 -0.07 0.7

RS2167176 -0.35 0.6 -0.198 -0.03 0.6 -1.406 -0.07 0.7

RS4678770 -0.35 0.6 -0.198 -0.03 0.6 -1.406 -0.07 0.7

RS6805985 -0.35 0.6 -0.198 -0.03 0.6 -1.404 -0.07 0.7

RS4678802 -0.35 0.6 -0.198 -0.03 0.6 -1.403 -0.07 0.7

RS9847358 -0.35 0.6 -0.198 -0.03 0.6 -1.403 -0.07 0.7

RS10049409 -0.35 0.6 -0.198 -0.03 0.6 -1.403 -0.07 0.7

RS6550381 -0.35 0.6 -0.198 -0.03 0.6 -1.404 -0.07 0.7

RS10510676 -0.35 0.6 -0.198 -0.03 0.6 -1.406 -0.07 0.7

RS772622 -0.35 0.6 -0.198 -0.03 0.6 -1.411 -0.07 0.7

RS10510683 -0.35 0.6 -0.198 -0.03 0.6 -1.415 -0.07 0.7

RS6550429 -0.38 0.6 -0.198 -0.03 0.6 -1.445 -0.08 0.7

RS9843434 -0.38 0.6 -0.198 -0.03 0.6 -1.448 -0.08 0.7

RS906475 -0.38 0.6 -0.198 -0.03 0.6 -1.454 -0.08 0.7

RS3796187 -0.41 0.7 -0.198 -0.03 0.7 -1.482 -0.08 0.7

RS7620657 -0.41 0.7 -0.198 -0.03 0.7 -1.491 -0.09 0.7

RS1472864 -0.46 0.7 -0.198 -0.04 0.7 -1.535 -0.10 0.7

RS7619689 -0.46 0.7 -0.198 -0.04 0.7 -1.535 -0.10 0.7

RS1800734 -0.46 0.7 -0.198 -0.04 0.7 -1.535 -0.10 0.7

RS9823993 -0.46 0.7 -0.198 -0.04 0.7 -1.535 -0.10 0.7

RS6550497 -0.46 0.7 -0.198 -0.04 0.7 -1.535 -0.10 0.7

RS17036845 -0.46 0.7 -0.198 -0.04 0.7 -1.535 -0.10 0.7

RS1984311 -0.46 0.7 -0.198 -0.04 0.7 -1.535 -0.10 0.7

RS9857730 -0.46 0.7 -0.198 -0.04 0.7 -1.535 -0.10 0.7

RS4679028 -0.46 0.7 -0.198 -0.04 0.7 -1.535 -0.10 0.7

RS1002675 -0.46 0.7 -0.198 -0.04 0.7 -1.535 -0.10 0.7

RS6793245 -0.46 0.7 -0.198 -0.04 0.7 -1.535 -0.10 0.7

RS7427874 -0.46 0.7 -0.198 -0.04 0.7 -1.535 -0.10 0.7

RS9853984 -0.46 0.7 -0.198 -0.04 0.7 -1.535 -0.10 0.7

RS7373102 -0.46 0.7 -0.198 -0.04 0.7 -1.535 -0.10 0.7

RS7373154 -0.46 0.7 -0.198 -0.04 0.7 -1.535 -0.10 0.7

RS7374804 -0.46 0.7 -0.198 -0.04 0.7 -1.535 -0.10 0.7

RS9815891 -0.46 0.7 -0.198 -0.04 0.7 -1.535 -0.10 0.7

RS7635472 -0.46 0.7 -0.198 -0.04 0.7 -1.535 -0.10 0.7

RS9311199 -0.46 0.7 -0.198 -0.04 0.7 -1.535 -0.10 0.7

RS784502 -0.46 0.7 -0.198 -0.04 0.7 -1.536 -0.10 0.7

RS4676617 -0.46 0.7 -0.198 -0.04 0.7 -1.535 -0.10 0.7

RS4676487 -0.46 0.7 -0.198 -0.04 0.7 -1.535 -0.10 0.7

RS12107527 -0.46 0.7 -0.198 -0.04 0.7 -1.536 -0.10 0.7

RS17756489 -0.46 0.7 -0.198 -0.04 0.7 -1.536 -0.10 0.7

RS13091037 -0.46 0.7 -0.198 -0.04 0.7 -1.536 -0.10 0.7

RS6797988 -0.46 0.7 -0.198 -0.04 0.7 -1.536 -0.10 0.7

RS2371091 -0.46 0.7 -0.198 -0.04 0.7 -1.536 -0.10 0.7

RS9827374 -0.46 0.7 -0.198 -0.04 0.7 -1.535 -0.10 0.7

RS13060185 -0.46 0.7 -0.198 -0.04 0.7 -1.535 -0.10 0.7

RS13326962 -0.46 0.7 -0.198 -0.04 0.7 -1.535 -0.10 0.7

RS1317317 -0.46 0.7 -0.198 -0.04 0.7 -1.535 -0.10 0.7

RS1317217 -0.46 0.7 -0.198 -0.04 0.7 -1.535 -0.10 0.7

RS6599111 -0.46 0.7 -0.198 -0.04 0.7 -1.535 -0.10 0.7

RS6599129 -0.46 0.7 -0.198 -0.04 0.7 -1.535 -0.10 0.7

RS11716816 -0.46 0.7 -0.198 -0.04 0.7 -1.535 -0.10 0.7

RS17052699 -0.46 0.7 -0.198 -0.04 0.7 -1.535 -0.10 0.7

RS9883523 -0.46 0.7 -0.198 -0.04 0.7 -1.535 -0.10 0.7

RS1722850 -0.46 0.7 -0.198 -0.04 0.7 -1.535 -0.10 0.7

RS749932 -0.46 0.7 -0.198 -0.04 0.7 -1.536 -0.10 0.7

RS4973881 -0.46 0.7 -0.198 -0.04 0.7 -1.536 -0.10 0.7

RS7627367 -0.46 0.7 -0.198 -0.04 0.7 -1.536 -0.10 0.7

RS9311289 -0.46 0.7 -0.198 -0.04 0.7 -1.535 -0.10 0.7

RS2055285 -0.46 0.7 -0.198 -0.04 0.7 -1.536 -0.10 0.7

RS9311317 -0.46 0.7 -0.198 -0.04 0.7 -1.536 -0.10 0.7

RS4377469 -0.46 0.7 -0.198 -0.04 0.7 -1.536 -0.10 0.7

RS11571842 -0.46 0.7 -0.198 -0.04 0.7 -1.536 -0.10 0.7

RS11129960 -0.46 0.7 -0.198 -0.04 0.7 -1.536 -0.10 0.7

RS512848 -0.46 0.7 -0.198 -0.04 0.7 -1.536 -0.10 0.7

RS6441802 -0.46 0.7 -0.198 -0.04 0.7 -1.535 -0.10 0.7

RS11717603 -0.46 0.7 -0.198 -0.04 0.7 -1.535 -0.10 0.7

RS601204 -0.46 0.7 -0.198 -0.04 0.7 -1.535 -0.10 0.7

RS6797545 -0.46 0.7 -0.198 -0.04 0.7 -1.536 -0.10 0.7

RS6793102 -0.46 0.7 -0.198 -0.04 0.7 -1.536 -0.10 0.7

RS11717346 -0.46 0.7 -0.198 -0.04 0.7 -1.536 -0.10 0.7

RS3732860 -0.46 0.7 -0.198 -0.04 0.7 -1.536 -0.10 0.7

RS9874514 -0.46 0.7 -0.198 -0.04 0.7 -1.536 -0.10 0.7

RS513875 -0.46 0.7 -0.198 -0.04 0.7 -1.535 -0.10 0.7

RS7641667 -0.46 0.7 -0.198 -0.04 0.7 -1.535 -0.10 0.7

RS4547674 -0.46 0.7 -0.198 -0.04 0.7 -1.535 -0.10 0.7

RS10490784 -0.46 0.7 -0.198 -0.04 0.7 -1.536 -0.10 0.7

RS4392389 -0.46 0.7 -0.198 -0.04 0.7 -1.536 -0.10 0.7

RS9863510 -0.46 0.7 -0.198 -0.04 0.7 -1.536 -0.10 0.7

RS7612822 -0.46 0.7 -0.198 -0.04 0.7 -1.536 -0.10 0.7

RS3852012 -0.46 0.7 -0.198 -0.04 0.7 -1.535 -0.10 0.7

RS10510742 -0.46 0.7 -0.198 -0.04 0.7 -1.535 -0.10 0.7

RS9855433 -0.46 0.7 -0.198 -0.04 0.7 -1.535 -0.10 0.7

RS3804583 -0.46 0.7 -0.198 -0.04 0.7 -1.535 -0.10 0.7

RS4683055 -0.46 0.7 -0.198 -0.04 0.7 -1.535 -0.10 0.7

RS4683071 -0.45 0.7 -0.198 -0.04 0.7 -1.492 -0.09 0.7

RS7639787 -0.44 0.7 -0.198 -0.04 0.7 -1.433 -0.09 0.7

RS6793847 -0.41 0.7 -0.198 -0.03 0.7 -1.242 -0.07 0.7

RS17637580 -0.39 0.7 -0.198 -0.03 0.6 -1.127 -0.06 0.7

RS11130070 -0.34 0.6 -0.198 -0.03 0.6 -0.869 -0.05 0.7

RS2742417 -0.31 0.6 -0.198 -0.03 0.6 -0.723 -0.04 0.7

RS11712150 -0.16 0.6 -0.198 -0.01 0.6 -0.230 -0.01 0.6

RS883740 -0.12 0.5 -0.198 -0.01 0.6 -0.148 -0.00 0.6

RS11713015 -0.11 0.5 -0.198 -0.01 0.6 -0.136 -0.00 0.5

RS9883208 -0.11 0.5 -0.198 -0.01 0.6 -0.136 -0.00 0.5

RS13353497 -0.11 0.5 -0.198 -0.01 0.6 -0.136 -0.00 0.5

RS1520489 -0.11 0.5 -0.198 -0.01 0.6 -0.136 -0.00 0.5

RS9861101 -0.11 0.5 -0.198 -0.01 0.6 -0.136 -0.00 0.5

RS9847407 -0.11 0.5 -0.198 -0.01 0.6 -0.136 -0.00 0.5

RS7623501 -0.11 0.5 -0.198 -0.01 0.6 -0.136 -0.00 0.5

RS11130112 -0.11 0.5 -0.198 -0.01 0.6 -0.136 -0.00 0.5

RS3732530 -0.11 0.5 -0.198 -0.01 0.6 -0.136 -0.00 0.5

RS9838618 -0.11 0.5 -0.198 -0.01 0.6 -0.136 -0.00 0.5

RS6791542 -0.11 0.5 -0.198 -0.01 0.6 -0.136 -0.00 0.5

RS2005557 -0.11 0.5 -0.198 -0.01 0.6 -0.136 -0.00 0.5

RS6446298 -0.11 0.5 -0.198 -0.01 0.6 -0.136 -0.00 0.5

RS2624834 -0.11 0.5 -0.198 -0.01 0.6 -0.136 -0.00 0.5

RS11711407 -0.11 0.5 -0.198 -0.01 0.6 -0.136 -0.00 0.5

RS762898 -0.11 0.5 -0.198 -0.01 0.6 -0.136 -0.00 0.5

RS2239752 -0.11 0.5 -0.198 -0.01 0.6 -0.136 -0.00 0.5

RS9869826 -0.11 0.5 -0.198 -0.01 0.6 -0.136 -0.00 0.5

RS6773188 -0.11 0.5 -0.198 -0.01 0.6 -0.136 -0.00 0.5

RS1605070 -0.11 0.5 -0.198 -0.01 0.6 -0.136 -0.00 0.5

RS747343 -0.11 0.5 -0.198 -0.01 0.6 -0.136 -0.00 0.5

RS2239547 -0.11 0.5 -0.198 -0.01 0.6 -0.136 -0.00 0.5

RS6763768 -0.11 0.5 -0.198 -0.01 0.6 -0.136 -0.00 0.5

RS3774473 -0.11 0.5 -0.198 -0.01 0.6 -0.136 -0.00 0.5

RS6445597 -0.11 0.5 -0.198 -0.01 0.6 -0.136 -0.00 0.5

RS3796347 -0.11 0.5 -0.198 -0.01 0.6 -0.136 -0.00 0.5

RS2278516 -0.11 0.5 -0.198 -0.01 0.6 -0.136 -0.00 0.5

RS7636856 -0.11 0.5 -0.198 -0.01 0.6 -0.136 -0.00 0.5

RS1868514 -0.11 0.5 -0.198 -0.01 0.6 -0.136 -0.00 0.5

RS12629593 -0.11 0.5 -0.198 -0.01 0.6 -0.136 -0.00 0.5

RS11926147 -0.11 0.5 -0.198 -0.01 0.6 -0.136 -0.00 0.5

RS11130399 -0.11 0.5 -0.198 -0.01 0.6 -0.136 -0.00 0.5

RS11130406 -0.11 0.5 -0.198 -0.01 0.6 -0.136 -0.00 0.5

RS7615792 -0.11 0.5 -0.198 -0.01 0.6 -0.136 -0.00 0.5

RS7619590 -0.11 0.5 -0.198 -0.01 0.6 -0.136 -0.00 0.5

RS7645545 -0.11 0.5 -0.198 -0.01 0.6 -0.136 -0.00 0.5

RS7614229 -0.11 0.5 -0.198 -0.01 0.6 -0.136 -0.00 0.5

RS4955856 -0.11 0.5 -0.198 -0.01 0.6 -0.136 -0.00 0.5

RS1607754 -0.11 0.5 -0.198 -0.01 0.6 -0.136 -0.00 0.5

RS496625 -0.11 0.5 -0.198 -0.01 0.6 -0.136 -0.00 0.5

RS510605 -0.11 0.5 -0.198 -0.01 0.6 -0.136 -0.00 0.5

RS1179911 -0.11 0.5 -0.198 -0.01 0.6 -0.136 -0.00 0.5

RS3773573 -0.11 0.5 -0.198 -0.01 0.6 -0.136 -0.00 0.5

RS11130454 -0.11 0.5 -0.198 -0.01 0.6 -0.136 -0.00 0.5

RS2036487 -0.11 0.5 -0.198 -0.01 0.6 -0.136 -0.00 0.5

RS12491607 -0.11 0.5 -0.198 -0.01 0.6 -0.136 -0.00 0.5

RS374563 -0.11 0.5 -0.198 -0.01 0.6 -0.136 -0.00 0.5

RS422712 -0.11 0.5 -0.198 -0.01 0.6 -0.136 -0.00 0.5

RS443840 -0.11 0.5 -0.198 -0.01 0.6 -0.136 -0.00 0.5

RS458308 -0.11 0.5 -0.198 -0.01 0.6 -0.136 -0.00 0.5

RS2004214 -0.11 0.5 -0.198 -0.01 0.6 -0.136 -0.00 0.5

RS2885453 -0.11 0.5 -0.198 -0.01 0.6 -0.136 -0.00 0.5

RS476986 -0.11 0.5 -0.198 -0.01 0.6 -0.136 -0.00 0.5

RS815460 -0.11 0.5 -0.198 -0.01 0.6 -0.136 -0.00 0.5

RS1126230 -0.11 0.5 -0.198 -0.01 0.6 -0.136 -0.00 0.5

RS9311578 -0.11 0.5 -0.198 -0.01 0.6 -0.136 -0.00 0.5

RS9827757 -0.11 0.5 -0.198 -0.01 0.6 -0.136 -0.00 0.5

RS6766607 -0.11 0.5 -0.198 -0.01 0.6 -0.136 -0.00 0.5

RS9811620 -0.11 0.5 -0.198 -0.01 0.6 -0.136 -0.00 0.5

RS12635403 -0.11 0.5 -0.198 -0.01 0.6 -0.136 -0.00 0.5

RS2200432 -0.11 0.5 -0.198 -0.01 0.6 -0.136 -0.00 0.5

RS9815821 -0.11 0.5 -0.198 -0.01 0.6 -0.136 -0.00 0.5

RS7645578 -0.11 0.5 -0.198 -0.01 0.6 -0.136 -0.00 0.5

RS10212341 -0.11 0.5 -0.198 -0.01 0.6 -0.136 -0.00 0.5

RS3772219 -0.11 0.5 -0.198 -0.01 0.6 -0.136 -0.00 0.5

RS10866002 -0.11 0.5 -0.198 -0.01 0.6 -0.136 -0.00 0.5

RS13062174 -0.11 0.5 -0.198 -0.01 0.6 -0.136 -0.00 0.5

RS6445829 -0.11 0.5 -0.198 -0.01 0.6 -0.136 -0.00 0.5

RS11922193 -0.11 0.5 -0.198 -0.01 0.6 -0.136 -0.00 0.5

RS1553992 -0.11 0.5 -0.198 -0.01 0.6 -0.136 -0.00 0.5

RS13070406 -0.11 0.5 -0.198 -0.01 0.6 -0.136 -0.00 0.5

RS17058156 -0.11 0.5 -0.198 -0.01 0.6 -0.136 -0.00 0.5

RS6445902 -0.11 0.5 -0.198 -0.01 0.6 -0.136 -0.00 0.5

RS2577322 -0.11 0.5 -0.198 -0.01 0.6 -0.136 -0.00 0.5

RS9828717 -0.11 0.5 -0.198 -0.01 0.6 -0.136 -0.00 0.5

RS865726 -0.11 0.5 -0.198 -0.01 0.6 -0.136 -0.00 0.5

RS9855113 -0.11 0.5 -0.198 -0.01 0.6 -0.136 -0.00 0.5

RS4681679 -0.11 0.5 -0.198 -0.01 0.6 -0.136 -0.00 0.5

RS7625655 -0.11 0.5 -0.198 -0.01 0.6 -0.136 -0.00 0.5

RS12488633 -0.11 0.5 -0.198 -0.01 0.6 -0.136 -0.00 0.5

RS6776877 -0.11 0.5 -0.198 -0.01 0.6 -0.136 -0.00 0.5

RS731608 -0.11 0.5 -0.198 -0.01 0.6 -0.136 -0.00 0.5

RS2365401 -0.11 0.5 -0.198 -0.01 0.6 -0.136 -0.00 0.5

RS9880478 -0.11 0.5 -0.198 -0.01 0.6 -0.136 -0.00 0.5

RS4679485 -0.11 0.5 -0.198 -0.01 0.6 -0.136 -0.00 0.5

RS9869521 -0.11 0.5 -0.198 -0.01 0.6 -0.136 -0.00 0.5

RS962791 -0.11 0.5 -0.198 -0.01 0.6 -0.136 -0.00 0.5

RS4679582 -0.11 0.5 -0.198 -0.01 0.6 -0.136 -0.00 0.5

RS12491058 -0.11 0.5 -0.198 -0.01 0.6 -0.136 -0.00 0.5

RS13325549 -0.11 0.5 -0.198 -0.01 0.6 -0.136 -0.00 0.5

RS17359485 -0.11 0.5 -0.198 -0.01 0.6 -0.136 -0.00 0.5

RS4679610 -0.11 0.5 -0.198 -0.01 0.6 -0.136 -0.00 0.5

RS1905866 -0.11 0.5 -0.198 -0.01 0.6 -0.136 -0.00 0.5

RS9810994 -0.11 0.5 -0.198 -0.01 0.6 -0.136 -0.00 0.5

RS4679622 -0.11 0.5 -0.198 -0.01 0.6 -0.136 -0.00 0.5

RS1385816 -0.11 0.5 -0.198 -0.01 0.6 -0.136 -0.00 0.5

RS3772482 -0.11 0.5 -0.198 -0.01 0.6 -0.136 -0.00 0.5

RS9311745 -0.11 0.5 -0.198 -0.01 0.6 -0.136 -0.00 0.5

RS11711227 -0.11 0.5 -0.198 -0.01 0.6 -0.136 -0.00 0.5

RS10510828 -0.11 0.5 -0.198 -0.01 0.6 -0.136 -0.00 0.5

RS2064020 -0.11 0.5 -0.198 -0.01 0.6 -0.136 -0.00 0.5

RS213323 -0.11 0.5 -0.198 -0.01 0.6 -0.136 -0.00 0.5

RS4679524 -0.11 0.5 -0.198 -0.01 0.6 -0.136 -0.00 0.5

RS6762886 -0.11 0.5 -0.198 -0.01 0.6 -0.136 -0.00 0.5

RS9878437 -0.11 0.5 -0.198 -0.01 0.6 -0.136 -0.00 0.5

RS12489534 -0.11 0.5 -0.198 -0.01 0.6 -0.136 -0.00 0.5

RS2734361 -0.11 0.5 -0.198 -0.01 0.6 -0.136 -0.00 0.5

RS7644852 -0.11 0.5 -0.198 -0.01 0.6 -0.136 -0.00 0.5

RS6771732 -0.11 0.5 -0.198 -0.01 0.6 -0.136 -0.00 0.5

RS241698 -0.11 0.5 -0.198 -0.01 0.6 -0.136 -0.00 0.5

RS2006807 -0.11 0.5 -0.198 -0.01 0.6 -0.136 -0.00 0.5

RS17031618 -0.11 0.5 -0.198 -0.01 0.6 -0.136 -0.00 0.5

RS17670198 -0.11 0.5 -0.198 -0.01 0.6 -0.136 -0.00 0.5

RS2856076 -0.11 0.5 -0.198 -0.01 0.6 -0.136 -0.00 0.5

RS3920477 -0.11 0.5 -0.198 -0.01 0.6 -0.136 -0.00 0.5

RS2736745 -0.11 0.5 -0.198 -0.01 0.6 -0.136 -0.00 0.5

RS1562521 -0.11 0.5 -0.198 -0.01 0.6 -0.136 -0.00 0.5

RS1562519 -0.11 0.5 -0.198 -0.01 0.6 -0.136 -0.00 0.5

RS1439007 -0.20 0.6 -0.198 -0.02 0.6 -0.319 -0.01 0.6

RS4309721 -0.25 0.6 -0.198 -0.02 0.6 -0.460 -0.02 0.6

RS12632761 -0.31 0.6 -0.198 -0.03 0.6 -0.783 -0.04 0.7

RS9812140 -0.34 0.6 -0.198 -0.03 0.6 -0.975 -0.05 0.7

RS11716449 -0.43 0.7 -0.198 -0.04 0.7 -1.697 -0.09 0.7

RS1023859 -0.44 0.7 -0.198 -0.04 0.7 -1.748 -0.10 0.7

RS7643514 -0.49 0.7 -0.198 -0.04 0.7 -2.173 -0.13 0.8

RS17624623 -0.52 0.7 -0.198 -0.04 0.7 -2.385 -0.15 0.8

RS625163 -0.52 0.7 -0.198 -0.04 0.7 -2.385 -0.15 0.8

RS2680237 -0.52 0.7 -0.198 -0.04 0.7 -2.385 -0.15 0.8

RS6797184 -0.52 0.7 -0.198 -0.04 0.7 -2.385 -0.15 0.8

RS13085295 -0.52 0.7 -0.198 -0.04 0.7 -2.385 -0.15 0.8

RS4688121 -0.52 0.7 -0.198 -0.04 0.7 -2.385 -0.15 0.8

RS10510870 -0.52 0.7 -0.198 -0.04 0.7 -2.385 -0.15 0.8

RS1388613 -0.52 0.7 -0.198 -0.04 0.7 -2.385 -0.15 0.8

RS12630374 -0.55 0.7 -0.198 -0.04 0.7 -2.934 -0.18 0.8

RS3411 -0.58 0.7 -0.198 -0.05 0.7 -3.838 -0.23 0.8

RS2135582 -0.59 0.7 -0.198 -0.05 0.7 -4.191 -0.25 0.9

RS2367315 -0.60 0.7 -0.198 -0.05 0.7 -4.397 -0.26 0.9

RS7610255 -0.60 0.7 -0.198 -0.05 0.7 -4.396 -0.26 0.9

RS4688376 -0.60 0.7 -0.198 -0.05 0.7 -4.397 -0.26 0.9

RS6445317 -0.60 0.7 -0.198 -0.05 0.7 -4.397 -0.26 0.9

RS7635111 -0.60 0.7 -0.198 -0.05 0.7 -4.397 -0.26 0.9

RS12491231 -0.60 0.7 -0.198 -0.05 0.7 -4.396 -0.26 0.9

RS7648383 -0.60 0.7 -0.198 -0.05 0.7 -4.396 -0.26 0.9

RS11709894 -0.60 0.7 -0.198 -0.05 0.7 -4.396 -0.26 0.9

RS1562859 -0.60 0.7 -0.198 -0.05 0.7 -4.396 -0.26 0.9

RS1839733 -0.60 0.7 -0.198 -0.05 0.7 -4.396 -0.26 0.9

RS17068745 -0.60 0.7 -0.198 -0.05 0.7 -4.396 -0.26 0.9

RS1394898 -0.60 0.7 -0.198 -0.05 0.7 -4.396 -0.26 0.9

RS6796563 -0.60 0.7 -0.198 -0.05 0.7 -4.396 -0.26 0.9

RS751191 -0.60 0.7 -0.198 -0.05 0.7 -4.396 -0.26 0.9

RS6764278 -0.60 0.7 -0.198 -0.05 0.7 -4.396 -0.26 0.9

RS6797570 -0.60 0.7 -0.198 -0.05 0.7 -4.396 -0.26 0.9

RS166229 -0.60 0.7 -0.198 -0.05 0.7 -4.396 -0.26 0.9

RS1096170 -0.60 0.7 -0.198 -0.05 0.7 -4.396 -0.26 0.9

RS696023 -0.60 0.7 -0.198 -0.05 0.7 -4.396 -0.26 0.9

RS12490122 -0.60 0.7 -0.198 -0.05 0.7 -4.397 -0.26 0.9

RS9833903 -0.60 0.7 -0.198 -0.05 0.7 -4.396 -0.26 0.9

RS7635505 -0.60 0.7 -0.198 -0.05 0.7 -4.396 -0.26 0.9

RS12638651 -0.60 0.7 -0.198 -0.05 0.7 -4.396 -0.26 0.9

RS2371766 -0.60 0.7 -0.198 -0.05 0.7 -4.396 -0.26 0.9

RS9830285 -0.60 0.7 -0.198 -0.05 0.7 -4.396 -0.26 0.9

RS704457 -0.60 0.7 -0.198 -0.05 0.7 -4.396 -0.26 0.9

RS12054271 -0.60 0.7 -0.198 -0.05 0.7 -4.396 -0.26 0.9

RS6773617 -0.60 0.7 -0.198 -0.05 0.7 -4.396 -0.26 0.9

RS9311933 -0.60 0.7 -0.198 -0.05 0.7 -4.396 -0.26 0.9

RS6807775 -0.60 0.7 -0.198 -0.05 0.7 -4.396 -0.26 0.9

RS931233 -0.60 0.7 -0.198 -0.05 0.7 -4.396 -0.26 0.9

RS9828000 -0.60 0.7 -0.198 -0.05 0.7 -4.396 -0.26 0.9

RS1109195 -0.60 0.7 -0.198 -0.05 0.7 -4.397 -0.26 0.9

RS13095497 -0.60 0.7 -0.198 -0.05 0.7 -4.397 -0.26 0.9

RS4688577 -0.60 0.7 -0.198 -0.05 0.7 -4.396 -0.26 0.9

RS12634999 -0.60 0.7 -0.198 -0.05 0.7 -4.396 -0.26 0.9

RS9880851 -0.60 0.7 -0.198 -0.05 0.7 -4.396 -0.26 0.9

RS9859634 -0.60 0.7 -0.198 -0.05 0.7 -4.396 -0.26 0.9

RS4437178 -0.60 0.7 -0.198 -0.05 0.7 -4.396 -0.26 0.9

RS6782678 -0.60 0.7 -0.198 -0.05 0.7 -4.396 -0.26 0.9

RS264083 -0.60 0.7 -0.198 -0.05 0.7 -4.396 -0.26 0.9

RS17073750 -0.60 0.7 -0.198 -0.05 0.7 -4.396 -0.26 0.9

RS13063316 -0.60 0.7 -0.198 -0.05 0.7 -4.396 -0.26 0.9

RS264079 -0.60 0.7 -0.198 -0.05 0.7 -4.396 -0.26 0.9

RS1874320 -0.60 0.7 -0.198 -0.05 0.7 -4.396 -0.26 0.9

RS2643727 -0.60 0.7 -0.198 -0.05 0.7 -4.396 -0.26 0.9

RS1680394 -0.60 0.7 -0.198 -0.05 0.7 -4.395 -0.26 0.9

RS782733 -0.60 0.7 -0.198 -0.05 0.7 -4.395 -0.26 0.9

RS4443106 -0.60 0.7 -0.198 -0.05 0.7 -4.396 -0.26 0.9

RS9862866 -0.60 0.7 -0.198 -0.05 0.7 -4.396 -0.26 0.9

RS7638955 -0.60 0.7 -0.198 -0.05 0.7 -4.396 -0.26 0.9

RS17045031 -0.60 0.7 -0.198 -0.05 0.7 -4.396 -0.26 0.9

RS7613063 -0.60 0.7 -0.198 -0.05 0.7 -4.396 -0.26 0.9

RS6796957 -0.60 0.7 -0.198 -0.05 0.7 -4.396 -0.26 0.9

RS7633975 -0.60 0.7 -0.198 -0.05 0.7 -4.396 -0.26 0.9

RS4487196 -0.60 0.7 -0.198 -0.05 0.7 -4.396 -0.26 0.9

RS11128375 -0.60 0.7 -0.198 -0.05 0.7 -4.396 -0.26 0.9

RS6549715 -0.60 0.7 -0.198 -0.05 0.7 -4.396 -0.26 0.9

RS6549776 -0.60 0.7 -0.198 -0.05 0.7 -4.396 -0.26 0.9

RS12485939 -0.60 0.7 -0.198 -0.05 0.7 -4.396 -0.26 0.9

RS13095630 -0.60 0.7 -0.198 -0.05 0.7 -4.396 -0.26 0.9

RS4856863 -0.60 0.7 -0.198 -0.05 0.7 -4.396 -0.26 0.9

RS4130105 -0.60 0.7 -0.198 -0.05 0.7 -4.396 -0.26 0.9

RS9851908 -0.60 0.7 -0.198 -0.05 0.7 -4.396 -0.26 0.9

RS11717561 -0.60 0.7 -0.198 -0.05 0.7 -4.396 -0.26 0.9

RS2362440 -0.60 0.7 -0.198 -0.05 0.7 -4.396 -0.26 0.9

RS6787449 -0.60 0.7 -0.198 -0.05 0.7 -4.396 -0.26 0.9

RS6548962 -0.60 0.7 -0.198 -0.05 0.7 -4.396 -0.26 0.9

RS12487860 -0.60 0.7 -0.198 -0.05 0.7 -4.396 -0.26 0.9

RS17047387 -0.60 0.7 -0.198 -0.05 0.7 -4.396 -0.26 0.9

RS6549098 -0.60 0.7 -0.198 -0.05 0.7 -4.396 -0.26 0.9

RS17047423 -0.60 0.7 -0.198 -0.05 0.7 -4.396 -0.26 0.9

RS1039878 -0.60 0.7 -0.198 -0.05 0.7 -4.396 -0.26 0.9

RS1039879 -0.60 0.7 -0.198 -0.05 0.7 -4.396 -0.26 0.9

RS1394251 -0.60 0.7 -0.198 -0.05 0.7 -4.396 -0.26 0.9

RS902247 -0.60 0.7 -0.198 -0.05 0.7 -4.396 -0.26 0.9

RS4855544 -0.60 0.7 -0.198 -0.05 0.7 -4.396 -0.26 0.9

RS9812253 -0.60 0.7 -0.198 -0.05 0.7 -4.396 -0.26 0.9

RS11128111 -0.60 0.7 -0.198 -0.05 0.7 -4.396 -0.26 0.9

RS4855550 -0.60 0.7 -0.198 -0.05 0.7 -4.396 -0.26 0.9

RS9847661 -0.60 0.7 -0.198 -0.05 0.7 -4.396 -0.26 0.9

RS4855381 -0.60 0.7 -0.198 -0.05 0.7 -4.396 -0.26 0.9

RS7635662 -0.60 0.7 -0.198 -0.05 0.7 -4.396 -0.26 0.9

RS986204 -0.60 0.7 -0.198 -0.05 0.7 -4.396 -0.26 0.9

RS17354320 -0.60 0.7 -0.198 -0.05 0.7 -4.397 -0.26 0.9

RS6549238 -0.60 0.7 -0.198 -0.05 0.7 -4.397 -0.26 0.9

RS1121758 -0.60 0.7 -0.198 -0.05 0.7 -4.397 -0.26 0.9

RS6792167 -0.60 0.7 -0.198 -0.05 0.7 -4.397 -0.26 0.9

RS4855446 -0.60 0.7 -0.198 -0.05 0.7 -4.397 -0.26 0.9

RS7614990 -0.60 0.7 -0.198 -0.05 0.7 -4.397 -0.26 0.9

RS7432941 -0.60 0.7 -0.198 -0.05 0.7 -4.397 -0.26 0.9

RS9846850 -0.60 0.7 -0.198 -0.05 0.7 -4.397 -0.26 0.9

RS6549329 -0.60 0.7 -0.198 -0.05 0.7 -4.397 -0.26 0.9

RS9833791 -0.60 0.7 -0.198 -0.05 0.7 -4.396 -0.26 0.9

RS1491610 -0.60 0.7 -0.198 -0.05 0.7 -4.396 -0.26 0.9

RS11927332 -0.60 0.7 -0.198 -0.05 0.7 -4.396 -0.26 0.9

RS1288974 -0.60 0.7 -0.198 -0.05 0.7 -4.396 -0.26 0.9

RS9840529 -0.60 0.7 -0.198 -0.05 0.7 -4.397 -0.26 0.9

RS17655393 -0.60 0.7 -0.198 -0.05 0.7 -4.396 -0.26 0.9

RS6773658 -0.60 0.7 -0.198 -0.05 0.7 -4.396 -0.26 0.9

RS6782714 -0.60 0.7 -0.198 -0.05 0.7 -4.396 -0.26 0.9

RS1447899 -0.60 0.7 -0.198 -0.05 0.7 -4.397 -0.26 0.9

RS2322143 -0.60 0.7 -0.198 -0.05 0.7 -4.397 -0.26 0.9

RS7629632 -0.60 0.7 -0.198 -0.05 0.7 -4.396 -0.26 0.9

RS7648163 -0.60 0.7 -0.198 -0.05 0.7 -4.396 -0.26 0.9

RS6802036 -0.60 0.7 -0.198 -0.05 0.7 -4.396 -0.26 0.9

RS1109569 -0.60 0.7 -0.198 -0.05 0.7 -4.396 -0.26 0.9

RS9874496 -0.60 0.7 -0.198 -0.05 0.7 -4.396 -0.26 0.9

RS924961 -0.60 0.7 -0.198 -0.05 0.7 -4.396 -0.26 0.9

RS6784559 -0.60 0.7 -0.198 -0.05 0.7 -4.396 -0.26 0.9

RS4677109 -0.60 0.7 -0.198 -0.05 0.7 -4.396 -0.26 0.9

RS4464415 -0.60 0.7 -0.198 -0.05 0.7 -4.396 -0.26 0.9

RS7429249 -0.60 0.7 -0.198 -0.05 0.7 -4.396 -0.26 0.9

RS17009723 -0.60 0.7 -0.198 -0.05 0.7 -4.396 -0.26 0.9

RS9870697 -0.60 0.7 -0.198 -0.05 0.7 -4.396 -0.26 0.9

RS9849164 -0.60 0.7 -0.198 -0.05 0.7 -4.396 -0.26 0.9

RS4677179 -0.60 0.7 -0.198 -0.05 0.7 -4.396 -0.26 0.9

RS8179915 -0.60 0.7 -0.198 -0.05 0.7 -4.396 -0.26 0.9

RS11708505 -0.60 0.7 -0.198 -0.05 0.7 -4.396 -0.26 0.9

RS4676894 -0.60 0.7 -0.198 -0.05 0.7 -4.396 -0.26 0.9

RS6807627 -0.60 0.7 -0.198 -0.05 0.7 -4.396 -0.26 0.9

RS13080133 -0.62 0.7 -0.198 -0.05 0.7 -5.686 -0.31 0.9

RS4585135 -0.62 0.7 -0.198 -0.05 0.7 -5.712 -0.31 0.9

RS17010333 -0.63 0.7 -0.198 -0.05 0.7 -6.491 -0.34 0.9

RS9870408 -0.65 0.7 -0.198 -0.05 0.7 -7.257 -0.38 0.9

RS2322847 -0.65 0.7 -0.198 -0.05 0.7 -7.257 -0.38 0.9

RS9846109 -0.65 0.7 -0.198 -0.05 0.7 -7.257 -0.38 0.9

RS6772476 -0.65 0.7 -0.198 -0.05 0.7 -7.257 -0.38 0.9

RS17751397 -0.65 0.7 -0.198 -0.05 0.7 -7.257 -0.38 0.9

RS12638528 -0.65 0.7 -0.198 -0.05 0.7 -7.257 -0.38 0.9

RS1482171 -0.65 0.7 -0.198 -0.05 0.7 -7.258 -0.38 0.9

RS10865676 -0.65 0.7 -0.198 -0.05 0.7 -7.258 -0.38 0.9

RS13319277 -0.65 0.7 -0.198 -0.05 0.7 -7.258 -0.38 0.9

RS11128332 -0.65 0.7 -0.198 -0.05 0.7 -7.258 -0.38 0.9

RS7648118 -0.65 0.7 -0.198 -0.05 0.7 -7.258 -0.38 0.9

RS953582 -0.65 0.7 -0.198 -0.05 0.7 -7.258 -0.38 0.9

RS4676952 -0.65 0.7 -0.198 -0.05 0.7 -7.257 -0.38 0.9

RS4392360 -0.65 0.7 -0.198 -0.05 0.7 -7.257 -0.38 0.9

RS6799987 -0.65 0.7 -0.198 -0.05 0.7 -7.257 -0.38 0.9

RS2323206 -0.65 0.7 -0.198 -0.05 0.7 -7.257 -0.38 0.9

RS13075070 -0.65 0.7 -0.198 -0.05 0.7 -7.256 -0.38 0.9

RS13098451 -0.65 0.7 -0.198 -0.05 0.7 -7.257 -0.38 0.9

RS7631363 -0.65 0.7 -0.198 -0.05 0.7 -7.257 -0.38 0.9

RS7651173 -0.65 0.7 -0.198 -0.05 0.7 -7.257 -0.38 0.9

RS589344 -0.65 0.7 -0.198 -0.05 0.7 -7.257 -0.38 0.9

RS1579905 -0.65 0.7 -0.198 -0.05 0.7 -7.257 -0.38 0.9

RS13067637 -0.65 0.7 -0.198 -0.05 0.7 -7.257 -0.38 0.9

RS4428227 -0.65 0.7 -0.198 -0.05 0.7 -7.256 -0.38 0.9

RS6809268 -0.65 0.7 -0.198 -0.05 0.7 -7.256 -0.38 0.9

RS6765044 -0.65 0.7 -0.198 -0.05 0.7 -7.257 -0.38 0.9

RS4856003 -0.65 0.7 -0.198 -0.05 0.7 -7.258 -0.38 0.9

RS3913581 -0.65 0.7 -0.198 -0.05 0.7 -7.256 -0.38 0.9

RS4645140 -0.65 0.7 -0.198 -0.05 0.7 -7.256 -0.38 0.9

RS9851014 -0.65 0.7 -0.198 -0.05 0.7 -7.256 -0.38 0.9

RS3849508 -0.65 0.7 -0.198 -0.05 0.7 -7.256 -0.38 0.9

RS1167035 -0.65 0.7 -0.198 -0.05 0.7 -7.257 -0.38 0.9

RS7618823 -0.65 0.7 -0.198 -0.05 0.7 -7.257 -0.38 0.9

RS1146021 -0.65 0.7 -0.198 -0.05 0.7 -7.257 -0.38 0.9

RS4585239 -0.65 0.7 -0.198 -0.05 0.7 -7.257 -0.38 0.9

RS7427534 -0.65 0.7 -0.198 -0.05 0.7 -7.257 -0.38 0.9

RS4074163 -0.65 0.7 -0.198 -0.05 0.7 -7.257 -0.38 0.9

RS6769992 -0.65 0.7 -0.198 -0.05 0.7 -7.257 -0.38 0.9

RS1441951 -0.65 0.7 -0.198 -0.05 0.7 -7.257 -0.38 0.9

RS9882239 -0.65 0.7 -0.198 -0.05 0.7 -7.257 -0.38 0.9

RS7629183 -0.65 0.7 -0.198 -0.05 0.7 -7.257 -0.38 0.9

RS9872416 -0.65 0.7 -0.198 -0.05 0.7 -7.257 -0.38 0.9

RS4684011 -0.65 0.7 -0.198 -0.05 0.7 -7.257 -0.38 0.9

RS1470569 -0.65 0.7 -0.198 -0.05 0.7 -7.257 -0.38 0.9

RS1666130 -0.65 0.7 -0.198 -0.05 0.7 -7.257 -0.38 0.9

RS876675 -0.65 0.7 -0.198 -0.05 0.7 -7.257 -0.38 0.9

RS1163750 -0.65 0.7 -0.198 -0.05 0.7 -7.257 -0.38 0.9

RS13091812 -0.65 0.7 -0.198 -0.05 0.7 -7.257 -0.38 0.9

RS2872373 -0.65 0.7 -0.198 -0.05 0.7 -7.257 -0.38 0.9

RS11127617 -0.65 0.7 -0.198 -0.05 0.7 -7.257 -0.38 0.9

RS1512493 -0.65 0.7 -0.198 -0.05 0.7 -7.257 -0.38 0.9

RS2068704 -0.65 0.7 -0.198 -0.05 0.7 -7.257 -0.38 0.9

RS9839790 -0.65 0.7 -0.198 -0.05 0.7 -7.257 -0.38 0.9

RS17016763 -0.65 0.7 -0.198 -0.05 0.7 -7.257 -0.38 0.9

RS4680962 -0.65 0.7 -0.198 -0.05 0.7 -7.257 -0.38 0.9

RS4680967 -0.65 0.7 -0.198 -0.05 0.7 -7.257 -0.38 0.9

RS1502288 -0.65 0.7 -0.198 -0.05 0.7 -7.257 -0.38 0.9

RS11719555 -0.65 0.7 -0.198 -0.05 0.7 -7.257 -0.38 0.9

RS9867579 -0.65 0.7 -0.198 -0.05 0.7 -7.257 -0.38 0.9

RS6548699 -0.65 0.7 -0.198 -0.05 0.7 -7.257 -0.38 0.9

RS4856330 -0.65 0.7 -0.198 -0.05 0.7 -7.257 -0.38 0.9

RS6802413 -0.65 0.7 -0.198 -0.05 0.7 -7.257 -0.38 0.9

RS7611835 -0.65 0.7 -0.198 -0.05 0.7 -7.257 -0.38 0.9

RS7650907 -0.65 0.7 -0.198 -0.05 0.7 -7.257 -0.38 0.9

RS1461612 -0.65 0.7 -0.198 -0.05 0.7 -7.257 -0.38 0.9

RS7645184 -0.65 0.7 -0.198 -0.05 0.7 -7.257 -0.38 0.9

RS275356 -0.65 0.7 -0.198 -0.05 0.7 -7.257 -0.38 0.9

RS17018940 -0.65 0.7 -0.198 -0.05 0.7 -7.257 -0.38 0.9

RS1524557 -0.65 0.7 -0.198 -0.05 0.7 -7.257 -0.38 0.9

RS1992983 -0.65 0.7 -0.198 -0.05 0.7 -7.257 -0.38 0.9

RS6548823 -0.65 0.7 -0.198 -0.05 0.7 -7.257 -0.38 0.9

RS834864 -0.65 0.7 -0.198 -0.05 0.7 -7.257 -0.38 0.9

RS7639667 -0.65 0.7 -0.198 -0.05 0.7 -7.257 -0.38 0.9

RS7619844 -0.65 0.7 -0.198 -0.05 0.7 -7.257 -0.38 0.9

RS12491148 -0.65 0.7 -0.198 -0.05 0.7 -7.257 -0.38 0.9

RS4589949 -0.65 0.7 -0.198 -0.05 0.7 -7.257 -0.38 0.9

RS9990164 -0.65 0.7 -0.198 -0.05 0.7 -7.257 -0.38 0.9

RS4568144 -0.65 0.7 -0.198 -0.05 0.7 -7.257 -0.38 0.9

RS12636524 -0.65 0.7 -0.198 -0.05 0.7 -7.257 -0.38 0.9

RS1614844 -0.65 0.7 -0.198 -0.05 0.7 -7.257 -0.38 0.9

RS17022107 -0.65 0.7 -0.198 -0.05 0.7 -7.257 -0.38 0.9

RS1551213 -0.65 0.7 -0.198 -0.05 0.7 -7.257 -0.38 0.9

RS6780644 -0.65 0.7 -0.198 -0.05 0.7 -7.257 -0.38 0.9

RS10511084 -0.65 0.7 -0.198 -0.05 0.7 -7.257 -0.38 0.9

RS1448610 -0.65 0.7 -0.198 -0.05 0.7 -7.257 -0.38 0.9

RS12494510 -0.65 0.7 -0.198 -0.05 0.7 -7.257 -0.38 0.9

RS1913188 -0.65 0.7 -0.198 -0.05 0.7 -7.257 -0.38 0.9

RS1355686 -0.65 0.7 -0.198 -0.05 0.7 -7.257 -0.38 0.9

RS7623440 -0.65 0.7 -0.198 -0.05 0.7 -7.257 -0.38 0.9

RS9881535 -0.65 0.7 -0.198 -0.05 0.7 -7.257 -0.38 0.9

RS6763673 -0.65 0.7 -0.198 -0.05 0.7 -7.257 -0.38 0.9

RS17024118 -0.65 0.7 -0.198 -0.05 0.7 -7.257 -0.38 0.9

RS4859055 -0.65 0.7 -0.198 -0.05 0.7 -7.257 -0.38 0.9

RS7614061 -0.65 0.7 -0.198 -0.05 0.7 -7.257 -0.38 0.9

RS2102480 -0.65 0.7 -0.198 -0.05 0.7 -7.257 -0.38 0.9

RS2048284 -0.65 0.7 -0.198 -0.05 0.7 -7.257 -0.38 0.9

RS4569679 -0.65 0.7 -0.198 -0.05 0.7 -7.257 -0.38 0.9

RS6799017 -0.65 0.7 -0.198 -0.05 0.7 -7.257 -0.38 0.9

RS9880959 -0.65 0.7 -0.198 -0.05 0.7 -7.257 -0.38 0.9

RS2011414 -0.65 0.7 -0.198 -0.05 0.7 -7.257 -0.38 0.9

RS7645228 -0.65 0.7 -0.198 -0.05 0.7 -7.257 -0.38 0.9

RS11924091 -0.65 0.7 -0.198 -0.05 0.7 -7.257 -0.38 0.9

RS1496522 -0.65 0.7 -0.198 -0.05 0.7 -7.257 -0.38 0.9

RS1496504 -0.65 0.7 -0.198 -0.05 0.7 -7.257 -0.38 0.9

RS870899 -0.65 0.7 -0.198 -0.05 0.7 -7.257 -0.38 0.9

RS6777442 -0.65 0.7 -0.198 -0.05 0.7 -7.257 -0.38 0.9

RS1521802 -0.65 0.7 -0.198 -0.05 0.7 -7.257 -0.38 0.9

RS6123 -0.65 0.7 -0.198 -0.05 0.7 -7.257 -0.38 0.9

RS17815316 -0.65 0.7 -0.198 -0.05 0.7 -7.257 -0.38 0.9

RS1352463 -0.65 0.7 -0.198 -0.05 0.7 -7.257 -0.38 0.9

RS13321334 -0.65 0.7 -0.198 -0.05 0.7 -7.257 -0.38 0.9

RS6802456 -0.65 0.7 -0.198 -0.05 0.7 -7.257 -0.38 0.9

RS7620512 -0.65 0.7 -0.198 -0.05 0.7 -7.257 -0.38 0.9

RS9847422 -0.65 0.7 -0.198 -0.05 0.7 -7.257 -0.38 0.9

RS7631830 -0.65 0.7 -0.198 -0.05 0.7 -7.257 -0.38 0.9

RS9856079 -0.65 0.7 -0.198 -0.05 0.7 -7.257 -0.38 0.9

RS13081814 -0.65 0.7 -0.198 -0.05 0.7 -7.257 -0.38 0.9

RS13095700 -0.65 0.7 -0.198 -0.05 0.7 -7.257 -0.38 0.9

RS7615218 -0.65 0.7 -0.198 -0.05 0.7 -7.257 -0.38 0.9

RS4857176 -0.65 0.7 -0.198 -0.05 0.7 -7.257 -0.38 0.9

RS4857185 -0.65 0.7 -0.198 -0.05 0.7 -7.257 -0.38 0.9

RS12490647 -0.65 0.7 -0.198 -0.05 0.7 -7.257 -0.38 0.9

RS10934513 -0.65 0.7 -0.198 -0.05 0.7 -7.257 -0.38 0.9

RS9825558 -0.61 0.7 -0.198 -0.05 0.7 -4.927 -0.28 0.9

RS1598915 -0.60 0.7 -0.198 -0.05 0.7 -4.396 -0.26 0.9

RS301957 -0.60 0.7 -0.198 -0.05 0.7 -4.396 -0.26 0.9

RS9864542 -0.60 0.7 -0.198 -0.05 0.7 -4.396 -0.26 0.9

RS1492030 -0.60 0.7 -0.198 -0.05 0.7 -4.396 -0.26 0.9

RS1826352 -0.60 0.7 -0.198 -0.05 0.7 -4.396 -0.26 0.9

RS1497534 -0.60 0.7 -0.198 -0.05 0.7 -4.396 -0.26 0.9

RS9853906 -0.60 0.7 -0.198 -0.05 0.7 -4.396 -0.26 0.9

RS9844815 -0.60 0.7 -0.198 -0.05 0.7 -4.396 -0.26 0.9

RS10935397 -0.60 0.7 -0.198 -0.05 0.7 -4.396 -0.26 0.9

RS1350790 -0.60 0.7 -0.198 -0.05 0.7 -4.396 -0.26 0.9

RS4857401 -0.60 0.7 -0.198 -0.05 0.7 -4.396 -0.26 0.9

RS6797035 -0.60 0.7 -0.198 -0.05 0.7 -4.396 -0.26 0.9

RS11918855 -0.60 0.7 -0.198 -0.05 0.7 -4.396 -0.26 0.9

RS2250022 -0.60 0.7 -0.198 -0.05 0.7 -4.396 -0.26 0.9

RS1038273 -0.60 0.7 -0.198 -0.05 0.7 -4.396 -0.26 0.9

RS7620833 -0.60 0.7 -0.198 -0.05 0.7 -4.396 -0.26 0.9

RS6800453 -0.60 0.7 -0.198 -0.05 0.7 -4.397 -0.26 0.9

RS9861463 -0.60 0.7 -0.198 -0.05 0.7 -4.397 -0.26 0.9

RS792835 -0.52 0.7 -0.198 -0.04 0.7 -2.385 -0.15 0.8

RS6797397 -0.52 0.7 -0.198 -0.04 0.7 -2.385 -0.15 0.8

RS764684 -0.52 0.7 -0.198 -0.04 0.7 -2.385 -0.15 0.8

RS973309 -0.52 0.7 -0.198 -0.04 0.7 -2.385 -0.15 0.8

RS4928093 -0.52 0.7 -0.198 -0.04 0.7 -2.385 -0.15 0.8

RS1449308 -0.52 0.7 -0.198 -0.04 0.7 -2.385 -0.15 0.8

RS2246096 -0.52 0.7 -0.198 -0.04 0.7 -2.385 -0.15 0.8

RS12492516 -0.52 0.7 -0.198 -0.04 0.7 -2.385 -0.15 0.8

RS6441582 -0.52 0.7 -0.198 -0.04 0.7 -2.385 -0.15 0.8

RS9870022 -0.52 0.7 -0.198 -0.04 0.7 -2.385 -0.15 0.8

RS12638134 -0.52 0.7 -0.198 -0.04 0.7 -2.385 -0.15 0.8

RS2554962 -0.52 0.7 -0.198 -0.04 0.7 -2.385 -0.15 0.8

RS677011 -0.52 0.7 -0.198 -0.04 0.7 -2.385 -0.15 0.8

RS1473857 -0.52 0.7 -0.198 -0.04 0.7 -2.385 -0.15 0.8

RS6783446 -0.52 0.7 -0.198 -0.04 0.7 -2.385 -0.15 0.8

RS771790 -0.52 0.7 -0.198 -0.04 0.7 -2.385 -0.15 0.8

RS12696408 -0.52 0.7 -0.198 -0.04 0.7 -2.385 -0.15 0.8

RS6774331 -0.52 0.7 -0.198 -0.04 0.7 -2.385 -0.15 0.8

RS2619283 -0.52 0.7 -0.198 -0.04 0.7 -2.385 -0.15 0.8

RS11720464 -0.52 0.7 -0.198 -0.04 0.7 -2.385 -0.15 0.8

RS10511198 -0.52 0.7 -0.198 -0.04 0.7 -2.384 -0.15 0.8

RS9870734 -0.52 0.7 -0.198 -0.04 0.7 -2.384 -0.15 0.8

RS1284718 -0.52 0.7 -0.198 -0.04 0.7 -2.384 -0.15 0.8

RS12330616 -0.52 0.7 -0.198 -0.04 0.7 -2.384 -0.15 0.8

RS1241287 -0.52 0.7 -0.198 -0.04 0.7 -2.384 -0.15 0.8

RS6441713 -0.52 0.7 -0.198 -0.04 0.7 -2.383 -0.15 0.8

RS2029659 -0.52 0.7 -0.198 -0.04 0.7 -2.384 -0.15 0.8

RS6788478 -0.52 0.7 -0.198 -0.04 0.7 -2.384 -0.15 0.8

RS11706808 -0.52 0.7 -0.198 -0.04 0.7 -2.384 -0.15 0.8

RS7612581 -0.52 0.7 -0.198 -0.04 0.7 -2.385 -0.15 0.8

RS1379626 -0.52 0.7 -0.198 -0.04 0.7 -2.385 -0.15 0.8

RS12054076 -0.52 0.7 -0.198 -0.04 0.7 -2.385 -0.15 0.8

RS7628112 -0.52 0.7 -0.198 -0.04 0.7 -2.385 -0.15 0.8

RS1345189 -0.52 0.7 -0.198 -0.04 0.7 -2.385 -0.15 0.8

RS2713684 -0.56 0.7 -0.198 -0.05 0.7 -3.392 -0.20 0.8

RS7648727 -0.61 0.7 -0.198 -0.05 0.7 -4.789 -0.28 0.9

RS6768068 -0.61 0.7 -0.198 -0.05 0.7 -4.789 -0.28 0.9

RS1946725 -0.61 0.7 -0.198 -0.05 0.7 -4.789 -0.28 0.9

RS1865784 -0.61 0.7 -0.198 -0.05 0.7 -4.789 -0.28 0.9

RS1503075 -0.61 0.7 -0.198 -0.05 0.7 -4.789 -0.28 0.9

RS7628453 -0.61 0.7 -0.198 -0.05 0.7 -4.789 -0.28 0.9

RS9288800 -0.61 0.7 -0.198 -0.05 0.7 -4.789 -0.28 0.9

RS7613684 -0.61 0.7 -0.198 -0.05 0.7 -4.789 -0.28 0.9

RS16851279 -0.61 0.7 -0.198 -0.05 0.7 -4.789 -0.28 0.9

RS6792801 -0.61 0.7 -0.198 -0.05 0.7 -4.789 -0.28 0.9

RS9857831 -0.61 0.7 -0.198 -0.05 0.7 -4.789 -0.28 0.9

RS1546733 -0.61 0.7 -0.198 -0.05 0.7 -4.789 -0.28 0.9

RS1503920 -0.61 0.7 -0.198 -0.05 0.7 -4.789 -0.28 0.9

RS16851901 -0.61 0.7 -0.198 -0.05 0.7 -4.789 -0.28 0.9

RS16852060 -0.61 0.7 -0.198 -0.05 0.7 -4.789 -0.28 0.9

RS4594651 -0.61 0.7 -0.198 -0.05 0.7 -4.789 -0.28 0.9

RS1445059 -0.61 0.7 -0.198 -0.05 0.7 -4.789 -0.28 0.9

RS2712473 -0.61 0.7 -0.198 -0.05 0.7 -4.789 -0.28 0.9

RS7634049 -0.61 0.7 -0.198 -0.05 0.7 -4.789 -0.28 0.9

RS7613153 -0.61 0.7 -0.198 -0.05 0.7 -4.789 -0.28 0.9

RS11928487 -0.61 0.7 -0.198 -0.05 0.7 -4.789 -0.28 0.9

RS11713414 -0.61 0.7 -0.198 -0.05 0.7 -4.789 -0.28 0.9

RS1402304 -0.61 0.7 -0.198 -0.05 0.7 -4.789 -0.28 0.9

RS1712566 -0.61 0.7 -0.198 -0.05 0.7 -4.789 -0.28 0.9

RS6798375 -0.61 0.7 -0.198 -0.05 0.7 -4.789 -0.28 0.9

RS1283101 -0.61 0.7 -0.198 -0.05 0.7 -4.789 -0.28 0.9

RS1283098 -0.61 0.7 -0.198 -0.05 0.7 -4.789 -0.28 0.9

RS6782559 -0.61 0.7 -0.198 -0.05 0.7 -4.789 -0.28 0.9

RS7630522 -0.61 0.7 -0.198 -0.05 0.7 -4.789 -0.28 0.9

RS9851055 -0.61 0.7 -0.198 -0.05 0.7 -4.789 -0.28 0.9

RS696377 -0.61 0.7 -0.198 -0.05 0.7 -4.789 -0.28 0.9

RS6802043 -0.61 0.7 -0.198 -0.05 0.7 -4.789 -0.28 0.9

RS13097121 -0.61 0.7 -0.198 -0.05 0.7 -4.789 -0.28 0.9

RS709521 -0.60 0.7 -0.198 -0.05 0.7 -4.438 -0.26 0.9

RS9821923 -0.57 0.7 -0.198 -0.05 0.7 -3.399 -0.21 0.8

RS9860959 -0.56 0.7 -0.198 -0.05 0.7 -3.279 -0.20 0.8

RS2116693 -0.56 0.7 -0.198 -0.05 0.7 -3.136 -0.19 0.8

RS11922147 -0.56 0.7 -0.198 -0.05 0.7 -3.136 -0.19 0.8

RS7636263 -0.56 0.7 -0.198 -0.05 0.7 -3.136 -0.19 0.8

RS11919162 -0.56 0.7 -0.198 -0.05 0.7 -3.136 -0.19 0.8

RS2290055 -0.56 0.7 -0.198 -0.05 0.7 -3.136 -0.19 0.8

RS11715374 -0.56 0.7 -0.198 -0.05 0.7 -3.136 -0.19 0.8

RS2399250 -0.56 0.7 -0.198 -0.05 0.7 -3.136 -0.19 0.8

RS2593959 -0.56 0.7 -0.198 -0.05 0.7 -3.136 -0.19 0.8

RS9882896 -0.56 0.7 -0.198 -0.05 0.7 -3.136 -0.19 0.8

RS769544 -0.56 0.7 -0.198 -0.05 0.7 -3.136 -0.19 0.8

RS17499967 -0.56 0.7 -0.198 -0.05 0.7 -3.136 -0.19 0.8

RS10511276 -0.56 0.7 -0.198 -0.05 0.7 -3.136 -0.19 0.8

RS602763 -0.56 0.7 -0.198 -0.05 0.7 -3.136 -0.19 0.8

RS709581 -0.56 0.7 -0.198 -0.05 0.7 -3.136 -0.19 0.8

RS9860143 -0.56 0.7 -0.198 -0.05 0.7 -3.136 -0.19 0.8

RS12636638 -0.56 0.7 -0.198 -0.05 0.7 -3.136 -0.19 0.8

RS2068253 -0.56 0.7 -0.198 -0.05 0.7 -3.136 -0.19 0.8

RS11918532 -0.56 0.7 -0.198 -0.05 0.7 -3.136 -0.19 0.8

RS4682013 -0.56 0.7 -0.198 -0.05 0.7 -3.136 -0.19 0.8

RS7620372 -0.56 0.7 -0.198 -0.05 0.7 -3.136 -0.19 0.8

RS7432694 -0.56 0.7 -0.198 -0.05 0.7 -3.136 -0.19 0.8

RS6783451 -0.56 0.7 -0.198 -0.05 0.7 -3.136 -0.19 0.8

RS4682048 -0.56 0.7 -0.198 -0.05 0.7 -3.136 -0.19 0.8

RS2173318 -0.56 0.7 -0.198 -0.05 0.7 -3.136 -0.19 0.8

RS9868266 -0.56 0.7 -0.198 -0.05 0.7 -3.136 -0.19 0.8

RS13078891 -0.56 0.7 -0.198 -0.05 0.7 -3.136 -0.19 0.8

RS1282948 -0.56 0.7 -0.198 -0.05 0.7 -3.136 -0.19 0.8

RS13062225 -0.56 0.7 -0.198 -0.05 0.7 -3.136 -0.19 0.8

RS10511302 -0.56 0.7 -0.198 -0.05 0.7 -3.136 -0.19 0.8

RS774764 -0.56 0.7 -0.198 -0.05 0.7 -3.136 -0.19 0.8

RS6773206 -0.56 0.7 -0.198 -0.05 0.7 -3.136 -0.19 0.8

RS1317244 -0.56 0.7 -0.198 -0.05 0.7 -3.136 -0.19 0.8

RS9847446 -0.56 0.7 -0.198 -0.05 0.7 -3.136 -0.19 0.8

RS9855848 -0.56 0.7 -0.198 -0.05 0.7 -3.136 -0.19 0.8

RS9870933 -0.56 0.7 -0.198 -0.05 0.7 -3.136 -0.19 0.8

RS6438101 -0.56 0.7 -0.198 -0.05 0.7 -3.136 -0.19 0.8

RS11918522 -0.56 0.7 -0.198 -0.05 0.7 -3.136 -0.19 0.8

RS1387024 -0.56 0.7 -0.198 -0.05 0.7 -3.136 -0.19 0.8

RS16860236 -0.56 0.7 -0.198 -0.05 0.7 -3.136 -0.19 0.8

RS2133946 -0.56 0.7 -0.198 -0.05 0.7 -3.136 -0.19 0.8

RS11918021 -0.56 0.7 -0.198 -0.05 0.7 -3.136 -0.19 0.8

RS10934215 -0.56 0.7 -0.198 -0.05 0.7 -3.136 -0.19 0.8

RS1797626 -0.56 0.7 -0.198 -0.05 0.7 -3.136 -0.19 0.8

RS13086237 -0.56 0.7 -0.198 -0.05 0.7 -3.136 -0.19 0.8

RS6770031 -0.56 0.7 -0.198 -0.05 0.7 -3.136 -0.19 0.8

RS6782058 -0.56 0.7 -0.198 -0.05 0.7 -3.136 -0.19 0.8

RS1875113 -0.56 0.7 -0.198 -0.05 0.7 -3.136 -0.19 0.8

RS4580515 -0.56 0.7 -0.198 -0.05 0.7 -3.136 -0.19 0.8

RS9810754 -0.56 0.7 -0.198 -0.05 0.7 -3.136 -0.19 0.8

RS2669910 -0.56 0.7 -0.198 -0.05 0.7 -3.136 -0.19 0.8

RS3773688 -0.56 0.7 -0.198 -0.05 0.7 -3.136 -0.19 0.8

RS3773683 -0.56 0.7 -0.198 -0.05 0.7 -3.136 -0.19 0.8

RS1469559 -0.56 0.7 -0.198 -0.05 0.7 -3.136 -0.19 0.8

RS3903132 -0.56 0.7 -0.198 -0.05 0.7 -3.136 -0.19 0.8

RS324029 -0.56 0.7 -0.198 -0.05 0.7 -3.136 -0.19 0.8

RS9871676 -0.56 0.7 -0.198 -0.05 0.7 -3.136 -0.19 0.8

RS9883949 -0.56 0.7 -0.198 -0.05 0.7 -3.136 -0.19 0.8

RS2669885 -0.56 0.7 -0.198 -0.05 0.7 -3.136 -0.19 0.8

RS1274262 -0.56 0.7 -0.198 -0.05 0.7 -3.136 -0.19 0.8

RS2140345 -0.56 0.7 -0.198 -0.05 0.7 -3.136 -0.19 0.8

RS6782656 -0.56 0.7 -0.198 -0.05 0.7 -3.135 -0.19 0.8

RS2028248 -0.56 0.7 -0.198 -0.05 0.7 -3.136 -0.19 0.8

RS12488667 -0.56 0.7 -0.198 -0.05 0.7 -3.136 -0.19 0.8

RS2944402 -0.56 0.7 -0.198 -0.05 0.7 -3.136 -0.19 0.8

RS1878219 -0.56 0.7 -0.198 -0.05 0.7 -3.136 -0.19 0.8

RS2724466 -0.56 0.7 -0.198 -0.05 0.7 -3.136 -0.19 0.8

RS7638681 -0.56 0.7 -0.198 -0.05 0.7 -3.136 -0.19 0.8

RS1882012 -0.56 0.7 -0.198 -0.05 0.7 -3.136 -0.19 0.8

RS1996490 -0.56 0.7 -0.198 -0.05 0.7 -3.136 -0.19 0.8

RS9834397 -0.56 0.7 -0.198 -0.05 0.7 -3.136 -0.19 0.8

RS11923597 -0.56 0.7 -0.198 -0.05 0.7 -3.136 -0.19 0.8

RS6790547 -0.56 0.7 -0.198 -0.05 0.7 -3.136 -0.19 0.8

RS9817566 -0.56 0.7 -0.198 -0.05 0.7 -3.136 -0.19 0.8

RS10511360 -0.56 0.7 -0.198 -0.05 0.7 -3.136 -0.19 0.8

RS1914812 -0.56 0.7 -0.198 -0.05 0.7 -3.136 -0.19 0.8

RS693456 -0.56 0.7 -0.198 -0.05 0.7 -3.136 -0.19 0.8

RS1521412 -0.56 0.7 -0.198 -0.05 0.7 -3.136 -0.19 0.8

RS9844832 -0.56 0.7 -0.198 -0.05 0.7 -3.136 -0.19 0.8

RS2137213 -0.56 0.7 -0.198 -0.05 0.7 -3.136 -0.19 0.8

RS3849471 -0.56 0.7 -0.198 -0.05 0.7 -3.136 -0.19 0.8

RS2927280 -0.56 0.7 -0.198 -0.05 0.7 -3.136 -0.19 0.8

RS4616651 -0.56 0.7 -0.198 -0.05 0.7 -3.136 -0.19 0.8

RS7644537 -0.56 0.7 -0.198 -0.05 0.7 -3.136 -0.19 0.8

RS6764568 -0.56 0.7 -0.198 -0.05 0.7 -3.136 -0.19 0.8

RS4320082 -0.56 0.7 -0.198 -0.05 0.7 -3.136 -0.19 0.8

RS9861022 -0.56 0.7 -0.198 -0.05 0.7 -3.136 -0.19 0.8

RS11915336 -0.56 0.7 -0.198 -0.05 0.7 -3.136 -0.19 0.8

RS4688078 -0.56 0.7 -0.198 -0.05 0.7 -3.136 -0.19 0.8

RS4688095 -0.56 0.7 -0.198 -0.05 0.7 -3.136 -0.19 0.8

RS1447594 -0.56 0.7 -0.198 -0.05 0.7 -3.136 -0.19 0.8

RS524438 -0.56 0.7 -0.198 -0.05 0.7 -3.136 -0.19 0.8

RS17676122 -0.56 0.7 -0.198 -0.05 0.7 -3.136 -0.19 0.8

RS9831518 -0.56 0.7 -0.198 -0.05 0.7 -3.136 -0.19 0.8

RS705236 -0.56 0.7 -0.198 -0.05 0.7 -3.136 -0.19 0.8

RS881604 -0.56 0.7 -0.198 -0.05 0.7 -3.136 -0.19 0.8

RS1081896 -0.56 0.7 -0.198 -0.05 0.7 -3.136 -0.19 0.8

RS2649881 -0.56 0.7 -0.198 -0.05 0.7 -3.136 -0.19 0.8

RS4687835 -0.56 0.7 -0.198 -0.05 0.7 -3.136 -0.19 0.8

RS12636639 -0.56 0.7 -0.198 -0.05 0.7 -3.136 -0.19 0.8

RS7610546 -0.56 0.7 -0.198 -0.05 0.7 -3.136 -0.19 0.8

RS3195852 -0.56 0.7 -0.198 -0.05 0.7 -3.136 -0.19 0.8

RS7628626 -0.56 0.7 -0.198 -0.05 0.7 -3.136 -0.19 0.8

RS3915166 -0.56 0.7 -0.198 -0.05 0.7 -3.136 -0.19 0.8

RS2280580 -0.56 0.7 -0.198 -0.05 0.7 -3.136 -0.19 0.8

RS3814057 -0.56 0.7 -0.198 -0.05 0.7 -3.136 -0.19 0.8

RS4563433 -0.56 0.7 -0.198 -0.05 0.7 -3.136 -0.19 0.8

RS12489527 -0.56 0.7 -0.198 -0.05 0.7 -3.136 -0.19 0.8

RS11710115 -0.56 0.7 -0.198 -0.05 0.7 -3.136 -0.19 0.8

RS16831033 -0.56 0.7 -0.198 -0.05 0.7 -3.136 -0.19 0.8

RS13075278 -0.56 0.7 -0.198 -0.05 0.7 -3.136 -0.19 0.8

RS6787364 -0.56 0.7 -0.198 -0.05 0.7 -3.136 -0.19 0.8

RS399491 -0.56 0.7 -0.198 -0.05 0.7 -3.136 -0.19 0.8

RS9848328 -0.56 0.7 -0.198 -0.05 0.7 -3.136 -0.19 0.8

RS12489855 -0.56 0.7 -0.198 -0.05 0.7 -3.136 -0.19 0.8

RS9878946 -0.56 0.7 -0.198 -0.05 0.7 -3.136 -0.19 0.8

RS9810670 -0.56 0.7 -0.198 -0.05 0.7 -3.136 -0.19 0.8

RS4482697 -0.56 0.7 -0.198 -0.05 0.7 -3.136 -0.19 0.8

RS17203397 -0.56 0.7 -0.198 -0.05 0.7 -3.136 -0.19 0.8

RS2681420 -0.56 0.7 -0.198 -0.05 0.7 -3.136 -0.19 0.8

RS7648041 -0.56 0.7 -0.198 -0.05 0.7 -3.136 -0.19 0.8

RS4677948 -0.56 0.7 -0.198 -0.05 0.7 -3.136 -0.19 0.8

RS1802757 -0.56 0.7 -0.198 -0.05 0.7 -3.136 -0.19 0.8

RS17589 -0.56 0.7 -0.198 -0.05 0.7 -3.136 -0.19 0.8

RS1961957 -0.56 0.7 -0.198 -0.05 0.7 -3.136 -0.19 0.8

RS6796228 -0.56 0.7 -0.198 -0.05 0.7 -3.136 -0.19 0.8

RS1909808 -0.56 0.7 -0.198 -0.05 0.7 -3.136 -0.19 0.8

RS6726 -0.56 0.7 -0.198 -0.05 0.7 -3.136 -0.19 0.8

RS7647957 -0.56 0.7 -0.198 -0.05 0.7 -3.136 -0.19 0.8

RS12635724 -0.56 0.7 -0.198 -0.05 0.7 -3.136 -0.19 0.8

RS9816759 -0.56 0.7 -0.198 -0.05 0.7 -3.136 -0.19 0.8

RS9880238 -0.56 0.7 -0.198 -0.05 0.7 -3.136 -0.19 0.8

RS836871 -0.56 0.7 -0.198 -0.05 0.7 -3.136 -0.19 0.8

RS4677887 -0.56 0.7 -0.198 -0.05 0.7 -3.136 -0.19 0.8

RS9841477 -0.56 0.7 -0.198 -0.05 0.7 -3.136 -0.19 0.8

RS1055137 -0.56 0.7 -0.198 -0.05 0.7 -3.136 -0.19 0.8

RS40305 -0.56 0.7 -0.198 -0.05 0.7 -3.136 -0.19 0.8

RS2124508 -0.56 0.7 -0.198 -0.05 0.7 -3.136 -0.19 0.8

RS1920623 -0.56 0.7 -0.198 -0.05 0.7 -3.136 -0.19 0.8

RS987613 -0.56 0.7 -0.198 -0.05 0.7 -3.136 -0.19 0.8

RS3772753 -0.56 0.7 -0.198 -0.05 0.7 -3.136 -0.19 0.8

RS556031 -0.56 0.7 -0.198 -0.05 0.7 -3.136 -0.19 0.8

RS1392912 -0.56 0.7 -0.198 -0.05 0.7 -3.136 -0.19 0.8

RS333352 -0.56 0.7 -0.198 -0.05 0.7 -3.136 -0.19 0.8

RS16835912 -0.56 0.7 -0.198 -0.05 0.7 -3.136 -0.19 0.8

RS3772836 -0.56 0.7 -0.198 -0.05 0.7 -3.136 -0.19 0.8

RS9968182 -0.56 0.7 -0.198 -0.05 0.7 -3.136 -0.19 0.8

RS2948791 -0.44 0.7 -0.198 -0.04 0.7 -1.615 -0.09 0.7

RS2333033 -0.42 0.7 -0.198 -0.03 0.7 -1.441 -0.08 0.7

RS2877839 -0.41 0.7 -0.198 -0.03 0.7 -1.297 -0.07 0.7

RS6790154 -0.36 0.6 -0.198 -0.03 0.6 -0.874 -0.05 0.7

RS2945118 -0.35 0.6 -0.198 -0.03 0.6 -0.849 -0.05 0.7

RS2228677 -0.35 0.6 -0.198 -0.03 0.6 -0.814 -0.04 0.7

RS587926 -0.31 0.6 -0.198 -0.03 0.6 -0.615 -0.03 0.6

RS7638240 -0.31 0.6 -0.198 -0.03 0.6 -0.615 -0.03 0.6

RS7609684 -0.31 0.6 -0.198 -0.03 0.6 -0.615 -0.03 0.6

RS7610077 -0.31 0.6 -0.198 -0.03 0.6 -0.615 -0.03 0.6

RS2001665 -0.31 0.6 -0.198 -0.03 0.6 -0.615 -0.03 0.6

RS2979989 -0.31 0.6 -0.198 -0.03 0.6 -0.615 -0.03 0.6

RS4646754 -0.31 0.6 -0.198 -0.03 0.6 -0.615 -0.03 0.6

RS2290053 -0.31 0.6 -0.198 -0.03 0.6 -0.615 -0.03 0.6

RS13060596 -0.31 0.6 -0.198 -0.03 0.6 -0.615 -0.03 0.6

RS10470321 -0.31 0.6 -0.198 -0.03 0.6 -0.615 -0.03 0.6

RS9825812 -0.31 0.6 -0.198 -0.03 0.6 -0.614 -0.03 0.6

RS9880406 -0.31 0.6 -0.198 -0.03 0.6 -0.613 -0.03 0.6

RS1849900 -0.31 0.6 -0.198 -0.03 0.6 -0.612 -0.03 0.6

RS6801075 -0.31 0.6 -0.198 -0.03 0.6 -0.612 -0.03 0.6

RS4679241 -0.30 0.6 -0.198 -0.03 0.6 -0.612 -0.03 0.6

RS9875507 -0.30 0.6 -0.198 -0.03 0.6 -0.611 -0.03 0.6

RS1799388 -0.30 0.6 -0.198 -0.03 0.6 -0.611 -0.03 0.6

RS16837548 -0.30 0.6 -0.198 -0.03 0.6 -0.611 -0.03 0.6

RS6806049 -0.30 0.6 -0.198 -0.03 0.6 -0.611 -0.03 0.6

RS6789402 -0.30 0.6 -0.198 -0.03 0.6 -0.611 -0.03 0.6

RS11921451 -0.30 0.6 -0.198 -0.03 0.6 -0.611 -0.03 0.6

RS11914882 -0.30 0.6 -0.198 -0.03 0.6 -0.611 -0.03 0.6

RS729154 -0.31 0.6 -0.198 -0.03 0.6 -0.613 -0.03 0.6

RS6414306 -0.31 0.6 -0.198 -0.03 0.6 -0.615 -0.03 0.6

RS11709061 -0.31 0.6 -0.198 -0.03 0.6 -0.615 -0.03 0.6

RS11714815 -0.31 0.6 -0.198 -0.03 0.6 -0.615 -0.03 0.6

RS2336408 -0.31 0.6 -0.198 -0.03 0.6 -0.615 -0.03 0.6

RS7633484 -0.31 0.6 -0.198 -0.03 0.6 -0.615 -0.03 0.6

RS605188 -0.31 0.6 -0.198 -0.03 0.6 -0.615 -0.03 0.6

RS9846048 -0.31 0.6 -0.198 -0.03 0.6 -0.615 -0.03 0.6

RS2217628 -0.31 0.6 -0.198 -0.03 0.6 -0.615 -0.03 0.6

RS9851497 -0.31 0.6 -0.198 -0.03 0.6 -0.615 -0.03 0.6

RS4613470 -0.31 0.6 -0.198 -0.03 0.6 -0.615 -0.03 0.6

RS1697 -0.31 0.6 -0.198 -0.03 0.6 -0.615 -0.03 0.6

RS6775459 -0.31 0.6 -0.198 -0.03 0.6 -0.615 -0.03 0.6

RS1683813 -0.31 0.6 -0.198 -0.03 0.6 -0.615 -0.03 0.6

RS10512711 -0.31 0.6 -0.198 -0.03 0.6 -0.615 -0.03 0.6

RS6805004 -0.31 0.6 -0.198 -0.03 0.6 -0.615 -0.03 0.6

RS9853279 -0.31 0.6 -0.198 -0.03 0.6 -0.615 -0.03 0.6

RS6810203 -0.31 0.6 -0.198 -0.03 0.6 -0.615 -0.03 0.6

RS2811450 -0.31 0.6 -0.198 -0.03 0.6 -0.615 -0.03 0.6

RS907930 -0.31 0.6 -0.198 -0.03 0.6 -0.615 -0.03 0.6

RS11716060 -0.31 0.6 -0.198 -0.03 0.6 -0.615 -0.03 0.6

RS13080253 -0.31 0.6 -0.198 -0.03 0.6 -0.615 -0.03 0.6

RS7649424 -0.31 0.6 -0.198 -0.03 0.6 -0.615 -0.03 0.6

RS2971565 -0.31 0.6 -0.198 -0.03 0.6 -0.615 -0.03 0.6

RS2645961 -0.31 0.6 -0.198 -0.03 0.6 -0.615 -0.03 0.6

RS1508518 -0.31 0.6 -0.198 -0.03 0.6 -0.615 -0.03 0.6

RS1484937 -0.31 0.6 -0.198 -0.03 0.6 -0.615 -0.03 0.6

RS10512789 -0.31 0.6 -0.198 -0.03 0.6 -0.615 -0.03 0.6

RS2208985 -0.31 0.6 -0.198 -0.03 0.6 -0.615 -0.03 0.6

RS2291382 -0.31 0.6 -0.198 -0.03 0.6 -0.615 -0.03 0.6

RS7621659 -0.31 0.6 -0.198 -0.03 0.6 -0.615 -0.03 0.6

RS3891970 -0.31 0.6 -0.198 -0.03 0.6 -0.615 -0.03 0.6

RS16837372 -0.31 0.6 -0.198 -0.03 0.6 -0.615 -0.03 0.6

RS1566695 -0.31 0.6 -0.198 -0.03 0.6 -0.615 -0.03 0.6

RS7637944 -0.31 0.6 -0.198 -0.03 0.6 -0.615 -0.03 0.6

RS17294289 -0.31 0.6 -0.198 -0.03 0.6 -0.615 -0.03 0.6

RS10512824 -0.31 0.6 -0.198 -0.03 0.6 -0.615 -0.03 0.6

RS1112706 -0.31 0.6 -0.198 -0.03 0.6 -0.615 -0.03 0.6

RS10934987 -0.31 0.6 -0.198 -0.03 0.6 -0.615 -0.03 0.6

RS2091650 -0.31 0.6 -0.198 -0.03 0.6 -0.615 -0.03 0.6

RS7630827 -0.31 0.6 -0.198 -0.03 0.6 -0.615 -0.03 0.6

RS884431 -0.31 0.6 -0.198 -0.03 0.6 -0.615 -0.03 0.6

RS4854895 -0.31 0.6 -0.198 -0.03 0.6 -0.615 -0.03 0.6

RS2670104 -0.31 0.6 -0.198 -0.03 0.6 -0.615 -0.03 0.6

RS310001 -0.31 0.6 -0.198 -0.03 0.6 -0.615 -0.03 0.6

RS9873881 -0.31 0.6 -0.198 -0.03 0.6 -0.615 -0.03 0.6

RS747328 -0.31 0.6 -0.198 -0.03 0.6 -0.615 -0.03 0.6

RS6807073 -0.31 0.6 -0.198 -0.03 0.6 -0.615 -0.03 0.6

RS9879280 -0.31 0.6 -0.198 -0.03 0.6 -0.615 -0.03 0.6

RS1464401 -0.31 0.6 -0.198 -0.03 0.6 -0.616 -0.03 0.6

RS12493846 -0.31 0.6 -0.198 -0.03 0.6 -0.615 -0.03 0.6

RS13073410 -0.31 0.6 -0.198 -0.03 0.6 -0.615 -0.03 0.6

RS13059279 -0.31 0.6 -0.198 -0.03 0.6 -0.615 -0.03 0.6

RS4854727 -0.31 0.6 -0.198 -0.03 0.6 -0.615 -0.03 0.6

RS3192149 -0.31 0.6 -0.198 -0.03 0.6 -0.615 -0.03 0.6

RS4854742 -0.31 0.6 -0.198 -0.03 0.6 -0.615 -0.03 0.6

RS8649 -0.31 0.6 -0.198 -0.03 0.6 -0.615 -0.03 0.6

RS940898 -0.31 0.6 -0.198 -0.03 0.6 -0.615 -0.03 0.6

RS6763132 -0.31 0.6 -0.198 -0.03 0.6 -0.615 -0.03 0.6

RS7428024 -0.31 0.6 -0.198 -0.03 0.6 -0.616 -0.03 0.6

RS9834727 -0.31 0.6 -0.198 -0.03 0.6 -0.616 -0.03 0.6

RS4241367 -0.31 0.6 -0.198 -0.03 0.6 -0.616 -0.03 0.6

RS9882590 -0.31 0.6 -0.198 -0.03 0.6 -0.616 -0.03 0.6

RS3846051 -0.31 0.6 -0.198 -0.03 0.6 -0.616 -0.03 0.6

RS13060869 -0.31 0.6 -0.198 -0.03 0.6 -0.615 -0.03 0.6

RS9868348 -0.31 0.6 -0.198 -0.03 0.6 -0.615 -0.03 0.6

RS36198 -0.31 0.6 -0.198 -0.03 0.6 -0.615 -0.03 0.6

RS36064 -0.31 0.6 -0.198 -0.03 0.6 -0.615 -0.03 0.6

RS7625747 -0.31 0.6 -0.198 -0.03 0.6 -0.615 -0.03 0.6

RS6777929 -0.31 0.6 -0.198 -0.03 0.6 -0.615 -0.03 0.6

RS9862078 -0.31 0.6 -0.198 -0.03 0.6 -0.615 -0.03 0.6

RS1501202 -0.31 0.6 -0.198 -0.03 0.6 -0.615 -0.03 0.6

RS11712655 -0.31 0.6 -0.198 -0.03 0.6 -0.615 -0.03 0.6

RS4894293 -0.31 0.6 -0.198 -0.03 0.6 -0.615 -0.03 0.6

RS7613386 -0.31 0.6 -0.198 -0.03 0.6 -0.615 -0.03 0.6

RS1523599 -0.31 0.6 -0.198 -0.03 0.6 -0.615 -0.03 0.6

RS10935178 -0.31 0.6 -0.198 -0.03 0.6 -0.615 -0.03 0.6

RS687339 -0.31 0.6 -0.198 -0.03 0.6 -0.615 -0.03 0.6

RS6791142 -0.31 0.6 -0.198 -0.03 0.6 -0.615 -0.03 0.6

RS6780453 -0.31 0.6 -0.198 -0.03 0.6 -0.615 -0.03 0.6

RS6439680 -0.31 0.6 -0.198 -0.03 0.6 -0.615 -0.03 0.6

RS7624691 -0.31 0.6 -0.198 -0.03 0.6 -0.615 -0.03 0.6

RS9830729 -0.31 0.6 -0.198 -0.03 0.6 -0.615 -0.03 0.6

RS428788 -0.31 0.6 -0.198 -0.03 0.6 -0.615 -0.03 0.6

RS13324482 -0.31 0.6 -0.198 -0.03 0.6 -0.615 -0.03 0.6

RS6777144 -0.31 0.6 -0.198 -0.03 0.6 -0.615 -0.03 0.6

RS6772470 -0.31 0.6 -0.198 -0.03 0.6 -0.615 -0.03 0.6

RS6768778 -0.31 0.6 -0.198 -0.03 0.6 -0.615 -0.03 0.6

RS10490795 -0.31 0.6 -0.198 -0.03 0.6 -0.615 -0.03 0.6

RS2724693 -0.31 0.6 -0.198 -0.03 0.6 -0.615 -0.03 0.6

RS13314286 -0.31 0.6 -0.198 -0.03 0.6 -0.615 -0.03 0.6

RS1199330 -0.31 0.6 -0.198 -0.03 0.6 -0.615 -0.03 0.6

RS7627685 -0.31 0.6 -0.198 -0.03 0.6 -0.615 -0.03 0.6

RS559160 -0.31 0.6 -0.198 -0.03 0.6 -0.615 -0.03 0.6

RS12107422 -0.31 0.6 -0.198 -0.03 0.6 -0.615 -0.03 0.6

RS6775443 -0.31 0.6 -0.198 -0.03 0.6 -0.615 -0.03 0.6

RS6439841 -0.31 0.6 -0.198 -0.03 0.6 -0.615 -0.03 0.6

RS2046965 -0.08 0.5 -0.198 -0.01 0.6 -0.184 -0.00 0.5

RS1602629 0.03 0.5 1.309 0.02 0.4 0.062 0.00 0.5

RS12495890 0.77 0.2 1.309 0.30 0.12 0.508 0.12 0.2

RS7625738 1.36 0.09 1.309 0.44 0.08 0.587 0.23 0.2

RS11720063 1.42 0.08 1.309 0.46 0.07 0.592 0.24 0.15

RS10935353 1.51 0.07 1.309 0.47 0.07 0.599 0.25 0.14

RS970929 1.56 0.06 1.309 0.48 0.07 0.603 0.26 0.14

RS987193 1.58 0.06 1.309 0.49 0.07 0.604 0.26 0.14

RS4305391 1.62 0.05 1.309 0.49 0.07 0.606 0.27 0.13

RS9844835 1.63 0.05 1.309 0.50 0.07 0.607 0.27 0.13

RS2061929 1.63 0.05 1.309 0.50 0.07 0.607 0.27 0.13

RS9868325 1.64 0.05 1.309 0.50 0.07 0.608 0.27 0.13

RS6439928 1.64 0.05 1.309 0.50 0.07 0.608 0.27 0.13

RS524554 1.64 0.05 1.309 0.50 0.07 0.608 0.27 0.13

RS9876562 1.64 0.05 1.309 0.50 0.06 0.608 0.27 0.13

RS9826671 1.64 0.05 1.309 0.50 0.06 0.608 0.27 0.13

RS7620622 1.64 0.05 1.309 0.50 0.06 0.608 0.27 0.13

RS2048456 1.64 0.05 1.309 0.50 0.06 0.608 0.27 0.13

RS9880195 1.64 0.05 1.309 0.50 0.06 0.608 0.27 0.13

RS4683600 1.64 0.05 1.309 0.50 0.06 0.608 0.27 0.13

RS4683618 1.64 0.05 1.309 0.50 0.06 0.608 0.27 0.13

RS17195428 1.64 0.05 1.309 0.50 0.06 0.608 0.27 0.13

RS9882031 1.64 0.05 1.309 0.50 0.06 0.608 0.27 0.13

RS10433346 1.64 0.05 1.309 0.50 0.06 0.608 0.27 0.13

RS6806847 1.64 0.05 1.309 0.50 0.06 0.608 0.27 0.13

RS6764361 1.64 0.05 1.309 0.50 0.06 0.608 0.27 0.13

RS13061823 1.64 0.05 1.309 0.50 0.06 0.608 0.27 0.13

RS11715610 1.64 0.05 1.309 0.50 0.06 0.608 0.27 0.13

RS7621642 1.64 0.05 1.309 0.50 0.06 0.608 0.27 0.13

RS7647899 1.64 0.05 1.309 0.50 0.06 0.608 0.27 0.13

RS2581618 1.64 0.05 1.309 0.50 0.06 0.608 0.27 0.13

RS9832727 1.64 0.05 1.309 0.50 0.06 0.608 0.27 0.13

RS6664 1.64 0.05 1.309 0.50 0.06 0.608 0.27 0.13

RS16853045 1.64 0.05 1.309 0.50 0.06 0.608 0.27 0.13

RS4839620 1.64 0.05 1.309 0.50 0.06 0.608 0.27 0.13

RS9839394 1.64 0.05 1.309 0.50 0.06 0.608 0.27 0.13

RS7625382 1.64 0.05 1.309 0.50 0.06 0.608 0.27 0.13

RS9857832 1.64 0.05 1.309 0.50 0.06 0.608 0.27 0.13

RS11707857 1.64 0.05 1.309 0.50 0.06 0.608 0.27 0.13

RS1554672 1.64 0.05 1.309 0.50 0.06 0.608 0.27 0.13

RS9871107 1.64 0.05 1.309 0.50 0.06 0.608 0.27 0.13

RS11919836 1.64 0.05 1.309 0.50 0.06 0.608 0.27 0.13

RS13085041 1.64 0.05 1.309 0.50 0.06 0.608 0.27 0.13

RS13089318 1.64 0.05 1.309 0.50 0.06 0.608 0.27 0.13

RS1447738 1.64 0.05 1.309 0.50 0.06 0.608 0.27 0.13

RS7631319 1.64 0.05 1.309 0.50 0.06 0.608 0.27 0.13

RS7649414 1.64 0.05 1.309 0.50 0.06 0.608 0.27 0.13

RS11714850 1.64 0.05 1.309 0.50 0.06 0.608 0.27 0.13

RS12491888 1.64 0.05 1.309 0.50 0.06 0.608 0.27 0.13

RS12233446 1.64 0.05 1.309 0.50 0.06 0.608 0.27 0.13

RS6769847 1.64 0.05 1.309 0.50 0.06 0.608 0.27 0.13

RS6803662 1.64 0.05 1.309 0.50 0.06 0.608 0.27 0.13

RS7631704 1.64 0.05 1.309 0.50 0.06 0.608 0.27 0.13

RS4431107 1.64 0.05 1.309 0.50 0.06 0.608 0.27 0.13

RS9841242 1.64 0.05 1.309 0.50 0.06 0.608 0.27 0.13

RS6440251 1.64 0.05 1.309 0.50 0.06 0.608 0.27 0.13

RS13068539 1.64 0.05 1.309 0.50 0.06 0.608 0.27 0.13

RS9852425 1.64 0.05 1.309 0.50 0.06 0.608 0.27 0.13

RS1092103 1.64 0.05 1.309 0.50 0.06 0.608 0.27 0.13

RS1949727 1.64 0.05 1.309 0.50 0.06 0.608 0.27 0.13

RS10935515 1.64 0.05 1.309 0.50 0.06 0.608 0.27 0.13

RS4611857 1.64 0.05 1.309 0.50 0.06 0.608 0.27 0.13

RS3924267 1.64 0.05 1.309 0.50 0.06 0.608 0.27 0.13

RS7632966 1.64 0.05 1.309 0.50 0.06 0.608 0.27 0.13

RS7646194 1.64 0.05 1.309 0.50 0.06 0.608 0.27 0.13

RS345017 1.64 0.05 1.309 0.50 0.06 0.608 0.27 0.13

RS1881036 1.64 0.05 1.309 0.50 0.06 0.608 0.27 0.13

RS2459136 1.64 0.05 1.309 0.50 0.06 0.608 0.27 0.13

RS3863994 1.64 0.05 1.309 0.50 0.06 0.608 0.27 0.13

RS7614629 1.64 0.05 1.309 0.50 0.06 0.608 0.27 0.13

RS1398775 1.64 0.05 1.309 0.50 0.06 0.608 0.27 0.13

RS1553977 1.64 0.05 1.309 0.50 0.06 0.608 0.27 0.13

RS1449444 1.64 0.05 1.309 0.50 0.06 0.608 0.27 0.13

RS12632001 1.64 0.05 1.309 0.50 0.06 0.608 0.27 0.13

RS13068178 1.64 0.05 1.309 0.50 0.06 0.608 0.27 0.13

RS343292 1.64 0.05 1.309 0.50 0.06 0.608 0.27 0.13

RS9842222 1.64 0.05 1.309 0.50 0.06 0.608 0.27 0.13

RS11924592 1.64 0.05 1.309 0.50 0.06 0.608 0.27 0.13

RS9871995 1.64 0.05 1.309 0.50 0.06 0.608 0.27 0.13

RS9289731 1.64 0.05 1.309 0.50 0.06 0.608 0.27 0.13

RS13091393 1.64 0.05 1.309 0.50 0.06 0.608 0.27 0.13

RS11721056 1.64 0.05 1.309 0.50 0.06 0.608 0.27 0.13

RS9865951 1.64 0.05 1.309 0.50 0.06 0.608 0.27 0.13

RS1814982 1.64 0.05 1.309 0.50 0.06 0.608 0.27 0.13

RS1354054 1.64 0.05 1.309 0.50 0.06 0.608 0.27 0.13

RS9289748 1.64 0.05 1.309 0.50 0.06 0.608 0.27 0.13

RS9855367 1.64 0.05 1.309 0.50 0.06 0.608 0.27 0.13

RS4681371 1.64 0.05 1.309 0.50 0.06 0.608 0.27 0.13

RS4681375 1.64 0.05 1.309 0.50 0.06 0.608 0.27 0.13

RS7627412 1.64 0.05 1.309 0.50 0.06 0.608 0.27 0.13

RS4681379 1.64 0.05 1.309 0.50 0.06 0.608 0.27 0.13

RS9814408 1.64 0.05 1.309 0.50 0.06 0.608 0.27 0.13

RS13058910 1.64 0.05 1.309 0.50 0.06 0.608 0.27 0.13

RS9825391 1.64 0.05 1.309 0.50 0.06 0.608 0.27 0.13

RS4681158 1.64 0.05 1.309 0.50 0.06 0.608 0.27 0.13

RS3772604 1.64 0.05 1.309 0.50 0.06 0.608 0.27 0.13

RS3772603 1.64 0.05 1.309 0.50 0.06 0.608 0.27 0.13

RS1440640 1.64 0.05 1.309 0.50 0.06 0.608 0.27 0.13

RS7623948 1.64 0.05 1.309 0.50 0.06 0.608 0.27 0.13

RS4470441 1.64 0.05 1.309 0.50 0.06 0.608 0.27 0.13

RS7624541 1.64 0.05 1.309 0.50 0.06 0.608 0.27 0.13

RS6766575 1.64 0.05 1.309 0.50 0.06 0.608 0.27 0.13

RS1344817 1.64 0.05 1.309 0.50 0.06 0.608 0.27 0.13

RS6779455 1.64 0.05 1.309 0.50 0.06 0.608 0.27 0.13

RS1564996 1.64 0.05 1.309 0.50 0.06 0.608 0.27 0.13

RS1386287 1.64 0.05 1.309 0.50 0.06 0.608 0.27 0.13

RS9790045 1.64 0.05 1.309 0.50 0.06 0.608 0.27 0.13

RS7623697 1.64 0.05 1.309 0.50 0.06 0.608 0.27 0.13

RS7652403 1.64 0.05 1.309 0.50 0.06 0.608 0.27 0.13

RS13087661 1.64 0.05 1.309 0.50 0.06 0.608 0.27 0.13

RS12639090 1.64 0.05 1.309 0.50 0.06 0.608 0.27 0.13

RS7634891 1.64 0.05 1.309 0.50 0.06 0.608 0.27 0.13

RS9849937 1.64 0.05 1.309 0.50 0.06 0.608 0.27 0.13

RS13074670 1.64 0.05 1.309 0.50 0.06 0.608 0.27 0.13

RS7615125 1.64 0.05 1.309 0.50 0.06 0.608 0.27 0.13

RS10935819 1.64 0.05 1.309 0.50 0.06 0.608 0.27 0.13

RS3732753 1.64 0.05 1.309 0.50 0.06 0.608 0.27 0.13

RS1456139 1.64 0.05 1.309 0.50 0.06 0.608 0.27 0.13

RS1014691 1.64 0.05 1.309 0.50 0.06 0.608 0.27 0.13

RS6766361 1.64 0.05 1.309 0.50 0.06 0.608 0.27 0.13

RS1703802 1.64 0.05 1.309 0.50 0.06 0.608 0.27 0.13

RS358966 1.64 0.05 1.309 0.50 0.06 0.608 0.27 0.13

RS7644001 1.64 0.05 1.309 0.50 0.06 0.608 0.27 0.13

RS765281 1.64 0.05 1.309 0.50 0.06 0.608 0.27 0.13

RS9842591 1.64 0.05 1.309 0.50 0.06 0.608 0.27 0.13

RS1402740 1.64 0.05 1.309 0.50 0.06 0.608 0.27 0.13

RS13080679 1.64 0.05 1.309 0.50 0.06 0.608 0.27 0.13

RS7644103 1.64 0.05 1.309 0.50 0.06 0.608 0.27 0.13

RS13081526 1.64 0.05 1.309 0.50 0.06 0.608 0.27 0.13

RS2271942 1.64 0.05 1.309 0.50 0.06 0.608 0.27 0.13

RS13315275 1.64 0.05 1.309 0.50 0.06 0.608 0.27 0.13

RS2046034 1.64 0.05 1.309 0.50 0.06 0.608 0.27 0.13

RS323614 1.52 0.06 1.309 0.48 0.07 0.584 0.24 0.15

RS10513419 1.50 0.07 1.309 0.47 0.07 0.581 0.24 0.15

RS17372441 1.34 0.09 1.309 0.44 0.08 0.542 0.19 0.2

RS12233485 1.34 0.09 1.309 0.44 0.08 0.542 0.19 0.2

RS6773552 1.34 0.09 1.309 0.44 0.08 0.542 0.19 0.2

RS11718068 1.34 0.09 1.309 0.44 0.08 0.542 0.19 0.2

RS9872160 3.61 0.0002 1.309 0.76 0.03 3.124 1.44 0.005

RS9883654 4.70 0.00000 1.309 0.85 0.02 3.125 1.62 0.003

RS13070843 4.70 0.00000 1.309 0.85 0.02 3.125 1.62 0.003

RS528581 4.70 0.00000 1.309 0.85 0.02 3.125 1.62 0.003

RS1899695 4.70 0.00000 1.309 0.85 0.02 3.125 1.62 0.003

RS9839852 4.70 0.00000 1.309 0.85 0.02 3.125 1.62 0.003

RS6782397 4.70 0.00000 1.309 0.85 0.02 3.125 1.62 0.003

RS1388008 4.70 0.00000 1.309 0.85 0.02 3.125 1.62 0.003

RS9823065 4.70 0.00000 1.309 0.85 0.02 3.125 1.62 0.003

RS9857982 4.70 0.00000 1.309 0.85 0.02 3.125 1.62 0.003

RS17803063 4.70 0.00000 1.309 0.85 0.02 3.125 1.62 0.003

RS3112291 4.70 0.00000 1.309 0.85 0.02 3.125 1.62 0.003

RS355748 4.70 0.00000 1.309 0.85 0.02 3.126 1.62 0.003

RS2136837 4.70 0.00000 1.309 0.85 0.02 3.126 1.62 0.003

RS7643334 4.70 0.00000 1.309 0.85 0.02 3.125 1.62 0.003

RS10513470 4.70 0.00000 1.309 0.85 0.02 3.125 1.62 0.003

RS2016846 4.70 0.00000 1.309 0.85 0.02 3.125 1.62 0.003

RS3773876 4.70 0.00000 1.309 0.85 0.02 3.125 1.62 0.003

RS7624578 4.70 0.00000 1.309 0.85 0.02 3.125 1.62 0.003

RS1385481 4.70 0.00000 1.309 0.85 0.02 3.125 1.62 0.003

RS2200392 4.70 0.00000 1.309 0.85 0.02 3.125 1.62 0.003

RS6783837 4.70 0.00000 1.309 0.85 0.02 3.125 1.62 0.003

RS7619671 4.70 0.00000 1.309 0.85 0.02 3.125 1.62 0.003

RS4680230 4.70 0.00000 1.309 0.85 0.02 3.125 1.62 0.003

RS1474024 4.70 0.00000 1.309 0.85 0.02 3.125 1.62 0.003

RS4359774 4.70 0.00000 1.309 0.85 0.02 3.125 1.62 0.003

RS6777084 1.51 0.06 1.309 0.47 0.07 3.094 0.34 0.10

RS2293198 1.34 0.09 1.309 0.44 0.08 0.542 0.20 0.2

RS16826325 1.34 0.09 1.309 0.44 0.08 0.542 0.20 0.2

RS9867857 1.34 0.09 1.309 0.44 0.08 0.542 0.19 0.2

RS35649103 1.34 0.09 1.309 0.44 0.08 0.542 0.19 0.2

RS3845978 1.34 0.09 1.309 0.44 0.08 0.542 0.19 0.2

RS9877620 1.34 0.09 1.309 0.44 0.08 0.542 0.19 0.2

RS9289990 1.34 0.09 1.309 0.44 0.08 0.542 0.19 0.2

RS16828412 1.34 0.09 1.309 0.44 0.08 0.542 0.19 0.2

RS7622880 1.34 0.09 1.309 0.44 0.08 0.542 0.19 0.2

RS10936111 1.34 0.09 1.309 0.44 0.08 0.542 0.19 0.2

RS4679819 1.34 0.09 1.309 0.44 0.08 0.542 0.19 0.2

RS1346834 1.34 0.09 1.309 0.44 0.08 0.542 0.19 0.2

RS17645548 1.34 0.09 1.309 0.44 0.08 0.542 0.19 0.2

RS340270 1.34 0.09 1.309 0.44 0.08 0.542 0.19 0.2

RS9811068 1.34 0.09 1.309 0.44 0.08 0.542 0.19 0.2

RS1112924 1.34 0.09 1.309 0.44 0.08 0.542 0.19 0.2

RS4501157 1.34 0.09 1.309 0.44 0.08 0.542 0.19 0.2

RS6781001 1.34 0.09 1.309 0.44 0.08 0.542 0.19 0.2

RS1449004 1.34 0.09 1.309 0.44 0.08 0.542 0.19 0.2

RS1489919 1.34 0.09 1.309 0.44 0.08 0.542 0.19 0.2

RS9877650 1.34 0.09 1.309 0.44 0.08 0.542 0.19 0.2

RS2243143 1.34 0.09 1.309 0.44 0.08 0.542 0.19 0.2

RS574808 1.34 0.09 1.309 0.44 0.08 0.542 0.19 0.2

RS868916 1.34 0.09 1.309 0.44 0.08 0.542 0.19 0.2

RS1047210 1.34 0.09 1.309 0.44 0.08 0.542 0.19 0.2

RS7651855 1.34 0.09 1.309 0.44 0.08 0.542 0.19 0.2

RS456611 1.34 0.09 1.309 0.44 0.08 0.542 0.20 0.2

RS6780945 1.34 0.09 1.309 0.44 0.08 0.542 0.19 0.2

RS6779918 1.34 0.09 1.309 0.44 0.08 0.542 0.19 0.2

RS6771785 1.34 0.09 1.309 0.44 0.08 0.542 0.19 0.2

RS12496424 1.34 0.09 1.309 0.44 0.08 0.542 0.19 0.2

RS7611892 1.34 0.09 1.309 0.44 0.08 0.542 0.19 0.2

RS35780213 1.34 0.09 1.309 0.44 0.08 0.542 0.19 0.2

RS903943 1.34 0.09 1.309 0.44 0.08 0.542 0.19 0.2

RS3853175 1.34 0.09 1.309 0.44 0.08 0.542 0.19 0.2

RS9836765 1.34 0.09 1.309 0.44 0.08 0.542 0.19 0.2

RS2046696 1.34 0.09 1.309 0.44 0.08 0.542 0.20 0.2

RS13060071 1.34 0.09 1.309 0.44 0.08 0.542 0.20 0.2

RS6810129 1.34 0.09 1.309 0.44 0.08 0.542 0.20 0.2

RS1581740 1.34 0.09 1.309 0.44 0.08 0.542 0.20 0.2

RS10936400 1.34 0.09 1.309 0.44 0.08 0.542 0.20 0.2

RS892765 1.34 0.09 1.309 0.44 0.08 0.542 0.19 0.2

RS892761 1.34 0.09 1.309 0.44 0.08 0.542 0.19 0.2

RS12633984 1.34 0.09 1.309 0.44 0.08 0.542 0.19 0.2

RS1877596 1.34 0.09 1.309 0.44 0.08 0.542 0.19 0.2

RS2722633 1.34 0.09 1.309 0.44 0.08 0.542 0.19 0.2

RS9812705 1.34 0.09 1.309 0.44 0.08 0.542 0.19 0.2

RS9878522 1.34 0.09 1.309 0.44 0.08 0.542 0.19 0.2

RS6803772 1.34 0.09 1.309 0.44 0.08 0.542 0.19 0.2

RS1403617 1.34 0.09 1.309 0.44 0.08 0.542 0.19 0.2

RS11923311 1.34 0.09 1.309 0.44 0.08 0.542 0.19 0.2

RS2084525 1.34 0.09 1.309 0.44 0.08 0.542 0.19 0.2

RS6797530 1.34 0.09 1.309 0.44 0.08 0.542 0.19 0.2

RS10936474 1.34 0.09 1.309 0.44 0.08 0.542 0.19 0.2

RS9820644 1.34 0.09 1.309 0.44 0.08 0.542 0.19 0.2

RS13062023 1.34 0.09 1.309 0.44 0.08 0.542 0.19 0.2

RS16850977 1.34 0.09 1.309 0.44 0.08 0.542 0.19 0.2

RS1607650 1.34 0.09 1.309 0.44 0.08 0.542 0.19 0.2

RS9878131 1.34 0.09 1.309 0.44 0.08 0.542 0.19 0.2

RS9846882 1.34 0.09 1.309 0.44 0.08 0.542 0.19 0.2

RS11918193 1.34 0.09 1.309 0.44 0.08 0.542 0.19 0.2

RS6774632 1.34 0.09 1.309 0.44 0.08 0.542 0.19 0.2

RS9858935 1.34 0.09 1.309 0.44 0.08 0.542 0.19 0.2

RS11914302 1.34 0.09 1.309 0.44 0.08 0.542 0.19 0.2

RS9824277 1.34 0.09 1.309 0.44 0.08 0.542 0.19 0.2

RS1025217 1.34 0.09 1.309 0.44 0.08 0.542 0.19 0.2

RS884828 1.34 0.09 1.309 0.44 0.08 0.542 0.19 0.2

RS8179961 1.34 0.09 1.309 0.44 0.08 0.542 0.19 0.2

RS3914117 1.34 0.09 1.309 0.44 0.08 0.542 0.19 0.2

RS16852581 1.34 0.09 1.309 0.44 0.08 0.542 0.19 0.2

RS496741 1.34 0.09 1.309 0.44 0.08 0.542 0.19 0.2

RS1498444 1.34 0.09 1.309 0.44 0.08 0.542 0.19 0.2

RS13089423 1.28 0.10 1.309 0.43 0.08 0.533 0.18 0.2

RS12629466 1.20 0.11 1.309 0.41 0.08 0.519 0.17 0.2

RS16853318 1.19 0.12 1.309 0.41 0.09 0.516 0.17 0.2

RS9850919 1.07 0.14 1.309 0.38 0.09 0.490 0.14 0.2

RS7652987 1.06 0.14 1.309 0.38 0.09 0.489 0.14 0.2

RS1881305 1.05 0.15 1.309 0.38 0.09 0.487 0.14 0.2

RS933605 0.98 0.2 1.309 0.36 0.10 0.469 0.12 0.2

RS16854236 0.98 0.2 1.309 0.36 0.10 0.468 0.12 0.2

RS12486767 0.87 0.2 1.309 0.33 0.11 0.438 0.10 0.2

RS2901585 0.80 0.2 1.309 0.31 0.12 0.414 0.09 0.3

RS1082976 0.73 0.2 1.309 0.29 0.12 0.389 0.07 0.3

RS13096001 0.68 0.2 1.309 0.27 0.13 0.365 0.06 0.3

RS10936636 0.68 0.2 1.309 0.27 0.13 0.365 0.06 0.3

RS1861939 0.68 0.2 1.309 0.27 0.13 0.365 0.06 0.3

RS1468977 0.68 0.2 1.309 0.27 0.13 0.365 0.06 0.3

RS1011295 0.68 0.2 1.309 0.27 0.13 0.365 0.06 0.3

RS12488260 0.87 0.2 1.309 0.33 0.11 0.438 0.10 0.2

RS1356012 0.93 0.2 1.309 0.35 0.10 0.455 0.11 0.2

RS6791187 1.34 0.09 1.309 0.44 0.08 0.542 0.19 0.2

RS902952 1.34 0.09 1.309 0.44 0.08 0.542 0.19 0.2

RS6807555 1.34 0.09 1.309 0.44 0.08 0.542 0.19 0.2

RS12107455 1.34 0.09 1.309 0.44 0.08 0.542 0.19 0.2

RS3850186 1.34 0.09 1.309 0.44 0.08 0.542 0.19 0.2

RS9826915 1.34 0.09 1.309 0.44 0.08 0.542 0.20 0.2

RS559235 1.34 0.09 1.309 0.44 0.08 0.542 0.20 0.2

RS7620170 1.34 0.09 1.309 0.44 0.08 0.542 0.20 0.2

RS4894413 1.34 0.09 1.309 0.44 0.08 0.542 0.19 0.2

RS8180024 1.34 0.09 1.309 0.44 0.08 0.542 0.19 0.2

RS6773689 1.34 0.09 1.309 0.44 0.08 0.542 0.19 0.2

RS7642511 1.34 0.09 1.309 0.44 0.08 0.542 0.19 0.2

RS9809402 1.34 0.09 1.309 0.44 0.08 0.542 0.19 0.2

RS231997 1.34 0.09 1.309 0.44 0.08 0.542 0.19 0.2

RS231989 1.34 0.09 1.309 0.44 0.08 0.542 0.19 0.2

RS234025 1.34 0.09 1.309 0.44 0.08 0.542 0.19 0.2

RS2901811 1.34 0.09 1.309 0.44 0.08 0.542 0.19 0.2

RS10513704 1.34 0.09 1.309 0.44 0.08 0.542 0.19 0.2

RS4894576 1.34 0.09 1.309 0.44 0.08 0.542 0.19 0.2

RS6445091 1.34 0.09 1.309 0.44 0.08 0.542 0.20 0.2

RS12630529 1.34 0.09 1.309 0.44 0.08 0.542 0.20 0.2

RS10936748 1.34 0.09 1.309 0.44 0.08 0.542 0.20 0.2

RS12489095 1.34 0.09 1.309 0.44 0.08 0.542 0.20 0.2

RS9840933 1.34 0.09 1.309 0.44 0.08 0.542 0.20 0.2

RS1402216 1.34 0.09 1.309 0.44 0.08 0.542 0.20 0.2

RS6771377 1.34 0.09 1.309 0.44 0.08 0.542 0.19 0.2

RS9868927 1.34 0.09 1.309 0.44 0.08 0.542 0.19 0.2

RS9290476 1.34 0.09 1.309 0.44 0.08 0.542 0.19 0.2

RS3980098 1.34 0.09 1.309 0.44 0.08 0.542 0.19 0.2

RS9290488 1.34 0.09 1.309 0.44 0.08 0.542 0.19 0.2

RS1345537 1.34 0.09 1.309 0.44 0.08 0.542 0.19 0.2

RS2193744 1.34 0.09 1.309 0.44 0.08 0.542 0.19 0.2

RS9812312 1.34 0.09 1.309 0.44 0.08 0.542 0.20 0.2

RS1515592 1.34 0.09 1.309 0.44 0.08 0.542 0.20 0.2

RS6793059 1.34 0.09 1.309 0.44 0.08 0.542 0.20 0.2

RS13072735 1.34 0.09 1.309 0.44 0.08 0.542 0.20 0.2

RS10936812 1.34 0.09 1.309 0.44 0.08 0.542 0.20 0.2

RS6774160 1.34 0.09 1.309 0.44 0.08 0.542 0.20 0.2

RS7609643 1.34 0.09 1.309 0.44 0.08 0.542 0.20 0.2

RS4425273 1.34 0.09 1.309 0.44 0.08 0.542 0.19 0.2

RS9823658 1.34 0.09 1.309 0.44 0.08 0.542 0.19 0.2

RS2861999 1.34 0.09 1.309 0.44 0.08 0.542 0.19 0.2

RS1463525 1.34 0.09 1.309 0.44 0.08 0.542 0.19 0.2

RS7619752 1.34 0.09 1.309 0.44 0.08 0.542 0.19 0.2

RS6443300 1.34 0.09 1.309 0.44 0.08 0.542 0.19 0.2

RS9879950 1.34 0.09 1.309 0.44 0.08 0.542 0.19 0.2

RS6799976 1.34 0.09 1.309 0.44 0.08 0.542 0.19 0.2

RS7625843 1.34 0.09 1.309 0.44 0.08 0.542 0.19 0.2

RS4894485 1.34 0.09 1.309 0.44 0.08 0.542 0.19 0.2

RS9290550 1.34 0.09 1.309 0.44 0.08 0.542 0.19 0.2

RS6789345 1.34 0.09 1.309 0.44 0.08 0.542 0.19 0.2

RS4894489 1.34 0.09 1.309 0.44 0.08 0.542 0.19 0.2

RS6784051 1.34 0.09 1.309 0.44 0.08 0.542 0.19 0.2

RS2184207 1.34 0.09 1.309 0.44 0.08 0.542 0.19 0.2

RS9869315 1.26 0.10 1.309 0.42 0.08 0.529 0.18 0.2

RS4577477 0.72 0.2 1.309 0.29 0.12 0.380 0.07 0.3

RS1921476 0.72 0.2 1.309 0.29 0.12 0.380 0.07 0.3

RS6799767 0.72 0.2 1.309 0.29 0.12 0.380 0.07 0.3

RS12489250 0.72 0.2 1.309 0.29 0.12 0.380 0.07 0.3

RS7632772 0.72 0.2 1.309 0.29 0.12 0.380 0.07 0.3

RS6778967 0.72 0.2 1.309 0.29 0.12 0.380 0.07 0.3

RS7651271 0.72 0.2 1.309 0.29 0.12 0.380 0.07 0.3

RS7647703 0.72 0.2 1.309 0.29 0.12 0.380 0.07 0.3

RS6772982 0.72 0.2 1.309 0.29 0.12 0.380 0.07 0.3

RS13097413 0.72 0.2 1.309 0.29 0.12 0.380 0.07 0.3

RS6443423 0.72 0.2 1.309 0.29 0.12 0.380 0.07 0.3

RS12490455 0.72 0.2 1.309 0.29 0.12 0.380 0.07 0.3

RS6443443 0.72 0.2 1.309 0.29 0.12 0.380 0.07 0.3

RS9873211 0.72 0.2 1.309 0.29 0.12 0.380 0.07 0.3

RS6804202 0.72 0.2 1.309 0.29 0.12 0.380 0.07 0.3

RS9830263 0.42 0.3 1.309 0.19 0.2 0.286 0.03 0.4

RS9881791 0.37 0.4 1.309 0.17 0.2 0.266 0.02 0.4

RS4857702 0.30 0.4 1.309 0.14 0.2 0.232 0.02 0.4

RS6804861 -0.09 0.5 -0.198 -0.01 0.6 -0.110 -0.00 0.5

RS4857737 -0.09 0.5 -0.198 -0.01 0.6 -0.110 -0.00 0.5

RS6788525 -0.09 0.5 -0.198 -0.01 0.6 -0.110 -0.00 0.5

RS12490085 -0.09 0.5 -0.198 -0.01 0.6 -0.110 -0.00 0.5

RS1565327 -0.09 0.5 -0.198 -0.01 0.6 -0.110 -0.00 0.5

RS282875 -0.09 0.5 -0.198 -0.01 0.6 -0.110 -0.00 0.5

RS6769947 -0.09 0.5 -0.198 -0.01 0.6 -0.110 -0.00 0.5

RS1477773 -0.09 0.5 -0.198 -0.01 0.6 -0.110 -0.00 0.5

RS6798240 -0.09 0.5 -0.198 -0.01 0.6 -0.110 -0.00 0.5

RS7433956 -0.09 0.5 -0.198 -0.01 0.6 -0.110 -0.00 0.5

RS9839376 -0.09 0.5 -0.198 -0.01 0.6 -0.110 -0.00 0.5

RS4370006 -0.09 0.5 -0.198 -0.01 0.6 -0.110 -0.00 0.5

RS4624543 -0.09 0.5 -0.198 -0.01 0.6 -0.110 -0.00 0.5

RS4401404 -0.09 0.5 -0.198 -0.01 0.6 -0.110 -0.00 0.5

RS7610174 -0.09 0.5 -0.198 -0.01 0.6 -0.110 -0.00 0.5

RS2287312 -0.09 0.5 -0.198 -0.01 0.6 -0.110 -0.00 0.5

RS9822116 -0.09 0.5 -0.198 -0.01 0.6 -0.110 -0.00 0.5

RS7428403 -0.09 0.5 -0.198 -0.01 0.6 -0.110 -0.00 0.5

RS4855081 -0.09 0.5 -0.198 -0.01 0.6 -0.110 -0.00 0.5

RS6775913 -0.09 0.5 -0.198 -0.01 0.6 -0.110 -0.00 0.5

RS16830685 -0.09 0.5 -0.198 -0.01 0.6 -0.110 -0.00 0.5

RS4854948 -0.09 0.5 -0.198 -0.01 0.6 -0.110 -0.00 0.5

RS7643653 -0.09 0.5 -0.198 -0.01 0.6 -0.110 -0.00 0.5

RS2056596 -0.09 0.5 -0.198 -0.01 0.6 -0.110 -0.00 0.5

RS7636571 -0.09 0.5 -0.198 -0.01 0.6 -0.110 -0.00 0.5

RS4855129 -0.09 0.5 -0.198 -0.01 0.6 -0.110 -0.00 0.5

RS12488363 -0.09 0.5 -0.198 -0.01 0.6 -0.110 -0.00 0.5

RS956395 -0.09 0.5 -0.198 -0.01 0.6 -0.110 -0.00 0.5

RS2339932 -0.09 0.5 -0.198 -0.01 0.6 -0.110 -0.00 0.5

RS16831406 -0.09 0.5 -0.198 -0.01 0.6 -0.110 -0.00 0.5

RS9817579 -0.09 0.5 -0.198 -0.01 0.6 -0.110 -0.00 0.5

RS12496529 -0.09 0.5 -0.198 -0.01 0.6 -0.110 -0.00 0.5

RS7628371 -0.09 0.5 -0.198 -0.01 0.6 -0.110 -0.00 0.5

RS9810292 -0.09 0.5 -0.198 -0.01 0.6 -0.110 -0.00 0.5

RS6778227 -0.09 0.5 -0.198 -0.01 0.6 -0.110 -0.00 0.5

RS11927844 -0.09 0.5 -0.198 -0.01 0.6 -0.110 -0.00 0.5

RS4855037 -0.09 0.5 -0.198 -0.01 0.6 -0.110 -0.00 0.5

RS12489879 -0.09 0.5 -0.198 -0.01 0.6 -0.110 -0.00 0.5

RS833282 -0.09 0.5 -0.198 -0.01 0.6 -0.110 -0.00 0.5

RS13096015 -0.09 0.5 -0.198 -0.01 0.6 -0.110 -0.00 0.5

RS4855069 -0.09 0.5 -0.198 -0.01 0.6 -0.110 -0.00 0.5

RS4854932 -0.09 0.5 -0.198 -0.01 0.6 -0.110 -0.00 0.5

RS13074509 -0.09 0.5 -0.198 -0.01 0.6 -0.110 -0.00 0.5

RS2339722 -0.09 0.5 -0.198 -0.01 0.6 -0.110 -0.00 0.5

RS9283647 -0.09 0.5 -0.198 -0.01 0.6 -0.110 -0.00 0.5

RS2315505 -0.09 0.5 -0.198 -0.01 0.6 -0.110 -0.00 0.5

RS7355912 -0.09 0.5 -0.198 -0.01 0.6 -0.110 -0.00 0.5

RS2717297 -0.09 0.5 -0.198 -0.01 0.6 -0.110 -0.00 0.5

RS940072 -0.09 0.5 -0.198 -0.01 0.6 -0.110 -0.00 0.5

RS6780927 -0.09 0.5 -0.198 -0.01 0.6 -0.110 -0.00 0.5

RS13098857 -0.09 0.5 -0.198 -0.01 0.6 -0.110 -0.00 0.5

RS3772715 -0.09 0.5 -0.198 -0.01 0.6 -0.110 -0.00 0.5

RS1496802 -0.09 0.5 -0.198 -0.01 0.6 -0.110 -0.00 0.5

RS6809726 -0.09 0.5 -0.198 -0.01 0.6 -0.110 -0.00 0.5

RS4859211 -0.09 0.5 -0.198 -0.01 0.6 -0.110 -0.00 0.5

RS6799350 -0.09 0.5 -0.198 -0.01 0.6 -0.110 -0.00 0.5

RS3805114 -0.09 0.5 -0.198 -0.01 0.6 -0.110 -0.00 0.5

RS4148594 -0.09 0.5 -0.198 -0.01 0.6 -0.110 -0.00 0.5

RS10937159 -0.09 0.5 -0.198 -0.01 0.6 -0.110 -0.00 0.5

RS6807670 -0.09 0.5 -0.198 -0.01 0.6 -0.110 -0.00 0.5

RS7627615 -0.09 0.5 -0.198 -0.01 0.6 -0.110 -0.00 0.5

RS9860004 -0.09 0.5 -0.198 -0.01 0.6 -0.110 -0.00 0.5

RS4558788 -0.09 0.5 -0.198 -0.01 0.6 -0.110 -0.00 0.5

RS4912567 -0.09 0.5 -0.198 -0.01 0.6 -0.110 -0.00 0.5

RS4912574 -0.09 0.5 -0.198 -0.01 0.6 -0.110 -0.00 0.5

RS11709414 -0.09 0.5 -0.198 -0.01 0.6 -0.110 -0.00 0.5

RS7652597 -0.09 0.5 -0.198 -0.01 0.6 -0.110 -0.00 0.5

RS10937179 -0.09 0.5 -0.198 -0.01 0.6 -0.110 -0.00 0.5

RS9846698 -0.09 0.5 -0.198 -0.01 0.6 -0.110 -0.00 0.5

RS7631034 -0.09 0.5 -0.198 -0.01 0.6 -0.110 -0.00 0.5

RS9844571 -0.09 0.5 -0.198 -0.01 0.6 -0.110 -0.00 0.5

RS9818534 -0.09 0.5 -0.198 -0.01 0.6 -0.110 -0.00 0.5

RS6444007 -0.09 0.5 -0.198 -0.01 0.6 -0.110 -0.00 0.5

RS9878804 -0.09 0.5 -0.198 -0.01 0.6 -0.110 -0.00 0.5

RS11718277 -0.09 0.5 -0.198 -0.01 0.6 -0.110 -0.00 0.5

RS1065096 -0.09 0.5 -0.198 -0.01 0.6 -0.110 -0.00 0.5

RS17217776 -0.09 0.5 -0.198 -0.01 0.6 -0.110 -0.00 0.5

RS10937226 -0.09 0.5 -0.198 -0.01 0.6 -0.110 -0.00 0.5

RS17826758 -0.09 0.5 -0.198 -0.01 0.6 -0.110 -0.00 0.5

RS13076013 -0.09 0.5 -0.198 -0.01 0.6 -0.110 -0.00 0.5

RS12631770 -0.09 0.5 -0.198 -0.01 0.6 -0.110 -0.00 0.5

RS7614436 -0.09 0.5 -0.198 -0.01 0.6 -0.110 -0.00 0.5

RS9867846 -0.09 0.5 -0.198 -0.01 0.6 -0.110 -0.00 0.5

RS7627157 -0.09 0.5 -0.198 -0.01 0.6 -0.110 -0.00 0.5

RS2284842 -0.09 0.5 -0.198 -0.01 0.6 -0.110 -0.00 0.5

RS3819863 -0.09 0.5 -0.198 -0.01 0.6 -0.110 -0.00 0.5

RS3935586 -0.09 0.5 -0.198 -0.01 0.6 -0.110 -0.00 0.5

RS9681406 -0.09 0.5 -0.198 -0.01 0.6 -0.110 -0.00 0.5

RS9844915 -0.09 0.5 -0.198 -0.01 0.6 -0.110 -0.00 0.5

RS13089682 -0.09 0.5 -0.198 -0.01 0.6 -0.110 -0.00 0.5

RS1530641 -0.09 0.5 -0.198 -0.01 0.6 -0.110 -0.00 0.5

RS1131364 -0.09 0.5 -0.198 -0.01 0.6 -0.110 -0.00 0.5

RS5030062 -0.09 0.5 -0.198 -0.01 0.6 -0.110 -0.00 0.5

RS266745 -0.09 0.5 -0.198 -0.01 0.6 -0.110 -0.00 0.5

RS182052 -0.09 0.5 -0.198 -0.01 0.6 -0.110 -0.00 0.5

RS16861329 -0.09 0.5 -0.198 -0.01 0.6 -0.110 -0.00 0.5

RS6444191 -0.09 0.5 -0.198 -0.01 0.6 -0.110 -0.00 0.5

RS6808800 -0.09 0.5 -0.198 -0.01 0.6 -0.110 -0.00 0.5

RS257101 -0.09 0.5 -0.198 -0.01 0.6 -0.110 -0.00 0.5

RS270161 -0.09 0.5 -0.198 -0.01 0.6 -0.110 -0.00 0.5

RS270144 -0.09 0.5 -0.198 -0.01 0.6 -0.110 -0.00 0.5

RS3864098 -0.09 0.5 -0.198 -0.01 0.6 -0.110 -0.00 0.5

RS698108 -0.09 0.5 -0.198 -0.01 0.6 -0.110 -0.00 0.5

RS1003995 -0.09 0.5 -0.198 -0.01 0.6 -0.110 -0.00 0.5

RS9853501 -0.09 0.5 -0.198 -0.01 0.6 -0.110 -0.00 0.5

RS2378265 -0.09 0.5 -0.198 -0.01 0.6 -0.110 -0.00 0.5

RS1559772 -0.09 0.5 -0.198 -0.01 0.6 -0.110 -0.00 0.5

RS512178 -0.09 0.5 -0.198 -0.01 0.6 -0.110 -0.00 0.5

RS763342 -0.09 0.5 -0.198 -0.01 0.6 -0.110 -0.00 0.5

RS2030033 -0.09 0.5 -0.198 -0.01 0.6 -0.110 -0.00 0.5

RS10937330 -0.09 0.5 -0.198 -0.01 0.6 -0.110 -0.00 0.5

RS16862908 -0.09 0.5 -0.198 -0.01 0.6 -0.110 -0.00 0.5

RS2221070 -0.09 0.5 -0.198 -0.01 0.6 -0.110 -0.00 0.5

RS9852144 -0.09 0.5 -0.198 -0.01 0.6 -0.110 -0.00 0.5

RS1711001 -0.09 0.5 -0.198 -0.01 0.6 -0.110 -0.00 0.5

RS9989991 -0.09 0.5 -0.198 -0.01 0.6 -0.110 -0.00 0.5

RS6809682 -0.09 0.5 -0.198 -0.01 0.6 -0.110 -0.00 0.5

RS9868086 -0.09 0.5 -0.198 -0.01 0.6 -0.110 -0.00 0.5

RS1559810 -0.09 0.5 -0.198 -0.01 0.6 -0.110 -0.00 0.5

RS9851525 -0.09 0.5 -0.198 -0.01 0.6 -0.110 -0.00 0.5

RS4686969 -0.09 0.5 -0.198 -0.01 0.6 -0.110 -0.00 0.5

RS6444300 -0.09 0.5 -0.198 -0.01 0.6 -0.110 -0.00 0.5

RS9837401 -0.09 0.5 -0.198 -0.01 0.6 -0.110 -0.00 0.5

RS9877898 -0.09 0.5 -0.198 -0.01 0.6 -0.110 -0.00 0.5

RS4686492 -0.09 0.5 -0.198 -0.01 0.6 -0.110 -0.00 0.5

RS1355720 -0.09 0.5 -0.198 -0.01 0.6 -0.110 -0.00 0.5

RS4686998 -0.09 0.5 -0.198 -0.01 0.6 -0.110 -0.00 0.5

RS9834801 -0.09 0.5 -0.198 -0.01 0.6 -0.110 -0.00 0.5

RS6783147 -0.09 0.5 -0.198 -0.01 0.6 -0.110 -0.00 0.5

RS9875124 -0.09 0.5 -0.198 -0.01 0.6 -0.110 -0.00 0.5

RS16864324 -0.09 0.5 -0.198 -0.01 0.6 -0.110 -0.00 0.5

RS9850625 -0.09 0.5 -0.198 -0.01 0.6 -0.110 -0.00 0.5

RS6787246 -0.09 0.5 -0.198 -0.01 0.6 -0.110 -0.00 0.5

RS6790258 -0.09 0.5 -0.198 -0.01 0.6 -0.110 -0.00 0.5

RS7636839 -0.09 0.5 -0.198 -0.01 0.6 -0.110 -0.00 0.5

RS7649341 -0.15 0.6 -0.198 -0.01 0.6 -0.201 -0.01 0.6

RS2889918 -0.17 0.6 -0.198 -0.01 0.6 -0.243 -0.01 0.6

RS11720810 -0.35 0.6 -0.198 -0.03 0.6 -1.008 -0.05 0.7

RS2037128 -0.37 0.6 -0.198 -0.03 0.6 -1.138 -0.06 0.7

RS17448036 -0.42 0.7 -0.198 -0.03 0.7 -1.488 -0.08 0.7

RS7642848 -0.50 0.7 -0.198 -0.04 0.7 -2.059 -0.13 0.8

RS568904 -0.50 0.7 -0.198 -0.04 0.7 -2.059 -0.13 0.8

RS3098262 -0.50 0.7 -0.198 -0.04 0.7 -2.059 -0.13 0.8

RS1436659 -0.50 0.7 -0.198 -0.04 0.7 -2.059 -0.13 0.8

RS9852867 -0.50 0.7 -0.198 -0.04 0.7 -2.059 -0.13 0.8

RS3774021 -0.50 0.7 -0.198 -0.04 0.7 -2.059 -0.13 0.8

RS3846102 -0.50 0.7 -0.198 -0.04 0.7 -2.059 -0.13 0.8

RS1946326 -0.50 0.7 -0.198 -0.04 0.7 -2.059 -0.13 0.8

RS6797395 -0.50 0.7 -0.198 -0.04 0.7 -2.059 -0.13 0.8

RS3773997 -0.50 0.7 -0.198 -0.04 0.7 -2.059 -0.13 0.8

RS4686554 -0.50 0.7 -0.198 -0.04 0.7 -2.059 -0.13 0.8

RS3773982 -0.51 0.7 -0.198 -0.04 0.7 -2.173 -0.14 0.8

RS10937439 -0.53 0.7 -0.198 -0.04 0.7 -2.437 -0.15 0.8

RS2193873 -0.53 0.7 -0.198 -0.04 0.7 -2.437 -0.15 0.8

RS9873487 -0.53 0.7 -0.198 -0.04 0.7 -2.437 -0.15 0.8

RS9830737 -0.53 0.7 -0.198 -0.04 0.7 -2.437 -0.15 0.8

RS16865974 -0.53 0.7 -0.198 -0.04 0.7 -2.437 -0.15 0.8

RS1501600 -0.53 0.7 -0.198 -0.04 0.7 -2.437 -0.15 0.8

RS9880180 -0.53 0.7 -0.198 -0.04 0.7 -2.437 -0.15 0.8

RS7645672 -0.53 0.7 -0.198 -0.04 0.7 -2.437 -0.15 0.8

RS7613308 -0.53 0.7 -0.198 -0.04 0.7 -2.437 -0.15 0.8

RS2063421 -0.53 0.7 -0.198 -0.04 0.7 -2.437 -0.15 0.8

RS9814786 -0.53 0.7 -0.198 -0.04 0.7 -2.437 -0.15 0.8

RS12152251 -0.53 0.7 -0.198 -0.04 0.7 -2.437 -0.15 0.8

RS7637404 -0.53 0.7 -0.198 -0.04 0.7 -2.437 -0.15 0.8

RS1473866 -0.53 0.7 -0.198 -0.04 0.7 -2.437 -0.15 0.8

RS2028572 -0.53 0.7 -0.198 -0.04 0.7 -2.437 -0.15 0.8

RS7618303 -0.53 0.7 -0.198 -0.04 0.7 -2.437 -0.15 0.8

RS406821 -0.53 0.7 -0.198 -0.04 0.7 -2.437 -0.15 0.8

RS11922996 -0.53 0.7 -0.198 -0.04 0.7 -2.437 -0.15 0.8

RS4074485 -0.53 0.7 -0.198 -0.04 0.7 -2.437 -0.15 0.8

RS7629648 -0.53 0.7 -0.198 -0.04 0.7 -2.437 -0.15 0.8

RS4686582 -0.53 0.7 -0.198 -0.04 0.7 -2.437 -0.15 0.8

RS1083696 -0.53 0.7 -0.198 -0.04 0.7 -2.437 -0.15 0.8

RS10937527 -0.53 0.7 -0.198 -0.04 0.7 -2.437 -0.15 0.8

RS12497223 -0.53 0.7 -0.198 -0.04 0.7 -2.437 -0.15 0.8

RS9869920 -0.53 0.7 -0.198 -0.04 0.7 -2.437 -0.15 0.8

RS2366659 -0.53 0.7 -0.198 -0.04 0.7 -2.437 -0.15 0.8

RS1525910 -0.53 0.7 -0.198 -0.04 0.7 -2.437 -0.15 0.8

RS12488767 -0.53 0.7 -0.198 -0.04 0.7 -2.437 -0.15 0.8

RS12107377 -0.53 0.7 -0.198 -0.04 0.7 -2.437 -0.15 0.8

RS1532684 -0.53 0.7 -0.198 -0.04 0.7 -2.437 -0.15 0.8

RS2654694 -0.53 0.7 -0.198 -0.04 0.7 -2.437 -0.15 0.8

RS9813078 -0.53 0.7 -0.198 -0.04 0.7 -2.437 -0.15 0.8

RS2986 -0.53 0.7 -0.198 -0.04 0.7 -2.437 -0.15 0.8

RS9831317 -0.53 0.7 -0.198 -0.04 0.7 -2.437 -0.15 0.8

RS9813423 -0.53 0.7 -0.198 -0.04 0.7 -2.437 -0.15 0.8

RS9852198 -0.53 0.7 -0.198 -0.04 0.7 -2.437 -0.15 0.8

RS10428211 -0.53 0.7 -0.198 -0.04 0.7 -2.437 -0.15 0.8

RS9824804 -0.53 0.7 -0.198 -0.04 0.7 -2.437 -0.15 0.8

RS1980263 -0.53 0.7 -0.198 -0.04 0.7 -2.437 -0.15 0.8

RS6764121 -0.53 0.7 -0.198 -0.04 0.7 -2.436 -0.15 0.8

RS6803383 -0.52 0.7 -0.198 -0.04 0.7 -2.436 -0.15 0.8

RS587612 -0.52 0.7 -0.198 -0.04 0.7 -2.436 -0.15 0.8

RS13324043 -0.53 0.7 -0.198 -0.04 0.7 -2.437 -0.15 0.8

RS1078814 -0.53 0.7 -0.198 -0.04 0.7 -2.437 -0.15 0.8

RS9851309 -0.53 0.7 -0.198 -0.04 0.7 -2.437 -0.15 0.8

RS7628226 -0.53 0.7 -0.198 -0.04 0.7 -2.437 -0.15 0.8

RS9848849 -0.53 0.7 -0.198 -0.04 0.7 -2.437 -0.15 0.8

RS6796696 -0.53 0.7 -0.198 -0.04 0.7 -2.437 -0.15 0.8

RS6766839 -0.53 0.7 -0.198 -0.04 0.7 -2.437 -0.15 0.8

RS7613652 -0.53 0.7 -0.198 -0.04 0.7 -2.437 -0.15 0.8

RS10933727 -0.53 0.7 -0.198 -0.04 0.7 -2.437 -0.15 0.8

RS11922293 -0.51 0.7 -0.198 -0.04 0.7 -2.265 -0.14 0.8

RS10933657 -0.37 0.6 -0.198 -0.03 0.6 -0.960 -0.05 0.7

RS6437384 -0.36 0.6 -0.198 -0.03 0.6 -0.912 -0.05 0.7

RS1675951 -0.27 0.6 -0.198 -0.02 0.6 -0.497 -0.02 0.6

RS4677854 -0.27 0.6 -0.198 -0.02 0.6 -0.497 -0.02 0.6

RS13084613 -0.27 0.6 -0.198 -0.02 0.6 -0.496 -0.02 0.6

RS6764953 -0.27 0.6 -0.198 -0.02 0.6 -0.496 -0.02 0.6

RS4677766 -0.27 0.6 -0.198 -0.02 0.6 -0.496 -0.02 0.6

RS6764929 -0.27 0.6 -0.198 -0.02 0.6 -0.496 -0.02 0.6

RS7641855 -0.27 0.6 -0.198 -0.02 0.6 -0.496 -0.02 0.6

RS2676854 -0.27 0.6 -0.198 -0.02 0.6 -0.496 -0.02 0.6

RS2676894 -0.27 0.6 -0.198 -0.02 0.6 -0.496 -0.02 0.6

RS1105819 -0.27 0.6 -0.198 -0.02 0.6 -0.496 -0.02 0.6

RS823302 -0.27 0.6 -0.198 -0.02 0.6 -0.496 -0.02 0.6

RS2259661 -0.38 0.6 -0.198 -0.03 0.6 -1.196 -0.06 0.7

RS1908173 -0.39 0.7 -0.198 -0.03 0.7 -1.341 -0.07 0.7

RS4677695 -0.42 0.7 -0.198 -0.03 0.7 -1.771 -0.09 0.7

RS2550295 -0.54 0.7 -0.198 -0.04 0.7 -5.065 -0.22 0.8

RS9858622 -0.63 0.7 -0.198 -0.05 0.7 -6.274 -0.34 0.9

RS9859401 -0.63 0.7 -0.198 -0.05 0.7 -6.274 -0.34 0.9

RS4927868 -0.63 0.7 -0.198 -0.05 0.7 -6.274 -0.34 0.9

RS12490036 -0.63 0.7 -0.198 -0.05 0.7 -6.274 -0.34 0.9

RS3850643 -0.63 0.7 -0.198 -0.05 0.7 -6.274 -0.34 0.9

RS7639752 -0.63 0.7 -0.198 -0.05 0.7 -6.274 -0.34 0.9

RS6799210 -0.63 0.7 -0.198 -0.05 0.7 -6.274 -0.34 0.9

RS2089979 -0.63 0.7 -0.198 -0.05 0.7 -6.274 -0.34 0.9

RS12497912 -0.63 0.7 -0.198 -0.05 0.7 -6.274 -0.34 0.9

RS555582 -0.63 0.7 -0.198 -0.05 0.7 -6.274 -0.34 0.9

RS498738 -0.63 0.7 -0.198 -0.05 0.7 -6.274 -0.34 0.9

RS6764023 -0.63 0.7 -0.198 -0.05 0.7 -6.274 -0.34 0.9

RS6583202 -0.63 0.7 -0.198 -0.05 0.7 -6.274 -0.34 0.9

RS4916471 -0.63 0.7 -0.198 -0.05 0.7 -6.274 -0.34 0.9

RS7644219 -0.63 0.7 -0.198 -0.05 0.7 -6.274 -0.34 0.9

RS13099300 -0.63 0.7 -0.198 -0.05 0.7 -6.274 -0.34 0.9

RS2567344 -0.63 0.7 -0.198 -0.05 0.7 -6.274 -0.34 0.9

RS2116294 -0.63 0.7 -0.198 -0.05 0.7 -6.274 -0.34 0.9

RS6781945 -0.63 0.7 -0.198 -0.05 0.7 -6.274 -0.34 0.9

RS9841534 -0.63 0.7 -0.198 -0.05 0.7 -6.273 -0.34 0.9

RS1606519 -0.63 0.7 -0.198 -0.05 0.7 -6.299 -0.34 0.9

RS10428227 -0.63 0.7 -0.198 -0.05 0.7 -6.313 -0.34 0.9

RS9809736 -0.63 0.7 -0.198 -0.05 0.7 -6.344 -0.33 0.9

Analysing Chromosome 4

Phenotype: AFFSTAT [ALL] (1 family)

===============================================================================

Pos Zmean pvalue linDelta LOD pvalue expDelta LOD pvalue

min -0.76 0.8 -0.198 -0.06 0.7 -9.999 -0.55 0.9

max 5.05 0.00000 1.309 0.88 0.02 9.999 2.62 0.0003

RS7699689 -0.43 0.7 -0.198 -0.04 0.7 -1.287 -0.08 0.7

RS6817633 -0.43 0.7 -0.198 -0.04 0.7 -1.291 -0.08 0.7

RS11944122 -0.43 0.7 -0.198 -0.04 0.7 -1.298 -0.08 0.7

RS6838241 -0.43 0.7 -0.198 -0.04 0.7 -1.298 -0.08 0.7

RS11722977 -0.43 0.7 -0.198 -0.04 0.7 -1.298 -0.08 0.7

RS1564280 -0.43 0.7 -0.198 -0.04 0.7 -1.298 -0.08 0.7

RS7687129 -0.43 0.7 -0.198 -0.04 0.7 -1.298 -0.08 0.7

RS11724804 -0.43 0.7 -0.198 -0.04 0.7 -1.298 -0.08 0.7

RS11734750 -0.43 0.7 -0.198 -0.04 0.7 -1.298 -0.08 0.7

RS11247975 -0.43 0.7 -0.198 -0.04 0.7 -1.298 -0.08 0.7

RS1250116 -0.43 0.7 -0.198 -0.04 0.7 -1.298 -0.08 0.7

RS1564508 -0.43 0.7 -0.198 -0.04 0.7 -1.298 -0.08 0.7

RS13117476 -0.43 0.7 -0.198 -0.04 0.7 -1.298 -0.08 0.7

RS4865424 -0.43 0.7 -0.198 -0.04 0.7 -1.298 -0.08 0.7

RS3936194 -0.43 0.7 -0.198 -0.04 0.7 -1.298 -0.08 0.7

RS7667125 -0.43 0.7 -0.198 -0.04 0.7 -1.298 -0.08 0.7

RS11248073 -0.43 0.7 -0.198 -0.04 0.7 -1.298 -0.08 0.7

RS7668207 -0.43 0.7 -0.198 -0.04 0.7 -1.298 -0.08 0.7

RS580602 -0.43 0.7 -0.198 -0.04 0.7 -1.298 -0.08 0.7

RS2097298 -0.43 0.7 -0.198 -0.04 0.7 -1.298 -0.08 0.7

RS4257708 -0.43 0.7 -0.198 -0.04 0.7 -1.298 -0.08 0.7

RS9990857 -0.43 0.7 -0.198 -0.04 0.7 -1.298 -0.08 0.7

RS17825119 -0.43 0.7 -0.198 -0.04 0.7 -1.298 -0.08 0.7

RS1751848 -0.43 0.7 -0.198 -0.04 0.7 -1.298 -0.08 0.7

RS16843589 -0.43 0.7 -0.198 -0.04 0.7 -1.298 -0.08 0.7

RS362331 -0.43 0.7 -0.198 -0.04 0.7 -1.298 -0.08 0.7

RS3095081 -0.43 0.7 -0.198 -0.04 0.7 -1.298 -0.08 0.7

RS13116176 -0.43 0.7 -0.198 -0.04 0.7 -1.298 -0.08 0.7

RS16844383 -0.43 0.7 -0.198 -0.04 0.7 -1.298 -0.08 0.7

RS1203111 -0.43 0.7 -0.198 -0.04 0.7 -1.298 -0.08 0.7

RS1730775 -0.43 0.7 -0.198 -0.04 0.7 -1.298 -0.08 0.7

RS6856283 -0.43 0.7 -0.198 -0.04 0.7 -1.298 -0.08 0.7

RS7673350 -0.43 0.7 -0.198 -0.04 0.7 -1.298 -0.08 0.7

RS177798 -0.43 0.7 -0.198 -0.04 0.7 -1.298 -0.08 0.7

RS445275 -0.43 0.7 -0.198 -0.04 0.7 -1.298 -0.08 0.7

RS6822427 -0.43 0.7 -0.198 -0.04 0.7 -1.298 -0.08 0.7

RS28366830 -0.43 0.7 -0.198 -0.04 0.7 -1.298 -0.08 0.7

RS1991915 -0.43 0.7 -0.198 -0.04 0.7 -1.298 -0.08 0.7

RS4688956 -0.43 0.7 -0.198 -0.04 0.7 -1.298 -0.08 0.7

RS6446665 -0.43 0.7 -0.198 -0.04 0.7 -1.298 -0.08 0.7

RS4552410 -0.43 0.7 -0.198 -0.04 0.7 -1.298 -0.08 0.7

RS11732485 -0.43 0.7 -0.198 -0.04 0.7 -1.298 -0.08 0.7

RS12648481 -0.43 0.7 -0.198 -0.04 0.7 -1.298 -0.08 0.7

RS6854227 -0.43 0.7 -0.198 -0.04 0.7 -1.298 -0.08 0.7

RS13117653 -0.43 0.7 -0.198 -0.04 0.7 -1.298 -0.08 0.7

RS4689946 -0.43 0.7 -0.198 -0.04 0.7 -1.298 -0.08 0.7

RS4435686 -0.43 0.7 -0.198 -0.04 0.7 -1.298 -0.08 0.7

RS11733243 -0.43 0.7 -0.198 -0.04 0.7 -1.298 -0.08 0.7

RS4689967 -0.43 0.7 -0.198 -0.04 0.7 -1.298 -0.08 0.7

RS11933917 -0.43 0.7 -0.198 -0.04 0.7 -1.298 -0.08 0.7

RS6820110 -0.43 0.7 -0.198 -0.04 0.7 -1.298 -0.08 0.7

RS1868047 -0.43 0.7 -0.198 -0.04 0.7 -1.298 -0.08 0.7

RS6825132 -0.43 0.7 -0.198 -0.04 0.7 -1.298 -0.08 0.7

RS12651470 -0.43 0.7 -0.198 -0.04 0.7 -1.298 -0.08 0.7

RS10011769 -0.43 0.7 -0.198 -0.04 0.7 -1.298 -0.08 0.7

RS1562425 -0.43 0.7 -0.198 -0.04 0.7 -1.298 -0.08 0.7

RS12504675 -0.43 0.7 -0.198 -0.04 0.7 -1.298 -0.08 0.7

RS7670299 -0.43 0.7 -0.198 -0.04 0.7 -1.299 -0.08 0.7

RS10804964 -0.43 0.7 -0.198 -0.04 0.7 -1.299 -0.08 0.7

RS7681283 -0.43 0.7 -0.198 -0.04 0.7 -1.299 -0.08 0.7

RS2286280 -0.44 0.7 -0.198 -0.04 0.7 -1.366 -0.08 0.7

RS4456913 -0.44 0.7 -0.198 -0.04 0.7 -1.399 -0.09 0.7

RS17694785 -0.44 0.7 -0.198 -0.04 0.7 -1.424 -0.09 0.7

RS9684740 -0.44 0.7 -0.198 -0.04 0.7 -1.424 -0.09 0.7

RS4689333 -0.44 0.7 -0.198 -0.04 0.7 -1.424 -0.09 0.7

RS4689355 -0.44 0.7 -0.198 -0.04 0.7 -1.424 -0.09 0.7

RS10002743 -0.44 0.7 -0.198 -0.04 0.7 -1.424 -0.09 0.7

RS3796403 -0.44 0.7 -0.198 -0.04 0.7 -1.424 -0.09 0.7

RS13124487 -0.44 0.7 -0.198 -0.04 0.7 -1.424 -0.09 0.7

RS6446495 -0.44 0.7 -0.198 -0.04 0.7 -1.424 -0.09 0.7

RS6851340 -0.44 0.7 -0.198 -0.04 0.7 -1.424 -0.09 0.7

RS2240268 -0.44 0.7 -0.198 -0.04 0.7 -1.424 -0.09 0.7

RS7434469 -0.44 0.7 -0.198 -0.04 0.7 -1.424 -0.09 0.7

RS9291117 -0.44 0.7 -0.198 -0.04 0.7 -1.424 -0.09 0.7

RS4404624 -0.44 0.7 -0.198 -0.04 0.7 -1.424 -0.09 0.7

RS7654321 -0.44 0.7 -0.198 -0.04 0.7 -1.424 -0.09 0.7

RS11731327 -0.44 0.7 -0.198 -0.04 0.7 -1.424 -0.09 0.7

RS6847759 -0.44 0.7 -0.198 -0.04 0.7 -1.424 -0.09 0.7

RS4689498 -0.44 0.7 -0.198 -0.04 0.7 -1.424 -0.09 0.7

RS3172604 -0.44 0.7 -0.198 -0.04 0.7 -1.424 -0.09 0.7

RS4393997 -0.44 0.7 -0.198 -0.04 0.7 -1.424 -0.09 0.7

RS10001636 -0.44 0.7 -0.198 -0.04 0.7 -1.424 -0.09 0.7

RS11732898 -0.44 0.7 -0.198 -0.04 0.7 -1.424 -0.09 0.7

RS3857176 -0.44 0.7 -0.198 -0.04 0.7 -1.424 -0.09 0.7

RS6446566 -0.44 0.7 -0.198 -0.04 0.7 -1.424 -0.09 0.7

RS10155062 -0.41 0.7 -0.198 -0.03 0.7 -1.181 -0.07 0.7

RS7687509 -0.40 0.7 -0.198 -0.03 0.7 -1.138 -0.07 0.7

RS6836687 -0.39 0.7 -0.198 -0.03 0.7 -1.097 -0.06 0.7

RS9999241 -0.38 0.6 -0.198 -0.03 0.6 -0.992 -0.06 0.7

RS951449 -0.36 0.6 -0.198 -0.03 0.6 -0.865 -0.05 0.7

RS4234797 -0.35 0.6 -0.198 -0.03 0.6 -0.816 -0.04 0.7

RS4689707 -0.27 0.6 -0.198 -0.02 0.6 -0.497 -0.02 0.6

RS10005640 -0.27 0.6 -0.198 -0.02 0.6 -0.497 -0.02 0.6

RS4558873 -0.28 0.6 -0.198 -0.02 0.6 -0.497 -0.02 0.6

RS7682286 -0.28 0.6 -0.198 -0.02 0.6 -0.497 -0.02 0.6

RS10033032 -0.28 0.6 -0.198 -0.02 0.6 -0.497 -0.02 0.6

RS4380522 -0.28 0.6 -0.198 -0.02 0.6 -0.497 -0.02 0.6

RS9647418 -0.27 0.6 -0.198 -0.02 0.6 -0.497 -0.02 0.6

RS6812745 -0.27 0.6 -0.198 -0.02 0.6 -0.497 -0.02 0.6

RS10012347 -0.27 0.6 -0.198 -0.02 0.6 -0.497 -0.02 0.6

RS16840670 -0.27 0.6 -0.198 -0.02 0.6 -0.495 -0.02 0.6

RS4689838 -0.27 0.6 -0.198 -0.02 0.6 -0.492 -0.02 0.6

RS12233824 -0.27 0.6 -0.198 -0.02 0.6 -0.492 -0.02 0.6

RS4689866 -0.27 0.6 -0.198 -0.02 0.6 -0.492 -0.02 0.6

RS4234831 -0.27 0.6 -0.198 -0.02 0.6 -0.492 -0.02 0.6

RS17770177 -0.27 0.6 -0.198 -0.02 0.6 -0.496 -0.02 0.6

RS4478172 -0.27 0.6 -0.198 -0.02 0.6 -0.496 -0.02 0.6

RS4696886 -0.32 0.6 -0.198 -0.03 0.6 -0.668 -0.03 0.7

RS13129405 -0.33 0.6 -0.198 -0.03 0.6 -0.747 -0.04 0.7

RS6830813 -0.36 0.6 -0.198 -0.03 0.6 -0.913 -0.05 0.7

RS1320058 -0.37 0.6 -0.198 -0.03 0.6 -0.958 -0.05 0.7

RS922521 -0.44 0.7 -0.198 -0.04 0.7 -1.424 -0.09 0.7

RS1281152 -0.44 0.7 -0.198 -0.04 0.7 -1.424 -0.09 0.7

RS2631735 -0.44 0.7 -0.198 -0.04 0.7 -1.424 -0.09 0.7

RS6447879 -0.44 0.7 -0.198 -0.04 0.7 -1.424 -0.09 0.7

RS4696842 -0.44 0.7 -0.198 -0.04 0.7 -1.424 -0.09 0.7

RS11735274 -0.44 0.7 -0.198 -0.04 0.7 -1.424 -0.09 0.7

RS3115395 -0.44 0.7 -0.198 -0.04 0.7 -1.424 -0.09 0.7

RS1282 -0.44 0.7 -0.198 -0.04 0.7 -1.424 -0.09 0.7

RS10938726 -0.44 0.7 -0.198 -0.04 0.7 -1.424 -0.09 0.7

RS3822202 -0.44 0.7 -0.198 -0.04 0.7 -1.424 -0.09 0.7

RS10938742 -0.44 0.7 -0.198 -0.04 0.7 -1.425 -0.09 0.7

RS6858299 -0.44 0.7 -0.198 -0.04 0.7 -1.425 -0.09 0.7

RS3733591 -0.44 0.7 -0.198 -0.04 0.7 -1.424 -0.09 0.7

RS4604059 -0.44 0.7 -0.198 -0.04 0.7 -1.424 -0.09 0.7

RS993173 -0.44 0.7 -0.198 -0.04 0.7 -1.424 -0.09 0.7

RS4333176 -0.44 0.7 -0.198 -0.04 0.7 -1.424 -0.09 0.7

RS1974584 -0.44 0.7 -0.198 -0.04 0.7 -1.424 -0.09 0.7

RS4697777 -0.44 0.7 -0.198 -0.04 0.7 -1.424 -0.09 0.7

RS4074743 -0.44 0.7 -0.198 -0.04 0.7 -1.424 -0.09 0.7

RS17280854 -0.44 0.7 -0.198 -0.04 0.7 -1.424 -0.09 0.7

RS10222862 -0.44 0.7 -0.198 -0.04 0.7 -1.424 -0.09 0.7

RS6846529 -0.44 0.7 -0.198 -0.04 0.7 -1.424 -0.09 0.7

RS10034712 -0.44 0.7 -0.198 -0.04 0.7 -1.424 -0.09 0.7

RS4697681 -0.44 0.7 -0.198 -0.04 0.7 -1.424 -0.09 0.7

RS224481 -0.44 0.7 -0.198 -0.04 0.7 -1.424 -0.09 0.7

RS7699705 -0.44 0.7 -0.198 -0.04 0.7 -1.424 -0.09 0.7

RS1603186 -0.22 0.6 -0.198 -0.02 0.6 -0.334 -0.01 0.6

RS10155331 -0.21 0.6 -0.198 -0.02 0.6 -0.328 -0.01 0.6

RS1032502 -0.21 0.6 -0.198 -0.02 0.6 -0.328 -0.01 0.6

RS10488991 -0.21 0.6 -0.198 -0.02 0.6 -0.328 -0.01 0.6

RS1388867 -0.21 0.6 -0.198 -0.02 0.6 -0.328 -0.01 0.6

RS7661409 -0.21 0.6 -0.198 -0.02 0.6 -0.328 -0.01 0.6

RS6843251 -0.21 0.6 -0.198 -0.02 0.6 -0.328 -0.01 0.6

RS2171726 -0.21 0.6 -0.198 -0.02 0.6 -0.328 -0.01 0.6

RS11725886 -0.21 0.6 -0.198 -0.02 0.6 -0.328 -0.01 0.6

RS1479273 -0.21 0.6 -0.198 -0.02 0.6 -0.328 -0.01 0.6

RS7677522 -0.21 0.6 -0.198 -0.02 0.6 -0.328 -0.01 0.6

RS1378233 -0.21 0.6 -0.198 -0.02 0.6 -0.328 -0.01 0.6

RS7663013 -0.21 0.6 -0.198 -0.02 0.6 -0.328 -0.01 0.6

RS17366799 -0.21 0.6 -0.198 -0.02 0.6 -0.328 -0.01 0.6

RS6857474 -0.21 0.6 -0.198 -0.02 0.6 -0.328 -0.01 0.6

RS2540821 -0.21 0.6 -0.198 -0.02 0.6 -0.328 -0.01 0.6

RS7675207 -0.21 0.6 -0.198 -0.02 0.6 -0.328 -0.01 0.6

RS6850802 -0.21 0.6 -0.198 -0.02 0.6 -0.328 -0.01 0.6

RS16888112 -0.21 0.6 -0.198 -0.02 0.6 -0.328 -0.01 0.6

RS496099 -0.21 0.6 -0.198 -0.02 0.6 -0.328 -0.01 0.6

RS503506 -0.21 0.6 -0.198 -0.02 0.6 -0.328 -0.01 0.6

RS562920 -0.21 0.6 -0.198 -0.02 0.6 -0.328 -0.01 0.6

RS6851825 -0.21 0.6 -0.198 -0.02 0.6 -0.328 -0.01 0.6

RS2109590 -0.21 0.6 -0.198 -0.02 0.6 -0.328 -0.01 0.6

RS12651430 -0.21 0.6 -0.198 -0.02 0.6 -0.328 -0.01 0.6

RS10025528 -0.21 0.6 -0.198 -0.02 0.6 -0.328 -0.01 0.6

RS759261 -0.21 0.6 -0.198 -0.02 0.6 -0.328 -0.01 0.6

RS7697386 -0.21 0.6 -0.198 -0.02 0.6 -0.328 -0.01 0.6

RS887924 -0.21 0.6 -0.198 -0.02 0.6 -0.328 -0.01 0.6

RS4698178 -0.21 0.6 -0.198 -0.02 0.6 -0.328 -0.01 0.6

RS1861135 -0.21 0.6 -0.198 -0.02 0.6 -0.328 -0.01 0.6

RS9917971 -0.21 0.6 -0.198 -0.02 0.6 -0.328 -0.01 0.6

RS3096587 -0.21 0.6 -0.198 -0.02 0.6 -0.328 -0.01 0.6

RS10017183 -0.21 0.6 -0.198 -0.02 0.6 -0.328 -0.01 0.6

RS1541757 -0.21 0.6 -0.198 -0.02 0.6 -0.328 -0.01 0.6

RS16889955 -0.21 0.6 -0.198 -0.02 0.6 -0.328 -0.01 0.6

RS6824715 -0.21 0.6 -0.198 -0.02 0.6 -0.328 -0.01 0.6

RS1501170 -0.21 0.6 -0.198 -0.02 0.6 -0.328 -0.01 0.6

RS4327484 -0.21 0.6 -0.198 -0.02 0.6 -0.328 -0.01 0.6

RS11731529 -0.27 0.6 -0.198 -0.02 0.6 -0.495 -0.02 0.6

RS9884305 -0.31 0.6 -0.198 -0.03 0.6 -0.649 -0.03 0.7

RS12498517 -0.33 0.6 -0.198 -0.03 0.6 -0.740 -0.04 0.7

RS7687891 -0.41 0.7 -0.198 -0.03 0.7 -1.247 -0.07 0.7

RS10014445 -0.46 0.7 -0.198 -0.04 0.7 -1.536 -0.10 0.7

RS6811795 -0.46 0.7 -0.198 -0.04 0.7 -1.536 -0.10 0.7

RS6449134 -0.46 0.7 -0.198 -0.04 0.7 -1.536 -0.10 0.7

RS3733593 -0.46 0.7 -0.198 -0.04 0.7 -1.535 -0.10 0.7

RS2532070 -0.46 0.7 -0.198 -0.04 0.7 -1.535 -0.10 0.7

RS4698424 -0.46 0.7 -0.198 -0.04 0.7 -1.535 -0.10 0.7

RS876320 -0.32 0.6 -0.198 -0.03 0.6 -0.664 -0.04 0.7

RS12503130 -0.32 0.6 -0.198 -0.03 0.6 -0.664 -0.04 0.7

RS6835120 -0.32 0.6 -0.198 -0.03 0.6 -0.664 -0.04 0.7

RS10428501 -0.32 0.6 -0.198 -0.03 0.6 -0.664 -0.04 0.7

RS2078622 -0.32 0.6 -0.198 -0.03 0.6 -0.664 -0.04 0.7

RS3857152 -0.32 0.6 -0.198 -0.03 0.6 -0.664 -0.04 0.7

RS884227 -0.32 0.6 -0.198 -0.03 0.6 -0.664 -0.04 0.7

RS1115917 -0.32 0.6 -0.198 -0.03 0.6 -0.664 -0.04 0.7

RS1712493 -0.32 0.6 -0.198 -0.03 0.6 -0.664 -0.04 0.7

RS2041538 -0.32 0.6 -0.198 -0.03 0.6 -0.664 -0.04 0.7

RS13131868 -0.32 0.6 -0.198 -0.03 0.6 -0.664 -0.04 0.7

RS2313547 -0.32 0.6 -0.198 -0.03 0.6 -0.664 -0.04 0.7

RS4698149 -0.32 0.6 -0.198 -0.03 0.6 -0.664 -0.04 0.7

RS6816070 -0.32 0.6 -0.198 -0.03 0.6 -0.664 -0.04 0.7

RS10805351 -0.32 0.6 -0.198 -0.03 0.6 -0.664 -0.04 0.7

RS6818414 -0.32 0.6 -0.198 -0.03 0.6 -0.664 -0.04 0.7

RS1972463 -0.32 0.6 -0.198 -0.03 0.6 -0.664 -0.04 0.7

RS1027784 -0.32 0.6 -0.198 -0.03 0.6 -0.664 -0.04 0.7

RS157619 -0.32 0.6 -0.198 -0.03 0.6 -0.664 -0.04 0.7

RS16893890 -0.32 0.6 -0.198 -0.03 0.6 -0.664 -0.04 0.7

RS207713 -0.32 0.6 -0.198 -0.03 0.6 -0.664 -0.04 0.7

RS13126845 -0.32 0.6 -0.198 -0.03 0.6 -0.664 -0.04 0.7

RS2314597 -0.32 0.6 -0.198 -0.03 0.6 -0.664 -0.04 0.7

RS1356028 -0.32 0.6 -0.198 -0.03 0.6 -0.664 -0.04 0.7

RS627206 -0.32 0.6 -0.198 -0.03 0.6 -0.664 -0.04 0.7

RS6449278 -0.32 0.6 -0.198 -0.03 0.6 -0.664 -0.04 0.7

RS501015 -0.32 0.6 -0.198 -0.03 0.6 -0.664 -0.04 0.7

RS12507442 -0.32 0.6 -0.198 -0.03 0.6 -0.664 -0.04 0.7

RS4698560 -0.32 0.6 -0.198 -0.03 0.6 -0.664 -0.04 0.7

RS13130097 -0.32 0.6 -0.198 -0.03 0.6 -0.664 -0.04 0.7

RS2122574 -0.32 0.6 -0.198 -0.03 0.6 -0.664 -0.04 0.7

RS2697684 -0.32 0.6 -0.198 -0.03 0.6 -0.664 -0.04 0.7

RS2518641 -0.32 0.6 -0.198 -0.03 0.6 -0.664 -0.04 0.7

RS5005545 -0.32 0.6 -0.198 -0.03 0.6 -0.664 -0.04 0.7

RS10604 -0.32 0.6 -0.198 -0.03 0.6 -0.664 -0.04 0.7

RS4698603 -0.32 0.6 -0.198 -0.03 0.6 -0.664 -0.04 0.7

RS2109520 -0.32 0.6 -0.198 -0.03 0.6 -0.664 -0.04 0.7

RS9884404 -0.32 0.6 -0.198 -0.03 0.6 -0.664 -0.04 0.7

RS6825100 -0.32 0.6 -0.198 -0.03 0.6 -0.664 -0.04 0.7

RS1506613 -0.32 0.6 -0.198 -0.03 0.6 -0.664 -0.04 0.7

RS6856758 -0.32 0.6 -0.198 -0.03 0.6 -0.664 -0.04 0.7

RS1503884 -0.32 0.6 -0.198 -0.03 0.6 -0.664 -0.04 0.7

RS1395099 -0.47 0.7 -0.198 -0.04 0.7 -4.008 -0.14 0.8

RS1568234 -0.67 0.7 -0.198 -0.05 0.7 -9.999 -0.49 0.9

RS9291679 -0.68 0.8 -0.198 -0.05 0.7 -9.999 -0.49 0.9

RS1356472 -0.68 0.8 -0.198 -0.05 0.7 -9.999 -0.49 0.9

RS16897709 -0.68 0.8 -0.198 -0.05 0.7 -9.999 -0.49 0.9

RS2215762 -0.68 0.8 -0.198 -0.05 0.7 -9.999 -0.49 0.9

RS1452559 -0.68 0.8 -0.198 -0.05 0.7 -9.999 -0.49 0.9

RS207305 -0.68 0.8 -0.198 -0.05 0.7 -9.999 -0.49 0.9

RS7671224 -0.68 0.8 -0.198 -0.05 0.7 -9.999 -0.49 0.9

RS11939712 -0.68 0.8 -0.198 -0.05 0.7 -9.999 -0.49 0.9

RS4261984 -0.68 0.8 -0.198 -0.05 0.7 -9.999 -0.49 0.9

RS6858624 -0.68 0.8 -0.198 -0.05 0.7 -9.999 -0.49 0.9

RS13135681 -0.68 0.8 -0.198 -0.05 0.7 -9.999 -0.49 0.9

RS13435197 -0.68 0.8 -0.198 -0.05 0.7 -9.999 -0.49 0.9

RS3756147 -0.68 0.8 -0.198 -0.05 0.7 -9.999 -0.49 0.9

RS7666974 -0.68 0.8 -0.198 -0.05 0.7 -9.999 -0.49 0.9

RS2322559 -0.68 0.8 -0.198 -0.05 0.7 -9.999 -0.49 0.9

RS11930632 -0.68 0.8 -0.198 -0.05 0.7 -9.999 -0.49 0.9

RS1465522 -0.68 0.8 -0.198 -0.05 0.7 -9.999 -0.49 0.9

RS10024417 -0.68 0.8 -0.198 -0.05 0.7 -9.999 -0.49 0.9

RS10755201 -0.68 0.8 -0.198 -0.05 0.7 -9.999 -0.49 0.9

RS11721944 -0.68 0.8 -0.198 -0.05 0.7 -9.999 -0.49 0.9

RS1352797 -0.68 0.8 -0.198 -0.05 0.7 -9.999 -0.49 0.9

RS10001877 -0.68 0.8 -0.198 -0.05 0.7 -9.999 -0.49 0.9

RS9291414 -0.68 0.8 -0.198 -0.05 0.7 -9.999 -0.49 0.9

RS17556817 -0.68 0.8 -0.198 -0.05 0.7 -9.999 -0.49 0.9

RS6811024 -0.68 0.8 -0.198 -0.05 0.7 -9.999 -0.49 0.9

RS6825511 -0.68 0.8 -0.198 -0.05 0.7 -9.999 -0.49 0.9

RS7696887 -0.68 0.8 -0.198 -0.05 0.7 -9.999 -0.49 0.9

RS10011670 -0.68 0.8 -0.198 -0.05 0.7 -9.999 -0.49 0.9

RS1863289 -0.68 0.8 -0.198 -0.05 0.7 -9.999 -0.49 0.9

RS1425339 -0.68 0.8 -0.198 -0.05 0.7 -9.999 -0.49 0.9

RS7661530 -0.68 0.8 -0.198 -0.05 0.7 -9.999 -0.49 0.9

RS11730849 -0.68 0.8 -0.198 -0.05 0.7 -9.999 -0.49 0.9

RS16871434 -0.68 0.8 -0.198 -0.05 0.7 -9.999 -0.49 0.9

RS7676653 -0.68 0.8 -0.198 -0.05 0.7 -9.999 -0.49 0.9

RS2323070 -0.68 0.8 -0.198 -0.05 0.7 -9.999 -0.49 0.9

RS6448063 -0.68 0.8 -0.198 -0.05 0.7 -9.999 -0.49 0.9

RS1354390 -0.68 0.8 -0.198 -0.05 0.7 -9.999 -0.49 0.9

RS6850480 -0.68 0.8 -0.198 -0.05 0.7 -9.999 -0.49 0.9

RS10034770 -0.68 0.8 -0.198 -0.05 0.7 -9.999 -0.49 0.9

RS9291431 -0.68 0.8 -0.198 -0.05 0.7 -9.999 -0.49 0.9

RS11931022 -0.68 0.8 -0.198 -0.05 0.7 -9.999 -0.49 0.9

RS4697275 -0.68 0.8 -0.198 -0.05 0.7 -9.999 -0.49 0.9

RS7697959 -0.68 0.8 -0.198 -0.05 0.7 -9.999 -0.49 0.9

RS12163757 -0.68 0.8 -0.198 -0.05 0.7 -9.999 -0.49 0.9

RS2618940 -0.68 0.8 -0.198 -0.05 0.7 -9.999 -0.49 0.9

RS11946006 -0.68 0.8 -0.198 -0.05 0.7 -9.999 -0.49 0.9

RS11944694 -0.68 0.8 -0.198 -0.05 0.7 -9.999 -0.49 0.9

RS1396669 -0.68 0.8 -0.198 -0.05 0.7 -9.999 -0.49 0.9

RS215475 -0.68 0.8 -0.198 -0.05 0.7 -9.999 -0.49 0.9

RS12505641 -0.68 0.8 -0.198 -0.05 0.7 -9.999 -0.49 0.9

RS4317189 -0.68 0.8 -0.198 -0.05 0.7 -9.999 -0.49 0.9

RS13103252 -0.68 0.8 -0.198 -0.05 0.7 -9.999 -0.49 0.9

RS13121107 -0.68 0.8 -0.198 -0.05 0.7 -9.999 -0.49 0.9

RS13122003 -0.68 0.8 -0.198 -0.05 0.7 -9.999 -0.49 0.9

RS2932971 -0.68 0.8 -0.198 -0.05 0.7 -9.999 -0.49 0.9

RS4235308 -0.68 0.8 -0.198 -0.05 0.7 -9.999 -0.49 0.9

RS2970860 -0.68 0.8 -0.198 -0.05 0.7 -9.999 -0.49 0.9

RS2946403 -0.68 0.8 -0.198 -0.05 0.7 -9.999 -0.49 0.9

RS10025406 -0.68 0.8 -0.198 -0.05 0.7 -9.999 -0.49 0.9

RS7662477 -0.68 0.8 -0.198 -0.05 0.7 -9.999 -0.49 0.9

RS6448254 -0.68 0.8 -0.198 -0.05 0.7 -9.999 -0.49 0.9

RS11938023 -0.68 0.8 -0.198 -0.05 0.7 -9.999 -0.49 0.9

RS7690462 -0.68 0.8 -0.198 -0.05 0.7 -9.999 -0.49 0.9

RS6823107 -0.68 0.8 -0.198 -0.05 0.7 -9.999 -0.49 0.9

RS12506649 -0.62 0.7 -0.198 -0.05 0.7 -5.521 -0.31 0.9

RS13137535 -0.62 0.7 -0.198 -0.05 0.7 -5.521 -0.31 0.9

RS12507254 -0.62 0.7 -0.198 -0.05 0.7 -5.521 -0.31 0.9

RS6826722 -0.62 0.7 -0.198 -0.05 0.7 -5.521 -0.31 0.9

RS12501287 -0.62 0.7 -0.198 -0.05 0.7 -5.521 -0.31 0.9

RS13434451 -0.62 0.7 -0.198 -0.05 0.7 -5.521 -0.31 0.9

RS4697489 -0.62 0.7 -0.198 -0.05 0.7 -5.521 -0.31 0.9

RS4053966 -0.62 0.7 -0.198 -0.05 0.7 -5.521 -0.31 0.9

RS736831 -0.62 0.7 -0.198 -0.05 0.7 -5.521 -0.31 0.9

RS759245 -0.62 0.7 -0.198 -0.05 0.7 -5.521 -0.31 0.9

RS13139513 -0.62 0.7 -0.198 -0.05 0.7 -5.521 -0.31 0.9

RS12502384 -0.62 0.7 -0.198 -0.05 0.7 -5.521 -0.31 0.9

RS2667295 -0.62 0.7 -0.198 -0.05 0.7 -5.521 -0.31 0.9

RS10008104 -0.62 0.7 -0.198 -0.05 0.7 -5.521 -0.31 0.9

RS13114364 -0.62 0.7 -0.198 -0.05 0.7 -5.521 -0.31 0.9

RS4697098 -0.62 0.7 -0.198 -0.05 0.7 -5.521 -0.31 0.9

RS6851586 -0.62 0.7 -0.198 -0.05 0.7 -5.521 -0.31 0.9

RS4697584 -0.62 0.7 -0.198 -0.05 0.7 -5.521 -0.31 0.9

RS1980239 -0.62 0.7 -0.198 -0.05 0.7 -5.521 -0.31 0.9

RS6830401 -0.62 0.7 -0.198 -0.05 0.7 -5.521 -0.31 0.9

RS13147366 -0.56 0.7 -0.198 -0.05 0.7 -5.520 -0.29 0.9

RS3733543 0.58 0.3 1.309 0.25 0.14 -5.403 0.42 0.5

RS6831024 0.86 0.2 1.309 0.33 0.11 0.423 0.10 0.3

RS6820540 0.86 0.2 1.309 0.33 0.11 0.423 0.10 0.3

RS2309291 0.86 0.2 1.309 0.33 0.11 0.423 0.10 0.3

RS6847589 0.86 0.2 1.309 0.33 0.11 0.423 0.10 0.3

RS10155365 0.86 0.2 1.309 0.33 0.11 0.423 0.10 0.3

RS1512105 0.86 0.2 1.309 0.33 0.11 0.423 0.10 0.3

RS6824647 0.86 0.2 1.309 0.33 0.11 0.423 0.10 0.3

RS11735620 0.86 0.2 1.309 0.33 0.11 0.423 0.10 0.3

RS10805262 0.86 0.2 1.309 0.33 0.11 0.423 0.10 0.3

RS10517099 0.86 0.2 1.309 0.33 0.11 0.423 0.10 0.3

RS2130250 0.86 0.2 1.309 0.33 0.11 0.423 0.10 0.3

RS902096 0.86 0.2 1.309 0.33 0.11 0.423 0.10 0.3

RS7692829 0.86 0.2 1.309 0.33 0.11 0.423 0.10 0.3

RS6822297 0.86 0.2 1.309 0.33 0.11 0.423 0.10 0.3

RS7682298 0.86 0.2 1.309 0.33 0.11 0.423 0.10 0.3

RS2075093 0.86 0.2 1.309 0.33 0.11 0.423 0.10 0.3

RS7356206 0.86 0.2 1.309 0.33 0.11 0.423 0.10 0.3

RS9291495 0.86 0.2 1.309 0.33 0.11 0.423 0.10 0.3

RS4599400 0.86 0.2 1.309 0.33 0.11 0.423 0.10 0.3

RS16879587 0.86 0.2 1.309 0.33 0.11 0.423 0.10 0.3

RS1449766 0.86 0.2 1.309 0.33 0.11 0.423 0.10 0.3

RS2309628 0.86 0.2 1.309 0.33 0.11 0.423 0.10 0.3

RS929620 0.86 0.2 1.309 0.33 0.11 0.423 0.10 0.3

RS1609116 0.86 0.2 1.309 0.33 0.11 0.423 0.10 0.3

RS16880558 0.86 0.2 1.309 0.33 0.11 0.423 0.10 0.3

RS292016 0.86 0.2 1.309 0.33 0.11 0.423 0.10 0.3

RS3114003 0.86 0.2 1.309 0.33 0.11 0.423 0.10 0.3

RS7693029 0.86 0.2 1.309 0.33 0.11 0.423 0.10 0.3

RS12645808 0.86 0.2 1.309 0.33 0.11 0.423 0.10 0.3

RS12647787 0.86 0.2 1.309 0.33 0.11 0.423 0.10 0.3

RS12642441 0.86 0.2 1.309 0.33 0.11 0.423 0.10 0.3

RS11934543 0.86 0.2 1.309 0.33 0.11 0.423 0.10 0.3

RS6448638 0.86 0.2 1.309 0.33 0.11 0.423 0.10 0.3

RS166333 0.86 0.2 1.309 0.33 0.11 0.423 0.10 0.3

RS10033986 0.86 0.2 1.309 0.33 0.11 0.423 0.10 0.3

RS2310077 0.86 0.2 1.309 0.33 0.11 0.423 0.10 0.3

RS17735010 0.86 0.2 1.309 0.33 0.11 0.423 0.10 0.3

RS1394130 0.86 0.2 1.309 0.33 0.11 0.423 0.10 0.3

RS6836424 0.86 0.2 1.309 0.33 0.11 0.423 0.10 0.3

RS976057 0.86 0.2 1.309 0.33 0.11 0.423 0.10 0.3

RS1435787 0.86 0.2 1.309 0.33 0.11 0.423 0.10 0.3

RS10010739 0.86 0.2 1.309 0.33 0.11 0.423 0.10 0.3

RS1463844 0.86 0.2 1.309 0.33 0.11 0.423 0.10 0.3

RS1426985 0.86 0.2 1.309 0.33 0.11 0.423 0.10 0.3

RS933954 0.86 0.2 1.309 0.33 0.11 0.423 0.10 0.3

RS6448771 0.86 0.2 1.309 0.33 0.11 0.423 0.10 0.3

RS1966546 0.86 0.2 1.309 0.33 0.11 0.423 0.10 0.3

RS10428460 0.86 0.2 1.309 0.33 0.11 0.423 0.10 0.3

RS16885694 0.86 0.2 1.309 0.33 0.11 0.423 0.10 0.3

RS1157893 0.86 0.2 1.309 0.33 0.11 0.423 0.10 0.3

RS6553979 0.86 0.2 1.309 0.33 0.11 0.423 0.10 0.3

RS10027346 0.86 0.2 1.309 0.33 0.11 0.423 0.10 0.3

RS1388281 0.86 0.2 1.309 0.33 0.11 0.423 0.10 0.3

RS10016605 0.56 0.3 1.309 0.24 0.15 0.354 0.05 0.3

RS1377892 0.33 0.4 1.309 0.15 0.2 0.261 0.02 0.4

RS4862928 -0.22 0.6 -0.198 -0.02 0.6 -0.348 -0.01 0.6

RS7657618 -0.22 0.6 -0.198 -0.02 0.6 -0.348 -0.01 0.6

RS7680003 -0.22 0.6 -0.198 -0.02 0.6 -0.348 -0.01 0.6

RS11947379 -0.22 0.6 -0.198 -0.02 0.6 -0.348 -0.01 0.6

RS755773 -0.22 0.6 -0.198 -0.02 0.6 -0.348 -0.01 0.6

RS4862896 -0.22 0.6 -0.198 -0.02 0.6 -0.348 -0.01 0.6

RS7659735 -0.22 0.6 -0.198 -0.02 0.6 -0.348 -0.01 0.6

RS1349084 -0.22 0.6 -0.198 -0.02 0.6 -0.348 -0.01 0.6

RS2585909 -0.22 0.6 -0.198 -0.02 0.6 -0.348 -0.01 0.6

RS1378043 -0.22 0.6 -0.198 -0.02 0.6 -0.348 -0.01 0.6

RS17353301 -0.22 0.6 -0.198 -0.02 0.6 -0.348 -0.01 0.6

RS7654957 -0.22 0.6 -0.198 -0.02 0.6 -0.348 -0.01 0.6

RS10023150 -0.22 0.6 -0.198 -0.02 0.6 -0.348 -0.01 0.6

RS13434494 -0.22 0.6 -0.198 -0.02 0.6 -0.348 -0.01 0.6

RS7678322 -0.22 0.6 -0.198 -0.02 0.6 -0.348 -0.01 0.6

RS11943039 -0.22 0.6 -0.198 -0.02 0.6 -0.348 -0.01 0.6

RS1383561 -0.22 0.6 -0.198 -0.02 0.6 -0.348 -0.01 0.6

RS1600683 -0.22 0.6 -0.198 -0.02 0.6 -0.348 -0.01 0.6

RS7691687 -0.22 0.6 -0.198 -0.02 0.6 -0.348 -0.01 0.6

RS10016775 -0.22 0.6 -0.198 -0.02 0.6 -0.348 -0.01 0.6

RS9306924 -0.22 0.6 -0.198 -0.02 0.6 -0.348 -0.01 0.6

RS317014 -0.22 0.6 -0.198 -0.02 0.6 -0.348 -0.01 0.6

RS1022261 -0.22 0.6 -0.198 -0.02 0.6 -0.348 -0.01 0.6

RS12640371 -0.22 0.6 -0.198 -0.02 0.6 -0.348 -0.01 0.6

RS2375563 -0.22 0.6 -0.198 -0.02 0.6 -0.348 -0.01 0.6

RS6849048 -0.22 0.6 -0.198 -0.02 0.6 -0.348 -0.01 0.6

RS7668984 -0.22 0.6 -0.198 -0.02 0.6 -0.348 -0.01 0.6

RS13111222 -0.22 0.6 -0.198 -0.02 0.6 -0.348 -0.01 0.6

RS2889174 -0.22 0.6 -0.198 -0.02 0.6 -0.348 -0.01 0.6

RS13152028 -0.22 0.6 -0.198 -0.02 0.6 -0.348 -0.01 0.6

RS17516600 -0.22 0.6 -0.198 -0.02 0.6 -0.348 -0.01 0.6

RS949807 -0.22 0.6 -0.198 -0.02 0.6 -0.348 -0.01 0.6

RS12507648 -0.22 0.6 -0.198 -0.02 0.6 -0.348 -0.01 0.6

RS11936311 -0.22 0.6 -0.198 -0.02 0.6 -0.348 -0.01 0.6

RS10517382 -0.22 0.6 -0.198 -0.02 0.6 -0.348 -0.01 0.6

RS2376183 -0.22 0.6 -0.198 -0.02 0.6 -0.348 -0.01 0.6

RS12510063 -0.22 0.6 -0.198 -0.02 0.6 -0.348 -0.01 0.6

RS1023730 -0.22 0.6 -0.198 -0.02 0.6 -0.348 -0.01 0.6

RS13340226 -0.35 0.6 -0.198 -0.03 0.6 -0.802 -0.04 0.7

RS1002363 -0.35 0.6 -0.198 -0.03 0.6 -0.802 -0.04 0.7

RS10028027 -0.35 0.6 -0.198 -0.03 0.6 -0.802 -0.04 0.7

RS2973217 -0.35 0.6 -0.198 -0.03 0.6 -0.802 -0.04 0.7

RS1382984 -0.35 0.6 -0.198 -0.03 0.6 -0.802 -0.04 0.7

RS1552021 -0.35 0.6 -0.198 -0.03 0.6 -0.802 -0.04 0.7

RS2939739 -0.35 0.6 -0.198 -0.03 0.6 -0.802 -0.04 0.7

RS1876804 -0.35 0.6 -0.198 -0.03 0.6 -0.802 -0.04 0.7

RS16993717 -0.35 0.6 -0.198 -0.03 0.6 -0.802 -0.04 0.7

RS6818798 -0.35 0.6 -0.198 -0.03 0.6 -0.802 -0.04 0.7

RS7660340 -0.35 0.6 -0.198 -0.03 0.6 -0.802 -0.04 0.7

RS870953 -0.35 0.6 -0.198 -0.03 0.6 -0.802 -0.04 0.7

RS3775793 -0.35 0.6 -0.198 -0.03 0.6 -0.802 -0.04 0.7

RS9996566 -0.35 0.6 -0.198 -0.03 0.6 -0.802 -0.04 0.7

RS3893296 -0.35 0.6 -0.198 -0.03 0.6 -0.802 -0.04 0.7

RS6841167 -0.35 0.6 -0.198 -0.03 0.6 -0.802 -0.04 0.7

RS17579337 -0.35 0.6 -0.198 -0.03 0.6 -0.802 -0.04 0.7

RS1425576 -0.35 0.6 -0.198 -0.03 0.6 -0.802 -0.04 0.7

RS6844679 -0.35 0.6 -0.198 -0.03 0.6 -0.802 -0.04 0.7

RS6849562 -0.35 0.6 -0.198 -0.03 0.6 -0.802 -0.04 0.7

RS10012308 -0.35 0.6 -0.198 -0.03 0.6 -0.802 -0.04 0.7

RS6531617 -0.35 0.6 -0.198 -0.03 0.6 -0.802 -0.04 0.7

RS907314 -0.35 0.6 -0.198 -0.03 0.6 -0.802 -0.04 0.7

RS13133642 -0.35 0.6 -0.198 -0.03 0.6 -0.802 -0.04 0.7

RS10012289 -0.35 0.6 -0.198 -0.03 0.6 -0.802 -0.04 0.7

RS17615370 -0.35 0.6 -0.198 -0.03 0.6 -0.802 -0.04 0.7

RS2703904 -0.35 0.6 -0.198 -0.03 0.6 -0.802 -0.04 0.7

RS7678850 -0.35 0.6 -0.198 -0.03 0.6 -0.802 -0.04 0.7

RS6531673 -0.35 0.6 -0.198 -0.03 0.6 -0.802 -0.04 0.7

RS11946488 -0.35 0.6 -0.198 -0.03 0.6 -0.802 -0.04 0.7

RS2566104 -0.35 0.6 -0.198 -0.03 0.6 -0.802 -0.04 0.7

RS11096992 -0.35 0.6 -0.198 -0.03 0.6 -0.802 -0.04 0.7

RS6833806 -0.35 0.6 -0.198 -0.03 0.6 -0.802 -0.04 0.7

RS2890698 -0.35 0.6 -0.198 -0.03 0.6 -0.802 -0.04 0.7

RS6816573 -0.35 0.6 -0.198 -0.03 0.6 -0.802 -0.04 0.7

RS7686844 -0.35 0.6 -0.198 -0.03 0.6 -0.802 -0.04 0.7

RS4974961 -0.35 0.6 -0.198 -0.03 0.6 -0.802 -0.04 0.7

RS17586314 -0.35 0.6 -0.198 -0.03 0.6 -0.802 -0.04 0.7

RS11729556 -0.35 0.6 -0.198 -0.03 0.6 -0.802 -0.04 0.7

RS7667815 -0.35 0.6 -0.198 -0.03 0.6 -0.802 -0.04 0.7

RS9685160 -0.35 0.6 -0.198 -0.03 0.6 -0.802 -0.04 0.7

RS11728461 -0.35 0.6 -0.198 -0.03 0.6 -0.802 -0.04 0.7

RS10026108 -0.35 0.6 -0.198 -0.03 0.6 -0.802 -0.04 0.7

RS4861106 -0.35 0.6 -0.198 -0.03 0.6 -0.802 -0.04 0.7

RS2008438 -0.35 0.6 -0.198 -0.03 0.6 -0.802 -0.04 0.7

RS10018317 -0.35 0.6 -0.198 -0.03 0.6 -0.802 -0.04 0.7

RS278962 -0.35 0.6 -0.198 -0.03 0.6 -0.802 -0.04 0.7

RS13152696 -0.35 0.6 -0.198 -0.03 0.6 -0.802 -0.04 0.7

RS12498704 -0.35 0.6 -0.198 -0.03 0.6 -0.802 -0.04 0.7

RS790142 -0.35 0.6 -0.198 -0.03 0.6 -0.802 -0.04 0.7

RS2437320 -0.35 0.6 -0.198 -0.03 0.6 -0.802 -0.04 0.7

RS7690478 -0.35 0.6 -0.198 -0.03 0.6 -0.802 -0.04 0.7

RS1396110 -0.35 0.6 -0.198 -0.03 0.6 -0.802 -0.04 0.7

RS17528008 -0.35 0.6 -0.198 -0.03 0.6 -0.802 -0.04 0.7

RS11735648 -0.35 0.6 -0.198 -0.03 0.6 -0.802 -0.04 0.7

RS4349633 -0.35 0.6 -0.198 -0.03 0.6 -0.802 -0.04 0.7

RS4392535 -0.35 0.6 -0.198 -0.03 0.6 -0.802 -0.04 0.7

RS4861123 -0.35 0.6 -0.198 -0.03 0.6 -0.802 -0.04 0.7

RS6447103 -0.35 0.6 -0.198 -0.03 0.6 -0.802 -0.04 0.7

RS1061597 -0.35 0.6 -0.198 -0.03 0.6 -0.802 -0.04 0.7

RS6826373 -0.35 0.6 -0.198 -0.03 0.6 -0.802 -0.04 0.7

RS2043904 -0.35 0.6 -0.198 -0.03 0.6 -0.802 -0.04 0.7

RS16853775 -0.35 0.6 -0.198 -0.03 0.6 -0.802 -0.04 0.7

RS7672382 -0.35 0.6 -0.198 -0.03 0.6 -0.802 -0.04 0.7

RS9999533 -0.35 0.6 -0.198 -0.03 0.6 -0.802 -0.04 0.7

RS729179 -0.35 0.6 -0.198 -0.03 0.6 -0.802 -0.04 0.7

RS17530845 -0.35 0.6 -0.198 -0.03 0.6 -0.802 -0.04 0.7

RS748360 -0.35 0.6 -0.198 -0.03 0.6 -0.802 -0.04 0.7

RS11735318 -0.35 0.6 -0.198 -0.03 0.6 -0.802 -0.04 0.7

RS13139219 -0.35 0.6 -0.198 -0.03 0.6 -0.802 -0.04 0.7

RS898500 -0.35 0.6 -0.198 -0.03 0.6 -0.802 -0.04 0.7

RS6851867 -0.35 0.6 -0.198 -0.03 0.6 -0.802 -0.04 0.7

RS2612507 -0.35 0.6 -0.198 -0.03 0.6 -0.802 -0.04 0.7

RS1460352 -0.35 0.6 -0.198 -0.03 0.6 -0.802 -0.04 0.7

RS4861040 -0.35 0.6 -0.198 -0.03 0.6 -0.802 -0.04 0.7

RS1993059 -0.35 0.6 -0.198 -0.03 0.6 -0.802 -0.04 0.7

RS1450906 -0.35 0.6 -0.198 -0.03 0.6 -0.802 -0.04 0.7

RS6818868 -0.35 0.6 -0.198 -0.03 0.6 -0.802 -0.04 0.7

RS7672044 -0.35 0.6 -0.198 -0.03 0.6 -0.802 -0.04 0.7

RS12503562 -0.35 0.6 -0.198 -0.03 0.6 -0.802 -0.04 0.7

RS13114756 -0.35 0.6 -0.198 -0.03 0.6 -0.802 -0.04 0.7

RS13120255 -0.35 0.6 -0.198 -0.03 0.6 -0.802 -0.04 0.7

RS13113494 -0.35 0.6 -0.198 -0.03 0.6 -0.802 -0.04 0.7

RS4447896 -0.35 0.6 -0.198 -0.03 0.6 -0.802 -0.04 0.7

RS2347347 -0.35 0.6 -0.198 -0.03 0.6 -0.802 -0.04 0.7

RS6858778 -0.35 0.6 -0.198 -0.03 0.6 -0.802 -0.04 0.7

RS11941070 -0.35 0.6 -0.198 -0.03 0.6 -0.802 -0.04 0.7

RS1490452 -0.35 0.6 -0.198 -0.03 0.6 -0.802 -0.04 0.7

RS4312793 -0.35 0.6 -0.198 -0.03 0.6 -0.802 -0.04 0.7

RS16857230 -0.35 0.6 -0.198 -0.03 0.6 -0.802 -0.04 0.7

RS7682029 -0.35 0.6 -0.198 -0.03 0.6 -0.802 -0.04 0.7

RS13130484 -0.35 0.6 -0.198 -0.03 0.6 -0.802 -0.04 0.7

RS1476295 -0.35 0.6 -0.198 -0.03 0.6 -0.802 -0.04 0.7

RS6829634 -0.35 0.6 -0.198 -0.03 0.6 -0.802 -0.04 0.7

RS1456508 -0.35 0.6 -0.198 -0.03 0.6 -0.802 -0.04 0.7

RS1353014 -0.35 0.6 -0.198 -0.03 0.6 -0.802 -0.04 0.7

RS7683876 -0.35 0.6 -0.198 -0.03 0.6 -0.802 -0.04 0.7

RS13120165 -0.35 0.6 -0.198 -0.03 0.6 -0.802 -0.04 0.7

RS4695161 -0.35 0.6 -0.198 -0.03 0.6 -0.802 -0.04 0.7

RS9790574 -0.35 0.6 -0.198 -0.03 0.6 -0.802 -0.04 0.7

RS12503660 -0.35 0.6 -0.198 -0.03 0.6 -0.802 -0.04 0.7

RS953380 -0.35 0.6 -0.198 -0.03 0.6 -0.802 -0.04 0.7

RS6447520 -0.35 0.6 -0.198 -0.03 0.6 -0.802 -0.04 0.7

RS1866990 -0.35 0.6 -0.198 -0.03 0.6 -0.802 -0.04 0.7

RS1372499 -0.35 0.6 -0.198 -0.03 0.6 -0.802 -0.04 0.7

RS7665508 0.06 0.5 1.309 0.03 0.3 0.078 0.00 0.5

RS10517184 0.09 0.5 1.309 0.05 0.3 0.106 0.00 0.5

RS1062858 0.47 0.3 1.309 0.21 0.2 0.327 0.04 0.3

RS4695329 0.56 0.3 1.309 0.24 0.15 0.355 0.05 0.3

RS1567217 0.56 0.3 1.309 0.24 0.15 0.356 0.05 0.3

RS1509657 0.59 0.3 1.309 0.25 0.14 0.364 0.06 0.3

RS2664019 0.59 0.3 1.309 0.25 0.14 0.364 0.06 0.3

RS309875 0.60 0.3 1.309 0.25 0.14 0.366 0.06 0.3

RS3107023 0.61 0.3 1.309 0.25 0.14 0.369 0.06 0.3

RS11725957 0.63 0.3 1.309 0.26 0.14 0.375 0.06 0.3

RS225160 0.81 0.2 1.309 0.31 0.11 0.413 0.09 0.3

RS7692196 0.81 0.2 1.309 0.31 0.11 0.413 0.09 0.3

RS11731574 0.81 0.2 1.309 0.31 0.11 0.414 0.09 0.3

RS6834296 0.82 0.2 1.309 0.32 0.11 0.416 0.09 0.3

RS10026162 0.85 0.2 1.309 0.33 0.11 0.421 0.09 0.3

RS301126 0.86 0.2 1.309 0.33 0.11 0.422 0.09 0.3

RS1531836 0.86 0.2 1.309 0.33 0.11 0.422 0.09 0.3

RS2411290 0.86 0.2 1.309 0.33 0.11 0.422 0.09 0.3

RS7697542 0.86 0.2 1.309 0.33 0.11 0.423 0.10 0.3

RS2616404 0.86 0.2 1.309 0.33 0.11 0.423 0.10 0.3

RS2668532 0.86 0.2 1.309 0.33 0.11 0.423 0.10 0.3

RS6827185 0.86 0.2 1.309 0.33 0.11 0.423 0.10 0.3

RS1498821 0.86 0.2 1.309 0.33 0.11 0.423 0.10 0.3

RS1109789 0.86 0.2 1.309 0.33 0.11 0.423 0.10 0.3

RS7659596 0.86 0.2 1.309 0.33 0.11 0.423 0.10 0.3

RS17691810 0.86 0.2 1.309 0.33 0.11 0.423 0.10 0.3

RS1396066 0.86 0.2 1.309 0.33 0.11 0.423 0.10 0.3

RS17693577 0.86 0.2 1.309 0.33 0.11 0.423 0.10 0.3

RS2159935 0.86 0.2 1.309 0.33 0.11 0.423 0.10 0.3

RS2173231 0.86 0.2 1.309 0.33 0.11 0.423 0.10 0.3

RS2067951 0.86 0.2 1.309 0.33 0.11 0.423 0.10 0.3

RS11722442 0.86 0.2 1.309 0.33 0.11 0.423 0.10 0.3

RS7441352 0.76 0.2 1.309 0.30 0.12 0.401 0.08 0.3

RS11732292 0.71 0.2 1.309 0.28 0.13 0.387 0.07 0.3

RS13132085 0.23 0.4 1.309 0.12 0.2 0.187 0.01 0.4

RS12648836 0.12 0.5 1.309 0.06 0.3 0.103 0.00 0.5

RS11725155 0.08 0.5 1.309 0.04 0.3 0.070 0.00 0.5

RS2899048 0.08 0.5 1.309 0.04 0.3 0.070 0.00 0.5

RS2593075 0.08 0.5 1.309 0.04 0.3 0.070 0.00 0.5

RS2593068 0.08 0.5 1.309 0.04 0.3 0.070 0.00 0.5

RS6856844 0.08 0.5 1.309 0.04 0.3 0.070 0.00 0.5

RS1993916 0.08 0.5 1.309 0.04 0.3 0.070 0.00 0.5

RS140645 0.08 0.5 1.309 0.04 0.3 0.070 0.00 0.5

RS7690043 0.08 0.5 1.309 0.04 0.3 0.070 0.00 0.5

RS6842825 0.08 0.5 1.309 0.04 0.3 0.070 0.00 0.5

RS536002 0.08 0.5 1.309 0.04 0.3 0.070 0.00 0.5

RS10015634 0.08 0.5 1.309 0.04 0.3 0.070 0.00 0.5

RS4280802 0.08 0.5 1.309 0.04 0.3 0.070 0.00 0.5

RS4865131 0.08 0.5 1.309 0.04 0.3 0.070 0.00 0.5

RS4406078 0.08 0.5 1.309 0.04 0.3 0.070 0.00 0.5

RS6845520 0.08 0.5 1.309 0.04 0.3 0.070 0.00 0.5

RS4864599 0.08 0.5 1.309 0.04 0.3 0.070 0.00 0.5

RS1842295 0.08 0.5 1.309 0.04 0.3 0.070 0.00 0.5

RS6818605 0.08 0.5 1.309 0.04 0.3 0.070 0.00 0.5

RS13102386 0.08 0.5 1.309 0.04 0.3 0.070 0.00 0.5

RS2170073 0.08 0.5 1.309 0.04 0.3 0.070 0.00 0.5

RS1277311 0.08 0.5 1.309 0.04 0.3 0.070 0.00 0.5

RS1718861 0.08 0.5 1.309 0.04 0.3 0.070 0.00 0.5

RS4444899 0.08 0.5 1.309 0.04 0.3 0.070 0.00 0.5

RS7663355 0.08 0.5 1.309 0.04 0.3 0.070 0.00 0.5

RS10033422 0.08 0.5 1.309 0.04 0.3 0.070 0.00 0.5

RS1860621 0.08 0.5 1.309 0.04 0.3 0.070 0.00 0.5

RS10001448 0.08 0.5 1.309 0.04 0.3 0.070 0.00 0.5

RS11731338 0.08 0.5 1.309 0.04 0.3 0.070 0.00 0.5

RS1404372 0.08 0.5 1.309 0.04 0.3 0.069 0.00 0.5

RS7668422 0.08 0.5 1.309 0.04 0.3 0.068 0.00 0.5

RS6855484 0.08 0.5 1.309 0.04 0.3 0.068 0.00 0.5

RS1469235 0.07 0.5 1.309 0.04 0.3 0.066 0.00 0.5

RS3843894 0.07 0.5 1.309 0.04 0.3 0.061 0.00 0.5

RS922605 0.07 0.5 1.309 0.04 0.3 0.061 0.00 0.5

RS1480308 0.06 0.5 1.309 0.03 0.3 0.057 0.00 0.5

RS9637716 0.06 0.5 1.309 0.03 0.3 0.056 0.00 0.5

RS270233 0.05 0.5 1.309 0.03 0.4 0.049 0.00 0.5

RS2610166 0.05 0.5 1.309 0.03 0.4 0.047 0.00 0.5

RS1433409 0.04 0.5 1.309 0.02 0.4 0.035 0.00 0.5

RS3943387 0.02 0.5 1.309 0.01 0.4 0.023 0.00 0.5

RS10517441 -0.33 0.6 -0.198 -0.03 0.6 -0.731 -0.04 0.7

RS282720 -0.35 0.6 -0.198 -0.03 0.6 -0.802 -0.04 0.7

RS10033443 -0.35 0.6 -0.198 -0.03 0.6 -0.802 -0.04 0.7

RS11932542 -0.35 0.6 -0.198 -0.03 0.6 -0.802 -0.04 0.7

RS9942216 -0.35 0.6 -0.198 -0.03 0.6 -0.802 -0.04 0.7

RS13347305 -0.35 0.6 -0.198 -0.03 0.6 -0.802 -0.04 0.7

RS7664607 -0.35 0.6 -0.198 -0.03 0.6 -0.802 -0.04 0.7

RS970313 -0.35 0.6 -0.198 -0.03 0.6 -0.802 -0.04 0.7

RS11936764 -0.35 0.6 -0.198 -0.03 0.6 -0.802 -0.04 0.7

RS17089786 -0.35 0.6 -0.198 -0.03 0.6 -0.802 -0.04 0.7

RS7699730 -0.35 0.6 -0.198 -0.03 0.6 -0.802 -0.04 0.7

RS349211 -0.35 0.6 -0.198 -0.03 0.6 -0.802 -0.04 0.7

RS10000971 -0.35 0.6 -0.198 -0.03 0.6 -0.802 -0.04 0.7

RS6852374 -0.35 0.6 -0.198 -0.03 0.6 -0.802 -0.04 0.7

RS1553862 -0.35 0.6 -0.198 -0.03 0.6 -0.802 -0.04 0.7

RS11131268 -0.35 0.6 -0.198 -0.03 0.6 -0.802 -0.04 0.7

RS17081884 -0.35 0.6 -0.198 -0.03 0.6 -0.802 -0.04 0.7

RS6851977 -0.35 0.6 -0.198 -0.03 0.6 -0.802 -0.04 0.7

RS1486493 -0.35 0.6 -0.198 -0.03 0.6 -0.802 -0.04 0.7

RS12501471 -0.35 0.6 -0.198 -0.03 0.6 -0.802 -0.04 0.7

RS966084 -0.35 0.6 -0.198 -0.03 0.6 -0.802 -0.04 0.7

RS2053864 -0.35 0.6 -0.198 -0.03 0.6 -0.802 -0.04 0.7

RS10866114 -0.35 0.6 -0.198 -0.03 0.6 -0.802 -0.04 0.7

RS1505664 -0.35 0.6 -0.198 -0.03 0.6 -0.802 -0.04 0.7

RS11729528 -0.35 0.6 -0.198 -0.03 0.6 -0.802 -0.04 0.7

RS6821028 -0.35 0.6 -0.198 -0.03 0.6 -0.802 -0.04 0.7

RS10029192 -0.35 0.6 -0.198 -0.03 0.6 -0.802 -0.04 0.7

RS6813884 -0.35 0.6 -0.198 -0.03 0.6 -0.802 -0.04 0.7

RS12642037 -0.35 0.6 -0.198 -0.03 0.6 -0.802 -0.04 0.7

RS34855831 -0.35 0.6 -0.198 -0.03 0.6 -0.802 -0.04 0.7

RS11131347 -0.35 0.6 -0.198 -0.03 0.6 -0.802 -0.04 0.7

RS778554 -0.35 0.6 -0.198 -0.03 0.6 -0.802 -0.04 0.7

RS13126803 -0.35 0.6 -0.198 -0.03 0.6 -0.802 -0.04 0.7

RS6817682 -0.35 0.6 -0.198 -0.03 0.6 -0.802 -0.04 0.7

RS13116204 -0.35 0.6 -0.198 -0.03 0.6 -0.802 -0.04 0.7

RS12500171 -0.35 0.6 -0.198 -0.03 0.6 -0.802 -0.04 0.7

RS6815661 -0.35 0.6 -0.198 -0.03 0.6 -0.802 -0.04 0.7

RS1395745 -0.35 0.6 -0.198 -0.03 0.6 -0.802 -0.04 0.7

RS2346648 -0.35 0.6 -0.198 -0.03 0.6 -0.802 -0.04 0.7

RS7693548 -0.35 0.6 -0.198 -0.03 0.6 -0.802 -0.04 0.7

RS6551774 -0.35 0.6 -0.198 -0.03 0.6 -0.802 -0.04 0.7

RS7689785 -0.35 0.6 -0.198 -0.03 0.6 -0.802 -0.04 0.7

RS13133029 -0.35 0.6 -0.198 -0.03 0.6 -0.802 -0.04 0.7

RS1531202 -0.35 0.6 -0.198 -0.03 0.6 -0.802 -0.04 0.7

RS17084731 -0.35 0.6 -0.198 -0.03 0.6 -0.802 -0.04 0.7

RS12501821 -0.35 0.6 -0.198 -0.03 0.6 -0.802 -0.04 0.7

RS11725219 -0.35 0.6 -0.198 -0.03 0.6 -0.802 -0.04 0.7

RS1425377 -0.35 0.6 -0.198 -0.03 0.6 -0.802 -0.04 0.7

RS7664375 -0.35 0.6 -0.198 -0.03 0.6 -0.802 -0.04 0.7

RS13152245 -0.35 0.6 -0.198 -0.03 0.6 -0.802 -0.04 0.7

RS986208 -0.35 0.6 -0.198 -0.03 0.6 -0.802 -0.04 0.7

RS4860656 -0.35 0.6 -0.198 -0.03 0.6 -0.802 -0.04 0.7

RS2198103 -0.35 0.6 -0.198 -0.03 0.6 -0.802 -0.04 0.7

RS4337775 -0.35 0.6 -0.198 -0.03 0.6 -0.802 -0.04 0.7

RS7654891 -0.35 0.6 -0.198 -0.03 0.6 -0.802 -0.04 0.7

RS11947738 -0.35 0.6 -0.198 -0.03 0.6 -0.802 -0.04 0.7

RS4588513 -0.35 0.6 -0.198 -0.03 0.6 -0.802 -0.04 0.7

RS7660970 -0.35 0.6 -0.198 -0.03 0.6 -0.802 -0.04 0.7

RS10023064 -0.35 0.6 -0.198 -0.03 0.6 -0.802 -0.04 0.7

RS13102033 -0.35 0.6 -0.198 -0.03 0.6 -0.802 -0.04 0.7

RS7669583 -0.35 0.6 -0.198 -0.03 0.6 -0.802 -0.04 0.7

RS11934386 -0.35 0.6 -0.198 -0.03 0.6 -0.802 -0.04 0.7

RS1397453 -0.35 0.6 -0.198 -0.03 0.6 -0.802 -0.04 0.7

RS10866173 -0.35 0.6 -0.198 -0.03 0.6 -0.802 -0.04 0.7

RS1353320 -0.35 0.6 -0.198 -0.03 0.6 -0.802 -0.04 0.7

RS923994 -0.35 0.6 -0.198 -0.03 0.6 -0.802 -0.04 0.7

RS1390067 -0.35 0.6 -0.198 -0.03 0.6 -0.802 -0.04 0.7

RS730934 -0.35 0.6 -0.198 -0.03 0.6 -0.802 -0.04 0.7

RS4639131 -0.30 0.6 -0.198 -0.02 0.6 -0.591 -0.03 0.6

RS10008670 -0.22 0.6 -0.198 -0.02 0.6 -0.335 -0.01 0.6

RS13119483 -0.22 0.6 -0.198 -0.02 0.6 -0.335 -0.01 0.6

RS1348079 -0.22 0.6 -0.198 -0.02 0.6 -0.335 -0.01 0.6

RS6849403 -0.22 0.6 -0.198 -0.02 0.6 -0.335 -0.01 0.6

RS17088850 -0.22 0.6 -0.198 -0.02 0.6 -0.335 -0.01 0.6

RS7659406 -0.22 0.6 -0.198 -0.02 0.6 -0.335 -0.01 0.6

RS4860296 -0.22 0.6 -0.198 -0.02 0.6 -0.335 -0.01 0.6

RS4148271 -0.22 0.6 -0.198 -0.02 0.6 -0.335 -0.01 0.6

RS9994887 -0.22 0.6 -0.198 -0.02 0.6 -0.335 -0.01 0.6

RS4576082 -0.22 0.6 -0.198 -0.02 0.6 -0.335 -0.01 0.6

RS6600900 -0.22 0.6 -0.198 -0.02 0.6 -0.335 -0.01 0.6

RS13133166 -0.22 0.6 -0.198 -0.02 0.6 -0.335 -0.01 0.6

RS10031583 -0.22 0.6 -0.198 -0.02 0.6 -0.335 -0.01 0.6

RS13107309 -0.22 0.6 -0.198 -0.02 0.6 -0.335 -0.01 0.6

RS9329032 -0.22 0.6 -0.198 -0.02 0.6 -0.335 -0.01 0.6

RS17148794 -0.22 0.6 -0.198 -0.02 0.6 -0.335 -0.01 0.6

RS4024031 -0.22 0.6 -0.198 -0.02 0.6 -0.335 -0.01 0.6

RS28661403 -0.22 0.6 -0.198 -0.02 0.6 -0.335 -0.01 0.6

RS12512502 -0.22 0.6 -0.198 -0.02 0.6 -0.335 -0.01 0.6

RS1979537 -0.22 0.6 -0.198 -0.02 0.6 -0.335 -0.01 0.6

RS12506660 -0.22 0.6 -0.198 -0.02 0.6 -0.335 -0.01 0.6

RS6854303 -0.22 0.6 -0.198 -0.02 0.6 -0.335 -0.01 0.6

RS1453458 -0.22 0.6 -0.198 -0.02 0.6 -0.335 -0.01 0.6

RS6446756 -0.22 0.6 -0.198 -0.02 0.6 -0.335 -0.01 0.6

RS6837549 -0.22 0.6 -0.198 -0.02 0.6 -0.335 -0.01 0.6

RS962227 -0.22 0.6 -0.198 -0.02 0.6 -0.335 -0.01 0.6

RS1520499 -0.22 0.6 -0.198 -0.02 0.6 -0.335 -0.01 0.6

RS12512773 -0.22 0.6 -0.198 -0.02 0.6 -0.335 -0.01 0.6

RS957045 -0.22 0.6 -0.198 -0.02 0.6 -0.335 -0.01 0.6

RS12645174 -0.22 0.6 -0.198 -0.02 0.6 -0.335 -0.01 0.6

RS12644269 -0.22 0.6 -0.198 -0.02 0.6 -0.335 -0.01 0.6

RS316440 -0.22 0.6 -0.198 -0.02 0.6 -0.335 -0.01 0.6

RS6840693 -0.22 0.6 -0.198 -0.02 0.6 -0.335 -0.01 0.6

RS961873 -0.22 0.6 -0.198 -0.02 0.6 -0.335 -0.01 0.6

RS10050235 -0.22 0.6 -0.198 -0.02 0.6 -0.335 -0.01 0.6

RS2051512 -0.22 0.6 -0.198 -0.02 0.6 -0.335 -0.01 0.6

RS9996759 -0.22 0.6 -0.198 -0.02 0.6 -0.335 -0.01 0.6

RS4694627 -0.22 0.6 -0.198 -0.02 0.6 -0.335 -0.01 0.6

RS1951242 -0.22 0.6 -0.198 -0.02 0.6 -0.335 -0.01 0.6

RS3117598 -0.22 0.6 -0.198 -0.02 0.6 -0.335 -0.01 0.6

RS2472649 -0.22 0.6 -0.198 -0.02 0.6 -0.335 -0.01 0.6

RS1837559 -0.22 0.6 -0.198 -0.02 0.6 -0.335 -0.01 0.6

RS62314947 -0.22 0.6 -0.198 -0.02 0.6 -0.335 -0.01 0.6

RS6836453 -0.22 0.6 -0.198 -0.02 0.6 -0.335 -0.01 0.6

RS11097027 -0.22 0.6 -0.198 -0.02 0.6 -0.335 -0.01 0.6

RS4412061 -0.22 0.6 -0.198 -0.02 0.6 -0.335 -0.01 0.6

RS977266 -0.22 0.6 -0.198 -0.02 0.6 -0.335 -0.01 0.6

RS984991 -0.22 0.6 -0.198 -0.02 0.6 -0.335 -0.01 0.6

RS896577 -0.22 0.6 -0.198 -0.02 0.6 -0.335 -0.01 0.6

RS1062293 -0.22 0.6 -0.198 -0.02 0.6 -0.335 -0.01 0.6

RS9090 -0.22 0.6 -0.198 -0.02 0.6 -0.335 -0.01 0.6

RS11944489 -0.22 0.6 -0.198 -0.02 0.6 -0.335 -0.01 0.6

RS4859943 -0.22 0.6 -0.198 -0.02 0.6 -0.335 -0.01 0.6

RS11942566 -0.22 0.6 -0.198 -0.02 0.6 -0.335 -0.01 0.6

RS6535270 -0.22 0.6 -0.198 -0.02 0.6 -0.335 -0.01 0.6

RS3853187 -0.22 0.6 -0.198 -0.02 0.6 -0.335 -0.01 0.6

RS10857225 -0.22 0.6 -0.198 -0.02 0.6 -0.335 -0.01 0.6

RS1553985 -0.22 0.6 -0.198 -0.02 0.6 -0.335 -0.01 0.6

RS13147538 -0.22 0.6 -0.198 -0.02 0.6 -0.335 -0.01 0.6

RS17001357 -0.22 0.6 -0.198 -0.02 0.6 -0.335 -0.01 0.6

RS6532208 -0.22 0.6 -0.198 -0.02 0.6 -0.335 -0.01 0.6

RS17001561 -0.22 0.6 -0.198 -0.02 0.6 -0.335 -0.01 0.6

RS2289514 -0.22 0.6 -0.198 -0.02 0.6 -0.335 -0.01 0.6

RS3733248 -0.22 0.6 -0.198 -0.02 0.6 -0.335 -0.01 0.6

RS4859682 -0.22 0.6 -0.198 -0.02 0.6 -0.335 -0.01 0.6

RS6817593 -0.22 0.6 -0.198 -0.02 0.6 -0.335 -0.01 0.6

RS7687284 -0.22 0.6 -0.198 -0.02 0.6 -0.335 -0.01 0.6

RS4859711 -0.22 0.6 -0.198 -0.02 0.6 -0.335 -0.01 0.6

RS6821290 -0.22 0.6 -0.198 -0.02 0.6 -0.335 -0.01 0.6

RS6810774 -0.22 0.6 -0.198 -0.02 0.6 -0.335 -0.01 0.6

RS6532676 -0.22 0.6 -0.198 -0.02 0.6 -0.335 -0.01 0.6

RS2703147 -0.22 0.6 -0.198 -0.02 0.6 -0.335 -0.01 0.6

RS2703111 -0.22 0.6 -0.198 -0.02 0.6 -0.335 -0.01 0.6

RS883698 -0.22 0.6 -0.198 -0.02 0.6 -0.335 -0.01 0.6

RS11937061 -0.22 0.6 -0.198 -0.02 0.6 -0.335 -0.01 0.6

RS17396361 -0.22 0.6 -0.198 -0.02 0.6 -0.335 -0.01 0.6

RS11726795 -0.22 0.6 -0.198 -0.02 0.6 -0.335 -0.01 0.6

RS1596231 -0.22 0.6 -0.198 -0.02 0.6 -0.335 -0.01 0.6

RS920666 -0.22 0.6 -0.198 -0.02 0.6 -0.335 -0.01 0.6

RS17002804 -0.22 0.6 -0.198 -0.02 0.6 -0.335 -0.01 0.6

RS7439347 -0.22 0.6 -0.198 -0.02 0.6 -0.335 -0.01 0.6

RS11940556 -0.22 0.6 -0.198 -0.02 0.6 -0.335 -0.01 0.6

RS9284617 -0.25 0.6 -0.198 -0.02 0.6 -0.415 -0.02 0.6

RS366463 -0.38 0.6 -0.198 -0.03 0.6 -0.995 -0.06 0.7

RS17003174 -0.40 0.7 -0.198 -0.03 0.7 -1.099 -0.07 0.7

RS931602 -0.40 0.7 -0.198 -0.03 0.7 -1.099 -0.07 0.7

RS4975154 -0.40 0.7 -0.198 -0.03 0.7 -1.099 -0.07 0.7

RS11728698 -0.40 0.7 -0.198 -0.03 0.7 -1.099 -0.07 0.7

RS6852257 -0.40 0.7 -0.198 -0.03 0.7 -1.099 -0.07 0.7

RS1378376 -0.40 0.7 -0.198 -0.03 0.7 -1.099 -0.07 0.7

RS4975172 -0.40 0.7 -0.198 -0.03 0.7 -1.099 -0.07 0.7

RS4401473 -0.40 0.7 -0.198 -0.03 0.7 -1.099 -0.07 0.7

RS6534361 -0.40 0.7 -0.198 -0.03 0.7 -1.099 -0.07 0.7

RS10027646 -0.40 0.7 -0.198 -0.03 0.7 -1.099 -0.07 0.7

RS1371990 -0.40 0.7 -0.198 -0.03 0.7 -1.099 -0.07 0.7

RS4362850 -0.40 0.7 -0.198 -0.03 0.7 -1.099 -0.07 0.7

RS13134009 -0.40 0.7 -0.198 -0.03 0.7 -1.099 -0.07 0.7

RS4632591 -0.40 0.7 -0.198 -0.03 0.7 -1.099 -0.07 0.7

RS6857026 -0.40 0.7 -0.198 -0.03 0.7 -1.099 -0.07 0.7

RS4308327 -0.40 0.7 -0.198 -0.03 0.7 -1.099 -0.07 0.7

RS6839672 -0.40 0.7 -0.198 -0.03 0.7 -1.099 -0.07 0.7

RS13102102 -0.40 0.7 -0.198 -0.03 0.7 -1.099 -0.07 0.7

RS17004844 -0.40 0.7 -0.198 -0.03 0.7 -1.099 -0.07 0.7

RS17004846 -0.40 0.7 -0.198 -0.03 0.7 -1.099 -0.07 0.7

RS1379706 -0.40 0.7 -0.198 -0.03 0.7 -1.085 -0.06 0.7

RS7664124 -0.39 0.7 -0.198 -0.03 0.7 -1.052 -0.06 0.7

RS2055177 -0.32 0.6 -0.198 -0.03 0.6 -0.683 -0.04 0.7

RS2685462 -0.30 0.6 -0.198 -0.03 0.6 -0.612 -0.03 0.6

RS710839 -0.29 0.6 -0.198 -0.02 0.6 -0.547 -0.03 0.6

RS7681976 -0.27 0.6 -0.198 -0.02 0.6 -0.500 -0.02 0.6

RS2159657 -0.26 0.6 -0.198 -0.02 0.6 -0.450 -0.02 0.6

RS1011631 -0.25 0.6 -0.198 -0.02 0.6 -0.414 -0.02 0.6

RS10019003 -0.25 0.6 -0.198 -0.02 0.6 -0.414 -0.02 0.6

RS10022264 -0.25 0.6 -0.198 -0.02 0.6 -0.414 -0.02 0.6

RS11099493 -0.25 0.6 -0.198 -0.02 0.6 -0.414 -0.02 0.6

RS751104 -0.25 0.6 -0.198 -0.02 0.6 -0.414 -0.02 0.6

RS10516668 -0.25 0.6 -0.198 -0.02 0.6 -0.414 -0.02 0.6

RS2131361 -0.25 0.6 -0.198 -0.02 0.6 -0.414 -0.02 0.6

RS10009173 -0.25 0.6 -0.198 -0.02 0.6 -0.414 -0.02 0.6

RS6822283 -0.25 0.6 -0.198 -0.02 0.6 -0.414 -0.02 0.6

RS6843699 -0.25 0.6 -0.198 -0.02 0.6 -0.414 -0.02 0.6

RS17005836 -0.25 0.6 -0.198 -0.02 0.6 -0.414 -0.02 0.6

RS10009661 -0.25 0.6 -0.198 -0.02 0.6 -0.414 -0.02 0.6

RS897945 -0.25 0.6 -0.198 -0.02 0.6 -0.414 -0.02 0.6

RS12510343 -0.25 0.6 -0.198 -0.02 0.6 -0.414 -0.02 0.6

RS13122305 -0.25 0.6 -0.198 -0.02 0.6 -0.414 -0.02 0.6

RS10025120 -0.25 0.6 -0.198 -0.02 0.6 -0.414 -0.02 0.6

RS4693608 -0.25 0.6 -0.198 -0.02 0.6 -0.414 -0.02 0.6

RS4693614 -0.25 0.6 -0.198 -0.02 0.6 -0.414 -0.02 0.6

RS10004897 -0.25 0.6 -0.198 -0.02 0.6 -0.414 -0.02 0.6

RS11099613 -0.25 0.6 -0.198 -0.02 0.6 -0.414 -0.02 0.6

RS6821747 -0.25 0.6 -0.198 -0.02 0.6 -0.414 -0.02 0.6

RS10004130 -0.25 0.6 -0.198 -0.02 0.6 -0.414 -0.02 0.6

RS13120080 -0.25 0.6 -0.198 -0.02 0.6 -0.414 -0.02 0.6

RS10516704 -0.25 0.6 -0.198 -0.02 0.6 -0.414 -0.02 0.6

RS2017802 -0.25 0.6 -0.198 -0.02 0.6 -0.414 -0.02 0.6

RS1343762 -0.25 0.6 -0.198 -0.02 0.6 -0.413 -0.02 0.6

RS6827060 -0.25 0.6 -0.198 -0.02 0.6 -0.413 -0.02 0.6

RS1017557 -0.25 0.6 -0.198 -0.02 0.6 -0.413 -0.02 0.6

RS10516727 -0.25 0.6 -0.198 -0.02 0.6 -0.413 -0.02 0.6

RS340185 -0.25 0.6 -0.198 -0.02 0.6 -0.414 -0.02 0.6

RS1481775 -0.25 0.6 -0.198 -0.02 0.6 -0.414 -0.02 0.6

RS1026873 -0.25 0.6 -0.198 -0.02 0.6 -0.414 -0.02 0.6

RS1482085 -0.25 0.6 -0.198 -0.02 0.6 -0.414 -0.02 0.6

RS12498568 -0.25 0.6 -0.198 -0.02 0.6 -0.414 -0.02 0.6

RS6834349 -0.25 0.6 -0.198 -0.02 0.6 -0.414 -0.02 0.6

RS2665869 -0.25 0.6 -0.198 -0.02 0.6 -0.414 -0.02 0.6

RS346480 -0.25 0.6 -0.198 -0.02 0.6 -0.414 -0.02 0.6

RS2575675 -0.25 0.6 -0.198 -0.02 0.6 -0.414 -0.02 0.6

RS12640395 -0.25 0.6 -0.198 -0.02 0.6 -0.414 -0.02 0.6

RS11725943 -0.25 0.6 -0.198 -0.02 0.6 -0.414 -0.02 0.6

RS10213444 -0.25 0.6 -0.198 -0.02 0.6 -0.414 -0.02 0.6

RS2121871 -0.25 0.6 -0.198 -0.02 0.6 -0.414 -0.02 0.6

RS6836128 -0.25 0.6 -0.198 -0.02 0.6 -0.414 -0.02 0.6

RS340630 -0.25 0.6 -0.198 -0.02 0.6 -0.414 -0.02 0.6

RS4134363 -0.25 0.6 -0.198 -0.02 0.6 -0.414 -0.02 0.6

RS13138404 -0.25 0.6 -0.198 -0.02 0.6 -0.414 -0.02 0.6

RS4610302 -0.25 0.6 -0.198 -0.02 0.6 -0.414 -0.02 0.6

RS1381633 -0.25 0.6 -0.198 -0.02 0.6 -0.414 -0.02 0.6

RS6822632 -0.25 0.6 -0.198 -0.02 0.6 -0.414 -0.02 0.6

RS10028688 -0.25 0.6 -0.198 -0.02 0.6 -0.414 -0.02 0.6

RS12650760 -0.25 0.6 -0.198 -0.02 0.6 -0.414 -0.02 0.6

RS7655902 -0.25 0.6 -0.198 -0.02 0.6 -0.414 -0.02 0.6

RS17013516 -0.25 0.6 -0.198 -0.02 0.6 -0.414 -0.02 0.6

RS2725225 -0.25 0.6 -0.198 -0.02 0.6 -0.414 -0.02 0.6

RS2231148 -0.25 0.6 -0.198 -0.02 0.6 -0.414 -0.02 0.6

RS10856870 -0.25 0.6 -0.198 -0.02 0.6 -0.414 -0.02 0.6

RS881561 -0.25 0.6 -0.198 -0.02 0.6 -0.414 -0.02 0.6

RS10022462 -0.25 0.6 -0.198 -0.02 0.6 -0.414 -0.02 0.6

RS4693954 -0.25 0.6 -0.198 -0.02 0.6 -0.414 -0.02 0.6

RS12651141 -0.25 0.6 -0.198 -0.02 0.6 -0.414 -0.02 0.6

RS3846281 -0.25 0.6 -0.198 -0.02 0.6 -0.414 -0.02 0.6

RS6850944 -0.25 0.6 -0.198 -0.02 0.6 -0.414 -0.02 0.6

RS9991328 -0.25 0.6 -0.198 -0.02 0.6 -0.414 -0.02 0.6

RS2704588 -0.25 0.6 -0.198 -0.02 0.6 -0.414 -0.02 0.6

RS16996144 -0.25 0.6 -0.198 -0.02 0.6 -0.414 -0.02 0.6

RS1795734 -0.25 0.6 -0.198 -0.02 0.6 -0.414 -0.02 0.6

RS756345 -0.25 0.6 -0.198 -0.02 0.6 -0.414 -0.02 0.6

RS518156 -0.27 0.6 -0.198 -0.02 0.6 -0.470 -0.02 0.6

RS1495552 -0.35 0.6 -0.198 -0.03 0.6 -0.801 -0.04 0.7

RS903600 -0.35 0.6 -0.198 -0.03 0.6 -0.802 -0.04 0.7

RS17015738 -0.35 0.6 -0.198 -0.03 0.6 -0.802 -0.04 0.7

RS6852411 -0.35 0.6 -0.198 -0.03 0.6 -0.802 -0.04 0.7

RS1812923 -0.35 0.6 -0.198 -0.03 0.6 -0.802 -0.04 0.7

RS13114320 -0.35 0.6 -0.198 -0.03 0.6 -0.802 -0.04 0.7

RS6834254 -0.35 0.6 -0.198 -0.03 0.6 -0.802 -0.04 0.7

RS17017104 -0.35 0.6 -0.198 -0.03 0.6 -0.802 -0.04 0.7

RS6532233 -0.35 0.6 -0.198 -0.03 0.6 -0.802 -0.04 0.7

RS6532239 -0.35 0.6 -0.198 -0.03 0.6 -0.802 -0.04 0.7

RS6819554 -0.35 0.6 -0.198 -0.03 0.6 -0.802 -0.04 0.7

RS12508842 -0.35 0.6 -0.198 -0.03 0.6 -0.802 -0.04 0.7

RS1544017 -0.35 0.6 -0.198 -0.03 0.6 -0.802 -0.04 0.7

RS6532345 -0.35 0.6 -0.198 -0.03 0.6 -0.802 -0.04 0.7

RS4455379 -0.35 0.6 -0.198 -0.03 0.6 -0.802 -0.04 0.7

RS1844346 -0.35 0.6 -0.198 -0.03 0.6 -0.802 -0.04 0.7

RS2199291 -0.35 0.6 -0.198 -0.03 0.6 -0.802 -0.04 0.7

RS8180202 -0.35 0.6 -0.198 -0.03 0.6 -0.802 -0.04 0.7

RS1513460 -0.35 0.6 -0.198 -0.03 0.6 -0.802 -0.04 0.7

RS7659159 -0.35 0.6 -0.198 -0.03 0.6 -0.802 -0.04 0.7

RS7661958 -0.35 0.6 -0.198 -0.03 0.6 -0.802 -0.04 0.7

RS17019538 -0.35 0.6 -0.198 -0.03 0.6 -0.802 -0.04 0.7

RS17019814 -0.35 0.6 -0.198 -0.03 0.6 -0.802 -0.04 0.7

RS954467 -0.35 0.6 -0.198 -0.03 0.6 -0.802 -0.04 0.7

RS6834311 -0.35 0.6 -0.198 -0.03 0.6 -0.802 -0.04 0.7

RS13144042 -0.35 0.6 -0.198 -0.03 0.6 -0.802 -0.04 0.7

RS11728147 -0.35 0.6 -0.198 -0.03 0.6 -0.802 -0.04 0.7

RS6848888 -0.35 0.6 -0.198 -0.03 0.6 -0.802 -0.04 0.7

RS4692987 -0.35 0.6 -0.198 -0.03 0.6 -0.802 -0.04 0.7

RS10024195 -0.35 0.6 -0.198 -0.03 0.6 -0.802 -0.04 0.7

RS1841925 -0.35 0.6 -0.198 -0.03 0.6 -0.802 -0.04 0.7

RS2452600 -0.35 0.6 -0.198 -0.03 0.6 -0.802 -0.04 0.7

RS7680214 -0.35 0.6 -0.198 -0.03 0.6 -0.802 -0.04 0.7

RS6846079 -0.35 0.6 -0.198 -0.03 0.6 -0.802 -0.04 0.7

RS2719175 -0.35 0.6 -0.198 -0.03 0.6 -0.802 -0.04 0.7

RS3755876 -0.35 0.6 -0.198 -0.03 0.6 -0.802 -0.04 0.7

RS10010112 -0.35 0.6 -0.198 -0.03 0.6 -0.802 -0.04 0.7

RS2865421 -0.35 0.6 -0.198 -0.03 0.6 -0.802 -0.04 0.7

RS2621458 -0.35 0.6 -0.198 -0.03 0.6 -0.802 -0.04 0.7

RS6532555 -0.35 0.6 -0.198 -0.03 0.6 -0.802 -0.04 0.7

RS3846457 -0.35 0.6 -0.198 -0.03 0.6 -0.802 -0.04 0.7

RS3846452 -0.35 0.6 -0.198 -0.03 0.6 -0.802 -0.04 0.7

RS6532561 -0.35 0.6 -0.198 -0.03 0.6 -0.802 -0.04 0.7

RS10032931 -0.35 0.6 -0.198 -0.03 0.6 -0.802 -0.04 0.7

RS11097474 -0.35 0.6 -0.198 -0.03 0.6 -0.802 -0.04 0.7

RS977737 -0.35 0.6 -0.198 -0.03 0.6 -0.802 -0.04 0.7

RS13147380 -0.35 0.6 -0.198 -0.03 0.6 -0.802 -0.04 0.7

RS6851495 -0.35 0.6 -0.198 -0.03 0.6 -0.802 -0.04 0.7

RS5012929 -0.35 0.6 -0.198 -0.03 0.6 -0.802 -0.04 0.7

RS6819078 -0.35 0.6 -0.198 -0.03 0.6 -0.802 -0.04 0.7

RS13131407 -0.35 0.6 -0.198 -0.03 0.6 -0.802 -0.04 0.7

RS6823094 -0.35 0.6 -0.198 -0.03 0.6 -0.802 -0.04 0.7

RS10026084 -0.35 0.6 -0.198 -0.03 0.6 -0.802 -0.04 0.7

RS1840042 -0.35 0.6 -0.198 -0.03 0.6 -0.802 -0.04 0.7

RS13124250 -0.35 0.6 -0.198 -0.03 0.6 -0.802 -0.04 0.7

RS7685841 -0.35 0.6 -0.198 -0.03 0.6 -0.802 -0.04 0.7

RS6532654 -0.35 0.6 -0.198 -0.03 0.6 -0.802 -0.04 0.7

RS929290 -0.35 0.6 -0.198 -0.03 0.6 -0.802 -0.04 0.7

RS10488826 -0.35 0.6 -0.198 -0.03 0.6 -0.793 -0.04 0.7

RS981391 -0.34 0.6 -0.198 -0.03 0.6 -0.792 -0.04 0.7

RS733323 -0.34 0.6 -0.198 -0.03 0.6 -0.792 -0.04 0.7

RS2865763 -0.34 0.6 -0.198 -0.03 0.6 -0.792 -0.04 0.7

RS10856927 -0.34 0.6 -0.198 -0.03 0.6 -0.791 -0.04 0.7

RS7671104 -0.34 0.6 -0.198 -0.03 0.6 -0.792 -0.04 0.7

RS4699665 -0.34 0.6 -0.198 -0.03 0.6 -0.792 -0.04 0.7

RS971169 -0.35 0.6 -0.198 -0.03 0.6 -0.793 -0.04 0.7

RS6532765 -0.35 0.6 -0.198 -0.03 0.6 -0.793 -0.04 0.7

RS1867343 -0.35 0.6 -0.198 -0.03 0.6 -0.793 -0.04 0.7

RS11723037 -0.35 0.6 -0.198 -0.03 0.6 -0.795 -0.04 0.7

RS1037475 -0.35 0.6 -0.198 -0.03 0.6 -0.797 -0.04 0.7

RS1803037 -0.35 0.6 -0.198 -0.03 0.6 -0.799 -0.04 0.7

RS7687322 -0.35 0.6 -0.198 -0.03 0.6 -0.800 -0.04 0.7

RS4148883 -0.35 0.6 -0.198 -0.03 0.6 -0.802 -0.04 0.7

RS7678936 -0.35 0.6 -0.198 -0.03 0.6 -0.802 -0.04 0.7

RS2173199 -0.35 0.6 -0.198 -0.03 0.6 -0.802 -0.04 0.7

RS35743364 -0.35 0.6 -0.198 -0.03 0.6 -0.802 -0.04 0.7

RS283415 -0.35 0.6 -0.198 -0.03 0.6 -0.802 -0.04 0.7

RS1442490 -0.35 0.6 -0.198 -0.03 0.6 -0.802 -0.04 0.7

RS729147 -0.35 0.6 -0.198 -0.03 0.6 -0.802 -0.04 0.7

RS1154476 -0.35 0.6 -0.198 -0.03 0.6 -0.802 -0.04 0.7

RS9994042 -0.35 0.6 -0.198 -0.03 0.6 -0.802 -0.04 0.7

RS868911 -0.35 0.6 -0.198 -0.03 0.6 -0.802 -0.04 0.7

RS10001464 -0.35 0.6 -0.198 -0.03 0.6 -0.802 -0.04 0.7

RS4699395 -0.35 0.6 -0.198 -0.03 0.6 -0.802 -0.04 0.7

RS4235450 -0.35 0.6 -0.198 -0.03 0.6 -0.802 -0.04 0.7

RS1019947 -0.35 0.6 -0.198 -0.03 0.6 -0.802 -0.04 0.7

RS11097720 -0.35 0.6 -0.198 -0.03 0.6 -0.802 -0.04 0.7

RS992834 -0.35 0.6 -0.198 -0.03 0.6 -0.802 -0.04 0.7

RS4699824 -0.35 0.6 -0.198 -0.03 0.6 -0.802 -0.04 0.7

RS6847657 -0.35 0.6 -0.198 -0.03 0.6 -0.802 -0.04 0.7

RS2178006 -0.35 0.6 -0.198 -0.03 0.6 -0.802 -0.04 0.7

RS13112866 -0.35 0.6 -0.198 -0.03 0.6 -0.802 -0.04 0.7

RS17031441 -0.35 0.6 -0.198 -0.03 0.6 -0.802 -0.04 0.7

RS7663310 -0.35 0.6 -0.198 -0.03 0.6 -0.802 -0.04 0.7

RS17250305 -0.35 0.6 -0.198 -0.03 0.6 -0.802 -0.04 0.7

RS7682523 -0.35 0.6 -0.198 -0.03 0.6 -0.802 -0.04 0.7

RS10031210 -0.35 0.6 -0.198 -0.03 0.6 -0.802 -0.04 0.7

RS11934097 -0.35 0.6 -0.198 -0.03 0.6 -0.802 -0.04 0.7

RS7667281 -0.35 0.6 -0.198 -0.03 0.6 -0.802 -0.04 0.7

RS2165265 -0.35 0.6 -0.198 -0.03 0.6 -0.802 -0.04 0.7

RS6843751 -0.35 0.6 -0.198 -0.03 0.6 -0.802 -0.04 0.7

RS13115343 -0.35 0.6 -0.198 -0.03 0.6 -0.802 -0.04 0.7

RS230535 -0.35 0.6 -0.198 -0.03 0.6 -0.802 -0.04 0.7

RS223374 -0.35 0.6 -0.198 -0.03 0.6 -0.802 -0.04 0.7

RS3796974 -0.35 0.6 -0.198 -0.03 0.6 -0.802 -0.04 0.7

RS10516503 -0.35 0.6 -0.198 -0.03 0.6 -0.802 -0.04 0.7

RS11733295 -0.35 0.6 -0.198 -0.03 0.6 -0.802 -0.04 0.7

RS6837683 -0.35 0.6 -0.198 -0.03 0.6 -0.802 -0.04 0.7

RS2866673 -0.35 0.6 -0.198 -0.03 0.6 -0.802 -0.04 0.7

RS12645446 -0.35 0.6 -0.198 -0.03 0.6 -0.802 -0.04 0.7

RS882920 -0.35 0.6 -0.198 -0.03 0.6 -0.802 -0.04 0.7

RS10002598 -0.35 0.6 -0.198 -0.03 0.6 -0.802 -0.04 0.7

RS9307295 -0.35 0.6 -0.198 -0.03 0.6 -0.802 -0.04 0.7

RS10516510 -0.35 0.6 -0.198 -0.03 0.6 -0.802 -0.04 0.7

RS3017707 -0.35 0.6 -0.198 -0.03 0.6 -0.802 -0.04 0.7

RS3017722 -0.35 0.6 -0.198 -0.03 0.6 -0.802 -0.04 0.7

RS624909 -0.35 0.6 -0.198 -0.03 0.6 -0.802 -0.04 0.7

RS2522502 -0.35 0.6 -0.198 -0.03 0.6 -0.802 -0.04 0.7

RS6533163 -0.35 0.6 -0.198 -0.03 0.6 -0.802 -0.04 0.7

RS2726485 -0.35 0.6 -0.198 -0.03 0.6 -0.802 -0.04 0.7

RS2074396 -0.35 0.6 -0.198 -0.03 0.6 -0.802 -0.04 0.7

RS199330 -0.06 0.5 -0.198 -0.01 0.6 -0.108 -0.00 0.5

RS715706 -0.05 0.5 -0.198 -0.00 0.6 -0.078 -0.00 0.5

RS4699193 0.06 0.5 1.309 0.03 0.3 0.076 0.00 0.5

RS4340795 0.40 0.3 1.309 0.18 0.2 0.300 0.03 0.4

RS4597836 0.43 0.3 1.309 0.19 0.2 0.312 0.03 0.3

RS7690115 0.73 0.2 1.309 0.29 0.12 0.398 0.08 0.3

RS2686305 0.76 0.2 1.309 0.30 0.12 0.404 0.08 0.3

RS4956275 0.84 0.2 1.309 0.32 0.11 0.419 0.09 0.3

RS17510289 0.85 0.2 1.309 0.33 0.11 0.421 0.09 0.3

RS7694364 0.86 0.2 1.309 0.33 0.11 0.422 0.09 0.3

RS4403120 0.86 0.2 1.309 0.33 0.11 0.422 0.09 0.3

RS6827724 0.86 0.2 1.309 0.33 0.11 0.422 0.10 0.3

RS717435 0.86 0.2 1.309 0.33 0.11 0.422 0.10 0.3

RS6847720 0.86 0.2 1.309 0.33 0.11 0.423 0.10 0.3

RS975219 0.86 0.2 1.309 0.33 0.11 0.423 0.10 0.3

RS4956035 0.86 0.2 1.309 0.33 0.11 0.423 0.10 0.3

RS3796999 0.86 0.2 1.309 0.33 0.11 0.423 0.10 0.3

RS219457 0.86 0.2 1.309 0.33 0.11 0.423 0.10 0.3

RS6838036 0.86 0.2 1.309 0.33 0.11 0.423 0.10 0.3

RS11734670 0.86 0.2 1.309 0.33 0.11 0.423 0.10 0.3

RS2077246 0.86 0.2 1.309 0.33 0.11 0.423 0.10 0.3

RS7692671 0.86 0.2 1.309 0.33 0.11 0.423 0.10 0.3

RS7686077 0.86 0.2 1.309 0.33 0.11 0.423 0.10 0.3

RS884049 0.86 0.2 1.309 0.33 0.11 0.423 0.10 0.3

RS1405160 0.86 0.2 1.309 0.33 0.11 0.423 0.10 0.3

RS4368681 0.86 0.2 1.309 0.33 0.11 0.423 0.10 0.3

RS4512071 0.86 0.2 1.309 0.33 0.11 0.423 0.10 0.3

RS10516556 0.86 0.2 1.309 0.33 0.11 0.423 0.10 0.3

RS10032323 0.86 0.2 1.309 0.33 0.11 0.423 0.10 0.3

RS9685587 0.86 0.2 1.309 0.33 0.11 0.423 0.10 0.3

RS34415611 0.86 0.2 1.309 0.33 0.11 0.423 0.10 0.3

RS2035415 0.86 0.2 1.309 0.33 0.11 0.423 0.10 0.3

RS7656124 0.86 0.2 1.309 0.33 0.11 0.423 0.10 0.3

RS763298 0.86 0.2 1.309 0.33 0.11 0.423 0.10 0.3

RS244005 0.86 0.2 1.309 0.33 0.11 0.423 0.10 0.3

RS17041821 0.86 0.2 1.309 0.33 0.11 0.423 0.10 0.3

RS1982360 0.86 0.2 1.309 0.33 0.11 0.423 0.10 0.3

RS11098092 0.86 0.2 1.309 0.33 0.11 0.423 0.10 0.3

RS536099 0.86 0.2 1.309 0.33 0.11 0.423 0.10 0.3

RS10002518 0.86 0.2 1.309 0.33 0.11 0.423 0.10 0.3

RS159439 0.86 0.2 1.309 0.33 0.11 0.423 0.10 0.3

RS297012 0.86 0.2 1.309 0.33 0.11 0.423 0.10 0.3

RS17042944 0.86 0.2 1.309 0.33 0.11 0.423 0.10 0.3

RS17043035 0.86 0.2 1.309 0.33 0.11 0.423 0.10 0.3

RS17576345 0.86 0.2 1.309 0.33 0.11 0.423 0.10 0.3

RS4834781 0.86 0.2 1.309 0.33 0.11 0.423 0.10 0.3

RS7684254 0.86 0.2 1.309 0.33 0.11 0.423 0.10 0.3

RS3109360 0.86 0.2 1.309 0.33 0.11 0.423 0.10 0.3

RS924758 0.86 0.2 1.309 0.33 0.11 0.423 0.10 0.3

RS980387 0.86 0.2 1.309 0.33 0.11 0.423 0.10 0.3

RS2717207 0.86 0.2 1.309 0.33 0.11 0.423 0.10 0.3

RS6843121 0.86 0.2 1.309 0.33 0.11 0.423 0.10 0.3

RS4834106 0.86 0.2 1.309 0.33 0.11 0.423 0.10 0.3

RS12510189 0.86 0.2 1.309 0.33 0.11 0.423 0.10 0.3

RS1544382 0.86 0.2 1.309 0.33 0.11 0.423 0.10 0.3

RS7664678 0.86 0.2 1.309 0.33 0.11 0.423 0.10 0.3

RS11936846 0.86 0.2 1.309 0.33 0.11 0.423 0.10 0.3

RS10001894 0.86 0.2 1.309 0.33 0.11 0.423 0.10 0.3

RS6851781 0.86 0.2 1.309 0.33 0.11 0.423 0.10 0.3

RS1448221 0.86 0.2 1.309 0.33 0.11 0.423 0.10 0.3

RS13115459 0.86 0.2 1.309 0.33 0.11 0.423 0.10 0.3

RS10015472 0.86 0.2 1.309 0.33 0.11 0.423 0.10 0.3

RS592670 0.86 0.2 1.309 0.33 0.11 0.423 0.10 0.3

RS7697503 0.86 0.2 1.309 0.33 0.11 0.423 0.10 0.3

RS4834321 0.86 0.2 1.309 0.33 0.11 0.423 0.10 0.3

RS29394 0.86 0.2 1.309 0.33 0.11 0.423 0.10 0.3

RS1525004 0.86 0.2 1.309 0.33 0.11 0.423 0.10 0.3

RS916874 0.86 0.2 1.309 0.33 0.11 0.423 0.10 0.3

RS10000775 0.86 0.2 1.309 0.33 0.11 0.423 0.10 0.3

RS6812422 0.86 0.2 1.309 0.33 0.11 0.423 0.10 0.3

RS4345183 0.86 0.2 1.309 0.33 0.11 0.423 0.10 0.3

RS6822455 0.86 0.2 1.309 0.33 0.11 0.423 0.10 0.3

RS766183 0.86 0.2 1.309 0.33 0.11 0.423 0.10 0.3

RS17581675 0.86 0.2 1.309 0.33 0.11 0.423 0.10 0.3

RS7340953 0.86 0.2 1.309 0.33 0.11 0.423 0.10 0.3

RS2353563 0.86 0.2 1.309 0.33 0.11 0.423 0.10 0.3

RS7660554 4.38 0.00001 1.309 0.83 0.03 1.427 1.36 0.006

RS7675371 4.39 0.00001 1.309 0.83 0.03 1.427 1.36 0.006

RS13131555 4.39 0.00001 1.309 0.83 0.03 1.427 1.36 0.006

RS1430934 4.39 0.00001 1.309 0.83 0.03 1.427 1.36 0.006

RS1456365 4.39 0.00001 1.309 0.83 0.03 1.427 1.36 0.006

RS6854280 4.39 0.00001 1.309 0.83 0.03 1.427 1.36 0.006

RS1906699 4.39 0.00001 1.309 0.83 0.03 1.427 1.36 0.006

RS13124273 4.39 0.00001 1.309 0.83 0.03 1.427 1.36 0.006

RS4640736 4.39 0.00001 1.309 0.83 0.03 1.427 1.36 0.006

RS12507901 4.39 0.00001 1.309 0.83 0.03 1.427 1.36 0.006

RS298928 4.39 0.00001 1.309 0.83 0.03 1.427 1.36 0.006

RS13147087 4.39 0.00001 1.309 0.83 0.03 1.427 1.36 0.006

RS13138792 4.39 0.00001 1.309 0.83 0.03 1.427 1.36 0.006

RS6534034 4.39 0.00001 1.309 0.83 0.03 1.427 1.36 0.006

RS17861918 4.39 0.00001 1.309 0.83 0.03 1.427 1.36 0.006

RS2245104 4.39 0.00001 1.309 0.83 0.03 1.427 1.36 0.006

RS13129382 4.39 0.00001 1.309 0.83 0.03 1.427 1.36 0.006

RS7678262 4.39 0.00001 1.309 0.83 0.03 1.427 1.36 0.006

RS788663 4.39 0.00001 1.309 0.83 0.03 1.427 1.36 0.006

RS7689541 4.39 0.00001 1.309 0.83 0.03 1.427 1.36 0.006

RS10019751 4.39 0.00001 1.309 0.83 0.03 1.427 1.36 0.006

RS2389655 4.39 0.00001 1.309 0.83 0.03 1.427 1.36 0.006

RS7690236 4.39 0.00001 1.309 0.83 0.03 1.427 1.36 0.006

RS7676506 4.39 0.00001 1.309 0.83 0.03 1.427 1.36 0.006

RS1021377 4.39 0.00001 1.309 0.83 0.03 1.427 1.36 0.006

RS9307465 4.39 0.00001 1.309 0.83 0.03 1.427 1.36 0.006

RS4834733 4.39 0.00001 1.309 0.83 0.03 1.427 1.36 0.006

RS1459056 4.39 0.00001 1.309 0.83 0.03 1.427 1.36 0.006

RS9784534 4.39 0.00001 1.309 0.83 0.03 1.427 1.36 0.006

RS11098498 4.39 0.00001 1.309 0.83 0.03 1.427 1.36 0.006

RS12646525 4.39 0.00001 1.309 0.83 0.03 1.427 1.36 0.006

RS9994651 4.39 0.00001 1.309 0.83 0.03 1.427 1.36 0.006

RS7699214 4.39 0.00001 1.309 0.83 0.03 1.427 1.36 0.006

RS2389923 4.39 0.00001 1.309 0.83 0.03 1.427 1.36 0.006

RS7677531 4.39 0.00001 1.309 0.83 0.03 1.427 1.36 0.006

RS13121806 4.39 0.00001 1.309 0.83 0.03 1.427 1.36 0.006

RS705103 4.39 0.00001 1.309 0.83 0.03 1.427 1.36 0.006

RS1685593 4.39 0.00001 1.309 0.83 0.03 1.427 1.36 0.006

RS17050662 4.39 0.00001 1.309 0.83 0.03 1.427 1.36 0.006

RS10024986 4.39 0.00001 1.309 0.83 0.03 1.427 1.36 0.006

RS17343008 4.39 0.00001 1.309 0.83 0.03 1.427 1.36 0.006

RS7675777 4.39 0.00001 1.309 0.83 0.03 1.427 1.36 0.006

RS11723050 4.39 0.00001 1.309 0.83 0.03 1.427 1.36 0.006

RS6824931 4.39 0.00001 1.309 0.83 0.03 1.427 1.36 0.006

RS3733560 4.39 0.00001 1.309 0.83 0.03 1.427 1.36 0.006

RS13142482 4.45 0.00000 1.309 0.83 0.03 1.534 1.41 0.005

RS4642269 4.61 0.00000 1.309 0.85 0.02 2.310 1.54 0.004

RS4425401 4.70 0.00000 1.309 0.85 0.02 3.125 1.62 0.003

RS577737 4.70 0.00000 1.309 0.85 0.02 3.125 1.62 0.003

RS1513702 4.70 0.00000 1.309 0.85 0.02 3.125 1.62 0.003

RS4833736 4.70 0.00000 1.309 0.85 0.02 3.125 1.62 0.003

RS9685971 4.70 0.00000 1.309 0.85 0.02 3.125 1.62 0.003

RS13145977 4.70 0.00000 1.309 0.85 0.02 3.125 1.62 0.003

RS11732306 4.70 0.00000 1.309 0.85 0.02 3.125 1.62 0.003

RS11934307 4.70 0.00000 1.309 0.85 0.02 3.125 1.62 0.003

RS11726196 4.70 0.00000 1.309 0.85 0.02 3.125 1.62 0.003

RS1358229 4.70 0.00000 1.309 0.85 0.02 3.125 1.62 0.003

RS1880864 4.70 0.00000 1.309 0.85 0.02 3.125 1.62 0.003

RS7682281 4.70 0.00000 1.309 0.85 0.02 3.125 1.62 0.003

RS309414 4.70 0.00000 1.309 0.85 0.02 3.126 1.62 0.003

RS2292493 4.70 0.00000 1.309 0.85 0.02 3.126 1.62 0.003

RS10021762 4.70 0.00000 1.309 0.85 0.02 3.126 1.62 0.003

RS11098710 4.70 0.00000 1.309 0.85 0.02 3.126 1.62 0.003

RS10461205 4.70 0.00000 1.309 0.85 0.02 3.126 1.62 0.003

RS13115814 4.70 0.00000 1.309 0.85 0.02 3.126 1.62 0.003

RS986345 4.70 0.00000 1.309 0.85 0.02 3.125 1.62 0.003

RS4833961 4.70 0.00000 1.309 0.85 0.02 3.125 1.62 0.003

RS17007771 4.70 0.00000 1.309 0.85 0.02 3.125 1.62 0.003

RS17008117 4.70 0.00000 1.309 0.85 0.02 3.125 1.62 0.003

RS13119813 4.70 0.00000 1.309 0.85 0.02 3.125 1.62 0.003

RS9993168 4.70 0.00000 1.309 0.85 0.02 3.125 1.62 0.003

RS1909100 4.70 0.00000 1.309 0.85 0.02 3.125 1.62 0.003

RS1498040 4.70 0.00000 1.309 0.85 0.02 3.125 1.62 0.003

RS6534458 4.70 0.00000 1.309 0.85 0.02 3.125 1.62 0.003

RS6534467 4.70 0.00000 1.309 0.85 0.02 3.125 1.62 0.003

RS10034706 4.70 0.00000 1.309 0.85 0.02 3.125 1.62 0.003

RS17009641 4.70 0.00000 1.309 0.85 0.02 3.125 1.62 0.003

RS1506360 4.70 0.00000 1.309 0.85 0.02 3.125 1.62 0.003

RS1567049 4.70 0.00000 1.309 0.85 0.02 3.125 1.62 0.003

RS13140903 4.70 0.00000 1.309 0.85 0.02 3.125 1.62 0.003

RS4590050 4.70 0.00000 1.309 0.85 0.02 3.125 1.62 0.003

RS3956582 4.70 0.00000 1.309 0.85 0.02 3.125 1.62 0.003

RS10026567 4.70 0.00000 1.309 0.85 0.02 3.125 1.62 0.003

RS561662 4.70 0.00000 1.309 0.85 0.02 3.125 1.62 0.003

RS17010784 4.70 0.00000 1.309 0.85 0.02 3.125 1.62 0.003

RS12500408 4.70 0.00000 1.309 0.85 0.02 3.125 1.62 0.003

RS12509279 4.70 0.00000 1.309 0.85 0.02 3.125 1.62 0.003

RS12647099 4.70 0.00000 1.309 0.85 0.02 3.125 1.62 0.003

RS1543000 4.70 0.00000 1.309 0.85 0.02 3.125 1.62 0.003

RS313045 4.70 0.00000 1.309 0.85 0.02 3.125 1.62 0.003

RS313055 4.70 0.00000 1.309 0.85 0.02 3.125 1.62 0.003

RS7688921 4.70 0.00000 1.309 0.85 0.02 3.125 1.62 0.003

RS17012435 4.70 0.00000 1.309 0.85 0.02 3.125 1.62 0.003

RS1008995 4.70 0.00000 1.309 0.85 0.02 3.125 1.62 0.003

RS10434049 4.70 0.00000 1.309 0.85 0.02 3.125 1.62 0.003

RS2305958 4.70 0.00000 1.309 0.85 0.02 3.125 1.62 0.003

RS4975181 4.70 0.00000 1.309 0.85 0.02 3.125 1.62 0.003

RS1216412 4.70 0.00000 1.309 0.85 0.02 3.125 1.62 0.003

RS1615523 4.70 0.00000 1.309 0.85 0.02 3.125 1.62 0.003

RS1553142 4.70 0.00000 1.309 0.85 0.02 3.125 1.62 0.003

RS13147397 4.70 0.00000 1.309 0.85 0.02 3.125 1.62 0.003

RS11098986 4.70 0.00000 1.309 0.85 0.02 3.125 1.62 0.003

RS9991981 4.70 0.00000 1.309 0.85 0.02 3.125 1.62 0.003

RS2034497 4.70 0.00000 1.309 0.85 0.02 3.125 1.61 0.003

RS10857138 4.70 0.00000 1.309 0.85 0.02 3.125 1.61 0.003

RS512552 4.69 0.00000 1.309 0.85 0.02 3.125 1.61 0.003

RS477926 4.69 0.00000 1.309 0.85 0.02 3.125 1.61 0.003

RS11933154 4.68 0.00000 1.309 0.85 0.02 3.125 1.61 0.003

RS10001834 4.68 0.00000 1.309 0.85 0.02 3.125 1.61 0.003

RS995637 4.68 0.00000 1.309 0.85 0.02 3.125 1.61 0.003

RS17372420 4.65 0.00000 1.309 0.85 0.02 3.125 1.61 0.003

RS17439675 4.63 0.00000 1.309 0.85 0.02 3.125 1.61 0.003

RS2637613 4.57 0.00000 1.309 0.84 0.02 3.125 1.60 0.003

RS6827640 4.54 0.00000 1.309 0.84 0.02 3.125 1.60 0.003

RS6534761 3.91 0.00005 1.309 0.79 0.03 3.126 1.54 0.004

RS11933661 3.79 0.00008 1.309 0.78 0.03 3.126 1.53 0.004

RS11099040 3.46 0.0003 1.309 0.74 0.03 3.126 1.50 0.004

RS10024383 3.22 0.0006 1.309 0.72 0.03 3.126 1.47 0.005

RS7662168 3.08 0.0010 1.309 0.70 0.04 3.126 1.45 0.005

RS12502992 1.00 0.2 1.309 0.36 0.10 3.125 0.96 0.02

RS306364 -0.60 0.7 -0.198 -0.05 0.7 -4.397 -0.26 0.9

RS7658306 -0.60 0.7 -0.198 -0.05 0.7 -4.397 -0.26 0.9

RS1948720 -0.60 0.7 -0.198 -0.05 0.7 -4.397 -0.26 0.9

RS500131 -0.60 0.7 -0.198 -0.05 0.7 -4.397 -0.26 0.9

RS17051169 -0.60 0.7 -0.198 -0.05 0.7 -4.397 -0.26 0.9

RS9992696 -0.60 0.7 -0.198 -0.05 0.7 -4.397 -0.26 0.9

RS350995 -0.60 0.7 -0.198 -0.05 0.7 -4.397 -0.26 0.9

RS4863859 -0.60 0.7 -0.198 -0.05 0.7 -4.396 -0.26 0.9

RS1499346 -0.60 0.7 -0.198 -0.05 0.7 -4.396 -0.26 0.9

RS7689868 -0.60 0.7 -0.198 -0.05 0.7 -4.396 -0.26 0.9

RS12645565 -0.60 0.7 -0.198 -0.05 0.7 -4.396 -0.26 0.9

RS1037177 -0.60 0.7 -0.198 -0.05 0.7 -4.396 -0.26 0.9

RS11099230 -0.60 0.7 -0.198 -0.05 0.7 -4.396 -0.26 0.9

RS7675150 -0.60 0.7 -0.198 -0.05 0.7 -4.396 -0.26 0.9

RS6846610 -0.60 0.7 -0.198 -0.05 0.7 -4.396 -0.26 0.9

RS2009274 -0.60 0.7 -0.198 -0.05 0.7 -4.396 -0.26 0.9

RS1389331 -0.60 0.7 -0.198 -0.05 0.7 -4.396 -0.26 0.9

RS1948964 -0.60 0.7 -0.198 -0.05 0.7 -4.396 -0.26 0.9

RS7699345 -0.60 0.7 -0.198 -0.05 0.7 -4.396 -0.26 0.9

RS6811342 -0.60 0.7 -0.198 -0.05 0.7 -4.396 -0.26 0.9

RS2901383 -0.60 0.7 -0.198 -0.05 0.7 -4.396 -0.26 0.9

RS17037260 -0.60 0.7 -0.198 -0.05 0.7 -4.396 -0.26 0.9

RS11934048 -0.60 0.7 -0.198 -0.05 0.7 -4.396 -0.26 0.9

RS7684927 -0.60 0.7 -0.198 -0.05 0.7 -4.396 -0.26 0.9

RS13102973 -0.60 0.7 -0.198 -0.05 0.7 -4.396 -0.26 0.9

RS4507423 -0.60 0.7 -0.198 -0.05 0.7 -4.396 -0.26 0.9

RS7687865 -0.60 0.7 -0.198 -0.05 0.7 -4.396 -0.26 0.9

RS10034725 -0.60 0.7 -0.198 -0.05 0.7 -4.396 -0.26 0.9

RS10009456 -0.60 0.7 -0.198 -0.05 0.7 -4.396 -0.26 0.9

RS984160 -0.60 0.7 -0.198 -0.05 0.7 -4.396 -0.26 0.9

RS7340923 -0.60 0.7 -0.198 -0.05 0.7 -4.396 -0.26 0.9

RS7666345 -0.60 0.7 -0.198 -0.05 0.7 -4.396 -0.26 0.9

RS1504865 -0.60 0.7 -0.198 -0.05 0.7 -4.396 -0.26 0.9

RS7655183 -0.60 0.7 -0.198 -0.05 0.7 -4.396 -0.26 0.9

RS4311361 -0.60 0.7 -0.198 -0.05 0.7 -4.396 -0.26 0.9

RS4455480 -0.60 0.7 -0.198 -0.05 0.7 -4.396 -0.26 0.9

RS4863947 -0.60 0.7 -0.198 -0.05 0.7 -4.396 -0.26 0.9

RS13102201 -0.60 0.7 -0.198 -0.05 0.7 -4.396 -0.26 0.9

RS6848692 -0.60 0.7 -0.198 -0.05 0.7 -4.396 -0.26 0.9

RS11733040 -0.60 0.7 -0.198 -0.05 0.7 -4.396 -0.26 0.9

RS2405826 -0.60 0.7 -0.198 -0.05 0.7 -4.396 -0.26 0.9

RS6812959 -0.60 0.7 -0.198 -0.05 0.7 -4.396 -0.26 0.9

RS2125883 -0.60 0.7 -0.198 -0.05 0.7 -4.396 -0.26 0.9

RS9996792 -0.60 0.7 -0.198 -0.05 0.7 -4.396 -0.26 0.9

RS7692053 -0.60 0.7 -0.198 -0.05 0.7 -4.396 -0.26 0.9

RS693365 -0.60 0.7 -0.198 -0.05 0.7 -4.396 -0.26 0.9

RS1400918 -0.60 0.7 -0.198 -0.05 0.7 -4.396 -0.26 0.9

RS1517945 -0.60 0.7 -0.198 -0.05 0.7 -4.396 -0.26 0.9

RS534612 -0.60 0.7 -0.198 -0.05 0.7 -4.396 -0.26 0.9

RS10519416 -0.60 0.7 -0.198 -0.05 0.7 -4.396 -0.26 0.9

RS17049362 -0.60 0.7 -0.198 -0.05 0.7 -4.396 -0.26 0.9

RS1425983 -0.60 0.7 -0.198 -0.05 0.7 -4.396 -0.26 0.9

RS6816675 -0.60 0.7 -0.198 -0.05 0.7 -4.396 -0.26 0.9

RS11730485 -0.60 0.7 -0.198 -0.05 0.7 -4.396 -0.26 0.9

RS4864420 -0.60 0.7 -0.198 -0.05 0.7 -4.396 -0.26 0.9

RS13112145 -0.60 0.7 -0.198 -0.05 0.7 -4.396 -0.26 0.9

RS4478233 -0.60 0.7 -0.198 -0.05 0.7 -4.396 -0.26 0.9

RS10021969 -0.60 0.7 -0.198 -0.05 0.7 -4.396 -0.26 0.9

RS2462870 -0.60 0.7 -0.198 -0.05 0.7 -4.396 -0.26 0.9

RS7667668 -0.60 0.7 -0.198 -0.05 0.7 -4.396 -0.26 0.9

RS4863772 -0.60 0.7 -0.198 -0.05 0.7 -4.396 -0.26 0.9

RS1513765 -0.60 0.7 -0.198 -0.05 0.7 -4.396 -0.26 0.9

RS6817763 -0.60 0.7 -0.198 -0.05 0.7 -4.396 -0.26 0.9

RS7670671 -0.60 0.7 -0.198 -0.05 0.7 -4.396 -0.26 0.9

RS7657271 -0.60 0.7 -0.198 -0.05 0.7 -4.396 -0.26 0.9

RS2976499 -0.60 0.7 -0.198 -0.05 0.7 -4.396 -0.26 0.9

RS2976490 -0.60 0.7 -0.198 -0.05 0.7 -4.396 -0.26 0.9

RS9308208 -0.60 0.7 -0.198 -0.05 0.7 -4.396 -0.26 0.9

RS13108353 -0.60 0.7 -0.198 -0.05 0.7 -4.396 -0.26 0.9

RS299113 -0.60 0.7 -0.198 -0.05 0.7 -4.396 -0.26 0.9

RS1817031 -0.60 0.7 -0.198 -0.05 0.7 -4.396 -0.26 0.9

RS1450453 -0.60 0.7 -0.198 -0.05 0.7 -4.396 -0.26 0.9

RS12502933 -0.60 0.7 -0.198 -0.05 0.7 -4.396 -0.26 0.9

RS7692637 -0.60 0.7 -0.198 -0.05 0.7 -4.396 -0.26 0.9

RS10020229 -0.60 0.7 -0.198 -0.05 0.7 -4.396 -0.26 0.9

RS11099746 -0.60 0.7 -0.198 -0.05 0.7 -4.396 -0.26 0.9

RS6844434 -0.60 0.7 -0.198 -0.05 0.7 -4.396 -0.26 0.9

RS6535748 -0.60 0.7 -0.198 -0.05 0.7 -4.396 -0.26 0.9

RS9790390 -0.60 0.7 -0.198 -0.05 0.7 -4.396 -0.26 0.9

RS11723586 -0.60 0.7 -0.198 -0.05 0.7 -4.396 -0.26 0.9

RS998310 -0.55 0.7 -0.198 -0.05 0.7 -3.103 -0.19 0.8

RS2166596 -0.53 0.7 -0.198 -0.04 0.7 -2.553 -0.16 0.8

RS2602219 -0.52 0.7 -0.198 -0.04 0.7 -2.385 -0.15 0.8

RS12640152 -0.52 0.7 -0.198 -0.04 0.7 -2.385 -0.15 0.8

RS3822044 -0.52 0.7 -0.198 -0.04 0.7 -2.385 -0.15 0.8

RS7678266 -0.52 0.7 -0.198 -0.04 0.7 -2.385 -0.15 0.8

RS6815550 -0.52 0.7 -0.198 -0.04 0.7 -2.385 -0.15 0.8

RS6849438 -0.52 0.7 -0.198 -0.04 0.7 -2.385 -0.15 0.8

RS1350038 -0.52 0.7 -0.198 -0.04 0.7 -2.385 -0.15 0.8

RS13127289 -0.52 0.7 -0.198 -0.04 0.7 -2.385 -0.15 0.8

RS7656823 -0.52 0.7 -0.198 -0.04 0.7 -2.385 -0.15 0.8

RS1521491 -0.52 0.7 -0.198 -0.04 0.7 -2.385 -0.15 0.8

RS7692035 -0.52 0.7 -0.198 -0.04 0.7 -2.385 -0.15 0.8

RS7659292 -0.52 0.7 -0.198 -0.04 0.7 -2.385 -0.15 0.8

RS12643256 -0.52 0.7 -0.198 -0.04 0.7 -2.384 -0.15 0.8

RS1487565 -0.52 0.7 -0.198 -0.04 0.7 -2.385 -0.15 0.8

RS6537018 -0.52 0.7 -0.198 -0.04 0.7 -2.385 -0.15 0.8

RS1425431 -0.52 0.7 -0.198 -0.04 0.7 -2.385 -0.15 0.8

RS3851424 -0.52 0.7 -0.198 -0.04 0.7 -2.385 -0.15 0.8

RS12505705 -0.52 0.7 -0.198 -0.04 0.7 -2.385 -0.15 0.8

RS12650097 -0.52 0.7 -0.198 -0.04 0.7 -2.385 -0.15 0.8

RS4956344 -0.52 0.7 -0.198 -0.04 0.7 -2.385 -0.15 0.8

RS1550057 -0.52 0.7 -0.198 -0.04 0.7 -2.385 -0.15 0.8

RS6840028 -0.52 0.7 -0.198 -0.04 0.7 -2.385 -0.15 0.8

RS1057260 -0.52 0.7 -0.198 -0.04 0.7 -2.385 -0.15 0.8

RS1519551 -0.52 0.7 -0.198 -0.04 0.7 -2.385 -0.15 0.8

RS1995960 -0.52 0.7 -0.198 -0.04 0.7 -2.385 -0.15 0.8

RS336350 -0.52 0.7 -0.198 -0.04 0.7 -2.385 -0.15 0.8

RS336292 -0.52 0.7 -0.198 -0.04 0.7 -2.385 -0.15 0.8

RS2636670 -0.52 0.7 -0.198 -0.04 0.7 -2.385 -0.15 0.8

RS1425533 -0.52 0.7 -0.198 -0.04 0.7 -2.385 -0.15 0.8

RS975136 -0.52 0.7 -0.198 -0.04 0.7 -2.385 -0.15 0.8

RS3805236 -0.52 0.7 -0.198 -0.04 0.7 -2.385 -0.15 0.8

RS4423828 -0.52 0.7 -0.198 -0.04 0.7 -2.385 -0.15 0.8

RS7674504 -0.52 0.7 -0.198 -0.04 0.7 -2.385 -0.15 0.8

RS7677035 -0.52 0.7 -0.198 -0.04 0.7 -2.385 -0.15 0.8

RS7654947 -0.52 0.7 -0.198 -0.04 0.7 -2.385 -0.15 0.8

RS2353934 -0.52 0.7 -0.198 -0.04 0.7 -2.385 -0.15 0.8

RS813138 -0.52 0.7 -0.198 -0.04 0.7 -2.385 -0.15 0.8

RS11100872 -0.52 0.7 -0.198 -0.04 0.7 -2.385 -0.15 0.8

RS10006257 -0.52 0.7 -0.198 -0.04 0.7 -2.385 -0.15 0.8

RS4373182 -0.52 0.7 -0.198 -0.04 0.7 -2.385 -0.15 0.8

RS11100883 -0.52 0.7 -0.198 -0.04 0.7 -2.385 -0.15 0.8

RS1874572 -0.52 0.7 -0.198 -0.04 0.7 -2.385 -0.15 0.8

RS11934305 -0.52 0.7 -0.198 -0.04 0.7 -2.385 -0.15 0.8

RS4544728 -0.52 0.7 -0.198 -0.04 0.7 -2.385 -0.15 0.8

RS17020769 -0.52 0.7 -0.198 -0.04 0.7 -2.385 -0.15 0.8

RS1567224 -0.52 0.7 -0.198 -0.04 0.7 -2.385 -0.15 0.8

RS13125215 -0.52 0.7 -0.198 -0.04 0.7 -2.385 -0.15 0.8

RS6821225 -0.52 0.7 -0.198 -0.04 0.7 -2.385 -0.15 0.8

RS12374409 -0.52 0.7 -0.198 -0.04 0.7 -2.385 -0.15 0.8

RS1960018 -0.52 0.7 -0.198 -0.04 0.7 -2.385 -0.15 0.8

RS9784489 -0.52 0.7 -0.198 -0.04 0.7 -2.385 -0.15 0.8

RS13152024 -0.52 0.7 -0.198 -0.04 0.7 -2.385 -0.15 0.8

RS6813768 -0.52 0.7 -0.198 -0.04 0.7 -2.385 -0.15 0.8

RS10019569 -0.52 0.7 -0.198 -0.04 0.7 -2.385 -0.15 0.8

RS1561223 -0.52 0.7 -0.198 -0.04 0.7 -2.385 -0.15 0.8

RS10034640 -0.52 0.7 -0.198 -0.04 0.7 -2.385 -0.15 0.8

RS2357604 -0.52 0.7 -0.198 -0.04 0.7 -2.385 -0.15 0.8

RS6537478 -0.52 0.7 -0.198 -0.04 0.7 -2.385 -0.15 0.8

RS4835088 -0.52 0.7 -0.198 -0.04 0.7 -2.385 -0.15 0.8

RS9684012 -0.52 0.7 -0.198 -0.04 0.7 -2.385 -0.15 0.8

RS6815554 -0.52 0.7 -0.198 -0.04 0.7 -2.385 -0.15 0.8

RS7660368 -0.52 0.7 -0.198 -0.04 0.7 -2.385 -0.15 0.8

RS5534 -0.52 0.7 -0.198 -0.04 0.7 -2.385 -0.15 0.8

RS1403143 -0.52 0.7 -0.198 -0.04 0.7 -2.385 -0.15 0.8

RS6836191 -0.52 0.7 -0.198 -0.04 0.7 -2.385 -0.15 0.8

RS3846329 -0.52 0.7 -0.198 -0.04 0.7 -2.385 -0.15 0.8

RS3920217 -0.52 0.7 -0.198 -0.04 0.7 -2.385 -0.15 0.8

RS4635799 -0.52 0.7 -0.198 -0.04 0.7 -2.385 -0.15 0.8

RS6535612 -0.52 0.7 -0.198 -0.04 0.7 -2.385 -0.15 0.8

RS875758 -0.52 0.7 -0.198 -0.04 0.7 -2.385 -0.15 0.8

RS7437928 -0.52 0.7 -0.198 -0.04 0.7 -2.385 -0.15 0.8

RS10008892 -0.52 0.7 -0.198 -0.04 0.7 -2.385 -0.15 0.8

RS2204206 -0.52 0.7 -0.198 -0.04 0.7 -2.385 -0.15 0.8

RS7691878 -0.52 0.7 -0.198 -0.04 0.7 -2.385 -0.15 0.8

RS11946353 -0.52 0.7 -0.198 -0.04 0.7 -2.385 -0.15 0.8

RS7673586 -0.52 0.7 -0.198 -0.04 0.7 -2.385 -0.15 0.8

RS1503697 -0.52 0.7 -0.198 -0.04 0.7 -2.385 -0.15 0.8

RS17502597 -0.52 0.7 -0.198 -0.04 0.7 -2.385 -0.15 0.8

RS11726953 -0.52 0.7 -0.198 -0.04 0.7 -2.385 -0.15 0.8

RS6833562 -0.52 0.7 -0.198 -0.04 0.7 -2.385 -0.15 0.8

RS11099754 -0.52 0.7 -0.198 -0.04 0.7 -2.385 -0.15 0.8

RS4240358 -0.52 0.7 -0.198 -0.04 0.7 -2.385 -0.15 0.8

RS11944163 -0.52 0.7 -0.198 -0.04 0.7 -2.385 -0.15 0.8

RS7698239 -0.52 0.7 -0.198 -0.04 0.7 -2.385 -0.15 0.8

RS4455394 -0.52 0.7 -0.198 -0.04 0.7 -2.385 -0.15 0.8

RS10520104 -0.52 0.7 -0.198 -0.04 0.7 -2.385 -0.15 0.8

RS10033020 -0.52 0.7 -0.198 -0.04 0.7 -2.385 -0.15 0.8

RS11732407 -0.52 0.7 -0.198 -0.04 0.7 -2.385 -0.15 0.8

RS6846809 -0.52 0.7 -0.198 -0.04 0.7 -2.385 -0.15 0.8

RS10857265 -0.52 0.7 -0.198 -0.04 0.7 -2.385 -0.15 0.8

RS11737270 -0.52 0.7 -0.198 -0.04 0.7 -2.385 -0.15 0.8

RS17361160 -0.52 0.7 -0.198 -0.04 0.7 -2.385 -0.15 0.8

RS12644477 -0.52 0.7 -0.198 -0.04 0.7 -2.385 -0.15 0.8

RS10011174 -0.52 0.7 -0.198 -0.04 0.7 -2.385 -0.15 0.8

RS10020796 -0.52 0.7 -0.198 -0.04 0.7 -2.385 -0.15 0.8

RS4508863 -0.52 0.7 -0.198 -0.04 0.7 -2.385 -0.15 0.8

RS4276265 -0.52 0.7 -0.198 -0.04 0.7 -2.385 -0.15 0.8

RS10013266 -0.52 0.7 -0.198 -0.04 0.7 -2.385 -0.15 0.8

RS13131114 -0.52 0.7 -0.198 -0.04 0.7 -2.385 -0.15 0.8

RS12504106 -0.52 0.7 -0.198 -0.04 0.7 -2.385 -0.15 0.8

RS1607074 -0.52 0.7 -0.198 -0.04 0.7 -2.385 -0.15 0.8

RS4696392 -0.52 0.7 -0.198 -0.04 0.7 -2.385 -0.15 0.8

RS7691951 -0.52 0.7 -0.198 -0.04 0.7 -2.385 -0.15 0.8

RS1878285 -0.52 0.7 -0.198 -0.04 0.7 -2.385 -0.15 0.8

RS4696428 -0.52 0.7 -0.198 -0.04 0.7 -2.385 -0.15 0.8

RS9997214 -0.52 0.7 -0.198 -0.04 0.7 -2.385 -0.15 0.8

RS11723584 -0.52 0.7 -0.198 -0.04 0.7 -2.385 -0.15 0.8

RS11732163 -0.52 0.7 -0.198 -0.04 0.7 -2.385 -0.15 0.8

RS10030193 -0.52 0.7 -0.198 -0.04 0.7 -2.385 -0.15 0.8

RS13125518 -0.52 0.7 -0.198 -0.04 0.7 -2.385 -0.15 0.8

RS3864176 -0.52 0.7 -0.198 -0.04 0.7 -2.385 -0.15 0.8

RS17370297 -0.52 0.7 -0.198 -0.04 0.7 -2.385 -0.15 0.8

RS13150331 -0.52 0.7 -0.198 -0.04 0.7 -2.385 -0.15 0.8

RS11723258 -0.52 0.7 -0.198 -0.04 0.7 -2.385 -0.15 0.8

RS1878449 -0.52 0.7 -0.198 -0.04 0.7 -2.385 -0.15 0.8

RS12641646 -0.52 0.7 -0.198 -0.04 0.7 -2.385 -0.15 0.8

RS6835823 -0.52 0.7 -0.198 -0.04 0.7 -2.385 -0.15 0.8

RS9307919 -0.52 0.7 -0.198 -0.04 0.7 -2.385 -0.15 0.8

RS17031151 -0.52 0.7 -0.198 -0.04 0.7 -2.385 -0.15 0.8

RS4385038 -0.52 0.7 -0.198 -0.04 0.7 -2.385 -0.15 0.8

RS17031387 -0.52 0.7 -0.198 -0.04 0.7 -2.385 -0.15 0.8

RS4696207 -0.52 0.7 -0.198 -0.04 0.7 -2.385 -0.15 0.8
[truncated: 1,096,464 more chars]
